# Effects of preconception lifestyle intervention in infertile women with obesity: The FIT-PLESE randomized controlled trial

**DOI:** 10.1371/journal.pmed.1003883

**Published:** 2022-01-18

**Authors:** Richard S. Legro, Karl R. Hansen, Michael P. Diamond, Anne Z. Steiner, Christos Coutifaris, Marcelle I. Cedars, Kathleen M. Hoeger, Rebecca Usadi, Erica B. Johnstone, Daniel J. Haisenleder, Robert A. Wild, Kurt T. Barnhart, Jennifer Mersereau, J. C. Trussell, Stephen A. Krawetz, Penny M. Kris-Etherton, David B. Sarwer, Nanette Santoro, Esther Eisenberg, Hao Huang, Heping Zhang, for the Reproductive Medicine Network

**Affiliations:** 1 Department of Obstetrics and Gynecology, Penn State College of Medicine, Hershey, Pennsylvania, United States of America; 2 Department of Obstetrics and Gynecology, University of Oklahoma Health Sciences Center, Oklahoma City, Oklahoma, United States of America; 3 Department of Obstetrics and Gynecology, Augusta University, Augusta, Georgia, United States of America; 4 Department of Obstetrics and Gynecology, University of North Carolina, Chapel Hill, North Carolina, United States of America; 5 Department of Obstetrics and Gynecology, University of Pennsylvania, Philadelphia, Pennsylvania, United States of America; 6 Department of Obstetrics, Gynecology and Reproductive Sciences, University of California at San Francisco, San Francisco, California, United States of America; 7 Department of Obstetrics and Gynecology, University of Rochester, Rochester, New York, United States of America; 8 Department of Obstetrics and Gynecology, Atrium Health, Charlotte, North Carolina, United States of America; 9 Department of Obstetrics and Gynecology, University of Utah, Salt Lake City, Utah, United States of America; 10 Ligand Core Laboratory, University of Virginia Center for Research in Reproduction, Charlottesville, Virginia, United States of America; 11 Department of Urology, SUNY Upstate University Hospital, Syracuse, New York, United States of America; 12 Department of Obstetrics and Gynecology and Center for Molecular Medicine and Genetics, Wayne State University, Detroit, Michigan, United States of America; 13 Department of Nutritional Sciences, Penn State College of Health and Human Development, Pennsylvania, United States of America; 14 Center for Obesity Research and Education, College of Public Health, Temple University, Philadelphia, Pennsylvania, United States of America; 15 Department of Obstetrics and Gynecology, University of Colorado School of Medicine, Aurora, Colorado, United States of America; 16 Fertility and Infertility Branch, NICHD, Rockville, Maryland, United States of America; 17 Department of Biostatistics, Yale University, New Haven, Connecticut, United States of America; University of Manchester, UNITED KINGDOM

## Abstract

**Background:**

Women with obesity and infertility are counseled to lose weight prior to conception and infertility treatment to improve pregnancy rates and birth outcomes, although confirmatory evidence from randomized trials is lacking. We assessed whether a preconception intensive lifestyle intervention with acute weight loss is superior to a weight neutral intervention at achieving a healthy live birth.

**Methods and findings:**

In this open-label, randomized controlled study (FIT-PLESE), 379 women with obesity (BMI ≥ 30 kg/m^2^) and unexplained infertility were randomly assigned in a 1:1 ratio to 2 preconception lifestyle modification groups lasting 16 weeks, between July 2015 and July 2018 (final follow-up September 2019) followed by infertility therapy. The primary outcome was the healthy live birth (term infant of normal weight without major anomalies) incidence. This was conducted at 9 academic health centers across the United States. The intensive group underwent increased physical activity and weight loss (target 7%) through meal replacements and medication (Orlistat) compared to a standard group with increased physical activity alone without weight loss. This was followed by standardized empiric infertility treatment consisting of 3 cycles of ovarian stimulation/intrauterine insemination. Outcomes of any resulting pregnancy were tracked. Among 191 women randomized to standard lifestyle group, 40 dropped out of the study before conception; among 188 women randomized to intensive lifestyle group, 31 dropped out of the study before conception. All the randomized women were included in the intent-to-treat analysis for primary outcome of a healthy live birth. There were no significant differences in the incidence of healthy live births [standard 29/191(15.2%), intensive 23/188(12.2%), rate ratio 0.81 (0.48 to 1.34), *P* = 0.40]. Intensive had significant weight loss compared to standard (−6.6 ± 5.4% versus −0.3 ± 3.2%, *P* < 0.001). There were improvements in metabolic health, including a marked decrease in incidence of the metabolic syndrome (baseline to 16 weeks: standard: 53.6% to 49.4%, intensive 52.8% to 32.2%, *P* = 0.003). Gastrointestinal side effects were significantly more common in intensive. There was a higher, but nonsignificant, first trimester pregnancy loss in the intensive group (33.3% versus 23.7% in standard, 95% rate ratio 1.40, 95% confidence interval [CI]: 0.79 to 2.50). The main limitations of the study are the limited power of the study to detect rare complications and the design difficulty in finding an adequate time matched control intervention, as the standard exercise intervention may have potentially been helpful or harmful.

**Conclusions:**

A preconception intensive lifestyle intervention for weight loss did not improve fertility or birth outcomes compared to an exercise intervention without targeted weight loss. Improvement in metabolic health may not translate into improved female fecundity.

**Trial registration:**

ClinicalTrials.gov NCT02432209.

## Abstract 2

Richard Legro and colleagues investigate the impact of a preconception weight loss intervention on healthy live birth rates in women with obesity and unexplained infertility.

## Author summary

**Why was this study done?:**

- Obesity in women is associated with an increased time to pregnancy, pregnancy loss, and many pregnancy complications.
- Consequently, women who are obese and infertile are recommended to lose weight prior to conception to increase their chance for a healthy baby.
- There are few data from prospective trials to support this recommendation.

**What did the researchers do and find?:**

- In this study, we conducted an open-label, randomized controlled trial examining the effects of an intensive preconception intervention designed to lose weight acutely compared to a standard intervention designed to maintain weight consisting of exercise alone for 16 weeks prior to initiating routine infertility treatment in women with unexplained infertility in the US.
- We found that our intensive intervention resulted in about 7% weight loss in the intensive group versus no significant weight loss in the standard group.
- After the preconception intervention, both groups received 3 cycles of routine infertility treatment for both groups. There were no significant differences in cumulative pregnancy or live birth rates between groups.
- The small number of pregnancies reduced the statistical power for examining differences in the rate of pregnancy complications between groups.

**What do these findings mean?:**

- Our study supports other studies that have found no improved live birth or healthy live birth rates in women who were obese or overweight who participated in a preconception weight loss intervention prior to starting infertility therapy.
- Further research will be needed to investigate potential harms and benefits of weight loss interventions that did not reach statistical significance in the present trial.
- There is not strong evidence to recommend weight loss prior to conception in women who are obese with unexplained infertility.

## Introduction

Weight loss in women with obesity prior to pregnancy is thought to improve not only the chance for pregnancy but also a healthy live birth. Epidemiological evidence overwhelmingly supports a strong association between obesity and infertility [1], pregnancy loss [2–4], and an increased rate of maternal and fetal complications of pregnancy [5–7]. Consequently, experts, major medical societies, and public health programs have endorsed or mandated weight loss in women with obesity before initiating infertility therapy [8–10]. Data from case series [11], prospective randomized trials of preconception interventions in women with obesity [12,13], or from registry studies of women who have conceived after bariatric surgery [14,15] are less supportive of benefit. Some studies identified potential harms such as pregnancy loss after dietary caloric restriction [11,12] or increased rates of preterm delivery and/or small for gestational age (SGA) babies after bariatric surgery [15].

We designed a trial to test 2 preconception lifestyle interventions, one (intensive) with a multifocal approach of caloric restriction, weight loss medication, and increased physical activity to achieve clinically meaningful weight loss (7% target) and the second intervention (standard) primarily to increase activity without targeted weight loss [16]. The rationale is that previous preconception intervention studies have randomized patients to either immediate infertility treatment or intensive lifestyle intervention followed by infertility treatment [12,13], creating a lack of equipoise and a bias toward success with immediate infertility treatment. Increased physical activity alone was chosen as a comparator given the extensive literature summarizing this as an effective complementary therapy to infertility treatment [17]. Our hypothesis was that the intensive intervention was more likely to achieve a healthy term normal weight infant than standard intervention for women with obesity and unexplained infertility.

## Methods

### Study design and participants

The FIT-PLESE trial was a multicenter randomized controlled parallel group trial of 2 types of preconception lifestyle modification, one designed to lose weight and one to keep weight constant in which participants were randomly allocated in a 1:1 ratio. The FIT-PLESE protocol (S1 Protocol), designed by the steering committee of the Eunice Kennedy Shriver National Institute of Child Health and Human Development (NICHD) Reproductive Medicine Network, was based on previous studies of preinfertility treatment weight loss [18] and treatments of unexplained infertility [19] to create a 2 phase study, the first phase of lifestyle intervention and a standardized second phase of infertility treatment (ClinicalTrials.gov: NCT02432209). It was approved before study initiation by both an NICHD appointed advisory board and a data and safety monitoring board, which provided oversight. A single Institutional Review Board (IRB) at the University of Pennsylvania approved the study with administrative review by each site’s IRB. All participants (women and their male partners) gave written informed consent at 9 study sites across the US. Enrollment began in July 2015 and finished in July 2018.

A total of 379 women (ages 18 to 40 years) whose body mass index (BMI) was ≥30 kg/m^2^ were randomized. They were in good health, had a history of ≥1 year of infertility, and had regular ovulation (defined as ≥9 spontaneous menses per year) with normal ovarian reserve. A normal uterine cavity and at least 1 open fallopian tube were confirmed by sonohysterography, hysterosalpingography, a combined hysteroscopy and laparoscopy, or evidence of an unassisted intrauterine pregnancy within the immediate 3 years [19,20]. The male partner had at least 5 million total motile sperm in the ejaculate within 1 year of study initiation. Additionally, the couple agreed to comply with intercourse instructions and collection of semen for insemination.

### Randomization and masking

A SAS procedure (PROC PLAN) was used to generate the random allocation sequence. Randomization was stratified by study site and female BMI at baseline ≥40 with random block sizes of 2, 4, and 6. The random sequence was imported to a data management system owned and administrated by an investigative drug service company (Almac’s WebEZ system) and blinded to study investigators. The statisticians at the DCC generated the random allocation sequence. The investigators or nurse coordinators enrolled participants and assigned participants to intervention. Interventions were known to the investigator and patients.

### Procedures

This clinical trial compared 2 types of 16-week lifestyle modifications: one intensive, focusing on weight loss through increasing physical activity, caloric restriction, and anti-obesity medication and the other less intensive (standard), focused on increasing physical activity alone. In the second phase, both groups received 3 cycles of empiric ovarian stimulation with clomiphene citrate (CC) combined with intrauterine insemination.

The lifestyle modification interventions were adapted from gold standard interventions for obesity treatment [21] and used in other infertility studies [18]. The interventions were implemented in a manner that could be replicated in clinical practice. Both groups had identical on-site in person study visits (monthly during the first phase). Wireless devices were used to monitor physical activity (Fitbit Activity Monitors) and weight (Fitbit Aria Scale), and data were automatically uploaded to a central website accessible to study personnel. Women with infertility have been shown to be very compliant with tracking physical activity using Fitbit Activity Monitors [22].

The intensive group received nutritional counseling and meal replacement products (3 meals/day) to promote portion control and energy restriction (Nutrisystem) and a gastric lipase inhibitor (Orlistat, to reduce fat absorption) with a target of 7% weight loss. In addition, patients were instructed to consume 2 servings of fruit, 3 servings of vegetables, and 2 servings of low-fat dairy per day. This diet provided approximately 1,100 kcal/day with the following macronutrient profile: 30% calories from protein, 45% calories from carbohydrate; and 25% calories from fat. An additional 100 calories could be consumed as desired. This meal plan, totaling approximately 1,200 kcal/d, was consistent with that used in the Look AHEAD study [23]. Orlistat was initiated at a dose of 60 mg per meal at lunch and dinner. The morning dose was avoided due to the low-fat content of breakfast meal replacements. Patients in this group were also given a daily multivitamin supplement to be taken at least 2 hours before or after Orlistat to ensure adequate vitamin status.

Both groups received identical physical activity interventions. Patients used a Fitbit physical activity tracker during the screening phase to establish the mean number of steps over a 7-day period at baseline. They were instructed to increase steps by 500 steps a day per week until the upper limit of 10,000 steps a day was reached and then to maintain this rate throughout the study. Patients in the standard lifestyle group did not receive any dietary instruction or weight loss medication. An algorithm was created to ensure the weekly step increase in both groups (500 step/week up to but not exceeding 10,000 steps per week) and to avoid excessive weight loss. Weekly, weight and steps were monitored remotely by study coordinators by the Fitbit database and a twice monthly teleconference with a nutritionist (Dr. Kris-Etherton) and a psychologist (Dr. Sarwer) to troubleshoot management of noncompliant patients. Patients had intercourse ad lib during this intervention, and no contraception was prescribed. Meal replacements and Orlistat were discontinued after a positive pregnancy test.

Patients who did not conceive naturally during first phase received up to 3 cycles of ovarian stimulation/intrauterine insemination in the second phase. Both groups were advised to maintain weight and activity during this treatment period. CC was administered at a dose of 100 mg/d for 5 days starting on cycle Day 3 (±2 days).

The patients were monitored by transvaginal ultrasound after completing clomiphene. Visits were individualized based on follicular development until criteria for human chorionic gonadotropin (hCG) administration (at a dose of 10,000 U) were met [19]. The cycle was canceled if a leading follicle did not reach a mean diameter of 18 mm after 18 days, endogenous LH surge happened, or when more than 4 follicles developed (mean diameter >18 mm) to avoid the risk of ovarian hyperstimulation and/or high-order multiple gestations [19]. Doses were adjusted accordingly after cycle cancelation. One insemination was performed ≤44 hours after hCG administration. For inseminations, each site utilized its own standard semen preparation method and catheter [24].

A serum quantitative hCG pregnancy test was performed after menses, any positive home pregnancy test, or 2 weeks after insemination if a participant had no menses. Levels were followed for an appropriate rise. Transvaginal ultrasound documented the location of the pregnancy and number of implantation sites and was repeated to document fetal cardiac activity. Follow-up during pregnancy was then arranged with the treating obstetricians. Pregnancy outcomes were documented by review of maternal and neonatal records. Any patients who withdrew from the study without conception before the end of cycle 3 in Phase II were defined as dropouts.

### Outcomes

The primary outcome was a healthy birth outcome defined as defined as a live birth of an infant born at ≥37 weeks, with a birth weight between 2,500 and 4,000 g and without a major congenital anomaly.

Secondary outcomes included live birth (birth after 20 weeks) rate, time to pregnancy, pregnancy loss rate, multiple pregnancy rate, and pregnancy complication rate including development of gestational hypertension and diabetes, birth weight, and neonatal complication rate.

### Statistical analysis

Estimates used for the power calculation are based on our experience from previous trial [18,19]. We chose this narrower definition of a healthy birth outcome rather than live birth per se, as a healthy child is the patient, and provider-desired outcome and similar criteria have been used in previous trials [12,13]. We anticipated the proportion to be 0.25 in the standard lifestyle intervention arm and 0.40 in the intensive lifestyle modification arm. A sample size of 152 per treatment arm provided 80% power to detect a 0.15 absolute difference in the proportions of healthy births using a 2-sided test with a significance level of 0.05. The sample size was inflated to a total of 380 participants to allow for a 20% dropouts. The 20% dropout rate was used based on our experience in previous multicenter infertility trials [18–20,25].

All data entry, data management, and analyses were performed at the data coordinating center (DCC; Collaborative Center for Statistics in Science at Yale University).

The primary analyses used an intent-to-treat principle, wherein all randomized patients were analyzed according to their randomized treatment assignment, regardless of the actual treatment they received, protocol violations, or dropouts for the primary outcome. A chi-squared test (or Fisher exact test if any frequency count was <5) was used for testing differences between the 2 treatment groups for categorical variables and a Wilcoxon rank sum test used for continuous comparisons. All hypothesis tests were 2 sided, and all analyses were performed using SAS software, version 9.4 (SAS Institute, Cary, North Carolina, US) or R (open source). Statistical significance was defined as a 2-sided *P* value of less than 0.05. Cox proportional hazards models and the Kaplan–Meier method were applied to compare time to pregnancy and time to live birth in the treatment groups (S1 Fig). This study is reported as per the Consolidated Standards of Reporting Trials (CONSORT) guideline (S1 Checklist).

## Results

### Characteristics of the patients

**Fig 1:**
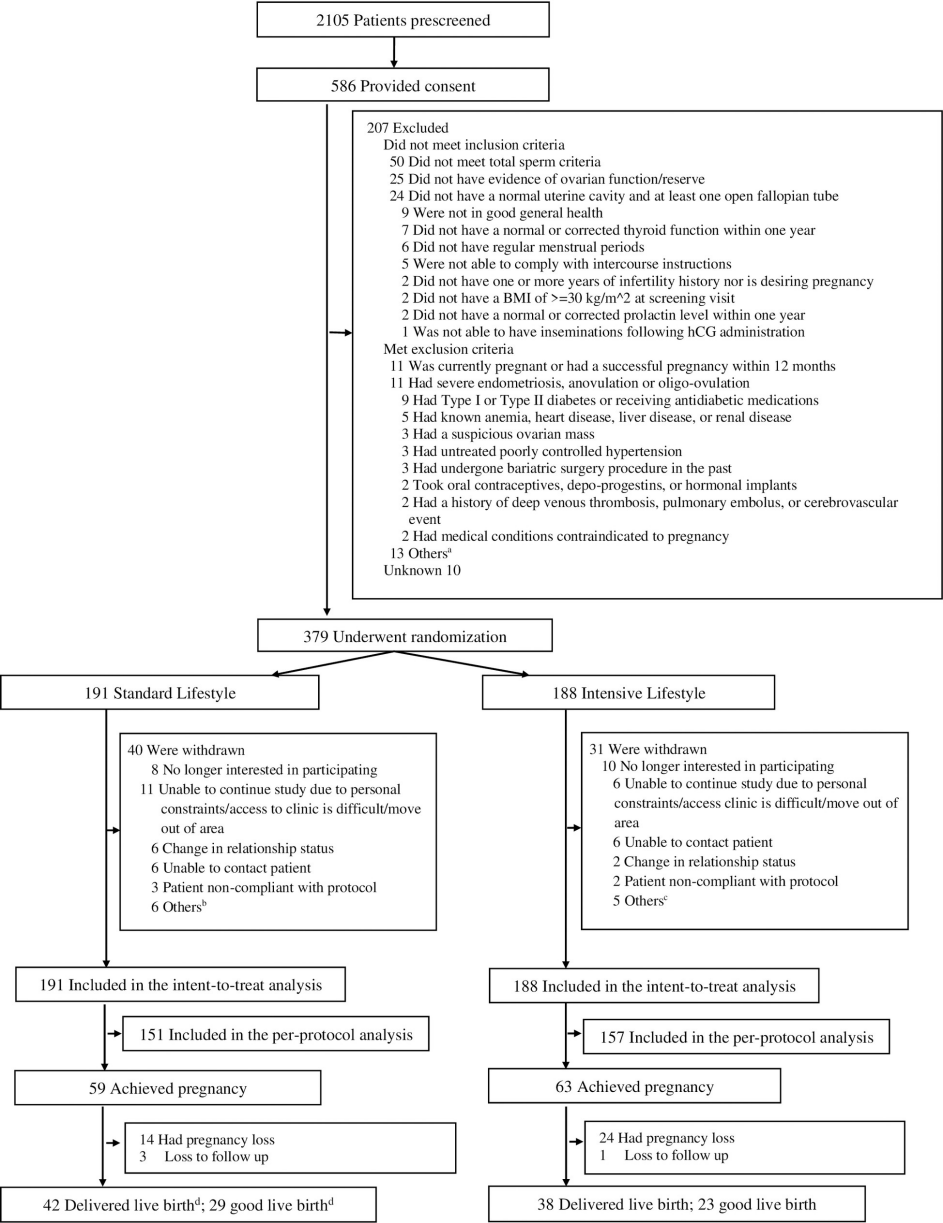
Flowchart: Enrollment and outcomes of the trial. ^a^One had a history of or suspected cervical/endometrial/or breast carcinoma; 1 had known Cushing disease, known or suspected adrenal or ovarian secreting tumors, or a history of gout; 1 had a history of alcohol abuse; 1 had an allergy, known hypersensitivity, or contraindication to the treatment medications; 1 had a presence of severe, untreated psychiatric illness; 1 had medical conditions that would be contraindicated to Orlistat, 1 had contraindication to study requirements including diet recommendation and activity requirements; 1 currently participating in lifestyle intervention program; and 1 in a period of acute weight loss or lost more than 5% body weight within the last 6 months. One participant or her male partner had previous sterilization procedures; 1 participant or her partner legally married to someone else; 1 using donated semen; and 1 have had pelvic radiation. ^b^One borderline HgA1C, 1 diagnosed with pulmonary embolism; 1 no response to Clomid; 1 patient developed a large complex cyst; 1 persistent cyst unable to start CC/IUI cycle; and 1 did not want to return for end of study visit. ^c^One patient had persistent ovarian cyst greater than 3 cm in size; 1 patient refused to see mental health provider regarding suicidal ideation; 1 PI discontinued patient from study; 1 patient’s spouse did not have adequate amount of sperm for IUI; and 1 husband did not want to proceed. ^d^Including 1 patient who withdrew the study due to the borderline HgA1C and later achieved pregnancy and delivered live twin babies. BMI, body mass index; CC/IUI, clomiphene citrate/intrauterine insemination; hCG, human chorionic gonadotropin; HgA1C, hemoglobin A1c; PI, principal investigator.

A total of 379 women were randomized to 1 of 2 treatment groups (Fig 1). The 2 groups were well matched at baseline (Table 1). A total of 40 of 191(20.9%) women withdrew from the standard lifestyle group and 31 of 188 (16.5%) from the intensive group (*P* = 0.267). There were no significant differences between groups for reasons of study withdrawal (S1–S8 Tables). The last patient was delivered in September of 2019.

**Table 1:** Baseline characteristics of the patients.

	Standard lifestyle	Intensive lifestyle
**Demographic**		
Age (y)	191	188
	32.4 ± 4.0	32.1 ± 4.5
	32.0 (30.0 to 35.0)	32.0 (29.0 to 35.0)
Level of education		
High school graduate or less	19/191 (9.9%)	20/188 (10.6%)
College graduate or some college	134/191 (70.2%)	138/188 (73.4%)
Graduate degree	38/191 (19.9%)	30/188 (16.0%)
Ethnicity		
Not Hispanic or Latino	177/191 (92.7%)	179/188 (95.2%)
Hispanic or Latino	9/191 (4.7%)	3/188 (1.6%)
Unknown	5/191 (2.6%)	6/188 (3.2%)
Race		
White	140/191 (73.3%)	126/188 (67.0%)
Black	37/191 (19.4%)	45/188 (23.9%)
Asian	3/191 (1.6%)	4/188 (2.1%)
American Indian or Alaska Native	2/191 (1.0%)	2/188 (1.1%)
Native Hawaiian or other Pacific Islander	0/191 (0.0%)	0/188 (0.0%)
Unknown	4/191 (2.1%)	5/188 (2.7%)
Mixed race	5/191 (2.6%)	6/188 (3.2%)
How long has the patient been attempting conception (months)?	188	187
	39.1 ± 33.0	38.7 ± 28.6
	25.0 (16.5 to 48.0)	24.0 (18.0 to 48.0)
Prior live birth	70/191 (36.6%)	57/187 (30.5%)
Current smoker	16/191 (8.4%)	16/188 (8.5%)
Weight (kg)	191	187
	107.4 ± 20.8	108.4 ± 22.7
	106.3 (91.8 to 119.9)	105.0 (92.9 to 120.3)
BMI at baseline (kg/m^2^)	191	188
	39.4 ± 6.9	39.2 ± 7.0
	38.1 (33.9 to 44.3)	37.8 (33.6 to 43.4)
Waist circumference at baseline (cm)	191	188
	115.3 ± 15.6	115.6 ± 15.6
	114.0 (103.5 to 125.0)	113.0 (104.1 to 123.5)
Systolic blood pressure (mm Hg)	189	187
	123.3 ± 11.4	123.7 ± 12.6
	122.0 (116.0 to 131.0)	124.0 (115.0 to 131.0)
Diastolic blood pressure (mm Hg)	190	187
	79.8 ± 10.6	80.0 ± 9.9
	80.0 (74.0 to 87.0)	81.0 (74.0 to 86.0)
Average steps per day at baseline (steps)	189	187
	6,945 ± 2,770	6,723 ± 2,501
	6,635 (4,845 to 8,708)	6,260 (4,869 to 8,091)
	**Standard lifestyle**	**Intensive lifestyle**
**Biochemical**		
Total testosterone (ng/dL)	189	185
	23.3 ± 15.2	26.1 ± 17.4
	18.5 (12.1 to 30.8)	20.7 (13.3 to 33.1)
SHBG (nmol/L)	190	185
	35.8 ± 18.9	39.0 ± 19.9
	32.9 (23.6 to 43.4)	34.3 (25.9 to 48.4)
Fasting glucose (mg/dL)	190	185
	95.4 ± 14.3	94.0 ± 13.6
	92.4 (84.7 to 104.2)	91.8 (85.3 to 99.3)
Fasting insulin (uIU/mL)	188	185
	17.9 ± 20.7	19.3 ± 16.9
	14.0 (9.1 to 19.8)	14.7 (9.5 to 22.8)
Leptin (ng/mL)	190	185
	77.9 ± 49.7	79.9 ± 49.8
	62.2 (44.6 to 85.7)	66.1 (48.5 to 86.2)
HbA1c (%)	172	169
	5.5 ± 0.4	5.4 ± 0.4
	5.4 (5.2 to 5.7)	5.4 (5.2 to 5.6)
Adiponectin (ng/mL)	190	185
	12,517 ± 8,323.9	11,409 ± 6,969.8
	10,518 (6,909.4 to 15,634)	9,746.6 (6,536.0 to 13,963)
hs-CRP (mg/L)	189	185
	7.5 ± 6.4	9.9 ± 13.7
	5.7 (2.8 to 10.1)	6.2 (2.9 to 13.1)
Total cholesterol (mg/dL)	183	176
	186.8 ± 35.0	187.3 ± 33.9
	183.0 (161.0 to 210.0)	183.5 (165.5 to 210.0)
Triglycerides (mg/dL)	183	176
	128.8 ± 62.5	130.0 ± 59.2
	118.0 (83.0 to 158.0)	117.0 (90.5 to 157.0)
HDL cholesterol (mg/dL)	183	176
	40.3 ± 9.0	40.1 ± 8.4
	39.0 (34.0 to 46.0)	39.5 (33.5 to 45.0)
**Integrated biometric and biochemical**		
Metabolic syndrome	98/183 (53.6%)	93/176 (52.8%)
**Questionnaire**		
Total score of fertility QoL^a^	188	184
	75.7 ± 13.1	78.0 ± 12.6
	77.1 (66.7 to 85.9)	80.7 (69.8 to 87.5)
**Male partner**		
Age (y), male^b^	191	187
	34.9 ± 5.8	33.5 ± 5.3
	35.0 (30.0 to 39.0)	33.0 (30.0 to 37.0)
BMI (kg/m^2^), male	191	188
	32.8 ± 7.5	32.5 ± 7.7
	32.3 (26.9 to 37.2)	30.9 (27.5 to 36.0)
Total motile sperm (million)	191	188
	86.4 ± 154.6	77.7 ± 85.3
	44.5 (21.6 to 105.3)	46.9 (21.5 to 94.7)

Continuous variables are shown as *n* (top), mean ± SD (middle), and median (interquartile range) (bottom). Categorical variables are shown as no./total *n* (%).

^a^Score ranges from 0 to 100, with higher scores indicating better quality of life.

^b^*P* = 0.025 for the comparison between the 2 groups.

BMI, body mass index; HbA1c, hemoglobin A1c; HDL, high-density lipoprotein; hs-CRP, high-sensitivity C-reactive protein; QoL, Quality of Life; SHBG, sex hormone–binding globulin.

### Effects of preconception intervention

Both groups significantly increased their step counts compared to baseline; there was no signficant difference between groups. Weight loss was significantly greater in the intensive group and approached our target of 7% (−6.6 ± 5.4%) compared to the standard group (*P* < 0.001), which did not experience significant weight loss (Table 2). A significantly greater proportion achieved a 5% and 10% weight loss in the intensive group than in the standard group (*P* < 0.001). Compared to the standard, the intensive lifestyle group experienced significant improvements in multiple metabolic and reproductive parameters, both biometric (decreased blood pressure and waist circumference) and biochemical (decreased total testosterone, insulin, glycohemoglobin, leptin, hsCRP, and triglycerides and increased sex hormone–binding globulin and adiponectin levels). The incidence of the metabolic syndrome decreased significantly in the intensive group compared to the standard group (*P* = 0.003). There were no significant differences between groups in quality of life measures and multiple other measures (S1–S8 Tables).

**Table 2:** Effect of 16-week lifestyle intervention programs on biometric and biochemical parameters.

	Standard lifestyle	Intensive lifestyle	*P* value^b^
**Biometric**			
Change in average steps per day (steps)	187	185	
	1,819 ± 2,844	2,022 ± 2,794	
	1,732 (61 to 3,840)	2,108 (140 to 4,245)	0.430
Absolute change BMI (kg/m^2^)	184	180	
	−0.1 ± 1.3	−2.6 ± 2.1	
	−0.1 (−0.8 to 0.6)	−2.3 (−4.2 to −1.0)	<0.001
Absolute change in weight (kg)	184	180	
	−0.3 ± 3.4	−7.3 ± 6.0	
	−0.3 (−2.2 to 1.5)	−6.4 (−11.4 to −2.9)	<0.001
Percentage of weight (%)	184	180	
	−0.3 ± 3.2	−6.6 ± 5.4	
	−0.3 (−2.0 to 1.5)	−6.2 (−10.3 to −2.9)	<0.001
Weight loss by 5% or more	12/184 (6.5%)	107/180 (59.4%)	<0.001
Weight loss by 10% or more	2/184 (1.1%)	48/180 (26.7%)	<0.001
Absolute change in systolic blood pressure	182	180	
	−1.1 ± 12.7	−3.1 ± 11.6	
	0.0 (−9.0 to 8.0)	−4.5 (−10.5 to 5.0)	0.064
Absolute change in diastolic blood pressure	183	180	
	0.3 ± 10.8	−1.8 ± 10.3	
	1.0 (−6.0 to 5.0)	−2.0 (−8.5 to 4.0)	0.012
Absolute change in waist circumference (cm)	184	180	
	−0.8 ± 6.8	−7.7 ± 8.4	
	−1.0 (−5.0 to 3.0)	−7.0 (−12.0 to −2.3)	<0.001
**Biochemical**			
Total testosterone (ng/dL)	156	146	
	1.1 ± 14.0	−3.6 ± 13.1	
	0.0 (−7.9 to 9.1)	−3.5 (−11.3 to 2.5)	0.002
SHBG (nmol/L)	159	149	
	1.5 ± 14.1	4.6 ± 14.2	
	0.6 (−5.3 to 6.3)	2.5 (−3.6 to 13.4)	0.018
Fasting insulin (uIU/mL)	158	149	
	−1.2 ± 22.1	−4.1 ± 15.0	
	0.0 (−3.9 to 3.7)	−3.7 (−7.6 to 0.3)	<0.001
Leptin (ng/mL)	160	149	
	−5.1 ± 37.6	−29.0 ± 43.0	
	−2.4 (−20.0 to 13.5)	−21.1 (−38.2 to −6.4)	<0.001
	133	125	
HgbA1c (%)	0.09 ± 0.33	−0.03 ± 0.25	
	0.10 (−0.10 to 0.20)	0.00 (−0.20 to 0.10)	0.005
Adiponectin (ng/mL)	160	149	
	−324.6 ± 7,402.0	1,900.0 ± 6,333.8	
	114.7 (−2,389 to 2,813.2)	1,555.0 (−500.5 to 4,751.0)	0.001
hs-CRP (mg/L)	159	149	
	0.4 ± 5.2	−2.3 ± 14.1	
	0.3 (−1.6 to 2.1)	−1.1 (−2.9 to 0.6)	<0.001
	**Standard lifestyle**	**Intensive lifestyle**	***P* value**
Triglycerides (mg/dL)	152	136	
	−2.1 ± 50.3	−15.9 ± 48.9	
	−1.0 (−25.0 to 18.5)	−15.0 (−35.0 to 3.5)	0.005
**Integrated biochemical and biometric**			
Prevalence of the metabolic syndrome	78/158 (49.4%)	46/143 (32.2%)	0.003
**Questionnaire**			
Total score of fertility QoL^a^	161	146	
	−0.8 ± 9.0	−1.6 ± 10.2	
	0.0 (−6.3 to 5.2)	−0.4 (−7.3 to 5.2)	0.670

Continuous variables are shown as *n* (top), mean ± SD (middle), and median (interquartile range) (bottom). Categorical variables are shown as no./total *n* (%) at the end of the intervention. Data reported are the change from baseline reported in Table 1.

^a^Score ranges from 0 to 100, with higher scores indicating better quality of life.

^b^*P* values were calculated using chi-squared or Fisher exact test for categorical variables and Wilcoxon rank sum test for continuous variables.

BMI, body mass index; hs-CRP, high-sensitivity C-reactive protein; QoL, Quality of Life; SHBG, sex hormone–binding globulin.

### Healthy live births and secondary outcomes

The rate of having a healthy live birth was not significantly different between groups (S1 Fig, Table 3). Similarly, there was no significant difference in the rates of live births, multiple pregnancies, or the time to live birth. Pregnancy loss was greater in the intensive group, although not statistically significant. Duration of pregnancy, rate of cesarean section, and birth weight in grams were similar between the 2 groups. Per protocol analysis, likewise found no signficant differences (S1–S8 Tables). There were no significant differences in the pregnancy rates by time of conception during the study (S1–S8 Tables, S5 Fig). A post hoc BMI tertile analysis of the groups did not find any significant subgroup benefit of either lifestyle intervention on the primary outcome (S2 Fig) or having a live birth (S3 Fig). Results were similar with no statistically significant interaction when the patients were stratified by male partner age (S8 Table).

**Table 3:** Pregnancy outcomes according to intervention groups.

	Standard lifestyle	Intensive lifestyle	Rate ratio in intensive lifestyle group (95% CI)	*P* value^a^
Good live birth	29/191 (15.2%)	23/188 (12.2%)	0.81 (0.48 to 1.34)	0.404
Live birth	42/191 (22.0%)	38/188 (20.2%)	0.92 (0.62 to 1.36)	0.672
Singleton live birth	39/42 (92.9%)	32/38 (84.2%)	0.91 (0.77 to 1.07)	0.296
Twin live birth	3/42 (7.1%)	6/38 (15.8%)	2.21 (0.59 to 8.23)	0.296
Birth weight, grams	*N* = 42	*N* = 36		
	3,105.9 ± 794.4	3,198.9 ± 711.7		
	3,189.3 (2,636.5 to 3,671.3)	3,217.7 (2,802.4 to 3,642.9)		0.952
Low birth weight (<2,500 g)	8/42 (19.0%)	5/36 (13.9%)	0.73 (0.26 to 2.03)	0.542
High birth weight (>4,000 g)	5/42 (11.9%)	4/36 (11.1%)	0.93 (0.27 to 3.22)	1.000
Duration of pregnancy, weeks	*N* = 42	*N* = 36		
	37.8 ± 2.6	38.2 ± 1.7		
	38.8 (37.0 to 39.0)	38.3 (37.0 to 39.5)		0.984
Method of delivery				
Vaginal birth	17/40 (42.5%)	17/36 (47.2%)	1.11 (0.67 to 1.83)	0.679
Cesarean section	23/40 (57.5%)	19/36 (52.8%)	0.92 (0.61 to 1.38)	0.679
Conception	59/191 (30.9%)	63/188 (33.5%)	1.08 (0.81 to 1.45)	0.585
Time to conception, days	*N* = 52	*N* = 59		
	158.1 ± 80.1	148.6 ± 62.4		
	163.0 (98.5 to 214.0)	160.0 (102.0 to 190.0)		0.564
Clinical pregnancy	47/191 (24.6%)	52/188 (27.7%)	1.12 (0.80 to 1.58)	0.499
Pregnancy	45/191 (23.6%)	48/188 (25.5%)	1.08 (0.76 to 1.54)	0.656
Singleton pregnancy	42/45 (93.3%)	41/48 (85.4%)	0.92 (0.80 to 1.05)	0.319
Twin pregnancy	3/45 (6.7%)	7/48 (14.6%)	2.19 (0.60 to 7.95)	0.319
Sex ratio at birth (boys: girls)	0.88 (21:24)	0.76 (19:25)	0.87 (0.38 to 2.00)	0.741
Pregnancy loss among women who conceived	14/59 (23.7%)	24/63 (38.1%)	1.61 (0.92 to 2.80)	0.087
Loss in first trimester	14/59 (23.7%)	21/63 (33.3%)	1.40 (0.79 to 2.50)	0.241
Biochemical	6/59 (10.2%)	7/63 (11.1%)	1.09 (0.39 to 3.06)	0.866
Miscarriage	3/59 (5.1%)	10/63 (15.9%)	3.12 (0.90 to 10.79)	0.077
Ectopic pregnancy	4/59 (6.8%)	2/63 (3.2%)	0.47 (0.09 to 2.46)	0.428
Pregnancy of unknown location	1/59 (1.7%)	2/63 (3.2%)	1.87 (0.17 to 20.12)	1.000
	**Standard lifestyle**	**Intensive lifestyle**	**Rate ratio in intensive lifestyle group (95% CI)**	***P* value** ^a^
Loss in second or third trimester	0/59 (0.0%)	3/63 (4.8%)	NA	0.245

Live birth was defined by the delivery of a live-born infant. Good live birth was defined by the delivery of a live birth of an infant born at ≥37 weeks, with a birth weight between 2,500 and 4,000 g and without a major congenital anomaly. Conception was defined as having a rising serum level of hCG for 2 consecutive tests. Clinical pregnancy was defined by the observation of gestational sac on ultrasound. Pregnancy was defined by observation of fetal heart motion on ultrasonography. Miscarriage was defined as the loss of a clinical pregnancy.

^a^*P* values were calculated with the use of the chi-squared test or Fisher exact test for categorical data and the Wilcoxon rank sum test for continuous data.

CI, confidence interval; hCG, human chorionic gonadotropin; NA, not applicable.

### Adverse events and pregnancy and neonatal complications

Four serious adverse events occurred in the standard group and 3 in the intensive group with no clear relationship to the interventions (Table 4). Gastrointestinal adverse events, specifically diarrhea, oily stools or discharge, and flatulence, were significantly more common in the intensive group (consistent with acknowledged side effects of Orlistat). While we were not able to demonstrate a statistically significant benefit to preconception weight loss on later perinatal complications, most major pregnancy (i.e., preterm labor, premature rupture of membranes [PROM], preeclampsia, and gestational diabetes) and neonatal (intrauterine growth restriction [IUGR] and neonatal intensive care unit [NICU] admission) morbidities had nonsignificant rate improvements in the intervention group (Table 4). The list of adverse events occurring at a frequency ≥2% is in S5 Table (S1–S8 Tables).

**Table 4:** Serious adverse events (all) and adverse events (with more than 2% of patients experiencing them) between the intervention groups.

	Standard lifestyle	Intensive lifestyle	*P* value^a^
**Before conception**			
**Serious adverse**			
Hospitalization	0/191	2/188 (1.1%)	0.245
Pelvic pain	0/191	1/188 (0.5%)	0.496
Appendicitis	1/191 (0.5%)	0/188	1.000
Pneumonia	1/191 (0.5%)	0/188	1.000
Pulmonary embolism	1/191 (0.5%)	0/188	1.000
Complex cyst resulting in surgical intervention	1/191 (0.5%)	0/188	1.000
**Other adverse events**			
Constipation	7/191 (3.7%)	23/188 (12.2%)	0.002
Diarrhea	9/191 (4.7%)	35/188 (18.6%)	<0.001
Fever	0/191	5/188 (2.7%)	0.029
Flatulence	2/191 (1.0%)	33/188 (17.6%)	<0.001
Mood swings	9/191 (4.7%)	2/188 (1.1%)	0.062
Nausea/vomiting	24/191 (12.6%)	41/188 (21.8%)	0.017
Oily stools/discharge	0/191	43/188 (22.9%)	<0.001
**After conception**			
**Serious adverse events—mother**			
Hospitalization during first trimester	0/59	1/63 (1.6%)	1.000
Ectopic pregnancy	2/59 (3.4%)	1/63 (1.6%)	0.610
Pregnancy of unknown location	3/59 (3.4%)	3/63 (4.8%)	1.000
Marginal placenta previa	0/59	1/63 (1.6%)	1.000
Placenta previa and preterm birth	1/59 (1.7%)	0/63	0.484
Hospitalization	1/59 (1.7%)	2/63 (3.2%)	1.000
**Other adverse events—mother**			
Preterm labor	6/59 (10.2%)	2/63 (3.2%)	0.154
Preeclampsia/eclampsia	7/59 (11.9%)	4/63 (6.3%)	0.352
Gestational diabetes	10/59 (16.9%)	6/63 (9.5%)	0.225
Incompetent cervix	0/59	2/63 (3.2%)	0.496
PROM	4/59 (6.8%)	2/63 (3.2%)	0.428
Other complication	3/59 (5.1%)	4/63 (6.3%)	1.000
Placental abnormalities	4/59 (6.8%)	5/63 (7.9%)	1.000
Postpartum infection	2/59 (3.4%)	0/63	0.232
Postpartum hemorrhage	1/59 (1.7%)	0/63	0.484
Other postpartum complication(s)	1/59 (1.7%)	3/63 (4.8%)	0.620
**Serious adverse events—fetus/infant**			
Hospitalization—infant	2/42 (4.8%)	1/38 (2.6%)	1.000
Myelomeningocele	0/42	1/38 (2.6%)	0.475
Neonatal death	0/42	0/38	
Stillbirth	0/42	0/38	
**Other adverse events—fetus/infant**			
	**Standard lifestyle**	**Intensive lifestyle**	***P* value**
IUGR	4/42 (9.5%)	1/38 (2.6%)	0.362

^a^*P* value was calculated using chi-squared or Fisher exact test.

IUGR, intrauterine growth restriction; PROM, premature rupture of membranes.

## Discussion

In women with both obesity and unexplained infertility, an intensive preconception lifestyle intervention with an average weight loss of 7% did not improve the rate of having a healthy live birth or any live birth compared to an activity based intervention that was weight neutral. Likewise, there was no improvement in pregnancy rates, time to pregnancy, or birth weight. These results were unexpected despite improved cardiometabolic indicators after weight loss. These findings support that weight loss per se and improved cardiometabolic health obtained through preconception intervention do not guarantee improved pregnancy outcomes.

Our results are similar to 2 recent high-quality clinical trials of preconception weight loss interventions for women with obesity prior to infertility treatments. A trial conducted in the Netherlands found a significantly lower probability of live birth in infertile women treated with a 6-month preconception lifestyle intervention compared with proceeding immediately to infertility treatment [12]. A trial conducted in Sweden, which evaluated a 12-week preconception intervention, albeit with a more aggressive caloric restriction prior to in vitro fertilization (IVF), found no benefit for weight loss on the live birth rate compared to those patients who did not lose weight and immediately underwent IVF [13].

Despite similar outcomes, our trial offered several novel strengths. First, our trial only included women with unexplained infertility with the assumption that obesity per se is a significant infertility factor and did not include patients with other known infertility factors such as tubal disease or anovulation. Second, unlike our trial, neither of the 2 trials referenced provided a comparator treatment. The nonintervention groups went immediately to infertility treatment, thus skewing the nonintervention group toward a shorter time to pregnancy and the benefit of a longer period to receive infertility treatment. We sought moderate weight loss utilizing a multifocal approach with treatments transferable to the clinic and wireless digital devices to achieve and monitor compliance. Our patients achieved an average weight loss intermediate between the Dutch and Swedish studies. Differences to note are that the Dutch women lost weight over a comparatively longer period (only 37% achieved the targeted ≥5% weight loss), and the Swedish women lost excessive weight over a comparatively short time period (55% achieved a ≥10% weight loss). Nevertheless, there was a similar lack of benefit.

There are potential concerns with preconception lifestyle interventions that small trials, such as ours and the others, may be underpowered to detect. However, each of the studies including ours found more pregnancy loss in the weight loss intervention groups (albeit not significant for any individual trial). Pooling the results did indicate more miscarriages (relative risk 1.79, 95% confidence interval [CI]: 1.20 to 2.67). Pooled results are in S6 Table (63/620 or 10.0% in the weight loss group versus 35/628 or 5.6% in the nonweight loss groups) (S6 Table). Our results indicate that these losses tended to occur after implantation and ultrasound visualization of the gestational sac (S4 Fig) and were more likely to occur in later rather than earlier cycles of infertility treatment (*P* = 0.049) (S1–S8 Tables). It is possible that in our studies and others, vitamin or micronutrient deficiency, including decreased long-chain polyunsaturated fatty acid absorption, may have contributed to pregnancy loss. While we did not assess dietary intake and composition throughout the study, participants in the intensive intervention group received a multivitamin supplement during the intervention phase as commonly done with the use of Orlistat, and both groups were prescribed a prenatal vitamin upon randomization. We also chose our meal replacements as they were of high quality and nutritionally balanced.

Another strength of our study is that we collected not only pregnancy outcomes, but also all maternal and neonatal complications after conception. Future studies, including individual patient data meta-analyses, tracking these outcomes will better illuminate the effects of preconception weight loss. The best available epidemiologic evidence of the mixed effects of significant preconception weight loss on pregnancy morbidities comes from Swedish national registry reports, where women who underwent bariatric surgery had a lower rate of gestational diabetes, but significantly higher rates of spontaneous preterm delivery and SGA babies compared to women without obesity [15]. The weight loss mechanism differs after different types of bariatric surgery, and more significant weight loss usually occurs after surgery compared to our intervention with a corresponding greater chance for malabsorption.

The present investigation was underpowered to address these other perinatal outcomes. Further studies and the use of individual patient data meta-analysis may be necessary to achieve the necessary numbers required to see differences in rare but severe morbidities related to obesity and pregnancy. In our study, birth weight (which did not differ between the lifestyle groups) may be the best integrated marker of perinatal health [26,27]. Our findings may only be specific to women with unexplained infertility as opposed to other disorders such as anovulation due to polycystic ovary syndrome [18]. Our live birth rates were also significantly less than we expected, presumably due to both the severity of obesity in our patient population and the comparative ineffectiveness of our frontline infertility therapies.

There are many avenues for future research. Other interventions of varying duration and/or intensity prior to conception may yield more favorable outcomes. A period of weight stabilization and maintenance after a weight loss intervention prior to commencing infertility therapy is worth exploring. However, this must be balanced against the desire of the couple to have a baby as soon as possible and their unwillingness to delay meaningful treatment. Recruitment and retention into such a trial with prolonged participation and delayed treatment may be difficult. Developing better comparators for weight loss interventions, beyond exercise or observation (which the couple may interpret as doing nothing), is another option. Alternate trial designs and comparators must provide equipoise to all participants.

Our findings directly impact current standards of clinical care, where women who are obese with unexplained infertility are to our knowledge routinely counseled to lose weight prior to initiation of infertility treatment. Presently, there is no Level I evidence to support the recommendation that preconception weight loss in women with obesity and unexplained infertility prior to treatment leads to either a higher chance of a healthy live birth or a shorter time to pregnancy.

In conclusion, we have demonstrated that we can achieve significant weight loss and improvement in cardiometabolic health through an intensive lifestyle intervention in women who are obese with unexplained infertility in a reasonably short time period of 16 weeks. This does not translate to a shortened time to pregnancy, an improved live birth, or healthy live birth rate. Improved weight and female cardiometabolic health may not equal improved fecundity.

## Supporting information

S1 Protocol Protocol containing the original protocol, final protocol, and summary of changes. (PDF)

S1 Table Dropout or study exclusion reasons by study time point. (DOCX)

S2 Table Change in hormones from baseline after 16-week preconception intervention. (DOCX)

S3 Table Pregnancy outcomes—per protocol analysis. (DOCX)

S4 Table Good live birth, live birth, and conception rates per study phase and treatment cycle. (DOCX)

S5 Table Serious adverse events (all) and adverse events (with more than 2% of patients experiencing them) between the intervention groups. (DOCX)

S6 Table Pregnancy loss of selected lifestyle intervention trials in women with infertility. (DOCX)

S7 Table Pregnancy loss according to the time point of achieved conception (Phase I, cycle 1 in Phase II versus cycle 2, and 3 in Phase II). (DOCX)

S8 Table Good live birth, live birth, and conception according to male partner age. (DOCX)

S1 Fig Kaplan–Meier curves for good live birth and live birth. Good live birth rate is shown according to treatment group in panel A, and live birth rate is shown in panel B.
(TIF)

S2 Fig Kaplan–Meier curves for good live birth. Good live birth rates are shown according to treatment group and maternal BMI (the weight in kilograms divided by the square of the height in meters) in panels A, B, and C. BMI, body mass index.
(TIF)

S3 Fig Kaplan–Meier curves for live birth. Live birth rates are shown according to treatment group and maternal BMI (the weight in kilograms divided by the square of the height in meters) in panels A, B, and C. BMI, body mass index.
(TIF)

S4 Fig Kaplan–Meier curves for pregnancy loss among those who conceived. Pregnancy loss rates are shown according to treatment groups.
(TIF)

S5 Fig Good live birth, live birth, and conception per study phase and treatment cycles. *P* value was for the testing of the difference across the study phase and treatment cycles using the generalized linear model.
(TIF)

S1 Checklist CONSORT Checklist. CONSORT, Consolidated Standards of Reporting Trials.
(DOC)

## Abbreviations

BMI: body mass index
CC: clomiphene citrate
CI: confidence interval
CONSORT: Consolidated Standards of Reporting Trials
DCC: data coordinating center
hCG: human chorionic gonadotropin
IRB: Institutional Review Board
IUGR: intrauterine growth restriction
IVF: in vitro fertilization
NICHD: National Institute of Child Health and Human Development
NICU: neonatal intensive care unit
PROM: premature rupture of membranes
SGA: small for gestational age
