# Supplementary material for: Effects of preconception lifestyle intervention in infertile women with obesity: The FIT-PLESE randomized controlled trial
Source: PLoS Med. 2022 Jan 18;19(1):e1003883. doi: 10.1371/journal.pmed.1003883 (PMC8765626; doi:10.1371/journal.pmed.1003883)
Supplement: S1 Protocol — (PDF) [file pmed.1003883.s001.pdf]

Protocol

This supplement contain the following:

1. Original protocol
2. Final prototol
3. Summary of changes

Reproductive Medicine Network
Funded by *Eunice Kennedy Shriver* NICHD

Improving Reproductive Fitness through Pretreatment with Lifestyle Modification in Obese Women with Unexplained Infertility: (FIT-PLESE FEMALE)

Protocol Leader: Richard S. Legro, MD
Data and Coordination Leader: Heping Zhang, PhD

Version 2.0

Supported by:
The Eunice Kennedy Shriver National Institute of Child Health and Human Development (NICHD)

Supported by Grants U10 HD38992 (to R.S.L.), U10 HD27049 (to C.C.), U10HD055925 (to H.Z.), U10 HD39005 (to M.P.D.), U10 HD077680 (to K.H.), U10HD077844 (to A.S.), and U10HD077841 (to M.C)

Study Intervention Provided by: No outside support
Sponsor of IND(IDE): Not Applicable

Prepared by:

The RMN Data Coordination Center

Collaborative Center for Statistics in Science

Yale University School of Public Health

300 George Street, Suite 523

New Haven, CT 06511

(203) 785-5185

Last updated: August 18, 2015

Protocol Subcommittee

Group email address: ***rmn.fit@mailman.yale.edu***

Name	E-mail
**Richard Legro, MD**	**rsl1@psu.edu**
Esther Eisenberg, MD, MPH	eisenbes@mail.nih.gov
Heather Huddleston, MD	HuddlestonH@obgyn.ucsf.edu
Penny Kris-Etherton	pmk3@psu.edu
Alex Polotsky, MD	*Alex.polotsky@ucdenver.edu*
Nanette Santoro, MD	nanette.santoro@ucdenver.edu
David Sarwer, PhD	dsarwer@mail.med.upenn.edu
Peter Snyder, MD	pjs@mail.med.upenn.edu
Anne Z. Steiner, MD, MPH	anne_steiner@med.unc.edu
J. C. Trussell, MD	jctrussell1@verizon.net
Heping Zhang, PhD	heping.zhang@yale.edu

Table of Contents

1 STUDY SYNOPSIS 10

1.1 OBJECTIVES 10
1.2 HYPOTHESIS 10
1.3 PATIENT POPULATION 10
1.4 STUDY DESIGN 10
1.5 TREATMENT 10
1.6 PRIMARY EFFICACY PARAMETER 11
1.7 SECONDARY EFFICACY PARAMETERS 11
1.8 STATISTICAL ANALYSIS 11
1.9 ANTICIPATED TIME TO COMPLETION 12
1.10 REGULATORY COMPLIANCE 12

2 STUDY OBJECTIVES 13

2.1 PRIMARY AIM 13
2.2 SECONDARY AIM 13
2.3 TERTIARY AIMS 13

3 BACKGROUND 14

3.1 RATIONALE 14
3.2 OBESITY AND REPRODUCTION 14
3.3 ADVERSE EFFECTS OF LIFESTYLE AND WEIGHT LOSS ON PREGNANCY 15
3.4 CURRENT STATUS OF OBESITY MANAGEMENT 16
3.5 GOOD BIRTH OUTCOME 18
3.6 SUPPORTING DATA 19
3.7 SUMMARY OF AMIGOS DATA 25

4 STUDY DESIGN 28

4.1 OVERVIEW 28
4.1.1 Treatment Design 29
4.1.2 Study Population 29

5 SELECTION AND ENROLLMENT OF SUBJECTS 30

5.1 INCLUSION CRITERIA 30
5.2 EXCLUSION CRITERIA 30
5.3 STUDY TERMINATION CRITERIA 31
5.4 STUDY ENROLLMENT PROCEDURES 32
5.4.1 Recruitment 32
5.5 PROCEDURES FOR TRACKING SOURCES OF SUBJECTS AND THEIR DISPOSITION 33
5.6 OBTAINING INFORMED CONSENT 33
5.7 INTERVENTION GROUP ASSIGNMENT 34

6 STUDY INTERVENTIONS 35

6.1 INTERVENTIONS, ADMINISTRATION AND DURATION 35
6.1.1 Determine Baseline Physical Activity 36
6.2 HANDLING OF STUDY INTERVENTIONS 37
6.3 CONCOMITANT INTERVENTIONS 37
6.4 ADHERENCE ASSESSMENT 37

7 CLINICAL AND LABORATORY EVALUATIONS 38

7.1 SCHEDULE OF EVALUATIONS 38
7.2 TIMING OF EVALUATIONS 40
7.3 SPECIAL INSTRUCTIONS AND DEFINITIONS OF EVALUATIONS 49
7.3.1 History and Demographics 49

7.3.2 *Physical Exam* ... *49*
7.3.3 *Ultrasound and Imaging Exams* ... *51*
7.3.4 *Biospecimens* ... *51*
7.3.5 *Questionnaires* ... *52*

8 MANAGEMENT OF ADVERSE EXPERIENCES ... 53

8.1 EXPECTED ADVERSE EXPERIENCES ... 53
8.1.1 *Phase I* ... *53*
8.1.2 *Phase II* ... *53*
8.1.3 *Expected Serious Adverse Events* ... *53*

9 CRITERIA FOR MODIFICATION OR DISCONTINUATION OF STUDY INTERVENTIONS ... 54

9.1 PHASE I ... 54
9.1.1 *Intensive Lifestyle Modification* ... *54*
9.1.2 *Standard Lifestyle Intervention* ... *54*
9.2 PHASE II ... 55
9.3 PHASE III AND IV ... 55

10 STATISTICAL CONSIDERATIONS ... 56

10.1 GENERAL DESIGN ISSUES ... 56
10.2 RATIONALE FOR DESIGN ... 56
10.3 OUTCOMES ... 56
10.3.1 *Primary Outcome Measurements* ... *56*
10.3.2 *Secondary Outcome Measurements* ... *56*
10.4 SAMPLE SIZE AND ACCRUALS ... 57
10.4.1 *Sample Size and Power Calculations* ... *57*
10.4.2 *Accrual* ... *57*
10.5 DATA MONITORING ... 58
10.6 STUDY MONITORING ... 58
10.7 DATA AND SAFETY MONITORING BOARD ... 59
10.8 REPORTING ... 59
10.9 DATA ANALYSIS ... 59

11 DATA COLLECTION, SITE MONITORING AND ADVERSE EXPERIENCE REPORTING ... 62

11.1 RECORDS TO BE KEPT ... 62
11.1.1 *Maintenance/Retention of site records* ... *62*
11.2 ROLE OF DATA MANAGEMENT ... 62
11.3 QUALITY ASSURANCE ... 62
11.3.1 *Data Entry and Forms* ... *62*
11.3.2 *Features of Data Management System* ... *62*
11.3.3 *Data Security* ... *63*
11.3.4 *Data Quality Control* ... *63*
11.3.5 *Obligation of the Investigator* ... *64*
11.3.6 *Establishment of Pregnancy Registry* ... *64*
11.3.7 *Regulatory Requirements* ... *65*
11.3.8 *Protocol Amendments* ... *65*
11.4 ADVERSE EVENT REPORTING ... 65
11.4.1 *Serious Adverse Events* ... *65*

12 HUMAN SUBJECTS ... 69

12.1 INSTITUTIONAL REVIEW BOARD (IRB) REVIEW AND INFORMED CONSENT ... 69
12.2 SUBJECT CONFIDENTIALITY ... 69
12.3 STUDY MODIFICATION/DISCONTINUATION ... 69

13 PUBLICATION OF RESEARCH FINDINGS ... 70

13.1 OVERALL POLICY 70
13.2 MAIN STUDY 70
13.2.1 Major Publications (Tables 13-14) *70*
13.2.2 Minor Publications *72*
13.2.3 Ancillary Study *73*
13.2.4 Pilot Study *73*
13.2.5 Related Publications *73*
13.2.6 Outside Studies *74*
13.2.7 Presentations *74*

14 REFERENCES 75

15 HUMAN SUBJECTS/INFORMED CONSENT/FEMALE 81

16 HUMAN SUBJECTS/INFORMED CONSENT/MALE 108

17 APPENDIX A: RISK FACTORS FOR GENETIC DISORDERS 123

18 APPENDIX B: LIST OF COMMON MEDICATIONS EXCLUDED OR REQUIRING WASH-OUT PERIOD 127

19 APPENDIX C: SF 12 HEALTH SURVEY 129

20 APPENDIX E: PHQ-9 136

21 APPENDIX F: FEMALE SEXUAL DISTRESS SCALE (FSDS - REVISED 2005) 138

22 APPENDIX G: FERTIQOL 139

23 APPENDIX H: STOP BANG QUESTIONNAIRE 140

24 APPENDIX I: EPWORTH SLEEPINESS SCALE 141

25 APPENDIX J: INTERNATIONAL INDEX OF ERECTILE FUNCTION (IIEF) 142

26 APPENDIX K: DIET HISTORY QUESTIONNAIRE II (DHQ II) 145

27 APPENDIX L: AUTOMATED SELF-ADMINISTERED 24-HOUR RECALL (ASA24) 185

28 APPENDIX M: QADAM QUESTIONNAIRE 197

29 APPENDIX N: PSYCHOSEXUAL DAILY QUESTIONNAIRE 198

30 APPENDIX O: DSMB CHARTER 199

31 APPENDIX P: INVESTIGATOR SIGNATURE OF AGREEMENT 208

FIGURE 1. RELATIONSHIP BETWEEN TOTAL CALORIES AT THE TIME OF CONCEPTION AND BIRTH RATE IN THE NETHERLANDS DURING THE POST WAR FAMINE 1944-46 14
FIGURE 2: LIVE BIRTH PROBABILITY BY TREATMENT ARM IN OWL PCOS TRIAL 20
FIGURE 3: FIT-PLESE STUDY FLOWCHART 28
FIGURE 4: MODIFIED FERRIMAN-GALLWEY HIRSUTISM SCALE 49
FIGURE 5: SERIOUS ADVERSE EVENT FLOW CHART 67
FIGURE 5: SERIOUS ADVERSE EVENT FLOW CHART 67

Table 1: Effect of Duration of Exercise on IVF and pregnancy outcomes in a cohort of women undergoing IVF treatment 15
Table 2 Summary of RCTs from recent systematic review ([1] examining interventions on weight and fertility outcomes in overweight or obese women). 19

Table 3: Effect of preconception intervention on ovulation, pregnancy, and live birth 21
Table 4: Baseline values of select reproductive/metabolic parameters, and mean change at the end of Phase I (four months of treatment) from baseline. P-value is among the 3 groups for mean change..**Error! Bookmark not defined.**
Table 5: Adverse events with significant differences between groups by Phase of Study and all Serious Adverse Events....**Error! Bookmark not defined.**
Table 6: Live birth per treatment cycles of AMIGOS patients with BMI>=30 25
Table 7: Study Visits and Procedures ....**Error! Bookmark not defined.**
Table 8: Female Local Laboratory Screening Blood Tests 41
Table 9: Study Medication and Dosing ....**Error! Bookmark not defined.**
Table 10: Acne Grading Method 50

Acronyms

Advisory Board	AB	Institutional Review Board	IRB
American Congress of Obstetricians and Gynecologists	ACOG	Intrauterine Insemination	IUI
Anti-Mullerian Hormone	AMH	Investigational New Drug	IND
Assessment of Multiple Intrauterine Gestations from Ovarian Stimulation	AMIGOS	Large Gestational Age	LGA
Assisted Reproductive Technologies	ART	Logistic Regression	LR
Aromatase Inhibitors	AI	Luteinizing Hormone	LH
Clinical Report Form	CRF	National Institute of Child Health and Human Development	NICHD
Clomiphene Citrate	CC	Non-obstructive Azospermia	NOA
Centers for Disease Control	CDC	Ovarian Stimulation	OS
Data Coordination Center	DCC	Ovarian Hyperstimulation Syndrome	OHSS
Data and Safety Monitoring Board	DSMB	Principal Investigator	PI
Deoxyribonucleic Acid	DNA	Progesterone	P4
Estrogen	E	Pregnancy in Polycystic Ovary Syndrome Study	PPCOS
Estradiol	E2	Pregnancy of Unknown Location	PUL
Estrogen Receptor	ER	Protected Heath Information	PHI
Food and Drug Administration	FDA	Quality of Life	QOL
Follicle Stimulating Hormone	FSH	Reproductive Medicine Network	RMN
Female Sexual Distress Scale	FSDS	Serious Adverse Event	SAE
Female Sexual Function Index	FSFI	Sex Hormone Binding Globulin	SHBG
Good Birth Outcome	GBO	Single-Nucleotide Polymorphism	SNP
Health Insurance Portability and Accountability Act	HIPAA	Specialized Cooperative Center Programs in Reproductive Research	SCCPIR
Human Investigation Committee	HIC	Sonohysterogram	SHG
Human Chorionic Gonadotropin	hCG	Thyroid-Stimulating Hormone	TSH
Hysterosalpingography	HSG	Total and Free Testosterone	T, FT
Identification	ID	Ultrasound	U/S
International Index of Erectile Dysfunction	IIEF	World Health Organization	WHO

Intent-to-Treat	ITT		
Intramuscular	IM		

1 Study Synopsis

1.1 Objectives

To determine the safety and efficacy of pretreatment with lifestyle modification in obese women with unexplained infertility.

1.2 Hypothesis

An intensive lifestyle modification intervention (which includes caloric restriction, use of an over-the-counter weight loss medication, and increased physical activity) designed to promote a weight loss of approximately 7% of initial body weight is more likely to achieve a good perinatal outcome (i.e. a healthy term normal weight infant) than a recommendation to standard lifestyle modification with increased physical activity (based on publicly available activity recommendations) in obese women with unexplained infertility.

1.3 Patient Population

The population will consist of 380 obese women with unexplained infertility, age 18-40 years old. Subjects must have normal ovulatory function and normal ovarian reserve. Additionally, the couple will have no other major infertility factor: the subject will have at least one patent fallopian tube and a normal uterine cavity, and a partner total motile sperm count of at least 5 million in at least one ejaculate.

1.4 Study Design

This will be a two-arm, multicenter, prospective, randomized clinical trial of a lifestyle modification program with increased physical activity and weight loss (intensive) compared to recommendations to increase physical activity alone with weight maintenance (standard) in women with obesity and unexplained infertility. This 16 week period of lifestyle modification will be followed by an open label empiric infertility treatment regimen consisting of three cycles of ovarian stimulation with oral medication (clomiphene), triggering of ovulation with human chorionic gonadotropin (hCG) and intrauterine insemination (IUI).

1.5 Treatment

The intensive lifestyle modification intervention will consist of caloric restriction (consumption of approximately 1200-1500 kcal/d) centering around the use of meal replacements (Nutrisystem), use of an over-the-counter weight loss medication (Alli, which is brand name Orlistat, a gastric lipase inhibitor which limits gut fat absorption), and increased physical activity (goal of reaching 10,000 steps a day). The pretreatment intervention will last 16 weeks and is designed to promote a weight loss of approximately 7% of total body weight in the intensive group. Women in the standard lifestyle intervention (standard) will receive publicly available written materials that promote engagement in increased physical activity. Detailed instruction of physical activity will not be provided. Participants in both groups will receive activity tracking devices (Fitbit Wireless Activity Tracker) and wireless scales (Fitbit Aria Wireless Activity Scale) to promote adherence to the inventions and to allow monitoring for compliance by study personnel. The pretreatment intervention will last 16 weeks. Both groups will aim to build up to 10,000

steps/day with a recommendation to increase steps from baseline by 500 steps/per week. We will monitor subjects monthly during this preconception intervention. All subjects randomized will receive after 16 weeks of lifestyle modification, regardless of adherence or success in achieving treatment goals, a standardized empiric infertility treatment. This will consist of ovarian stimulation with an oral medication (clomiphene) followed by ultrasound follicular monitoring, human chorionic gonadotropin (hCG) trigger of ovulation, and a single partner intrauterine insemination (IUI) per treatment cycle for up to three treatment cycles. The goal for both treatment groups will be to maintain levels of physical activity and weight achieved during the pretreatment phase during the empiric infertility treatment phase. Subjects who conceive will be followed throughout pregnancy with the wireless activity monitor and wireless scale. Additionally there will be three brief onsite visits during pregnancy (per trimester at 16, 24, and 32 weeks) for onsite determination of weight, blood pressure, glycemic parameters, and collection of biospecimens. All pregnancy outcomes will be tracked. Subjects who deliver will be encouraged to donate placenta and cord blood to the study repository and then to enroll in our Pregnancy Registry for continued infant follow-up. We will also expand the number and variety of specimens we collect for the repository from both partners including urine and serum, semen, placenta and cord blood.

1.6 Primary efficacy parameter

Cumulative Good Birth Outcome during the study. Good Birth Outcome will be defined as a live birth of an infant born at ≥ 37 weeks, with a birth weight between 2500 and 4000g and without a major congenital anomaly.

1.7 Secondary efficacy parameters

We will assess secondary outcomes including live birth (birth after 20 weeks), time to pregnancy, pregnancy loss rate [including ectopics and Pregnancies of Unknown Location (PULs)], multiple pregnancy rate, pregnancy complication rate including development of gestational hypertension and diabetes, birth weight, neonatal complication rate, predictive factors for response including DNA polymorphisms, sleep quality, sexual function and psychosocial functioning (health-related quality of life and weight-related quality of life).

1.8 Statistical Analysis

The primary analysis will use an intent-to-treat approach examining differences in the good birth outcome rate in the two treatment arms. The study is powered with 80% power and an alpha of 0.05 to detect an absolute 15% difference in good birth outcome (a live birth of an infant born at ≥ 37 weeks, with a birth weight between 2500 and 4000g and without a major congenital anomaly) between the two treatment groups. We project the proportion of good birth outcomes to be 0.25 in the standard lifestyle group and 0.40 in the intensive lifestyle group. The sample size is inflated to reflect a 20% dropout rate, giving us 190 subjects in each group. A logistic regression model will be fit to compare the treatment arms with respect to the primary outcome of good birth outcome while adjusting for the randomization stratification factors of study site and baseline BMI. The analysis of other secondary (supplemental) outcomes measured over time will entail the application of statistical methods that have been developed for correlated data since repeated observations will be made over time on each individual. For secondary outcomes

such as weight loss, a linear mixed-effects model will be fit where the main independent variables will be treatment group, time, and their interaction as well as the randomization stratification factors as covariates. Logistic regression models will be used in secondary analyses to evaluate the predictive value of treatment arm, clinical site, prior exposure to ovulation induction drugs, age, and other explanatory variables on binary outcomes (e.g., singleton live birth, abortion). Cox proportional hazards models and a Kaplan-Meier method will be applied to compare time to pregnancy in the treatment groups.

1.9 Anticipated time to completion

A total of 2.6 years will be required to complete the study after start up; 15 month enrollment period (based on 2 subjects per site/month x 13 sites), 7 month treatment period (4 months lifestyle pretreatment and 3 months ovarian stimulation/IUI), with 9 month additional observation to determine pregnancy outcomes.

1.10 Regulatory Compliance

The DCC is working with The Eunice Kennedy Shriver National Institute of Child Health and Human Development (NICHD) to ensure that clinical study data and regulatory requirements are met regarding the Food and Drug Administration (FDA) code for federal regulations. This trial is registered on *http://www.clinicaltrials.gov* (NCT#02432209).

2 Study Objectives

2.1 Primary Aim

Our primary goal is to examine the efficacy of an intensive lifestyle modification intervention, which includes caloric restriction by using meal replacements, a weight loss medication (over the counter orlistat) and physical activity recommendations versus a standard lifestyle intervention consisting of increasing physical activity alone on improving good birth outcomes in obese women with unexplained infertility. Implicit in the primary aim is the goal of tracking safety of our interventions at all Phases of the study (through lifestyle modification to infertility treatment to pregnancy to infant).

2.2 Secondary Aim

We will assess secondary outcomes including live birth rate, time to pregnancy, pregnancy loss rate [including ectopics and Pregnancies of Unknown Location (PULs)], multiple pregnancy rate, pregnancy complication rate including development of gestational hypertension and diabetes, birth weight, mode of delivery, neonatal complication rate, predictive factors for response including DNA polymorphisms, stool and vaginal microbiome composition, psychosocial outcomes, and cost effectiveness.

2.3 Tertiary Aims

1. Is there a relationship between amount of weight loss or amount of physical activity during lifestyle modification and ovulatory response and pregnancy?
2. Does physical activity alone lead to healthier pregnancies (i.e. better gestational weight gain, blood pressure change, glycohemoglobin levels)?
3. Is there a biphasic effect of either weight loss or physical activity on conception and pregnancy?
4. Does lifestyle modification affect the menstrual cycle of regularly ovulating women?
5. Is this treatment more cost effective than immediately proceeding with empiric infertility treatment? (compare with AMIGOS data)
6. Does weight loss or physical activity increase sexual function and amount of intercourse?
7. Can sperm microarray identify couples more or less likely to conceive?
8. Does perceived stress and self-reported quality of life by husband and wife influence success? [Assess by SF-12 and PHQ-9]]
9. Is length of luteal phase predictive of pregnancy outcome?
10. Is there a relationship between length of infertility and conception?
11. Is there a difference in pregnancy rate in the first vs. second vs. third vs. fourth cycle?
12. Does weight loss or physical activity reduce the rate of metabolic syndrome?
13. Does the baseline vaginal or stool microbiome effect response to treatment and outcomes.
14. Does treatment effect the stool or vaginal microbiome?
15. Is the stool microbiome heritable to offspring?
16. Are there changes in stress as determined by salivary cortisol according to treatments?

3 Background

3.1 Rationale

3.2 Obesity and Reproduction

Obesity is associated with poor reproductive outcomes in women. This includes a longer time to pregnancy,[1] an association with ovulatory dysfunction,[2] lower conception rates with infertility treatment from ovulation induction to ovarian stimulation to IVF,[3-6] an increased chance of pregnancy loss with conception,[7,8]}[9] and higher rates of major pregnancy complications such as pre-eclampsia, pre-term labor, and gestational diabetes leading to increased risk of maternal, fetal, and infant morbidity and mortality.[10-12] The epidemiologic evidence linking female obesity to poor reproductive outcomes is massive and can be overwhelming. Consequently experts, major medical societies and public health programs have endorsed or mandated weight loss in obese women before initiating infertility therapy.[13,14] For example, in the United Kingdom infertility treatment is discouraged in women with a BMI > 35,[15] and in New Zealand, it is forbidden to provide public assistance to treat infertility in women with a BMI > 32.[16,17] While obesity is associated with poor reproductive outcomes, it is not known if weight loss by obese women improves reproductive outcomes. Common sense supports this belief, as well as extrapolated data from lifestyle modification studies to prevent diabetes or to secondarily prevent cardiovascular events. _Unfortunately the clinical trial evidence that lessening obesity improves reproductive outcomes is less than underwhelming, it is non-existent._ A recent Cochrane meta-analysis highlighted the complete absence of randomized trials indicating a benefit of weight loss on pregnancy rates or pregnancy outcomes.[18]

Figure 1. Relationship between total calories at the time of conception and birth rate in the Netherlands during the post war famine 1944-46

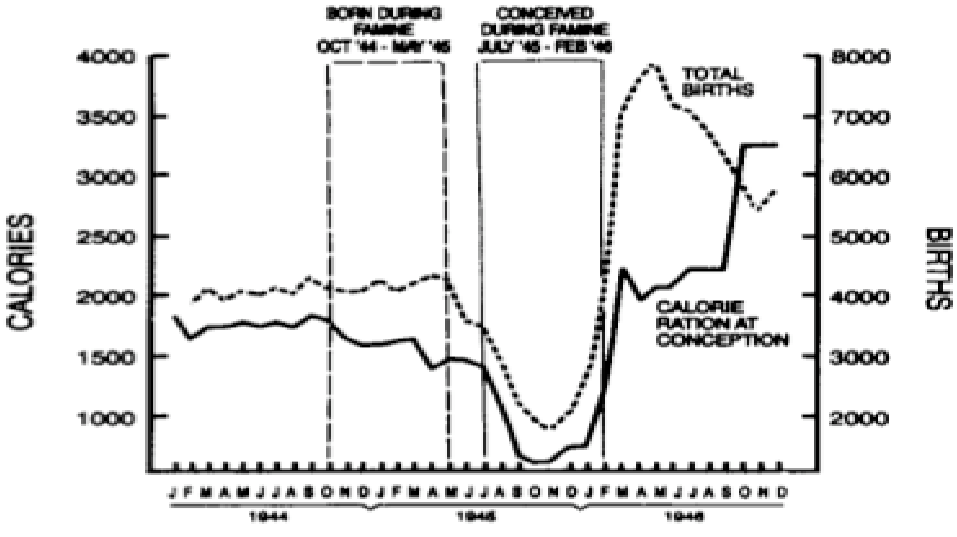

While no one can deny the health benefits of weight loss and lifestyle modification on overall health, the unique energy requirements for ovulation and pregnancy may create a scenario where weight

loss, or the stress of exercise, may produce counterintuitive and adverse reproductive outcomes. Weight loss, or at least meaningful weight loss, requires a significant investment of resources, effort, and time; therefore, it is imperative, especially where age remains the single most important predictor of pregnancy success in infertility treatment,[19] that we not waste time without clear evidence of benefit.

Why is weight and body fat critical to reproduction? A certain threshold of body fat is essential to reproductive maturation and menarche. Without it menarche is delayed. This concept known as the Frisch Hypothesis[20] has become widely accepted and supported by our own studies.[21] The rapid decline in the age of menarche over the last 100 years in most developed countries is attributed to improved food supplies and increased fat mass in prepubertal girls.[22] Further loss of fat mass due to disorders such as anorexia nervosa or among high performance athletes (such as gymnasts or runners) is associated with secondary amenorrhea. There does not appear to be an upper threshold of body fat that causes similar ovulatory dysfunction. The notion that obesity causes PCOS is debunked by many authors and certainly in many parts of the world such as East and South Asia where most women with PCOS are normal weight.[23] Further there is evidence from detailed studies of morbidly obese women undergoing bariatric surgery that their ovulatory function is normal, though they may have longer follicular phases or relatively inadequate luteal phases, both of which tend to normalize with massive weight loss following surgery.[24-26]

3.3 Adverse effects of lifestyle and weight loss on pregnancy

It is difficult to accept the hypothesis that weight loss and lifestyle modification may not have the desired effect on improving fertility in obese women. However there is evidence from the epidemiologic literature that forced caloric restriction is associated with decreased population fecundity. In developing nations, famines are often associated with marked declines in birth rates (ranging from 25-26%). One of the most closely studied famines in the developed world occurred in the Netherlands immediately after WWII from 1944-1946. The observation of this nutritionally challenged birth cohort formed the basis for the Barker Hypothesis regarding developmental origins. Interestingly the birth rate plummeted with the onset of the famine

Table 1: Effect of Duration of Exercise on IVF and pregnancy outcomes in a cohort of women undergoing IVF treatment

	No Exercise (n=859)	All Categories (n=1,305)	Category 1 1–3 h/wk for 1–9 y (n=221)	Category 2 4 h or more/wk for 1–9 y (n=230)
Cycle cancellation	1.0 (referent)	1.2 (0.8–1.8)	1.0 (0.5–1.9)	2.8 (1.5–5.3)*
Failed fertilization	1.0 (referent)	1.1 (0.7–1.6)	0.7 (0.4–1.6)	1.2 (0.6–2.6)
Implantation failure	1.0 (referent)	1.3 (1.0–1.6)†	1.0 (0.7–1.5)	2.0 (1.4–3.1)*
Pregnancy loss	1.0 (referent)	1.3 (1.0–1.8)	1.3 (0.8–2.2)	2.0 (1.2–3.4)*
Successful live birth	1.0 (referent)	0.8 (0.7–1.0)	0.9 (0.6–1.3)	0.6 (0.4–0.8)*

and rapidly climbed back following the end of the famine, implying that rather than fat supply, caloric restriction and restoration were central to the birth rate (**Figure 1**). The hazards of

attempting conception and maintaining conception during periods of acute caloric restriction are also supported by the experience with bariatric surgery leading to recommendations to avoid conception in the 6-12 months after surgery during the period of greatest weight loss and gastric restriction. There are also examples from the infertility literature that acute and severe caloric restriction during the period of infertility treatment is associated with reduced fecundity. For example, one trial severely calorie restricted (456-1200 kcal/d) obese infertile women during an IVF cycle prior to and during the IVF cycle up to oocyte retrieval and noted poor fertilization and implantation rates and was stopped prematurely.[27] We note here that publishing such adverse results of studies is rare in our field, and the absence of data regarding benefit and lack of harm may in part reflect a publication bias. There is also evidence that exercise, or at least relatively more exercise in a population of women undergoing IVF, may be associated with lower implantation rates, but once implanted higher delivery rates, implying a bi-phasic association with fecundity (**Table 1**).[28] There is also evidence from Norway that women who exercise more over a lifetime are more likely to be nulliparous.[29] Exercise, especially when associated with energy deficit, has been associated with inducing menstrual disturbances and anovulation in eumenorrheic women.[30-32] It is important to highlight these potential adverse effects of lifestyle modification to underscore the importance of a prospective randomized trial to better address the risk benefit ratio.

3.4 Current Status of Obesity Management

A program of diet, physical activity, and behavior therapy (i.e., lifestyle modification) is the cornerstone of treatment for most obese individuals.[33] In trials conducted in academic medical centers, persons treated by a 1200-1500 kcal/d diet, combined with regular exercise and a comprehensive program of group or individual lifestyle modification, lose approximately 7%-10% of initial weight in 20-26 weeks.[34-36]

<u>Effects of exercise alone in lifestyle modification on health</u> It is generally agreed that programs emphasizing exercise alone (as compared to or combined with weight loss) generally have no or little associated weight loss.[37] However exercise alone, with respect to the prevention and treatment of diabetes and cardiovascular disease has been found to have clear benefit with minimal risks. Programs of exercise alone (without dietary modification) have been found to significantly improve glucose and glycohemoglobin levels in patients with type 2 diabetes.[38] Further multiple large cohort studies have found that increasing amounts of habitual aerobic fitness and/or physical activity are associated with significantly lower cardiovascular and overall mortality to a much greater extent than could be explained by decreases in glucose lowering alone.[37-39] Exercise alone has also been associated with favorable changes in body composition,[40] i.e. decrease in centripetal obesity, which may favorably impact reproduction.[41] No significant effect on sex hormones was noted in an aerobic exercise intervention (150 mins/week) in a cohort of premenopausal women compared to control women.[42] In our FIT-PLESE study we will be proposing exercise alone as a comparison to weight loss through caloric restriction, anticipating minimal weight loss but an overall improvement in fitness and health which may favorably impact reproduction.

<u>Pharmacotherapy</u> Following the NHLBI's stepped care algorithm,[33,43] pharmacotherapy is an option for persons unable to lose 10% of initial weight with lifestyle modification alone and who

have a BMI > 30 kg/m^2 (or > 27 kg/m^2 in the presence of co-morbid conditions). Pharmacotherapy has a checkered history with many medications being removed post FDA approval due to unacceptable cardiovascular complications, including fenfluramine and sibutramine, and others never achieving FDA approval, such as rimonabant, due to concerns about mood changes and suicidality. Although a number of weight loss medications have recently been approved by the FDA, these medications lack a proven track record of safety and efficacy, and this had discouraged us from incorporating them into a clinical trial. However, there is a medication that has survived the test of time and found to be both safe and efficacious, which is orlistat. Orlistat is a gastric and pancreatic lipase inhibitor that induces weight loss by blocking the absorption of about one-third of the fat contained in a meal. [44-47] This medication combined with a modest program of lifestyle modification, is associated with mean loss of 8% - 10% of initial weight and improvements in metabolic risk factors.[44-53] As evidence of its safety, this medication is available in a reduced dose as an over the counter (OTC) medication (Alli). Further, as noted below in our preliminary data, we have successfully used this OTC medication without major adverse effects in a trial of lifestyle modification in obese women with PCOS seeking pregnancy. Overall the medication is well tolerated, and to patients it provides a "gut check" as excessive fat consumption will lead to a powerful wave of steatorrhea.

Increasing initial weight loss Although loss of as little as 5% of initial weight is associated with improvements in health, larger losses are generally associated with greater improvements in glycemic control, B/P, and lipids [43,54,55] and presumably reproductive function. The use of portion-controlled servings of conventional foods,[56,57] as well as liquid meal replacements,[58,59] is effective in increasing initial weight losses by approximately 3 kg, as compared with the prescription of a self-selected diet of conventional foods with the same calorie goal. Portion-controlled servings, by providing foods of pre-determined quantity and energy content, reduce the obese individual's tendency to underestimate his/her calorie intake,[60] which has been found to be as great as 50% when a self-selected diet of conventional foods is consumed.[61] The addition of pharmacotherapy to lifestyle modification also increases initial weight loss by approximately 4 to 6 percentage points (e.g., from 6% to 10%), compared with lifestyle modification alone.[46,62] This additive benefit is observed whether participants receive a modest program of lifestyle modification (e.g., a few visits with a dietitian)[62] or a comprehensive program (weekly group meetings).[63] We successfully used meal replacements in our OWL PCOS trial discussed below, and note that this contributed substantially to meeting our goal of a 7% weight loss in a 16 week pretreatment period in obese women with PCOS.

Barriers to Treating Obesity in Medical Practice Obesity is the most frequently encountered problem in primary care practice and the one least likely to be addressed. Primary care physicians, by their own report (or that of their patients) do not discuss weight management with 50% or more of their overweight and obese patients.[64,65] *Ob/Gyns, for instance, tend to not refer their patients for lifestyle modification interventions for weight loss or to use weight loss medications.[66]* Physicians' inactivity in this area appears to be attributable to multiple factors including providers' perceptions that: 1) obesity is a problem of willpower, not medicine (representing a bias against obesity); 2) broaching the subject of weight control is uncomfortable for both patient and provider; 3) most therapies are ineffective; and 4) treatment is not

adequately reimbursed.[67,68] Practitioners also believe they lack the training and time to provide adequate weight counseling. Other countries with socialized medical systems have developed multi-specialty interventions to treat obesity in women seeking conception. The Fertility Fitness program, established in an urban setting in Australia, involved weekly dietetic and behavioral intervention in a group environment, and used a multidisciplinary team approach, including an obstetrician/gynecologist, psychiatrist, dietician, fitness professional, and nurse.[69] This program reported restoration of ovulation in women with PCOS, improvement in pregnancy, and reduction in miscarriage rates,[70] but the dropout rate was 30% due to the intensive monitoring and multiple weekly visits that were part of the program. These results, however, were obtained without randomization or alternative treatment, and there was no standardized infertility treatment following the intervention. Further, we believe that such a program, given its expense and lack of a U.S. clinical practice model, is impractical and unlikely to result in a widespread adaption. We have instead sought to create a more streamlined approach to lifestyle modification that can be readily understood by patients and clinicians, and easily incorporated into clinical practice.

3.5 Good Birth Outcome

In this protocol we propose to narrow the primary outcome of a RMN trial from live birth, traditionally defined as any delivery beyond 20 weeks to one that reflects the desires of our patients and the demands of public health, i.e. a good perinatal outcome and specifically a good birth outcome. A sole focus on live birth as the metric of ART success in the U.S. has led to practices which encourage multiple embryo transfer to ensure high live birth rates, at the cost of an unacceptably high multiple pregnancy rate and among the multiple births a higher risk of adverse maternal (gestational hypertension, pre-eclampsia, gestational diabetes, cesarean delivery and hospital admission and neonatal (low birth weight, prematurity, NICU admission, birth defects) outcomes.[71,72] A recent study of the National ART Surveillance Group at the CDC defined a "good perinatal outcome" as "a singleton or twin term live birth with normal birth weight neonates".[73]

When "good perinatal outcome" was substituted for live birth, transfer of one embryo significantly improved the chance for this compared to multiple embryo transfers in women < 35 years old as well as women 35-37 years old. It is estimated that in the U.S. in 2011 a total of 36% of twin births and 77% of triplet and higher-order births resulted from conception assisted by fertility treatments.[75] Most of the twin births are thought to be due to ovarian stimulation and not IVF, and the rate of twin births in the U.S. has continued to increase over recent years, despite the dramatic reduction in triplet and other high order pregnancies (related to fewer embryos transferred during IVF).[75] We believe that the goals of any infertility treatment, either ovarian stimulation or IVF should be preferably a pregnancy with a good perinatal outcome.[75] We note a similar pattern with ovarian stimulation.[74] Gonadotropins for couples with unexplained infertility has a significantly higher live birth rate, but due to the high multiple rate, a lower proportion of good perinatal outcomes. In this study we have chosen a good perinatal outcome, revised here to exclude LGA babies (birth weight ≥ 4500g) as they pose a significantly greater risk for poor perinatal outcomes due to macrosomia such as operative delivery, perineal trauma, shoulder dystocia, fetal injury, NICU admissions etc., especially in obese mothers.

Because we are focusing on livebirth, we have altered the name from “good perinatal outcome” to “good birth outcome” in our protocol. Good birth outcome is also a term more comprehensible to our target patient population.

3.6 Supporting Data

<u>Systematic Review and Meta-analyses</u>

A recent systematic review and meta-analysis was performed examining the effects of pretreatment weight loss on subsequent fertility.[1] Of note, these authors reviewed 499 published records and could locate only 2 randomized controlled trials, one of which was described as a pilot trial.[76] (An excerpt is found in **Table 2**) Both of these were small, with less than 100 total subjects in each trial, included various causes of infertility, not just unexplained, and utilized IVF for infertility treatment as opposed to less invasive strategies. The treatment effect was large in the Sim et al trial[77] (absolute difference in live birth rate of 30% favoring weight loss), and less so in the Moran et al trial,[76] absolute difference in live birth rate of 5%, favoring weight loss. <u>We conclude that there have been a paucity of well designed RCTs in this field and that available data from cohort studies and the two RCTs support a benefit to weight loss prior to infertility treatment.</u>

Table 2 Summary of RCTs from recent systematic review ([1] examining interventions on weight and fertility outcomes in overweight or obese women).

Authors, year, reference	Study design	Type of weight loss intervention and description	Duration of weight loss intervention (follow-up)	Mean age in years and/or (range)	Sample size (intervention/control or drop-out)	Cause of infertility	Main findings for body mass index and fertility			Quality of study
							Weight loss	Pregnancy rate and live birth outcomes	Other	
Clark *et al.*, 1995 (23)	Cohort study (prospective)	Diet, exercise and behavioural support	6 months (12 months)	(21–40)	18 (13/5)	Anovulation with previous clomiphene resistance	6.3 ± 4.2 kg (intervention); 1.4 ± 1.8 kg (comparison); $P < 0.001$. No alteration in waist-to-hip ratio	Pregnancy rate: 85% (11/13), 55% (6/11) were naturally conceived (intervention); no pregnancies (comparison) Live births: 77% (10/13)	Ovulation: 92% (12/13) women were ovulating spontaneously (intervention); none (comparison)	Weak
Clark *et al.*, 1998 (24)	Cohort study (prospective)	Diet, exercise behavioural support	6 months (12 months)	(21–40)	87 (67/20)	Various, including anovulation and PCOS	10.2 ± 4.3 kg (intervention); 1.2 ± 3.6 kg (control); $P < 0.001$	Pregnancy rate: 78% (52/67), 35% (18/52) were naturally conceived (intervention); no pregnancies (comparison) Live births: 67% (45/67)	Ovulation: 90% (62/69) of the previously anovulatory women were ovulating spontaneously (intervention); none (comparison)	Weak
Galletly *et al.*, 1996 (25)	Cohort study (prospective)	Diet, exercise and behavioural support	6 months (21–36 months)	(22–40)	58 (37/21)	Various	6.2 ± 4.5 kg; $P < 0.001$. No comparative group data was reported for any parameter	Pregnancy rate: 78% (29/37), 24% (7/29) were naturally conceived (intervention) Live births: not reported		Weak
Sim *et al.*, 2014 (21)	RCT	Diet (including VLED), exercise and behavioural support	3 months (12 months)	(18–37)	49 (27/22)	Various	6.6 ± 4.6 kg (intervention); 1.8 ± 3.6 kg (control); $P = 0.001$ WC: 8.7 ± 5.6 cm (intervention); 1.0 ± 6.3 cm (control); $P = 0.001$	Pregnancy rate: 48% (13/27), 23% (3/13) were naturally conceived (intervention); 14% (3/22), of which none were naturally conceived (control); $P = 0.014$ for pregnancy rate Live births: 44% (12/27) (intervention), 14% (3/22) (control); $P = 0.02$	ART cycles taken to achieve pregnancy: 2.46 (intervention), 4.33 (control); $P = 0.01$	Moderate
Moran *et al.*, 2011 (22)	RCT	Diet and exercise	5–9 weeks prior to oocyte pick-up (not reported)	(18–40)	38 (18/20)	Various	3.8 ± 3.0 kg (intervention); 0.5 ± 1.2 kg (control); $P < 0.001$ WC: 5.3 ± 4.6 cm (intervention); 3.5 ± 3.5 cm (control); $P = 0.22$	Pregnancy rate: 67% (12/18) (intervention); 40% (8/20) (control); $P = 0.12$; natural conceptions not reported Live births: 39% (7/18) (intervention), 25% (5/20) (control); $P = 0.48$		Moderate

Preliminary Data from a Randomized Trial of Preconception Inverventions in women with Polycystic Ovary Syndrome (PCOS)

Figure 2: Live Birth probability by Treatment Arm in OWL PCOS trial

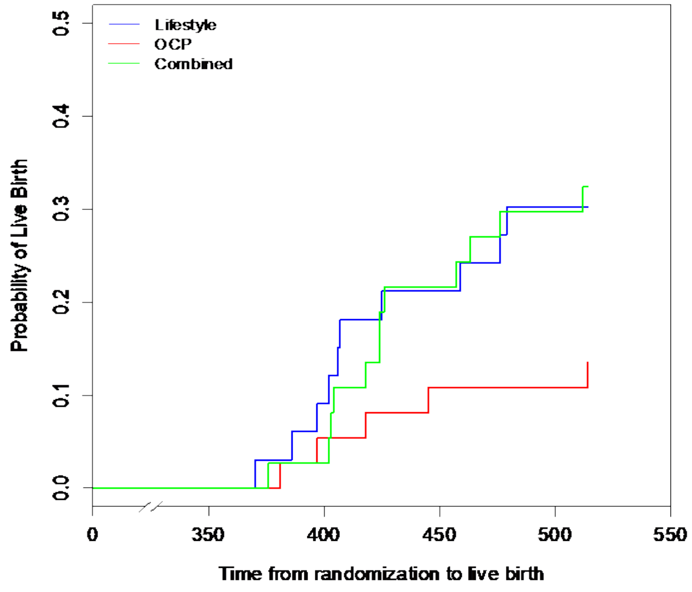

Overview We present now preliminary data from a randomized preconception lifestyle modification program that we are completing in obese women with PCOS. Though the target population in our FIT-PLESE trial is obese women with unexplained infertility, we believe the lessons from our preliminary trial are very relevant to this protocol. These lessons include that we can achieve substantial and projected weight loss in a finite period with meal replacements and orlistat, that our drop-out rates at all time points in in the trial (including lifestyle modification and infertility treatment phases) are low, that adverse event rates are minimal and serious adverse event rates absent, and that there may be a substantial treatment benefit in terms of pregnancy and live birth to lifestyle modification.

OWL PCOS study The OWL PCOS Study is a randomized, open-label clinical trial in overweight/obese PCOS women sponsored by an RO1 grant with Dr. Legro as PI and Drs. Dodson, Penny Kris-Etherton (Penn State) and Drs. Coutifaris and Sarwer (U Penn) as Co- Investigators (age 18-40 years old, BMI 27-42 km/m^2, and no other major infertility factors), of three preconception interventions (Phase I) followed by 4 cycles of ovulation induction with CC to all groups (Phase II), followed by pregnancy for those who conceive up to live birth (Phase III). The three treatment arms were: Lifestyle: weight loss through a regimen of weight loss drug/meal replacements/exercise enforced by behavioral modification with a target of 5-10% weight loss, OCP: Hormonal suppression through continuous oral contraceptive pills (OCP), and Combined: the simultaneous combination of both the Lifestyle Arm and the OCP arm, i.e. weight loss with

hormonal suppression. The primary hypothesis is that Combined > OCP > Lifestyle at achieving live birth. It was powered for 246 subjects, and we randomized 149 subjects. After randomization, this was an open label study so that we were able to track the outcomes of our participants. Based on these findings (**Table 3**) and the recommendations of our DSMB, we stopped enrollment in December 2012, and followed the final subjects through to live birth.

<u>Main Outcomes:</u> The primary outcome for OWL PCOS was live birth. As seen in the Kaplan Meier Curve in Figure 2 those women randomized to OCP (hormonal suppression, red curve) had the lowest probability of live birth, though it was not statistically significant (Table 3). Live birth rates were highest in Lifestyle and Combined arms (weight loss groups with or without hormonal suppression), which is less in Arm B-OCP compared to the other arms (**Figure 2**) These curves parallel the pregnancy rate curves with no differences in pregnancy loss between groups. (**Table 3**) Both Lifestyle and Combined treatments are superior to OCP, and also superior to extracted data from the PPCOS I and II studies (Live Birth Rate after 4 cycles CC/Placebo in PPCOS I =18.6% (29/209) and after 4 cycles CC in PPCOS II 16.2% (61/376). Compared to these results, both Lifestyle and Combined from our trial offered a significantly better ovulation rate[Lifestyle vs Clomiphene: relative risk=1.4; 95% confidence interval=(1.1, 1.7); p=0.005 and Combined vs Clomiphene: relative risk=1.5; 95% confidence interval=(1.2, 1.8); p<0.0001] and a significantly better live birth rate[Lifestyle vs Clomiphene: relative risk=2.5; 95% confidence interval=(1.3, 4.6); p=0.004 and Combined vs Clomiphene: relative risk=2.3; 95% confidence interval=(1.2, 4.3); p=0.01]. These data support the benefit of preconception weight loss in women with PCOS.

Table 3: Effect of preconception intervention on ovulation, pregnancy, and live birth

Outcome	OCP	Lifestyle	Combined	Lifestyle vs. OCP*			Combined vs. OCP*			Lifestyle vs. Combined*		
	no./total no. (%)	no./total no. (%)	no./total no. (%)	Absolute difference % (95% CI)	Risk ratio (95% CI)	p value[$]	Absolute difference % (95% CI)	Risk ratio (95% CI)	p value[$]	Absolute difference % (95% CI)	Risk ratio (95% CI)	p value[$]
**Ovulation**												
Ovulation (total number of ovulations/total treatment cycles)	71/154 (46.1)	82/136 (60.3)	94/140 (67.1)	14.1 (2.8, 25.3)	1.3 (1.0, 1.7)	0.06	20.7 (9.9, 31.6)	1.5 (1.1, 1.9)	0.002	-7.1 (-18.2, 4.1)	0.9 (0.7, 1.1)	0.28
**Pregnancy**												
Conception (serum hCG level>10 mIU/mL)	8/49 (16.3)	16/50 (32.0)	14/50 (28.0)	15.7 (-0.9, 32.2)	1.9 (0.9, 4.1)	0.09	11.7 (-4.2, 27.5)	1.7 (0.8, 3.7)	0.16	4.3 (-13.5, 22.2)	1.1 (0.6, 2.0)	0.72
Clinical pregnancy (fetal heart motion visualized on ultrasound)	7/49 (14.3)	13/50 (26.0)	13/50 (26.0)	11.7 (-3.9, 27.3)	1.8 (0.8, 4.2)	0.16	11.6 (-3.7, 27.0)	1.8 (0.8, 4.2)	0.15	0.3 (-16.8, 17.3)	1.0 (0.5, 1.9)	0.99
**Birth Outcomes**												
Live birth	5/49 (10.2)	13/50 (26.0)	12/50 (24.0)	15.9 (1.2, 30.6)	2.5 (1.0, 6.6)	0.06	13.8 (-0.5, 28.0)	2.3 (0.9, 6.1)	0.08	2.2 (-14.7, 19.0)	1.1 (0.5, 2.1)	0.82

We have also experienced excellent retention in this study. Our combined dropout rate is 19% which is lower than that for clomiphene in PPCOS I (23% and markedly higher for metformin alone) and in PPCOS II (20% overall), which is remarkable considering that the OWL PCOS study is much more intensive, of longer duration, and entails a lifestyle intervention which traditionally

has higher dropout rates than a purely pharmaceutical intervention such as the PPCOS trials. (**Figure 2**).

<u>Other Secondary Outcomes:</u> We have collected various data to ascertain the effectiveness of our preconception interventions (**Table 4**). Indeed, we have achieved excellent weight loss in the lifestyle modification arms (A and C) and weight maintenance in Arm B (erasing the concern that study participation *per se* would lead to weight loss). We initially started the study with sibutramine, an amphetamine-like appetite suppressant, as our weight loss medication, but removed this from our study in January 2010 just prior to the FDA ruling to remove it from the market because of concerns about increased cardiovascular event rates. We substituted over the counter (OTC) orlistat due to its lack of major safety concerns, including no known adverse pregnancy effects. We noted no difference in weight loss between the groups that received orlistat versus those who received sibutramine (data not shown). We examined a number of other parameters to show that the interventions have varied effects on the reproductive and metabolic phenotype of PCOS. These results show that greater suppression of AMH/ovarian volume with OCP suggests a mechanism for lower ovulation/pregnancy rates seen with Arm B (OCP). Our data show that weight loss erases metabolic exacerbations caused by OCP alone. High compliance with medical treatments was supported by pill counts: average compliance for OCP (Arm B) 97% and for orlistat (Arm A) 83%.

Table 4: Baseline values of select reproductive/metabolic parameters, and mean change at the end of Phase I (four months of treatment) from baseline. P-value is among the 3 groups for mean change

	**Lifestyle**		**Continuous OCP**		**Combined Weight Loss/OCP**		
**Parameter**	**Base-line Mean (SD)**	**Mean Change (95% CI)**	**Base-line Mean (SD)**	**Mean Change (95% CI)**	**Base-line Mean (SD)**	**Mean Change (95% CI)**	**P-value**
**Weight (kg)**	**96 (16)**	**-6 (-7,-5)**	**95 (14)**	**-1 (-2,-0)**	**95 (15)**	**-6 (-7,-5)**	**<.0001**
**Waist Circumference (cm)**	**107 (14)**	**-6 (-9,-4)**	**106 (11)**	**-2 (-5,1)**	**107 (12)**	**-6 (-9,-3)**	**0.04**
**Mean Arterial Pressure (mmHg)**	**86 (9)**	**-2 (-5,2)**	**88 (8)**	**2 (-2,6)**	**89 (10)**	**-1 (-5,2)**	**0.29**
**F-G Score**	**19 (9)**	**-0 (-1,1)**	**17 (8)**	**-1 (-2,0)**	**18 (9)**	**-1 (-2,0)**	**0.62**
**Sebum (mcg/cm^3)**	**98 (50)**	**7 (-15,29)**	**101 (59)**	**-21 (-42,1)**	**107 (53)**	**-28 (-50,-7)**	**0.06**
**AMH (pmol/L)**	**9 (6)**	**-1 (-2,1)**	**9 (5)**	**-3 (-4,-2)**	**9 (5)**	**-3 (-4,-2)**	**0.03**
**Antral Follicle Count (AFC)**	**54 (34)**	**-5 (-14,5)**	**66 (39)**	**-25 (-34,-15)**	**58 (34)**	**-19 (-29,-9)**	**0.01**
**Total Ovarian Volume (cm^3)***	**21 (14, 27)**	**1.0 0.9, 1.1)**	**22 (18, 30)**	**0.7 (0.6, 0.8)**	**19 (14, 25)**	**0.9 (0.8,1.0)**	**0.002**
**Fasting Glucose (mg/dL)**	**87 (9)**	**-3 (-7,2)**	**87 (10)**	**1 (-3,6)**	**90 (15)**	**-7 (-12,-3)**	**0.03**
**Fasting Insulin (mIU/mL)***	**25 (18, 32)**	**0.9 0.8, 1.1)**	**24 (17, 32)**	**1.0 (0.9, 1.2)**	**24 (15, 33)**	**1.0 (0.8,1.1)**	**0.63**
**TTG (mg/dL)***	**122 (99, 171)**	**1.0 (0.9, 1.1)**	**117 (94, 155)**	**1.2 (1.1,1.3)**	**120 (87, 165)**	**1.0 (0.9,1.2)**	**0.02**

<u>Adverse Events (AEs)</u> We have had 4 serious AEs to date (all brief hospitalizations): 3 were likely unrelated to study medications/interventions and 1 was an ectopic pregnancy (**Table 5**). We had no congenital anomalies and one IUFD and one neonatal death from a set of twins who delivered at 20 weeks. Potential side effects related to orlistat use are steatorrhea and diarrhea as well as abdominal pain (Table 5). These side effects are common with this treatment. We found the treatments to be equally safe. The fact that both Lifestyle and Combined have similar ovulation and pregnancy rates suggest that weight loss appears to be the predominant effect modifier, and that OCP exposure *per se* is not harmful. We also eliminated the elective use of progestin to induce withdrawal bleeds if anovulatory after CC early in the study.

Table 5: Adverse events with significant differences between groups by Phase of Study and all Serious Adverse Events.

	OCP	Lifestyle	Combined
	**N subjects (%)**	**N subjects (%)**	**N subjects (%)**
**Phase 1: Lifestyle intervention**	**N=49(%)**	**N=50(%)**	**N=50(%)**
Steatorrhea/Diarrhea†‡	0 (0.0)	6 (12.0)	12 (24.0)
Breast Pain†	10 (20.4)	1 (2.0)	6 (12.0)
Abdominal Pain‡	1 (2.0)	5 (10.0)	10 (20.0)
Dysmenorrhea†	8 (16.3)	1 (2.0)	3 (6.0)
Abnormal uterine bleeding¶	4 (8.2)	0 (0.0)	6 (12.0)
**Phase 2: Ovulation induction**	**N=44(%)**	**N=44(%)**	**N=43(%)**
Headache¶	6 (13.6)	4 (9.1)	12 (27.9)
**Serious Adverse Events: All Phases**	3 (6.1)	1 (8.3)	0 (0.0)
Phase 1: Episode of menorrhagia leading to ER visit	1 (2.0)	N/A	N/A
Phase 3: Ectopic pregnancy requiring surgery	1 (2.0)	N/A	N/A
Phase 3: Preterm Delivery of twins at 20 weeks: 1 live birth and 1 IUFD secondary to infection	1 (2.0)	N/A	N/A
Phase 3: Postpartum hospitalization for perforated appendix	N/A	N/A	1 (2.0)

* Indicates Serious Adverse Event (SAE). In addition to the 4 SAE cases reported above, there was one addition al

SAE in a non-randomized subject. The subject developed a pelvic infection following the screening hysterosalpingogram andwas diagnosed with Pelvic Inflammatory Disease which requi red hospitalization.

†P<0.05 for the comparison between Lifestyle and OCP.

‡ P<0.05 for the comparison between Combined and OCP.

¶ P<0.05 for the comparison between Lifestyle and Combined.

Summary of Results We have learned that our hypothesis about the treatment effects of OCP pretreatment versus lifestyle modification is reversed and analogous to our hypothesis in PPCOS I where we hypothesized that metformin was superior to CC at achieving live birth, and the combination superior to both. [78] In PPCOS I we also found the opposite, i.e. metformin markedly inferior to CC, and no clear benefit to the combination therapy. Similarly with OWL PCOS, we did not observe the trend we theorized, nor did we see that the combination is superior. Rather, lifestyle, alone or in combination with OCP, appeared superior to OCP alone. We do have solid data on which to power future studies about lifestyle modifications and effects on human fecundity. We also can accurately project recruitment rates, as we noted steady, though slower than anticipated recruiting. Although the study overlapped with PPCOS II at both of our centers, Penn State and University of Pennsylvania, there was the same rate of recruiting into OWL PCOS during the year after PPCOS II closed as when both studies were recruiting simultaneously. This indicates to us that this trial is possible in the context of other unexplained infertility trials in the RMN, and that until lifestyle becomes mainstream and open to all comers (not just below a certain BMI as the number one reason for screen failure in OWL PCOS was a BMI ≥ 42), this may not be the first choice therapy of patients.

Further and most importantly, we have demonstrated the feasibility and effectiveness of our preconception interventions on our main parameters and successfully validated our methods for collecting the primary/secondary outcomes of the study. Our pregnancy rates in this trial of overweight/obese women treated with weight loss and 4 cycles of CC is superior to that after 4 cycles of medication alone in either PPCOS I or PPCOS II. Our preliminary results of marked suppression of ovarian volume and AMH levels in the OCP arm and slightly lower ovulation rates suggests a possible ovarian mechanism of action for lower ovulation and pregnancy rates, and therefore we will not utilize this treatment as a control situation in the FIT-PLESE protocol.

Empiric Treatment of Infertility after Lifestyle Modification

We plan to proceed with a standardized empiric therapy for infertility after lifestyle modification that both treatment groups in this trial will receive. We plan to use our experience and data from the AMIGOS trial to power the sample size for this trial and aid in the design of the infertility treatments.

AMIGOS Preliminary Results The AMIGOS trial (Assessment of Multiple Intrauterine Gestations from Ovarian Stimulation) is a randomized trial of three empiric treatments for unexplained infertility: Clomiphene/IUI, letrozole/IUI, or human menopausal gonadotropins/IUI for up to four treatment cycles. This trial randomized 900 couples with unexplained infertility to these treatments and was completed in the last funding cycle of the RMN. We propose to adapt many of the inclusion/exclusion criteria of that study as well as some of the methodology for the empiric treatment phase. Based on the results of this trial, we have elected to use clomiphene/IUI as our empiric treatment for unexplained infertility after completion of the lifestyle modification phase. Although gonadotropin/IUI had the highest live birth rate, it also had a high rate of multiple pregnancy, including high order multiple pregnancy (triplets). Further, clomiphene/IUI had a higher cumulative live birth rate (23.3%) than letrozole/IUI (18.7%). We further extrapolated the data from the obese women (BMI ≥ 30) with unexplained infertility over

the first three treatment cycles in the AMIGOS trial and found that clomiphene IUI trended better both in terms of live birth and healthy live birth than letrozole (**Table 6**). We opted for three treatment cycles here to reflect current practice (i.e. to conduct 3 cycles of empiric therapy before moving on) as well as the declining pregnancy and live birth rates we experienced in the fourth treatment cycle (data not shown).

3.7 Summary of AMIGOS data

The AMIGOS data provides reassurance that clomiphene/IUI is an effective empiric infertility treatment and is non-inferior to letrozole (and trending better in terms of live birth and a healthy live birth).

Table 6: Live birth per treatment cycles of AMIGOS patients with BMI>=30

Variable	Clomiphene group (N=70)	Letrozole group (N=90)	Gonadotropin group (N=81)	All (N=241)
		no./total no. (%)		
**Live birth per treatment cycle**				
**Treatment cycle 1**				
Total live birth	4/70(5.7)	9/90(10.0)	16/81(19.8)	29/241(12.0)
Live birth with EGA>=37 wks and 2500<=birth weight<=4000 g	3/70(4.3)	6/90(6.7)	10/81(12.4)	19/241(7.9)
**Treatment cycle 2**				
Total live birth	7/66(10.6)	5/81(6.2)	4/65(6.2)	16/212(7.6)
Live birth with EGA>=37 wks and 2500<=birth weight<=4000 g	6/66(9.1)	3/81(3.7)	3/65(4.6)	12/212(5.7)
**Treatment cycle 3**				
Total live birth	3/59(5.1)	1/76(1.3)	4/61(6.6)	8/196(4.1)
Live birth with EGA>=37 wks and 2500<=birth weight<=4000 g	3/59(5.1)	0/76(0)	3/61(4.9)	6/196(3.1)
**Cumulative Live Birth Rate after 3 cycles**				
Total live birth	14/70(20.0)	15/90(16.6)	24/81(29.6)	53/241(22.0)
Live birth with EGA>=37 wks and 2500<=birth weight<=4000 g	12/70(17.1)	9/90(10.0)	16/81(19.8)	37/241(15.4)

Implications for the FIT-PLESE Protocol

Changes in the proposed FIT-PLESE protocol from the OWL PCOS I Protocol – We propose changes to lower participant burden in terms of time and visits, ease the conduct of the trial for the RMN and RMN sites, and lower the overall cost of the trial. (NOTE: We have conducted the RO1 OWL PCOS trial with direct costs < $500,000 per year, i.e. within the limits of an RO1 grant, most of which goes toward salary support that would be covered by the infrastructure support of the RMN grant).

First we propose to modify the primary outcome from live birth, to a good birth outcome (GBO) which we will define as: Singleton or twin infant (s) born at ≥ 37 weeks between 2500 and 4000g without a major congenital anomaly. We are modifying this to meet both our patient's and public health expectation to produce healthy infants. No one considers a 24 week, 400g premature infant who dies 1 day after delivery an optimal outcome. This is an emerging concept in infertility treatment recently used to examine the outcomes from single embryo transfers in the SART database.[74]

Second we propose to eliminate intensive testing, that sought out mechanisms of action of the preconception interventions. These would not be necessary for a larger Phase III type clinical trial. We propose eliminating serial OGTTs for glucose tolerance changes as well as derived measures of insulin action, serial DXA scans to determine changes in body composition, and serial VO2 submaximal treadmill testing to detect changes in exercise tolerance. All of these require specialized oversight and research facilities, which might not be present at all of the RMN sites, and involve substantial time commitment on the part of research subjects and staff. Additionally some of these tests including OGTT and exercise testing became especially unwieldy in the third trimester, and finally, they are disproportionately expensive. We will continue to track related parameters including homeostatic measures of insulin action and add a glycohemoglobin (HgbA1C) as an integrated measure of glucose homeostasis at several time points in the study.

Third we propose to add both the option to participate in the RMN Repository and Pregnancy Registry to the protocol, and thus we will collect serum, urine, semen, and saliva on the participants who consent (i.e. females, males, and parents for their infants). Additionally on females we will collect vaginal and stool samples for microbiome analysis, and on infants a meconium analysis. We will also encourage collection of placental samples and cord blood.

Fourth we propose to eliminate the upper limit weight cutoff (BMI <42) imposed on us by the study section for the RO1 OWL PCOS study and instead utilize the upper limit utilized in the PPCOS II and AMIGOS trials (which was none). This lack of an upper weight limit for these studies was approved by the NIH program officers, the SC, the Advisory Board, the DSMB, and the site IRBs. This will ease enrollment, as excessive BMI was the number one reason for screen failure in the study (N=182). It also allows access to all who suffer from obesity, not a select few, and thus will increase the external study validity. It is inherently unfair to let an obese woman with unexplained infertility immediately enroll in an empiric infertility study (i.e. AMIGOS), yet deny her the opportunity to first participate in a preconception intervention to improve her reproductive fitness (i.e. the current FIT PLESE Female protocol). We have designed the lifestyle

modification intervention such that we are modifying lifestyle through a combined approach of caloric restriction, pharmacotherapy, and increased physical activity.

4 Study Design

Figure 3: FIT-PLESE Study Flowchart

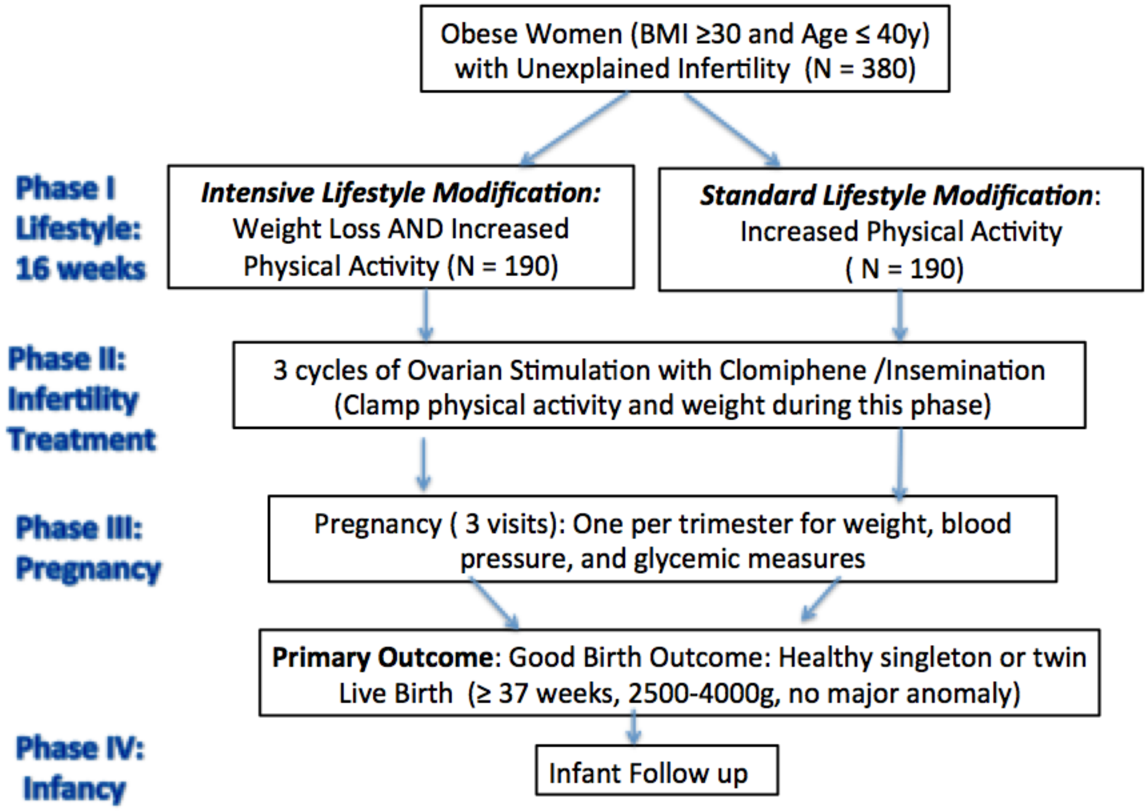

4.1 Overview

The flowchart (**Figure 3**) summarizes this study which will consist of infertility treatment commencing with randomization at equal allocation (1:1) to two interventions (Phase I), an intensive lifestyle modification which includes caloric restriction primarily with meal replacements (Nutrisystem), use of a weight loss medication (Alli), and physical activity recommendations, and a less intensive lifestyle modification intervention focused on increasing physical activity with weight maintenance. This will serve as the comparison group. After completion of the 16 week lifestyle intervention, subjects will receive 3 cycles of ovarian stimulation with an oral medication (clomiphene citrate), triggered ovulation with hCG, and a single intrauterine insemination per cycle (Phase II). During the infertility treatment phase, subjects will be encouraged to maintain their weight and activity levels achieved during the initial intervention. Subjects who conceive will be followed to the point of documented viable intrauterine pregnancy, and then will be seen once per trimester for a brief visit with serum and urine collection. All pregnancies will be followed until completion (Phase III). Subjects who deliver viable infants will be offered participation in our Pregnancy Registry (Phase IV). The details of the Pregnancy Registry protocol can be found in that protocol document.

4.1.1 Treatment Design

This will be a two-arm, multi-center, prospective randomized trial of two types of lifestyle modification in obese women with unexplained infertility who will be randomized to either an intensive lifestyle modification with weight loss and increased physical activity or a standard lifestyle modification consisting of increased physical activity for 16 weeks prior to initiating infertility treatment with an oral drug, clomiphene, to stimulate follicular development followed by intrauterine insemination (IUI). The lifestyle modifications will be open label with adequate allocation concealment and the randomization ratio will be equal between modifications (1:1). The appropriate study candidates will be recruited from the main sites of the Reproductive Medicine Network and their ancillary partners. Recruited subjects will meet the inclusion and exclusion criteria detailed below. Monitoring of this trial at all sites will be conducted by the RMN Data Coordination Center, with progress reports provided to the RMN Data and Safety Monitoring Board (DSMB) no less than every three months in order to review trial progress and subject safety.

4.1.2 Study Population

Three hundred eighty (380) infertile obese ovulatory women actively seeking pregnancy (190 per treatment arm), age 18-40 years, will be enrolled in the participating sites. The overall goal of the inclusion/exclusion criteria is to identify a population of healthy infertile women who are regularly ovulating. These will include a normal uterine cavity with at least one patent tube and a partner with a motile sperm count of at least 5 million in the ejaculate. If existing medical records are used to verify inclusion or exclusion criteria, the site should keep a copy of these in the source documents. A general list of exclusionary medications requiring a washout period is found in the appendix. The list is not exhaustive and questionable medications can be looked up to see if they belong to one of the families of exclusionary medications, or the Project Leader (Dr. Richard Legro) and/or DCC can be queried.

5 Selection and Enrollment of Subjects

5.1 Inclusion Criteria

1. Women ≥ 18 to ≤ 40 years of age, with one or more years infertility history, desirous of conceiving, regularly ovulating (defined as 9 or more menses per year), at initiation of participation.
2. BMI ≥ 30 kg/m2 obtained at screening visit.
3. Normal uterine cavity and at least one open fallopian tube confirmed by hysterosalpingography (HSG), sonohysterography (SHG), or laparoscopy/hysteroscopy in the last three years preceding enrollment into the study. An uncomplicated intrauterine non-IVF pregnancy and uncomplicated delivery and postpartum course resulting in live birth within the last three years will also serve as sufficient evidence of a patent tube and normal uterine cavity as long as the subject did not have, during the pregnancy or subsequently, risk factors for Asherman's syndrome or tubal disease or other disorder leading to an increased suspicion for intrauterine abnormality or tubal occlusion.
4. Evidence of ovarian function/reserve as assessed by day 3 (+/-2 days) FSH ≤ 10 IU/L with Estradiol ≤ 70 pg/mL OR serum AMH ≥ 1 ng/mL within one year prior to study initiation.
5. Normal or corrected thyroid function within one year of study initiation.
6. Normal or corrected prolactin level within one year of study initiation.
7. In general good health, not taking any medications which could interfere with the study (e.g., FSH, insulin sensitizers - see Appendix for full list).
8. Ability to have inseminations following hCG administration
9. Male partner with total motile sperm in the ejaculate of at least 5 million sperm, within one year of study initiation
10. Able to have intercourse and collect semen for insemination

5.2 Exclusion Criteria

1. Currently pregnant or successful pregnancies within 12 months of initiating participation. Clinical intrauterine miscarriages prior to initiating participation, within ASRM guidelines: subjects 35 and over must wait six months, while subjects under 35 must wait 12 months. No exclusion for biochemical pregnancies.
2. Undiagnosed abnormal uterine bleeding.
3. Suspicious ovarian mass.
4. Subjects on oral contraceptives, depo-progestins, or hormonal implants (including Implanon). A two month washout period will be required prior to screening for patients on these agents. Longer washouts may be necessary for certain depot contraceptive forms or implants, especially when the implants are still in place. A one-month washout will be required for patients who have taken oral cyclic progestins.
5. Known 21-hydroxylase deficiency or other enzyme defect causing congenital adrenal hyperplasia.
6. Type I or Type II diabetes mellitus, or if receiving antidiabetic medications.

7. Known significant anemia (Hemoglobin <10 g/dL).
8. History of deep venous thrombosis, pulmonary embolus, or cerebrovascular event.
9. Known heart disease (New York Heart Association Class II or higher).
10. Known liver disease (defined as AST or ALT >2 times normal, or total bilirubin >2.5 mg/dL).
11. Known renal disease (defined as BUN >30 mg/dL or serum creatinine > 1.4 mg/dL).
12. History of, or suspected cervical carcinoma, endometrial carcinoma or breast carcinoma.
13. History of alcohol abuse (defined as >15 drinks/week) or binge drinking of ≥ 6 drinks at one time).
14. Known Cushing's disease.
15. Known or suspected adrenal or ovarian androgen secreting tumors.
16. Allergy, known hypersensitivity or contraindication to the treatment medications used in this study including orlistat (This will include patients with chronic malabsorption syndrome or cholestasis or clomiphene (previous change in vision).
17. Couples with previous sterilization procedures (e.g. vasectomy, tubal ligation) whether or not it has been reversed.
18. Subjects with untreated poorly controlled hypertension defined as a systolic blood pressure ≥ 160 mm Hg or a diastolic ≥ 100 mm Hg obtained on two measures obtained at least 60 minutes apart.
19. Subjects who have undergone a bariatric surgery procedure in the past or are in a period of acute weight loss (defined as a weight loss of greater than 5% body weight in the last 6 months).
20. Known severe endometriosis
21. Anovulation or oligo-ovulation including hypothalamic amenorrhea, polycystic ovary syndrome, etc.
22. Donated semen.
23. Couples in which either partner is legally married to someone else.
24. Medical conditions that are contraindications to pregnancy.
25. Presence of severe, untreated psychiatric illness (major depression, substance abuse, eating disorder, etc.) that would, in the opinion of the site investigator, interfere with the patient's ability to successfully complete the study.
26. Any additional medical conditions that would be a contraindication to orlistat.
27. Any contraindication to study requirements including diet recommendations and activity requirements.
28. Currently participating in a lifestyle intervention program (such as Weight Watchers, Atkins Diet, Curves)
29. History of Gout.
30. History of pelvic radiation

5.3 Study Termination Criteria

1. Development or suspicion of an allergic or serious adverse reaction to any of the medications in the study.
2. Uncontrolled hypertension.
3. Persistent ovarian cyst > 30 mm mean diameter that does not resolve in two cycles.

4. Serious or severe Ovarian Hyperstimulation Syndrome (OHSS).

5.4 Study Enrollment Procedures

5.4.1 Recruitment

Clinical Practices of Investigators

Infertile couples presenting for consultation with an obese female and unexplained infertility (after clinical confirmation of their eligibility for admission into the study based on inclusion and exclusion criteria) will be approached to participate by the research coordinator or non-care providing physician. In the recruitment visit, study details including the expectations of lifestyle modification, as well as the experimental nature of the preconception intervention will be explained. Risks and benefits will be thoroughly discussed and consents given to the patient and partner.

Hospital/Local Health Care Referrals

Subjects will be recruited at each site from individual practice(s) of the investigators as noted above as well as faculty/resident clinics. Ongoing contact with practice and faculty members as well as with residents will be made by the investigators and coordinators, reminding them of the inclusion criteria, importance of the study, etc. In addition, the investigators will describe the study to members of other departments in the hospital, primarily family practice, medical endocrinology, urology, and gynecology who also see and treat these patients. Contact with local physicians will be made and/or grand rounds will be given to disseminate information about the study.

Referrals from Study Participants

Study participants often refer friends, acquaintances and colleagues to be potential participants.

Local Publicity Office

Investigators will meet with their local Public Relations offices and plan a news release about the study. They will also make themselves available for any newspaper, radio, or TV stories that may increase public awareness of the study. The full gamut of local media sources should be utilized. Often there is greater yield with more extensive coverage in smaller local outlets as opposed to brief mentions in outlets with larger circulation. News releases will mention the uniqueness of the study.

Local Advertisements

Advertisements will be placed in local newspapers and will be continued on a regular basis if response is good.

Contact with infertility support groups

Contact will be made with both national and local support groups to spread information about the study through informational brochures and/or participation in local meetings. The American Infertility Association also may be helpful in promoting awareness of this study.

National Professional Organizations

Contact will be made with the publicity office of The American Society for Reproductive Medicine, and other potentially helpful organizations to solicit their support and potential informational releases.

Web sites

The study will be prominently displayed on the RMN web site. Additionally each RMN center should have a web page devoted to this study with general as well as contact information. Information should also be available at the NICHD web site with links to each RMN center. An ad should also be placed at "Center Watch" on the web.

National Advertising

We would consider placing a trial ad in the health section of a select or a series of selected national publications with all of our local numbers/contacts.

IRB Approval

It is expressly acknowledged that all informational material that could be construed to be advertising will be approved by the appropriate IRB prior to dissemination.

5.5 Procedures for Tracking Sources of Subjects and their Disposition

We will track all contacts from subjects interested in the study. We will develop a pre-screening list that documents date and point of contact, eligibility based on telephone screening, and follow-up if subject meets prescreening and is interested in further participation. The consent form is mailed or emailed prior to the screening visit which is the next point of contact.

5.6 Obtaining Informed Consent

Once potential couples have been prescreened, they will be referred to the site clinical coordinator or his/her designee for a screening visit The consent will be explained to potential subjects and their partners by the coordinator or possibly the physician investigator depending on the clinical circumstances.

Inclusion and exclusion criteria will be reviewed. After the study has been completely explained to the potential couple, they will be given the informed consent documents to review (sample consent forms, in the format of Penn State University, are included in Section 10). Some individuals may wish to complete the informed consent process at the time of this discussion. In these cases the informed consent documents will be signed once all questions are resolved. In other cases the subjects may wish to take the consent forms home for further consideration. In these cases the coordinator will confirm the couple's willingness to be contacted, and set up a tentative timeframe to be back in touch with the subjects. The consents can be signed either with the coordinator or with a physician once all questions have been answered to the satisfaction of the potential patient and partner. A signed informed consent document, approved by the IRB at the study site, will be confirmed on all subjects prior to the baseline evaluation. The PI at each RMN site should not simultaneously be managing the care of a patient in the study and be the primary caregiver for a study participant, as specified in the NICHD clinical Research Policy guidelines.

In order to be eligible for enrollment and randomization, each member of the couple must be confirmed to meet all inclusion and exclusion criteria described above.

5.7 Intervention Group Assignment

After screening is completed the information will be uploaded to the WebEZ system electronically, and randomization will be performed by the program at the time of the randomization visit. Subjects will be immediately informed of their treatment assignment and orientation will begin.

6 Study Interventions

6.1 Interventions, Administration and Duration

<u>Phase I – Preconception Treatment</u>

<u>Overview</u>
Phase 1 will involve randomization to either an Intensive Lifestyle Modification program or a Standard Physical Activity Program. Each will last 16 weeks. The Intensive Lifestyle Modifciation Program will consist of an over the counter anti-obesity drug, orlistat, caloric restriction assisted by the use of meal replacements, and increased physical activity. The Standard Physical activity Program will consist of increased physical activity alone. All subjects will receive folic acid supplementation throughout all phases of the study.

Our goal in designing the lifestyle modification program is to develop simple methods that are easily comprehended by our subjects and study personnel. Therefore we are dispensing with labor intensive recall questionnaires of diet and activity that are frequently utilized in such studies but have little correlation with change in hard outcomes such as weight or biochemical parameters. We will create identical on-site in-person study visits and self-monitoring schedules for both lifestyle modification groups. We will utilize in both groups wireless devices that monitor activity (Fitbit) and weight (Fitbit Aria Scale) and automatically upload data to a central website (maintained by the DCC with appropriate consultants). This will enable both self-monitoring by the subject and central monitoring by the study personnel (who will provide devices, a web account, and joint access to the website). This will allow both subjects and study personnel the ability to view integrated and time related data and will also save time for subjects and study personnel. Finally, it allows a seamless record of weight and activity from preconception to conception to pregnancy to delivery. We will develop algorithms to address poor compliance with activity recommendations in both groups based on Fitbit self-monitoring as well as lack of weight loss within the intensive lifestyle modification with caloric restriction group. We will pilot text-based automated responses on smart phones to achieve these goals, as well as using more traditional (phone or email) methods for those without access to smart phones. Both groups will have similar formal site visit schedules and interactions with study personnel.

<u>Intensive Lifestyle Modification:</u> The primary focus of this modification will be to achieve weight loss as well as to increase physical activity. We will use a multifocal approach to achieve weight loss, utilizing meal replacements as well as a gastric lipase inhibitor (orlistat OTC - brand name Alli at 60 mg per dose) to prevent fat absorption. Multifocal approaches have been shown to increase weight loss beyond single therapy based interventions.

Medication (Orlistat) will be initiated at a dose of 60 mg per meal at lunch and dinner. We will skip the morning dose, as subjects have a low fat meal replacement (MR) breakfast: *<u>MRs as basis of a hypocaloric diet (~1200-1500 kcal/d).</u>* We, led by Dr. Penny Kris-Etherton, will develop a complete daily diet plan for the 16 week intervention plan with meal replacements that includes

two prepared entrees for lunch and dinner and a cereal bar or beverage. The meal replacements will be provided without cost to the participants in this treatment group. In addition, subjects will be instructed to consume two servings of fruit, 3 servings of vegetables, and two servings of low fat dairy per day. This diet will provide about 1100 calories with this macronutrient profile: 30% calories from protein, 45% calories from carbohydrate; and 25% calories from fat. Since we want to provide 1200 calories per day, there are 100 calories that can be consumed as desired. This meal plan totals approximately 1200 kcal/d and is consistent with the lifestyle modification in the Look AHEAD study.[79] Participants will be provided full instructions on how to use MRs and will develop a daily schedule for consuming them. At the randomization visit in Phase I, participants will receive instructions on ordering MRs directly from the Nutrisystem web site which will be shipped directly to their house. They will receive a monthly code to order further meal replacements. They will also at the randomization visit receive a two month supply of medication. We will ask subjects to weigh themselves at home on a weekly basis and will capture results automatically with the Fitbit Aria scale.

Increased Physical Activity Subjects will be asked to use the Fitbit Physical Activity Monitor during the screening phase to establish a mean number of steps over a 7 day period. This will then form the baseline number of steps. This baseline average of steps will be used as the starting point for increasing average steps a day by 500 steps a day per week until the desired maximum of 10,000 steps is reached. Subjects will be asked to maintain that level of physical activity throughout the study.

6.1.1 Determine Baseline Physical Activity

Subjects will be dispensed a Fitbit Physical Activity monitor during the randomization visit which will be used to determine average daily steps over a week's time.

Standard Physical Activity Intervention: This will be identical to the physical activity described above for the intensive physical activity group. There will be no dietary recommendations, no use of meal replacements, and no use of anti-obesity drugs. Subjects will have the same monitoring schedule both on site and off site as subjects in the intensive lifestyle modification.

Conception during Lifestyle Modification We will encourage intercourse without barriers during the lifestyle modification phase and welcome any pregnancies which happen. They will be managed identically as though that occur during the empiric infertility treatment phase of this study (Phase II below). Of course such pregnancies will be analyzed by treatment group according to intention-to-treat (ITT) principles. We are using safe and widely utilized lifestyle modifications. We are using an OTC weight loss drug, Alli, that has no known early adverse pregnancy effects and we will immediately discontinue it if pregnancy occurs. The ability to conceive at all points in this study will increase the appeal to participate and also the external validity of our results. No one with infertility wants to stop attempting pregnancy as part of infertility treatment. This also led us to reject using the oral contraceptive pill as a control arm for unexplained infertility.

Phase II – Standardized infertility treatment

After completion of Phase I (or 16 weeks of pretreatment), all subjects will enter Phase II. This phase will be universally administered to all participants in an open label, but standardized fashion. In this part of the study, subjects will receive three cycles of ovarian stimulation/hCG trigger for ovulation/ timed IUI and followed by midluteal visit. We will clamp weight and activity in both groups and continue to monitor with the Fitbit and the Aria scale. We will modify and streamline the study procedures from our AMIGOS study.

Phase III – Pregnancy
There will be no treatment intervention during pregnancy, though we will continue to monitor weight and have trimester study visits.

Phase IV – Pregnancy Registry
All subjects who deliver will be encouraged to participate in our Pregnancy Registry which currently provides infant follow up till age 3. This is currently a separate protocol and we will also obtain separate informed consent for participation in this protocol.

6.2 Handling of Study Interventions
All study interventions are FDA approved for the indication (i.e. orlistat to treat obesity), clomiphene to treat infertility or are common practices, i.e. prenatal and folic acid supplementation, meal replacements to achieve caloric restriction. They will be dispensed open label in concordance with policies at each site (i.e. some may require orlistat, though available over the counter to be dispensed from a research pharmacy). We will also use meal replacement gift certificates for purchasing meal replacements. Further details can be found in the Manual of Operations and Procedures.

6.3 Concomitant Interventions
No other interventions, other than those specified in the protocol are allowed to treat obesity or infertility during participation in the study. Exclusionary medications and conditions for randomization are also excluded during participation in the study.

6.4 Adherence Assessment
Adherence to the interventions during Phase I will be monitored by the use of the FitBit activity monitor and Aria Scale off site, and by monthly visits on site. Additionally pill counts and dietary logs will confirm adherence to the Intensive Lifestyle Modification program. We will also perform pill counts and collect the used hCG vial during the Phase II infertility treatment to confirm compliance. We will also collect intercourse and menstrual diaries during the study. We will make accommodations for those subjects who do not have regular access to the internet or a smart phone by arranging regular downloads of FITBIT data through alternate means at regular intervals (2 weeks).

7 Clinical and Laboratory Evaluations

7.1 Schedule of Evaluations

The table below (**Table 7**) summarizes the clinical and laboratory evaluations during the trial. A description of the visits follows.

Table 7: Study Visits and Procedures

Phases	Phase 1: Lifestyle Intervention (16 weeks)				Phase 2: Infertility Treatment						Phase 3: Pregnancy
Visit Titles	*Screen (can be done in 2 visits)*	*Baseline /Drug Assign*	*Month Visit 1, 2 & 3*	*End of Phase 1 (week 16)*	*Cycle 1 Baseline (day1-5 of menses)*	*Monitor Visit *(cycle day 8-12)*	*IUI*	*Urine Preg Test ***	*Serum hCG ****	*End of Phase 2*	*16, 24, 32 week Pregnancy Visit*
Informed Consent	X										
Medical Assessment	X										
Vital Signs & Biometrics	X	X	X	X	X					X	X
Complete Physical Exam	X										
Gynecological Exam and pap smear	X										
Acne/Hirsutism Assessment	X			X						X	
Transvaginal ultrasound	X				X	X					
Sonohysterogram or Hysterosalpingogram	X										
Questionnaires & QOL surveys	X			X						X	
Urine pregnancy test	X		X	X	X			X (@home)			
Serum pregnancy test									X		
Blood work for Rubella, Varicella, HIV (optional)	X										
Blood work for female hormones	X										
Blood work for safety labs, lipid panel and HgbA1C	X			X						X	
Blood work for central core	X	X	X	X	X					X	X
Blood work and urine for reserve ancillary studies (optional)	X	X	X	X	X					X	X
Blood work for Whole blood (optional)		X									
Blood work for RMN Repository (optional)		X									
Vaginal Swab	X				X						X
Rectal swab	X				X						X
Saliva sample (optional)		X		X							
Dispense meds		X	X		X		X				
Collect meds			X	X		X				X	
Order meal replacements (if applicable)		X	X								
Dispense Fitbit and Aria scale		X									
Dispense diaries		X	X		X		X				
Collect diaries			X	X		X				X	
Adverse Events & Concomitant Meds		X	X	X	X	X		X		X	X
Intrauterine Insemination							X				
MALE PROCEDURES											
Informed Consent	X										
Medical Assessment	X										
Vital signs & Biometrics	X									X	
Questionnaires & QOL surveys	X									X	
Semen analysis/collection	X						X				

Semen for RMN Repository (optional)	X			
Blood work and urine for RMN repository (optional)	X			
Saliva sample (optional)	X			

*Monitor visits will be repeated on an individual basis until hCG administration
**To be done 2 weeks after insemination at home. Repeat cycles until pregnancy occurs or treatment cycles end; may have hCG up to 3 times (cycles). If positive pregnancy, will schedule visit for serum pregnancy test and move to Phase 3 for follow-up. If negative, start new cycle.
***Will repeat hCG 2 days after + result. OB ultrasound will be scheduled 14-21 days from + result.

7.2 Timing of Evaluations

Screening visit(s):

Although we anticipate that the items described below could be completed in a single visit, we will allow coordinators to divide them into two visits. For example, if the subject or couple has had little to no prior evaluation, it may be time and cost effective to obtain parameters in a stepwise fashion. We anticipate that consent, biometrics, and medical history would be obtained at first visit, whereas we would distribute kits to collect saliva, stool and dispense the FitBit activity monitor at the second visit for subjects with a high probability of randomization.

1. Obtain informed, signed consent from female and male partner.

   Female and partner must specifically be made aware of potential drug complications and adverse outcomes, and partner must sign the Male Consent Form.

2. Complete medical history and counseling of the couple

   Pre-conception counseling

   Female and partner must complete a Genetic Risk Factors questionnaire and RMN Medical History questionnaire

3. Complete Questionnaires.
   - Female: Female Sexual Function Index (FSFI), SF-12, PHQ-9, FertiQoL, Epworth Sleepiness, Stop-Bang (Sleep apnea), Female Sexual Distress Scale (FSDS), Automated Self-Administered 24-hour Recall (ASA24), and Diet History Questionnaire (DHQ II)
   - Male Partner: SF-12, PHQ-9, FertiQoL, Epworth Sleepiness, Stop-Bang (Sleep apnea), International Index of Erectile Function (IIEF), ASA24, and Diet History Questionnaire (DHQ II), qADAM Questionnaire, and Psychosexual Daily Questionnaire (PDQ)

4. Physical Exams of Male and Female Subject
   - Female
     - Vital signs, height, weight, hip and abdominal circumference, BMI
     - Pap smear if necessary per current ACOG time-frame guidelines

Table 8: Female Local Laboratory Screening Blood Tests

- Standard pelvic and breast exam conducted by physician (or within past 12 months)
- Ferriman-Gallwey hirsutism scoring, acne lesion count

o Male Partner
  - Vital signs, height, weight, hip and abdominal circumference, BMI

5. Perform radiological exams.

   Transvaginal ultrasound measuring uterine and ovarian characteristics

   Sonohysterogram or hysterosalpingogram to verify patency in at least one tube, and normal uterine cavity (or documentation within past 3 years). An uncomplicated intrauterine non-IVF pregnancy and uncomplicated delivery and postpartum course resulting in live birth within the last three years will also serve as sufficient evidence of a patent tube and normal uterine cavity as long as the subject did not have, during the pregnancy or subsequently, risk factors for Asherman's syndrome or tubal disease or other disorder leading to an increased suspicion for intrauterine abnormality or tubal occlusion.

6. Laboratory tests.

   Collect blood for the following tests for inclusion/exclusion (Table 8) (if not already obtained)

   -Offer optional blood test for Rubella, Varicella, and HIV. The costs for these blood tests are not included in determining the patient care budget for this protocol.

   -Collect central core blood specimen to be sent to a central laboratory for testing

   -At each blood draw during the study, retain a reserve blood and urine specimen (aliquotted to at least 3 cc each) for banking on site for future ancillary studies as plasma and serum, if subject has consented for optional blood and urine collection.

   -Collect vaginal microbiome swab and rectal microbiome swab.

**FEMALE LOCAL LABORATORY SCREENING BLOOD TESTS**	
**Eligibility Labs (within 1 year)**	**Safety Screen (fasting) Labs**
AMH*	Urine pregnancy test
Prolactin	Hemoglobin
TSH	Hematocrit
HgbA1c	WBC
	Platelets
	BUN
	Creatinine
	Total Bilirubin
	ALT/AST

	Total Cholesterol
	HDL-C
	Triglycerides

In lieu of AMH to determine ovarian reserve to meet inclusion criteria, investigators can obtain or use a prior Day 3 (+/-2 day) FSH and Estradiol

Eligibility tests in Column 1 are valid for one year prior to randomization.

Safety Screen tests, except the lipid panel must be obtained within 60 days prior to randomization.

Lipid Panel (cholesterol, triglycerides, HDL) results are valid for one year prior to randomization.

Males: obtain a semen specimen.

- Local lab semen analysis (or documentation within 1 year)
- Retain semen sample for future microarray analysis if RMN Biologic Repository consent is signed
- Collect urine and blood sample for RMN Biologic Repository if consent is signed
- Collect saliva sample if consented

7. Distribute Testing and Study Supplies
    a. Distribute salivary collection kit, if consented.

<u>Randomization Visit:</u>

Randomization visit can take place regardless of menstrual cycle day and as soon after screening visit as subject and couple meet inclusion/exclusion criteria. Subjects will be informed in person of their randomization assignment. This is to allow for in person counseling of the lifestyle modification and answer face to face any concerns about their treatment assignment. Subjects will undergo during this visit: extensive orientation to the goals of the lifestyle modification, dispensing of all supplies and medications, and review of tracking and self-reporting requirements.

1. Perform limited physical exam, including weight and vital signs.

2. Orient subject to study treatment group including activity recommendations and use of meal replacements and orlistat.

3. Dispense study supplies such as Aria Wifi scale and Fitbit Activity tracker.

4. Dispense weight loss medication (orlistat) and provide instructions for ordering meal replacements on Nutrisystem's website to subjects randomized to the extensive lifestyle modification arm.
5. Dispense intercourse and menstrual diaries.

6. Prenatal vitamins with folic acid dispensed.

7. Collect central core blood specimen as well as urine and blood reserve sample for ancillary studies (to be stored on site) (with consent). This will include hormonal assays for sex steroids (E2, P4, Testosterone), other reproductive hormones (SHBG), adipokines (leptin, adiponectin) and other hormonal and metabolic parameters that are determined relevant to the study.

8. Collect blood for DNA for the pharmacogenomic studies (with consent) to be stored on site.

9. Collect all available biospecimens from subject and male partner for RMN Biologic Repository (with consent).

10. Collect saliva specimen (with consent).

Monthly Visit during Lifestyle Modification Phase (Phase 1) (N = 3)

1. Perform limited physical exam, including weight, waist circumference, and vital signs (respiratory rate, pulse, and blood pressure).

2. Perform urine pregnancy test.

3. Collect menstrual and intercourse diaries, dispense new for next month.

4. Collect remaining study medications and perform pill count.

5. Dispense study supplies and medications, Prenatal vitamin, if applicable, orlistat.

6. Ensure that meal replacements for the next month have been ordered through Nutrisystem.

7. Collect adverse events.

8. Collect blood and urine reserve sample for ancillary studies (to be stored on site) and central core specimens which will be run at a core lab facility.

9. During the 3rd monthly visit, dispense saliva kit for collection to be returned Phase 1 closeout, if consented.

Lifestyle Modification Closeout Visit (Week 16)

Subjects will undergo a repeat baseline visit at the end of their 16 week lifestyle modification phase. This will again be independent of cycle day. We will not repeat breast and pelvic exam,

pap smear or other items required for inclusion in the study that were obtained at the baseline or screening visits. Intercourse and menstrual diaries will be collected. Female study participants will have a limited physical exam. We will defer transvaginal ultrasound exam until the baseline visit of the infertility treatment cycle. We will obtain questionnaires and biospecimens.

1. Perform limited physical exam, including weight, waist circumference and vital signs (as above).

2. Perform urine pregnancy test.

3. Collect menstrual and intercourse diaries.

4. Collect remaining study medications and perform pill count.

5. Collect adverse events.

6. Collect blood and urine reserve sample for ancillary studies (to be stored on site) and central core specimens which will be run at a core lab facility. Collect specimens for repeat safety screening (Table 8).

7. Collect saliva sample.

8. Repeat full screening visit including hirsutism and acne scoring, QOL questionnaires (excluding DHQII) (See above screening visit) with the exception of full physical exam and transvaginal ultrasound (which will be performed at the start of the subsequent infertility treatment baseline visit).

Baseline Ultrasound Visit for Empirical Infertility Treatment (Phase 2)

This baseline visit will be performed at the start of Cycle 1 only. The initial dose of clomiphene citrate will be 100 mg/day, which may be adjusted upwards or downward depending on response in subsequent cycles. Women may undergo a total of up to 3 cycles culminating in hCG administration. Subjects may begin subsequent cycles immediately following failed cycles, as long as they meet baseline criteria of a negative urine pregnancy test. Subjects may continue in the study after one failed cycle (i.e. either no development of follicles on study medication). If a subject experiences a second failed cycle, it is recommended to discontinue the subject from the study.

1. Subject will call with menses and come in between days 1-5 of their menstrual cycle (Preferably day 3 for baseline monitoring visit).

2. Check Urine Pregnancy test.

3. Obtain weight.

4. Perform baseline ultrasound. If subject has a cyst with a mean diameter of >30mm, do not continue the visit. Have the subject return with her next menses for a repeat baseline visit.

5. Collect vaginal microbiome and rectal microbiome swab.

6. Dispense Infertility Treatment Study Medication (see Table 9).

7. Obtain central core lab specimens and blood and urine reserve (to be stored on site) for ancillary studies (with consent).

8. Dispense menstrual and intercourse diaries.

9. Schedule Day 8-12 visit for follicular monitoring.

Table 9: Study Medication and Dosing

Protocol	interval	dose	method	start	finish	future cycles
CC	Daily	100 mg	pill	Day 3*	Day 7	Can be started at 50-150 mg/d= (1 to 3 pills)

*(+/- 2 days)

Empirical treatment Cycle Visit number 2:
Within 3 days after completing 5-day drug cycle (Cycle Day 8-12)

1. Transvaginal ultrasound for endometrial and follicular monitoring.
2. Adverse effects and concomitant medications query.
3. During cycle 2 & 3, medication bottles and menstrual diaries from previous cycle will be collected.

Study treatment Cycle visits number 3 and beyond:
Visits for subjects will be conducted on an individualized basis until hCG administration. When a decision has been made to give hCG on the next day, a follow up monitoring visit on that day is unnecessary.

1. Transvaginal ultrasound examination for endometrial and follicular monitoring.
2. Adverse effects and concomitant medications query.

hCG administration day:
1. 10,000 IU in 1 cc diluent delivered IM in clinic if applicable, or subjects will be instructed in the use of intramuscular hCG and a written plan for its use will be provided.
2. Transvaginal ultrasound examination for endometrial and follicular monitoring is not necessary if one has been performed within 2 days.

Criteria for hCG administration:

1. First occurrence of lead follicle reaching 20 mm (in average diameter in two dimensions), or
2. First occurrence of two lead follicles greater than 18 mm diameter (in average diameter in two dimensions), or
3. The day after the lead follicle reaching 18 mm (in average diameter in two dimensions), or
4. The day of detection of presumptive ovulation by ultrasound.

Criteria for withholding hCG:

1. If a leading follicle does not reach a mean diameter of 18 mm after 18 days of treatment, or
2. Endogenous LH surge happens [which can lead to premature luteinization], or
3. Increased risk for OHSS and/or high-order multiple gestational pregnancy exists when more than 4 growing follicles develop (mean diameter >18 mm)

**Protocol medication adjustment:*

1. If no ovulation or development of at least one follicle ≥ 18 mm in average diameter, then increase dosage in the subsequent cycle.
2. If only one follicle ≥ 18 mm in average diameter, the dosage increase for subsequent cycles will be left to physician's discretion.
3. If more than four follicles are ≥ 18 mm in average diameter, dosage will be reduced in the subsequent cycle.

**Cancellation criteria:* Cycles will be cancelled if significant adverse reactions develop in response to administered medications, if criteria for withholding hCG administration are encountered, or per patient's request.

Insemination protocol:

One insemination will be performed within 44 hours after hCG administration, and time of insemination from hCG administration will be recorded. For inseminations, each site will utilize its standard semen preparation method and catheter.

Insemination day visit:

1. Each site will perform its standard insemination procedure.
2. Record parameters of semen analysis before and after sperm preparation.
3. Record time of insemination.
4. During cycle 1 & 2, medication for the next cycle will be dispensed along with menstrual diaries. The subject will be instructed that this medication SHOULD NOT be started until the current cycle is completed, either pregnancy or no pregnancy has been confirmed with a pregnancy test and the subject has spoken with the site. The subject will be instructed to perform a urine pregnancy test (see below under Urine pregnancy test) two weeks after the insemination.

Urine Pregnancy test:
To be conducted 2 weeks after insemination at home or at start of menses.

Patient will perform a urine pregnancy test at home and call the site with the results. If the test is positive, the patient will be scheduled for a serum hCG test. If the urine pregnancy test is negative, and menses have begun, instructions will be provided to begin the next cycle. If the urine test is negative and menses has not begun, the subject should come in for a serum pregnancy test and the site should follow standard practice for a patient with no menses following IUI.

Serum hCG Visits:
To be conducted after + urine pregnancy test

1. Obtain blood sample for qHCG. If positive result, will schedule for repeat 2 days later.

Three possible outcomes:

- **A.** No pregnancy
- **B.** Positive test→ retest for rising level in 2 days→ no pregnancy
- **C.** Positive test→ retest for rising level in 2 days→ positive pregnancy

A. Negative Serum or Urine Pregnancy Test/No pregnancy

Repeat cycles until pregnancy occurs or treatment cycles conclude; women may receive hCG up to 3 times (cycles). There may be anticipated or unanticipated breaks in protocol due to medical necessity, patient request or due to site holiday recess, but none of the breaks should exceed approximately 4 weeks (allowing the subject approximately 16 weeks to complete Phase 2 of the protocol).

Criteria to initiate Cycles 2 or 3 (Menstrual Cycle Day 3 +/- 2 days):

1. Negative urine pregnancy test (within 2 days)

B. Positive Pregnancy Test (no pregnancy)

Patient will return in 2 days to check for a rising level in serum βhCG; if there is not a rising level, patient will initiate Cycles 2, or 3 (see above)

C. Positive Pregnancy Test (pregnancy)

Biochemical pregnancy will be defined as serum hCG >5 units/ml (or >10 depending on local laboratory) 2 weeks after insemination followed by a rising level two days later.

- Patient will return in 2 days to check a rising level in serum βhCG; threshold level of 2,000-4000 mIU/mL will be obtained
- Schedule Pregnancy Ultrasound Visit
- Schedule End of Study Drug Visit

Pregnancy ultrasound(s):

To be done 14-21 days after a positive pregnancy test

Clinical pregnancy rate will be defined as the identification of intrauterine sac(s) with positive fetal cardiac activity in at least one sac, 2 weeks after biochemical pregnancy identification.

1. Use to determine location of pregnancy and number of implantation sites
2. Repeat in 7-14 days if no cardiac activity detected
3. Arrange obstetrical follow-up as soon as cardiac activity is observed and there are no pregnancy abnormalities requiring further monitoring.

Pregnancy Follow up:

1. Prenatal records will be requested from the treating physician.
2. Copy of each prenatal visit records to be kept until delivery
3. Hospital record of delivery outcome will be obtained from patient's labor & delivery hospital (these will include both maternal and infant hospital records).
4. Obtain a separate consent form to enter the patient into the pregnancy registry.

End of Empirical Infertility treatment study (Phase 2) Drug Visit:

This visit will mirror the end of Phase I visit with the exception that an additional ultrasound will not be performed specifically for this end of study visit, and we will repeat biometric measurements and QOL surveys in the male. We will repeat most of the parameters obtained at the baseline visit:

If pregnant: perform as early in the visit as possible, skip the FertiQoL questionnaire.

If no conception within 3 cycles: perform after the pregnancy test from the third cycle of therapy is returned negative, showing the subject is not pregnant

Other: Study withdrawal

1. Physical exam.
    a. Height, weight, waist and hip circumference
    b. Vital signs: respiratory rate, pulse and blood pressure
    c. Ferriman-Gallwey hirsutism assessment, acne lesion count.
2. Administration.
    a. Subjects will return remaining study drug.
    b. Subjects will turn in journal logs; physician will review data.
    c. Query for adverse events and concomitant medications.
    d. Females will repeat QOL surveys (excluding DHQII).
        - Pregnant women will not repeat FertiQoL.

    e. Males will return for biometric measurements and repeat QOL surveys (excluding DHQII).
3. Laboratory tests.
    a. Repeat the same panel of serum tests including safety labs.
    b. Obtain Biospecimens for ancillary studies, if consented, and blood for central core lab.

Figure 4: Modified Ferriman-Gallwey Hirsutism Scale

Pregnancy Visits per trimester (Phase 3) (at 16, 24, and 32 weeks)

1. Perform limited exam including weight, waist circumference, and vital signs (as above).
2. Obtain central core blood and urine and blood reserve for ancillary studies (store on site) (with consent).
3. Obtain vaginal microbiome and rectal microbiome swab.
4. Collect adverse pregnancy events.

Phase 4: Pregnancy Registry

All subjects who deliver will be encouraged to participate in our Pregnancy Registry which currently provides infant follow up till age 3. This is currently a separate protocol and we will also obtain separate informed consent for participation in this protocol.

7.3 Special Instructions and Definitions of Evaluations

7.3.1 History and Demographics

Medical history, including infertility treatment history, and concomitant medicines will be obtained using standardized procedures and case report forms (CRFs) developed by the RMN.

7.3.2 Physical Exam

A physical exam with a standard pelvic and breast exam will be performed on all patients by a study physician. Height, weight and waist and hip circumferences will be recorded to the nearest 0.1 cm, 0.1 kg and 1 cm, respectively. Waist will be measured at the level of the umbilicus and hip circumference will be measured at the widest diameter. Participants will be weighed while dressed in light clothing, without shoes. Weight and waist circumference will be collected at each site visit. Blood pressure will be determined in the right arm in the sitting position, and a large cuff will be used as necessary. Blood pressure will be assessed at each visit. Elevated blood pressures (≥160/100) will be repeated following acclimation to the study environment. All patients aged 21 and older should have had a normal Pap smear in accordance with current ACOG guidelines[80]; otherwise, one should be performed at the screening exam. Subjects with cytological abnormalities will need to have these resolved prior to study entry.

A hirsutism assessment will be made via the modified Ferriman-Gallwey hirsutism score (**Figure 4**) by trained study personnel [81].

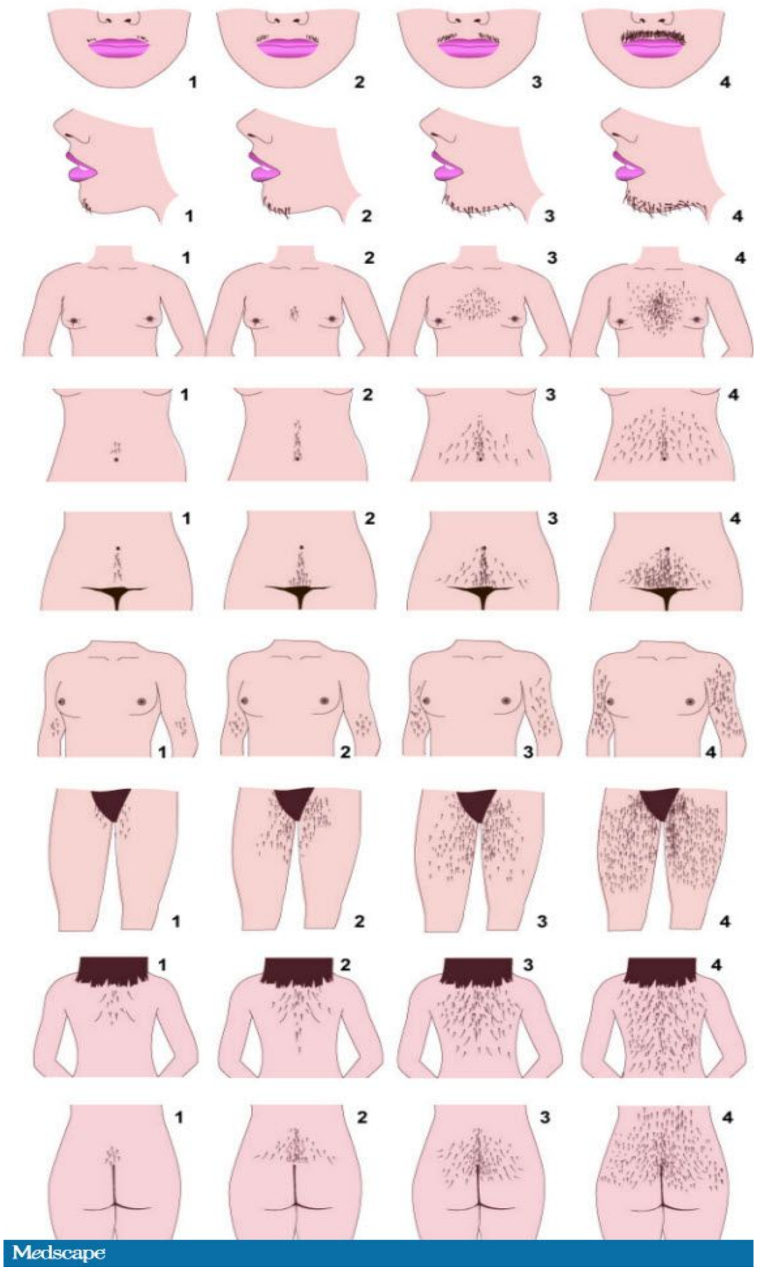

An acne assessment will be made by trained personnel using a standard acne lesion assessment (count) diagram and definitions (**Table 10**). Photographic examples of each grade will be provided to investigators as well as training to study personnel. When counting facial acne lesions, it is important that all lesions be counted, both non-inflammatory and inflammatory, examining areas of the forehead, cheeks, and chin and avoiding the nose.

Table 9: Acne Grading Method

Name	Description
Open	Results when residual skin oil, makeup, dirt, dead skin, and small hairs impact

Comedone (Blackhead)	a sebaceous follicle and prevent the pore from functioning correctly. If the pore is open, the comedone will be dark in appearance and is thus called a blackhead.
Closed Comedone (Whitehead)	Non-inflammatory comedone with a white center.
Papule	An inflammatory comedone that resembles a small red bump on the skin.
Pustule	An active infection of the skin that consists of dead skin cells and bacteria. These lesions are spherical in appearance and are filled with pus. Often reddish in color, pustules may be painful and will break open easily if scratched or bumped.
Nodule	A natural progression of a papule. They appear very similar to papules, but are inflamed and penetrate deep into the skin. They are often very painful.

7.3.3 Ultrasound and Imaging Exams

An ultrasound exam will be performed with a transvaginal probe. The following measures will be obtained: uterine dimensions, leiomyoma presence size and location, other uterine abnormalities (Mullerian or vascular), endometrial thickness, ovarian size in three dimensions, the size of the largest ovarian follicle, antral follicle count in each ovary (complete ovary) and ovarian morphology. Endometrial thickness is the largest anterior-posterior measurement of the endometrium in the sagittal plane. Ovarian size is determined by measuring the largest plane of the ovary in two dimensions and then turning the vaginal probe 90 degrees and obtaining a third measurement. Ovarian volume is determined by the formula for a prolate ellipsoid (length x width x height x $\pi/6$)[82]. This same ultrasound will be performed at each subsequent ultrasound visit (including follicular monitoring visits during Phase II). If the patient has had no prior test of tubal patency, this may be the desired time to perform a sonohysterogram to determine tubal patency.

7.3.4 Biospecimens

Serum: A phlebotomy will be performed at the appropriate visit. Blood will be obtained in the appropriate tubes for specimen processing and eventual aliquoting. If a subject has been fasting, this will be the first order of the study visit agenda, and subjects will be given an appropriate snack or drink after to ease hunger.

Blood for DNA: Whole blood will be collected (if consented) in an EDTA tube for eventual extraction of DNA from WBCs. This will be frozen after collection.

Urine: A urine specimen will be obtained at the visit and aliquoted into the approiate storage units.

Stool Microbiome: During a pelvic exam, a Dacron swab will be used to sample the rectum and placed in a NAT (Nucleic Acid Transport) collection tube. After collection, the samples will be refrigerated until processing. This collection method will allow for DNA analysis of the stool microbiome.

Vaginal Microbiome: During a pelvic exam with speculum in place in the vagina, Two Dacron swabs will be used to sample the posterior fornix and cervix and placed in a NAT (Nucleic Acid Transport, CentraCare Laboratory Services, St. Cloud, MN) collection tube. After collection the samples will be refrigerated until processing to preserve the DNA material from degradation. This collection method will allow for DNA analysis of the vaginal microbiome.

Salivary Analytes: A salivary collection kit will be used (SalivaBio Oral Swab (SOS)). This will be dispensed on the visit prior to the collection visit. A swab will be collected and placed in the collection tube for preservation and processing according to standard operating procedures (https://www.salimetrics.com/collection-systems). This will be collected at home at 9pm on the evening before the visit, and at 9am the day of the visit (in clinic if an am visit). The goal is to measure cortisol at the respective peak and nadir of the diurnal cycle.

Biospecimen Disposition: Specimens obtained under consent for the FIT-PLESE study will be aliquoted for storage at the study site, shipment to the RMN Central Core Laboratories, or shipment to the RMN Biologic Repository. Additionally subjects will be offered the possiblility to sign a separate consent to participate in our Registry Protocol, and will contribute additional specimens as indicated by that protocol. Further details of specimen processing can be found in the Manual of Operations and Procedures.

7.3.5 Questionnaires

Mood, quality of life, and sexual function will be assessed at baseline and at the end of the study visit. Quality of life and mood will be assessed by the PHQ-9[83] and Short Form 12 (SF-12).[84] Female sexual function will be assessed by the Female Sexual Function Inventory (FSFI) along with the Female Sexual Distress Scale (FSDS).[85] This measure is considered the “gold standard” paper and pencil assessments of sexual function and has excellent psychometric properties.[85] Male sexual response will be assessed by the International Index of Erectile Function (IIEF), a multidimensional scale for assessment of erectile dysfunction. The measure addresses the relevant domains of male sexual function (erectile function, orgasmic function, sexual desire, intercourse satisfaction, and overall satisfaction).[86] We will assess hypogonadal symptoms with the Androgen Deficiency and the Aging Male quantitative (qADAM) questionnaire and psychosexual function with the Psychosexual Daily Questionnaire (PDQ). We will assess quality of life relating to infertility and treatment with the FertiQoL survey.[87] There may also be some impact on fertility and we will investigate this in our study. We will use two simple scales to evaluate sleep, the STOP BANG questionnaire for sleep apnea,[88] and the Epworth Sleepiness Scale.[89] The Automated Self-Administered 24-hour Recall (ASA24) and Diet History Questionnaire II (DHQ II) will be used to assess past and current dietary intake.

8 Management of Adverse Experiences

8.1 Expected Adverse Experiences

All medications hold the possibility of an allergic reaction.

8.1.1 Phase I

There are no known side effects from meal replacements, except perhaps increased hunger. Orlistat is associated with gastrointestinal symptoms, most commonly flatulence, steatorrhea, and diarrhea. Prenatal vitamins may be associated with nausea or constipation (especially if they include iron). Increased physical activity may be associated with musculoskeletal pain. Pregnancy during Phase I is not an adverse event.

8.1.2 Phase II

The expected side effects with clomiphene include hot flashes, nausea, abdominal/pelvic pain and bloating, and mood changes. Rarely visual changes are noted. hCG given parenterally is associated with the risks of an intramuscular injection including pain at the injection site, bleeding, and infection. Intrauterine insemination is associated with the risk of pain and cramping after the procedure, spotting and less frequently bleeding and rarely pelvic infection. Both clomiphene and hCG alone and in combination are associated with an increased risk of multiple pregnancy. In the AMIGOS trial the rate of multiple pregnancy after conception was 7.6%.

8.1.3 Expected Serious Adverse Events

We do not anticipate serious adverse events during the first phase of this trial with lifestyle modification. These are more likely during the second and third phases of the trial. The expected serious adverse events are likely related to ovarian cyst formation which can result in hospitalization or surgery. Pregnancy and especially multiple pregnancy may also be a source of ectopic pregnancy, prematurity and maternal complications such as pre-eclampsia or eclampsia requiring hospital admission and induction of labor. None of the drugs or interventions are associated with increased risk of congenital anomalies.

9 Criteria for Modification or Discontinuation of Study Interventions

9.1 Phase I

There are no predetermined criteria for discontinuation during Phase I. Subjects will be allowed to continue in the trial even if they do not achieve the goals of the two interventions and eventually receive the infertility treatment in Phase II. However each subject will have to complete the 16 week pretreatment period before receving the Phase II treatments. Subjects can not be accelerated into Phase II.

9.1.1 Intensive Lifestyle Modification

Orlistat intolerance: Subjects who are unable to tolerate the dose of orlistat will be allowed to skip doses at specific mealtimes to ease symptoms or to discontinue altogether with continued intolerance based on the decision of the site PI.

Algorithm for poor responders: Subjects who fail to lose appropriate weight in the intensive lifestyle modification group or fail to meet physical activity goals in either group will be contacted by the study coordinator on a weekly basis to review reasons. We will pilot smart phone automated responses also within this trial. Drs. Kris-Etherton and Sarwer will lead the training (prior to the start of the study) and supervision (through monthly conference calls) of the interventions as well as review on a weekly basis of persistent poor responders and excessive responders (see below).

Algorithm for excessive responders: Excessive and rapid weight loss can be associated with health risks including formation of gall stones, development of hypotension, loss of energy, but rarely by an attack of gout. Subjects will be counseled at the onset about these health risks and to avoid excessive weight loss. Excessive response will be defined as a weight loss exceeding an average of 2.5 pounds per week or 10 pounds in a 4 week period. Subjects who exceed this at a monthly visit will be counseled to increase their caloric intake, by approximately 300-500 kcal/d, to slow the weight loss to our prescribed limits. We will follow subjects with their weekly weights collected by their Fitbit Aria wireless scales, and discuss them at both monthly investigator conferences and weekly coordinator conferences until they meet our guidelines. We will monitor the Fitbit steps during the first month of the study online to ensure that subjects do not exceed our guidelines of increasing 500 steps a day. We will also review monthly Fitbit records to ensure that subjects do not exceed the 10,000 steps on a regular basis. Subjects who consistently exceed our guidelines (more than 7 days in a 28 day period) will be advised to lower their activity to meet guidelines, and this will become a topic for weekly study coordinator and monthly investigator phone conferences

9.1.2 Standard Lifestyle Intervention

Algorithm for poor responders: Subjects who fail to meet physical activity goals or who lose or gain excessive weight will be contacted by the study coordinator on a weekly basis to review reasons. We will pilot smart phone automated responses also within this trial. Drs. Kris-Etherton

and Sarwer will lead the training (prior to the start of the study) and supervision (through monthly conference calls) of the interventions as well as review of persistent poor responders.

Algorithm for excessive responders: We will monitor the FitBit steps during the first month of the study online to ensure that subjects do not exceed our guidelines of increasing 500 steps a day. We will also review monthly Fitbit records to ensure that subjects do not exceed the 10,000 steps on a regular basis. Subjects who consistently exceed our guidelines (more than 7 days in a 28 day period) will be advised to lower their activity to meet guidelines, and this will become a topic for weekly study coordinator and monthly investigator phone conferences.

9.2 Phase II

Criteria for altering the clomiphene dose and withholding hCG are noted above in description of the visits. Additionally a change in visual symptoms determined by the site investigator to be caused by clomiphene administration should be considered as a reason for discontinuing further challenge with clomiphene.

9.3 Phase III and IV

There are no study interventions during this part of the study.

10 Statistical Considerations

10.1 General Design Issues

This will be a randomized open label clinical trial of two types of lifestyle modification: one intensive focusing on weight loss through caloric restriction and anti-obesity medication, and the other less intensive (“standard”) focused on increasing physical activity for 16 weeks followed by three cycles of empiric ovarian stimulation combined with intrauterine insemination in obese women with unexplained infertility. We will track all pregnancies to completion. Subjects will be randomized 1:1 to the two treatments and stratified by site.

10.2 Rationale for Design

Obesity is associated with subfertility through multiple mechanisms and lifestyle modification is frequently recommended as primary treatment before initiating infertility therapy. However this common sense recommendation is tempered by data that suggest that excessive or rapid weight loss as well as excessive physical activity may be associated with lower conception rates. We propose to focus on obtaining reproductive fitness through two types of lifestyle modification prior to infertility treatment. The first will be an intensive lifestyle modification designed to improve fitness through weight loss (with a dual approach of meal replacements combined with an anti-obesity medication) AND by increasing physical activity and the second arm will be a standard lifestyle modification which will focus on increasing physical activity alone. The meal replacements will be considered a prescribed medical treatment and will be dispensed and tracked to the intensive lifestyle intervention group throughout the pre-conception intervention. Both groups will be encouraged and monitored to achieve the same amount of physical activity. We hypothesize that weight loss as achieved by the more intensive intervention will be superior to the likely weight maintenance achieved with physical activity alone. However as noted in the background there are few data beyond our preliminary data to support that hypothesis. We do not have a control arm of no treatment as currently lifestyle modification is recommended universally for obese women.

10.3 Outcomes

10.3.1 Primary Outcome Measurements

The primary outcome for this trial is the cumulative good birth outcome defined as a singleton or twin infant born at ≥ 37 weeks between 2500 and 4000g without a major congenital anomaly.

10.3.2 Secondary Outcome Measurements

We will assess secondary outcomes including live birth, time to pregnancy, pregnancy loss rate [including ectopics and Pregnancies of Unknown Location (PULs)], multiple pregnancy rate, pregnancy complication rate including development of gestational hypertension and diabetes, mode of delivery, birth weight, neonatal complication rate, predictive factors for response including DNA polymorphisms, salivary cortisol, quality of life, and cost effectiveness. Additionally we will be examining changes in the microbiome and association with outcomes.

10.4 Sample Size and Accruals

10.4.1 Sample Size and Power Calculations

Estimates used for the power calculation are based on our experience from the OWL PCOS and AMIGOS trials as provided in the preliminary data section. The primary outcome for this trial is a good birth outcome defined as an infant (singleton or twin) born at ≥ 37 weeks between 2500 and 4000g without a major congenital anomaly. We will examine the cumulative rates between groups. We anticipate the proportion of good birth outcomes to be 0.25 in the standard lifestyle intervention (physical activity and weight maintenance) arm and 0.40 in the Intensive lifestyle modification arm. We anticipate that there will be an increased proportion of good birth outcomes in each group beyond our preliminary data, as we will not actively prevent pregnancy during the lifestyle intervention; therefore, they will have an additional 16 weeks to conceive prior to initiating empiric treatment. A sample size of 190 per treatment arm will provide 80% power to detect a 0.15 absolute difference in the proportion of good perinatal births (0.25 in the standard lifestyle modification and 0.40 in the intensive group using a two-sided test having a significance level of 0.05. Although the randomization is stratified by two factors (recruitment site and BMI status), this factor was not taken into consideration for the power analysis to be conservative because accounting for the correlation of these covariates with the outcome would simply increase the precision, and hence, increase the power. The sample size has been inflated to a total of 380 participants to allow for a dropout rate of 20%. We believe this 20% dropout rate is a reasonable estimate based on current experience in our PPCOS I, PPCOS II, OWL PCOS, and AMIGOS trials. The table below summarizes other possible sample size scenarios having a 0.15 absolute difference in the proportion of good birth outcomes between the two treatment arms, all of which we would have excellent power to detect (**Table 11**).

Table 11: Sample size scenarios based on varying assumptions

Standard Lifestyle Modification: Physical Activity Arm having a Good Birth Outcome	Intensive Lifestyle Modification Weight Loss and Physical Activity Arm: Proportion having a Good Birth Outcome		Significance Level (alpha)	Power (%)	N per Arm (0% Dropout)	N per Arm (20% Dropout)
0.15	0.30		0.05	80	121	152
			0.05	90	161	202
0.20	0.35		0.05	80	138	173
			0.05	90	185	232
0.25	0.40		0.05	80	152	190
			0.05	90	203	254

10.4.2 Accrual

A total of 2.6 years will be required to complete the study after start up; 15 month enrollment period (based on 2 subjects per site/month x 13 sites), 7 month treatment period (4 months

lifestyle pretreatment and 3 months ovarian stimulation/IUI), with 9 month additional observation to determine pregnancy outcomes.

10.5 Data Monitoring

There will be no pre-determined stopping rules and no interim analysis.

10.6 Study Monitoring

A monitoring plan that satisfies the ICH/GCP guidelines for clinical monitoring will be used. A Project Manager from the DCC will lead this effort, and report findings to the DCC PI. The Project Manager will have full knowledge of the study protocol, Manuals of Procedures, and is familiar with the database system and is trained to review patient charts. The Project Manager will be responsible for training and supervising other personnel.

Once personnel at participating site are trained to recruit patients, the Project Manager will be sent to the site to help initiate the study according to the study protocol, and to ensure that the clinical site meets the scientific, clinical, and regulatory requirements. For example, the Project Manager will review all signed and dated forms (such as financial disclosure forms), the curriculum vitae and certifications of the investigators and personnel, CRF training, and the written IRB approval of the protocol and consent form.

The on-site monitor will return to the clinical site after a defined number of patients are recruited (can be as early as the recruitment of the 2nd patient) or a certain time period has passed, depending on the duration of the protocol execution. The schedule of visits will be discussed and agreed in the Steering Committee and we anticipate that the Project Manager will visit each participating site at least once.

During the site visit, the clinical sites should provide to the monitor a space and access to all relevant records including medical records and regulatory binders, and there would be immediate verbal feedback provided to the site after original source documents are compared to entries in the CRF. The clinical sites must agree to cooperate with the monitor to ensure that any problems detected in the course of these monitoring visits are resolved. The on-site monitor will conduct an audit of a random sample of entered information against the source documents, a review of all regulatory documents, a review of all informed consents, and a review of all pharmacy logs. The clinical site PI and coordinator should be available to meet the monitor during the visit. The monitor will review electronic data from all sites, providing a method for identifying systematic errors or problems.

To assure Good Clinical/Laboratory Practice, the monitor will control adherence to the protocol at the clinical sites and evaluate the competence of the personnel at the clinical sites including the ability to obtain written informed consents and record data correctly. The monitor will inform the DCC PI, the Steering Committee, and NICHD regarding problems relating to facilities, technical equipment, or medical staff. A thorough written report will follow each site-visit and will include a detailed itemization of discrepancies and items requiring follow-up or reconciliation. This report will also be forwarded to the NICHD Research Scientist for review. The monitor will be responsible for maintaining regular contacts between the investigators in the clinical sites and the RMN. When the study ends, the monitor will also visit the clinical site to provide assistance for close-out.

10.7 Data and Safety Monitoring Board

The NICHD has established a DSMB to review and interpret data generated from RMN studies and to review protocols prior to their implementation. Its primary objectives are to ensure the safety of study subjects, the integrity of the research data and to provide NICHD with advice on the ethical and safe progression of studies conducted in the RMN. The DSMB advises on research design issues, data quality and analysis, and research participant protections for each prospective and on-going study. A copy of the DSMB Charter can be found in the appendix.

The DSMB members are appointed by the Director of NICHD in accordance with established NIH and NICHD policies. DSMB members are experts in and represent the following fields: biostatistics, epidemiology, infertility, gynecology, andrology and ethics. The NICHD Committee Coordinator is responsible for scheduling regular committee meetings, recording all meeting minutes and summarizing the committee recommendations for the Steering Committee and NICHD. Steering Committee members are prohibited from attending closed sessions of the DSMB. Open sessions may be attended by Steering Committee members or Chairperson when requested by NICHD and the DSMB.

The DSMB meets regularly at a time and place of their choosing to review Network randomized trial protocols with respect to ethical and safety standards, monitors the safety of on-going clinical trials, monitors the integrity of the data with respect to original study design, and provides advice on study conduct. The DSMB periodically monitors data quality, including protocol adherence and adverse events. As outlined in the protocols, the DSMB will conduct interim evaluations of the data. It may recommend protocol modifications based on concern for subject welfare and scientific integrity.

10.8 Reporting

Administrative Reports will be prepared by the DCC, and they include monthly and quarterly reports to the RMN on accrual, data quality and study compliance and reports presented in the packet produced for each Steering Committee and DSMB. Statistical reports include reports to the SC and AB from the data analysis, and special reports for scientific manuscripts.

Statistical Reports will be generated in SAS. Reports are provided for DSMB reviews, and for final analysis of study results in preparation for scientific publications. The content of the interim reports will be very complete, and will serve as the template for the final report of each study, which in turn will form the basis of the publication of the results. Our proposed reports to the DSMB would include the following: a protocol description and history; accrual rates; site performance in terms of accrual; eligibility; protocol violations; data accuracy and minority representation; patient characteristics by treatment and site; and the rate of adverse experiences.

10.9 Data Analysis

Primary analyses will use an intent-to-treat paradigm, wherein all randomized subjects are included according to their randomized treatment assignment, regardless of actual treatment received, protocol violations, etc. for the primary outcome. The data will be summarized using descriptive statistics for continuous variables (mean, standard deviation, number of

observations, and percentiles) and frequency statistics (frequencies and percentages) for categorical variables. All hypothesis tests will be two-sided and all analyses will be performed using SAS software, (SAS Institute, Inc., Cary, NC) or R (open source).

Primary efficacy analysis will be done using a logistic regression model to compare the two treatment arms with respect to the primary outcome of a good birth outcome while adjusting for recruitment site and baseline BMI. The proportion of good birth outcomes in each treatment arm will be reported, as well as the odds ratio (OR) and corresponding 95% confidence interval (CI). We propose a number of secondary analyses and acknowledge that the power of our study may be low for some of these outcomes. However, even if not significant, examination of these secondary endpoints will guide future research in the area of infertility treatment. Logistic regression models will be fit to binary outcomes, such as pregnancy status (yes/no), to assess differences in the treatment arms while adjusting for recruitment site and baseline BMI. For rare binary events, e.g., multiple pregnancy births or other adverse events, Firth's penalized likelihood approach to logistic regression will be used to address the issue of having a small number of events.[90] Effect sizes from the logistic regression models will be quantified using the OR and 95% CI.

The analysis of other secondary outcomes will entail the application of statistical methods that have been developed for correlated data since repeated observations will be made over time on each individual, and these methods allow for both within-group and between-group comparisons to be assessed. For secondary outcomes such as weight loss or ultrasound parameters, a linear mixed-effects model will be fit where the main independent variables will be treatment arm, cycle number, and their interaction, as well as the randomization stratification factors of recruitment site and baseline BMI as covariates.[91] The linear mixed-effects model is an extension of linear regression that accounts for the within-subject variability inherent in longitudinal trials and has the flexibility to adjust for potentially confounding covariates if deemed necessary. Potential additional covariates in the models may include prior exposure to study medication, the baseline value of the outcome, and age. The effect size will be quantified from the mixed-effects models using the difference in the means between the treatment arms and corresponding 95% CI. For binary outcomes measured over time, e.g., ovulation rates, generalized estimating equations with a logit link, an extension of logistic regression that accounts for correlated data, will be fit where the main independent variables will be treatment arm, cycle number, and their interaction, as well as the randomization stratification factors of recruitment center and baseline BMI as covariates.[92] Cox proportional hazards models and the Kaplan-Meier method will be applied to compare time to pregnancy and time to live birth in the treatment groups.

<u>Participant Drop-out and Other Missing Data</u>: While every attempt will be made to collect complete data and prevent drop-out, missing data are inevitable. The sample size has been inflated by 20% in anticipation of some drop-out such as participant retention issues (based on experience in the PPCOS I, PPCOS II, OWL PCOS, and AMIGOS studies). The mechanism for missing data (missing completely at random, missing at random, or missing not at random) will be examined prior to beginning the analyses. We will compare the available characteristics of those with missing data to those with complete data and attempt to predict missingness with available

data using multiple logistic regression and calculating the area under the receiver operating characteristic curve.[93] If necessary, imputation techniques may be used,[94] and shared parameter models or random pattern-mixture models considered if the preliminary analyses suggest that the data are missing not at random so that individuals with missing data can be included in the analyses.

Statistical versus Clinical Significance and Multiple Testing*:* As our analyses are based upon defined hypotheses, we have not included corrections for multiple testing. While the issue of p-value adjustment for multiple testing has long been a topic of discussion in observational epidemiology and clinical trials, it generally is accepted that p-values should not be the sole criterion for assessing relationships. Conclusions will be based on the preponderance of scientific evidence related to each hypothesis, considering point estimates and confidence intervals, as well as statistical significance. Nonetheless, findings with marginal statistical significance will be interpreted cautiously, taking into consideration whether multiple testing could have contributed to a type I error.

11 Data Collection, Site Monitoring and Adverse Experience Reporting

11.1 Records to be kept

Data will be collected prospectively by designated research personnel at each study site, supervised by the site PI. Subject data will be entered into a web-based data management system created by the Data Coordination Center, using only a study ID number. Original source documents will be kept in the study subject folder.

11.1.1 Maintenance/Retention of site records

In order to comply with Good Clinical Practice (GCP) requirements, the investigators must maintain the master patient log that identifies all patients entered into the study for a period of two years after the study ends so that the subjects can be identified by audit. The PI must maintain adequate records pertaining to subjects' files and other source data for a minimum of 5 years after completion of the study.

11.2 Role of Data Management

Each clinical site and the DCC will be responsible for ensuring study personnel are trained and follow the data management guidelines of Good Clinical Practice and RMN policies.

11.3 Quality Assurance

The DCC will perform regular clinical site monitoring to assure protocol compliance, ethical standards, regulatory compliance and dat quality at the clinical sites, including review or records available for inspection by monitoring authorities. These data will be shared with the Steering committee and the DSMB as needed. Regular face to face meetings, monthly conference calls, and phone conferences of the protocol committees and recruitment committees will be forums for addressing quality assurance issues.

11.3.1 Data Entry and Forms

Case Report Forms (CRFs) will be developed as the protocol is developed. They will also be implemented in a Web-based Oracle data management system. The Web data entry forms will be similar to the paper forms with the same questions. However, the Web forms usually have more flexibility than the paper forms, such as pull-down menus.

11.3.2 Features of Data Management System

Features of the data management system include study definition; different types of data entry (and complete audit trail); forms control; query capture, reporting, and resolution; dictionary coding of Adverse Events (AEs) and medical terms; and clinical data review Tools. The end-user/reporting/ad hoc query front-end uses a standard Web browser, so that data entry and browsing can be done from any machine with Internet access, without purchase of special software. Login to this system will be through a secured Web server with the security under the protection of Yale Center for Clinical Investigations.

11.3.3 Data Security

A data server and Web server will be used. The data server will be managed by YNHH IT center and the servers will be managed by Forte Research Systems. The web server will be accessible through a secured login, but the data server can only be accessed through the web server. For security purposes, no login to the data server will be permitted, and access to the back end is limited to authorized individuals. PHI, including patient names and addresses, will be locked and secured at the participating sites, and data will be linked through a unique identification number, which will be assigned after a patient is screened or enrolled. Access will be limited to authorized individuals (21 CFR 11.10(d)). Each user of the system will have an individual account. The user will log into the account at the beginning of a data entry session, input information (include changes) on the electronic record, and log out at the completion of the data entry session. The system will be designed to limit the number of log-in attempts and record unauthorized access log-in attempts. Individuals will work only under their own access key, and not share these with others. The system will not allow an individual to log onto the system to provide another person access to the system. Users will be asked to change their passwords at established intervals commensurate with a documented risk assessment. This plan has been adapted from the guidelines for computerized systems used in clinical investigations established by the U.S Department of Health and Human Services Food and Drug Administration.

11.3.4 Data Quality Control

11.3.4.1 Competency to perform procedure/tests in the protocol

The site PI will be responsible for ensuring that study related tests are performed by competent personnel. The criteria for determination of competency may vary between sites in the study. Attempts will be made to standardize protocols whenever possible to minimize inter-site variation.

11.3.4.2 Quality Control Steps

Quality control of data will be handled on three different levels. The first level is the real-time logical and range checking built into the web-based data entry system. The research coordinators and data entry clerks at the participating sites are required to ensure the data accuracy as the first defense. The second is the remote data monitoring and validation that is the primary responsibility of the data manager and programmer at the DCC. The data manager will conduct monthly comprehensive data checks (SAS programs run on a regular basis as a systematic search for common errors and omissions), as well as regular manual checks (within the database system). Manual checks will identify more complicated and less common errors. The data manager will query sites until each irregularity is resolved. The third level of quality control will be the site visits, where data in our database will be compared against source documents. Identified errors will be resolved between our center and clinical sites. The visits will assure data quality and patient protection.

An audit trail will be added as another security measure. This will ensure that only authorized additions, deletions, or alterations of information in the electronic record have occurred and allows a means to reconstruct significant details about study conduct and source data collection

necessary to verify the quality and integrity of data. Computer generated, time-stamped audit trails will be implemented for tracking changes to electronic source documentation.
Controls will be established to ensure that the system's date and time are correct. This is a multi-center clinical trial and will be located in different time zones. System documentation will explain time zone references as well as zone acronyms. Dates and times will include the year, month, day, hour, and minute to the date provided by international standard-setting agencies (e.g. US National Institute of Standards and Technology). The ability to change the date or time will be limited to authorized personnel, and such personnel will be notified if a system date or time discrepancy is detected.
In addition to internal safeguards built into the computerized system, external safeguards will be implemented. Data will be stored at the Data Coordination Center. Records will be regularly backed up, and record logs maintained to prevent a catastrophic loss and ensure the quality and integrity of the data. This plan has been adapted from the guidelines for computerized systems used in clinical investigations established by the U.S Department of Health and Human Services Food and Drug Administration.

11.3.5 Obligation of the Investigator

11.3.5.1 IRB Review

The site PI is responsible for submitting the approved protocol and consent form to the local IRB for review. The IRB must approve all aspects of the study as detailed in the protocol, including the patient informed consent form. It is anticipated that there will be minor site-specific changes in the consent form. The IRB must periodically review the status of the study at appropriate intervals not exceeding one year. The site PI will also be responsible for submitting revisions to the protocol to the IRB, as directed by the DCC, and promptly communicating serious adverse events that result during the study, to both the local IRB and the DCC. After the approval, the informed consent and IRB approval (or amendment) letters must be forwarded to the DCC.

11.3.6 Establishment of Pregnancy Registry

We intend to establish a pregnancy registry for this trial to establish outcomes of pregnancy. Couples who conceive will be offered participation in the Pregnancy Registry and a separate informed consent form will be obtained if they choose to participate. We will track the outcomes of all randomized subjects who have a positive serum pregnancy screen during the course of this study. We will record biochemical pregnancies (defined as positive serum pregnancy screens without ultrasonically detected pregnancies), ectopic pregnancies, and all intrauterine pregnancy losses both before and after 20 weeks including missed abortions, spontaneous abortions, elective abortions, fetal demises, and stillbirths. We will review pregnancy and birth records of the mother and of the fetus to establish neonatal morbidity and mortality and the presence of fetal anomalies. This will be done for all subjects who conceive.

If they participate in the Pregnancy Registry, we will extract from these records concomitant medical and obstetrical conditions, exposure information on all other medical products used, including prescription products, over-the-counter (OTC) products, dietary supplements, vaccines, and insertable or implantable medical devices. We will file individual case reports for all

congenital anomalies which will be considered a serious adverse event. Additionally we will provide to the FDA a written annual status report of the pregnancy registry as specified in the guidelines above. We intend at a minimum to perform an annual parent directed screening questionnaire to assess the infant's developmental milestones for the first three years after birth as recommended by the FDA and to review the child's CDC growth curve and medical records. The registry will follow children until age 3. A survey will be administered to the mother annually that will screen for cognitive, and neurodevelopment up to age 3. If medical issues arise from the survey, a medical record review will be performed for more extensive assessment.

11.3.7 Regulatory Requirements

The DCC will work with NICHD staff by providing them with clinical study data, reports, and other support as required for AE Reporting. The Project Managers will work with NICHD colleagues in meeting all regulatory requirements including compliance with ICH and HIPAA requirements, FDA code for federal regulations (Title 21). For example, the DCC Project Managers have registered this clinical trial with ClinicalTrials.gov via a web based data entry system called the Protocol Registration System (PRS) (NCT02432209).

11.3.8 Protocol Amendments

Once the protocol is approved by the Steering Committee, it is then reviewed by an Advisory Board and Data and Safety Monitoring Board (DSMB). After all approvals, the DCC will finalize the protocol document that serves as the agreement among all members of the Network. In the meantime, because the DCC administers all patient care costs for the RMN, the DCC will promptly issue subcontracts to the participating sites based on the cost agreements made by the Steering Committee and NICHD.

After the protocols are approved by the RMN and the Steering Committee decides that changes are necessary for scientific or clinical reasons, the DCC will facilitate the procedure in a timely and diligent fashion. The RMN investigators and key personnel will participate in teleconferences and meetings, discuss, vote, and document circumstance and rationales for the changes and the implementation procedure for the changes. These include revising study hypotheses, designs, sample sizes, data entry forms, and appropriate statistical analyses. Once the amendments are finalized and agreed to by the RMN, they will be submitted for IRB and DSMB reviews and approvals.

11.4 Adverse Event Reporting

11.4.1 Serious Adverse Events

All serious adverse events (SAEs) that occur from randomization at Phase I through thirty days after the last dose of study medication (orlistat, clomiphene, or hCG) must be reported or if the patient is pregnant, 6 weeks following delivery. A serious adverse event is defined as: fatal or immediately life-threatening; severely or permanently disabling; requiring or prolonging inpatient hospitalization; overdose (intentional or accidental); congenital anomaly; pregnancy

loss after 20 weeks gestation; neonatal death up to 6 weeks after delivery; or, any event adversely affecting the study's risk/benefit ratio. Additionally, any event that, based on appropriate medical judgment, may jeopardize the subject's health and may require medical or surgical intervention to prevent one of the outcomes listed above is considered an SAE.

If an SAE occurs and is thought to be related to the study medication, the study medication will be discontinued.

The site PI will report the SAE by completing and signing the Serious Adverse Event Report Form [available in the "Study Forms" section of the RMN members-only website], and then emailing the document in PDF format to rmn.dcc@mailman.yale.edu. Subjects will be identified by study number only. No other identifying information will be included on the form. The site PI must determine and record on the SAE form whether the SAE is unanticipated or anticipated, and if it is related, possibly related, or unrelated to participation in the research.

DCC staff will enter the SAE information in the central database. The Safety Surveillance team, consisting of the DCC, NICHD research scientist and lead PI of the protocol, will analyze the SAE to determine if it meets the criteria listed in the OHRP 45CFR46 and/or FDA 21CFR312.32 & 3.14.80.

These determinations will dictate timeframes for sites' submission to the DCC, and the DCC's submission to the DSMB (**Table 12**):

Table 12: Types of Serious Adverse Events and their reporting requirements

TYPE	SITE	DCC
Unanticipated and related/possibly related SAE, fatal or life-threatening	Report to DCC within 1 business day of discovery	Notify DSMB by end of next business day of receiving site report
Other unanticipated and related/possibly related SAE	Report to DCC within 1 business day of discovery	Notify DSMB 5 business days of receiving site report
Anticipated and related/possibly related SAE	Report to DCC within 5 business days of discovery	Notify DSMB 5 business days of receiving site report
Unrelated SAE (anticipated or unanticipated	Report to DCC within 10 business days (no more than 3 weeks) of discovery	Notify DSMB within 10 business days (no more than 3 weeks) of receiving site report

Upon receiving notification of an SAE, the DSMB will review it via a closed-session email or conference-call discussion arranged by the NICHD RMN Committee Coordinator [RMN CC] (**Figure 5**). The DSMB will send a report to the RMN CC within two weeks; reports for life-threatening SAEs will be submitted in one week. The DSMB report will include: statement indicating what related information the DSMB reviewed; the review date; the DSMB's assessment of the information reviewed; and the DSMB's recommendation, if any, for the DCC.

Figure 6: Serious Adverse Event Flow Chart

The RMN CC will then forward the DSMB report to the DCC for the record and appropriate disbursal. The DCC will forward reportable events to all RMN investigators, NIHCD, and the FDA on behalf of the NIHCD if the protocol is under IND. The NIHCD Project Scientist will review, sign, and return the IND safety report to the DCC within 2 business days, and will follow up with the site PI and DCC on the SAE until it is resolved. The Protocol PI will evaluate the frequency and severity of the SAEs and determine if modifications to the protocol and consent form are required. Site PIs will report the SAE to their site IRB according to local IRB requirements. For more information, please see the RMN/DSMB Communication Procedure.

Adverse events deemed non-serious will also be recorded throughout study participation from the start of study drug through one week after the last dose of study medication, and reported to the DCC. If an anticipated serious adverse event occurs at a frequency greater than expected, the DCC will notify the DSMB by the end of the next business day of discovery and follow the procedures for reporting serious and unanticipated and related adverse events. The DCC will forward relevant safety information to the DSMB. If an adverse event not initially determined to be reportable to the FDA under 21CFR312.32 is so reportable, the DCC will report the adverse event to the FDA within 15 calendar days after the determination is made.

Figure 5: Serious Adverse Event Flow Chart

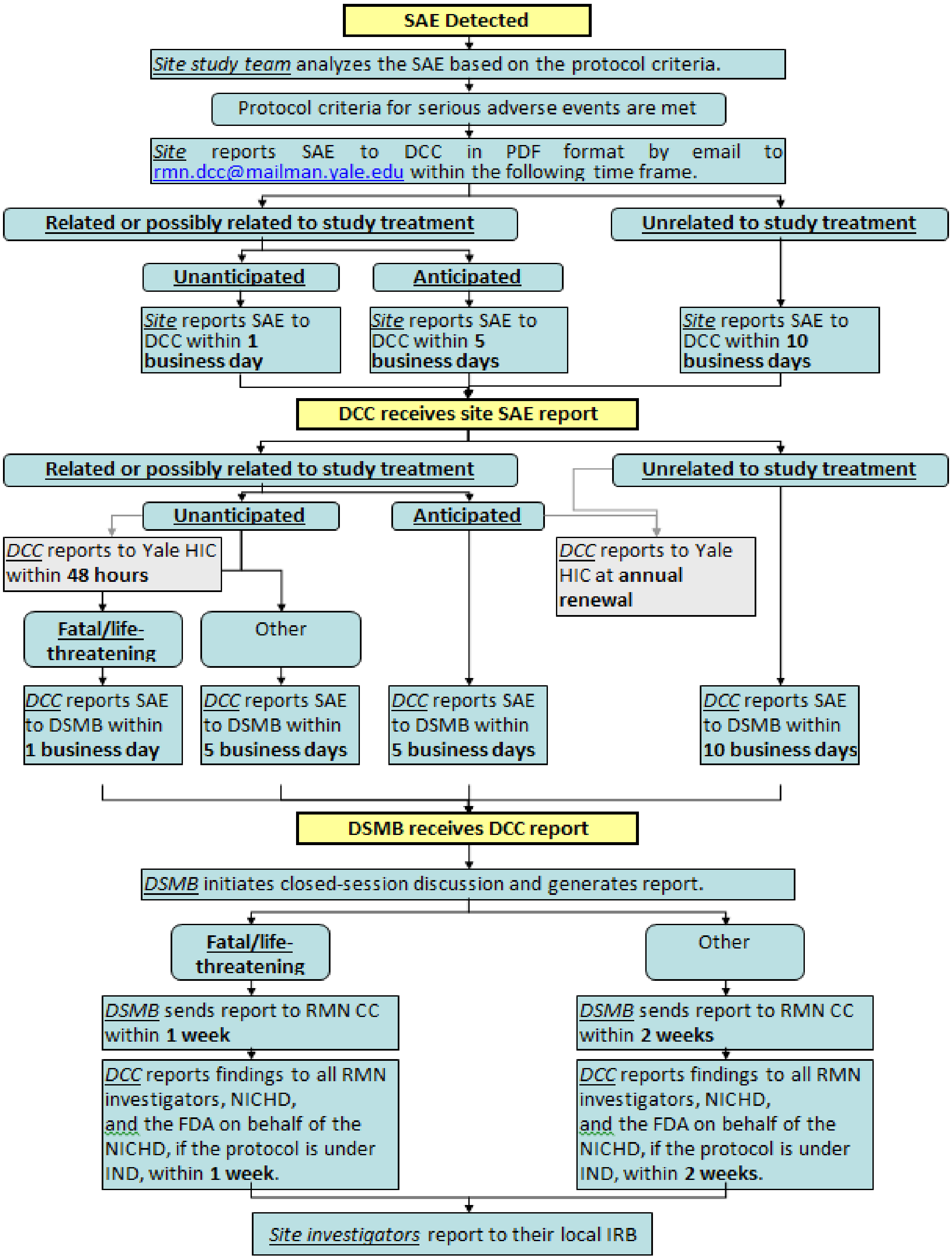

CFR= Code of Federal Regulations

12 Human Subjects

12.1 Institutional Review Board (IRB) Review and Informed Consent

This protocol and the informed consent document (see Appendix) and any subsequent modifications will be reviewed and approved by the IRB responsible for oversight of the study. A signed consent form will be obtained from the subject. The consent form will describe the purpose of the study, the procedures to be followed, and the risks and benefits of participation. A copy of the consent form will be given to the subject, parent, or legal guardian, and this fact will be documented in the subject's record.

12.2 Subject Confidentiality

All laboratory specimens, evaluation forms, reports, video recordings, and other records that leave the site will be identified only by the Study Identification Number (SID) to maintain subject confidentiality. All records will be kept in a locked file cabinet. All computer entry and networking programs will be done using SIDs only. Clinical information will not be released without written permission of the subject, except as necessary for monitoring by IRB, the NINDS, the OHRP, the sponsor, or the sponsor's designee.

12.3 Study Modification/Discontinuation

The study may be modified or discontinued at any time by the IRB, the NICHD, the OHRP, or other government agencies as part of their duties to ensure that research subjects are protected.

13 Publication of Research Findings

13.1 Overall Policy

The publications policy proposes guidelines for publications that originate from our collaborative Reproductive Medicine Network. Decisions concerning publications shall be determined by consensus (majority vote) of the collaborating principal investigators (or designate) noted below as the "Network". This policy is designed to promote prompt, exact, quality publications and presentations of Network studies with appropriate academic recognition of those with significant contributions. Protocols are classified into three types: 'Main Study' (which may include major and minor publications), 'Ancillary Study', and 'Pilot Study'. Additionally there may be publications from concepts or ideas generated by the RMN ("Related Publications") or from other groups utilizing RMN data and/or specimens "Outside Studies" (those utilizing data and/or specimens from the RMN studies). Abstract submissions to national meetings will also follow the publications policy below. The progress of publications (including presentations) will be a standing agenda note on all phone conferences and meetings. The Steering Committee will make the final disposition regarding disputes with respect to analysis request approval, prioritization, presentation, authorship and/or manuscript submission.

13.2 Main Study

A Main Study is a Network study designed prospectively by an investigator independent of other studies. Generally that investigator becomes Lead investigator of the protocol and Chair of the Protocol Subcommittee. At the end of each Main Study, a primary analysis resulting in the primary manuscript and a number of secondary analyses is produced based on the research questions stated in the protocol. The Protocol Subcommittee Chair is the primary author of the primary analysis. A main study can generate major (related to the major hypotheses) and minor publications (relating to secondary hypotheses).

13.2.1 Major Publications (Tables 13-14)

A major publication is defined as one reporting results of the major hypotheses tested. (For example, does hMG/IUI increase cyclic fecundity in couples with unexplained or male factor infertility?).

1. Authorship: Publications will include the names of investigators from each RMU and the DCC rather than merely identify the "Reproductive Medicine Network". Each RMU and DCC will have up to two authors per publication, ordinarily the PI and the Co-PI, but this may at times involve another investigator who has contributed to the study at their site, in lieu of the PI or Co-PI. The principal investigator at each RMU will be responsible for submitting the names of the two authors from that unit and designating them as either the primary and secondary authors of the unit. No more than 2 authors may represent a RMN site. An ancillary site (such as a SCCPIR) may only have 1 investigator. The Steering Committee Chair and NIH Project Scientist will be authors. Occasionally, additional authors, both within and outside the RMN may be appropriate. In these cases, the final decision will be by Network consensus (majority vote of the steering committee required).

2. First Author: The lead investigator initiating the protocol, chairing the Protocol Subcommittee will be the first author. The first author would always be expected to prepare the initial draft of the manuscript, after receiving approval from the Network to proceed. The author will prepare the first draft of the manuscript in a timely fashion after receiving all the relevant data analyses from the DCC. The primary author will circulate the final draft to all authors prior to submission, with a timely turnaround of comments from other authors expected. Final decision of the manuscript content will be determined by the Protocol Subcommittee. In the event that the initiating protocol investigator declines first authorship or fails to meet the timeline determined by the Steering Committee (as determined by majority vote) and monitored monthly, the next RMU investigator in the rank order of authors (described below) will be the first author.

3. Authorship Order: All authorships are expected to meet reasonable criteria as set forth by the International Committee of Medical Journal Editors. Uniform requirements for manuscripts submitted to biomedical journals. http://www.icmje.org. Updated February 2006. Accessed April 4, 2007. The overall authorship order will be 1) the primary author, 2) RMU investigators, additional outside investigators with a limit of one author per site (e.g. SCCPIR investigators if applicable), followed by the Steering Committee Chair, NIH Project Scientist, and then the authors of the DCC.

Table 13: RMN Publication Authorship

Authorship Order Category	Description
1	Lead Investigator of the Protocol (N =1)
2	Primary RMN Investigators of the Protocol (N = 6); DCC investigator (N = 1)
3	Outside Investigators (i.e. Primary Investigator of SCCPIR sites) (N to be determined)
4	Additional Investigators (by Steering Committee vote) (N to be determined)
5	Secondary RMN Investigators (N = 7)
6	Steering Committee Chair (N = 1)
7	NIH Project Scientist (N = 1)
8	DCC PI (N = 1)
9	"for the NICHD Reproductive Medicine Network"

It is anticipated there will be up to 18-25 authors per major manuscript. The authorship order of the RMUs and outside sites will be based upon subject recruitment, data accuracy and promptness of data report according to the chart below:

Table 14: Authorship Order of the RMUs and Outside Sites

Investigative Sites	# Subjects Rank	Accuracy Rank	Total Rank	Authorship Order
A	1	4	5	3
B	2	7	9	6
C	3	1	4	2
D	4	2	6	4
E	5	3	8	5
F	6	5	11	7

Data accuracy will be ranked according to the rate of missing or false data entries/randomized subject at each site. Inquires that show data was accurately entered will not count against this rate of data inaccuracy. Each site's PI will be responsible to document the contributions to the study of that site's authors. In the event the journal editor requires fewer authors even after written documentation of the authors' contribution has been provided, the steering committee will vote on the authorship order which will include at a minimum the Lead Investigator and PI of the DCC (or his/her designate) in the positions listed above with the authorship order ending with the footnoted statement "for the Reproductive Medicine Network". The other authors will be referenced in the footnote and listed in the title page.

4. Acknowledgement Section: The acknowledgement section will include other investigators and study personnel who contributed substantially to the study by site, as well as members of the Advisory Board and the Data and Safety Monitoring Board. The designation will list the initials of the individual followed by their highest degree (e.g. C. L. Gnatuk, J.A. Ober, R.N., etc.) Significant contributions include but are not limited to protocol review, initiation and participation at each site, subject recruitment and enrollment, study conduct, data analysis, and preparation of the manuscript.

13.2.2 Minor Publications

Minor studies are defined as those in which the hypotheses would not be the main elements of Network studies, but in which the study data base would be utilized to test secondary hypotheses. (One example would be testing whether metformin use spares the dose of clomiphene resulting in lower dose needs.) Ideas for "minor studies" will, in general, be proposed by a single individual, who would direct all efforts leading to publication and representation. The results from minor studies would be handled similarly to those from major studies. The "Protocol" is defined as the Concept Protocol/study design of the hypothesis resulting in the publication.

Authorship will follow the Major publications guidelines above with the exception that the individual leading the minor study would be the first author, followed by the ranked primary RMN investigators involved in developing the Concept Protocol. The Lead Investigator of the minor

publication can propose additional investigators who contributed to the study, whose inclusion in the authorship will be voted on by the Steering Committee (majority vote of SC required for inclusion in authorship. Centers may wish to withdraw inclusion from authorship of publications of minor studies in which only data are contributed, and this will be the decision of the individual site (RMU) PI.

13.2.3 Ancillary Study

An Ancillary Study is an observational study, conducted as a supplement to a Main Study. By definition, an Ancillary Study involves all or a subset of patients enrolled in a Main Study. An Ancillary Study does not involve any additional participants. To be defined as an Ancillary Study, there must be a need for collection of additional data not already collected in the Main Study. An Ancillary Study may also be designed by another Network investigator, who would serve as the lead investigator and primary author of the paper. Ancillary Studies may be a "single-center" or "multi-center".

A "single-center" Ancillary Study is a study in which all data are collected, stored and analyzed at a single center. The center bears the additional cost of such a study. The study requires approval of the Main Study Protocol Subcommittee and the Steering Committee. The center conducting the study is responsible for the analysis and reporting of the results. Abstracts and manuscripts resulting from data from the single-center Ancillary Study are not subject to the RMN Publications Policies.

A "multi-center" Ancillary Study is defined as one for which data or material (such as specimens) are collected at more than one center, or additional funds for conduct of the study are provided by the NICHD RMN and the DCC provides data analysis. Multi-center Ancillary Studies require the approval of the Main Study Protocol Subcommittee and the Steering Committee.

Authorship will be as per Major publications above with the exception that the individual leading the ancillary study and writing the paper would be the first author, followed by ranked RMN primary investigators, etc. A center not participating in the ancillary study would not receive authorship unless by majority vote of the steering committee.

13.2.4 Pilot Study

A Pilot Study is a preliminary study that generates data to help in the design of a Main Study and is the responsibility of the Main Study Protocol Subcommittee. The DCC collaborates with the Protocol Committee to complete the analysis, which may or may not generate an abstract for presentation and/or a manuscript for publication. The DCC writes a Final Report if there is sufficient data to justify one. It is not expected there will be any secondary analyses resulting from a Pilot Study.

13.2.5 Related Publications

A related publication is one that has had significant input from members of the RMN Steering Committee at formal meetings in terms of study significance and design. It is distinct from an ancillary publication in that a related publication reports on a study, concept or new methodology that has not been subjected to formal DSMB review and approval. Generally, "Related Publications" will arise from ideas and studies discussed with the Steering Committee, but not voted upon to become formal protocols.

The investigator who initiates, conducts and writes the study and those who (s)he names will be the sole authors. The authors should acknowledge the contribution of the NICHD Reproductive Medicine Network in the author line of the publication according to the format of the journal.

13.2.6 Outside Studies

Outside studies will result from the sharing of data and/or specimens with investigators whose protocols have been approved by the steering committee, and who comply with all components of those policies. All publications will acknowledge the assistance of NICHD, the RMN, and the Protocol Subcommittee in making the database available on behalf of the project. In addition, however, a disclaimer will need to be included stating, "the contents of this report represent the views of the authors and do not represent the views of the NICHD Reproductive Medicine Network." The authors will be requested to cc the submitted manuscript to the NICHD program official to ensure compliance. These policies apply to both Network centers and outside centers.

13.2.7 Presentations

Network data should be presented before national organizations by the lead investigators of Main Studies, Ancillary, and Pilot studies. Organizations that might be appropriate include the American Society of Reproductive Medicine, the Society for Gynecologic Investigation, the American College of Obstetricians and Gynecologists, the American Urology Society and other urology or andrology societies. All presentations will be approved by the P & P committee. Once data are published in at least abstract form, all members of the Network can cite them publicly in lectures.

However, investigators should avoid citing specific numbers in review articles and chapters, for this could jeopardize peer review publication. Authorship, First Author, and Author Order are as described for Major Publications, and if there is an authorship limit to the abstract we will follow the plan above under Major Publications. Oral and poster presentations, including those resulting from secondary analyses at professional societies, must list all authors and participating institutions. In addition, they must include both the NICHD RMN logo and NIH Department of Health and Human Services logos that can be found on the Network web site.

14 References

1. Sim KA, Partridge SR, Sainsbury A. Does weight loss in overweight or obese women improve fertility treatment outcomes? A systematic review. Obesity reviews : an official journal of the International Association for the Study of Obesity 2014;15:839-50.
2. Bolumar F, Olsen J, Rebagliato M, Saez-Lloret I, Bisanti L. Body mass index and delayed conception: a european multicenter study on infertility and subfecundity. American journal of epidemiology 2000 Jun 1;151:1072-9.
3. Pasquali R, Pelusi C, Genghini S, Cacciari M, Gambineri A. Obesity and reproductive disorders in women. Human reproduction update 2003;9:359-72.
4. Imani B, Eijkemans MJ, te Velde ER, Habbema JD, Fauser BC. A nomogram to predict the probability of live birth after clomiphene citrate induction of ovulation in normogonadotropic oligoamenorrheic infertility. Fertility & Sterility 2002 January;77:91-7.
5. Imani B, Eijkemans MJ, Faessen GH, Bouchard P, Giudice LC, Fauser BC. Prediction of the individual follicle-stimulating hormone threshold for gonadotropin induction of ovulation in normogonadotropic anovulatory infertility: an approach to increase safety and efficiency. Fertility & Sterility 2002 January;77:83-90.
6. Rausch ME, Legro RS, Barnhart HX, et al. Predictors of pregnancy in women with polycystic ovary syndrome. The Journal of clinical endocrinology and metabolism 2009;94:3458-66.
7. Luke B, Brown MB, Stern JE, Missmer SA, Fujimoto VY, Leach R. Female obesity adversely affects assisted reproductive technology (ART) pregnancy and live birth rates. Human Reproduction 2011;26:245-52.
8. Winter E, Wang J, Davies MJ, Norman R. Early pregnancy loss following assisted reproductive technology treatment. Hum Reprod 2002;17:3220-3.
9. Metwally M, Ong KJ, Ledger WL, Li TC. Does high body mass index increase the risk of miscarriage after spontaneous and assisted conception? A meta-analysis of the evidence. Fertility and sterility 2008;90:714-26.
10. Wang JX, Davies MJ, Norman RJ. Obesity increases the risk of spontaneous abortion during infertility treatment. Obesity research 2002;10:551-4.
11. Garbaciak JA, Jr., Richter M, Miller S, Barton JJ. Maternal weight and pregnancy complications. American journal of obstetrics and gynecology 1985;152:238-45.
12. Guelinckx I, Devlieger R, Beckers K, Vansant G. Maternal obesity: pregnancy complications, gestational weight gain and nutrition. Obesity reviews : an official journal of the International Association for the Study of Obesity 2008;9:140-50.
13. Sarwer DB, Allison KC, Gibbons LM, Markowitz JT, Nelson DB. Pregnancy and obesity: a review and agenda for future research. J Womens Health (Larchmt) 2006;15:720-33.
14. Consensus on infertility treatment related to polycystic ovary syndrome. Fertility and sterility 2008;89:505-22.
15. Consensus on infertility treatment related to polycystic ovary syndrome. Hum Reprod 2008;23:462-77.
16. Balen AH, Anderson RA, Policy, Practice Committee of the BFS. Impact of obesity on female reproductive health: British Fertility Society, Policy and Practice Guidelines. Hum Fertil (Camb) 2007;10:195-206.

17. Johnson NP, Stewart AW, Falkiner J, et al. PCOSMIC: a multi-centre randomized trial in women with PolyCystic Ovary Syndrome evaluating Metformin for Infertility with Clomiphene. Hum Reprod 2010;25:1675-83.
18. Farquhar CM, Gillett WR. Prioritising for fertility treatments--should a high BMI exclude treatment? BJOG : an international journal of obstetrics and gynaecology 2006;113:1107-9.
19. Anderson K, Norman RJ, Middleton P. Preconception lifestyle advice for people with subfertility. Cochrane Database of Systematic Reviews 2010:CD008189.
20. Hull MG, Glazener CM, Kelly NJ, et al. Population study of causes, treatment, and outcome of infertility. Br Med J (Clin Res Ed) 1985;291:1693-7.
21. Frisch RE, Revelle R. Height and weight at menarche and a hypothesis of critical body weights and adolescent events. . Science 1970 169:397-9.
22. Legro RS, Lin HM, Demers LM, Lloyd T. Rapid maturation of the reproductive axis during perimenarche independent of body composition. The Journal of clinical endocrinology and metabolism 2000;85:1021-5.
23. Wang Y. Is obesity associated with early sexual maturation? A comparison of the association in American boys versus girls. Pediatrics 2002;110:903-10.
24. Chen ZJ, Zhao H, He L, et al. Genome-wide association study identifies susceptibility loci for polycystic ovary syndrome on chromosome 2p16.3, 2p21 and 9q33.3. Nature genetics 2011;43:55-9.
25. Rochester D, Jain A, Polotsky AJ, et al. Partial recovery of luteal function after bariatric surgery in obese women. Fertility and sterility 2009;92:1410-5.
26. Legro RS, Dodson WC, Gnatuk CL, et al. Effects of gastric bypass surgery on female reproductive function. The Journal of clinical endocrinology and metabolism 2012;97:4540-8.
27. Sarwer DB, Spitzer JC, Wadden TA, et al. Changes in sexual functioning and sex hormone levels in women following bariatric surgery. JAMA surgery 2014;149:26-33.
28. Tsagareli V, Noakes M, Norman RJ. Effect of a very-low-calorie diet on in vitro fertilization outcomes. Fertility and sterility 2006;86:227-9.
29. Morris SN, Missmer SA, Cramer DW, Powers RD, McShane PM, Hornstein MD. Effects of lifetime exercise on the outcome of in vitro fertilization. Obstetrics and gynecology 2006;108:938-45.
30. Gudmundsdottir SL, Flanders WD, Augestad LB. Physical activity and fertility in women: the North-Trondelag Health Study. Hum Reprod 2009;24:3196-204.
31. De Souza MJ, Toombs RJ, Scheid JL, O'Donnell E, West SL, Williams NI. High prevalence of subtle and severe menstrual disturbances in exercising women: confirmation using daily hormone measures. Hum Reprod 2010;25:491-503.
32. Loucks AB, Verdun M, Heath EM. Low energy availability, not stress of exercise, alters LH pulsatility in exercising women. Journal of applied physiology 1998;84:37-46.
33. De Souza MJ, Lee DK, VanHeest JL, Scheid JL, West SL, Williams NI. Severity of energy-related menstrual disturbances increases in proportion to indices of energy conservation in exercising women. Fertility and sterility 2007;88:971-5.
34. Anonymous. Clinical guidelines on the identification, evaluation, and treatment of overweight and obesity in adults--the evidence report. National institutes of health. [See comments.] [Erratum appears in obes res 1998 nov;6(6):464.]. [Review] [769 refs]. Obesity Research 6 Suppl 2:51S-209S, 1998 Sep.

35. Heshka S, Anderson JW, Atkinson RL, et al. Weight loss with self-help compared with a structured commercial program: a randomized trial. JAMA : the journal of the American Medical Association 2003;289:1792-8.
36. Tate DF, Wing RR, Winett RA. Using Internet technology to deliver a behavioral weight loss program. JAMA : the journal of the American Medical Association 2001;285:1172-7.
37. Tate DF, Jackvony EH, Wing RR. Effects of Internet behavioral counseling on weight loss in adults at risk for type 2 diabetes: a randomized trial. JAMA : the journal of the American Medical Association 2003;289:1833-6.
38. Sigal RJ, Kenny GP, Wasserman DH, Castaneda-Sceppa C, White RD. Physical activity/exercise and type 2 diabetes: a consensus statement from the American Diabetes Association. Diabetes care 2006;29:1433-8.
39. Stofan JR, DiPietro L, Davis D, Kohl HW, 3rd, Blair SN. Physical activity patterns associated with cardiorespiratory fitness and reduced mortality: the Aerobics Center Longitudinal Study. American journal of public health 1998;88:1807-13.
40. Hu FB, Stampfer MJ, Solomon C, et al. Physical activity and risk for cardiovascular events in diabetic women. Annals of internal medicine 2001;134:96-105.
41. Foster-Schubert KE, Alfano CM, Duggan CR, et al. Effect of diet and exercise, alone or combined, on weight and body composition in overweight-to-obese postmenopausal women. Obesity (Silver Spring) 2012;20:1628-38.
42. Pasquali R, Casimirri F, Venturoli S, et al. Body fat distribution has weight-independent effects on clinical, hormonal, and metabolic features of women with polycystic ovary syndrome. Metabolism 1994;43:Clinical &-Clinical 13.
43. Smith AJ, Phipps WR, Arikawa AY, et al. Effects of aerobic exercise on premenopausal sex hormone levels: results of the WISER study, a randomized clinical trial in healthy, sedentary, eumenorrheic women. Cancer epidemiology, biomarkers & prevention : a publication of the American Association for Cancer Research, cosponsored by the American Society of Preventive Oncology 2011;20:1098-106.
44. Jensen MD, Ryan DH, Apovian CM, et al. 2013 AHA/ACC/TOS Guideline for the Management of Overweight and Obesity in Adults: A Report of the American College of Cardiology/American Heart Association Task Force on Practice Guidelines and The Obesity Society. Circulation 2013.
45. Hollander PA, Elbein SC, Hirsch IB, et al. Role of orlistat in the treatment of obese patients with type 2 diabetes. A 1-year randomized double-blind study. Diabetes Care 1998;21:1288-94.
46. Hill JO, Hauptman J, Anderson JW, et al. Orlistat, a lipase inhibitor, for weight maintenance after conventional dieting: a 1-y study. The American journal of clinical nutrition 1999;69:1108-16.
47. Davidson MH, Hauptman J, DiGirolamo M, et al. Weight control and risk factor reduction in obese subjects treated for 2 years with orlistat: a randomized controlled trial. JAMA : the journal of the American Medical Association 1999;281:235-42.
48. Sjostrom L, Rissanen A, Andersen T, et al. Randomised placebo-controlled trial of orlistat for weight loss and prevention of weight regain in obese patients. European Multicentre Orlistat Study Group. Lancet 1998;352:167-72.
49. Bray GA, Ryan DH, Gordon D, Heidingsfelder S, Cerise F, Wilson K. A double-blind randomized placebo-controlled trial of sibutramine. Obesity research 1996;4:263-70.

50. Rolls BJ, Shide DJ, Thorwart ML, Ulbrecht JS. Sibutramine reduces food intake in non-dieting women with obesity. Obesity research 1998;6:1-11.
51. Hansen DL, Toubro S, Stock MJ, Macdonald IA, Astrup A. The effect of sibutramine on energy expenditure and appetite during chronic treatment without dietary restriction. International journal of obesity and related metabolic disorders : journal of the International Association for the Study of Obesity 1999;23:1016-24.
52. Arterburn DE, Crane PK, Veenstra DL. The efficacy and safety of sibutramine for weight loss: a systematic review. Archives of internal medicine 2004;164:994-1003.
53. Kim SH, Lee YM, Jee SH, Nam CM. Effect of sibutramine on weight loss and blood pressure: a meta-analysis of controlled trials. Obesity research 2003;11:1116-23.
54. Yanovski SZ, Yanovski JA. Obesity. The New England journal of medicine 2002;346:591-602.
55. Stevens VJ, Corrigan SA, Obarzanek E, et al. Weight loss intervention in phase 1 of the Trials of Hypertension Prevention. The TOHP Collaborative Research Group. Archives of internal medicine 1993;153:849-58.
56. Dattilo AM, Kris-Etherton PM. Effects of weight reduction on blood lipids and lipoproteins: a meta-analysis. The American journal of clinical nutrition 1992;56:320-8.
57. Jeffery RW, Wing RR, Thorson C, et al. Strengthening behavioral interventions for weight loss: a randomized trial of food provision and monetary incentives. Journal of Consulting & Clinical Psychology 1993;61:1038-45.
58. Metz JA, Stern JS, Kris-Etherton P, et al. A randomized trial of improved weight loss with a prepared meal plan in overweight and obese patients: impact on cardiovascular risk reduction. Archives of internal medicine 2000;160:2150-8.
59. Ditschuneit HH, Flechtner-Mors M, Johnson TD, Adler G. Metabolic and weight-loss effects of a long-term dietary intervention in obese patients. American Journal of Clinical Nutrition 1999;69:198-204.
60. Heymsfield SB, van Mierlo CA, van der Knaap HC, Heo M, Frier HI. Weight management using a meal replacement strategy: meta and pooling analysis from six studies. International journal of obesity and related metabolic disorders : journal of the International Association for the Study of Obesity 2003;27:537-49.
61. Wadden TA, Butryn ML, Byrne KJ. Efficacy of lifestyle modification for long-term weight control. Obesity research 2004;12 Suppl:151S-62S.
62. Lichtman SW, Pisarska K, Berman ER, et al. Discrepancy between self-reported and actual caloric intake and exercise in obese subjects. New England Journal of Medicine 1992;327:1893-8.
63. Bray GA, Blackburn GL, Ferguson JM, et al. Sibutramine produces dose-related weight loss. Obesity research 1999;7:189-98.
64. Wadden TA, Berkowitz RI, Sarwer DB, Prus-Wisniewski R, Steinberg C. Benefits of lifestyle modification in the pharmacologic treatment of obesity: a randomized trial. Archives of internal medicine 2001;161:218-27.
65. Stafford RS, Farhat JH, Misra B, Schoenfeld DA. National patterns of physician activities related to obesity management. Arch Fam Med 2000;9:631-8.

66. Wadden TA, Anderson DA, Foster GD, Bennett A, Steinberg C, Sarwer DB. Obese women's perceptions of their physicians' weight management attitudes and practices. Arch Fam Med 2000;9:854-60.
67. Power ML, Cogswell, M.E., Schulkin J. Obesity Prevention and Treatment Practices of U.S. Obstetrician-Gynecologists. Obstetrics and gynecology 2006;108:961-8.
68. Frank A. Futility and avoidance. Medical professionals in the treatment of obesity. JAMA : the journal of the American Medical Association 1993;269:2132-3.
69. Kushner RF. Barriers to providing nutrition counseling by physicians: a survey of primary care practitioners. Prev Med 1995;24:546-52.
70. Moran LJ, Brinkworth G, Noakes M, Norman RJ. Effects of lifestyle modification in polycystic ovarian syndrome. Reproductive biomedicine online 2006;12:569-78.
71. Clark AM, Thornley B, Tomlinson L, Galletley C, Norman RJ. Weight loss in obese infertile women results in improvement in reproductive outcome for all forms of fertility treatment. Hum Reprod 1998;13:1502-5.
72. Reddy UM, Wapner RJ, Rebar RW, Tasca RJ. Infertility, assisted reproductive technology, and adverse pregnancy outcomes: executive summary of a National Institute of Child Health and Human Development workshop. Obstetrics and gynecology 2007;109:967-77.
73. Schieve LA, Meikle SF, Ferre C, Peterson HB, Jeng G, Wilcox LS. Low and very low birth weight in infants conceived with use of assisted reproductive technology. The New England journal of medicine 2002;346:731-7.
74. Kissin DM, Kulkarni AD, Kushnir VA, Jamieson DJ, National ARTSSG. Number of embryos transferred after in vitro fertilization and good perinatal outcome. Obstetrics and gynecology 2014;123:239-47.
75. Kulkarni AD, Jamieson DJ, Jones HW, Jr., et al. Fertility treatments and multiple births in the United States. The New England journal of medicine 2013;369:2218-25.
76. Moran L, Tsagareli V, Norman R, Noakes M. Diet and IVF pilot study: short-term weight loss improves pregnancy rates in overweight/obese women undertaking IVF. The Australian & New Zealand journal of obstetrics & gynaecology 2011;51:455-9.
77. Sim KA, Dezarnaulds GS, Denyer MR, Skilton MR, Carterson ID. Weight loss improves reproductive outcomes in obese women undergoing fertility treatment: a randomized controlled trial. Clinical Obesity 2014;4:61-8.
78. Legro RS, Barnhart HX, Schlaff WD, et al. Clomiphene, metformin, or both for infertility in the polycystic ovary syndrome. The New England journal of medicine 2007;356:551-66.
79. Group LAR. The Look AHEAD study: a description of the lifestyle intervention and the evidence supporting it. Obesity research 2006:In Press.
80. Committee on Practice B-G. ACOG Practice Bulletin Number 131: Screening for cervical cancer. Obstetrics and gynecology 2012;120:1222-38.
81. Hatch R, Rosenfield RL, Kim MH, Tredway D. Hirsutism: implications, etiology, and management. . American Journal of Obstetrics & Gynecology 1981 Aug 1;140:815-30.
82. Pache TD, Hop WC, Wladimiroff JW, Schipper J, Fauser BC. Transvaginal sonography and abnormal ovarian appearance in menstrual cycle disturbances. Ultrasound in Medicine & Biology 1991;17:589-93.
83. Kroenke K, Spitzer RL, Williams JB. The PHQ-9: validity of a brief depression severity measure. Journal of general internal medicine 2001;16:606-13.

84. Ware JE SK, Kosinski M, Gandek B. SF-36 health survey manual and interpretation guide. Boston: New England Medical Center,: The Health Institute; 1993.
85. Rosen R, Brown C, Heiman J, et al. The Female Sexual Function Index (FSFI): a multidimensional self-report instrument for the assessment of female sexual function. Journal of sex & marital therapy 2000;26:191-208.
86. Rosen RC, Riley A, Wagner G, Osterloh IH, Kirkpatrick J, Mishra A. The international index of erectile function (IIEF): a multidimensional scale for assessment of erectile dysfunction. Urology 1997;49:822-30.
87. Boivin J, Takefman J, Braverman A. The fertility quality of life (FertiQoL) tool: development and general psychometric properties. Hum Reprod 2011;26:2084-91.
88. Chung F, Yang Y, Liao P. Predictive performance of the STOP-Bang score for identifying obstructive sleep apnea in obese patients. Obesity surgery 2013;23:2050-7.
89. Johns MW. A new method for measuring daytime sleepiness: the Epworth sleepiness scale. Sleep 1991;14:540-5.
90. Heinze G, Schemper M. A solution to the problem of separation in logistic regression. Statistics in medicine 2002;21:2409-19.
91. Laird NM, Ware JH. Random-effects models for longitudinal data. Biometrics 1982;38:963-74.
92. Zeger SL, Liang KY, Albert PS. Models for longitudinal data: a generalized estimating equation approach. Biometrics 1988;44:1049-60.
93. Donders AR, van der Heijden GJ, Stijnen T, Moons KG. Review: a gentle introduction to imputation of missing values. Journal of clinical epidemiology 2006;59:1087-91.
94. Little RR, DB. Chapter 15: Nonignorable Missing-Data Models. Statistical Analysis with Missing Data,. 2nd ed. ed. New York, NY: Wiley; 2002.

15 Human Subjects/Informed Consent/Female

COMBINED INFORMED CONSENT AND
HIPAA AUTHORIZATION FORM
Penn State College of Medicine
The Milton S. Hershey Medical Center

**Protocol Title:**	Improving Reproductive Fitness with Pretreatment with Lifestyle Modification in Obese Women with Unexplained Infertility:<br>FIT-PLESE
**Principal Investigator:**	Richard Legro, M.D.<br>Obstetrics & Gynecology Department, Division of Reproductive Endocrinology<br>500 University Drive, Room C3604, MCH103<br>Hershey, PA 17033
**Emergency Contact:**	Weekdays: 8:00 a.m. to 5:00 p.m. (717) 531-8478.<br>After hours call (717) 531-8521 and ask for the Ob-Gyn doctor on 24-hour call.

Why am I being asked to volunteer?

We are asking you to be in this research because you have been diagnosed as being overweight with unexplained infertility and are seeking pregnancy. Your participation is voluntary which means you can choose whether or not you want to participate. If you choose not to participate, there will be no loss of benefits to which you are otherwise entitled. Before you can make your decision, you will need to know what the study is about, the possible risks and benefits of being in this study, and what you will have to do in this study. The research team is going to talk to you about the research study, and they will give you this consent form to read. You may also decide to discuss it with your family, friends, or family doctor. You may find some of the medical language difficult to understand. Please ask the study doctor and/or the research team about this form. If you decide to participate, you will be asked to sign this form.

What is the purpose of this research study?

We are asking you to be in this research because you have been diagnosed as being overweight with unexplained infertility and are seeking pregnancy.

This research is being done to find out the effects of lifestyle modification in obese women with unexplained infertility. Phase 1 of the study is 16 weeks and includes two types of lifestyle modifications: 1) increased physical activity by increasing steps per day OR 2) a calorie restricted diet using meal replacements and a weight loss medication called Orlistat (Alli) with increased physical activity. Orlistat 60mg is a United States Food and Drug Administration (FDA) approved medication to be taken with meals and works by reducing the absorption of fat in a meal.

After completion of Phase 1 lifestyle modification, you will move to Phase 2 of the study. This will involve 3 cycles of infertility treatment involving ovarian stimulation with an oral medication (clomiphene citrate) followed by triggered ovulation using hCG injection and single intrauterine insemination. Clomiphene Citrate is approved by the FDA for the treatment of infertility. hCG (human chorionic gonadotropin) is an injectable medicine to trigger ovulation and is also FDA approved.

Approximately 380 women will take part in this research study nationwide as part of the Reproductive Medicine Network (RMN).

How long will I be in the study?

If you agree to take part, it will take you about 18 months to complete this research study. Below is a time table that lists the visits and their length of times:

Visit Name	# of Visits	Approximate Length of time
Screening	1	2-3 hours
Baseline	1	1 hour
Phase 1-Lifestyle Monthly Visit 1, 2, & 3	3	1 hour each
Lifestyle Modification Close-out/End of Phase 1	1	1 ½ hours
Phase 2- Infertility Treatment Baseline cycle 1	1	1 hour
Monitoring	1+ additional as required(varies)	30 minutes
Insemination	Up to 3	1 hour
Serum Pregnancy Test	2 + additional as required	10 minutes
Infertility Treatment Close-out/End of Phase 2	1	1 ½ hours
Pregnancy 16 weeks	1	30 minutes
Pregnancy 24 weeks	1	30 minutes

Pregnancy 32 weeks	1	30 minutes
POSSIBLE TOTALS=	***17 visits***	***13 hours***

How many subjects will be in the study?

Approximately 380 women and their partner/spouse will take part in this research study nationwide as part of the Reproductive Medicine Network (RMN). Approximately 30 will be from the Penn State Hershey Medical Center.

What am I being asked to do?

At initial contact either by email, telephone, or in clinic, you will be screened with a brief eligibility questionnaire to determine if you qualify to take part in this research. If you are determined to be eligible, you will be scheduled for a screening visit. If you have had any of these tests performed recently within the time limitations set forth in the study, the results of the tests will be recorded and the tests will not be repeated for this study. This consent form will be reviewed by you and the study coordinator. The purpose of the study, all procedures involved, the risks, and the time commitment related to this study will be discussed. Once all questions have been answered, you will sign this consent form. A copy of this signed consent will be given to you for your records.

Below is a brief chart explaining the visits and procedures to be completed for the study. Following this chart, you will find explanations of the visits and each procedure in more detail.

Phases	Phase 1: Lifestyle Intervention (16 weeks)				Phase 2: Infertility Treatment						Phase 3: Pregnancy
Visit Titles	*Screen (can be done in 2 visits)*	*Baseline/Drug Assign*	*Month Visit 1, 2 & 3*	*End of Phase 1 (week 16)*	*Cycle 1 Baseline (day1-3 of menses)*	*Monitor Visit *(cycle day 8-12)*	*IUI*	*UrinePreg Test ***	*Serum hCG ****	*End of Phase 2*	*16, 24, 32 week Pregnancy Visit*
Informed Consent	X										
Medical Assessment	X										
Vital Signs & Biometrics	X	X	X	X	X					X	X
Complete Physical Exam	X										
Gynecological Exam and pap smear	X										
Acne/Hirsutism Assessment	X			X						X	
Transvaginal ultrasound	X				X	X					
Sonohysterogram or Hysterosalpingogram	X										
Questionnaires & QOL surveys	X			X						X	
Urine pregnancy test	X		X	X	X			X (@ home)			
Serum pregnancy test									X		
Blood work for Rubella, Varicella, HIV (optional)	X										
Blood work for female hormones	X										
Blood work for safety labs, lipid panel and HgbA1C	X			X						X	
Blood work for central core	X	X	X	X	X					X	X
Blood work and urine for reserve ancillary studies (optional)	X	X	X	X	X					X	X
Blood work for Whole blood (optional)		X									
Blood work for RMN Repository (optional)		X									
Vaginal Swab	X				X						X
Rectal swab	X				X						X
Saliva sample (optional)		X		X							
Dispense meds		X	X		X	X					
Collect meds			X	X		X				X	
Order meal replacements (if applicable)		X	X								
Dispense Fitbit and Aria scale	X	X									
Dispense diaries		X	X		X	X					
Collect diaries			X	X		X				X	
Adverse Events & Concomitant Meds		X	X	X	X	X		X		X	X
Intrauterine Insemination							X				
MALE PROCEDURES											
Informed Consent	X										
Medical Assessment	X										

Vital signs & Biometrics	X									X	
Questionnaires & QOL surveys	X									X	
Semen analysis/collection	X						X				
Semen for RMN Repository (optional)	X										
Blood work and urine for RMN repository (optional)	X										
Saliva sample (optional)	X										

*Monitor visits will be repeated on an individual basis until hCG administration
**To be done 2 weeks after insemination at home. Repeat cycles until pregnancy occurs or treatment cycles end; may have hCG up to 3 times (cycles). If positive pregnancy, will schedule visit for serum pregnancy test and move to Phase 3 for follow-up. If negative, start new cycle.
***Will repeat hCG 2 days after + result. OB ultrasound will be scheduled 14-21 days from + result.

<u>Screening Visit</u> - This visit will take approximately 2 hours to complete. You will report to the medical center after you have had *<u>nothing to eat or drink 8 hours prior to your visit</u>*. The following procedures are research related and will take place during this visit:

- This consent form will be reviewed by you and the study coordinator. You will have an opportunity to read this consent form in full and ask any questions you may have about the procedures involved, risks and time commitments related to this study. Once all of your questions have been answered, you will sign this consent form. A copy will be provided to you for your records.
- Your past medical and menstrual history will be recorded. This form will ask a series of questions about your medical health, family health history, reproductive and gynecological history, pregnancy history, and current use of medications.
- Your height, weight, vital signs (blood pressure and pulse) and hip and abdominal circumference will be collected and your BMI will be calculated.
- A urine pregnancy test will be performed.
- A physical exam including breast and pelvic exam will be performed by the physician if not done within the past 12 months. A pap smear will be collected if you are 21 or older and have not had one within the time period specified by current guidelines. A vaginal swab will also be collected and tested for vaginal microbiome (normal organisms found in the vagina). This is done by swabbing the vaginal wall using a cotton swab and then placed in a special media. A rectal swab will also be collected and tested for stool microbiome (normal organisms found in the gut). This is done by swabbing the rectal wall using a cotton swab and then placed in a special media.
- The amount of acne lesions on your face will be counted and your hirsutism (excessive hair growth) assessment will be completed.
- You will be given 9-10 questionnaires to complete. A Risk Factor for Genetic Disorders questionnaire is completed to determine if you have an increased risk of having a baby with a genetic disorder. Any potential problems that may lead to complications during pregnancy will be addressed at this time and we may refer you to a genetics counselor prior to starting the study. The Female Sexual Function Index (FSFI) and Female Sexual Distress Scale (FSDS)

will assess your sexual function. The PHQ-9 will assess your feelings for depression. The Short Form 12 (SF 12) will assess your daily activities. FertiQoL will assess how your infertility affects your thoughts and feelings. The Stop Bang Questionnaire and Epworth Sleepiness Scale (ESS) will assess your sleep habits. The ASA24 and the Diet History Questionnaire II (DHQ II) will be completed to assess your past and current dietary intake. You will be free to skip any questions that you would prefer not to answer.

- Blood work will be drawn to determine your eligibility for the study. This blood will consist of hormone levels to evaluate you for specific causes of infertility. If no reason for your infertility is identified, then you will be considered to have unexplained infertility. You will also have blood work to be sure your liver and kidneys are working normally and that you are not anemic. Approximately 8 tablespoons(6 tablespoons if not consented to optional section of study) of blood will be drawn at this visit for the following:

  - TSH, Prolactin, AMH and/or FSH, E2, and HgbA1C

  - Safety labs such as a blood count, lipid panel and liver and kidney tests.

    **If you have already had some of these hormone tests and a lipid panel done within the past year and the results were within normal range and we can obtain a copy of these results, you will not need to have these tests repeated.*

  - Blood will be collected for testing at the central core laboratory. Each site participating in this study will collect a blood sample from every participant at screening and each visit thereafter to be tested by this central core lab for hormone levels and other tests as decided by the RMN.

  - Blood (2 tablespoons) and urine reserve samples will also be collected at this time and stored on site to be used for future testing if you have consented to this optional section of the study.

  - Additionally you will be offered optional blood test to determine if you are immune to rubella (German Measles) and Varicella (Chicken Pox), or to see if you are infected with the HIV virus. If any of these tests are abnormal, you will be referred for appropriate treatment and counseling, however, they will not prevent you from being eligible for the study. If the HIV test is positive, we must report it to the State Health Department. These tests are <u>NOT</u> included or required for the study and will be at your cost.

The following procedures are a standard form of care and would be performed as part of an infertility work up even if you were not a participant in the study.

- A transvaginal ultrasound will be performed. This involves inserting an ultrasound probe into your vagina to visualize your ovaries and uterus. Measurements and ultrasound pictures will be recorded of your ovaries and uterus.

- At this time, if you have had not had a test within the past 3 years to determine that your fallopian tubes are open, a sonohysterogram (SHG) or hysterosalpingogram (HSG) will be completed. During the SHG, sterile saline fluid is injected through an intrauterine catheter

that contains a balloon. The balloon is inflated and the shape of your uterus can be seen and the fluid that accumulates in the back area of your uterus determines that at least one fallopian tube is open. If it can't be determined by the SHG, it will be necessary for a different procedure to be performed. An HSG procedure is done in the radiology department using radio-opaque contrast dye that is injected into the uterus and is visualized flowing through the fallopian tube. If you have been pregnant within the last three years and your pregnancy and delivery were uncomplicated, the SHG or HSG may not be necessary. You will be separately consented for these procedures if you need one of them.

- Your partner will need to provide a semen sample for analysis. A semen analysis is required to determine a sperm concentration of greater than 5 million total motile sperm in an ejaculate for entry into the study. If your partner/spouse has documentation with the past year of a semen analysis, he will be asked to sign a medical release form permitting the coordinator to obtain his semen analysis records, and he will not need to repeat the semen analysis testing if results are within study limits.

Randomization/Beginning of Phase 1 Lifestyle Modification

After the screening visit, if you are eligible to continue in the research, you will be scheduled for your baseline visit to begin Phase 1 of the study - Lifestyle Modification. You will be randomly assigned to receive one of the two study treatments. Randomization means whichever study treatment you receive it will be determined purely by chance, like a flip of a coin. You will have an equal chance to receive either increased physical activity only or increased physical activity with dietary modifications and medication. Neither you nor your physician will be able to decide which group you are assigned to. The following is a detailed description of the lifestyle treatments you could receive:

-Lifestyle Arm A: Increased Physical Activity

In this arm, you will be required to increase your physical activity. We will use your Fitbit measurements from screening to determine your baseline average number of steps per day. You will be asked to increase your amount of steps by 500 per day per week until you have reached the upper limit of 10,000 steps per day. You will be asked to then maintain that amount of steps over the 16 week period. There will be no restrictions on your diet. You will be given a prenatal vitamin with 800mcg of folic acid to be taken once a day.

-Lifestyle Arm B: Increased Physical Activity with weight loss

In this arm, you will be required to achieve weight loss through restricting your calories, increasing your physical activity and weight loss medication. You will receive information on ordering meal replacements and also receive a medication during this 16 week treatment period. You will take one tablet of Orlistat 60 mg (brand name Alli) twice a day at lunch and dinner. You will be instructed by the study coordinator when to begin your medication. You will continue this medication for 14 weeks with a 2 week break from medication before moving on to the next phase of the study. You will be given a prenatal vitamin with folic acid to be taken once a day at least 2 hours before or after Orlistat to ensure adequate vitamin effects because the Orlistat can interfere with the absorption of fat soluable vitamins, such as Vitamin D and beta-carotene. You

will also receive a complete daily diet plan for the 16 weeks with meal replacements (MR) which will include two prepared entrees for lunch and dinner and a cereal bar or beverage. You will be provided with the meal replacements through participation in the study as we will be considering the meal replacements as a prescribed medical treatment. In addition, you will be instructed to also consume 2 servings of fruit (1 serving= 1 small-medium fresh fruit, ½ canned or fresh fruit, 1 cup berries), 3 servings of vegetables (1 serving= ½ cooked vegetables, 1 cup raw vegetables), and 2 servings of low fat dairy (1 serving= 1 cup skim milk, 1 container 6 or 8 oz yogurt, 1 ½ oz natural cheese) per day. This will provide you about 1100 calories. You will be required to consume a total of 1200 calories per day so 100 calories can be consumed with something of your choice. You will also be required to follow the increased physical activity plan as described in Lifestyle A.

Both groups will receive the following during their baseline visit:

- A weight measurement and vital signs will be recorded.
- You will be dispensed a Fitbit Wireless Activity monitor. This will be used throughout the study to determine your daily steps. You will be instructed to download the Fitbit app to your smartphone and sync your Fitbit to the app. If you are unable to use the smart phone app, instructions will be provided to sync to your home computer. All data will be sent via Wi-Fi to the secure database for review.
- You will be dispensed an Aria Scale to monitor your weight. You will be required to weigh yourself weekly. This Wi-Fi smart scale will send your data to the secure database for review. If you are unable to use the Aria scale wirelessly, you will be required to contact the study coordinator via email, text or phone call to report your result.
- You will be given daily journal logs to complete. On these logs you will record menstrual cycles, dates when you took your study medication, intercourse frequency and side effects you have from the medications. You will also keep a list of all medications such as over-the-counter, herbal, or vitamins you are taking.
- If you are assigned to Arm B you will receive your medication, vitamins, and instructions on ordering your meal replacements.
- You will have approximately 6 tablespoons of blood drawn for the following:

  - Blood will be drawn for the central core laboratory.

  - Blood and urine for reserve will be collected (if consented).

  - If you have consented for your blood to be drawn for the study whole blood collection and/or the RMN Biologic Repository, this will be drawn at this time. The purpose of the whole blood collection is to identify certain key genes in your DNA (called polymorphisms) that may predict a response to the medications used in this trial. The repository collection will be used for future studies. These specimens will only be drawn if you have provided consent to do so.

- If you have consented to the collection of saliva for cortisol testing, this will be collected at this time. Cortisol is a stress hormone. This will be done by removing a cotton swab from

the package and placing the swab under your tongue for approximately 60-120 seconds. Once the time is complete, the swab is inserted into the tube and sealed for storage in a freezer.

<u>Monthly Visit (1, 2, & 3)-</u>

You will return at 4, 8 and 12 weeks for a monthly visit. The following procedures will occur at each monthly visit:

- A weight measurement and vital signs will be recorded.
- A urine pregnancy test will be done.
- Approximately 3 tablespoons of blood and a urine specimen will be collected for the ancillary reserve (if consented) and central core laboratory.
- Assessment of your adverse events and concomitant medications will be collected.
- The study coordinator will collect all menstrual/intercourse diaries and any leftover medication and a pill count will be performed for compliance, if applicable.
- New menstrual/intercourse diaries will be re-distributed.
- If applicable, medication will be dispensed and you will be instructed to order your meal replacements.
- During the 3rd monthly visit, you will be dispensed kits for saliva collection to be returned at Closeout Visit (week 16), if consented.

<u>Lifestyle Modification Close-out/End of Phase 1-</u>

At approximately 16 weeks after completing your lifestyle modification phase, you will have a closeout visit for this phase. You will need to be fasting for this visit. The following procedures will occur at this visit:

- Measurements of height, weight, abdominal circumference and vital signs will be recorded.
- A urine pregnancy test will be done.
- Hirsutism and acne assessments will be completed.
- Approximately 4 tablespoons of blood will be collected for safety labs, central core labs and blood and urine reserve for the ancillary storage (if consented).
- If you consented to the collection of saliva for cortisol testing, this sample will again be collected at this visit.
- You will be asked to complete the quality of life questionnaires as you did at the screening visit including FSFI, FSDS, PHQ-9, SF-12, FertiQOL, Stop Bang, ESS, and ASA24.
- Your menstrual and diary logs will be collected.
- Any remaining medications will be collected and pill count will be performed for compliance.
- Assessment of your adverse events and concomitant medications will be collected.

<u>Baseline Visit Infertility Treatment Phase 2-</u>

You will call on the first day of your next menstrual period after completing Phase 1 to schedule your baseline visit for infertility treatment between menstrual cycle day 1-5. At this visit the following procedures will occur:

- A weight measurement will be collected.

- You will have a transvaginal ultrasound and a repeat vaginal and rectal swab for microbiome (normal organisms found in the vagina) testing. This is done by swabbing the vaginal wall using a cotton swab and then placed in a special media
- A urine pregnancy test will be done.
- You will have 3 tablespoons of blood drawn for the central core and blood and urine reserve for the ancillary storage (if consented).
- You will be dispensed menstrual and intercourse diaries.
- Your study medications will be dispensed to you at this time. You will receive capsules containing Clomiphene Citrate. You will be instructed to begin your medications if your urine pregnancy test is negative and your ultrasound results are within a normal range. You will also receive hCG injection medication to be used when directed by the study physician prior to your insemination. You and your partner will be taught the technique for administering an intramuscular injection. Below is a table describing the medication Clomiphene Citrate and its dosages:

Study Medication	Interval	Dose	Method	Start	Finish	Future Cycles
Clomiphene Citrate	Daily	100mg	Capsule	Day 3*	Day 7	Can be started at 50-150 mg/day= 1 to 3 pills

**Can be started +/- 2 days (menstrual cycle day 1 to 5)*

<u>Monitor Visits (Cycle day 8 to 12 and then beyond as required)-</u>

You will be required to return to the clinic within 3 days after completing your 5 day treatment of Clomiphene Citrate. A transvaginal ultrasound will be performed. You will report any adverse events or concomitant medications you are taking. You may be required to return to the clinic regularly for additional monitoring visits until you have met the criteria for hCG administration. These visits will be individualized and based on the physician's decision. At this visit during cycle 2 and 3, you will return your medication bottles and menstrual diaries from the previous cycle.

When your main follicle (cyst which contains an egg) gets to a certain size, you will be instructed to self- or partner- administer, as previously instructed, an injection of hCG at a dose of 10,000 IU. A cycle may be cancelled if the lead follicles become too large or too small after 18 days of treatment.

<u>Intrauterine Insemination Visit-</u>

You must return to the clinic within 44 hours of the hCG shot. Your partner will provide a semen sample. This sample will be washed and prepared for the insemination. A speculum will be placed in your vagina and a thin flexible catheter will be placed into your cervix. The sperm will be injected through the catheter and into your uterus. During cycle 1 and cycle 2, at this visit you will be dispensed medication and your menstrual diaries for the next cycle. You will be instructed to NOT start your medications until the end of the cycle after a confirmed negative urine pregnancy test.

Urine Pregnancy Test-

Two weeks after insemination you will do a urine pregnancy test at home and call the study coordinator with your results.

If you have a *positive urine pregnancy test*, you will be required to return to the clinic for a blood pregnancy test (quantitative bhCG). If your bhCG is positive you will return 2 days later for repeat bhCG to check for a rising level. If the level is not rising, you will begin the next cycle of treatment with the start of your menstrual cycle. If the bhCG level has risen, you will be scheduled for an ultrasound approximately 14-21 days after the positive result. This will determine the location of your pregnancy and number of gestational sacs. A repeat pregnancy ultrasound will be done 7-14 days after the first one to assess fetal heart beat. You may need additional ultrasounds done to evaluate the progress of the pregnancy if necessary.

Pregnancy care is not part of the study. You will be scheduled with your obstetrician for follow up prenatal care. If you do not have a doctor who delivers babies, you will be referred to one. Before being released form the study, you will be asked to sign a medical release form so the study coordinator can obtain your pregnancy and delivery information. You will be contacted at 12 weeks of pregnancy to see how your pregnancy is progressing.

Follow up Initiation of Cycle 2 & 3-

If your urine pregnancy test is negative after a cycle, you may start another cycle of treatment. You may participate for a maximum of 3 completed cycles. A cycle may be cancelled if any significant adverse reactions develop in response to the medications, if you are unable to have the hCG injection, or if you request the cycle to be cancelled. The study coordinator will provide you with instructions to start your medication on cycle day 3 (+ or – 2 days). The study physician may change the start dose of your Clomiphene Citrate. You will be scheduled for a return monitoring visit during menstrual cycle day 8-12.

Infertility Treatment Close-out/End of Phase 2-

You will return for an end of study drug visit and will need to be fasting for this visits. If you do not conceive, this visit will take place at the end of the 3rd cycle. If you do conceive, the visit will take place as soon as possible after pregnancy is confirmed. The following procedures will be done:

- Measurements including height, weight, neck, waist and hip circumference will be collected.
- Hirsutism and acne assessments will be done.
- You will return remaining study drug and diaries.
- Assessment of adverse events and concomitant medications will be done.
- You will be asked to repeat the quality of life questionnaires as you did at the screening visit, excluding the DHQII. If you are pregnant, the FertiQOL questionnaire will be skipped.
- Approximately 4 tablespoons of blood will be collected for safety labs, central core labs and blood and urine reserve for ancillary storage (if consented).

Pregnancy Follow-up/Phase 3-

If you become pregnant during the study, you will participate in Phase 3. You will be seen for 3 visits at 16 weeks, 24 weeks and 32 weeks of pregnancy. You will return to the clinic for a brief visit and will need to be fasting for these visits. The following will occur:

- Measurements including waist and hip measurements, vital signs and weight will be obtained.
- You will have about 4 tablespoons of blood for central core labs, and blood and urine reserve for ancillary storage (if consented).
- Assessment of pregnancy adverse events will be done.
- A vaginal and rectal swab for stool microbiome will be collected.
- A medical release form will be signed to obtain copies of your prenatal record and delivery records.
- You will be offered the opportunity to participate in the Reproductive Medicine Network (RMN) Pregnancy Registry. The Registry is a separate study, so we will ask you to sign a separate consent form if you are interested.

What are my responsibilities if I take part in this research?

If you take part in this research, your major responsibilities will include:

- Compliance with study visits
- Reviewing all of your medications and side effects with your study doctor
- Participation in study related procedures
- Participation in the collection of all blood work
- Taking and storing your study medication as instructed and returning all of the unused study drug and empty containers to the study site at each visit
- Compliance with the documentation and collection of menstrual and intercourse daily logs
- Compliance with the collection of all data for your exercise and meal replacement use

What are the possible risks or discomforts?

Below is a table listing all the tests and procedures involved in this research and their related discomforts or risks. You should check with the study doctor before starting any new prescriptions, over the counter medications, vitamins or herbal supplements.

Test or Procedure	Discomforts and Risks Associated
Standard venipuncture for blood work	Slight pinch or pin prick, discomfort, black and blue mark at the site of puncture, small blood clot, infection or bleeding at the site, and fainting during the procedure
Transvaginal ultrasound	Abdominal or pelvic discomfort

Sonohysterogram/Hysterosalpingogram	Pain, bleeding, damage to the uterus, pelvic infection, interrupting an unrecognized pregnancy, small amount of radiation exposure, allergic reaction to the radio-opaque dye
Clomiphene Citrate	Visual changes (such as blurring of vision, double vision, light sensitivity waves), abdominal pain, nausea, vomiting, constipation, mood changes, headache, hot flashes, respiratory difficulty, fatigue, abnormal endometrial thickening, multiple pregnancies, formation of ovarian cyst causing ovarian hyperstimulation syndrome, ovarian enlargement, breast discomfort, abnormal uterine bleeding, bloating, hair loss, arrhythmias, chest pain, allergic reaction, palpitations, and stroke or pulmonary embolism
Low calorie diet	Hunger, weakness, changes in your electrolytes, mood changes, depression, gall stone formation from rapid weight loss, lowered blood pressure with dizziness, lack of energy, and new onset attack of gout (presents with joint pain)
Daily exercising	Fatigue, muscle strain, joint pain, or injury from repetitive exercise, stroke or heart attack in susceptible individuals
Orlistat (Alli)	Headache, oily spotting, stomach pain/discomfort, gas with discharge, bowel movement urgency, fatty/oily stools, increased bowl movements, back pain, upper respiratory infection, fatigue, anxiety, sleep disorder, dry skin, menstrual irregularities, nausea, rectal pain/discomfort, tooth disorder, rash. Rarely liver injury has occurred. Symptoms include jaundice (yellow color in skin, gums, eye and other tissues due to too much bile in the blood), weakness and abdominal pain
Insemination	Vasovagal response (feeling lightheaded, dizzy, sweating), possible infection, pelvic discomfort, mild vaginal bleeding

Infertility treatments	Emotional distress at various degrees
Questionnaires	Some parts of the questionnaires may make you feel uncomfortable

It is not expected that patients will have all of these side effects. If you are feeling fatigue or dizziness associated with any of the medications, caution should be taken while driving or operating machinery. Side effects are usually temporary and manageable. However, it is possible they could be more serious.

You will be assigned to a study treatment group by chance, like the flip of a coin. The study treatment you receive may prove to be less effective or to have more side effects than the other research treatments or other available treatments.

There is a risk associated with loss of confidentiality if your medical information or your identity are obtained by someone other than the investigators, but precautions will be taken to prevent this from happening.

Although these drugs may result in pregnancy, there is no guarantee that this will result in live birth. Clomiphene has been associated with a 12.5% multiple pregnancy rate. Multiple pregnancies are more likely to end early in preterm labor and are more likely to experience most pregnancy complications including diabetes and hypertensive disorders of pregnancy. It is possible that your pregnancy may be nonviable or a pregnancy is detected in your fallopian tube (ectopic pregnancy) and will require further medical or surgical treatment. The risk for ectopic pregnancy is the same even if you were not participating in the study.

The particular treatments being used in this study may involve risks to you or to the embryo or fetus if you become pregnant and these risks are currently unforeseeable. You will be given pre-conceptional counseling for healthy behaviors in pregnancy throughout this study. You will be followed through study, after a positive pregnancy test, to confirm the location and viability of the pregnancy before being released to your Ob-Gyn physician. The use of these drugs may be associated with an increased risk for fetal malformations, though no specific pattern has been associated with either drug. Clomiphene Citrate has been shown to be genotoxic (cause abnormalities to genes) in in-vitro laboratory tests.

In addition to the risks described above, there may be unknown risks we cannot predict while participating in this research. It is important that you notify your study coordinator if you experience any of these symptoms listed above and keep accurate documentation of these side effects in your daily journal logs. The investigators will let you know if they learn anything that might make you change your mind about participating in this research.

What if new information becomes available about the study?

During the course of this study, we may find more information that could be important to you. This includes information that, once learned, might cause you to change your mind about being in the study. We will notify you as soon as possible if such information becomes available.

What are the possible benefits of the study?

There is no guarantee that you will benefit from this research. The possible benefits you may experience from this research study include weight loss and a better chance for a healthier pregnancy. The information from the blood tests will let you know more about your health such as the levels of hormones in your blood involved in reproduction. The initial ultrasound examination will evaluate your pelvic anatomy, which may identify abnormalities such as ovarian cysts or abnormalities in the uterine lining. The infertility medications and intrauterine insemination may help you become pregnant.

The results of this research may guide the future of treatment of obesity for women with unexplained infertility by establishing effective lifestyle changes and weight loss prior to attempting pregnancy. This research may also help discover a better way to achieve pregnancy and a goal of a healthy pregnancy with live birth by the use of Clomiphene Citrate and intrauterine insemination for women with unexplained infertility.

What other choices do I have if I do not participate?

You do not have to take part in this study to be treated for your condition. Instead of participating in this research, you could:

- Receive commercially available treatments, including: over-the-counter orlistat or other weight loss medication; Clomid or other form of infertility medication along with intrauterine inseminations through your regular doctor.
- Be part of a different research study, if one is available.
- Choose not to be treated for your medical condition

Before you decide if you want to be in this research, we will discuss the other choices that are available to you. We will tell you about the possible benefits and risks of these choices.

The therapy offered in this research is available to you without taking part in this research study.

Will I be paid for being in this study?

You will not receive any payment or compensation for being in this research study.

Will I have to pay for anything?

For costs of tests and procedures that are only being done for the research study:

- The orlistat, Clomiphene Citrate, hCG injection, and vitamins will be provided by RMN at no cost to you.
- You and/or your insurance company will not be charged for the cost of any tests or procedures that are required as part of the research and are within the standard of care (what is normally done) for your condition.
- The research-related tests and procedures that will be provided at no cost to you include:
    - All study required blood tests
    - Physical exam

- Transvaginal ultrasound (s)
- Pap smear
- Sonohysterogram (SHG) and any required blood work needed to perform the test
- Semen Analysis
- Folic acid supplement and prenatal vitamins
- Meal replacements
- Fitbit
- Aria scale
- Intrauterine inseminations
- Supplies for injection medication such as: syringes, alcohol swabs, gauze, and band-aids
- 2 Obstetrical ultrasounds

For costs of medical services for care you would receive even if you were not in this research study:

- You and/or your insurance company will be responsible for the cost of routine medications, tests and procedures that you would receive even if you were not in this research that are not mentioned above as covered.
- You and/or your insurance company will be billed for the costs of these routine tests and procedures in the usual manner.
- You will be responsible for any co-payments, co-insurance and deductibles that are standard for your insurance coverage.
- You will be responsible for any charges not reimbursed by your insurance company.
- Some insurance companies will not pay for routine costs for people taking part in research studies. Before deciding to be in this research you should check with your insurance company to find out what they will pay for.

The following is a list of the non-covered services related to this study:

- Rubella, Varicella, & HIV testing
- Hysterosalpingogram (HSG) and any required blood work to perform the test
- Doxycycline prescription for after the HSG procedure
- Remaining obstetrical ultrasounds after the first 2
- Pregnancy care and delivery costs

If you have any questions about costs and insurance, ask the research study doctor or a member of the research team.

Who is paying for this research study?

The institution and investigators are receiving a grant from the National Institute of Health to support this research.

What happens if I am injured from being in the study?

It is possible that you could develop complications or injuries as a result of being in this research study. If you experience a side effect or injury and emergency medical treatment is required,

seek treatment immediately at any medical facility. If you experience a side effect or injury and you believe that emergency treatment is not necessary, you should contact the principal investigator listed on the first page of this consent form as soon as possible and he/she will arrange for medical treatment.

<u>The Milton S. Hershey Medical Center (HMC)/Penn State College of Medicine (PSU) compensation for injury</u>

- There are no plans for HMC/PSU to provide financial compensation or free medical treatment for research-related injury.
- If an injury occurs, medical treatment is available at the usual charge.
- Costs will be charged to your insurance carrier or to you.
- Some insurance companies may not cover costs associated with research injuries.
- If these costs are not covered by your insurance, they will be your responsibility.

When you sign this form you are not giving up any legal right to seek compensation for injury.

When is the Study over? Can I leave the Study before it ends?

Taking part in this research study is voluntary.

- You do not have to be in this research.
- If you choose to be in this research, you have the right to stop at any time.
- If you decide not to be in this research or if you decide to stop at a later date, there will be no penalty or loss of benefits to which you are entitled.

If you stop being in the research, already collected data may not be removed from the study database. You will be asked whether the investigator can collect medical information from your routine medical care. If you agree, this data will be handled the same as research data. If you withdraw completely from the research study, no further information will be collected and your participation will end. You may discontinue taking part at any time without penalty or loss of benefits to which you are otherwise entitled.

Your research doctor may take you out of the research study without your permission.

- Some possible reasons for this are: you experience serious side effects and continuing the research would be harmful, your condition has become worse, and you did not follow the instructions of the study doctor.
- Also, the sponsor of the research may end the research study early.
- If your participation ends early, you may be asked to visit the research doctor for a final visit.

If you will be in another clinical research study at the HMC/PSU or elsewhere while in this research, you should discuss the procedures and/or treatments with your physician or the study doctors. This precaution is to protect you from possible side effects from interactions of research drugs, treatments or testing.

Who can see or use my information? How will my personal information be protected?

Efforts will be made to limit the use and sharing of your personal research information. In our research files at HMC/PSU we will include these identifiers:

- Study identification number
- Name & Initials
- Address
- Phone number, cell phone number, work number
- Email address
- Social Security number
- Medical Record number
- Date of birth
- Visit date

A list that matches your name with your code number will be kept in a locked file.

Your research records will be labeled with your study identification number, your initials, date of birth and will be kept in a secure area. These records will be kept in the office for the amount of time necessary to complete data analysis and for a minimum of 5 years after completion of the study as required by law.

Results of some of the research-related tests including but not limited to your hormone, safety labs, and lipid blood results, pregnancy test results, ultrasound results, SHG/HSG results, pap smear results and intrauterine insemination information will be kept in your HMC/PSU medical record.

For research records sent to Yale University, you will be identified by your study identification number only. The research records are entered into an electronic password protected database. Only investigators from the HMC/PSU and RMN/Yale University have access to these electronic records for study purposes only. This data is managed by the RMN/Yale University Data Coordinating Center and will be kept indefinitely.

Your research samples for the central core laboratory will be labeled with your study identification number, visit name and visit date and will be stored in the research laboratory in a -80 degree freezer until shipment. These samples are analyzed by the core lab and will be stored there until the sample is exhausted. If any remaining sample is leftover, they will eventually be sent to the RMN Biologic Repository and be owned by the RMN for future use in research until it has been exhausted. The list linking your sample to your identification number will be kept in a password protected computer file. Only investigators involved in this research will have access to this list.

For optional specimens collected as reserve for ancillary studies, you will identified by your study identification number, visit name and visit date. These samples are stored in the research laboratory in a -80 freezer. These samples will remain there until the RMN has decided on additional testing to be completed. These samples will be analyzed by the laboratory designated by the RMN and will be stored at that laboratory until the sample is exhausted. If any remaining sample is leftover, they will eventually be sent to the RMN Biologic Repository and be owned by the RMN for future use in research until it has been exhausted.

For vaginal and rectal microbiome samples, and optional whole blood, stool and saliva samples you will not be identified. The sample will be labeled with a bar code label and a unique identifier. The sample will be stored in the research laboratory until shipment. These samples will remain there until the RMN has decided on the laboratory performing the testing. The samples will be sent to the laboratory and will be stored there until the sample is exhausted. If remaining sample is leftover, they will eventually be sent to the RMN Biologic Repository and be owned by the RMN for future use in research until it is exhausted.

For optional specimens collected for the RMN Biologic Repository, you will not be identified. The sample will be labeled with a bar code label and a unique identifier. The sample will be stored in the Repository until exhausted by the RMN.

To help us protect your privacy, we have obtained a Certificate of Confidentiality from the National Institute of Health. With this Certificate, the researchers cannot be forced to disclose information that may identify you, even by a court subpoena, in any federal, state, or local, civil, criminal, administrative, legislative, or other proceedings. The researchers will use the Certificate to resist any demands for information that would identify you, except as explained below.
The Certificate cannot be used to resist a demand for information from personnel of the United States Government that is used for auditing or evaluation of federally funded projects or for information that must be disclosed in order to meet the requirements of the federal Food and Drug Administration (FDA).
You should understand that a Certificate of Confidentiality does not prevent you or a member of your family from voluntarily releasing information about yourself or our involvement in this research. If an insurer, employer, or other person obtains your written consent to receive research information, then the researchers may not use the Certificate to withhold that information.
The Certificate of Confidentiality does not prevent researchers from disclosing voluntarily, without your consent, information that would identify you as a participant in the research project under the following circumstances: child abuse, intent to harm yourself or others, or if you have reportable communicable disease that state or federal law requires us to report such as Tuberculosis, HIV infection, or Syphilis.

In the event of any publication or presentation resulting from the research, no personally identifiable information will be shared.

Some of these records could contain information that personally identifies you. Reasonable efforts will be made to keep the personal information in your research record private. However, absolute confidentiality cannot be guaranteed.

A description of this clinical trial will be available on http://www.ClinicalTrials.gov, as required by U.S. Law. This Web site will not include information that can identify you. At most, the Web site will include a summary of the results. You can search this Web site at any time.

Electronic Medical Records and Research Results

What is an Electronic Medical Record?
An Electronic Medical Record (EMR) is an electronic version of the record of your care within a health system. An EMR is simply a computerized version of a paper medical record.

If you are receiving care or have received care within HMC (outpatient or inpatient) and are participating in a HMC/PSU research study, results of research-related procedures (i.e. laboratory tests, imaging studies and clinical procedures) may be placed in your existing EMR maintained by HMC.

If you have never received care within HMC and are participating in a HMC/PSU research study that uses HMC services, an EMR will be created for you for the purpose of maintaining any results of procedures performed as part of this research study. The creation of this EMR is required for your participation in this study. In order to create your EMR, the study team will need to obtain basic information about you that would be similar to the information you would provide the first time you visit a hospital or medical facility (i.e. your name, the name of your primary doctor, the type of insurance you have). Results of research procedures performed as part of your participation in the study (i.e. laboratory tests, imaging studies and clinical procedures) may be placed in this EMR.

Once placed in your EMR, these results are accessible to appropriate HMC workforce members that are not part of the research team. Information within your EMR may also be shared with others who are determined by HMC to be appropriate to have access to your EMR (e.g. health insurance company, disability provider, etc).

What information about me may be collected, used or shared with others?

- Name
- Address
- Phone number, cell phone number, work number
- Email address
- Social Security number
- Medical Record number
- Date of birth
- Past, present, and future medical records
- New health information from tests, procedures, visits, interviews, or forms filled out as part of this research study.

Why is my information being used?

Your information is used by the research team to contact you during the study. Your information and results of tests and procedures are used to:

- do the research

- oversee the research
- to see if the research was done right.

Who may use and share information about me?

The following people/groups may see, use, and share your identifiable health information:

- HMC/PSU research staff involved in this study
- The University of Pennsylvania Institutional Review Board, a group of people who review the research study to protect subjects' rights and welfare
- The University of Pennsylvania Human Subjects Protection Office
- The HMC/PSU Human Subjects Protection Office
- The HMC/PSU Research Quality Assurance Office
- Non-research staff within HMC/PSU who need this information to do their jobs (such as for treatment, payment (billing), or health care operations)
- Federal and state agencies (such as the U.S. Food and Drug Administration, the Office for Human Research Protections, the Department of Health and Human Services, the National Institutes of Health, and other U.S. or foreign government bodies that oversee or review research)
- Other researchers and medical centers that are part of this study and their IRBs
- The Data Coordinating Center at Yale University
- Reproductive Medicine Network of the The Eunice Kennedy Shriver National Institute of Child Health and Human Development
- A group that oversees the data (study information) and safety of this research
- Organizations that provide independent accreditation and oversight of hospitals and research
- Public health and safety authorities (for example, if we learn information that could mean harm to you or others, we may need to report this, as required by law)

These groups may also review and/or copy your original PSU/HMC records while looking at the results of the research study. It is possible that some of the other people/groups who receive your health information may not be required by Federal privacy laws to protect your information. We share your information only when we must, and we ask anyone who receives it from us to protect your privacy.

You have the right to see and get a copy of your health information that is used or shared for treatment or for payment. However, you may not be allowed to see or copy certain health information that is a part of this research study. This is only for the period of the study. You will be allowed to see that information when the entire research study is complete.

How long may HMC/PSU use or disclose my personal health information?

Your authorization for use of your personal health information for this specific study does not expire.

Your information may be held in a research database. However, HMC/PSU may not re-use or re-disclose information collected in this study for a purpose other than this study unless:

- You have given written authorization
- The University of Pennsylvania's Institutional Review Board grants permission
- As permitted by law

Can I change my mind about giving permission for use of my information?

You have the right to withdraw your permission for us to use or share your health information for this research study. If you want to withdraw your permission, you must notify the person in charge of this research study in writing using the address on the front of this form. Once permission is withdrawn, you cannot continue to take part in the study.

If you withdraw your permission, we will stop collecting health information about you for this study; we may continue to use and share your health information that we already have if it is necessary for safety and scientific soundness of the research study; and we will not be able to take back information that has already been used or shared with others.

What if I decide not to give permission to use and give out my health information?

You have the right to refuse to sign this form that allows us to use and share your health information for research; however, if you don't sign it, you will not be able to take part in this research study.

Who can I call with questions, complaints or if I'm concerned about my rights as a research subject?

If you have questions, concerns or complaints regarding your participation in this research study or if you have any questions about your rights as a research subject, you should speak with the Principal Investigator listed on page one of this form. If a member of the research team cannot be reached or you want to talk to someone other than those working on the study, you may contact the Office of Regulatory Affairs at the University of Pennsylvania by calling (215) 898-2614.

When you sign this form, you are agreeing to take part in this research study. This means that you have read the consent form, your questions have been answered, and you have decided to volunteer. Your signature also means that you are permitting the HMC/PSU to use your personal health information collected about you for research purposes within our institution. You are also allowing the HMC/PSU to disclose that personal health information to outside organizations or people involved with the operations of this study.

A copy of this consent and HIPAA authorization form will be given to you.

__________________________ _______________________________________

Name of Subject (Please Print) **Signature of Subject** **Date**

____

**Name of Person Obtaining Consent** (Please Print)	**Signature**	**Date**

Optional part(s) of the study

In addition to the main part of the research study, there are other parts of the research. You can be in the main part of the research without agreeing to be in these optional parts. Each optional section is listed below.

Optional Storage of Blood & Urine Reserve for Future Research

As part of an optional study, we want to obtain a sample of your blood and urine to be stored by the RMN for future use. If you agree, your blood and urine will be collected at each visit. Approximately 2 tablespoons of blood will be collected and a urine sample. The sample will be labeled with your study identification number, visit name and visit date. The samples will be stored in a locked laboratory at the HMC/PSU until the RMN has decided on further testing to be done to the specimens. When the RMN has decided to do further testing, your sample will be shipped to the designated laboratory as decided by the RMN. If there is any remaining specimen after the testing is completed, your sample will then be shipped to the RMN Biologic Repository for long term storage. Your samples will be stored for a minimum of 5 years, and perhaps further into the future. The samples will be tested for hormones and other substances in your blood and urine. These samples can be used for other research in the future after this study is over and also be shared with other investigators who collaborate with the RMN. Once your blood has left the HMC/PSU, there is no way to have it returned or withdrawn for testing. You will not be able to withdraw your permission for the use of your blood sample for future use as this time.

ρ

- **These future studies may be helpful in understanding obesity and unexplained infertility.**
- **It is unlikely that these studies will have a direct benefit to you.**
- **The results of these tests will not have an effect on your care.**
- **Neither your doctor nor you will receive results of these future research tests, nor will the results be put in your health record.**
- **It is possible that your blood/urine might be used to develop products or tests that could be patented and licensed. There are no plans to provide financial compensation to you should this occur. If you have any questions, you should contact a member of the research team.**

You should ***initial*** below to indicate your preference for the collection of your blood and urine reserve sample for ancillary studies in the future.

________ I **give my permission** for my blood and urine reserve sample to be collected and stored for future testing.

________ I **decline my permission** for my blood and urine reserve sample to be collected and stored for future testing.

Optional Study Whole Blood Collection

As part of an optional study, we would like to obtain, prior to you starting medication, a sample of your whole blood for DNA testing and to measure other substances in the blood. If you agree, less than 2 tablespoons of blood will collected at the randomization visit. The researchers would like to use your DNA to identify certain sequences of key genes (called polymorphisms) that may predict a response to the medications used in this study. If genes are identified, which are related to the medications used in the study, the sample may also be used in the development of diagnostic or prognostic testes to identify those who will or will not respond to these medications. We also may use this DNA to identify genes that cause infertility. There is no normal or abnormal result produced by the DNA and the researchers will not use the DNA to try to see if you have any genetic diseases or conditions. If you agree to allow us to collect and store a DNA sample from you for future use, your sample will be labeled with a bar code label and a unique identifier. These samples will be stored in in a locked laboratory at the HMC/PSU until shipment to the laboratory analyzing the DNA. The laboratory will be designated by the RMN. Once the DNA has been analyzed, and if there is leftover specimen, your sample will be shipped to the RMN Biologic Repository where it will be stored indefinitely or until the sample is exhausted by the RMN. These samples can be used for other research in the future after the study is over and also be shared with other investigators who collaborate with the RMN. Once your blood has left the HMC/PSU, there is no way to have it returned or withdrawn for testing. You will not be able to withdraw your permission for the use of your blood sample for future use as this time.

- The testing of DNA may provide additional information that will be helpful in understanding the medications used in this trial and the effects on infertility.
- It is unlikely that these studies will have a direct benefit to you.
- The results of these tests will not have an effect on your care.
- Neither your doctor nor you will receive results of these tests, nor will the results be put in your health record.

You should ***initial*** below to indicate your preference for the collection of your DNA sample:

________ I **give my permission** for my DNA sample to be collected and tested under the study.

________ I **decline my permission** for my DNA to be collected and tested under the study.

Optional Study for Cortisol Saliva Collection

As part of an optional study, we would like to obtain a sample of your saliva for cortisol testing. Cortisol is a stress hormone found in saliva. If you agree, a sample of your saliva will be collected by placing a cotton swab under your tongue for 60-120 seconds and then placed in a tube and sealed for storage in a freezer until testing. If you agree to allow us to collect your saliva, your sample will be labeled with a bar code label and a unique identifier. These samples will be stored in a locked

laboratory at the HMC/PSU, until shipment to the laboratory analyzing the saliva. The laboratory will be designated by the RMN. Once the saliva has been analyzed, and if there is leftover sample, your sample will be shipped to the RMN Biologic Repository where it will be stored indefinitely or until the sample is exhausted by the RMN. These samples can be used for other research in the future after the study is over and also be shared with other investigators who collaborate with the RMN. Once your saliva has left the HMC/PSU, there is no way to have it returned or withdrawn for testing. You will not be able to withdraw your permission for the use of your saliva for future use as this time.

- The testing of saliva may provide additional information that will be helpful in understanding the factors of stress in infertility.
- It is unlikely that these studies will have a direct benefit to you.
- The results of these tests will not have an effect on your care.
- Neither your doctor nor you will receive results of these tests, nor will the results be put in your health record.

You should ***initial*** below to indicate your preference for the collection of your saliva sample:

________ I **give my permission** for my saliva sample to be collected and tested under the study.

________ I **decline my permission** for my saliva to be collected and tested under the study.

Optional Blood and Urine for RMN Biologic Repository

As part of an optional study, we are obtaining a sample of your blood to be stored in a Biologic Repository by the Reproductive Medicine Network for future use. If you agree, a urine sample and approximately 2 tablespoons of your blood will be collected at the randomization visit and shipped to the RMN Biologic Repository (a central location) and stored for a minimum of 5 years, and perhaps into the future. Your sample will be tested for DNA and to measure other substances in your blood. If you agree to allow us to collect and store a blood sample from you for future use in the repository, your sample will be labeled with a bar code label and unique identifier. These samples will be stored in a locked laboratory at the HMC/PSU until shipment to the repository. If you consent to the collection of your blood for the repository, it will be kept indefinitely or until the sample is exhausted by the Reproductive Medicine Network. Once your blood has left the HMC/PSU, there is no way to have it returned or withdrawn for testing. You will not be able to withdraw your permission for the use of your blood sample for future use as this time.

We are also collecting blood and urine as a part of this study. If you agree, we would like to retain any leftovers after their use in the study for future use, including measuring other substances from your blood and urine. If you agree to let us retain these samples they will be stored in a locked laboratory at the HMC/PSU until shipment to the repository. If you consent to the retention of these samples blood and urine for the repository, they will be kept indefinitely or until the sample is exhausted by the Reproductive Medicine Network. Once your blood and urine has left the HMC/PSU, there is no way to have it returned or withdrawn for testing. You will not be able to withdraw your permission for the use of your blood and urine sample for future use at this time.

- The testing of your blood and urine may provide additional information that will be helpful in understanding the medications used in this trial, obesity and unexplained infertility.
- It is unlikely that these studies will have a direct benefit to you.
- The results of these tests will not have an effect on your care.
- Neither your doctor nor you will receive results of these tests, nor will the results be put in your health record.
- It is possible that your blood/urine might be used to develop products or tests that could be patented and licensed. There are no plans to provide financial compensation to you should this occur. If you have any questions, you should contact a member of the research team.

You should ***initial*** below to indicate your preference for the collection of your blood and urine sample for the RMN Biologic Repository:

______ I **give my permission** for my blood and urine sample to be collected and sent to the repository for future testing.
______ I **decline my permission** for my blood and urine sample to be collected and sent to the repository for future testing.

You should ***initial*** below to indicate your preference for the storage of your leftover blood and urine sample for the RMN Biologic Repository:

______ **I give my permission** for my leftover blood and urine sample(s) to be retained and sent to the repository for future testing.

______ **I decline my permission** my leftover blood and urine sample(s) to be retained and sent to the repository for future testing.

Signature of Person Obtaining Informed Consent

Your signature below means that you have explained the optional part(s) to the research to the subject or subject representative and have answered any questions he/she has about the research.

___________________________ _______________________________________

Name of Person Obtaining Consent (Please Print) **Signature** **Date**

Signature of Person Giving Informed Consent

Signature of Subject

By signing below, you indicate that you have read the information written above and have indicated your choices for the optional part(s) of the research study.

___________________________ ____

Name of Subject (Please Print) **Signature of Subject** **Date**

16 Human Subjects/Informed Consent/Male

COMBINED INFORMED CONSENT AND
HIPAA AUTHORIZATION FORM
Penn State College of Medicine
The Milton S. Hershey Medical Center

Protocol Title: Improving Reproductive Fitness with Pretreatment with Lifestyle Modification in Obese Women with Unexplained Infertility: FIT-PLESE

Principal Investigator: Richard Legro, M.D.
Obstetrics & Gynecology Department, Division of Reproductive Endocrinology
500 University Drive, Room C3604, MC H103
Hershey, PA 17033

Emergency Contact: Weekdays: 8:00 a.m. to 5:00 p.m. (717) 531-8478.
After hours call (717) 531-8521 and ask for the Ob-Gyn doctor on 24-hour call.

Why am I being asked to volunteer?

You are being invited to participate in a research study. Your participation is voluntary which means you can choose whether or not you want to participate. If you choose not to participate, there will be no loss of benefits to which you are otherwise entitled. Before you can make your decision, you will need to know what the study is about, the possible risks and benefits of being in this study, and what you will have to do in this study. The research team is going to talk to you about the research study, and they will give you this consent form to read. You may also decide to discuss it with your family, friends, or family doctor. You may find some of the medical language difficult to understand. Please ask the study doctor and/or the research team about this form. If you decide to participate, you will be asked to sign this form.

What is the purpose of this research study?

We are asking you to be in this research because you and your partner/spouse have a diagnosis of unexplained infertility and are seeking pregnancy and your partner is overweight.

This research is being done to find out the effects of lifestyle modification in obese women with unexplained infertility. Phase 1 of the study is 16 weeks and includes one of two types of lifestyle modifications for your partner, either increased physical activity by increasing steps per day OR

a calorie restricted diet using meal replacements, use of a weight loss medication called Orlistat (Alli) and increased physical activity. Orlistat 60mg is a United States Food and Drug Administration (FDA) approved medication to be taken with meals and works by reducing the absorption of fat in a meal.

After completion of Phase 1 lifestyle modification, your partner/spouse will move to Phase 2 of the study. This will involve 3 cycles of infertility treatment involving ovarian stimulation with an oral medication (clomiphene citrate) followed by triggered ovulation using hCG injection and single intrauterine insemination. Clomiphene Citrate is approved by the FDA for the treatment of infertility. hCG, a combination medication of Follicle Stimulating Hormone and Luteinizing Hormone. It is an injectable medicine to be used to trigger ovulation and is also FDA approved.

How long will I be in the study?

If you agree to take part, it will take you about 3-4 months to complete this research study. This includes a screening visit for semen analysis and then up to 3 cycles of inseminations for your partner/spouse where you will need to provide semen specimens. You will be asked to return to the research site 4 times. Each visit will take as long as you need for collection of semen for analysis and insemination. Your partner would not be able to have the intrauterine insemination without these specimens.

How many subjects will be in the study?

Approximately 380 women and their partner/spouse will take part in this research study nationwide as part of the Reproductive Medicine Network (RMN).

What am I being asked to do?

At initial contact either by email, telephone, or in clinic, your partner/spouse will be screened with a brief eligibility questionnaire to determine if she qualifies to take part in this research. If she is determined to be eligible, you and your partner/spouse will be scheduled for a screening visit. This consent form will be reviewed by you and the study coordinator. The purpose of the study, all procedures involved, the risks, and the time commitment related to this study will be discussed. Once all questions have been answered, you will sign this consent form. A copy of this signed consent will be given to you for your records. Your partner/spouse will also sign a separate informed consent for participation.

Medical Assessment & Questionnaires

Your height, weight, vital signs (blood pressure and pulse) and neck, hip and abdominal circumference will be collected and your BMI will be calculated. You will be required to complete a medical history questionnaire. This questionnaire will ask you questions about your medical health, infertility history, and demographic information. You will be given 11 additional questionnaires to complete. A Genetic Risk questionnaire is completed to determine if you have an increased risk of having a baby with a genetic disorder. A Patient Health Questionnaire (PHQ-9) and the Short Form 12 (SF-12) will assess your daily activities. The Fertility Quality of Life Questionnaire (FertiQoL) will assess how your infertility affects your thoughts and feelings. The Epworth Sleepiness Scale (ESS)

and Stop-Bang Questionnaire will assess your quality of sleep. The International Index of Erectile Function Survey (IIEF), Psychosexual Daily Questionnaire (PDQ), and qADAM questionnaire will assess your sexual function. The Automated Self-Administered 24-hour Recall (ASA24) and Dietary History (DHQ-II) will be completed to assess your past and current dietary intake. You will be free to skip any questions that you would prefer not to answer.

Collection of Semen Sample

If you agree to take part in this research study, you will be asked to provide a semen sample if you have not given one within the past 12 months. The study coordinator will schedule you for an appointment in the andrology laboratory. Instructions will be given to you prior to the collection appointment. For this semen analysis test, it is important to not have sex or masturbate for 48 hours prior to collecting your samples.

The following are the instructions you will follow to collect your sample. There is a private room near the lab where the specimen can be collected.

1. Wash your hands and genitals in the normal way and dry thoroughly.
2. Use the cup provided by the study coordinator or the lab. Do not open the container until you are ready to produce the sample.
3. Collect the sample by masturbation, putting the entire specimen directly into the cup.
4. Do not use lubricants or condoms, as these are harmful to sperm.
5. Seal the cup immediately with the lid.
6. Write your name on the label of the cup. Fill out the bottom of the lab form. Return the specimen and form to the laboratory staff.

Once you have completed your collection, a semen analysis will be performed to determine the concentration of sperm, motility (moving) and morphology (appearance). Once your specimen has been tested, the study coordinator will be given your results. Your results will be reviewed with principal investigator of the study. You will be notified of your results. If there are more than 5 million motile (moving) sperm in the ejaculate, your partner/spouse will be eligible to participate in the main study. If the results of your semen analysis are abnormal, you will be provided with the information and referred to your physician for further follow-up. If the number of motile sperm in an ejaculate is less than 5 million, your partner/spouse will be ineligible to participate in the main study at this time. If you have previously had a semen analysis test done within the past 12 months, we will ask you to sign a medical release form so we can obtain your results.

Trigger injection & Insemination

You will be asked to assist in the giving of an injection of the hCG trigger medication to your partner/spouse. The proper technique of the injection will be taught to you and your partner/spouse by the study coordinator. You may decline to assist in the injections and remain in the study, if your partner/spouse is going to self-administer the injections.

As part of the inclusion criteria for participation in this study, inseminations (placing your sperm into your partner's/spouse's uterus) of your partner must take place at least once each cycle for up to 3 cycles. Both you and your partner must agree to this requirement to participate in the study. For the inseminations, the semen specimen will be washed to concentrate the sperm and remove other components of the ejaculate specimen. The sperm will then be used for the insemination of your partner/spouse.

Repository Semen, Blood and Urine Specimen Collection
If you consent to the optional part of the study, you will provide a urine sample and have approximately 2 tablespoons of blood drawn for future testing. No blood or urine will be collected if you have not consented to this option. A portion of your semen will also be collected and stored as part of the repository. These will only be collected if you have consented to the optional part of the study.

Saliva Collection for Cortisol Testing
If you have consented to the collection of saliva for cortisol testing, this will be collected at this time. Cortisol is a stress hormone. This will be done by removing a cotton swab from the package and placing the swab under your tongue for approximately 60-120 seconds. Once the time is complete, the swab is inserted into the tube and sealed for storage in a freezer.

What are my responsibilities if I take part in this research?

If you take part in this research, your major responsibilities will include:

- Providing a semen sample for screening and for each insemination cycle up to 3 cycles
- Assisting in injections of hCG trigger medication to your partner/spouse
- Completing medical and quality of life questionnaires
- Provide blood, urine and saliva for optional portion of the study if you have consented to this part of the study.

What are the possible risks or discomforts?

By taking part in this study, you may experience the following risks:

Risks of standard venipuncture:
A slight pinch or pin prick, discomfort, black and blue mark at the site of puncture, small blood clot, infection or bleeding at the site and fainting during the procedure.

Risks of loss of Confidentiality:
There is a risk of loss of confidentiality if your medical information or your identity are obtained by someone other than the investigators, but precautions will be taken to prevent this from happening.

What if new information becomes available about the study?

During the course of this study, we may find more information that could be important to you. This includes information that, once learned, might cause you to change your mind about being in the study. We will notify you as soon as possible if such information becomes available.

What are the possible benefits of the study?

There is no guarantee that you will benefit from this research. The possible benefits you may experience from this research study include:

- You will be provided with your semen analysis results. Any abnormalities will be assessed by the principal investigator and followed through by your physician. The results of your semen analysis will determine eligibility for your partner/spouse to participate in the main study.
- The study interventions and medications your partner/spouse is taking may help her to become pregnant.

What are the possible benefits to others?
The results of this research may guide the future of treatment of obesity for women with unexplained infertility by establishing effective lifestyle changes and weight loss prior to attempting pregnancy. This research may also help discover a better way to achieve pregnancy and a goal of a healthy pregnancy with live birth by the use of Clomiphene Citrate and intrauterine insemination for women with unexplained infertility.

What other choices do I have if I do not participate?

You do not have to take part in this study. Choosing not to participate will not have any effect on your clinical care.

The therapy offered in this research is available to you without taking part in this research study.

Will I be paid for being in this study?

You will not receive any payment or compensation for being in this research study.

Will I have to pay for anything?

The study will provide the following:

- The semen analysis will be provided by the Reproductive Medicine Network at no cost to you.
- You and/or your insurance company will not be charged for the cost of any tests or procedures that are required as part of the research and are within the standard of care (what is normally done) for your condition.
- The research-related tests and procedures that will be provided at no cost to you include: semen analysis, blood work and urine collection for RMN Biologic Repository and saliva collection.

For costs of medical services for care you would receive even if you were not in this research study:

- You and/or your insurance company will be responsible for the cost of routine medications, tests and procedures that you would receive even if you were not in this research that are not mentioned above as covered.
- You and/or your insurance company will be billed for the costs of these routine tests and procedures in the usual manner.
- You will be responsible for any co-payments, co-insurance and deductibles that are standard for your insurance coverage. You will be responsible for any charges not reimbursed by your insurance company.
- Some insurance companies will not pay for routine costs for people taking part in research studies. Before deciding to be in this research you should check with your insurance company to find out what they will pay for.

If you have any questions about costs and insurance, ask the research study doctor or a member of the research team about putting you in touch with a financial counselor to determine exactly what the deductible and co-pay will be for you; this is highly variable depending on your type of insurance."

Who is paying for this research study?

The institution and investigators are receiving a grant from the National Institute of Health to support this research.

What happens if I am injured from being in the study?

It is possible that you could develop complications or injuries as a result of being in this research study. If you experience a side effect or injury and emergency medical treatment is required, seek treatment immediately at any medical facility. If you experience a side effect or injury and you believe that emergency treatment is not necessary, you should contact the principal investigator listed on the first page of this consent form as soon as possible and he/she will arrange for medical treatment.

The Milton S. Hershey Medical Center (HMC)/Penn State College of Medicine (PSU) compensation for injury

- There are no plans for HMC/PSU to provide financial compensation or free medical treatment for research-related injury.
- If an injury occurs, medical treatment is available at the usual charge.
- Costs will be charged to your insurance carrier or to you.
- Some insurance companies may not cover costs associated with research injuries.
- If these costs are not covered by your insurance, they will be your responsibility.

When you sign this form you are not giving up any legal right to seek compensation for injury.

When is the Study over? Can I leave the Study before it ends?

This study is expected to end after all participants have completed all visits, and all information has been collected. This study may also be stopped at any time by your physician, the study Sponsor, or the Food and Drug Administration (FDA) without your consent because:

- The Primary Investigator feels it is necessary for your health or safety. Such an action would not require your consent, but you will be informed if such a decision is made and the reason for this decision.
- You have not followed study instructions.
- The Sponsor, the study Principal Investigator, or the Food and Drug Administration (FDA) has decided to stop the study.

If you decide to participate, you are free to leave the study at anytime. Withdrawal will not interfere with your future care.

If you stop being in the research, already collected data may not be removed from the study database. You will be asked whether the investigator can collect medical information from your routine medical care. If you agree, this data will be handled the same as research data. If you withdraw completely from the research study, no further information will be collected and your participation will end. You may discontinue taking part at any time without penalty or loss of benefits to which you are otherwise entitled.

Who can see or use my information? How will my personal information be protected?

Efforts will be made to limit the use and sharing of your personal research information. In our research files at HMC/PSU we will include these identifiers:

- Study identification number
- Name & Initials
- Address
- Phone number, cell phone number, work number
- Email address
- Social Security number
- Medical Record number
- Date of birth
- Visit date

A list that matches your name with your code number will be kept in a locked file.

Your records that are used in the research at HMC/PSU will include your study identification number, your initials, and visit date and will be kept in a secured area in a locked file cabinet. Your samples collected for research purposes will be labeled with your study identification number, initials, and visit date and will be stored in a -80 degree freezer.

For research records sent to the Data Coordination Center at Yale University, you will be identified by your study identification number and study visit date. Your blood specimens will not identify you and you cannot be linked to your specimen. The list that matches your name with your code number will be kept in a secured area in a locked file cabinet at HMC/PSU.

For specimens sent to the RMN Biologic Repository and the saliva collection, you will not be identified and you cannot be linked to your sample. The sample will be labeled with a bar code label. The sample will be stored in the Repository until exhausted by the RMN.

To help protect your privacy, a Certificate of Confidentiality will be obtained from the federal government. This Certificate means that the researchers cannot be forced (for example by court subpoena) to share information that may identify you in any federal, state, or local civil, criminal, administrative, legislative, or other proceedings. The researchers will use the Certificate to resist any demands for information that would identify you, except as explained below.

The Certificate cannot be used to resist a demand for information from personnel of the U.S. government that is used for checking or evaluation of federally funded projects or for information that must be disclosed in order to meet the requirements of the federal Food and Drug Administration (FDA).

The Certificate, however, does not prevent you or a member of your family from voluntarily releasing information about yourself or your involvement in this research. If an insurer, employer, or other person obtains your written consent to receive research information, then the researchers may not use the Certificate to withhold that information. This means that you and your family should actively protect your own privacy.

In the event of any publication or presentation resulting from the research, no personally identifiable information will be shared.

A copy of this consent form will go in to your medical record. This will allow the doctors caring for you to know what study medications or tests you may be receiving as a part of the study and know how to take care of you if you have other health problems or needs during the study.

We will do our best to make sure that the personal information obtained during the course of this research study will be kept private. However, we cannot guarantee total privacy. Your personal information may be given out if required by law. If information from this study is published or presented at scientific meetings, your name and other personal information will not be used. If this study is being overseen by the Food and Drug Administration (FDA), they may review your research records.
If you test positive for reportable infectious diseases, by law we have to report the infection to the PA Department of Health. We would report your name, gender, racial/ethnic background, and the month and year you were born.

Electronic Medical Records and Research Results

Results of some of the research-related tests including but not limited to your screening semen analysis and semen analysis for insemination information will be kept in your electronic medical record.

What is an Electronic Medical Record?
An Electronic Medical Record (EMR) is an electronic version of the record of your care within a health system. An EMR is simply a computerized version of a paper medical record.

If you are receiving care or have received care within the HMC (outpatient or inpatient) and are participating in a HMC/PSU research study, results of research-related procedures (i.e. laboratory tests, imaging studies and clinical procedures) may be placed in your existing EMR maintained by HMC.

If you have never received care within HMC and are participating in a HMC/PSU research study that uses HMC services, an EMR will be created for you for the purpose of maintaining any results of procedures performed as part of this research study. The creation of this EMR is required for your participation in this study. In order to create your EMR, the study team will need to obtain basic information about you that would be similar to the information you would provide the first time you visit a hospital or medical facility (i.e. your name, the name of your primary doctor, the type of insurance you have). Results of research procedures performed as part of your participation in the study (i.e. laboratory tests, imaging studies and clinical procedures) may be placed in this EMR.

Once placed in your EMR, these results are accessible to appropriate HMC/PSU workforce members that are not part of the research team. Information within your EMR may also be shared with others who are determined by HMC/PSU to be appropriate to have access to your EMR (e.g. health insurance company, disability provider, etc.).

What information about me may be collected, used or shared with others?

- Name
- Address
- Phone number, cell phone number, work number
- Email address
- Social Security number
- Medical Record number
- Date of birth
- Past, present, and future medical records
- New health information from tests, procedures, visits, interviews, or forms filled out as part of this research study

Why is my information being used?

Your information is used by the research team to contact you during the study. Your information and results of tests and procedures are used to:

- do the research
- oversee the research
- see if the research was done right.

Who may use and share information about me?

The following people/groups may see, use, and share your identifiable health information:

- HMC/PSU research staff involved in this study
- The University of Pennsylvania Institutional Review Board, a group of people who review the research study to protect subjects' rights and welfare
- The University of Pennsylvania Human Subjects Protection Office
- The HMC/PSU Human Subjects Protection Office
- The HMC/PSU Research Quality Assurance Office
- Non-research staff within HMC/PSU who need this information to do their jobs (such as for treatment, payment (billing), or health care operations)
- Federal and state agencies (such as the U.S. Food and Drug Administration, the Office for Human Research Protections, the Department of Health and Human Services, the National Institutes of Health, and other U.S. or foreign government bodies that oversee or review research)
- Other researchers and medical centers that are part of this study and their IRBs
- The Data Coordinating Center at Yale University
- Reproductive Medicine Network of the Eunice Kennedy Shriver National Institute of Child Health and Human Development
- A group that oversees the data (study information) and safety of this research
- Organizations that provide independent accreditation and oversight of hospitals and research
- Public health and safety authorities (for example, if we learn information that could mean harm to you or others, we may need to report this, as required by law)

These groups may also review and/or copy your original PSU/HMC records while looking at the results of the research study. It is possible that some of the other people/groups who receive your health information may not be required by Federal privacy laws to protect your information. We share your information only when we must, and we ask anyone who receives it from us to protect your privacy

You have the right to see and get a copy of your health information that is used or shared for treatment or for payment. However, you may not be allowed to see or copy certain health

information that is a part of this research study. This is only for the period of the study. You will be allowed to see that information when the entire research study is complete.

How long may the HMC/PSU use or disclose my personal health information?

Your authorization for use of your personal health information for this specific study does not expire.

Your information may be held in a research database. However, HMC/PSU may not re-use or re-disclose information collected in this study for a purpose other than this study unless:

- You have given written authorization
- The University of Pennsylvania's Institutional Review Board grants permission
- As permitted by law

Can I change my mind about giving permission for use of my information?

You have the right to withdraw your permission for us to use or share your health information for this research study. If you want to withdraw your permission, you must notify the person in charge of this research study in writing using the address on the front of this form. Once permission is withdrawn, you cannot continue to take part in the study.

If you withdraw your permission, we will stop collecting health information about you for this study; we may continue to use and share your health information that we already have if it is necessary for safety and scientific soundness of the research study; and we will not be able to take back information that has already been used or shared with others.

What if I decide not to give permission to use and give out my health information?

You have the right to refuse to sign this form that allows us to use and share your health information for research; however, if you don't sign it, you will not be able to take part in this research study.

Who can I call with questions, complaints or if I'm concerned about my rights as a research subject?

If you have questions, concerns or complaints regarding your participation in this research study or if you have any questions about your rights as a research subject, you should speak with the Principal Investigator listed on page one of this form. If a member of the research team cannot be reached or you want to talk to someone other than those working on the study, you may contact the Office of Regulatory Affairs at the University of Pennsylvania by calling (215) 898-2614.

When you sign this form, you are agreeing to take part in this research study. This means that you have read the consent form, your questions have been answered, and you have decided to volunteer. Your signature also means that you are permitting

HMC/PSU to use your personal health information collected about you for research purposes within our institution. You are also allowing HMC/PSU to disclose that personal health information to outside organizations or people involved with the operations of this study.

A copy of this consent form will be given to you.

__________________________ _______________________________________

Name of Subject (Please Print) **Signature of Subject** **Date**

____

Name of Person Obtaining Consent (Please Print) **Signature** **Date**

Optional part(s) of the study

In addition to the main part of the research study, there is another part of the research. You can be in the main part of the research without agreeing to be in this optional part.

Optional Blood and Urine for RMN Biologic Repository

As part of an optional study, we are obtaining a sample of your blood to be stored in a Biologic Repository by the Reproductive Medicine Network for future use. If you agree, a urine sample and approximately 2 tablespoons of your blood will be collected at the randomization visit and shipped to the RMN Biologic Repository (a central location) and stored for a minimum of 5 years, and perhaps into the future. Your sample will be tested for DNA and to measure other substances in your blood. If you agree to allow us to collect and store a blood sample from you for future use in the repository, your sample will be labeled with a bar code label and unique identifier. If you consent to the collection of your blood for the repository, it will be kept indefinitely or until the sample is exhausted by the Reproductive Medicine Network. Once your blood has left the HMC/PSU, there is no way to have it returned or withdrawn for testing. You will not be able to withdraw your permission for the use of your blood sample for future use as this time. No blood or urine will be collected if you have not agreed to this option.

- **These future studies may be helpful in understanding testicular physiology and unexplained infertility.**
- It is unlikely that these studies will have a direct benefit to you.
- The results of these tests will not have an effect on your care.
- Neither your doctor nor you will receive results of these tests, nor will the results be put in your health record.

- It is possible that your blood/urine might be used to develop products or tests that could be patented and licensed. There are no plans to provide financial compensation to you should this occur. If you have any questions, you should contact a member of the research team.

You should ***initial*** below to indicate your preference for the collection of your blood and urine sample for the RMN Biologic Repository:

______ I **give my permission** for my blood and urine sample to be collected and sent to the repository for future testing.

______ I **decline my permission** for my blood and urine sample to be collected and sent to the repository for future testing.

Optional Study for Cortisol Saliva Collection

As part of an optional study, we would like to obtain a sample of your saliva for cortisol testing. Cortisol is a stress hormone found in saliva. If you agree, a sample of your saliva will be collected by placing a cotton swab under your tongue for 60-120 seconds and then placed in a tube and sealed for storage in a freezer until testing. If you agree to allow us to collect your saliva, your sample will be labeled with a bar code label and a unique identifier. These samples will be stored in Dr. Legro's locked laboratory, Room C3611 until shipment to the laboratory analyzing the saliva. The laboratory will be designated by the RMN. Once the saliva has been analyzed, and if there is leftover sample, your sample will be shipped to the RMN Biologic Repository where it will be stored indefinitely or until the sample is exhausted by the RMN. These samples can be used for other research in the future after the study is over and also be shared with other investigators who collaborate with the RMN. Once your saliva has left the Hershey Medical Center, there is no way to have it returned or withdrawn for testing. You will not be able to withdraw your permission for the use of your saliva for future use as this time.

- The testing of saliva may provide additional information that will be helpful in understanding the factors of stress in infertility.
- It is unlikely that these studies will have a direct benefit to you.
- The results of these tests will not have an effect on your care.
- Neither your doctor nor you will receive results of these tests, nor will the results be put in your health record.

You should ***initial*** below to indicate your preference for the collection of your saliva sample:

________ I **give my permission** for my saliva sample to be collected and tested under the study.

________ I **decline my permission** for my saliva to be collected and tested under the study.

Optional Storage of Semen for Future Use in Research

As part of an optional study, we want to obtain a sample of your semen to be stored by the RMN for future use in the RMN Biologic Repository. If you agree, your semen sample will be collected at the screening visit after informed consent. If you are collecting a semen sample at the screening visit for analysis as part of the screening requirement, a portion of that sample can be used for this optional collection. A separate collection would not be needed in this instance. If you have a semen analysis on file that meets the eligibility requirements, and do not need to collect for eligibility, then a semen sample would need to be collected for this optional part of the study. The sample will be stored at the HMC/PSU until shipment to the RMN Biologic Repository. Your sample will be stored for a minimum of 5 years, and perhaps further into the future. Your sample will be tested for the chemicals that make up all of your genes and contain your genetic information. These samples can be used for other research in the future after this study is over. Your sample will not be labeled with any of your personal information, such as your name or a code number. The sample for the repository is labeled with a bar code and cannot be directly linked back to you. They will be available for use in future research studies indefinitely and cannot be removed due to the inability to identify them.

- **These future studies may be helpful in understanding testicular physiology and unexplained infertility.**
- **It is unlikely that these studies will have a direct benefit to you.**
- **The results of these tests will not have an effect on your care.**
- **Neither your doctor nor you will receive results of these future research tests, nor will the results be put in your health record.**
- **It is possible that your semen sample might be used to develop products or tests that could be patented and licensed. There are no plans to provide financial compensation to you should this occur. If you have any questions, you should contact a member of the research team.**

You should ***initial*** below to indicate your preference for the collection of your semen sample for the repository:

________ I **give my permission** for my semen sample to be collected and sent to the repository for future testing.

________ I **decline my permission** for my semen sample to be collected and sent to the repository for future testing.

Signature of Person Obtaining Informed Consent

Your signature below means that you have explained the optional part(s) to the research to the subject or subject representative and have answered any questions he/she has about the research.

___________________________ _______________________________________

Name of Person Obtaining Consent (Please Print) **Signature** **Date**

Signature of Person Giving Informed Consent

Signature of Subject

By signing below, you indicate that you have read the information written above and have indicated your choices for the optional part(s) of the research study.

_________________________ ____

Name of Subject (Please Print) **Signature of Subject** **Date**

17 Appendix A: Risk Factors for Genetic Disorders

The following questions are designed to determine if you have an increased risk of having a baby with a genetic disorder. Sometimes genetic disorders occur even when there is no history of problems in your family. The following questions are related to you and your immediate family including mother, father, sister, brother, child, or grandparent. If you answer "yes" to any of the following questions and would like more information, please discuss this with your physician.

Questions	Yes	No	Unknown	N/A
1. If you conceive during this study, will you be 35 or older when your baby is due?				
2. If you are of Mediterranean or Asian descent, does anyone in your family have thalassemia? (a blood disorder that causes anemia)				
3. Is there a family history of neural tube defects?				
4. Have you had a child with a neural tube defect?				
5. Does anyone in your family have a history of congenital heart defects? (heart problems when they were born)				
6. Does anyone in your family have Down Syndrome?				
7. Have you ever had a child with Down Syndrome?				
8. If you are of Eastern European Jewish or French Canadian descent, does anyone in your family have a history of Tay-Sachs disease? (a disorder of the central nervous system)				
9. If you are of Eastern European Jewish descent, does anyone in your family have a history of Canavan disease? (a disorder of the central nervous system that leads to blindness and muscle weakness)				
10. If you are of African American descent, is there any history of sickle cell trait? (a type of anemia)				
11. If you are of African American descent, is there any history of sickle cell disease? (a type of anemia)				
12. Do you or anyone in your family have a history of hemophilia? (a disorder that causes bleeding)				
13. Do you or anyone in your family have a history of muscular dystrophy? (a neuromuscular disorder)				
14. Do you or anyone in your family have a history of cystic fibrosis? (a disorder that causes thick mucous in the lungs and other organs)				
15. Do you or anyone in your family have a history of Huntington's disease? (a degenerative brain disease)				
16. Do you or anyone in your family have a history of alpha-1 antitrypsin deficiency? (lack of a liver protein)				
17. Do you or anyone in your family have a history of mental retardation?				

17a. If yes to Question #17, was the person ever tested for fragile-x syndrome? (a condition which can cause mild to severe mental retardation)

18. Do you or anyone in your family have a history of any other genetic disease, chromosomal disorder or birth defect?

19. Do you have any metabolic disorders such as diabetes or phenylketonuria (PKU)?
(a disorder which prevents the normal use of protein foods)

20. Have you ever had 3 or more miscarriages in a row?

21. Have you ever had a baby that was stillborn?

Partner Family History

The following questions are designed to determine if you have an increased risk of having a baby with a genetic disorder. Sometimes genetic disorders occur even when there is no history of problems in your family. The following questions are related to you and your immediate family including mother, father, sister, brother, child, or grandparent. If you answer "yes" to any of the following questions and would like more information, please discuss this with your physician.

Questions	Yes	No	Unknown	N/A
1. If you are of Mediterranean or Asian descent, does anyone in your family have thalassemia? (a blood disorder that causes anemia)				
2. Is there a family history of neural tube defects?				
3. Have you had a child with a neural tube defect?				
4. Does anyone in your family have a history of congenital heart defects? (heart problems when they were born)				
5. Does anyone in your family have Down Syndrome?				
6. Have you ever had a child with Down Syndrome?				
7. If you are of Eastern European Jewish or French Canadian descent, does anyone in your family have a history of Tay-Sachs disease? (a disorder of the central nervous system)				
8. If you are of Eastern European Jewish descent, does anyone in your family have a history of Canavan disease? (a disorder of the central nervous system that leads to blindness and muscle weakness)				
9. If you are of African American descent, is there any history of sickle cell trait? (a type of anemia)				
10. If you are of African American descent, is there any history of sickle cell disease? (a type of anemia)				
11. Do you or anyone in your family have a history of hemophilia? (a disorder that causes bleeding)				
12. Do you or anyone in your family have a history of muscular dystrophy? (a neuromuscular disorder)				
13. Do you or anyone in your family have a history of cystic fibrosis? (a disorder that causes thick mucous in the lungs and other organs)				
14. Do you or anyone in your family have a history of Huntington's disease? (a degenerative brain disease)				
15. Do you or anyone in your family have a history of alpha-1 antitrypsin deficiency? (lack of a liver protein)				
16. Do you or anyone in your family have a history of mental retardation?				
16a. If yes to Question #17, was the person ever tested for fragile-x syndrome? (a condition which can cause mild to severe mental retardation)				

17. Do you or anyone in your family have a history of any other genetic disease, chromosomal disorder or birth defect?
18. Do you have any metabolic disorders such as diabetes or phenylketonuria (PKU)?
(a disorder which prevents the normal use of protein foods)

18 Appendix B: List of common medications excluded or requiring wash-out period

Excluded Medications

A patient will be excluded from study if they are taking any medication that should not be discontinued and this medication would affect reproductive function or metabolism, or would interact with either study medication.

Hormonal Medications Requiring 1 month wash-out:

Progestins (Oral or Cyclic)

- medroxyprogesterone acetate (Provera, Cycrin, Amen, Curretab)
- megestrol (Megase)
- norethindrone (Aygestin)
- progesterone gel (Crinone)
- Micronized progesterone (Prometrium)

Hormonal Medications Requiring 2 month Wash-Out Period:

GnRH Agonists/Antagonists

- Leuprolide (Lupron)
- nafarelin (Synarel)
- buserelin
- gosarelin (Zoladex)
- ganarelix (Antagon)
- cetrorelix (Cetrotide)

Gonadotropins

- Pergonal
- Repronex
- Follistim
- Gonal-F
- Fertinex
- Metrodin

Injectable Contraceptives

- medroxyprogesterone acetate (Depo Provera)

Oral Contraceptives

- Any Brand

Continuous Progestins (Not including cyclic)

- Any Brand

Other Medications Requiring 2 month Wash-out Period:

Somatostatin

- octreotide (Sandostatin)
- lanreaotide

Anti-Acne

- isotretinoin (Accutane)

Anti-androgens

- cyproterone (Cyprostat)
- spironolactone (Aldactone)
- flutamide (Eulexin)
- finasteride (Proscar, Propecia)

Anti-diabetic

Insulin

Gastric inhibitors

acarbose (Precose)

Thiazolidinediones
Rosiglitazone (Avandia)
Pioglitazone (Actos)

Sulfonylureas/Beta cell stimulators
acetahexamide (Dymelor)
chlorpropamide (Diabinese)
tolazamide (Tolinase)
tolbutamide (Orinase)
glimepiride (Amaryl)
glipizide (Glucotrol)
glyburide (DiaBeta Micronase)

Biguanides
metformin (Glucophage)

Incretins : GLP-1 Analogues/DPP-IV inhibitors
sitagliptin (Januvia)
vidagliptin (Glavus)
exenatide (Byetta)
liraglutide (Victoza)

Amylin Analogue
Pramlintide (Symlin)

Anti-obesity
diazoxide (Proglycem)
orlistat (Xenical
diethylpropion (Tenuate)
phendimetrazine (Bontril)
phentermine (Adipex-P, Fastin, Ionamin)
lorcarserin (Belviq)
Phentermine/Topiramate (Osymia)

Other drugs with weight loss as a side effect
Buproprion (Wellbutrin, Zyban)
Topiramate (Topamax)
Desfenlafaxine (Pristiq)

Medications with potential longer washouts (Contact DCC)
Contraceptive Implants
Norplant (levonorgestrel implants)
Implanon
Previous clomiphene and letrozole use requires a two-month washout

19 Appendix C: SF 12 Health Survey

Instructions: This survey asks for your views about your health. This information will help keep track of how you feel and how well you are able to do your usual activities. Thank you for completing this survey!
1. In general, would you say your health is: (Please check <u>one</u> box.)<br><br>☐1 Excellent ☐2 Very Good ☐3 Good ☐4 Fair ☐5 Poor

2. The following questions are about activities you might do during a typical day. Does **<u>your health now</u>** **<u>limit you</u>** in these activities? If so, how much? (Please circle one number on each line)

**<u>Activities</u>**	**Yes, Limited A Lot**	**Yes, Limited A Little**	**No, not limited at all**
2(a) <u>Moderate activities</u>, such as moving a table, pushing a vacuum cleaner, bowling, or playing golf	☐1	☐2	☐3
2(b) Climbing <u>several</u> flights of stairs	☐1	☐2	☐3

3. During the **<u>past 4 weeks</u>**, how much of the time have you had any of the following problems with your work or other regular daily activities **<u>as a result of your physical health</u>**?

**(Please circle one number on each line)**	**All of the Time**	**Most of the Time**	**Some of the Time**	**A Little of the Time**	**None of the time**
3(a) Accomplised less than you would like?	☐1	☐2	☐3	☐4	☐5
3(b) Were limited in the kind of work or other activities?	☐1	☐2	☐3	☐4	☐5

4. During the **<u>past 4 weeks</u>**, how much of the time have you had any of the following problems with your work or other regular daily activities **<u>as a result of any emotional problems</u>** (such as feeling depressed or anxious)?

**(Please circle one number on each line)**	**All of the Time**	**Most of the Time**	**Some of the Time**	**A Little of the Time**	**None of the time**
4(a) Accomplised less than you would like?	☐1	☐2	☐3	☐4	☐5
4(b) Did work or other activities less carefully than usual?	☐1	☐2	☐3	☐4	☐5

5. During the **<u>past 4 weeks</u>**, how much did **<u>pain</u>** interfere with your normal work (including both work outside the home and housework)? (Please check one box)

☐1 Not at all ☐2 A little bit ☐3 Moderately ☐4 Quite a bit
☐5 Extremely

6. These questions are about how you feel and how things have been with you **<u>during the past 4 weeks</u>**. For each question, please give the one answer that comes closest to the way you have been feeling. How much of the time **<u>during the past 4 weeks</u>**…

(Please circle one number on each line)	All of the Time	Most of the Time	Some of the Time	A Little of the Time	None of the time
6(a) Have you felt calm and peaceful?	☐1	☐2	☐3	☐4	☐5
6(b) Did you have a lot of energy?	☐1	☐2	☐3	☐4	☐5
6(c) Have you felt downhearted and depressed?	☐1	☐2	☐3	☐4	☐5

7. During the **past 4 weeks**, how much of the time has your **physical health or emotional problems** interfered with your social activities (like visiting with friends, relatives etc)? (Please check one box)

☐1 All of the time ☐3 Most of the time ☐5 Some of the time
☐2 A little of the time ☐4 None of the time

Appendix D: Female Sexual Function Index

Subject Identifier ______________________ Date ______________

INSTRUCTIONS: These questions ask about your sexual feelings and responses during the past 4 weeks. Please answer the following questions as honestly and clearly as possible. Your responses will be kept completely confidential. In answering these questions the following definitions apply:

Sexual activity can include caressing, foreplay, masturbation and vaginal intercourse.

Sexual intercourse is defined as penile penetration (entry) of the vagina.

Sexual stimulation includes situations like foreplay with a partner, self-stimulation (masturbation), or sexual fantasy.

CHECK ONLY ONE BOX PER QUESTION.

Sexual desire or interest is a feeling that includes wanting to have a sexual experience, feeling receptive to a partner's sexual initiation, and thinking or fantasizing about having sex.

1. Over the past 4 weeks, how **often** did you feel sexual desire or interest?

- ☐ Almost always or always
- ☐ Most times (more than half the time)
- ☐ Sometimes (about half the time)
- ☐ A few times (less than half the time)
- ☐ Almost never or never

2. Over the past 4 weeks, how would you rate your **level** (degree) of sexual desire or interest?

- ☐ Very high
- ☐ High
- ☐ Moderate
- ☐ Low
- ☐ Very low or none at all

Page 1 (of 5)

Sexual arousal is a feeling that includes both physical and mental aspects of sexual excitement. It may include feelings of warmth or tingling in the genitals, lubrication (wetness), or muscle contractions.

3. Over the past 4 weeks, how **often** did you feel sexually aroused ("turned on") during sexual activity or intercourse?

   - ☐ No sexual activity
   - ☐ Almost always or always
   - ☐ Most times (more than half the time)
   - ☐ Sometimes (about half the time)
   - ☐ A few times (less than half the time)
   - ☐ Almost never or never

4. Over the past 4 weeks, how would you rate your **level** of sexual arousal ("turn on") during sexual activity or intercourse?

   - ☐ No sexual activity
   - ☐ Very high
   - ☐ High
   - ☐ Moderate
   - ☐ Low
   - ☐ Very low or none at all

5. Over the past 4 weeks, how **confident** were you about becoming sexually aroused during sexual activity or intercourse?

   - ☐ No sexual activity
   - ☐ Very high confidence
   - ☐ High confidence
   - ☐ Moderate confidence
   - ☐ Low confidence
   - ☐ Very low or no confidence

6. Over the past 4 weeks, how **often** have you been satisfied with your arousal (excitement) during sexual activity or intercourse?

   - ☐ No sexual activity
   - ☐ Almost always or always
   - ☐ Most times (more than half the time)
   - ☐ Sometimes (about half the time)
   - ☐ A few times (less than half the time)
   - ☐ Almost never or never

7. Over the past 4 weeks, how **often** did you become lubricated ("wet") during sexual activity or intercourse?

- ☐ No sexual activity
- ☐ Almost always or always
- ☐ Most times (more than half the time)
- ☐ Sometimes (about half the time)
- ☐ A few times (less than half the time)
- ☐ Almost never or never

8. Over the past 4 weeks, how **difficult** was it to become lubricated ("wet") during sexual activity or intercourse?

- ☐ No sexual activity
- ☐ Extremely difficult or impossible
- ☐ Very difficult
- ☐ Difficult
- ☐ Slightly difficult
- ☐ Not difficult

9. Over the past 4 weeks, how often did you **maintain** your lubrication ("wetness") until completion of sexual activity or intercourse?

- ☐ No sexual activity
- ☐ Almost always or always
- ☐ Most times (more than half the time)
- ☐ Sometimes (about half the time)
- ☐ A few times (less than half the time)
- ☐ Almost never or never

10. Over the past 4 weeks, how **difficult** was it to maintain your lubrication ("wetness") until completion of sexual activity or intercourse?

- ☐ No sexual activity
- ☐ Extremely difficult or impossible
- ☐ Very difficult
- ☐ Difficult
- ☐ Slightly difficult
- ☐ Not difficult

11. Over the past 4 weeks, when you had sexual stimulation or intercourse, how **often** did you reach orgasm (climax)?

- ☐ No sexual activity
- ☐ Almost always or always
- ☐ Most times (more than half the time)
- ☐ Sometimes (about half the time)
- ☐ A few times (less than half the time)
- ☐ Almost never or never

12. Over the past 4 weeks, when you had sexual stimulation or intercourse, how **difficult** was it for you to reach orgasm (climax)?

- ☐ No sexual activity
- ☐ Extremely difficult or impossible
- ☐ Very difficult
- ☐ Difficult
- ☐ Slightly difficult
- ☐ Not difficult

13. Over the past 4 weeks, how **satisfied** were you with your ability to reach orgasm (climax) during sexual activity or intercourse?

- ☐ No sexual activity
- ☐ Very satisfied
- ☐ Moderately satisfied
- ☐ About equally satisfied and dissatisfied
- ☐ Moderately dissatisfied
- ☐ Very dissatisfied

14. Over the past 4 weeks, how **satisfied** have you been with the amount of emotional closeness during sexual activity between you and your partner?

- ☐ No sexual activity
- ☐ Very satisfied
- ☐ Moderately satisfied
- ☐ About equally satisfied and dissatisfied
- ☐ Moderately dissatisfied
- ☐ Very dissatisfied

15. Over the past 4 weeks, how **satisfied** have you been with your sexual relationship with your partner?

- ☐ Very satisfied
- ☐ Moderately satisfied
- ☐ About equally satisfied and dissatisfied
- ☐ Moderately dissatisfied
- ☐ Very dissatisfied

16. Over the past 4 weeks, how **satisfied** have you been with your overall sexual life?

- ☐ Very satisfied
- ☐ Moderately satisfied
- ☐ About equally satisfied and dissatisfied
- ☐ Moderately dissatisfied
- ☐ Very dissatisfied

17. Over the past 4 weeks, how **often** did you experience discomfort or pain <u>during</u> vaginal penetration?

- ☐ Did not attempt intercourse
- ☐ Almost always or always
- ☐ Most times (more than half the time)
- ☐ Sometimes (about half the time)
- ☐ A few times (less than half the time)
- ☐ Almost never or never

18. Over the past 4 weeks, how **often** did you experience discomfort or pain <u>following</u> vaginal penetration?

- ☐ Did not attempt intercourse
- ☐ Almost always or always
- ☐ Most times (more than half the time)
- ☐ Sometimes (about half the time)
- ☐ A few times (less than half the time)
- ☐ Almost never or never

19. Over the past 4 weeks, how would you rate your **level** (degree) of discomfort or pain during or following vaginal penetration?

- ☐ Did not attempt intercourse
- ☐ Very high
- ☐ High
- ☐ Moderate
- ☐ Low
- ☐ Very low or none at all

Thank you for completing this questionnaire

Copyright ©2000 All Rights Reserved

20 Appendix E: PHQ-9

PATIENT HEALTH QUESTIONNAIRE (PHQ-9)

NAME:______________________________ DATE:______________

Over the last *2 weeks*, how often have you been bothered by any of the following problems? *(use "✓" to indicate your answer)*	Not at all	Several days	More than half the days	Nearly every day
**1.** Little interest or pleasure in doing things	0	1	2	3
**2.** Feeling down, depressed, or hopeless	0	1	2	3
**3.** Trouble falling or staying asleep, or sleeping too much	0	1	2	3
**4.** Feeling tired or having little energy	0	1	2	3
**5.** Poor appetite or overeating	0	1	2	3
**6.** Feeling bad about yourself—or that you are a failure or have let yourself or your family down	0	1	2	3
**7.** Trouble concentrating on things, such as reading the newspaper or watching television	0	1	2	3
**8.** Moving or speaking so slowly that other people could have noticed. Or the opposite —being so figety or restless that you have been moving around a lot more than usual	0	1	2	3
**9.** Thoughts that you would be better off dead, or of hurting yourself	0	1	2	3
	add columns		+	+
(Healthcare professional: For interpretation of TOTAL, please refer to accompanying scoring card).	TOTAL:			

**10.** If you checked off *any problems*, how *difficult* have these problems made it for you to do your work, take care of things at home, or get along with other people?	Not difficult at all ________
	Somewhat difficult ________
	Very difficult ________
	Extremely difficult ________

PHQ-9 Patient Depression Questionnaire

For initial diagnosis:

1. Patient completes PHQ-9 Quick Depression Assessment.
2. If there are at least 4 ✓s in the shaded section (including Questions #1 and #2), consider a depressive disorder. Add score to determine severity.

Consider Major Depressive Disorder

- if there are at least 5 ✓s in the shaded section (one of which corresponds to Question #1 or #2)

Consider Other Depressive Disorder

- if there are 2-4 ✓s in the shaded section (one of which corresponds to Question #1 or #2)

Note: Since the questionnaire relies on patient self-report, all responses should be verified by the clinician, and a definitive diagnosis is made on clinical grounds taking into account how well the patient understood the questionnaire, as well as other relevant information from the patient.
Diagnoses of Major Depressive Disorder or Other Depressive Disorder also require impairment of social, occupational, or other important areas of functioning (Question #10) and ruling out normal bereavement, a history of a Manic Episode (Bipolar Disorder), and a physical disorder, medication, or other drug as the biological cause of the depressive symptoms.

To monitor severity over time for newly diagnosed patients or patients in current treatment for depression:

1. Patients may complete questionnaires at baseline and at regular intervals (eg, every 2 weeks) at home and bring them in at their next appointment for scoring or they may complete the questionnaire during each scheduled appointment.
2. Add up ✓s by column. For every ✓: Several days = 1 More than half the days = 2 Nearly every day = 3
3. Add together column scores to get a TOTAL score.
4. Refer to the accompanying **PHQ-9 Scoring Box** to interpret the TOTAL score.
5. Results may be included in patient files to assist you in setting up a treatment goal, determining degree of response, as well as guiding treatment intervention.

Scoring: add up all checked boxes on PHQ-9

For every ✓ Not at all = 0; Several days = 1;
More than half the days = 2; Nearly every day = 3

Interpretation of Total Score

Total Score	Depression Severity
1-4	Minimal depression
5-9	Mild depression
10-14	Moderate depression
15-19	Moderately severe depression
20-27	Severe depression

21 Appendix F: Female Sexual Distress Scale (FSDS - Revised 2005)

INSTRUCTIONS
Below is a list of feelings and problems that women sometimes have concerning their sexuality. Please read each item carefully, and circle the number that best describes HOW OFTEN THAT PROBLEM HAS BOTHERED YOU OR CAUSED YOU DISTRESS DURING THE PAST 7 DAYS INCLUDING TODAY. Circle only one number for each item, and take care not to skip any items. If you change your mind, erase your first circle carefully. Read the example before beginning, and if you have any questions please ask about them.
Example: How often did you feel: **Personal responsibility for your sexual problems.**

NEVER	RARELY	OCCASIONALLY	FREQUENTLY	ALWAYS
0	1	2	3	4

**HOW OFTEN DID YOU FEEL:**					
1. Distressed about your sex life	0	1	2	3	4
2. Unhappy about your sexual relationship	0	1	2	3	4
3. Guilty about sexual difficulties	0	1	2	3	4
4. Frustrated by your sexual problems	0	1	2	3	4
5. Stressed about sex	0	1	2	3	4
6. Inferior because of sexual problems	0	1	2	3	4
7. Worried about sex	0	1	2	3	4
8. Sexually inadequate	0	1	2	3	4
9. Regrets about your sexuality	0	1	2	3	4
10. Embarrassed about sexual problems	0	1	2	3	4
11. Dissatisfied with your sex life	0	1	2	3	4
12. Angry about your sex life	0	1	2	3	4
13. Bothered by low sexual desire	0	1	2	3	4

Copyright 2000 by American Foundation for Urological Disease Inc.

22 Appendix G: FertiQoL

FertiQoL International

Fertility Quality of Life Questionnaire (2008)

For each question, kindly check (tick the box) for the response that most closely reflects how you think and feel. Relate your answers to your current thoughts and feelings. Some questions may relate to your private life, but they are necessary to adequately measure all aspects of your life.

Please complete the items marked with an asterisk (*) only if you have a partner.

	For each question, check the response that is closest to your current thoughts and feelings	Very Poor	Poor	Nor good nor poor	Good	Very Good
A	How would you rate your health?	☐	☐	☐	☐	☐
	For each question, check the response that is closest to your current thoughts and feelings	Very Dissatisfied	Dissatisfied	Neither Satisfied Nor Dissatisfied	Satisfied	Very Satisfied
B	Are you satisfied with your quality of life?	☐	☐	☐	☐	☐
	For each question, check the response that is closest to your current thoughts and feelings	Completely	A Great Deal	Moderately	Not Much	Not At All
Q1	Are your attention and concentration impaired by thoughts of infertility?	☐	☐	☐	☐	☐
Q2	Do you think you cannot move ahead with other life goals and plans because of fertility problems?	☐	☐	☐	☐	☐
Q3	Do you feel drained or worn out because of fertility problems?	☐	☐	☐	☐	☐
Q4	Do you feel able to cope with your fertility problems?	☐	☐	☐	☐	☐
	For each question, check the response that is closest to your current thoughts and feelings	Very Dissatisfied	Dissatisfied	Neither Satisfied Nor Dissatisfied	Satisfied	Very Satisfied
Q5	Are you satisfied with the support you receive from friends with regard to your fertility problems?	☐	☐	☐	☐	☐
*Q6	Are you satisfied with your sexual relationship even though you have fertility problems?	☐	☐	☐	☐	☐
	For each question, check the response that is closest to your current thoughts and feelings	Always	Very Often	Quite Often	Seldom	Never
Q7	Do your fertility problems cause feelings of jealousy and resentment?	☐	☐	☐	☐	☐
Q8	Do you experience grief and/or feelings of loss about not being able to have a child (or more children)?	☐	☐	☐	☐	☐
Q9	Do you fluctuate between hope and despair because of fertility problems?	☐	☐	☐	☐	☐
Q10	Are you socially isolated because of fertility problems?	☐	☐	☐	☐	☐
*Q11	Are you and your partner affectionate with each other even though you have fertility problems?	☐	☐	☐	☐	☐
Q12	Do your fertility problems interfere with your day-to-day work or obligations?	☐	☐	☐	☐	☐
Q13	Do you feel uncomfortable attending social situations like holidays and celebrations because of your fertility problems?	☐	☐	☐	☐	☐
Q14	Do you feel your family can understand what you are going through?	☐	☐	☐	☐	☐
	For each question, check the response that is closest to your current thoughts and feelings	An Extreme Amount	Very Much	A Moderate Amount	A Little	Not At All
*Q15	Have fertility problems strengthened your commitment to your partner?	☐	☐	☐	☐	☐
Q16	Do you feel sad and depressed about your fertility problems?	☐	☐	☐	☐	☐
Q17	Do your fertility problems make you inferior to people with children?	☐	☐	☐	☐	☐
Q18	Are you bothered by fatigue because of fertility problems?	☐	☐	☐	☐	☐
*Q19	Have fertility problems had a negative impact on your relationship with your partner?	☐	☐	☐	☐	☐
*Q20	Do you find it difficult to talk to your partner about your feelings related to infertility?	☐	☐	☐	☐	☐
*Q21	Are you content with your relationship even though you have fertility problems?	☐	☐	☐	☐	☐
Q22	Do you feel social pressure on you to have (or have more) children?	☐	☐	☐	☐	☐
Q23	Do your fertility problems make you angry?	☐	☐	☐	☐	☐
Q24	Do you feel pain and physical discomfort because of your fertility problems?	☐	☐	☐	☐	☐

23 Appendix H: Stop Bang Questionnaire

STOP BANG Questionnaire

Height _____ inches/cm Weight _____ lb/kg
Age _____
Male/Female
BMI _____
Collar size of shirt: S, M, L, XL, or _____ inches/cm
Neck circumference* _____ cm

1. *S*noring
Do you *s*nore loudly (louder than talking or loud enough to be heard through closed doors)?
Yes No

2. *T*ired
Do you often feel *t*ired, fatigued, or sleepy during daytime?
Yes No

3. *O*bserved
Has anyone *o*bserved you stop breathing during your sleep?
Yes No

4. Blood *p*ressure
Do you have or are you being treated for high blood *p*ressure?
Yes No

5. *B*MI
*B*MI more than 35 kg/m^2?
Yes No

6. *A*ge
*A*ge over 50 yr old?
Yes No

7. *N*eck circumference
*N*eck circumference greater than 40 cm?
Yes No

8. *G*ender
*G*ender male?
Yes No

* Neck circumference is measured by staff

High risk of OSA: answering yes to three or more items
Low risk of OSA: answering yes to less than three items

Adapted from:
STOP Questionnaire
A Tool to Screen Patients for Obstructive Sleep Apnea
Frances Chung, F.R.C.P.C., Balaji Yegneswaran, M.B.B.S.,† Pu Liao, M.D.,‡ Sharon A. Chung, Ph.D.,§ Santhira Vairavanathan, M.B.B.S.,_ Sazzadul Islam, M.Sc.,_ Ali Khajehdehi, M.D.,† Colin M. Shapiro, F.R.C.P.C.#*

24 Appendix I: Epworth Sleepiness Scale

EPWORTH SLEEPINESS SCALE (ESS)

The following questionnaire will help you measure your general level of daytime sleepiness. You are to rate the chance that you would *doze off or fall asleep* during different routine daytime situations. Answers to the questions are rated on a reliable scale called the Epworth Sleepiness Scale (ESS). Each item is rated from 0 to 3: with 0 meaning you would never *doze or fall asleep* in a given situation; and 3 meaning that there is a very high chance that you would *doze or fall asleep* in that situation.

How likely are you to *doze off or fall asleep* in the following situations, in contrast to just feeling tired? Even if you haven't done some of the activities recently, think about how they would have affected you.

Use this scale to choose the most appropriate number for each situation:

0 = would never doze **2 = moderate chance of dozing**
1 = slight chance of dozing **3 = high chance of dozing**
It is important that you circle a number (0 to 3) for EACH situation.

SITUATION	CHANCE OF DOZING			
Sitting and Reading	0	1	2	3
Watching Television	0	1	2	3
Sitting inactive in a public place (theater/meeting)	0	1	2	3
As a passenger in a car for an hour without a break	0	1	2	3
Lying down to rest in the afternoon	0	1	2	3
Sitting and talking to someone	0	1	2	3
Sitting quietly after lunch (with no alcohol)	0	1	2	3
In a car, while stopped in traffic	0	1	2	3

TOTAL SCORE _________

Name: ___________________________________

Date: ______________________________

Revised 09/25/08

25 Appendix J: International Index of Erectile Function (IIEF)

(Write the number that best describes your erectile function for the past 4 weeks in the spaces provided.)

Over the past four weeks:	
1. How often were you able to get an erection during sexual activity? _______	0 = No sexual activity<br>1 = Almost never/never<br>2 = A few times (much less than half the time)<br>3 = Sometimes (about half the time)<br>4 = Most times (much more than half the time)<br>5 = Almost always/always
2. When you had erections with sexual stimulation, how often were your erections hard enough for penetration? _______	0 = No sexual activity<br>1 = Almost never/never<br>2 = A few times (much less than half the time)<br>3 = Sometimes (about half the time)<br>4 = Most times (much more than half the time)<br>5 = Almost always/always
3. When you attempted sexual intercourse, how often were you able to penetrate (enter) your partner? _______	0 = Did not attempt intercourse<br>1 = Almost never/never<br>2 = A few times (much less than half the time)<br>3 = Sometimes (about half the time)<br>4 = Most times (much more than half the time)<br>5 = Almost always/always
4. During intercourse, how often were you able to maintain your erection after you had penetrated (entered) your partner? _______	0 = Did not attempt intercourse<br>1 = Almost never/never<br>2 = A few times (much less than half the time)<br>3 = Sometimes (about half the time)<br>4 = Most times (much more than half the time)<br>5 = Almost always/always
5. During sexual intercourse, how difficult was it to maintain your erection to completion of intercourse? _______	0 = Did not attempt intercourse<br>1 = Extremely difficult<br>2 = Very difficult<br>3 = Difficult<br>4 = Slightly difficult<br>5 = Not difficult
6. How many times have you attempted sexual intercourse? _______	0 = No attempts<br>1 = One to two attempts<br>2 = Three to four attempts<br>3 = Five to six attempts<br>4 = Seven to ten attempts<br>5 = Eleven or more attempts

7. When you attempted sexual intercourse, how often was it satisfactory for you? _______	0 = Did not attempt intercourse<br>1 = Almost never/never<br>2 = A few times (much less than half the time)<br>3 = Sometimes (about half the time)<br>4 = Most times (much more than half the time)<br>5 = Almost always/always
8. How much have you enjoyed sexual intercourse? _______	0 = No intercourse<br>1 = No enjoyment<br>2 = Not very enjoyable<br>3 = Fairly enjoyable<br>4 = Highly enjoyable<br>5 = Very highly enjoyable
9. When you had sexual stimulation <u>or</u> intercourse, how often did you ejaculate? _______	0 = No sexual stimulation/intercourse<br>1 = Almost never/never<br>2 = A few times (much less than half the time)<br>3 = Sometimes (about half the time)<br>4 = Most times (much more than half the time)<br>5 = Almost always/always
10. When you had sexual stimulation <u>or</u> intercourse, how often did you have the feeling of orgasm or climax? _______	0 = No sexual stimulation/intercourse<br>1 = Almost never/never<br>2 = A few times (much less than half the time)<br>3 = Sometimes (about half the time)<br>4 = Most times (much more than half the time)<br>5 = Almost always/always
11. How often have you felt sexual desire? _______	1 = Almost never/never<br>2 = A few times (much less than half the time)<br>3 = Sometimes (about half the time)<br>4 = Most times (much more than half the time)<br>5 = Almost always/always
12. How would you rate your sexual desire? _______	1 = Very low/none at all<br>2 = Low<br>3 = Moderate<br>4 = High<br>5 = Very high
13. How satisfied have you been with your overall sex life? _______	1 = Very dissatisfied<br>2 = Moderately dissatisfied<br>3 = About equally satisfied and dissatisfied<br>4 = Moderately satisfied<br>5 = Very satisfied

14. How satisfied have you been with your <u>sexual relationship</u> with your partner? _______	1 = Very dissatisfied<br>2 = Moderately dissatisfied<br>3 = About equally satisfied and dissatisfied<br>4 = Moderately satisfied<br>5 = Very satisfied
15. How would you rate your <u>confidence</u> that you could get and keep an erection? _______	1 = Very low<br>2 = Low<br>3 = Moderate<br>4 = High<br>5 = Very high

*RC Rosen, A Riley, G Wagner et al. The international index of erectile dysfunction (IIEF): a multidimensional scale for assessment of erectile dysfunction. Urology 1997 49: 822-30.

Copyright 2004 Urology Specialists, S.C. All rights reserved. Last Updated: 18 May 2004

This is a sample form. Do not use for scanning.

NATIONAL INSTITUTES OF HEALTH

Diet History Questionnaire II

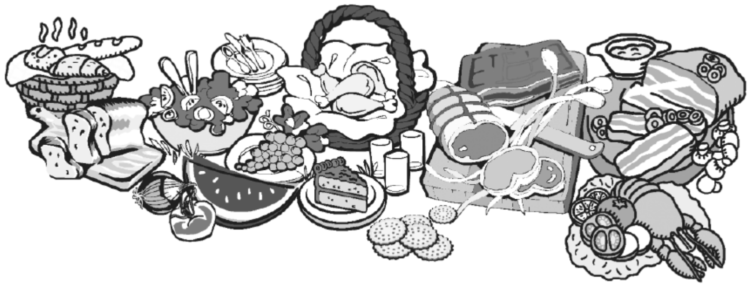

GENERAL INSTRUCTIONS

- Answer each question as best you can. Estimate if you are not sure. A guess is better than leaving a blank.
- Use only a black ball-point pen. Do not use a pencil or felt-tip pen. Do not fold, staple, or tear the pages.
- Put an X in the box next to your answer.
- If you make any changes, cross out the incorrect answer and put an X in the box next to the correct answer. Also draw a circle around the correct answer.
- If you mark NEVER, NO, or DON'T KNOW for a question, please follow any arrows or instructions that direct you to the next question.

BEFORE TURNING THE PAGE, PLEASE COMPLETE THE FOLLOWING QUESTIONS.

Today's date:

MONTH	DAY		YEAR
☐ Jan	|___|___|		☐ 2010
☐ Feb	☐0	☐0	☐ 2011
☐ Mar	☐1	☐1	☐ 2012
☐ Apr	☐2	☐2	☐ 2013
☐ May	☐3	☐3	☐ 2014
☐ Jun		☐4	☐ 2015
☐ Jul		☐5	☐ 2016
☐ Aug		☐6	☐ 2017
☐ Sep		☐7	☐ 2018
☐ Oct		☐8	☐ 2019
☐ Nov		☐9	☐ 2020
☐ Dec			

In what month were you born?

- ☐ Jan
- ☐ Feb
- ☐ Mar
- ☐ Apr
- ☐ May
- ☐ Jun
- ☐ Jul
- ☐ Aug
- ☐ Sep
- ☐ Oct
- ☐ Nov
- ☐ Dec

In what year were you born?

19 |___|___|

☐0	☐0
☐1	☐1
☐2	☐2
☐3	☐3
☐4	☐4
☐5	☐5
☐6	☐6
☐7	☐7
☐8	☐8
☐9	☐9

Are you male or female?

- ☐Male
- ☐Female

BAR CODE LABEL OR SUBJECT ID HERE

|___|___|___|___|___|___|___|___|

DHQ II PastYear

This is a sample form. Do not use for scanning.

1. Over the past 12 months, how often did you drink **carrot juice**?

☐ NEVER (GO TO QUESTION 2)

☐ 1 time per month or less
☐ 2–3 times per month
☐ 1–2 times per week
☐ 3–4 times per week
☐ 5–6 times per week
☐ 1 time per day
☐ 2–3 times per day
☐ 4–5 times per day
☐ 6 or more times per day

1a. Each time you drank **carrot juice**, how much did you usually drink?

☐ Less than ½ cup (4 ounces)
☐ ½ to 1¼ cups (4 to 10 ounces)
☐ More than 1¼ cups (10 ounces)

2. Over the past 12 months, how often did you drink **tomato juice** or **other vegetable juice**? *(Please do not include carrot juice.)*

☐ NEVER (GO TO QUESTION 3)

☐ 1 time per month or less
☐ 2–3 times per month
☐ 1–2 times per week
☐ 3–4 times per week
☐ 5–6 times per week
☐ 1 time per day
☐ 2–3 times per day
☐ 4–5 times per day
☐ 6 or more times per day

2a. Each time you drank **tomato juice** or **other vegetable juice**, how much did you usually drink?

☐ Less than ¾ cup (6 ounces)
☐ ¾ to 1¼ cups (6 to 10 ounces)
☐ More than 1¼ cups (10 ounces)

3. Over the past 12 months, how often did you drink **orange juice** or **grapefruit juice?**

☐ NEVER (GO TO QUESTION 4)

☐ 1 time per month or less
☐ 2–3 times per month
☐ 1–2 times per week
☐ 3–4 times per week
☐ 5–6 times per week
☐ 1 time per day
☐ 2–3 times per day
☐ 4–5 times per day
☐ 6 or more times per day

3a. Each time you drank **orange juice** or **grapefruit juice**, how much did you usually drink?

☐ Less than ¾ cup (6 ounces)
☐ ¾ to 1¼ cups (6 to 10 ounces)
☐ More than 1¼ cups (10 ounces)

Question 4 appears in the next column

3b. How often was the orange juice or grapefruit juice you drank **calcium-fortified**?

☐ Almost never or never
☐ About ¼ of the time
☐ About ½ of the time
☐ About ¾ of the time
☐ Almost always or always

4. Over the past 12 months, how often did you drink **other 100% fruit juice** or **100% fruit juice mixtures** (such as apple, grape, pineapple, or others)?

☐ NEVER (GO TO QUESTION 5)

☐ 1 time per month or less
☐ 2–3 times per month
☐ 1–2 times per week
☐ 3–4 times per week
☐ 5–6 times per week
☐ 1 time per day
☐ 2–3 times per day
☐ 4–5 times per day
☐ 6 or more times per day

4a. Each time you drank **other 100% fruit juice** or **100% fruit juice mixtures**, how much did you usually drink?

☐ Less than ¾ cup (6 ounces)
☐ ¾ to 1½ cups (6 to 12 ounces)
☐ More than 1½ cups (12 ounces)

4b. How often were the other 100% fruit juice or 100% fruit juice mixtures you drank **calcium-fortified**?

☐ Almost never or never
☐ About ¼ of the time
☐ About ½ of the time
☐ About ¾ of the time
☐ Almost always or always

5. How often did you drink **other fruit drinks** (such as cranberry cocktail, Hi-C, lemonade, or Kool-Aid, diet or regular)?

☐ NEVER (GO TO QUESTION 6)

☐ 1 time per month or less
☐ 2–3 times per month
☐ 1–2 times per week
☐ 3–4 times per week
☐ 5–6 times per week
☐ 1 time per day
☐ 2–3 times per day
☐ 4–5 times per day
☐ 6 or more times per day

Question 6 appears on the next page

This is a sample form. Do not use for scanning.

Over the past 12 months…

5a. Each time you drank **fruit drinks**, how much did you usually drink?

☐ Less than 1 cup (8 ounces)
☐ 1 to 2 cups (8 to 16 ounces)
☐ More than 2 cups (16 ounces)

5b. How often were your fruit drinks **diet** or **sugar-free**?

☐ Almost never or never
☐ About ¼ of the time
☐ About ½ of the time
☐ About ¾ of the time
☐ Almost always or always

6. How often did you drink **milk as a beverage** (NOT in coffee, NOT in cereal)? *(Please do not include chocolate milk and hot chocolate.)*

☐ NEVER (GO TO QUESTION 7)

☐ 1 time per month or less
☐ 2–3 times per month
☐ 1–2 times per week
☐ 3–4 times per week
☐ 5–6 times per week
☐ 1 time per day
☐ 2–3 times per day
☐ 4–5 times per day
☐ 6 or more times per day

6a. Each time you drank **milk as a beverage**, how much did you usually drink?

☐ Less than 1 cup (8 ounces)
☐ 1 to 1½ cups (8 to 12 ounces)
☐ More than 1½ cups (12 ounces)

6b. What kind of **milk** did you usually drink?

☐ Whole milk
☐ 2% fat milk
☐ 1 % fat milk
☐ Skim, nonfat, or ½% fat milk
☐ Soy milk
☐ Rice milk
☐ Other

7. How often did you drink **chocolate milk** (including hot chocolate)?

☐ NEVER (GO TO QUESTION 8)

☐ 1 time per month or less
☐ 2–3 times per month
☐ 1–2 times per week
☐ 3–4 times per week
☐ 5–6 times per week
☐ 1 time per day
☐ 2–3 times per day
☐ 4–5 times per day
☐ 6 or more times per day

Question 8 appears in the next column

7a. Each time you drank **chocolate milk**, how much did you usually drink?

☐ Less than 1 cup (8 ounces)
☐ 1 to 1½ cups (8 to 12 ounces)
☐ More than 1½ cups (12 ounces)

7b. How often was the chocolate milk **reduced-fat** or **fat-free**?

☐ Almost never or never
☐ About ¼ of the time
☐ About ½ of the time
☐ About ¾ of the time
☐ Almost always or always

8: How often did you drink **meal replacement** or **high-protein beverages** (such as Instant Breakfast, Ensure, Slimfast, Sustacal or others)?

☐ NEVER (GO TO QUESTION 9)

☐ 1 time per month or less
☐ 2–3 times per month
☐ 1–2 times per week
☐ 3–4 times per week
☐ 5–6 times per week
☐ 1 time per day
☐ 2–3 times per day
☐ 4–5 times per day
☐ 6 or more times per day

8a. Each time you drank **meal replacement** or **high-protein beverages**, how much did you usually drink?

☐ Less than 1 cup (8 ounces)
☐ 1 to 1½ cups (8 to 12 ounces)
☐ More than 1½ cups (12 ounces)

9. Over the past 12 months, did you drink **soda** or **pop**?

☐ NO (GO TO QUESTION 10)
☐ YES

9a. How often did you drink **soda** or **pop IN THE SUMMER**?

☐ NEVER

☐ 1 time per month or less
☐ 2–3 times per month
☐ 1–2 times per week
☐ 3–4 times per week
☐ 5–6 times per week
☐ 1 time per day
☐ 2–3 times per day
☐ 4–5 times per day
☐ 6 or more times per day

Question 10 appears on the next page

This is a sample form. Do not use for scanning.

Over the past 12 months…

9b. How often did you drink **soda** or **pop** **DURING THE REST OF THE YEAR**?

- ☐ NEVER
- ☐ 1 time per month or less
- ☐ 2–3 times per month
- ☐ 1–2 times per week
- ☐ 3–4 times per week
- ☐ 5–6 times per week
- ☐ 1 time per day
- ☐ 2–3 times per day
- ☐ 4–5 times per day
- ☐ 6 or more times per day

9c. Each time you drank **soda** or **pop,** how much did you usually drink?

- ☐ Less than 12 ounces or less than 1 can or bottle
- ☐ 12 to 16 ounces or 1 can or bottle
- ☐ More than 16 ounces or more than 1 can or bottle

9d. How often were these sodas or pop **diet** or **sugar-free**?

- ☐ Almost never or never
- ☐ About ¼ of the time
- ☐ About ½ of the time
- ☐ About ¾ of the time
- ☐ Almost always or always

9e. How often were these sodas or pop **caffeine-free**?

- ☐ Almost never or never
- ☐ About ¼ of the time
- ☐ About ½ of the time
- ☐ About ¾ of the time
- ☐ Almost always or always

10. Over the past 12 months, did you drink **sports drinks** (such as Propel, PowerAde, or Gatorade)?

- ☐ NO (GO TO QUESTION 11)
- ☐ YES

10a. How often did you drink **sports drinks IN THE SUMMER**?

- ☐ NEVER
- ☐ 1 time per month or less
- ☐ 2–3 times per month
- ☐ 1–2 times per week
- ☐ 3–4 times per week
- ☐ 5–6 times per week
- ☐ 1 time per day
- ☐ 2–3 times per day
- ☐ 4–5 times per day
- ☐ 6 or more times per day

Question 11 appears in the next column

10b. How often did you drink **sports drinks DURING THE REST OF THE YEAR**?

- ☐ NEVER
- ☐ 1 time per month or less
- ☐ 2–3 times per month
- ☐ 1–2 times per week
- ☐ 3–4 times per week
- ☐ 5–6 times per week
- ☐ 1 time per day
- ☐ 2–3 times per day
- ☐ 4–5 times per day
- ☐ 6 or more times per day

10c. Each time you drank **sports drinks**, how much did you usually drink?

- ☐ Less than 12 ounces or less than 1 bottle
- ☐ 12 to 24 ounces or 1 to 2 bottles
- ☐ More than 24 ounces or more than 2 bottles

11. Over the past 12 months, did you drink **energy drinks** (such as Red Bull or Jolt)?

- ☐ NO (GO TO QUESTION 12)
- ☐ YES

11a. How often did you drink **energy drinks IN THE SUMMER**?

- ☐ NEVER
- ☐ 1 time per month or less
- ☐ 2–3 times per month
- ☐ 1–2 times per week
- ☐ 3–4 times per week
- ☐ 5–6 times per week
- ☐ 1 time per day
- ☐ 2–3 times per day
- ☐ 4–5 times per day
- ☐ 6 or more times per day

11b. How often did you drink **energy drinks DURING THE REST OF THE YEAR**?

- ☐ NEVER
- ☐ 1 time per month or less
- ☐ 2–3 times per month
- ☐ 1–2 times per week
- ☐ 3–4 times per week
- ☐ 5–6 times per week
- ☐ 1 time per day
- ☐ 2–3 times per day
- ☐ 4–5 times per day
- ☐ 6 or more times per day

11c. Each time you drank **energy drinks**, how much did you usually drink?

- ☐ Less than 8 ounces or less than 1 cup
- ☐ 8 to 16 ounces or 1 to 2 cups
- ☐ More than 16 ounces or more than 2 cups

Question 12 appears on the next page

This is a sample form. Do not use for scanning.

Over the past 12 months…

12. Over the past 12 months, did you drink **beer**?

☐ NO (GO TO QUESTION 13)

☐ YES

12a. How often did you drink **beer IN THE SUMMER**?

☐ NEVER

☐ 1 time per month or less
☐ 2–3 times per month
☐ 1–2 times per week
☐ 3–4 times per week
☐ 5–6 times per week
☐ 1 time per day
☐ 2–3 times per day
☐ 4–5 times per day
☐ 6 or more times per day

12b. How often did you drink **beer DURING THE REST OF THE YEAR**?

☐ NEVER

☐ 1 time per month or less
☐ 2–3 times per month
☐ 1–2 times per week
☐ 3–4 times per week
☐ 5–6 times per week
☐ 1 time per day
☐ 2–3 times per day
☐ 4–5 times per day
☐ 6 or more times per day

12c. Each time you drank **beer**, how much did you usually drink?

☐ Less than a 12-ounce can or bottle
☐ 1 to 3 12-ounce cans or bottles
☐ More than 3 12-ounce cans or bottles

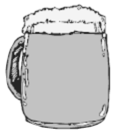

13. Over the past 12 months, did you drink **water** (including tap, bottled, and carbonated water)?

☐ NO (GO TO QUESTION 14)

☐ YES

13a. How often did you drink **water** (including tap, bottled, and carbonated water) **IN THE SUMMER**?

☐ NEVER

☐ 1 time per month or less
☐ 2–3 times per month
☐ 1–2 times per week
☐ 3–4 times per week
☐ 5–6 times per week
☐ 1 time per day
☐ 2–3 times per day
☐ 4–5 times per day
☐ 6 or more times per day

Question 14 appears in the next column

13b. How often did you drink **water** (including tap, bottled, and carbonated water) **DURING THE REST OF THE YEAR**?

☐ NEVER

☐ 1 time per month or less
☐ 2–3 times per month
☐ 1–2 times per week
☐ 3–4 times per week
☐ 5–6 times per week
☐ 1 time per day
☐ 2–3 times per day
☐ 4–5 times per day
☐ 6 or more times per day

13c. Each time you drank **water**, how much did you usually drink?

☐ Less than 12 ounces or less than 1 bottle
☐ 12 to 24 ounces or 1 to 2 bottles
☐ More than 24 ounces or more than 2 bottles

13d. How often was the water you drank **tap water**?

☐ Almost never or never
☐ About ¼ of the time
☐ About ½ of the time
☐ About ¾ of the time
☐ Almost always or always

13e. How often was the water you drank **bottled, sweetened water** (with low or no-calorie sweetener, including carbonated water)?

☐ Almost never or never
☐ About ¼ of the time
☐ About ½ of the time
☐ About ¾ of the time
☐ Almost always or always

13f. How often was the water you drank **bottled, unsweetened water** (including carbonated water)?

☐ Almost never or never
☐ About ¼ of the time
☐ About ½ of the time
☐ About ¾ of the time
☐ Almost always or always

14. How often did you drink **wine** or **wine coolers**?

☐ NEVER (GO TO QUESTION 15)

☐ 1 time per month or less
☐ 2–3 times per month
☐ 1–2 times per week
☐ 3–4 times per week
☐ 5–6 times per week
☐ 1 time per day
☐ 2–3 times per day
☐ 4–5 times per day
☐ 6 or more times per day

Question 15 appears on the next page

This is a sample form. Do not use for scanning.

Over the past 12 months…

14a. Each time you drank **wine** or **wine coolers**, how much did you usually drink?

- ☐ Less than 5 ounces or less than 1 glass
- ☐ 5 to 12 ounces or 1 to 2 glasses
- ☐ More than 12 ounces or more than 2 glasses

15. How often did you drink **liquor** or **mixed drinks**?

- ☐ NEVER (GO TO QUESTION 16)
- ☐ 1 time per month or less
- ☐ 2–3 times per month
- ☐ 1–2 times per week
- ☐ 3–4 times per week
- ☐ 5–6 times per week
- ☐ 1 time per day
- ☐ 2–3 times per day
- ☐ 4–5 times per day
- ☐ 6 or more times per day

15a. Each time you drank **liquor** or **mixed drinks**, how much did you usually drink?

- ☐ Less than 1 shot of liquor
- ☐ 1 to 3 shots of liquor
- ☐ More than 3 shots of liquor

16. Over the past 12 months, did you eat **oatmeal, grits,** or **other cooked cereal?**

- ☐ NO (GO TO QUESTION 17)
- ☐ YES

16a. How often did you eat **oatmeal**, **grits**, or **other cooked cereal IN THE WINTER**?

- ☐ NEVER
- ☐ 1–6 times per winter
- ☐ 7–11 times per winter
- ☐ 1 time per month
- ☐ 2–3 times per month
- ☐ 1 time per week
- ☐ 2 times per week
- ☐ 3–4 times per week
- ☐ 5–6 times per week
- ☐ 1 time per day
- ☐ 2 or more times per day

16b. How often did you eat **oatmeal**, **grits**, or **other cooked cereal DURING THE REST OF THE YEAR**?

- ☐ NEVER
- ☐ 1–6 times per year
- ☐ 7–11 times per year
- ☐ 1 time per month
- ☐ 2–3 times per month
- ☐ 1 time per week
- ☐ 2 times per week
- ☐ 3–4 times per week
- ☐ 5–6 times per week
- ☐ 1 time per day
- ☐ 2 or more times per day

Question 17 appears in the next column

16c. Each time you ate **oatmeal, grits,** or **other cooked cereal,** how much did you usually eat?

- ☐ Less than ¾ cup
- ☐ ¾ to 1¼ cups
- ☐ More than 1¼ cups

16d. How often was **butter** or **margarine** added to your oatmeal, grits or other cooked cereal?

- ☐ Almost never or never
- ☐ About ¼ of the time
- ☐ About ½ of the time
- ☐ About ¾ of the time
- ☐ Almost always or always

17. How often did you eat **cold cereal**?

- ☐ NEVER (GO TO QUESTION 18)
- ☐ 1–6 times per year
- ☐ 7–11 times per year
- ☐ 1 time per month
- ☐ 2–3 times per month
- ☐ 1 time per week
- ☐ 2 times per week
- ☐ 3–4 times per week
- ☐ 5–6 times per week
- ☐ 1 time per day
- ☐ 2 or more times per day

17a. Each time you ate **cold cereal**, how much did you usually eat?

- ☐ Less than 1 cup
- ☐ 1 to 2½ cups
- ☐ More than 2½ cups

17b. How often was the cold cereal you ate **Total Raisin Bran**, **Total Cereal**, or **Product 19**?

- ☐ Almost never or never
- ☐ About ¼ of the time
- ☐ About ½ of the time
- ☐ About ¾ of the time
- ☐ Almost always or always

17c. How often was the cold cereal you ate **All Bran**, **Fiber One**, **100% Bran**, or **All-Bran Bran Buds**?

- ☐ Almost never or never
- ☐ About ¼ of the time
- ☐ About ½ of the time
- ☐ About ¾ of the time
- ☐ Almost always or always

Question 18 appears on the next page

This is a sample form. Do not use for scanning.

Over the past 12 months…

17d. How often was the cold cereal you ate **some other bran** or **fiber cereal** (such as Cheerios, Shredded Wheat, Raisin Bran, Bran Flakes, Grape-Nuts, Granola, Wheaties, or Healthy Choice)?

- ☐ Almost never or never
- ☐ About ¼ of the time
- ☐ About ½ of the time
- ☐ About ¾ of the time
- ☐ Almost always or always

17e. How often was the cold cereal you ate any **other type of cold cereal** (such as Corn Flakes, Rice Krispies, Frosted Flakes, Special K, Froot Loops, Cap'n Crunch, or others)?

- ☐ Almost never or never
- ☐ About ¼ of the time
- ☐ About ½ of the time
- ☐ About ¾ of the time
- ☐ Almost always or always

17f. Was **milk** added to your cold cereal?

- ☐ NO (GO TO QUESTION 18)
- ☐ YES

17g. What kind of **milk** was usually added?

- ☐ Whole milk
- ☐ 2% fat milk
- ☐ 1% fat milk
- ☐ Skim, nonfat, or ½% fat milk
- ☐ Soy milk
- ☐ Rice milk
- ☐ Other

17h. Each time **milk was added to your cold cereal**, how much was usually added?

- ☐ Less than ½ cup
- ☐ ½ to 1 cup
- ☐ More than 1 cup

18. How often did you eat **applesauce**?

- ☐ NEVER (GO TO QUESTION 19)

☐ 1–6 times per year	☐ 2 times per week
☐ 7–11 times per year	☐ 3–4 times per week
☐ 1 time per month	☐ 5–6 times per week
☐ 2–3 times per month	☐ 1 time per day
☐ 1 time per week	☐ 2 or more times per day

Question 19 appears in the next column

18a. Each time you ate **applesauce**, how much did you usually eat?

- ☐ Less than ½ cup
- ☐ ½ to 1 cup
- ☐ More than 1 cup

19. How often did you eat **apples**?

- ☐ NEVER (GO TO QUESTION 20)

☐ 1–6 times per year	☐ 2 times per week
☐ 7–11 times per year	☐ 3–4 times per week
☐ 1 time per month	☐ 5–6 times per week
☐ 2–3 times per month	☐ 1 time per day
☐ 1 time per week	☐ 2 or more times per day

19a. Each time you ate **apples**, how many did you usually eat?

- ☐ Less than 1 apple
- ☐ 1 apple
- ☐ More than 1 apple

20. How often did you eat **pears** (fresh, canned, or frozen)?

- ☐ NEVER (GO TO QUESTION 21)

☐ 1–6 times per year	☐ 2 times per week
☐ 7–11 times per year	☐ 3–4 times per week
☐ 1 time per month	☐ 5–6 times per week
☐ 2–3 times per month	☐ 1 time per day
☐ 1 time per week	☐ 2 or more times per day

20a. Each time you ate **pears**, how many did you usually eat?

- ☐ Less than 1 pear
- ☐ 1 pear
- ☐ More than 1 pear

21. How often did you eat **bananas?**

- ☐ NEVER (GO TO QUESTION 22)

☐ 1–6 times per year	☐ 2 times per week
☐ 7–11 times per year	☐ 3–4 times per week
☐ 1 time per month	☐ 5–6 times per week
☐ 2–3 times per month	☐ 1 time per day
☐ 1 time per week	☐ 2 or more times per day

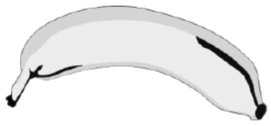

Question 22 appears on the next page

This is a sample form. Do not use for scanning.

Over the past 12 months…

21a. Each time you ate **bananas**, how many did you usually eat?

- ☐ Less than 1 banana
- ☐ 1 banana
- ☐ More than 1 banana

22. How often did you eat **dried fruit** (such as prunes or raisins)? *(Please do not include dried apricots.)*

- ☐ NEVER (GO TO QUESTION 23)
- ☐ 1–6 times per year
- ☐ 7–11 times per year
- ☐ 1 time per month
- ☐ 2–3 times per month
- ☐ 1 time per week
- ☐ 2 times per week
- ☐ 3–4 times per week
- ☐ 5–6 times per week
- ☐ 1 time per day
- ☐ 2 or more times per day

22a. Each time you ate **dried fruit**, how much did you usually eat?

- ☐ Less than 2 tablespoons
- ☐ 2 to 5 tablespoons
- ☐ More than 5 tablespoons

23. Over the past 12 months, did you eat **peaches, nectarines,** or **plums**?

- ☐ NO (GO TO QUESTION 24)
- ☐ YES

23a. How often did you eat **fresh peaches, nectarines**, or **plums WHEN IN SEASON**?

- ☐ NEVER
- ☐ 1–6 times per season
- ☐ 7–11 times per season
- ☐ 1 time per month
- ☐ 2–3 times per month
- ☐ 1 time per week
- ☐ 2 times per week
- ☐ 3–4 times per week
- ☐ 5–6 times per week
- ☐ 1 time per day
- ☐ 2 or more times per day

23b. How often did you eat **peaches, nectarines,** or **plums** (fresh, canned, or frozen) **DURING THE REST OF THE YEAR**?

- ☐ NEVER
- ☐ 1–6 times per year
- ☐ 7–11 times per year
- ☐ 1 time per month
- ☐ 2–3 times per month
- ☐ 1 time per week
- ☐ 2 times per week
- ☐ 3–4 times per week
- ☐ 5–6 times per week
- ☐ 1 time per day
- ☐ 2 or more times per day

Question 24 appears in the next column

23c. Each time you ate **peaches, nectarines,** or **plums**, how much did you usually eat?

- ☐ Less than 1 fruit or less than ½ cup
- ☐ 1 to 2 fruits or ½ to ¾ cup
- ☐ More than 2 fruits or more than ¾ cup

24. How often did you eat **grapes**?

- ☐ NEVER (GO TO QUESTION 25)
- ☐ 1–6 times per year
- ☐ 7–11 times per year
- ☐ 1 time per month
- ☐ 2–3 times per month
- ☐ 1 time per week
- ☐ 2 times per week
- ☐ 3–4 times per week
- ☐ 5–6 times per week
- ☐ 1 time per day
- ☐ 2 or more times per day

24a. Each time you ate **grapes**, how much did you usually eat?

- ☐ Less than ½ cup or less than 10 grapes
- ☐ ½ to 1 cup or 10 to 30 grapes
- ☐ More than 1 cup or more than 30 grapes

25. Over the past 12 months, did you eat **cantaloupe**?

- ☐ NO (GO TO QUESTION 26)
- ☐ YES

25a. How often did you eat **fresh cantaloupe WHEN IN SEASON**?

- ☐ NEVER
- ☐ 1–6 times per season
- ☐ 7–11 times per season
- ☐ 1 time per month
- ☐ 2–3 times per month
- ☐ 1 time per week
- ☐ 2 times per week
- ☐ 3–4 times per week
- ☐ 5–6 times per week
- ☐ 1 time per day
- ☐ 2 or more times per day

25b. How often did you eat **cantaloupe** (fresh or frozen) **DURING THE REST OF THE YEAR**?

- ☐ NEVER
- ☐ 1–6 times per year
- ☐ 7–11 times per year
- ☐ 1 time per month
- ☐ 2–3 times per month
- ☐ 1 time per week
- ☐ 2 times per week
- ☐ 3–4 times per week
- ☐ 5–6 times per week
- ☐ 1 time per day
- ☐ 2 or more times per day

Question 26 appears on the next page

This is a sample form. Do not use for scanning.

Over the past 12 months…

25c. Each time you ate **cantaloupe**, how much did you usually eat?

- ☐ Less than ¼ melon or less than ½ cup
- ☐ ¼ melon or ½ to 1 cup
- ☐ More than ¼ melon or more than 1 cup

26. Over the past 12 months, did you eat **melon, other than cantaloupe** (such as watermelon or honeydew)?

- ☐ NO (GO TO QUESTION 27)
- ☐ YES

26a. How often did you eat **fresh melon, other than cantaloupe**, **WHEN IN SEASON**?

☐ NEVER

☐ 1–6 times per season	☐ 2 times per week
☐ 7–11 times per season	☐ 3–4 times per week
☐ 1 time per month	☐ 5–6 times per week
☐ 2–3 times per month	☐ 1 time per day
☐ 1 time per week	☐ 2 or more times per day

26b. How often did you eat **melon other than cantaloupe** (fresh or frozen) **DURING THE REST OF THE YEAR**?

☐ NEVER

☐ 1–6 times per year	☐ 2 times per week
☐ 7–11 times per year	☐ 3–4 times per week
☐ 1 time per month	☐ 5–6 times per week
☐ 2–3 times per month	☐ 1 time per day
☐ 1 time per week	☐ 2 or more times per day

26c. Each time you ate **melon other than cantaloupe**, how much did you usually eat?

- ☐ Less than ½ cup or 1 small wedge
- ☐ ½ to 2 cups or 1 medium wedge
- ☐ More than 2 cups or 1 large wedge

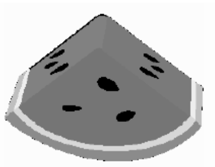

Question 27 appears in the next column

27. Over the past 12 months, did you eat **strawberries**?

- ☐ NO (GO TO QUESTION 28)
- ☐ YES

27a. How often did you eat **fresh strawberries WHEN IN SEASON**?

☐ NEVER

☐ 1–6 times per season	☐ 2 times per week
☐ 7–11 times per season	☐ 3–4 times per week
☐ 1 time per month	☐ 5–6 times per week
☐ 2–3 times per month	☐ 1 time per day
☐ 1 time per week	☐ 2 or more times per day

27b. How often did you eat **strawberries** (fresh or frozen) **DURING THE REST OF THE YEAR**?

☐ NEVER

☐ 1–6 times per year	☐ 2 times per week
☐ 7–11 times per year	☐ 3–4 times per week
☐ 1 time per month	☐ 5–6 times per week
☐ 2–3 times per month	☐ 1 time per day
☐ 1 time per week	☐ 2 or more times per day

27c. Each time you ate **strawberries**, how much did you usually eat?

- ☐ Less than ¼ cup or less than 3 berries
- ☐ ¼ to ¾ cup or 3 to 8 berries
- ☐ More than ¾ cup or more than 8 berries

28. Over the past 12 months, did you eat **oranges, tangerines**, or **clementines**?

- ☐ NO (GO TO QUESTION 29)
- ☐ YES

28a. How often did you eat **fresh oranges, tangerines**, or **clementines WHEN IN SEASON**?

☐ NEVER

☐ 1–6 times per season	☐ 2 times per week
☐ 7–11 times per season	☐ 3–4 times per week
☐ 1 time per month	☐ 5–6 times per week
☐ 2–3 times per month	☐ 1 time per day
☐ 1 time per week	☐ 2 or more times per day

Question 29 appears on the next page

This is a sample form. Do not use for scanning.

Over the past 12 months…

28b. How often did you eat **oranges**, **tangerines**, or **clementines** (fresh or canned) **DURING THE REST OF THE YEAR**?

- ☐ NEVER
- ☐ 1–6 times per year
- ☐ 7–11 times per year
- ☐ 1 time per month
- ☐ 2–3 times per month
- ☐ 1 time per week
- ☐ 2 times per week
- ☐ 3–4 times per week
- ☐ 5–6 times per week
- ☐ 1 time per day
- ☐ 2 or more times per day

28c. Each time you ate **oranges, tangerines,** or **clementines,** how many did you usually eat?

- ☐ Less than 1 fruit
- ☐ 1 fruit
- ☐ More than 1 fruit

29. Over the past 12 months, did you eat **grapefruit**?

- ☐ NO (GO TO QUESTION 30)
- ☐ YES

29a. How often did you eat **fresh grapefruit WHEN IN SEASON**?

- ☐ NEVER
- ☐ 1–6 times per season
- ☐ 7–11 times per season
- ☐ 1 time per month
- ☐ 2–3 times per month
- ☐ 1 time per week
- ☐ 2 times per week
- ☐ 3–4 times per week
- ☐ 5–6 times per week
- ☐ 1 time per day
- ☐ 2 or more times per day

29b. How often did you eat **grapefruit** (fresh or canned) **DURING THE REST OF THE YEAR?**

- ☐ NEVER
- ☐ 1–6 times per year
- ☐ 7–11 times per year
- ☐ 1 time per month
- ☐ 2–3 times per month
- ☐ 1 time per week
- ☐ 2 times per week
- ☐ 3–4 times per week
- ☐ 5–6 times per week
- ☐ 1 time per day
- ☐ 2 or more times per day

Question 30 appears in the next column

29c. Each time you ate **grapefruit**, how much did you usually eat?

- ☐ Less than ½ grapefruit
- ☐ ½ grapefruit
- ☐ More than ½ grapefruit

30. How often did you eat **pineapple**?

- ☐ NEVER (GO TO QUESTION 31)
- ☐ 1–6 times per year
- ☐ 7–11 times per year
- ☐ 1 time per month
- ☐ 2–3 times per month
- ☐ 1 time per week
- ☐ 2 times per week
- ☐ 3–4 times per week
- ☐ 5–6 times per week
- ☐ 1 time per day
- ☐ 2 or more times per day

30a. Each time you ate **pineapple**, how much did you usually eat?

- ☐ Less than ¼ cup or less than 1 medium slice
- ☐ ¼ to ¾ cup or 1 medium slice
- ☐ More than ¾ cup or more than 1 medium slice

31. How often did you eat **other kinds of fruit**?

- ☐ NEVER (GO TO QUESTION 32)
- ☐ 1–6 times per year
- ☐ 7–11 times per year
- ☐ 1 time per month
- ☐ 2–3 times per month
- ☐ 1 time per week
- ☐ 2 times per week
- ☐ 3–4 times per week
- ☐ 5–6 times per week
- ☐ 1 time per day
- ☐ 2 or more times per day

31a. Each time you ate **other kinds of fruit**, how much did you usually eat?

- ☐ Less than ¼ cup
- ☐ ¼ to ¾ cup
- ☐ More than ¾ cup

32. How often did you eat **COOKED greens** (such as spinach, turnip, collard, mustard, chard, or kale)?

- ☐ NEVER (GO TO QUESTION 33)
- ☐ 1–6 times per year
- ☐ 7–11 times per year
- ☐ 1 time per month
- ☐ 2–3 times per month
- ☐ 1 time per week
- ☐ 2 times per week
- ☐ 3–4 times per week
- ☐ 5–6 times per week
- ☐ 1 time per day
- ☐ 2 or more times per day

Question 33 appears on the next page

This is a sample form. Do not use for scanning.

Over the past 12 months…

32a. Each time you ate **COOKED greens**, how much did you usually eat?

- ☐ Less than ½ cup
- ☐ ½ to 1 cup
- ☐ More than 1 cup

33. How often did you eat **RAW greens** (such as spinach, turnip, collard, mustard, chard, or kale)? (*We will ask about lettuce later.*)

- ☐ NEVER (GO TO QUESTION 34)

☐ 1–6 times per year	☐ 2 times per week
☐ 7–11 times per year	☐ 3–4 times per week
☐ 1 time per month	☐ 5–6 times per week
☐ 2–3 times per month	☐ 1 time per day
☐ 1 time per week	☐ 2 or more times per day

33a. Each time you ate **RAW greens**, how much did you usually eat?

- ☐ Less than ½ cup
- ☐ ½ to 1 cup
- ☐ More than 1 cup

34. How often did you eat **coleslaw**?

- ☐ NEVER (GO TO QUESTION 35)

☐ 1–6 times per year	☐ 2 times per week
☐ 7–11 times per year	☐ 3–4 times per week
☐ 1 time per month	☐ 5–6 times per week
☐ 2–3 times per month	☐ 1 time per day
☐ 1 time per week	☐ 2 or more times per day

34a. Each time you ate **coleslaw**, how much did you usually eat?

- ☐ Less than ¼ cup
- ☐ ¼ to ¾ cup
- ☐ More than ¾ cup

35. How often did you eat **sauerkraut** or **cabbage** (other than coleslaw)?

- ☐ NEVER (GO TO QUESTION 36)

☐ 1–6 times per year	☐ 2 times per week
☐ 7–11 times per year	☐ 3–4 times per week
☐ 1 time per month	☐ 5–6 times per week
☐ 2–3 times per month	☐ 1 time per day
☐ 1 time per week	☐ 2 or more times per day

Question 36 appears in the next column

35a. Each time you ate **sauerkraut** or **cabbage**, how much did you usually eat?

- ☐ Less than ¼ cup
- ☐ ¼ to 1 cup
- ☐ More than 1 cup

36. How often did you eat **carrots** (fresh, canned, or frozen)?

- ☐ NEVER (GO TO QUESTION 37)

☐ 1–6 times per year	☐ 2 times per week
☐ 7–11 times per year	☐ 3–4 times per week
☐ 1 time per month	☐ 5–6 times per week
☐ 2–3 times per month	☐ 1 time per day
☐ 1 time per week	☐ 2 or more times per day

36a. Each time you ate **carrots**, how much did you usually eat?

- ☐ Less than ¼ cup or less than 2 baby carrots
- ☐ ¼ to ½ cup or 2 to 5 baby carrots
- ☐ More than ½ cup or more than 5 baby carrots

37. How often did you eat **string beans** or **green beans** (fresh, canned, or frozen)?

- ☐ NEVER (GO TO QUESTION 38)

☐ 1–6 times per year	☐ 2 times per week
☐ 7–11 times per year	☐ 3–4 times per week
☐ 1 time per month	☐ 5–6 times per week
☐ 2–3 times per month	☐ 1 time per day
☐ 1 time per week	☐ 2 or more times per day

37a. Each time you ate **string beans** or **green beans**, how much did you usually eat?

- ☐ Less than ½ cup
- ☐ ½ to 1 cup
- ☐ More than 1 cup

38. How often did you eat **peas** (fresh, canned, or frozen)?

- ☐ NEVER (GO TO QUESTION 39)

☐ 1–6 times per year	☐ 2 times per week
☐ 7–11 times per year	☐ 3–4 times per week
☐ 1 time per month	☐ 5–6 times per week
☐ 2–3 times per month	☐ 1 time per day
☐ 1 time per week	☐ 2 or more times per day

Question 39 appears on the next page

This is a sample form. Do not use for scanning.

Over the past 12 months…

38a. Each time you ate **peas**, how much did you usually eat?

- ☐ Less than ¼ cup
- ☐ ¼ to ¾ cup
- ☐ More than ¾ cup

39. Over the past 12 months, did you eat **corn**?

- ☐ NO (GO TO QUESTION 40)
- ☐ YES

39a. How often did you eat **fresh corn WHEN IN SEASON**?

☐ NEVER

- ☐ 1–6 times per season
- ☐ 7–11 times per season
- ☐ 1 time per month
- ☐ 2–3 times per month
- ☐ 1 time per week
- ☐ 2 times per week
- ☐ 3–4 times per week
- ☐ 5–6 times per week
- ☐ 1 time per day
- ☐ 2 or more times per day

39b. How often did you eat **corn** (fresh, canned, or frozen) **DURING THE REST OF THE YEAR**?

☐ NEVER

- ☐ 1–6 times per year
- ☐ 7–11 times per year
- ☐ 1 time per month
- ☐ 2–3 times per month
- ☐ 1 time per week
- ☐ 2 times per week
- ☐ 3–4 times per week
- ☐ 5–6 times per week
- ☐ 1 time per day
- ☐ 2 or more times per day

39c. Each time you ate **corn**, how much did you usually eat?

- ☐ Less than 1 ear or less than ½ cup
- ☐ 1 ear or ½ to 1 cup
- ☐ More than 1 ear or more than 1 cup

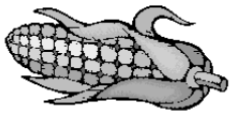

Question 40 appears in the next column

40. How often did you eat **broccoli** (fresh or frozen)?

☐ NEVER (GO TO QUESTION 41)

- ☐ 1–6 times per year
- ☐ 7–11 times per year
- ☐ 1 time per month
- ☐ 2–3 times per month
- ☐ 1 time per week
- ☐ 2 times per week
- ☐ 3–4 times per week
- ☐ 5–6 times per week
- ☐ 1 time per day
- ☐ 2 or more times per day

40a. Each time you ate **broccoli**, how much did you usually eat?

- ☐ Less than ¼ cup
- ☐ ¼ to 1 cup
- ☐ More than 1 cup

41. How often did you eat **cauliflower** or **Brussels sprouts** (fresh or frozen)?

☐ NEVER (GO TO QUESTION 42)

- ☐ 1–6 times per year
- ☐ 7–11 times per year
- ☐ 1 time per month
- ☐ 2–3 times per month
- ☐ 1 time per week
- ☐ 2 times per week
- ☐ 3–4 times per week
- ☐ 5–6 times per week
- ☐ 1 time per day
- ☐ 2 or more times per day

41a. Each time you ate **cauliflower** or **Brussels sprouts**, how much did you usually eat?

- ☐ Less than ¼ cup
- ☐ ¼ to ½ cup
- ☐ More than ½ cup

42. How often did you eat **asparagus** (fresh or frozen)?

☐ NEVER (GO TO QUESTION 43)

- ☐ 1–6 times per year
- ☐ 7–11 times per year
- ☐ 1 time per month
- ☐ 2–3 times per month
- ☐ 1 time per week
- ☐ 2 times per week
- ☐ 3–4 times per week
- ☐ 5–6 times per week
- ☐ 1 time per day
- ☐ 2 or more times per day

42a. Each time you ate **asparagus**, how much did you usually eat?

- ☐ Less than ⅓ cup or less than 4 spears
- ☐ ⅓ to ⅔ cup or 4 to 7 spears
- ☐ More than ⅔ cup or more than 7 spears

Question 43 appears on the next page

This is a sample form. Do not use for scanning.

Over the past 12 months…

43. How often did you eat **winter squash** (such as pumpkin, butternut, or acorn)?

- ☐ NEVER (GO TO QUESTION 44)
- ☐ 1–6 times per year
- ☐ 7–11 times per year
- ☐ 1 time per month
- ☐ 2–3 times per month
- ☐ 1 time per week
- ☐ 2 times per week
- ☐ 3–4 times per week
- ☐ 5–6 times per week
- ☐ 1 time per day
- ☐ 2 or more times per day

43a. Each time you ate **winter squash**, how much did you usually eat?

- ☐ Less than ½ cup
- ☐ ½ to ¾ cup
- ☐ More than ¾ cup

44. How often did you eat **mixed vegetables**?

- ☐ NEVER (GO TO QUESTION 45)
- ☐ 1–6 times per year
- ☐ 7–11 times per year
- ☐ 1 time per month
- ☐ 2–3 times per month
- ☐ 1 time per week
- ☐ 2 times per week
- ☐ 3–4 times per week
- ☐ 5–6 times per week
- ☐ 1 time per day
- ☐ 2 or more times per day

44a. Each time you ate **mixed vegetables**, how much did you usually eat?

- ☐ Less than ½ cup
- ☐ ½ to 1 cup
- ☐ More than 1 cup

45. How often did you eat **onions**?

- ☐ NEVER (GO TO QUESTION 46)
- ☐ 1–6 times per year
- ☐ 7–11 times per year
- ☐ 1 time per month
- ☐ 2–3 times per month
- ☐ 1 time per week
- ☐ 2 times per week
- ☐ 3–4 times per week
- ☐ 5–6 times per week
- ☐ 1 time per day
- ☐ 2 or more times per day

45a. Each time you ate **onions**, how much did you usually eat?

- ☐ Less than 1 slice or less than 1 tablespoon
- ☐ 1 slice or 1 to 4 tablespoons
- ☐ More than 1 slice or more than 4 tablespoons

Question 46 appears in the next column

46. Now think about all the **cooked vegetables** you ate in the past 12 months and how they were prepared. How often were your vegetables **COOKED WITH** some sort of **fat**, including oil spray? *(Please do not include potatoes.)*

- ☐ NEVER (GO TO QUESTION 47)
- ☐ 1–6 times per year
- ☐ 7–11 times per year
- ☐ 1 time per month
- ☐ 2–3 times per month
- ☐ 1 time per week
- ☐ 2 times per week
- ☐ 3–4 times per week
- ☐ 5–6 times per week
- ☐ 1 time per day
- ☐ 2 or more times per day

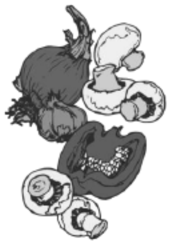

46a. Which fats were usually added to your vegetables **DURING COOKING**? *(Please do not include potatoes.* ***Mark all that apply.****)*

- ☐ Margarine (including low-fat)
- ☐ Butter (including low-fat)
- ☐ Lard, fatback, or bacon fat
- ☐ Olive oil
- ☐ Corn oil
- ☐ Canola or rapeseed oil
- ☐ Oil spray, such as Pam or others
- ☐ Other kinds of oils
- ☐ None of the above

47. Now, thinking again about all the **cooked vegetables** you ate in the past 12 months, how often was some sort of fat, sauce, or dressing added **AFTER COOKING OR AT THE TABLE**? *(Please do not include potatoes.)*

- ☐ NEVER (GO TO QUESTION 48)
- ☐ 1–6 times per year
- ☐ 7–11 times per year
- ☐ 1 time per month
- ☐ 2–3 times per month
- ☐ 1–2 times per week
- ☐ 3–4 times per week
- ☐ 5–6 times per week
- ☐ 1 time per day
- ☐ 2 times per day
- ☐ 3 or more times per day

Question 48 appears on the next page

This is a sample form. Do not use for scanning.

Over the past 12 months…

47a. Which fats, sauces, or dressings were usually added **AFTER COOKING OR AT THE TABLE**? *(Please do not include potatoes. **Mark all that apply.**)*

- ☐ Margarine (including low-fat)
- ☐ Butter (including low-fat)
- ☐ Lard, fatback, or bacon fat
- ☐ Salad dressing
- ☐ Cheese sauce
- ☐ White sauce
- ☐ Other

47b. If margarine, butter, lard, fatback, or bacon fat was added to your cooked vegetables **AFTER COOKING OR AT THE TABLE**, how much did you usually add?

- ☐ Did not usually add these
- ☐ Less than 1 teaspoon
- ☐ 1 to 3 teaspoons
- ☐ More than 3 teaspoons

47c. If salad dressing, cheese sauce, or white sauce was added to your cooked vegetables **AFTER COOKING OR AT THE TABLE**, how much did you usually add?

- ☐ Did not usually add these
- ☐ Less than 1 tablespoon
- ☐ 1 to 3 tablespoons
- ☐ More than 3 tablespoons

48. How often did you eat **sweet peppers** (green, red, or yellow)?

- ☐ NEVER (GO TO QUESTION 49)
- ☐ 1–6 times per year
- ☐ 7–11 times per year
- ☐ 1 time per month
- ☐ 2–3 times per month
- ☐ 1 time per week
- ☐ 2 times per week
- ☐ 3–4 times per week
- ☐ 5–6 times per week
- ☐ 1 time per day
- ☐ 2 or more times per day

48a. Each time you ate **sweet peppers**, how much did you usually eat?

- ☐ Less than ⅛ pepper
- ☐ ⅛ to ¼ pepper
- ☐ More than ¼ pepper

Question 49 appears in the next column

49. Over the past 12 months, did you eat **fresh tomatoes** (including those in salads)?

- ☐ NO (GO TO QUESTION 50)
- ☐ YES

49a. How often did you eat **fresh tomatoes** (including those in salads) **WHEN IN SEASON**?

- ☐ NEVER
- ☐ 1–6 times per season
- ☐ 7–11 times per season
- ☐ 1 time per month
- ☐ 2–3 times per month
- ☐ 1 time per week
- ☐ 2 times per week
- ☐ 3–4 times per week
- ☐ 5–6 times per week
- ☐ 1 time per day
- ☐ 2 or more times per day

49b. How often did you eat **fresh tomatoes** (including those in salads) **DURING THE REST OF THE YEAR**?

- ☐ NEVER
- ☐ 1–6 times per year
- ☐ 7–11 times per year
- ☐ 1 time per month
- ☐ 2–3 times per month
- ☐ 1 time per week
- ☐ 2 times per week
- ☐ 3–4 times per week
- ☐ 5–6 times per week
- ☐ 1 time per day
- ☐ 2 or more times per day

49c. Each time you ate **fresh tomatoes**, how much did you usually eat?

- ☐ Less than ¼ tomato
- ☐ ¼ to ½ tomato
- ☐ More than ½ tomato

50. How often did you eat **lettuce salads** (with or without other vegetables)?

- ☐ NEVER (GO TO QUESTION 51)
- ☐ 1–6 times per year
- ☐ 7–11 times per year
- ☐ 1 time per month
- ☐ 2–3 times per month
- ☐ 1 time per week
- ☐ 2 times per week
- ☐ 3–4 times per week
- ☐ 5–6 times per week
- ☐ 1 time per day
- ☐ 2 or more times per day

Question 51 appears on the next page

This is a sample form. Do not use for scanning.

Over the past 12 months…

50a. Each time you ate **lettuce salads**, how much did you usually eat?

- ☐ Less than ¼ cup
- ☐ ¼ to 1¼ cups
- ☐ More than 1¼ cups

50b. How often did the lettuce salads you ate include **dark green lettuce**?

- ☐ Almost never or never
- ☐ About ¼ of the time
- ☐ About ½ of the time
- ☐ About ¾ of the time
- ☐ Almost always or always

51. How often did you eat **salad dressing** (including low-fat) **on salads**?

☐ NEVER (GO TO QUESTION 52)

☐ 1–6 times per year	☐ 2 times per week
☐ 7–11 times per year	☐ 3–4 times per week
☐ 1 time per month	☐ 5–6 times per week
☐ 2–3 times per month	☐ 1 time per day
☐ 1 time per week	☐ 2 or more times per day

51a. Each time you ate **salad dressing on salads**, how much did you usually eat?

- ☐ Less than 2 tablespoons
- ☐ 2 to 4 tablespoons
- ☐ More than 4 tablespoons

52. How often did you eat **sweet potatoes** or **yams**?

☐ NEVER (GO TO QUESTION 53)

☐ 1–6 times per year	☐ 2 times per week
☐ 7–11 times per year	☐ 3–4 times per week
☐ 1 time per month	☐ 5–6 times per week
☐ 2–3 times per month	☐ 1 time per day
☐ 1 time per week	☐ 2 or more times per day

52a. Each time you ate **sweet potatoes** or **yams**, how much did you usually eat?

- ☐ 1 small potato or less than ¼ cup
- ☐ 1 medium potato or ¼ to ¾ cup
- ☐ 1 large potato or more than ¾ cup

Question 53 appears in the next column

53. How often did you eat **French fries**, **home fries**, **hash browned potatoes**, or **tater tots**?

☐ NEVER (GO TO QUESTION 54)

☐ 1–6 times per year	☐ 2 times per week
☐ 7–11 times per year	☐ 3–4 times per week
☐ 1 time per month	☐ 5–6 times per week
☐ 2–3 times per month	☐ 1 time per day
☐ 1 time per week	☐ 2 or more times per day

53a. Each time you ate **French fries**, **home fries**, **hash browned potatoes**, or **tater tots** how much did you usually eat?

- ☐ Less than 10 fries or less than ½ cup
- ☐ 10 to 25 fries or ½ to 1 cup
- ☐ More than 25 fries or more than 1 cup

54. How often did you eat **potato salad**?

☐ NEVER (GO TO QUESTION 55)

☐ 1–6 times per year	☐ 2 times per week
☐ 7–11 times per year	☐ 3–4 times per week
☐ 1 time per month	☐ 5–6 times per week
☐ 2–3 times per month	☐ 1 time per day
☐ 1 time per week	☐ 2 or more times per day

54a. Each time you ate **potato salad**, how much did you usually eat?

- ☐ Less than ½ cup
- ☐ ½ to 1 cup
- ☐ More than 1 cup

55. How often did you eat **baked**, **boiled**, or **mashed potatoes**?

☐ NEVER (GO TO QUESTION 56)

☐ 1–6 times per year	☐ 2 times per week
☐ 7–11 times per year	☐ 3–4 times per week
☐ 1 time per month	☐ 5–6 times per week
☐ 2–3 times per month	☐ 1 time per day
☐ 1 time per week	☐ 2 or more times per day

55a. Each time you ate **baked**, **boiled**, or **mashed potatoes**, how much did you usually eat?

- ☐ 1 small potato or less than ½ cup
- ☐ 1 medium potato or ½ to 1 cup
- ☐ 1 large potato or more than 1 cup

Question 56 appears on the next page

This is a sample form. Do not use for scanning.

Over the past 12 months...

55b. How often was **sour cream** (including low-fat) added to your potatoes, **EITHER IN COOKING OR AT THE TABLE**?

- ☐ Almost never or never (GO TO QUESTION 55d)
- ☐ About ¼ of the time
- ☐ About ½ of the time
- ☐ About ¾ of the time
- ☐ Almost always or always

55c. Each time **sour cream** was added to your potatoes, how much was usually added?

- ☐ Less than 1 tablespoon
- ☐ 1 to 3 tablespoons
- ☐ More than 3 tablespoons

55d. How often was **margarine** (including low-fat) added to your potatoes, **EITHER IN COOKING OR AT THE TABLE**?

- ☐ Almost never or never
- ☐ About ¼ of the time
- ☐ About ½ of the time
- ☐ About ¾ of the time
- ☐ Almost always or always

55e. How often was **butter** (including low-fat) added to your potatoes, **EITHER IN COOKING OR AT THE TABLE**?

- ☐ Almost never or never
- ☐ About ¼ of the time
- ☐ About ½ of the time
- ☐ About ¾ of the time
- ☐ Almost always or always

55f. Each time **margarine** or **butter** was added to your potatoes, how much was usually added?

- ☐ Never added
- ☐ Less than 1 teaspoon
- ☐ 1 to 3 teaspoons
- ☐ More than 3 teaspoons

55g. How often was **cheese** or **cheese sauce** added to your potatoes, **EITHER IN COOKING OR AT THE TABLE**?

- ☐ Almost never or never (GO TO QUESTION 56)
- ☐ About ¼ of the time
- ☐ About ½ of the time
- ☐ About ¾ of the time
- ☐ Almost always or always

Question 56 appears in the next column

55h. Each time **cheese** or **cheese sauce** was added to your potatoes, how much was usually added?

- ☐ Less than 1 tablespoon
- ☐ 1 to 3 tablespoons
- ☐ More than 3 tablespoons

56. How often did you eat **salsa**?

- ☐ NEVER (GO TO QUESTION 57)
- ☐ 1–6 times per year
- ☐ 7–11 times per year
- ☐ 1 time per month
- ☐ 2–3 times per month
- ☐ 1 time per week
- ☐ 2 times per week
- ☐ 3–4 times per week
- ☐ 5–6 times per week
- ☐ 1 time per day
- ☐ 2 or more times per day

56a. Each time you ate **salsa**, how much did you usually eat?

- ☐ Less than 1 tablespoon
- ☐ 1 to 5 tablespoons
- ☐ More than 5 tablespoons

57. How often did you eat **catsup**?

- ☐ NEVER (GO TO QUESTION 58)
- ☐ 1–6 times per year
- ☐ 7–11 times per year
- ☐ 1 time per month
- ☐ 2–3 times per month
- ☐ 1 time per week
- ☐ 2 times per week
- ☐ 3–4 times per week
- ☐ 5–6 times per week
- ☐ 1 time per day
- ☐ 2 or more times per day

57a. Each time you ate **catsup**, how much did you usually eat?

- ☐ Less than 1 teaspoon
- ☐ 1 to 6 teaspoons
- ☐ More than 6 teaspoons

58. How often did you eat **stuffing**, **dressing**, or **dumplings**?

- ☐ NEVER (GO TO QUESTION 59)
- ☐ 1–6 times per year
- ☐ 7–11 times per year
- ☐ 1 time per month
- ☐ 2–3 times per month
- ☐ 1 time per week
- ☐ 2 times per week
- ☐ 3–4 times per week
- ☐ 5–6 times per week
- ☐ 1 time per day
- ☐ 2 or more times per day

58a. Each time you ate **stuffing**, **dressing**, or **dumplings**, how much did you usually eat?

- ☐ Less than ½ cup
- ☐ ½ to 1 cup
- ☐ More than 1 cup

Question 59 appears on the next page

This is a sample form. Do not use for scanning.

Over the past 12 months…

59. How often did you eat **chili**?

- ☐ NEVER (GO TO QUESTION 60)
- ☐ 1–6 times per year
- ☐ 7–11 times per year
- ☐ 1 time per month
- ☐ 2–3 times per month
- ☐ 1 time per week
- ☐ 2 times per week
- ☐ 3–4 times per week
- ☐ 5–6 times per week
- ☐ 1 time per day
- ☐ 2 or more times per day

59a. Each time you ate **chili**, how much did you usually eat?

- ☐ Less than ½ cup
- ☐ ½ to 1¾ cups
- ☐ More than 1¾ cups

60. How often did you eat **Mexican foods** (such as tacos, tostados, burritos, tamales, fajitas, enchiladas, quesadillas, and chimichangas)?

- ☐ NEVER (GO TO QUESTION 61)
- ☐ 1–6 times per year
- ☐ 7–11 times per year
- ☐ 1 time per month
- ☐ 2–3 times per month
- ☐ 1 time per week
- ☐ 2 times per week
- ☐ 3–4 times per week
- ☐ 5–6 times per week
- ☐ 1 time per day
- ☐ 2 or more times per day

60a. Each time you ate **Mexican foods**, how much did you usually eat?

- ☐ Less than 1 taco, burrito, etc.
- ☐ 1 to 2 tacos, burritos, etc.
- ☐ More than 2 tacos, burritos, etc.

61. How often did you eat **cooked dried beans** (such as baked beans, pintos, kidney, blackeyed peas, lima, lentils, soybeans, or refried beans)? *(Please do not include bean soups or chili.)*

- ☐ NEVER (GO TO QUESTION 62)
- ☐ 1–6 times per year
- ☐ 7–11 times per year
- ☐ 1 time per month
- ☐ 2–3 times per month
- ☐ 1 time per week
- ☐ 2 times per week
- ☐ 3–4 times per week
- ☐ 5–6 times per week
- ☐ 1 time per day
- ☐ 2 or more times per day

61a. Each time you ate **beans**, how much did you usually eat?

- ☐ Less than ½ cup
- ☐ ½ to 1 cup
- ☐ More than 1 cup

Question 62 appears in the next column

61b. How often were the beans you ate **refried beans, beans prepared with any type of fat**, or **with meat added**?

- ☐ Almost never or never
- ☐ About ¼ of the time
- ☐ About ½ of the time
- ☐ About ¾ of the time
- ☐ Almost always or always

62. How often did you eat **other kinds of vegetables**?

- ☐ NEVER (GO TO QUESTION 63)
- ☐ 1–6 times per year
- ☐ 7–11 times per year
- ☐ 1 time per month
- ☐ 2–3 times per month
- ☐ 1 time per week
- ☐ 2 times per week
- ☐ 3–4 times per week
- ☐ 5–6 times per week
- ☐ 1 time per day
- ☐ 2 or more times per day

62a. Each time you ate **other kinds of vegetables**, how much did you usually eat?

- ☐ Less than ¼ cup
- ☐ ¼ to ½ cup
- ☐ More than ½ cup

63. How often did you eat **rice** or **other cooked grains** (such as bulgur, cracked wheat, or millet)?

- ☐ NEVER (GO TO QUESTION 64)
- ☐ 1–6 times per year
- ☐ 7–11 times per year
- ☐ 1 time per month
- ☐ 2–3 times per month
- ☐ 1 time per week
- ☐ 2 times per week
- ☐ 3–4 times per week
- ☐ 5–6 times per week
- ☐ 1 time per day
- ☐ 2 or more times per day

63a. Each time you ate **rice** or **other cooked grains**, how much did you usually eat?

- ☐ Less than ½ cup
- ☐ ½ to 1½ cups
- ☐ More than 1½ cups

63b. How often was **butter**, **margarine**, or **oil** added to your rice or other cooked grains **IN COOKING OR AT THE TABLE**?

- ☐ Almost never or never
- ☐ About ¼ of the time
- ☐ About ½ of the time
- ☐ About ¾ of the time
- ☐ Almost always or always

Question 64 appears on the next page

This is a sample form. Do not use for scanning.

Over the past 12 months...

64. How often did you eat **pancakes, waffles,** or **French toast**?

- ☐ NEVER (GO TO QUESTION 65)
- ☐ 1–6 times per year
- ☐ 7–11 times per year
- ☐ 1 time per month
- ☐ 2–3 times per month
- ☐ 1 time per week
- ☐ 2 times per week
- ☐ 3–4 times per week
- ☐ 5–6 times per week
- ☐ 1 time per day
- ☐ 2 or more times per day

64a. Each time you ate **pancakes, waffles,** or **French toast**, how much did you usually eat?

- ☐ Less than 1 medium piece
- ☐ 1 to 3 medium pieces
- ☐ More than 3 medium pieces

64b. How often was **margarine** (including low-fat) added to your pancakes, waffles, or French toast **AFTER COOKING OR AT THE TABLE**?

- ☐ Almost never or never
- ☐ About ¼ of the time
- ☐ About ½ of the time
- ☐ About ¾ of the time
- ☐ Almost always or always

64c. How often was **butter** (including low-fat) added to your pancakes, waffles, or French toast **AFTER COOKING OR AT THE TABLE**?

- ☐ Almost never or never
- ☐ About ¼ of the time
- ☐ About ½ of the time
- ☐ About ¾ of the time
- ☐ Almost always or always

64d. Each time **margarine** or **butter** was added to your pancakes, waffles, or French toast, how much was usually added?

- ☐ Never added
- ☐ Less than 1 teaspoon
- ☐ 1 to 3 teaspoons
- ☐ More than 3 teaspoons

64e. How often was **syrup** added to your pancakes, waffles, or French toast?

- ☐ Almost never or never (GO TO QUESTION 65)
- ☐ About ¼ of the time
- ☐ About ½ of the time
- ☐ About ¾ of the time
- ☐ Almost always or always

Question 65 appears in the next column

64f. Each time **syrup** was added to your pancakes, waffles, or French toast, how much was usually added?

- ☐ Less than 1 tablespoon
- ☐ 1 to 4 tablespoons
- ☐ More than 4 tablespoons

65. How often did you eat **lasagna, stuffed shells, stuffed manicotti, ravioli,** or **tortellini**? *(Please do not include spaghetti or other pasta.)*

- ☐ NEVER (GO TO QUESTION 66)
- ☐ 1–6 times per year
- ☐ 7–11 times per year
- ☐ 1 time per month
- ☐ 2–3 times per month
- ☐ 1 time per week
- ☐ 2 times per week
- ☐ 3–4 times per week
- ☐ 5–6 times per week
- ☐ 1 time per day
- ☐ 2 or more times per day

65a. Each time you ate **lasagna, stuffed shells, stuffed manicotti, ravioli,** or **tortellini**, how much did you usually eat?

- ☐ Less than 1 cup
- ☐ 1 to 2 cups
- ☐ More than 2 cups

66. How often did you eat **macaroni and cheese**?

- ☐ NEVER (GO TO QUESTION 67)
- ☐ 1–6 times per year
- ☐ 7–11 times per year
- ☐ 1 time per month
- ☐ 2–3 times per month
- ☐ 1 time per week
- ☐ 2 times per week
- ☐ 3–4 times per week
- ☐ 5–6 times per week
- ☐ 1 time per day
- ☐ 2 or more times per day

66a. Each time you ate **macaroni and cheese**, how much did you usually eat?

- ☐ Less than 1 cup
- ☐ 1 to 1½ cups
- ☐ More than 1½ cups

67. How often did you eat **pasta salad** or **macaroni salad**?

- ☐ NEVER (GO TO QUESTION 68)
- ☐ 1–6 times per year
- ☐ 7–11 times per year
- ☐ 1 time per month
- ☐ 2–3 times per month
- ☐ 1 time per week
- ☐ 2 times per week
- ☐ 3–4 times per week
- ☐ 5–6 times per week
- ☐ 1 time per day
- ☐ 2 or more times per day

Question 68 appears on the next page

This is a sample form. Do not use for scanning.

Over the past 12 months…

67a. Each time you ate **pasta salad** or **macaroni salad**, how much did you usually eat?

- ☐ Less than ½ cup
- ☐ ½ to 1 cup
- ☐ More than 1 cup

68. Other than the pastas listed in Questions 65, 66, and 67, how often did you eat **pasta, spaghetti**, or **other noodles**?

☐ NEVER (GO TO QUESTION 69)

- ☐ 1–6 times per year
- ☐ 7–11 times per year
- ☐ 1 time per month
- ☐ 2–3 times per month
- ☐ 1 time per week
- ☐ 2 times per week
- ☐ 3–4 times per week
- ☐ 5–6 times per week
- ☐ 1 time per day
- ☐ 2 or more times per day

68a. Each time you ate **pasta, spaghetti, or other noodles**, how much did you usually eat?

- ☐ Less than 1 cup
- ☐ 1 to 3 cups
- ☐ More than 3 cups

68b. How often did you eat your pasta, spaghetti, or other noodles with **tomato sauce** or **spaghetti sauce made WITH meat**?

- ☐ Almost never or never
- ☐ About ¼ of the time
- ☐ About ½ of the time
- ☐ About ¾ of the time
- ☐ Almost always or always

68c. How often did you eat your pasta, spaghetti, or other noodles with **tomato sauce** or **spaghetti sauce made WITHOUT meat**?

- ☐ Almost never or never
- ☐ About ¼ of the time
- ☐ About ½ of the time
- ☐ About ¾ of the time
- ☐ Almost always or always

68d. How often did you eat your pasta, spaghetti, or other noodles with **margarine, butter, oil**, or **cream sauce**?

- ☐ Almost never or never
- ☐ About ¼ of the time
- ☐ About ½ of the time
- ☐ About ¾ of the time
- ☐ Almost always or always

Question 69 appears in the next column

69. How often did you eat **bagels** or **English muffins**?

☐ NEVER (GO TO INTRODUCTION TO QUESTION 70)

- ☐ 1–6 times per year
- ☐ 7–11 times per year
- ☐ 1 time per month
- ☐ 2–3 times per month
- ☐ 1 time per week
- ☐ 2 times per week
- ☐ 3–4 times per week
- ☐ 5–6 times per week
- ☐ 1 time per day
- ☐ 2 or more times per day

69a. How often were the bagels or English muffins you ate **whole wheat**?

- ☐ Almost never or never
- ☐ About ¼ of the time
- ☐ About ½ of the time
- ☐ About ¾ of the time
- ☐ Almost always or always

69b. Each time you ate **bagels** or **English muffins**, how many did you usually eat?

- ☐ Less than 1 bagel or English muffin
- ☐ 1 bagel or English muffin
- ☐ More than 1 bagel or English muffin

69c. How often was **margarine** (including low-fat) added to your bagels or English muffins?

- ☐ Almost never or never
- ☐ About ¼ of the time
- ☐ About ½ of the time
- ☐ About ¾ of the time
- ☐ Almost always or always

69d. How often was **butter** (including low-fat) added to your bagels or English muffins?

- ☐ Almost never or never
- ☐ About ¼ of the time
- ☐ About ½ of the time
- ☐ About ¾ of the time
- ☐ Almost always or always

69e. Each time **margarine** or **butter** was added to your bagels or English muffins, how much was usually added?

- ☐ Never added
- ☐ Less than 1 teaspoon
- ☐ 1 to 2 teaspoons
- ☐ More than 2 teaspoons

Introduction to Question 70 appears on the next page

This is a sample form. Do not use for scanning.

Over the past 12 months...

69f. How often was **cream cheese** (including low-fat) spread on your bagels or English muffins?

- ☐ Almost never or never (GO TO INTRODUCTION TO QUESTION 70)
- ☐ About ¼ of the time
- ☐ About ½ of the time
- ☐ About ¾ of the time
- ☐ Almost always or always

69g. Each time **cream cheese** was added to your bagels or English muffins, how much was usually added?

- ☐ Less than 1 tablespoon
- ☐ 1 to 2 tablespoons
- ☐ More than 2 tablespoons

The next questions ask about your intake of breads other than bagels or English muffins. First, we will ask about bread you ate as part of sandwiches only. Then we will ask about all other bread you ate.

70. How often did you eat **breads** or **rolls AS PART OF SANDWICHES** (including burger and hot dog rolls)?
(Please do not include fast food sandwiches.)

- ☐ NEVER (GO TO QUESTION 71)

- ☐ 1–6 times per year
- ☐ 7–11 times per year
- ☐ 1 time per month
- ☐ 2–3 times per month
- ☐ 1 time per week
- ☐ 2 times per week
- ☐ 3–4 times per week
- ☐ 5–6 times per week
- ☐ 1 time per day
- ☐ 2 or more times per day

70a. Each time you ate **breads** or **rolls AS PART OF SANDWICHES**, how many did you usually eat?

- ☐ 1 slice or ½ roll
- ☐ 2 slices or 1 roll
- ☐ More than 2 slices or more than 1 roll

70b. How often were the breads or rolls that you used for your sandwiches **white bread** (including burger and hot dog rolls)?

- ☐ Almost never or never
- ☐ About ¼ of the time
- ☐ About ½ of the time
- ☐ About ¾ of the time
- ☐ Almost always or always

Question 71 appears in the next column

70c. How often was **mayonnaise** or **mayonnaise-type dressing** (including low-fat) added to the breads or rolls used for your sandwiches?

- ☐ Almost never or never (GO TO QUESTION 70e)
- ☐ About ¼ of the time
- ☐ About ½ of the time
- ☐ About ¾ of the time
- ☐ Almost always or always

70d. Each time **mayonnaise** or **mayonnaise-type dressing** was added to the breads or rolls used for your sandwiches, how much was usually added?

- ☐ Less than 1 teaspoon
- ☐ 1 to 3 teaspoons
- ☐ More than 3 teaspoons

70e. How often was **margarine** (including low-fat) added to the breads or rolls used for your sandwiches?

- ☐ Almost never or never
- ☐ About ¼ of the time
- ☐ About ½ of the time
- ☐ About ¾ of the time
- ☐ Almost always or always

70f. How often was **butter** (including low-fat) added to the breads or rolls used for your sandwiches?

- ☐ Almost never or never
- ☐ About ¼ of the time
- ☐ About ½ of the time
- ☐ About ¾ of the time
- ☐ Almost always or always

70g. Each time **margarine** or **butter** was added to the breads or rolls used for your sandwiches, how much was usually added?

- ☐ Never added
- ☐ Less than 1 teaspoon
- ☐ 1 to 2 teaspoons
- ☐ More than 2 teaspoons

71. How often did you eat **breads** or **dinner rolls**, **NOT AS PART OF SANDWICHES**?

- ☐ NEVER (GO TO QUESTION 72)

- ☐ 1–6 times per year
- ☐ 7–11 times per year
- ☐ 1 time per month
- ☐ 2–3 times per month
- ☐ 1 time per week
- ☐ 2 times per week
- ☐ 3–4 times per week
- ☐ 5–6 times per week
- ☐ 1 time per day
- ☐ 2 or more times per day

Question 72 appears on the next page

This is a sample form. Do not use for scanning.

Over the past 12 months…

71a. Each time you ate **breads** or **dinner rolls, NOT AS PART OF SANDWICHES**, how much did you usually eat?

- ☐ 1 slice or 1 dinner roll
- ☐ 2 slices or 2 dinner rolls
- ☐ More than 2 slices or 2 dinner rolls

71b. How often were the breads or rolls you ate **white bread**?

- ☐ Almost never or never
- ☐ About ¼ of the time
- ☐ About ½ of the time
- ☐ About ¾ of the time
- ☐ Almost always or always

71c. How often was **margarine** (including low-fat) added to your breads or rolls?

- ☐ Almost never or never
- ☐ About ¼ of the time
- ☐ About ½ of the time
- ☐ About ¾ of the time
- ☐ Almost always or always

71d. How often was **butter** (including low-fat) added to your breads or rolls?

- ☐ Almost never or never
- ☐ About ¼ of the time
- ☐ About ½ of the time
- ☐ About ¾ of the time
- ☐ Almost always or always

71e. Each time **margarine** or **butter** was added to your breads or rolls, how much was usually added?

- ☐ Never added
- ☐ Less than 1 teaspoon
- ☐ 1 to 2 teaspoons
- ☐ More than 2 teaspoons

71f. How often was **cream cheese** (including low-fat) added to your breads or rolls?

- ☐ Almost never or never (GO TO QUESTION 72)
- ☐ About ¼ of the time
- ☐ About ½ of the time
- ☐ About ¾ of the time
- ☐ Almost always or always

Question 72 appears in the next column

71g. Each time **cream cheese** was added to your breads or rolls, how much was usually added?

- ☐ Less than 1 tablespoon
- ☐ 1 to 2 tablespoons
- ☐ More than 2 tablespoons

72. How often did you eat **jam, jelly**, or **honey** on bagels, muffins, bread, rolls, or crackers?

- ☐ NEVER (GO TO QUESTION 73)
- ☐ 1–6 times per year
- ☐ 7–11 times per year
- ☐ 1 time per month
- ☐ 2–3 times per month
- ☐ 1 time per week
- ☐ 2 times per week
- ☐ 3–4 times per week
- ☐ 5–6 times per week
- ☐ 1 time per day
- ☐ 2 or more times per day

72a. Each time you ate **jam, jelly**, or **honey**, how much did you usually eat?

- ☐ Less than 1 teaspoon
- ☐ 1 to 3 teaspoons
- ☐ More than 3 teaspoons

73. How often did you eat **peanut butter** or **other nut butter**?

- ☐ NEVER (GO TO QUESTION 74)
- ☐ 1–6 times per year
- ☐ 7–11 times per year
- ☐ 1 time per month
- ☐ 2–3 times per month
- ☐ 1 time per week
- ☐ 2 times per week
- ☐ 3–4 times per week
- ☐ 5–6 times per week
- ☐ 1 time per day
- ☐ 2 or more times per day

73a. Each time you ate **peanut butter** or **other nut butter**, how much did you usually eat?

- ☐ Less than 1 tablespoon
- ☐ 1 to 2 tablespoons
- ☐ More than 2 tablespoons

74. How often did you eat **roast beef** or **steak IN SANDWICHES**?

- ☐ NEVER (GO TO QUESTION 75)
- ☐ 1–6 times per year
- ☐ 7–11 times per year
- ☐ 1 time per month
- ☐ 2–3 times per month
- ☐ 1 time per week
- ☐ 2 times per week
- ☐ 3–4 times per week
- ☐ 5–6 times per week
- ☐ 1 time per day
- ☐ 2 or more times per day

Question 75 appears on the next page

This is a sample form. Do not use for scanning.

Over the <u>past 12 months</u>...

74a. Each time you ate **roast beef** or **steak IN SANDWICHES**, how much did you usually eat?

- ☐ Less than 1 slice or less than 2 ounces
- ☐ 1 to 2 slices or 2 to 4 ounces
- ☐ More than 2 slices or more than 4 ounces

75. How often did you eat **turkey** or **chicken COLD CUTS** (such as loaf, luncheon meat, turkey ham, turkey salami, or turkey pastrami)? *(We will ask about other turkey or chicken later.)*

- ☐ NEVER (GO TO QUESTION 76)

☐ 1–6 times per year	☐ 2 times per week
☐ 7–11 times per year	☐ 3–4 times per week
☐ 1 time per month	☐ 5–6 times per week
☐ 2–3 times per month	☐ 1 time per day
☐ 1 time per week	☐ 2 or more times per day

75a. Each time you ate **turkey or chicken COLD CUTS**, how much did you usually eat?

- ☐ Less than 1 slice
- ☐ 1 to 3 slices
- ☐ More than 3 slices

76. How often did you eat **luncheon** or **deli-style ham**? *(We will ask about other ham later.)*

- ☐ NEVER (GO TO QUESTION 77)

☐ 1–6 times per year	☐ 2 times per week
☐ 7–11 times per year	☐ 3–4 times per week
☐ 1 time per month	☐ 5–6 times per week
☐ 2–3 times per month	☐ 1 time per day
☐ 1 time per week	☐ 2 or more times per day

76a. Each time you ate **luncheon** or **deli-style ham**, how much did you usually eat?

- ☐ Less than 1 slice
- ☐ 1 to 3 slices
- ☐ More than 3 slices

76b. How often was the luncheon or deli-style ham you ate **light**, **low-fat**, or **fat-free**?

- ☐ Almost never or never
- ☐ About ¼ of the time
- ☐ About ½ of the time
- ☐ About ¾ of the time
- ☐ Almost always or always

Question 77 appears in the next column

77. How often did you eat **other cold cuts** or **luncheon meats** (such as bologna, salami, corned beef, pastrami, or others, including low-fat)? *(Please do not include ham, turkey, or chicken cold cuts.)*

- ☐ NEVER (GO TO QUESTION 78)

☐ 1–6 times per year	☐ 2 times per week
☐ 7–11 times per year	☐ 3–4 times per week
☐ 1 time per month	☐ 5–6 times per week
☐ 2–3 times per month	☐ 1 time per day
☐ 1 time per week	☐ 2 or more times per day

77a. Each time you ate **other cold cuts** or **luncheon meats**, how much did you usually eat?

- ☐ Less than 1 slice
- ☐ 1 to 3 slices
- ☐ More than 3 slices

77b. How often were the other cold cuts or luncheon meats you ate **light**, **low-fat**, or **fat-free**? *(Please do not include ham, turkey, or chicken cold cuts.)*

- ☐ Almost never or never
- ☐ About ¼ of the time
- ☐ About ½ of the time
- ☐ About ¾ of the time
- ☐ Almost always or always

78. How often did you eat **canned tuna** (including in salads, sandwiches, or casseroles)?

- ☐ NEVER (GO TO QUESTION 79)

☐ 1–6 times per year	☐ 2 times per week
☐ 7–11 times per year	☐ 3–4 times per week
☐ 1 time per month	☐ 5–6 times per week
☐ 2–3 times per month	☐ 1 time per day
☐ 1 time per week	☐ 2 or more times per day

78a. Each time you ate **canned tuna**, how much did you usually eat?

- ☐ Less than ¼ cup or less than 2 ounces
- ☐ ¼ to ½ cup or 2 to 3 ounces
- ☐ More than ½ cup or more than 3 ounces

78b. How often was the canned tuna you ate **water-packed**?

- ☐ Almost never or never
- ☐ About ¼ of the time
- ☐ About ½ of the time
- ☐ About ¾ of the time
- ☐ Almost always or always

Question 79 appears on the next page

This is a sample form. Do not use for scanning.

Over the past 12 months…

78c. How often was the canned tuna you ate **prepared with mayonnaise** or **other dressing** (including low-fat)?

- ☐ Almost never or never
- ☐ About ¼ of the time
- ☐ About ½ of the time
- ☐ About ¾ of the time
- ☐ Almost always or always

79. How often did you eat **GROUND chicken** or **turkey**? *(We will ask about other chicken and turkey later.)*

- ☐ NEVER (GO TO QUESTION 80)

- ☐ 1–6 times per year
- ☐ 7–11 times per year
- ☐ 1 time per month
- ☐ 2–3 times per month
- ☐ 1 time per week
- ☐ 2 times per week
- ☐ 3–4 times per week
- ☐ 5–6 times per week
- ☐ 1 time per day
- ☐ 2 or more times per day

79a. Each time you ate **GROUND chicken** or **turkey**, how much did you usually eat?

- ☐ Less than 2 ounces or less than ½ cup
- ☐ 2 to 4 ounces or ½ to 1 cup
- ☐ More than 4 ounces or more than 1 cup

80. How often did you eat **beef hamburgers** or **cheeseburgers** from a **FAST FOOD** or **OTHER RESTAURANT**?

- ☐ NEVER (GO TO QUESTION 81)

- ☐ 1–6 times per year
- ☐ 7–11 times per year
- ☐ 1 time per month
- ☐ 2–3 times per month
- ☐ 1 time per week
- ☐ 2 times per week
- ☐ 3–4 times per week
- ☐ 5–6 times per week
- ☐ 1 time per day
- ☐ 2 or more times per day

80a. Each time you ate **beef hamburgers** or **cheeseburgers** from a **FAST FOOD** or **OTHER RESTAURANT**, what size did you usually eat?

- ☐ Small hamburger (such as a regular Burger King or McDonald's Hamburger)
- ☐ Medium (such as McDonald's or Burger King Double Burger or Cheeseburger)
- ☐ Large (such as Burger King Whopper or Double Whopper or a McDonald's Double Quarter Pounder)

Question 81 appears in the next column

80b. Each time you ate **beef hamburgers** or **cheeseburgers** from a **FAST FOOD** or **OTHER RESTAURANT**, how much did you usually eat?

- ☐ Less than 1 burger
- ☐ 1 burger
- ☐ More than 1 burger

80c. How often did you have **cheeseburgers** rather than **hamburgers**?

- ☐ Almost never or never
- ☐ About ¼ of the time
- ☐ About ½ of the time
- ☐ About ¾ of the time
- ☐ Almost always or always

81. How often did you eat **beef hamburgers** or **cheeseburgers** that were **NOT FROM A FAST FOOD** or **OTHER RESTAURANT**?

- ☐ NEVER (GO TO QUESTION 82)

- ☐ 1–6 times per year
- ☐ 7–11 times per year
- ☐ 1 time per month
- ☐ 2–3 times per month
- ☐ 1 time per week
- ☐ 2 times per week
- ☐ 3–4 times per week
- ☐ 5–6 times per week
- ☐ 1 time per day
- ☐ 2 or more times per day

81a. Each time you ate **beef hamburgers** or **cheeseburgers** that were **NOT FROM A FAST FOOD** or **OTHER RESTAURANT**, how much did you usually eat?

- ☐ Less than 1 patty or less than 2 ounces
- ☐ 1 patty or 2 to 4 ounces
- ☐ More than 1 patty or more than 4 ounces

81b. How often were these beef hamburgers or cheeseburgers made with **lean ground beef**?

- ☐ Almost never or never
- ☐ About ¼ of the time
- ☐ About ½ of the time
- ☐ About ¾ of the time
- ☐ Almost always or always

82. How often did you eat **ground beef in mixtures** (such as meatballs, casseroles, chili, or meatloaf)?

- ☐ NEVER (GO TO QUESTION 83)

- ☐ 1–6 times per year
- ☐ 7–11 times per year
- ☐ 1 time per month
- ☐ 2–3 times per month
- ☐ 1 time per week
- ☐ 2 times per week
- ☐ 3–4 times per week
- ☐ 5–6 times per week
- ☐ 1 time per day
- ☐ 2 or more times per day

Question 83 appears on the next page

This is a sample form. Do not use for scanning.

Over the past 12 months...

82a. Each time you ate **ground beef in mixtures**, how much did you usually eat?

- ☐ Less than 3 ounces or less than ½ cup
- ☐ 3 to 8 ounces or ½ to 1 cup
- ☐ More than 8 ounces or more than 1 cup

83. How often did you eat **hot dogs** or **frankfurters**? *(Please do not include sausages or vegetarian hot dogs.)*

☐ NEVER (GO TO QUESTION 84)

☐ 1–6 times per year	☐ 2 times per week
☐ 7–11 times per year	☐ 3–4 times per week
☐ 1 time per month	☐ 5–6 times per week
☐ 2–3 times per month	☐ 1 time per day
☐ 1 time per week	☐ 2 or more times per day

83a. Each time you ate **hot dogs** or **frankfurters**, how many did you usually eat?

- ☐ Less than 1 hot dog
- ☐ 1 to 2 hot dogs
- ☐ More than 2 hot dogs

83b. How often were the hot dogs or frankfurters you ate **light** or **low-fat**?

- ☐ Almost never or never
- ☐ About ¼ of the time
- ☐ About ½ of the time
- ☐ About ¾ of the time
- ☐ Almost always or always

84. How often did you eat **beef mixtures** (such as beef stew, beef pot pie, beef and noodles, or beef and vegetables)?

☐ NEVER (GO TO QUESTION 85)

☐ 1–6 times per year	☐ 2 times per week
☐ 7–11 times per year	☐ 3–4 times per week
☐ 1 time per month	☐ 5–6 times per week
☐ 2–3 times per month	☐ 1 time per day
☐ 1 time per week	☐ 2 or more times per day

84a. Each time you ate **beef mixtures**, how much did you usually eat?

- ☐ Less than 1 cup
- ☐ 1 to 2 cups
- ☐ More than 2 cups

Question 85 appears in the next column

85. How often did you eat **roast beef** or **pot roast**? *(Please do not include roast beef or pot roast in sandwiches.)*

☐ NEVER (GO TO QUESTION 86)

☐ 1–6 times per year	☐ 2 times per week
☐ 7–11 times per year	☐ 3–4 times per week
☐ 1 time per month	☐ 5–6 times per week
☐ 2–3 times per month	☐ 1 time per day
☐ 1 time per week	☐ 2 or more times per day

85a. Each time you ate **roast beef** or **pot roast**, how much did you usually eat?

- ☐ Less than 2 ounces
- ☐ 2 to 5 ounces
- ☐ More than 5 ounces

86. How often did you eat **steak** (beef)? *(Please do not include steak in sandwiches)*

☐ NEVER (GO TO QUESTION 87)

☐ 1–6 times per year	☐ 2 times per week
☐ 7–11 times per year	☐ 3–4 times per week
☐ 1 time per month	☐ 5–6 times per week
☐ 2–3 times per month	☐ 1 time per day
☐ 1 time per week	☐ 2 or more times per day

86a. Each time you ate **steak** (beef), how much did you usually eat?

- ☐ Less than 3 ounces
- ☐ 3 to 7 ounces
- ☐ More than 7 ounces

86b. How often was the steak you ate **lean steak**?

- ☐ Almost never or never
- ☐ About ¼ of the time
- ☐ About ½ of the time
- ☐ About ¾ of the time
- ☐ Almost always or always

87. How often did you eat **pork** or **beef spareribs**?

☐ NEVER (GO TO QUESTION 88)

☐ 1–6 times per year	☐ 2 times per week
☐ 7–11 times per year	☐ 3–4 times per week
☐ 1 time per month	☐ 5–6 times per week
☐ 2–3 times per month	☐ 1 time per day
☐ 1 time per week	☐ 2 or more times per day

Question 88 appears on the next page

This is a sample form. Do not use for scanning.

Over the past 12 months…

87a. Each time you ate **pork** or **beef spareribs**, how much did you usually eat?

☐ Less than 4 ribs
☐ 4 to 12 ribs
☐ More than 12 ribs

88. How often did you eat **roast turkey, turkey cutlets**, or **turkey nuggets** (including in sandwiches)?

☐ NEVER (GO TO QUESTION 89)

☐ 1–6 times per year
☐ 7–11 times per year
☐ 1 time per month
☐ 2–3 times per month
☐ 1 time per week
☐ 2 times per week
☐ 3–4 times per week
☐ 5–6 times per week
☐ 1 time per day
☐ 2 or more times per day

88a. Each time you ate **roast turkey, turkey cutlets**, or **turkey nuggets**, how much did you usually eat? *(Please note: 4 to 8 turkey nuggets = 3 ounces.)*

☐ Less than 2 ounces
☐ 2 to 4 ounces
☐ More than 4 ounces

89. How often did you eat **chicken mixtures** (such as salads, sandwiches, casseroles, stews, or other mixtures)?

☐ NEVER (GO TO QUESTION 90)

☐ 1–6 times per year
☐ 7–11 times per year
☐ 1 time per month
☐ 2–3 times per month
☐ 1 time per week
☐ 2 times per week
☐ 3–4 times per week
☐ 5–6 times per week
☐ 1 time per day
☐ 2 or more times per day

89a. Each time you ate **chicken mixtures**, how much did you usually eat?

☐ Less than ½ cup
☐ ½ to 1½ cups
☐ More than 1½ cups

Question 90 appears in the next column

90. How often did you eat **baked, broiled, roasted, stewed**, or **fried chicken** (including nuggets)? *(Please do not include chicken in mixtures.)*

☐ NEVER (GO TO QUESTION 91)

☐ 1–6 times per year
☐ 7–11 times per year
☐ 1 time per month
☐ 2–3 times per month
☐ 1 time per week
☐ 2 times per week
☐ 3–4 times per week
☐ 5–6 times per week
☐ 1 time per day
☐ 2 or more times per day

90a. Each time you ate **baked, broiled, roasted, stewed**, or **fried chicken** (including nuggets), how much did you usually eat?

☐ Less than 2 drumsticks or wings, less than 1 breast or thigh, or less than 4 nuggets
☐ 2 drumsticks or wings, 1 breast or thigh, or 4 to 8 nuggets
☐ More than 2 drumsticks or wings, more than 1 breast or thigh, or more than 8 nuggets

90b. How often was the chicken you ate **fried chicken** (including deep fried) or **chicken nuggets**?

☐ Almost never or never
☐ About ¼ of the time
☐ About ½ of the time
☐ About ¾ of the time
☐ Almost always or always

90c. How often was the chicken you ate **WHITE meat**?

☐ Almost never or never
☐ About ¼ of the time
☐ About ½ of the time
☐ About ¾ of the time
☐ Almost always or always

90d. How often did you eat chicken **WITH skin**?

☐ Almost never or never
☐ About ¼ of the time
☐ About ½ of the time
☐ About ¾ of the time
☐ Almost always or always

91. How often did you eat **baked ham** or **ham steak**?

☐ NEVER (GO TO QUESTION 92)

☐ 1–6 times per year
☐ 7–11 times per year
☐ 1 time per month
☐ 2–3 times per month
☐ 1 time per week
☐ 2 times per week
☐ 3–4 times per week
☐ 5–6 times per week
☐ 1 time per day
☐ 2 or more times per day

Question 92 appears on the next page

This is a sample form. Do not use for scanning.

Over the past 12 months…

91a. Each time you ate **baked ham** or **ham steak**, how much did you usually eat?

- ☐ Less than 1 ounce
- ☐ 1 to 3 ounces
- ☐ More than 3 ounces

92. How often did you eat **pork** (including chops, roasts, and in mixed dishes)? *(Please do not include ham, ham steak, or sausage.)*

- ☐ NEVER (GO TO QUESTION 93)

- ☐ 1–6 times per year
- ☐ 7–11 times per year
- ☐ 1 time per month
- ☐ 2–3 times per month
- ☐ 1 time per week
- ☐ 2 times per week
- ☐ 3–4 times per week
- ☐ 5–6 times per week
- ☐ 1 time per day
- ☐ 2 or more times per day

92a. Each time you ate **pork**, how much did you usually eat?

- ☐ Less than 2 ounces or less than 1 chop
- ☐ 2 to 5 ounces or 1 chop
- ☐ More than 5 ounces or more than 1 chop

93. How often did you eat **gravy** on meat, chicken, potatoes, rice, etc.?

- ☐ NEVER (GO TO QUESTION 94)

- ☐ 1–6 times per year
- ☐ 7–11 times per year
- ☐ 1 time per month
- ☐ 2–3 times per month
- ☐ 1 time per week
- ☐ 2 times per week
- ☐ 3–4 times per week
- ☐ 5–6 times per week
- ☐ 1 time per day
- ☐ 2 or more times per day

93a. Each time you ate **gravy** on meat, chicken, potatoes, rice, etc., how much did you usually eat?

- ☐ Less than ⅛ cup
- ☐ ⅛ to ½ cup
- ☐ More than ½ cup

94. How often did you eat **liver** (all kinds) or **liverwurst**?

- ☐ NEVER (GO TO QUESTION 95)

- ☐ 1–6 times per year
- ☐ 7–11 times per year
- ☐ 1 time per month
- ☐ 2–3 times per month
- ☐ 1 time per week
- ☐ 2 times per week
- ☐ 3–4 times per week
- ☐ 5–6 times per week
- ☐ 1 time per day
- ☐ 2 or more times per day

Question 95 appears in the next column

94a. Each time you ate **liver** or **liverwurst**, how much did you usually eat?

- ☐ Less than 1 ounce
- ☐ 1 to 4 ounces
- ☐ More than 4 ounces

95. How often did you eat **bacon** (including low-fat)?

- ☐ NEVER (GO TO QUESTION 96)

- ☐ 1–6 times per year
- ☐ 7–11 times per year
- ☐ 1 time per month
- ☐ 2–3 times per month
- ☐ 1 time per week
- ☐ 2 times per week
- ☐ 3–4 times per week
- ☐ 5–6 times per week
- ☐ 1 time per day
- ☐ 2 or more times per day

95a. Each time you ate **bacon**, how much did you usually eat?

- ☐ Fewer than 2 slices
- ☐ 2 to 3 slices
- ☐ More than 3 slices

95b. How often was the bacon you ate **light**, **low-fat**, or **lean**?

- ☐ Almost never or never
- ☐ About ¼ of the time
- ☐ About ½ of the time
- ☐ About ¾ of the time
- ☐ Almost always or always

96. How often did you eat **sausage** (including low-fat)?

- ☐ NEVER (GO TO QUESTION 97)

- ☐ 1–6 times per year
- ☐ 7–11 times per year
- ☐ 1 time per month
- ☐ 2–3 times per month
- ☐ 1 time per week
- ☐ 2 times per week
- ☐ 3–4 times per week
- ☐ 5–6 times per week
- ☐ 1 time per day
- ☐ 2 or more times per day

96a. Each time you ate **sausage**, how much did you usually eat?

- ☐ Less than 1 patty or 2 links
- ☐ 1 to 3 patties or 2 to 5 links
- ☐ More than 3 patties or 5 links

96b. How often was the sausage you ate **light**, **low-fat**, or **lean**?

- ☐ Almost never or never
- ☐ About ¼ of the time
- ☐ About ½ of the time
- ☐ About ¾ of the time
- ☐ Almost always or always

Question 97 appears on the next page

This is a sample form. Do not use for scanning.

Over the past 12 months…

97. How often did you eat **fried shellfish** (such as crab, lobster, shrimp)?

- ☐ NEVER (GO TO QUESTION 98)
- ☐ 1–6 times per year
- ☐ 7–11 times per year
- ☐ 1 time per month
- ☐ 2–3 times per month
- ☐ 1 time per week
- ☐ 2 times per week
- ☐ 3–4 times per week
- ☐ 5–6 times per week
- ☐ 1 time per day
- ☐ 2 or more times per day

97a. Each time you ate **fried shellfish**, how much did you usually eat?

- ☐ Less than 2 ounces
- ☐ 2 to 4 ounces
- ☐ More than 4 ounces

98. How often did you eat **shellfish** (such as crab, lobster, shrimp) **that was NOT FRIED**?

- ☐ NEVER (GO TO QUESTION 99)
- ☐ 1–6 times per year
- ☐ 7–11 times per year
- ☐ 1 time per month
- ☐ 2–3 times per month
- ☐ 1 time per week
- ☐ 2 times per week
- ☐ 3–4 times per week
- ☐ 5–6 times per week
- ☐ 1 time per day
- ☐ 2 or more times per day

98a. Each time you ate **shellfish that was NOT FRIED**, how much did you usually eat?

- ☐ Less than 1 ounce
- ☐ 1 to 4 ounces
- ☐ More than 4 ounces

99. How often did you eat **salmon, fresh tuna or trout**?

- ☐ NEVER (GO TO QUESTION 100)
- ☐ 1–6 times per year
- ☐ 7–11 times per year
- ☐ 1 time per month
- ☐ 2–3 times per month
- ☐ 1 time per week
- ☐ 2 times per week
- ☐ 3–4 times per week
- ☐ 5–6 times per week
- ☐ 1 time per day
- ☐ 2 or more times per day

99a. Each time you ate **salmon, fresh tuna or trout**, how much did you usually eat?

- ☐ Less than 2 ounces
- ☐ 2 to 6 ounces
- ☐ More than 6 ounces

Question 100 appears in the next column

100. How often did you eat **fish sticks** or other **fried fish** (not including shellfish)?

- ☐ NEVER (GO TO QUESTION 101)
- ☐ 1–6 times per year
- ☐ 7–11 times per year
- ☐ 1 time per month
- ☐ 2–3 times per month
- ☐ 1 time per week
- ☐ 2 times per week
- ☐ 3–4 times per week
- ☐ 5–6 times per week
- ☐ 1 time per day
- ☐ 2 or more times per day

100a. Each time you ate **fish sticks** or **other fried fish**, how much did you usually eat?

- ☐ Less than 2 ounces or less than 1 fillet
- ☐ 2 to 7 ounces or 1 fillet
- ☐ More than 7 ounces or more than 1 fillet

101. How often did you eat **other fish that was NOT FRIED** (not including shellfish)?

- ☐ NEVER (GO TO INTRODUCTION TO QUESTION 102)
- ☐ 1–6 times per year
- ☐ 7–11 times per year
- ☐ 1 time per month
- ☐ 2–3 times per month
- ☐ 1 time per week
- ☐ 2 times per week
- ☐ 3–4 times per week
- ☐ 5–6 times per week
- ☐ 1 time per day
- ☐ 2 or more times per day

101a. Each time you ate **other fish that was NOT FRIED**, how much did you usually eat?

- ☐ Less than 2 ounces or less than 1 fillet
- ☐ 2 to 5 ounces or 1 fillet
- ☐ More than 5 ounces or more than 1 fillet

Now think about all the meat, poultry, and fish you ate in the past 12 months and how they were prepared.

102. How often was **oil, butter, margarine, or other fat** used to **FRY, SAUTE, BASTE, OR MARINATE** any meat, poultry, or fish you ate? *(Please do not include deep frying.)*

- ☐ NEVER (GO TO QUESTION 103)
- ☐ 1–6 times per year
- ☐ 7–11 times per year
- ☐ 1 time per month
- ☐ 2–3 times per month
- ☐ 1 time per week
- ☐ 2 times per week
- ☐ 3–4 times per week
- ☐ 5–6 times per week
- ☐ 1 time per day
- ☐ 2 or more times per day

Question 103 appears on the next page

This is a sample form. Do not use for scanning.

Over the past 12 months…

102a. Which of the following **fats** were regularly used to prepare your meat, poultry, or fish? ***(Mark all that apply.)***

- ☐ Margarine (including low-fat)
- ☐ Butter (including low-fat)
- ☐ Lard, fatback, or bacon fat
- ☐ Olive oil
- ☐ Corn oil
- ☐ Canola or rapeseed oil
- ☐ Oil spray (such as Pam or others)
- ☐ Other kinds of oils
- ☐ None of the above

103. How often did you eat **tofu, soy burgers,** or **soy meat-substitutes**?

- ☐ NEVER (GO TO QUESTION 104)
- ☐ 1–6 times per year
- ☐ 7–11 times per year
- ☐ 1 time per month
- ☐ 2–3 times per month
- ☐ 1 time per week
- ☐ 2 times per week
- ☐ 3–4 times per week
- ☐ 5–6 times per week
- ☐ 1 time per day
- ☐ 2 or more times per day

103a. Each time you ate **tofu, soy burgers,** or **soy meat-substitutes**, how much did you usually eat?

- ☐ Less than ¼ cup or less than 2 ounces
- ☐ ¼ to ½ cup or 2 to 4 ounces
- ☐ More than ½ cup or more than 4 ounces

104. Over the past 12 months, did you eat **soups**?

- ☐ NO (GO TO QUESTION 105)
- ☐ YES

104a. How often did you eat **soup IN THE WINTER**?

- ☐ NEVER
- ☐ 1–6 times per winter
- ☐ 7–11 times per winter
- ☐ 1 time per month
- ☐ 2–3 times per month
- ☐ 1 time per week
- ☐ 2 times per week
- ☐ 3–4 times per week
- ☐ 5–6 times per week
- ☐ 1 time per day
- ☐ 2 or more times per day

104b. How often did you eat **soup DURING THE REST OF THE YEAR**?

- ☐ NEVER
- ☐ 1–6 times per year
- ☐ 7–11 times per year
- ☐ 1 time per month
- ☐ 2–3 times per month
- ☐ 1 time per week
- ☐ 2 times per week
- ☐ 3–4 times per week
- ☐ 5–6 times per week
- ☐ 1 time per day
- ☐ 2 or more times per day

Question 105 appears in the next column

104c. Each time you ate **soup**, how much did you usually eat?

- ☐ Less than 1 cup
- ☐ 1 to 2 cups
- ☐ More than 2 cups

104d. How often were the soups you ate **bean soups**?

- ☐ Almost never or never
- ☐ About ¼ of the time
- ☐ About ½ of the time
- ☐ About ¾ of the time
- ☐ Almost always or always

104e. How often were the soups you ate **cream soups** (including chowders)?

- ☐ Almost never or never
- ☐ About ¼ of the time
- ☐ About ½ of the time
- ☐ About ¾ of the time
- ☐ Almost always or always

104f. How often were the soups you ate **tomato** or **vegetable soups**?

- ☐ Almost never or never
- ☐ About ¼ of the time
- ☐ About ½ of the time
- ☐ About ¾ of the time
- ☐ Almost always or always

104g. How often were the soups you ate **broth soups** (including chicken) **with** or **without noodles** or **rice**?

- ☐ Almost never or never
- ☐ About ¼ of the time
- ☐ About ½ of the time
- ☐ About ¾ of the time
- ☐ Almost always or always

105. How often did you eat **pizza**?

- ☐ NEVER (GO TO QUESTION 106)
- ☐ 1–6 times per year
- ☐ 7–11 times per year
- ☐ 1 time per month
- ☐ 2–3 times per month
- ☐ 1 time per week
- ☐ 2 times per week
- ☐ 3–4 times per week
- ☐ 5–6 times per week
- ☐ 1 time per day
- ☐ 2 or more times per day

Question 106 appears on the next page

This is a sample form. Do not use for scanning.

Over the <u>past 12 months</u>…

105a. Each time you ate **pizza**, how much did you usually eat?

- ☐ Less than 1 slice or less than 1 mini pizza
- ☐ 1 to 3 slices or 1 mini pizza
- ☐ More than 3 slices or more than 1 mini pizza

105b. How often did you eat pizza with **pepperoni, sausage**, or **other meat**?

- ☐ Almost never or never
- ☐ About ¼ of the time
- ☐ About ½ of the time
- ☐ About ¾ of the time
- ☐ Almost always or always

106. How often did you eat **crackers**?

- ☐ NEVER (GO TO QUESTION 107)

☐ 1–6 times per year	☐ 2 times per week
☐ 7–11 times per year	☐ 3–4 times per week
☐ 1 time per month	☐ 5–6 times per week
☐ 2–3 times per month	☐ 1 time per day
☐ 1 time per week	☐ 2 or more times per day

106a. Each time you ate **crackers**, how many did you usually eat?

- ☐ Fewer than 4 crackers
- ☐ 4 to 10 crackers
- ☐ More than 10 crackers

107. How often did you eat **corn bread** or **corn muffins**?

- ☐ NEVER (GO TO QUESTION 108)

☐ 1–6 times per year	☐ 2 times per week
☐ 7–11 times per year	☐ 3–4 times per week
☐ 1 time per month	☐ 5–6 times per week
☐ 2–3 times per month	☐ 1 time per day
☐ 1 time per week	☐ 2 or more times per day

107a. Each time you ate **corn bread** or **corn muffins,** how much did you usually eat?

- ☐ Less than 1 piece or muffin
- ☐ 1 to 2 pieces or muffins
- ☐ More than 2 pieces or muffins

Question 108 appears in the next column

108. How often did you eat **biscuits**?

- ☐ NEVER (GO TO QUESTION 109)

☐ 1–6 times per year	☐ 2 times per week
☐ 7–11 times per year	☐ 3–4 times per week
☐ 1 time per month	☐ 5–6 times per week
☐ 2–3 times per month	☐ 1 time per day
☐ 1 time per week	☐ 2 or more times per day

108a. Each time you ate **biscuits**, how many did you usually eat?

- ☐ Fewer than 1 biscuit
- ☐ 1 to 2 biscuits
- ☐ More than 2 biscuits

109. How often did you eat **potato chips** (including low-fat, fat-free, or low-salt)?

- ☐ NEVER (GO TO QUESTION 110)

☐ 1–6 times per year	☐ 2 times per week
☐ 7–11 times per year	☐ 3–4 times per week
☐ 1 time per month	☐ 5–6 times per week
☐ 2–3 times per month	☐ 1 time per day
☐ 1 time per week	☐ 2 or more times per day

109a. Each time you ate **potato chips**, how much did you usually eat?

- ☐ Fewer than 10 chips or less than 1 cup
- ☐ 10 to 25 chips or 1 to 2 cups
- ☐ More than 25 chips or more than 2 cups

109b. How often were the potato chips you ate **fat-free**? *(Please do not include reduced-fat chips.)*

- ☐ Almost never or never
- ☐ About ¼ of the time
- ☐ About ½ of the time
- ☐ About ¾ of the time
- ☐ Almost always or always

110. How often did you eat **corn chips or tortilla chips** (including low-fat, fat-free, or low-salt)?

- ☐ NEVER (GO TO QUESTION 111)

☐ 1–6 times per year	☐ 2 times per week
☐ 7–11 times per year	☐ 3–4 times per week
☐ 1 time per month	☐ 5–6 times per week
☐ 2–3 times per month	☐ 1 time per day
☐ 1 time per week	☐ 2 or more times per day

Question 111 appears on the next page

This is a sample form. Do not use for scanning.

Over the past 12 months…

110a. Each time you ate **corn chips**, how much did you usually eat?

- ☐ Fewer than 10 chips or less than 1 cup
- ☐ 10 to 25 chips or 1 to 1½ cups
- ☐ More than 25 chips or more than 1½ cups

110b. How often were the corn chips or tortilla chips you ate **fat-free**? *(Please do not include reduced-fat chips.)*

- ☐ Almost never or never
- ☐ About ¼ of the time
- ☐ About ½ of the time
- ☐ About ¾ of the time
- ☐ Almost always or always

111. How often did you eat **popcorn** (including low-fat)?

- ☐ NEVER (GO TO QUESTION 112)
- ☐ 1–6 times per year
- ☐ 7–11 times per year
- ☐ 1 time per month
- ☐ 2–3 times per month
- ☐ 1 time per week
- ☐ 2 times per week
- ☐ 3–4 times per week
- ☐ 5–6 times per week
- ☐ 1 time per day
- ☐ 2 or more times per day

111a. Each time you ate **popcorn**, how much did you usually eat?

- ☐ Less than 2 cups, popped
- ☐ 2 to 5 cups, popped
- ☐ More than 5 cups, popped

112. How often did you eat **pretzels**?

- ☐ NEVER (GO TO QUESTION 113)
- ☐ 1–6 times per year
- ☐ 7–11 times per year
- ☐ 1 time per month
- ☐ 2–3 times per month
- ☐ 1 time per week
- ☐ 2 times per week
- ☐ 3–4 times per week
- ☐ 5–6 times per week
- ☐ 1 time per day
- ☐ 2 or more times per day

112a. Each time you ate **pretzels**, how many did you usually eat?

- ☐ Fewer than 5 average twists
- ☐ 5 to 20 average twists
- ☐ More than 20 average twists

Question 113 appears in the next column

113. How often did you eat **peanuts, walnuts, seeds**, or **other nuts**?

- ☐ NEVER (GO TO QUESTION 114)
- ☐ 1–6 times per year
- ☐ 7–11 times per year
- ☐ 1 time per month
- ☐ 2–3 times per month
- ☐ 1 time per week
- ☐ 2 times per week
- ☐ 3–4 times per week
- ☐ 5–6 times per week
- ☐ 1 time per day
- ☐ 2 or more times per day

113a. Each time you ate **peanuts, walnuts, seeds**, or **other nuts**, how much did you usually eat?

- ☐ Less than ¼ cup
- ☐ ¼ to ½ cup
- ☐ More than ½ cup

114. How often did you eat **energy, high-protein**, or **breakfast bars** (such as Power Bars, Balance, Clif, or others)?

- ☐ NEVER (GO TO QUESTION 115)
- ☐ 1–6 times per year
- ☐ 7–11 times per year
- ☐ 1 time per month
- ☐ 2–3 times per month
- ☐ 1 time per week
- ☐ 2 times per week
- ☐ 3–4 times per week
- ☐ 5–6 times per week
- ☐ 1 time per day
- ☐ 2 or more times per day

114a. Each time you ate **energy, high-protein**, or **breakfast bars**, how much did you usually eat?

- ☐ Less than 1 bar
- ☐ 1 bar
- ☐ More than 1 bar

115. How often did you eat **yogurt** (NOT including frozen yogurt)?

- ☐ NEVER (GO TO QUESTION 116)
- ☐ 1–6 times per year
- ☐ 7–11 times per year
- ☐ 1 time per month
- ☐ 2–3 times per month
- ☐ 1 time per week
- ☐ 2 times per week
- ☐ 3–4 times per week
- ☐ 5–6 times per week
- ☐ 1 time per day
- ☐ 2 or more times per day

115a. Each time you ate **yogurt**, how much did you usually eat?

- ☐ Less than ½ cup or less than 1 container
- ☐ ½ to 1 cup or 1 container
- ☐ More than 1 cup or more than 1 container

Question 116 appears on the next page

This is a sample form. Do not use for scanning.

Over the past 12 months…

115b. How often was the **yogurt** you ate **low-fat** or **fat-free**?

- ☐ Almost never or never
- ☐ About ¼ of the time
- ☐ About ½ of the time
- ☐ About ¾ of the time
- ☐ Almost always or always

116. How often did you eat **cottage cheese** (including low-fat)?

- ☐ NEVER (GO TO QUESTION 117)

- ☐ 1–6 times per year
- ☐ 7–11 times per year
- ☐ 1 time per month
- ☐ 2–3 times per month
- ☐ 1 time per week
- ☐ 2 times per week
- ☐ 3–4 times per week
- ☐ 5–6 times per week
- ☐ 1 time per day
- ☐ 2 or more times per day

116a. Each time you ate **cottage cheese**, how much did you usually eat?

- ☐ Less than ¼ cup
- ☐ ¼ to 1 cup
- ☐ More than 1 cup

117. How often did you eat **cheese** (including low-fat; including on cheeseburgers or in sandwiches or subs)?

- ☐ NEVER (GO TO QUESTION 118)

- ☐ 1–6 times per year
- ☐ 7–11 times per year
- ☐ 1 time per month
- ☐ 2–3 times per month
- ☐ 1 time per week
- ☐ 2 times per week
- ☐ 3–4 times per week
- ☐ 5–6 times per week
- ☐ 1 time per day
- ☐ 2 or more times per day

117a. Each time you ate **cheese**, how much did you usually eat?

- ☐ Less than ½ ounce or less than 1 slice
- ☐ ½ to 1½ ounces or 1 slice
- ☐ More than 1½ ounces or more than 1 slice

117b. How often was the cheese you ate **low-fat or fat-free**?

- ☐ Almost never or never
- ☐ About ¼ of the time
- ☐ About ½ of the time
- ☐ About ¾ of the time
- ☐ Almost always or always

Question 118 appears in the next column

118. How often did you eat **frozen yogurt**, **sorbet**, or **ices** (including low-fat or fat-free)?

- ☐ NEVER (GO TO QUESTION 119)

- ☐ 1–6 times per year
- ☐ 7–11 times per year
- ☐ 1 time per month
- ☐ 2–3 times per month
- ☐ 1 time per week
- ☐ 2 times per week
- ☐ 3–4 times per week
- ☐ 5–6 times per week
- ☐ 1 time per day
- ☐ 2 or more times per day

118a. Each time you ate **frozen yogurt**, **sorbet**, or **ices**, how much did you usually eat?

- ☐ Less than ½ cup or less than 1 scoop
- ☐ ½ to 1 cup or 1 to 2 scoops
- ☐ More than 1 cup or more than 2 scoops

119. How often did you eat **ice cream**, **ice cream bars**, or **sherbet** (including low-fat or fat-free)?

- ☐ NEVER (GO TO QUESTION 120)

- ☐ 1–6 times per year
- ☐ 7–11 times per year
- ☐ 1 time per month
- ☐ 2–3 times per month
- ☐ 1 time per week
- ☐ 2 times per week
- ☐ 3–4 times per week
- ☐ 5–6 times per week
- ☐ 1 time per day
- ☐ 2 or more times per day

119a. Each time you ate **ice cream**, **ice cream bars**, or **sherbet**, how much did you usually eat?

- ☐ Less than ½ cup or less than 1 scoop
- ☐ ½ to 1½ cups or 1 to 2 scoops
- ☐ More than 1½ cups or more than 2 scoops

119b. How often was the ice cream you ate **light**, **low-fat**, or **fat-free ice cream** or **sherbet**?

- ☐ Almost never or never
- ☐ About ¼ of the time
- ☐ About ½ of the time
- ☐ About ¾ of the time
- ☐ Almost always or always

120. How often did you eat **cake** (including low-fat or fat-free)?

- ☐ NEVER (GO TO QUESTION 121)

- ☐ 1–6 times per year
- ☐ 7–11 times per year
- ☐ 1 time per month
- ☐ 2–3 times per month
- ☐ 1 time per week
- ☐ 2 times per week
- ☐ 3–4 times per week
- ☐ 5–6 times per week
- ☐ 1 time per day
- ☐ 2 or more times per day

Question 121 appears on the next page

This is a sample form. Do not use for scanning.

Over the past 12 months…

120a. Each time you ate **cake**, how much did you usually eat?

- ☐ Less than 1 medium piece
- ☐ 1 medium piece
- ☐ More than 1 medium piece

121. How often did you eat **cookies** or **brownies** (including low-fat or fat-free)?

- ☐ NEVER (GO TO QUESTION 122)
- ☐ 1–6 times per year
- ☐ 7–11 times per year
- ☐ 1 time per month
- ☐ 2–3 times per month
- ☐ 1 time per week
- ☐ 2 times per week
- ☐ 3–4 times per week
- ☐ 5–6 times per week
- ☐ 1 time per day
- ☐ 2 or more times per day

121a. Each time you ate **cookies** or **brownies**, how much did you usually eat?

- ☐ Less than 2 cookies or 1 small brownie
- ☐ 2 to 4 cookies or 1 medium brownie
- ☐ More than 4 cookies or 1 large brownie

122. How often did you eat **doughnuts**, **sweet rolls**, **Danish**, or **pop-tarts**?

- ☐ NEVER (GO TO QUESTION 123)
- ☐ 1–6 times per year
- ☐ 7–11 times per year
- ☐ 1 time per month
- ☐ 2–3 times per month
- ☐ 1 time per week
- ☐ 2 times per week
- ☐ 3–4 times per week
- ☐ 5–6 times per week
- ☐ 1 time per day
- ☐ 2 or more times per day

122a. Each time you ate **doughnuts**, **sweet rolls**, **Danish**, or **pop-tarts**, how much did you usually eat?

- ☐ Less than 1 piece
- ☐ 1 to 2 pieces
- ☐ More than 2 pieces

123. How often did you eat **sweet muffins** or **dessert breads** (including low-fat or fat-free)?

- ☐ NEVER (GO TO QUESTION 124)
- ☐ 1–6 times per year
- ☐ 7–11 times per year
- ☐ 1 time per month
- ☐ 2–3 times per month
- ☐ 1 time per week
- ☐ 2 times per week
- ☐ 3–4 times per week
- ☐ 5–6 times per week
- ☐ 1 time per day
- ☐ 2 or more times per day

Question 124 appears in the next column

123a. Each time you ate **sweet muffins or dessert breads**, how much did you usually eat?

- ☐ Less than 1 medium piece
- ☐ 1 medium piece
- ☐ More than 1 medium piece

124. How often did you eat **fruit crisp**, **cobbler**, or **strudel**?

- ☐ NEVER (GO TO QUESTION 125)
- ☐ 1–6 times per year
- ☐ 7–11 times per year
- ☐ 1 time per month
- ☐ 2–3 times per month
- ☐ 1 time per week
- ☐ 2 times per week
- ☐ 3–4 times per week
- ☐ 5–6 times per week
- ☐ 1 time per day
- ☐ 2 or more times per day

124a. Each time you ate **fruit crisp**, **cobbler**, or **strudel**, how much did you usually eat?

- ☐ Less than ½ cup
- ☐ ½ to 1 cup
- ☐ More than 1 cup

125. How often did you eat **pie**?

- ☐ NEVER (GO TO QUESTION 126)
- ☐ 1–6 times per year
- ☐ 7–11 times per year
- ☐ 1 time per month
- ☐ 2–3 times per month
- ☐ 1 time per week
- ☐ 2 times per week
- ☐ 3–4 times per week
- ☐ 5–6 times per week
- ☐ 1 time per day
- ☐ 2 or more times per day

125a. Each time you ate **pie**, how much did you usually eat?

- ☐ Less than ⅛ of a pie
- ☐ About ⅛ of a pie
- ☐ More than ⅛ of a pie

The next four questions ask about the kinds of pie you ate. Please read all four questions before answering.

125b. How often were the pies you ate **fruit pie** (such as apple, blueberry, others)?

- ☐ Almost never or never
- ☐ About ¼ of the time
- ☐ About ½ of the time
- ☐ About ¾ of the time
- ☐ Almost always or always

Question 126 appears on the next page

This is a sample form. Do not use for scanning.

Over the past 12 months…

125c. How often were the pies you ate **cream, pudding, custard,** or **meringue pie**?

- ☐ Almost never or never
- ☐ About ¼ of the time
- ☐ About ½ of the time
- ☐ About ¾ of the time
- ☐ Almost always or always

125d. How often were the pies you ate **pumpkin** or **sweet potato pie**?

- ☐ Almost never or never
- ☐ About ¼ of the time
- ☐ About ½ of the time
- ☐ About ¾ of the time
- ☐ Almost always or always

125e. How often were the pies you ate **pecan pie**?

- ☐ Almost never or never
- ☐ About ¼ of the time
- ☐ About ½ of the time
- ☐ About ¾ of the time
- ☐ Almost always or always

126. How often did you eat **chocolate candy**?

☐ NEVER (GO TO QUESTION 127)

☐ 1–6 times per year	☐ 2 times per week
☐ 7–11 times per year	☐ 3–4 times per week
☐ 1 time per month	☐ 5–6 times per week
☐ 2–3 times per month	☐ 1 time per day
☐ 1 time per week	☐ 2 or more times per day

126a. Each time you ate **chocolate candy**, how much did you usually eat?

- ☐ Less than 1 average bar or less than 1 ounce
- ☐ 1 average bar or 1 to 2 ounces
- ☐ More than 1 average bar or more than 2 ounces

127. How often did you eat **other candy**?

☐ NEVER (GO TO QUESTION 128)

☐ 1–6 times per year	☐ 2 times per week
☐ 7–11 times per year	☐ 3–4 times per week
☐ 1 time per month	☐ 5–6 times per week
☐ 2–3 times per month	☐ 1 time per day
☐ 1 time per week	☐ 2 or more times per day

127a. Each time you ate **other candy**, how much did you usually eat?

- ☐ Fewer than 2 pieces
- ☐ 2 to 9 pieces
- ☐ More than 9 pieces

Question 128 appears in the next column

128. How often did you eat **eggs, egg whites,** or **egg substitutes** (NOT counting eggs in baked goods and desserts)? *(Please include eggs in salads, quiche, and soufflés.)*

☐ NEVER (GO TO QUESTION 129)

☐ 1–6 times per year	☐ 2 times per week
☐ 7–11 times per year	☐ 3–4 times per week
☐ 1 time per month	☐ 5–6 times per week
☐ 2–3 times per month	☐ 1 time per day
☐ 1 time per week	☐ 2 or more times per day

128a. Each time you ate **eggs**, how many did you usually eat?

- ☐ 1 egg
- ☐ 2 eggs
- ☐ 3 or more eggs

128b. How often were the eggs you ate **egg substitutes** or **egg whites only**?

- ☐ Almost never or never
- ☐ About ¼ of the time
- ☐ About ½ of the time
- ☐ About ¾ of the time
- ☐ Almost always or always

128c. How often were the eggs you ate **regular whole eggs**?

- ☐ Almost never or never
- ☐ About ¼ of the time
- ☐ About ½ of the time
- ☐ About ¾ of the time
- ☐ Almost always or always

128d. How often were the eggs you ate **cooked in oil, butter,** or **margarine**?

- ☐ Almost never or never
- ☐ About ¼ of the time
- ☐ About ½ of the time
- ☐ About ¾ of the time
- ☐ Almost always or always

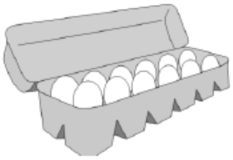

Question 129 appears on the next page

This is a sample form. Do not use for scanning.

Over the past 12 months...

128e. How often were the eggs you ate part of **egg salad**?

- ☐ Almost never or never
- ☐ About ¼ of the time
- ☐ About ½ of the time
- ☐ About ¾ of the time
- ☐ Almost always or always

129. How many cups of **coffee**, caffeinated or decaffeinated, did you drink (including coffee drinks such as Latte, Mocha, Frappuccino, etc.)?

- ☐ NONE (GO TO QUESTION 130)
- ☐ Less than 1 cup per month
- ☐ 1–3 cups per month
- ☐ 1 cup per week
- ☐ 2–4 cups per week
- ☐ 5–6 cups per week
- ☐ 1 cup per day
- ☐ 2–3 cups per day
- ☐ 4–5 cups per day
- ☐ 6 or more cups per day

129a. How often was the coffee you drank **decaffeinated**?

- ☐ Almost never or never
- ☐ About ¼ of the time
- ☐ About ½ of the time
- ☐ About ¾ of the time
- ☐ Almost always or always

130. How many glasses, cans, or bottles of **COLD** or **ICED tea**, caffeinated or decaffeinated, did you drink?

- ☐ NONE (GO TO QUESTION 131)
- ☐ Less than 1 glass, can or bottle per month
- ☐ 1–3 glasses, cans or bottles per month
- ☐ 1 glass, can or bottle per week
- ☐ 2–4 glasses, cans or bottles per week
- ☐ 5–6 glasses, cans or bottles per week
- ☐ 1 glass, can or bottle per day
- ☐ 2–3 glasses, cans or bottles per day
- ☐ 4–5 glasses, cans or bottles per day
- ☐ 6 or more glasses, cans or bottles per day

130a. How often was the cold or iced tea you drank **decaffeinated** or **herbal**?

- ☐ Almost never or never
- ☐ About ¼ of the time
- ☐ About ½ of the time
- ☐ About ¾ of the time
- ☐ Almost always or always

Question 131 appears in the next column

130b. How often was the cold or iced tea you drank **presweetened with either sugar or artificial sweeteners** (such as Splenda, Equal, Sweet'N Low or others)?

- ☐ Almost never or never (GO TO QUESTION 131)
- ☐ About ¼ of the time
- ☐ About ½ of the time
- ☐ About ¾ of the time
- ☐ Almost always or always

130c. What kind of **sweetener** was added to your presweetened cold or iced tea most of the time?

- ☐ Sugar or honey
- ☐ Artificial sweeteners (such as Splenda, Equal, Sweet'N Low or others)

131. How many cups of **HOT tea**, caffeinated or decaffeinated, did you drink?

- ☐ NONE (GO TO QUESTION 132)
- ☐ Less than 1 cup per month
- ☐ 1–3 cups per month
- ☐ 1 cup per week
- ☐ 2–4 cups per week
- ☐ 5–6 cups per week
- ☐ 1 cup per day
- ☐ 2–3 cups per day
- ☐ 4–5 cups per day
- ☐ 6 or more cups per day

131a. How often was the hot tea you drank **decaffeinated** or **herbal**?

- ☐ Almost never or never
- ☐ About ¼ of the time
- ☐ About ½ of the time
- ☐ About ¾ of the time
- ☐ Almost always or always

132. Over the past 12 months, did you add **sugar, honey or other sweeteners** to your tea or coffee (hot or iced)?

- ☐ NO (GO TO QUESTION 133)
- ☐ YES

132a. How often did you add **sugar** or **honey** to your coffee or tea (hot or iced)?

- ☐ Almost never or never (GO TO QUESTION 132c)
- ☐ About ¼ of the time
- ☐ About ½ of the time
- ☐ About ¾ of the time
- ☐ Almost always or always

Question 132c appears on the next page
Question 133 appears on the next page

This is a sample form. Do not use for scanning.

Over the past 12 months…

132b. Each time **sugar** or **honey** was added to your coffee or tea, how much was usually added?

- ☐ Less than 1 teaspoon
- ☐ 1 to 3 teaspoons
- ☐ More than 3 teaspoons

132c. How often did you add **artificial sweetener** (such as Splenda, Equal, Sweet'N Low or others) to your coffee or tea?

- ☐ Almost never or never (GO TO QUESTION 133)
- ☐ About ¼ of the time
- ☐ About ½ of the time
- ☐ About ¾ of the time
- ☐ Almost always or always

132d. What kind of **artificial sweetener** did you usually use?

- ☐ Equal or aspartame
- ☐ Sweet'N Low or saccharin
- ☐ Splenda or sucralose
- ☐ Herbal extracts or other kind

132e. Each time **artificial sweetener** was added to your coffee or tea, how much was usually added?

- ☐ Less than 1 packet or less than 1 teaspoon
- ☐ 1 packet or 1 teaspoon
- ☐ More than 1 packet or more than 1 teaspoon

133. Over the past 12 months, did you add **whiteners** (such as cream, milk, or non-dairy creamer) to your tea or coffee?

- ☐ NO (GO TO QUESTION 134)
- ☐ YES

133a. How often was **non-dairy creamer** added to your coffee or tea?

- ☐ Almost never or never (GO TO QUESTION 133d)
- ☐ About ¼ of the time
- ☐ About ½ of the time
- ☐ About ¾ of the time
- ☐ Almost always or always

Question 133d appears in the next column

Question 134 appears on the next page

133b. Each time **non-dairy creamer** was added to your coffee or tea, how much was usually used?

- ☐ Less than 1 teaspoon
- ☐ 1 to 3 teaspoons
- ☐ More than 3 teaspoons

133c. What kind of **non-dairy creamer** did you usually use?

- ☐ Regular powdered
- ☐ Low-fat or fat-free powdered
- ☐ Regular liquid
- ☐ Low-fat or fat-free liquid

133d. How often was **cream** or **half and half** added to your coffee or tea?

- ☐ Almost never or never (GO TO QUESTION 133f)
- ☐ About ¼ of the time
- ☐ About ½ of the time
- ☐ About ¾ of the time
- ☐ Almost always or always

133e. Each time **cream** or **half and half** was added to your coffee or tea, how much was usually added?

- ☐ Less than 1 tablespoon
- ☐ 1 to 2 tablespoons
- ☐ More than 2 tablespoons

133f. How often was **milk** added to your coffee or tea?

- ☐ Almost never or never (GO TO QUESTION 134)
- ☐ About ¼ of the time
- ☐ About ½ of the time
- ☐ About ¾ of the time
- ☐ Almost always or always

133g. Each time **milk** was added to your coffee or tea, how much was usually added?

- ☐ Less than 1 tablespoon
- ☐ 1 to 3 tablespoons
- ☐ More than 3 tablespoons

133h. What kind of **milk** was usually added to your coffee or tea?

- ☐ Whole milk
- ☐ 2% milk
- ☐ 1% milk
- ☐ Skim, nonfat, or ½% milk
- ☐ Evaporated or condensed (canned) milk
- ☐ Soy milk
- ☐ Rice milk
- ☐ Other

Question 134 appears on the next page

This is a sample form. Do not use for scanning.

Over the past 12 months...

134. How often was **sugar** or **honey** added to foods you ate? *(Please do not include sugar in coffee, tea, other beverages, or baked goods.)*

- ☐ NEVER (GO TO INTRODUCTION TO QUESTION 135)
- ☐ 1–6 times per year
- ☐ 7–11 times per year
- ☐ 1 time per month
- ☐ 2–3 times per month
- ☐ 1 time per week
- ☐ 2 times per week
- ☐ 3–4 times per week
- ☐ 5–6 times per week
- ☐ 1 time per day
- ☐ 2 or more times per day

134a. Each time **sugar** or **honey** was added to foods you ate, how much was usually added?

- ☐ Less than 1 teaspoon
- ☐ 1 to 3 teaspoons
- ☐ More than 3 teaspoons

The following questions are about the kinds of margarine, mayonnaise, sour cream, cream cheese, and salad dressing that you ate. If possible, please check the labels of these foods to help you answer.

135. Over the past 12 months, did you eat **margarine**?

- ☐ NO (GO TO QUESTION 136)
- ☐ YES

135a. How often was the margarine you ate **light**, **low-fat**, or **fat-free** (stick or tub)?

- ☐ Almost never or never
- ☐ About ¼ of the time
- ☐ About ½ of the time
- ☐ About ¾ of the time
- ☐ Almost always or always

136. Over the past 12 months, did you eat **butter**?

- ☐ NO (GO TO QUESTION 137)
- ☐ YES

136a. How often was the butter you ate **light** or **low-fat**?

- ☐ Almost never or never
- ☐ About ¼ of the time
- ☐ About ½ of the time
- ☐ About ¾ of the time
- ☐ Almost always or always

Question 137 appears in the next column

137. Over the past 12 months, did you eat **mayonnaise** or **mayonnaise-type dressing**?

- ☐ NO (GO TO QUESTION 138)
- ☐ YES

137a. How often was the mayonnaise you ate **light, low-fat or fat-free**?

- ☐ Almost never or never
- ☐ About ¼ of the time
- ☐ About ½ of the time
- ☐ About ¾ of the time
- ☐ Almost always or always

138. Over the past 12 months, did you eat **sour cream**?

- ☐ NO (GO TO QUESTION 139)
- ☐ YES

138a. How often was the sour cream you ate **light, low-fat**, or **fat-free**?

- ☐ Almost never or never
- ☐ About ¼ of the time
- ☐ About ½ of the time
- ☐ About ¾ of the time
- ☐ Almost always or always

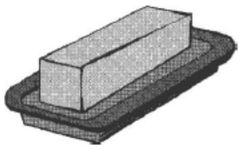

139. Over the past 12 months, did you eat **cream cheese**?

- ☐ NO (GO TO QUESTION 140)
- ☐ YES

139a. How often was the cream cheese you ate **light, low-fat**, or **fat-free**?

- ☐ Almost never or never
- ☐ About ¼ of the time
- ☐ About ½ of the time
- ☐ About ¾ of the time
- ☐ Almost always or always

Question 140 appears on the next page

This is a sample form. Do not use for scanning.

Over the past 12 months…

140. Over the past 12 months, did you eat **salad dressing**?

- ☐ NO (GO TO INTRODUCTION TO QUESTION 141)
- ☐ YES

140a. How often was the salad dressing you ate **light**, low-**fat** or **fat-free**?

- ☐ Almost never or never
- ☐ About ¼ of the time
- ☐ About ½ of the time
- ☐ About ¾ of the time
- ☐ Almost always or always

The following two questions ask you to summarize your usual intake of vegetables and fruits. Please do not include salads, potatoes, or juices.

141. Over the past 12 months, how many servings of **vegetables** (not including salad or potatoes) did you eat per week or per day?

- ☐ Less than 1 per week
- ☐ 1–2 per week
- ☐ 3–4 per week
- ☐ 5–6 per week
- ☐ 1 per day
- ☐ 2 per day
- ☐ 3 per day
- ☐ 4 per day
- ☐ 5 or more per day

142. Over the past 12 months, how many servings of **fruit** (not including juices) did you eat per week or per day?

- ☐ Less than 1 per week
- ☐ 1–2 per week
- ☐ 3–4 per week
- ☐ 5–6 per week
- ☐ 1 per day
- ☐ 2 per day
- ☐ 3 per day
- ☐ 4 per day
- ☐ 5 or more per day

143. Over the past month, which of the following foods did you eat **AT LEAST THREE TIMES?** ***(Mark all that apply.)***

- ☐ Avocado, guacamole
- ☐ Cheesecake
- ☐ Chocolate, fudge, or butterscotch toppings or syrups
- ☐ Chow mein noodles
- ☐ Croissants
- ☐ Dried apricots
- ☐ Egg rolls
- ☐ Granola bars
- ☐ Hot peppers
- ☐ Jell-O, gelatin
- ☐ Mangoes
- ☐ Milkshakes or ice-cream sodas
- ☐ Olives
- ☐ Oysters
- ☐ Pickles or pickled vegetables or fruit
- ☐ Plantains
- ☐ Pork neck bones, hock, head, feet
- ☐ Pudding or custard
- ☐ Veal, venison, lamb
- ☐ Whipped cream, regular
- ☐ Whipped cream, substitute
- ☐ NONE

144. For **ALL** of the past 12 months, have you followed any type of **vegetarian diet**?

- ☐ NO (GO TO INTRODUCTION TO QUESTION 145)
- ☐ YES

144a. Which of the following foods did you **TOTALLY EXCLUDE** from your diet? ***(Mark all that apply.)***

- ☐ Meat (beef, pork, lamb, etc.)
- ☐ Poultry (chicken, turkey, duck)
- ☐ Fish and seafood
- ☐ Eggs
- ☐ Dairy products (milk, cheese, etc.)

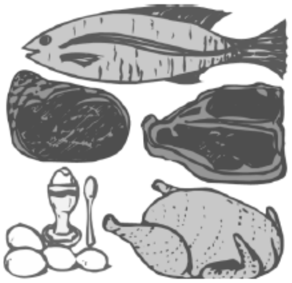

Introduction to Question 145 appears on the next page

This is a sample form. Do not use for scanning.

The next questions are about your use of vitamin pills or other supplements.

145. Over the past 12 months, did you take any **multivitamins**, such as One-a-Day-, Theragran-, Centrum-, or Prenatal-type multivitamins (as pills, liquids, or packets)?

☐ NO (GO TO INTRODUCTION TO QUESTION 147)
☐ YES

146. How often did you take **One-a-day-, Theragran-, Centrum- or Prenatal-type** multivitamins?

☐ Less than 1 day per month
☐ 1–3 days per month
☐ 1–3 days per week
☐ 4–6 days per week
☐ Every day

146a. Did your **multivitamin** usually contain **minerals** (such as iron, zinc, etc.)?

☐ NO
☐ YES
☐ Don't know

146b. For how many years have you taken **multivitamins**?

☐ Less than 1 year
☐ 1–4 years
☐ 5–9 years
☐ 10 or more years

146c. Over the past 12 months, did you take any **vitamins**, **minerals**, or **other herbal supplements** other than your multivitamin?

☐ NO

Thank you *very much* for completing this questionnaire! Because we want to be able to use all the information you have provided, we would greatly appreciate it if you would please take a moment to review each page making sure that you:

- **Did not skip any pages and**
- **Crossed out the incorrect answer and circled the correct answer if you made any changes.**

☐ YES (GO TO INTRODUCTION TO QUESTION 147

Introduction to Question 147 appears in the next column

These last questions are about the vitamins, minerals, or herbal supplements you took that are NOT part of a One-a-day-, Theragran-, or Centrum-type of multivitamin.

Over the past 12 months...

147. How often did you take **Antacids such as Tums or Rolaids**?

☐ NEVER (GO TO QUESTION 148)

☐ Less than 1 day per month
☐ 1–3 days per month
☐ 1–3 days per week
☐ 4–6 days per week
☐ Every day

147a. When you took **Antacids such as Tums or Rolaids**, about how many tablets or lozenges did you take in one day?

☐ Less than 1
☐ 1
☐ 2
☐ 3
☐ 4 or more
☐ Don't know

147b. Was your antacid usually "extra strength"?

☐ NO
☐ YES
☐ Don't know

147c. For how many years have you taken **Antacids such as Tums or Rolaids**?

☐ Less than 1 year
☐ 1–4 years
☐ 5–9 years
☐ 10 or more years

148. How often did you take **Calcium** (with or without Vitamin D) (**NOT** as part of a multivitamin in Question 146 or antacid in Question 147)?

☐ NEVER (GO TO QUESTION 149)

☐ Less than 1 day per month
☐ 1–3 days per month
☐ 1–3 days per week
☐ 4–6 days per week
☐ Every day

Question 149 appears on the next page

This is a sample form. Do not use for scanning.

Over the past 12 months...

148a. When you took **Calcium**, about how much elemental calcium did you take in one day? *(If possible, please check the label for elemental calcium.)*

- ☐ Less than 500 mg
- ☐ 500–599 mg
- ☐ 600–999 mg
- ☐ 1,000 mg or more
- ☐ Don't know

148b. Did your **Calcium** usually contain **Vitamin D**?

- ☐ NO
- ☐ YES
- ☐ Don't know

148c. Did your **Calcium** usually contain **Magnesium**?

- ☐ NO
- ☐ YES
- ☐ Don't know

148d. Did your **Calcium** usually contain **Zinc**?

- ☐ NO
- ☐ YES
- ☐ Don't know

148e. For how many years have you taken **Calcium**?

- ☐ Less than 1 year
- ☐ 1–4 years
- ☐ 5–9 years
- ☐ 10 or more years

149. How often did you take **Iron** (**NOT** as part of a multivitamin in Question 146)?

- ☐ NEVER (GO TO QUESTION 150)
- ☐ Less than 1 day per month
- ☐ 1–3 days per month
- ☐ 1–3 days per week
- ☐ 4–6 days per week
- ☐ Every day

149a. For how many years have you taken **Iron**?

- ☐ Less than 1 year
- ☐ 1–4 years
- ☐ 5–9 years
- ☐ 10 or more years

Question 150 appears in the next column

150. How often did you take **Vitamin C** (**NOT** as part of a multivitamin in Question 146)?

- ☐ NEVER (GO TO QUESTION 151)
- ☐ Less than 1 day per month
- ☐ 1–3 days per month
- ☐ 1–3 days per week
- ☐ 4–6 days per week
- ☐ Every day

150a. When you took **Vitamin C**, about how much did you take in one day?

- ☐ Less than 500 mg
- ☐ 500–999 mg
- ☐ 1,000–1,499 mg
- ☐ 1,500–1,999 mg
- ☐ 2,000 mg or more
- ☐ Don't know

150b. For how many years have you taken **Vitamin C**?

- ☐ Less than 1 year
- ☐ 1–4 years
- ☐ 5–9 years
- ☐ 10 or more years

151. How often did you take **Vitamin E** (**NOT** as part of a multivitamin in Question 146)?

- ☐ NEVER (GO TO INTRODUCTION TO QUESTION 152)
- ☐ Less than 1 day per month
- ☐ 1–3 days per month
- ☐ 1–3 days per week
- ☐ 4–6 days per week
- ☐ Every day

151a. When you took **Vitamin E**, about how much did you take in one day?

- ☐ Less than 400 IU
- ☐ 400–799 IU
- ☐ 800–999 IU
- ☐ 1,000 IU or more
- ☐ Don't know

151b. For how many years have you taken **Vitamin E**?

- ☐ Less than 1 year
- ☐ 1–4 years
- ☐ 5–9 years
- ☐ 10 or more years

Introduction to Question 152 appears on the next page

This is a sample form. Do not use for scanning.

Over the past 12 months…

The last two questions ask you about other supplements you took more than once per week.

152. Please mark any of the following **single supplements** you took more than once per week (**NOT** as part of a multivitamin in Question 147):

- ☐ B-6
- ☐ B-complex
- ☐ B-12
- ☐ Beta-carotene
- ☐ Folic acid/folate
- ☐ Magnesium
- ☐ Occu-vite/Eye health
- ☐ Potassium
- ☐ Selenium
- ☐ Vitamin A
- ☐ Vitamin D
- ☐ Zinc

153. Please mark any of the following **herbal, botanical, or other supplements** you took more than once per week.

- ☐ Chondroitin
- ☐ Coenzyme Q-10
- ☐ Echinacea
- ☐ Energy supplements
- ☐ Fish oil/omega 3's
- ☐ Flaxseed/oil
- ☐ Garlic
- ☐ Ginger
- ☐ Ginkgo biloba
- ☐ Ginseng
- ☐ Glucosamine/ chondroitin
- ☐ Peppermint
- ☐ Probiotics
- ☐ Saw palmetto
- ☐ Soy supplement
- ☐ Sports supplements
- ☐ St. John's wort
- ☐ Other

Thank you *very much* for completing this questionnaire! Because we want to be able to use all the information you have provided, we would greatly appreciate it if you would please take a moment to review each page making sure that you:

- **Did not skip any pages and**
- **Crossed out the incorrect answer and circled the correct answer if you made any changes.**

27 Appendix L: Automated Self-Administered 24-hour Recall (ASA24)

Overview of the ASA24 Respondent Web sites and Considerations Related to Data Security and Participant Confidentiality

Extensive evidence has demonstrated that 24-hour dieary recalls provide the highest quality, least biased dietary data. Traditional 24-hour recalls, however, are expensive and impractical for large-scale research because they rely on trained interviewers and multiple administrations to estimate usual intakes. As a result, researchers often make use of food frequency questionnaires, which are less expensive but contain substantial error.

To address this challenge, investigators at NCI created the Automated Self-administered 24-hour Recall (ASA24) system, a freely-available, web-based tool that enables multiple automated self-administered 24-hour recalls. ASA24 was developed under contract with Westat, a social science research firm located in Rockville, MD, and builds on t he Food Intake Recording Software System (FIRSSt) developed by Dr. Tom Baranowski of the Baylor College of Medicine. An Extern al Working Group provided advice about the needs and interests of potential users.

ASA24 consists of a Respondent Web site used to collect recall data and a Researcher Web site used to manage study logistics and obtain data analyses. Two Respondent Web sites are currently available in English and Spanish: ASA24-2014 and ASA24-Kids-2014. This document provides an overview of the methodology and main features of the ASA24 Respondent sites, as well as information on security of the data collected and protections to the confidentiality of the participants of studies that make use of ASA24. Images of the main Respondent site screens are also included.

ASA24 Respondent Web site Methodology

Respondents are guided through the 24-hour recall inter view using a modified version of the USDA's Automated Multiple-Pass Method (AMPM). The steps in the interview process include:

1. Meal-based Quick List
2. Meal Gap Review
3. Detail Pass
4. Forgotten Foods
5. Fina l Review
6. Last Chance
7. Usual Intake Question
8. Supplement Module (if selected by the researcher)

Meal-based Quick List
During the first pass of the interview, respondents are asked to provide a list of the foods and drinks consumed at each meal occasion for one of two possible 24-hour time periods, depending on the preference of the researcher: yesterday, from midnight to midnight; or the past 24-hours based on the time of participant login. Respondents are able to browse food group categories to find their foods and drinks or search for a particular item. Foods and drinks

reported at each meal are recorded in the My Foods and Drinks panel within the instrument. This initial step is called the meal-based Quick List. In addition to selecting an eating occasion (e.g., breakfast, lunch, snack), respondents are also prompted to specify the time of the occasion before reporting the foods and drinks consumed. The researcher can opt to collect additional contextual information including where meals were eaten, television and computer use during meals, and whether the meal was eaten alone or with others.

Meal Gap Review

Once respondents finish creating their My Foods and Drinks list at the end of the Quick List step, they are asked if they consumed anything during any 3-hour gaps between eating occasions, and, for the midnight to midnight version, between midnight and the first eating occasion, and between the last eating occasion and midnight. A response of "Yes" to any gap will return the respondent to the Quick List to add the appropriate food(s) or drink(s).

Detail Pass

Respondents are asked for details about the foods and drinks they recorded during the Quick List, including form (e.g., raw), preparation methods (e.g., grilled or roasted), the amount eaten, and any additions (e.g., sugar, coffee cream, salad dressing). Researchers can also opt to ask the source (grocery store, farmer's market, vending machine, etc.) of each food reported.

Forgotten Foods

Following the Detail Pass, a pop-up window appears with questions probing about the consumption of commonly forgotten foods and drinks (e.g., snack foods, fruits, vegetables, cheese, water, coffee, tea). Respondents must check either "Yes" or "No" for each food or drink probed. For any "Yes" response, the respondent will be returned to the Quick List to add the forgotten item(s).

Final Review

Respondents are prompted to review all of the foods and drinks reported for the intake day, and make edits and add meals, foods and drinks as necessary.

Last Chance

After the Final Review, a pop-up screen appears with an option to add food or drink items if respondents have still forgotten any. Again, respondents will be brought back to the Quick List to add more items; otherwise, they will move forward to the final question in the food and drink module.

Usual Intake Question

The final question asks: Was the amount of food that you ate yesterday more than usual, usual, or less than usual? This question probes whether this was a typical day's intake.

Supplement Module (if selected by the researcher during study set-up)

Respondents are asked to provide information about the type and dose of supplements consumed by comp leting a Quick List, a Detail Pass, and a Final Review . Supplements include vitamins, minerals, and other supplements including prescription supplements . Respondents are able to browse supplement categories (e.g., Multi-Vitamin/ Mineral, Calcium products, Herbal/Botanicals) or search for a particular supplement. The supplements included are based on those reported in the National Health and Nutrition Examination Survey.

ASA24 Respondent Web Site Features

The ASA24 Respondent Web site guides the part icipant t hrou gh the completion of a 24-hour recall for the previous day, from midnight to midnight, using a dynamic user interface. The instrument:

- provides an animated guide and audio and visual cues to instruct participants and enhance use in low-literacy populations and with children (with options to turn off the guide and/or the audio);
- asks respondents to report eating occasion and time of consumption;
- includes optional modules to query where meals were eaten, whether meals were eaten alone or with others, and television and computer use during meals;
- flows as per modified USDA Automated Multiple-Pass Method (AM PM) 24-hour recall; allows respondents to report foods and drinks by browsing by category or searching from a list of food and drink terms derived from the National Health and Nutrition Examination Survey (NHANES);
- asks detailed questions about food preparation, portion size, and additions so that food codes from USDA's Food and Nutrient Database for Dietary Studies (FNDDS) can be assigned; it also provides an option to query respondents about food source;
- uses images to assist respondents in reporting portion size;
- allows the respondent to add or modify food and drink choices at multiple points during the interview;
- includes an optional module to query dietary supplement intake based on supplements reported in NHANES;
- is available in English and Spanish; and
- is compliant with Section 508 of t he 197 3 Rehabilitation Act.

The Respondent Web sites do not provide any direct feedback to respondents. Instead, researchers can obtain analysis files from the Researcher Website and contact respondents with any findings they choose to share.

Data Security and Protections to Confidentiality of Participants using ASA24

Researchers using ASA24 do not provide the National Cancer Institute, Westat (the research firm that developed and maintains ASA24), or the ASA24 system with any identifying data for participants of their studies. Rather, researchers specify an identifier for each respondent and download system-generated usernames and encrypted passwords that they provide to respondents so that they may access the application.

AS24 also does not collect any identifying data directly from respondents. However, IP address information is accessed for the purpose of routing information between the server and the respondent's computer – often the IP address is that of the user's Internet Service Provider (ISP). IP addresses are not stored or tracked by ASA24. However, logs of connections are kept in the hosting environment for audit trail purposes. This information is not mined in any way but would be available if there were legal obligations to release it.

Response data are secured at the hosting site using industry standard security controls, including firewalls and encruyption. All data entered into ASA24 at the respondent's computer is encrypted by the internet browser (e.g., Internet Explorer, Firefox) before they are transmitted to our servers using Secure Socket Layer (SSL) Technology. SSL allows for the authentication of the sending and receiving computers.

Only a particular study's investigator(s) and the ASA24 operations team can access response data. Access is gained through usernames and strong passwords.

ASA24 Respondent Web site Screen Shots

Figure 1: The participant can choose to complete ASA24 in either English or Spanish.

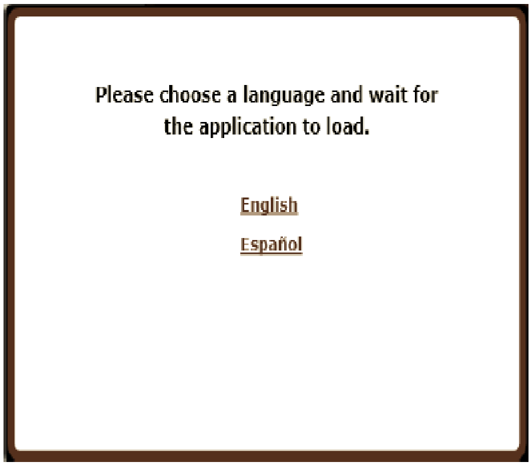

Figure 2: An introductory screen prompts the participant to report everything that she or he had to eat or drink yesterday.

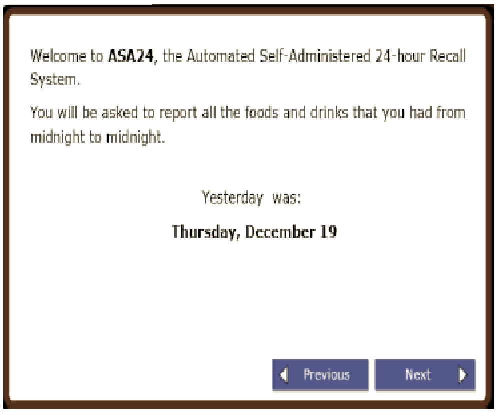

4

Figure 3: The main ASA24 screen is used to select foods and drinks to be added to My Foods and Drinks during the Quick Pass, and to add details and make changes. The animated guide provides an overview of the main ASA24 screen and gives instructions on how to report all foods, drinks, and supplements (if the optional Supplement Module is selected by the researcher) consumed either from midnight to midnight yesterday or for the past 24 hours, starting at the time of login (depending on the researcher preferences).

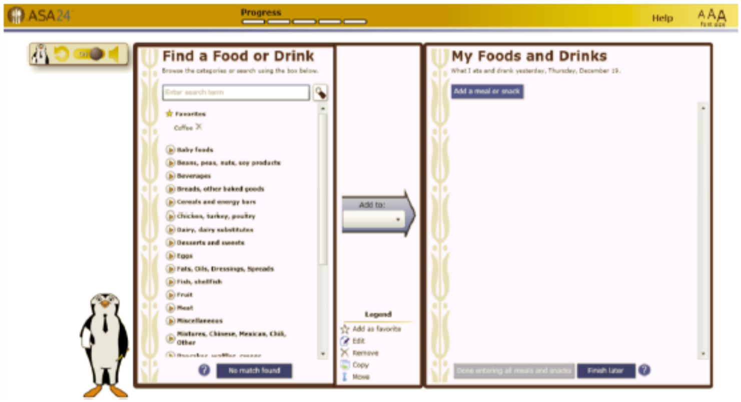

Figure 4: The meal details screen collects information on the eating occasion and time, and displays optional modules that can be selected by the researcher to query where meals were eaten, whether meals were eaten alone or with others, and television and computer use during meals. This is the first step in the meal-based Quick List.

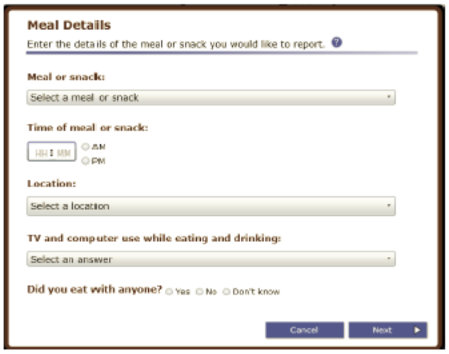

5

Figure 5: Participants complete the Quick List pass by browsing categories or searching for the foods and drinks they consumed. The food and drink terms are based on the National Health and Nutrition Examination Survey.

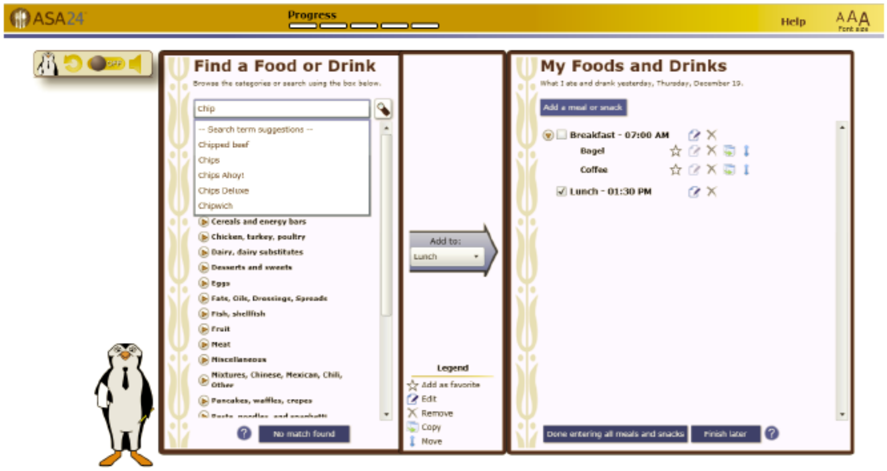

Figure 6: The foods and drinks that the participant reports during the Quick List are displayed in the My Foods and Drinks panel on the right of the main screen.

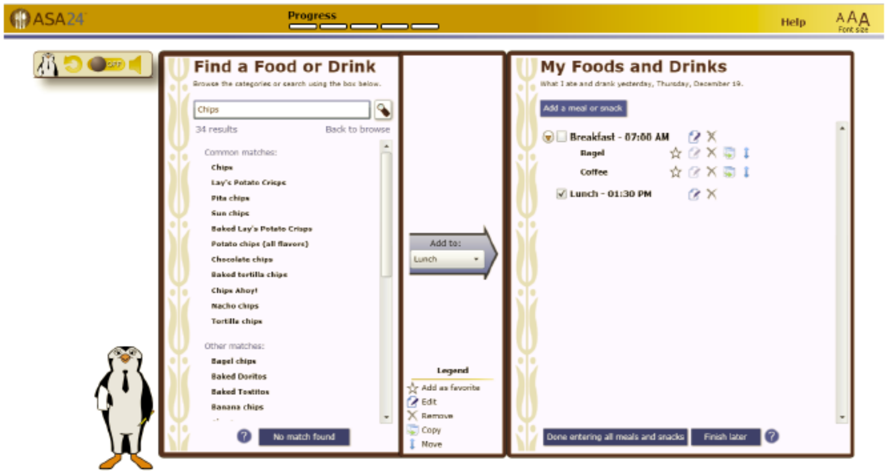

Figure 7: After the participant reports the foods and drinks consumed yesterday and selects Done entering all meals and snacks, the Meal Gap Review is displayed.

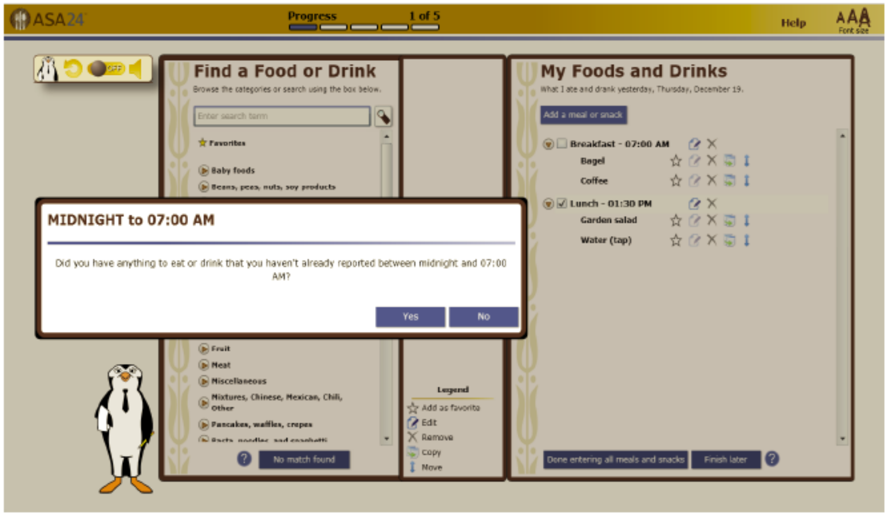

Figures 8a-c: The detail pass prompts the participant to report the details of each food and drink, including the specific type or how it was prepared, anything added to it, and the amount that was consumed.

Figure 8a.

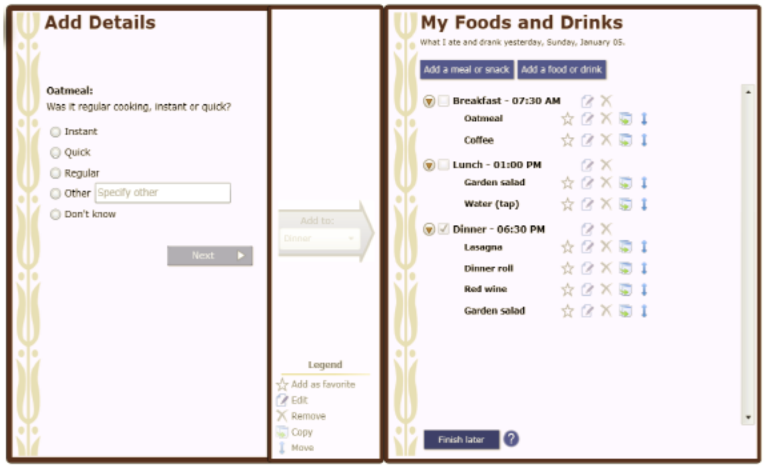

7

Figure 8b.

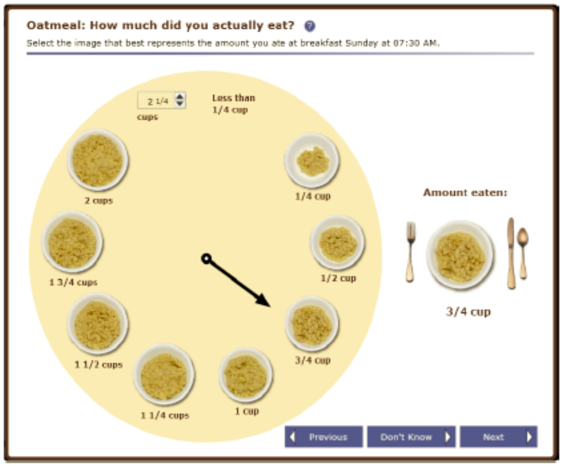

Figure 8c.

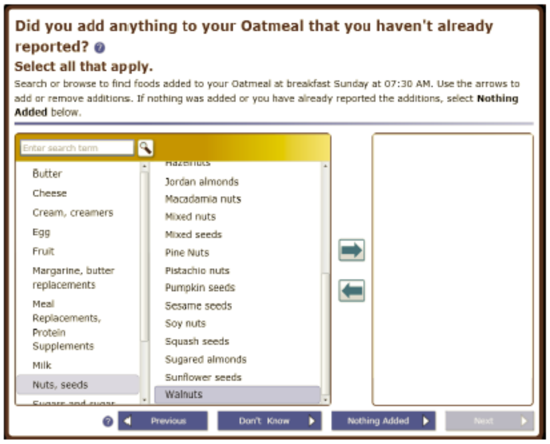

8

Figure 9: Once the Detail Pass is complete, the Final Review begins. The participant can make changes to the details for a given food or drink and add more foods and drinks if necessary.

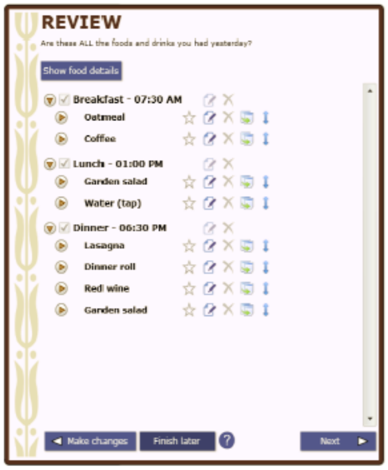

Figure 10: The forgotten foods pass queries the participant about frequently forgotten foods. Responding yes to one or more categories will result in a prompt to add the forgotten foods or drinks.

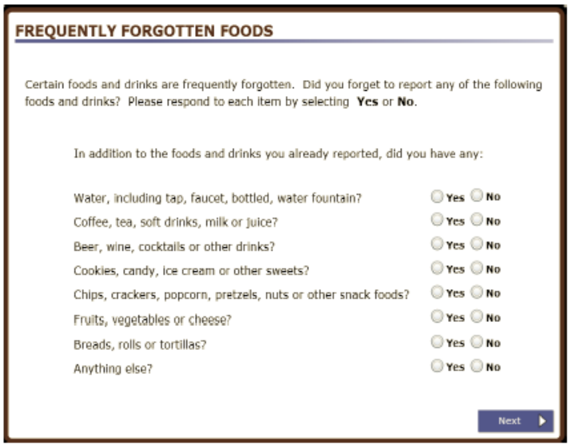

Figure 11: The final question in the food and drink module asks the participant whether the amount of foods and drinks consumed yesterday was usual.

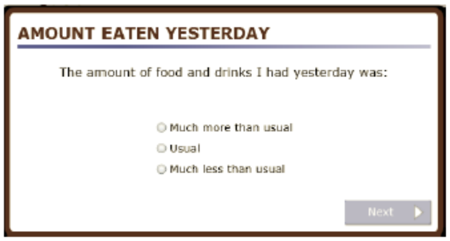

Figure 12: If the Supplement Module is selected by the researcher during study set up, the participant will also be asked about the supplements, if any, that he or she took either from midnight to midnight yesterday or for the past 24 hours, starting at the time of login (depending on researcher preferences).

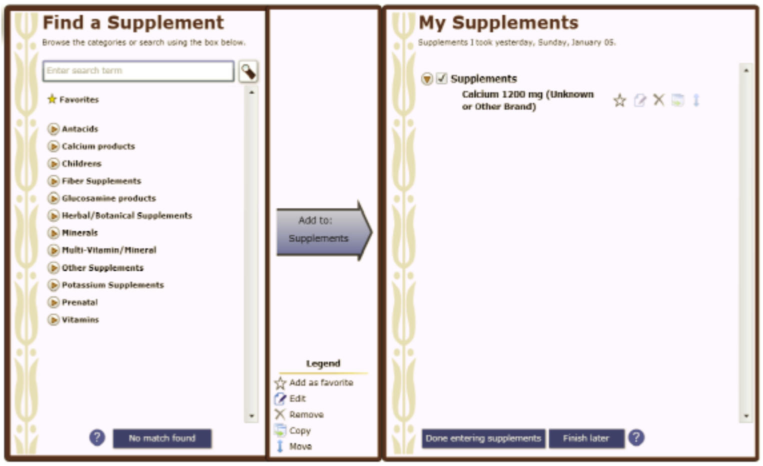

10

Figure 13: The Supplement Module collects details about each supplement reported.

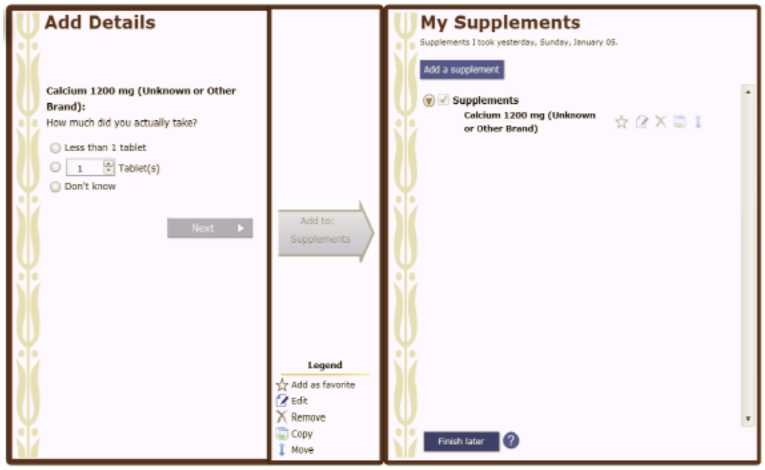

Figure 14: Once the participant completes the food and drink module and the supplement module, if selected by the researcher during study set up, his or her responses are saved in the secure database and he or she can exit ASA24. Only the researcher(s) who are registered to that study and the ASA24 operations team can access the response data.

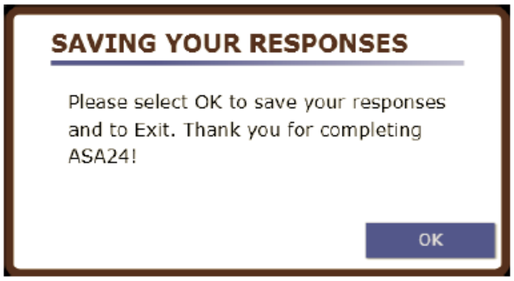

11

28 APPENDIX M: qADAM Questionnaire

Questions Used as Part of the qADAM Questionnaire

1. How would you rate your libido (sex drive)?
 1(terrible) 2(poor) 3(average) 4(good) 5(excellent)

2. How would you rate your energy level?
 1(terrible) 2(poor) 3(average) 4(good) 5(excellent)

3. How would you rate your strength/endurance?
 1(terrible) 2(poor) 3(average) 4(good) 5(excellent)

4. How would you rate your enjoyment of life?
 1(terrible) 2(poor) 3(average) 4(good) 5(excellent)

5. How would you rate your happiness level?
 1(terrible) 2(poor) 3(average) 4(good) 5(excellent)

6. How strong are your erections?
 (1= extremely weak 5= extremely strong)
 1 2 3 4 5

7. How would you rate your work performance over the past 4 weeks?
 1(terrible) 2(poor) 3(average) 4(good) 5(excellent)

8. How often do you fall asleep after dinner?
 1(never) 2(1-2/week) 3(3-4/week) 4(5-6/week) 5(every night)

9. How would you rate your sports ability over the past 4 weeks?
 1(terrible) 2(poor) 3(average) 4(good) 5(excellent)

10. How much height have you lost?
 1(2" or more) 2(1.5-1.9") 3(1-1.4") 4(0.5-0.9") 5(none-0.4")

The quantitative ADAM questionnaire. Int J Impot Res. 2010 Jan; 22(1): 20–24.

O Mohamed,[1] *R E Freundlich*,[1] *H K Dakik*,[1] *E D Grober*,[2] *B Najari*,[3] *L I Lipshultz*,[1] and *M Khera*[1,*]

Published online 2009 Aug 6. doi: *10.1038/ijir.2009.35*
PMCID: PMC2834355

29 Appendix N: Psychosexual Daily Questionnaire

1. Please rate your overall level of sexual desire today by circling the appropriate number below:

0 — 1 — 2 — 3 — 4 — 5 — 6 — 7

(0 = none; 1 = very low; 7 = very high)

2. Please rate highest level of enjoyment or pleasure of any sexual activity that you experienced today.

(a) without a partner (e.g., masturbationl sexual fantasies) and/or (b) with a partner (e.g., kissing, intercourse) by circling the appropriate number below.

(a) Without a partner — 0 — 1 — 2 — 3 — 4 — 5 — 6 — 7 (0 = none; 7 = very high enjoyment/pleasure)

(b) With a partner — 0 — 1 — 2 — 3 — 4 — 5 — 6 — 7 (0 = none; 7 = very high enjoyment/pleasure)

(c) Please indicate if partner is available ☐ yes ☐ no

3. Please rate your mood by writing the number that corresponds to the scale below. For each item 0 indicates that the descriptor is not at all true; 7 indicates that the descriptor is very true for you today.

not at all true 0 — 1 — 2 — 3 — 4 — 5 — 6 — 7 very true

(a) Angry? ___	(d) Full of pep/energetic? ___	(g) Friendly? ___
(b) Alert? ___	(e) Sad or Blue? ___	(h) Nervous? ___
(c) Irritable? ___	(f) Tired? ___	(i) Well/good? ___

4. For all of the items below check yes if you have experienced (or are experiencing) today, otherwise check no.

	Yes	No			Yes	No			Yes	No	
(a)	☐	☐	sexual daydreams	(e)	☐	☐	orgasm	(i)	☐	☐	masturbation
(b)	☐	☐	anticipation of sex	(f)	☐	☐	flirting (by others toward you)	(j)	☐	☐	night spontaneous erection
(c)	☐	☐	sexual interactions with partner	(g)	☐	☐	ejaculation	(k)	☐	☐	day spontaneous erection
(d)	☐	☐	flirting (by you)	(h)	☐	☐	intercourse	(l)	☐	☐	erection in response to sexual activity

Answer the following two questions only if you experienced an erection as shown in No. 4. j-l above.

5. If you experienced an erection today, indicate the % full erection that you experienced by circling the appropriate number below (make a reasonable estimate):

%= 0 10 20 30 40 50 60 70 80 90 100

6. If you experienced an erection today, indicate whether it was maintained for a satisfactory duration by circling the appropriate number below:

not satisfactory 0 — 1 — 2 — 3 — 4 — 5 — 6 — 7 very satisfactory

A Simple Self-Report Diary for Assessing Psychosexual Function in Hypogonadal Men
Ka Kui Lee[1,2], Nancy Berman[3], Gerianne M. Alexander[4], Laura Hull[1], Ronald S. Swerdloff[1] and Christina Wang[1,*]
Article first published online: 2 JAN 2013
DOI: 10.1002/j.1939-4640.2003.tb02728.x

30 Appendix O: DSMB Charter

Improving Reproductive Fitness through Pretreatment with Lifestyle Modification in Obese Women with Unexplained Infertility: (FIT-PLESE FEMALE)

DATA AND SAFETY MONITORING BOARD (DSMB) CHARTER

1. Purpose and Responsibilities of the DSMB

The members of the Data and Safety Monitoring Board (DSMB) identified in this Charter for the FIT-PLESE Female study are responsible for safeguarding the interests of study participants, assessing the safety and efficacy of all study procedures, and shall monitor the overall conduct of the FIT-PLESE Female trial. This Committee will serve as an independent advisory group to the Director of NICHD, and is required to provide recommendations about starting, continuing, and stopping the FIT-PLESE Female study.

This Committee is responsible for identifying mechanisms to complete various tasks that will impact the safety and efficacy of all study procedures, and overall conduct. The table below identifies the key areas where oversight is necessary and the ways in which the Committee for the FIT-PLESE Female study will complete those tasks.

Basic Responsibility of DSMB	Method DSMB for FIT-PLESE Female will use to complete task
Familiarize themselves with the study protocol	· Review study protocols and informed consent forms.
Monitor adverse events	· Adverse Event: Review quarterly progress reports prepared by the DCC on behalf of RMN.<br>· Serious Adverse Events: Review report submitted by the DCC on behalf of RMN within one week of the event if life threatening or fatal, or within two weeks otherwise. The DSMB will submit a report of their review to the NICHD Committee Coordinator within 7 business days if the SAE is life threatening or fatal, or within two weeks otherwise.
Monitor data quality	· Conduct interim evaluations of the data.
Oversee participant recruitment and enrollment	· Review interim progress reports prepared by the DCC on behalf of RMN.
Develop an understanding of the Study's risks and benefits	· Review study protocols and related literatures.<br>· Review interim reports of subject accrual and outcome measures provided by the DCC.

	· Assess the need to perform further in-depth evaluation of the benefits and risks of the study after reviewing each report.
Ensure the proper reporting occurs	· Review and approve the meeting and reporting schedule listed in Section 5 of this DSMB charter.

2. Contacts

NICHD
Louis DePaolo, PhD, Program Officer
Charisee Lamar, PhD, MPH, RRT Committee Coordinator
Esther Eisenberg, MD, MPH, Project Scientist

Data Coordination Center (DCC)
Heping Zhang, PhD, DCC Principal Investigator
Hao Huang, MD, DCC Data Manager

The Data Manager at the DCC will prepare and review the DSMB reports prior to submission to the DSMB, and will not be blind to treatment condition.

Lead Investigator
Richard S. Legro, MD

3. DSMB Members, Organizational Chart, & Communications

Members

The DSMB for the FIT-PLESE Female study is comprised of the members listed in the table below. In addition, their high level roles and responsibilities are identified in the table.

Name of Member	Role on DSMB	High Level Responsibilities
Frank Witter, MD	Chair of DSMB<br>Voting member	· Chair the DSMB discussion and prepare written recommendations to NICHD.<br>· Ensure the safety of study subjects, the integrity of the research data.<br>· Provide NICHD with advice on the ethical and safe progression of studies conducted in the RMN.<br>· Advises on research design issues, data quality and analysis, and research participant protection for each prospective and on-going study.
Rev. Phillip Cato, PhD	Voting member	· Ensure the safety of study subjects, the integrity of the research data.<br>· Provide NICHD with advice on the ethical and safe progression of studies conducted in the RMN.
PonJola Coney, MD	Voting member	
Lurdes Y.T. Inoue, PhD	Voting member	
Stacey A. Missmer, ScD	Voting member	

Robert E. Brannigan, MD	Voting member	· Advises on research design issues, data quality and analysis, and research participant protection for each prospective and on-going study.

Only Voting members for this DSMB may attend closed sessions for this Committee. In addition, only Voting members will have access to unblinded data points for this Committee.

Organizational Chart

The following diagram illustrates the relationship between the DSMB and other entities in the FIT-PLESE Female study.

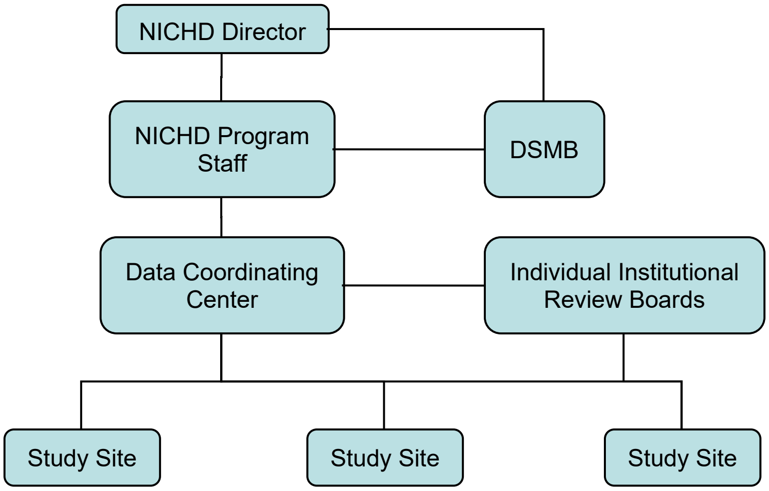

Communication

Communication for members of this DSMB will be primarily through the NICHD Program Office and, where applicable, the Data Coordination Center (DCC). Investigators from the FIT-PLESE Female study will not communicate directly with DSMB members about the study, except when making presentations or responding to questions at DSMB meetings or during scheduled conference calls.

4. Conflict of Interest and Compensation

It is extremely important that all members of the DSMB state any real or apparent conflicts of interests at the onset of the study. Members of the DSMB shall read the NICHD Clinical Research Guidance Document regarding Conflict of Interest and provide their signed summary of any COI for the study, at its onset, to the NICHD Committee Coordinator, Dr. Charisee Lamar. A table summarizing any COI within the DSMB is provided in the Appendix.

Prior to each meeting, all members of the RMN DSMB will have an opportunity to state whether they have developed any new conflicts of interest since the meeting. As a new COI is identified it must be documented in the table in the Appendix and a new signed

summary of the COI should be provided to the NICHD Committee Coordinator. The Coordinator will forward the COI documentation to the DCC for record-keeping purposes. If a new conflict is reported, the Coordinator and staff will determine if the conflict limits the ability of the DSMB member to participate in the discussion.

All DSMB members will be compensated for their role in supporting the committee. Compensation will include an honorarium for meeting attendance and any travel costs.

5. Meeting Schedule

DSMB meetings will be conducted quarterly. However, the DSMB may hold a meeting at any time in accordance with their mission. The NICHD Committee Coordinator will notify the DCC of any change in schedule.

6. Blinding

All summaries for DSMB reports will be presented in a blinded fashion, unless specified by the DSMB Chair.

7. Report Schedule and Content

The type of reports (full or brief) is indicated below, followed by a description of the contents of each type.

DSMB Report	Report Submission Date	Type of Report
1.	tbd	Brief
2.	tbd	Brief
3.	tbd	Brief
4.	tbd	**Full**
5.	tbd	Brief
6.	tbd	**Full**
7.	tbd	Brief
8.	tbd	**Full**
9.	tbd	Brief
10.	tbd	**Full**
11.	tbd	Brief
12.	tbd	**Full**
13.	tbd	Brief
14.	tbd	**Full**
15.	tbd	Brief
16.	tbd	**Full**

Brief DSMB reports will include the following summaries:

- overall actual versus projected enrollment accrual
- overall randomization update
- overall study drop-out rate
- serious adverse events
- primary outcome measures update

Full DSMB reports will include the following summaries:

- recruitment update (number screened) overall and by site
- enrollment update (enrolled defined as randomized to a treatment) overall and by site
- accrual status including actual enrollment compared to projections overall and by site
- randomization update (i.e., number assigned to each treatment arm)
- study drop-out rate for enrolled patients (number, reason, time point) overall and by site)
- pre-specified subset of baseline demographic data for enrolled patients
- safety data, adverse events, and serious adverse events
- number of case report forms expected
- number/percentage of expected case report forms received – overall and by site
- number of case report forms that are query clean
- primary outcome measures update

References

NIH Policy for Data and Safety Monitoring
http://grants.nih.gov/grants/guide/notice-files/NOT98-084.html
Guidance on Reporting Adverse Events to Institutional Review Boards for NIH-supported Multi-center Clinical Trials
http://grants.nih.gov/grants/guide/notice-files/not99-107.html.

Appendix: Summary of COI within the DSMB

DSMB Member Name	Date Submitted Signed COI	Was a COI Identified?	Will the Member Remain part of the Committee?
Rev. Phillip Cato, PhD			
PonJola Coney, MD			
Lurdes Y.T. Inoue, PhD			
Stacey A. Missmer, ScD			
Robert E. Brannigan, MD			
Frank Witter, MD			

Conflict of Interest Statement

Improving Reproductive Fitness through Pretreatment with Lifestyle Modification in Obese Women with Unexplained Infertility: (FIT-PLESE FEMALE)

I, ______________________, assuming the role of _____________________________ (insert role, for example: DSMB member)

for the ____
(insert project or study name)

agree to the following statements.

□ I agree to:

- protect the interests and safety of study participants;
- uphold the integrity of the research process including data collection and analysis to be as free from bias and preconception as I am able;
- adhere to the highest scientific and ethical standards, to comply with all relevant regulations and to eliminate or disclose, during my involvement with the proposed clinical research project, any real or apparent conflicts of interest.

In addition:

□ I declare that I, my spouse or dependent children, or organization with which I am connected, do/does not have any financial interest in the ___________________ study, where financial interested is defined by the DHHS, as anything of monetary value, including but not limited to, salary or other payments for services (for example, consulting fees or honoraria); equity interests (for example, stocks, stock options or other ownership interests); and intellectual property rights (for example, patents, copyrights and royalties from such rights).
The financial interest term does not include various items which can be found in The Federal regulation, PHS, DHHS Part 50--Policies of General Applicability; Subpart F- *Responsibility of Applicants for Promoting Objectivity in Research for Which PHS Funding Is Sought.*

For Federal employees, financial interests that are allowable and require disclosure are:

Financial Interest Disclosure: Financial interest that require disclosure are stock holdings in pharmaceutical firms, medical device manufacturers, and biotechnology companies

Allowable Financial Interests: In a company that produces a product that is being evaluated by a study, participants may hold up to $15,000 of stock: and up to an aggregate of $25,000 of the stock of that

company and its competitors who produce that (or a similar) product. As an alternative to individual stock holdings, participants may hold up to an aggregate of $50,000 in sector mutual funds-including pharmaceutical/health care sectors.

For holdings in excess of these *de minimus* levels, a conflict of interest analysis needs to be conducted by NIH regarding the holding, the company producing the product being evaluated under the study, and its competitors, and, if a conflict exists, could lead to the need to withdraw from the study.

□ I agree to not withhold any data related to the ____________________ study or to interfere with the analysis or publication of the study's results.

□ I will not engage in activities that could be viewed as real or apparent COI, including but not limited to:

> □ having a part-time, full-time, paid, or unpaid employee status of any organizations that are: (a) involved in the study under review; (b) whose products will be used or tested in the study under review, or whose products or services would be directly and predictably affected in a major way by the outcome of the study;
>
> □ being an officer, member, owner, trustee, director, expert advisor, or consultant of such organizations;
>
> □ being a current collaborator or associate of the principal investigator (applicable to potential members of data safety and monitoring boards);
>
> □ having a scientific interest beyond that required for my role, where scientific interest is defined as having influence over the protocol, the study design, conducting the study analysis or any reporting related to the investigation (applicable to potential members of data safety and monitoring boards).

Reproductive Medicine Network (RMN)
Data and Safety Monitoring Board (DSMB)
Confidentiality Agreement

As a DSMB member I understand that I will be provided with and have access to documents submitted by the NICHD or the Data Coordination Center as they relate to RMN protocols, Registries or other Network-related materials, including proprietary and confidential information.

I shall not disclose any confidential RMN information (oral or written) unless required to do so by law. Confidential documents may be distributed to an administrative assistant, who is not permitted to share Network materials with anyone other than me.

I agree that I will not distribute or publish RMN records. I further agree that I shall not make use of Network materials except for the express purpose of advising the RMN Steering Committee and the NICHD.

I have read this agreement and agree to abide by its terms.

Name (Print or Type): ____

Signature: ____________________________________ **Date:** _____________

31 Appendix P: Investigator Signature of Agreement

<u>Investigator Signature of Agreement</u>

The Eunice Kennedy Shriver National Institute of Child Health and Human Development
Reproductive Medicine Network

<u>Title:</u> **Improving Reproductive Fitness through Pretreatment with Lifestyle Modification in Obese Women with Unexplained Infertility: (FIT-PLESE FEMALE)**

Version: 1.0

<u>Principal Investigator:</u>

I, *[Insert PI's name]* , the Principal Investigator for *[Insert Institute Name]*, hereby certify that I have read and agree to conduct this study in accordance with this protocol on behalf of all RMN Investigators and research staff from my site.

I will conduct the clinical study as described and will adhere to the *Code of Federal Regulations,* Title 21 and Title 25, Part 46, Good Clinical Practices (GCP), International Conference on Harmonisation (ICH), and the Declaration of Helsinki. I have read and understood the contents of the Protocol.

The signature of the investigator below indicates acceptance of the protocol and a complete understanding of the investigator commitments as outlined in Form FDA 1572, Statement of Investigator.

____

Principal Investigator's Signature Date

____

Printed Name Date

Reproductive Medicine Network
Funded by *Eunice Kennedy Shriver* NICHD

Improving Reproductive Fitness through Pretreatment with Lifestyle Modification in Obese Women with Unexplained Infertility: (FIT-PLESE FEMALE)

Protocol Leader: Richard S. Legro, MD
Data and Coordination Leader: Heping Zhang, PhD

Version 4.0

Supported by:
The Eunice Kennedy Shriver National Institute of Child Health and Human Development (NICHD)

Supported by Grants U10 HD38992 (to R.S.L.), U10 HD27049 (to C.C.), U10HD055925 (to H.Z.), U10 HD39005 (to M.P.D.), U10 HD077680 (to K.H.), U10HD077844 (to A.S.), and U10HD077841 (to M.C)

Study Intervention Provided by: No outside support
Sponsor of IND(IDE): Not Applicable

Prepared by:

The RMN Data Coordination Center

Collaborative Center for Statistics in Science

Yale University School of Public Health

300 George Street, Suite 523

New Haven, CT 06511

(203) 785-5185

Last updated: July 3, 2017

Protocol Subcommittee

Group email address: rmn.fit@mailman.yale.edu

Name	E-mail
**Richard Legro, MD**	**rlegro@pennstatehealth.psu.edu**
Esther Eisenberg, MD, MPH	eisenbes@mail.nih.gov
Heather Huddleston, MD	HuddlestonH@obgyn.ucsf.edu
Penny Kris-Etherton	pmk3@psu.edu
Alex Polotsky, MD	Alex.polotsky@ucdenver.edu
Nanette Santoro, MD	nanette.santoro@ucdenver.edu
David Sarwer, PhD	dsarwer@mail.med.upenn.edu
Peter Snyder, MD	pjs@mail.med.upenn.edu
Anne Z. Steiner, MD, MPH	anne_steiner@med.unc.edu
J. C. Trussell, MD	jctrussell1@verizon.net
Heping Zhang, PhD	heping.zhang@yale.edu

Table of Contents

1 STUDY SYNOPSIS....10

1.1 OBJECTIVES....10
1.2 HYPOTHESIS....10
1.3 PATIENT POPULATION....10
1.4 STUDY DESIGN....10
1.5 TREATMENT....10
1.6 PRIMARY EFFICACY PARAMETER....11
1.7 SECONDARY EFFICACY PARAMETERS....11
1.8 STATISTICAL ANALYSIS....11
1.9 ANTICIPATED TIME TO COMPLETION....12
1.10 REGULATORY COMPLIANCE....12

2 STUDY OBJECTIVES....13

2.1 PRIMARY AIM....13
2.2 SECONDARY AIM....13
2.3 TERTIARY AIMS....13

3 BACKGROUND....15

3.1 RATIONALE....15
3.2 OBESITY AND REPRODUCTION....15
3.3 ADVERSE EFFECTS OF LIFESTYLE AND WEIGHT LOSS ON PREGNANCY....16
3.4 CURRENT STATUS OF OBESITY MANAGEMENT....17
3.5 GOOD BIRTH OUTCOME....19
3.6 SUPPORTING DATA....19
3.7 SUMMARY OF AMIGOS DATA....25

4 STUDY DESIGN....28

4.1 OVERVIEW....28
4.1.1 Treatment Design....29
4.1.2 Study Population....29

5 SELECTION AND ENROLLMENT OF SUBJECTS....30

5.1 INCLUSION CRITERIA....30
5.2 EXCLUSION CRITERIA....30
5.3 STUDY TERMINATION CRITERIA....31
5.4 STUDY ENROLLMENT PROCEDURES....32
5.4.1 Recruitment....32
5.5 PROCEDURES FOR TRACKING SOURCES OF SUBJECTS AND THEIR DISPOSITION....33
5.6 OBTAINING INFORMED CONSENT....33
5.7 INTERVENTION GROUP ASSIGNMENT....33

6 STUDY INTERVENTIONS....34

6.1 INTERVENTIONS, ADMINISTRATION AND DURATION....34
6.1.1 Determine Baseline Physical Activity....35
6.2 HANDLING OF STUDY INTERVENTIONS....36
6.3 CONCOMITANT INTERVENTIONS....36
6.4 ADHERENCE ASSESSMENT....36

7 CLINICAL AND LABORATORY EVALUATIONS....37

7.1 SCHEDULE OF EVALUATIONS....37
7.2 TIMING OF EVALUATIONS....39
7.3 SPECIAL INSTRUCTIONS AND DEFINITIONS OF EVALUATIONS....47
7.3.1 History and Demographics....47

7.3.2 Physical Exam ... *48*
7.3.3 Ultrasound and Imaging Exams ... *50*
7.3.4 Biospecimens ... *50*
7.3.5 Questionnaires ... *51*

8 MANAGEMENT OF ADVERSE EXPERIENCES ... 52

8.1 EXPECTED ADVERSE EXPERIENCES ... 52
8.1.1 Phase I ... *52*
8.1.2 Phase II ... *52*
8.1.3 Expected Serious Adverse Events ... *52*

9 CRITERIA FOR MODIFICATION OR DISCONTINUATION OF STUDY INTERVENTIONS ... 53

9.1 PHASE I ... 53
9.1.1 Intensive Lifestyle Modification ... *53*
9.1.2 Standard Lifestyle Intervention ... *53*
9.2 PHASE II ... 54
9.3 PHASE III AND IV ... 54

10 STATISTICAL CONSIDERATIONS ... 55

10.1 GENERAL DESIGN ISSUES ... 55
10.2 RATIONALE FOR DESIGN ... 55
10.3 OUTCOMES ... 55
10.3.1 Primary Outcome Measurements ... *55*
10.3.2 Secondary Outcome Measurements ... *55*
10.4 SAMPLE SIZE AND ACCRUALS ... 56
10.4.1 Sample Size and Power Calculations ... *56*
10.4.2 Accrual ... *56*
10.5 DATA MONITORING ... 57
10.6 STUDY MONITORING ... 57
10.7 DATA AND SAFETY MONITORING BOARD ... 57
10.8 REPORTING ... 58
10.9 DATA ANALYSIS ... 58

11 DATA COLLECTION, SITE MONITORING AND ADVERSE EXPERIENCE REPORTING ... 61

11.1 RECORDS TO BE KEPT ... 61
11.1.1 Maintenance/Retention of site records ... *61*
11.2 ROLE OF DATA MANAGEMENT ... 61
11.3 QUALITY ASSURANCE ... 61
11.3.1 Data Entry and Forms ... *61*
11.3.2 Features of Data Management System ... *61*
11.3.3 Data Security ... *61*
11.3.4 Data Quality Control ... *62*
11.3.5 Obligation of the Investigator ... *63*
11.3.6 Establishment of Pregnancy Registry ... *63*
11.3.7 Regulatory Requirements ... *64*
11.3.8 Protocol Amendments ... *64*
11.4 ADVERSE EVENT REPORTING ... 64
11.4.1 Serious Adverse Events ... *64*

12 HUMAN SUBJECTS ... 67

12.1 INSTITUTIONAL REVIEW BOARD (IRB) REVIEW AND INFORMED CONSENT ... 67
12.2 SUBJECT CONFIDENTIALITY ... 67
12.3 STUDY MODIFICATION/DISCONTINUATION ... 67

13 PUBLICATION OF RESEARCH FINDINGS ... 68

13.1 OVERALL POLICY 68
13.2 MAIN STUDY 68
13.2.1 Major Publications (Tables 13-14) *68*
13.2.2 Minor Publications *70*
13.2.3 Ancillary Study *70*
13.2.4 Pilot Study *71*
13.2.5 Related Publications *71*
13.2.6 Outside Studies *71*
13.2.7 Presentations *72*

14 REFERENCES 73

15 HUMAN SUBJECTS/INFORMED CONSENT/FEMALE 79

16 HUMAN SUBJECTS/INFORMED CONSENT/MALE 106

17 APPENDIX A: RISK FACTORS FOR GENETIC DISORDERS 121

18 APPENDIX B: LIST OF COMMON MEDICATIONS EXCLUDED OR REQUIRING WASH-OUT PERIOD 125

19 APPENDIX C: SF 12 HEALTH SURVEY 127

20 APPENDIX D: FEMALE SEXUAL FUNCTION INDEX 130

21 APPENDIX E: PHQ-9 135

22 APPENDIX F: FEMALE SEXUAL DISTRESS SCALE (FSDS - REVISED 2005) 137

23 APPENDIX G: FERTIQOL 138

24 APPENDIX H: STOP BANG QUESTIONNAIRE 139

25 APPENDIX I: EPWORTH SLEEPINESS SCALE 140

26 APPENDIX J: INTERNATIONAL INDEX OF ERECTILE FUNCTION (IIEF) 141

27 APPENDIX K: DIET HISTORY QUESTIONNAIRE II (DHQ II) 144

28 APPENDIX L: AUTOMATED SELF-ADMINISTERED 24-HOUR RECALL (ASA24) 184

29 APPENDIX M: QADAM QUESTIONNAIRE 195

30 APPENDIX N: PSYCHOSEXUAL DAILY QUESTIONNAIRE 196

31 APPENDIX O: SUN EXPOSURE AND BEHAVIOUR INVENTORY (SEBI V2) 197

32 APPENDIX P: DSMB CHARTER 200

33 APPENDIX Q: INVESTIGATOR SIGNATURE OF AGREEMENT 209

FIGURE 1. RELATIONSHIP BETWEEN TOTAL CALORIES AT THE TIME OF CONCEPTION AND BIRTH RATE IN THE NETHERLANDS DURING THE POST WAR FAMINE 1944-46 15
FIGURE 2: LIVE BIRTH PROBABILITY BY TREATMENT ARM IN OWL PCOS TRIAL 21
FIGURE 3: FIT-PLESE STUDY FLOWCHART 28
FIGURE 4: MODIFIED FERRIMAN-GALLWEY HIRSUTISM SCALE 48
FIGURE 5: SERIOUS ADVERSE EVENT FLOW CHART 66

TABLE 1: EFFECT OF DURATION OF EXERCISE ON IVF AND PREGNANCY OUTCOMES IN A COHORT OF WOMEN UNDERGOING IVF TREATMENT 16

Table 2 Summary of RCTs from recent systematic review ([1] examining interventions on weight and fertility outcomes in overweight or obese women). 20
Table 3: Effect of preconception intervention on ovulation, pregnancy, and live birth............ 22
Table 4: Baseline values of select reproductive/metabolic parameters, and mean change at the end of Phase I (four months of treatment) from baseline. P-value is among the 3 groups for mean change 23
Table 5: Adverse events with significant differences between groups by Phase of Study and all Serious Adverse Events. 24
Table 6: Live birth per treatment cycles of AMIGOS patients with BMI>=30............ 26
Table 7: Study Visits and Procedures............ 37
Table 8: Female Local Laboratory Screening Blood Tests 40
Table 9: Study Medication and Dosing 44
Table 10: Acne Grading Method 49

Acronyms

Advisory Board	AB	Institutional Review Board	IRB
American Congress of Obstetricians and Gynecologists	ACOG	Intrauterine Insemination	IUI
Anti-Mullerian Hormone	AMH	Investigational New Drug	IND
Assessment of Multiple Intrauterine Gestations from Ovarian Stimulation	AMIGOS	Large Gestational Age	LGA
Assisted Reproductive Technologies	ART	Logistic Regression	LR
Aromatase Inhibitors	AI	Luteinizing Hormone	LH
Clinical Report Form	CRF	National Institute of Child Health and Human Development	NICHD
Clomiphene Citrate	CC	Non-obstructive Azospermia	NOA
Centers for Disease Control	CDC	Ovarian Stimulation	OS
Data Coordination Center	DCC	Ovarian Hyperstimulation Syndrome	OHSS
Data and Safety Monitoring Board	DSMB	Principal Investigator	PI
Deoxyribonucleic Acid	DNA	Progesterone	P4
Estrogen	E	Pregnancy in Polycystic Ovary Syndrome Study	PPCOS
Estradiol	E2	Pregnancy of Unknown Location	PUL
Estrogen Receptor	ER	Protected Heath Information	PHI
Food and Drug Administration	FDA	Quality of Life	QOL
Follicle Stimulating Hormone	FSH	Reproductive Medicine Network	RMN
Female Sexual Distress Scale	FSDS	Serious Adverse Event	SAE
Female Sexual Function Index	FSFI	Sex Hormone Binding Globulin	SHBG
Good Birth Outcome	GBO	Single-Nucleotide Polymorphism	SNP
Health Insurance Portability and Accountability Act	HIPAA	Specialized Cooperative Center Programs in Reproductive Research	SCCPIR
Human Investigation Committee	HIC	Sonohysterogram	SHG
Human Chorionic Gonadotropin	hCG	Thyroid-Stimulating Hormone	TSH
Hysterosalpingography	HSG	Total and Free Testosterone	T, FT
Identification	ID	Ultrasound	U/S

International Index of Erectile Dysfunction	IIEF	World Health Organization	WHO
Intent-to-Treat	ITT		
Intramuscular	IM		

1 Study Synopsis

1.1 Objectives

To determine the safety and efficacy of pretreatment with lifestyle modification in obese women with unexplained infertility.

1.2 Hypothesis

An intensive lifestyle modification intervention (which includes caloric restriction, use of an over-the-counter weight loss medication, and increased physical activity) designed to promote a weight loss of approximately 7% of initial body weight is more likely to achieve a good perinatal outcome (i.e. a healthy term normal weight infant) than a recommendation to standard lifestyle modification with increased physical activity (based on publicly available activity recommendations) in obese women with unexplained infertility.

1.3 Patient Population

The population will consist of 380 obese women with unexplained infertility, age 18-40 years old. Subjects must have normal ovulatory function and normal ovarian reserve. Additionally, the couple will have no other major infertility factor: the subject will have at least one patent fallopian tube and a normal uterine cavity, and a partner total motile sperm count of at least 5 million in at least one ejaculate.

1.4 Study Design

This will be a two-arm, multicenter, prospective, randomized clinical trial of a lifestyle modification program with increased physical activity and weight loss (intensive) compared to recommendations to increase physical activity alone with weight maintenance (standard) in women with obesity and unexplained infertility. This 16 week period of lifestyle modification will be followed by an open label empiric infertility treatment regimen consisting of three cycles of ovarian stimulation with oral medication (clomiphene), triggering of ovulation with human chorionic gonadotropin (hCG) and intrauterine insemination (IUI).

1.5 Treatment

The intensive lifestyle modification intervention will consist of caloric restriction (consumption of approximately 1200-1500 kcal/d) centering around the use of meal replacements (Nutrisystem), use of an over-the-counter weight loss medication (Alli, which is brand name Orlistat, a gastric lipase inhibitor which limits gut fat absorption), and increased physical activity (goal of reaching 10,000 steps a day). The pretreatment intervention will last 16 weeks and is designed to promote a weight loss of approximately 7% of total body weight in the intensive group. Women in the standard lifestyle intervention (standard) will receive publicly available written materials that promote engagement in increased physical activity. Detailed instruction of physical activity will not be provided. Participants in both groups will receive activity tracking devices (Fitbit Wireless Activity Tracker) and wireless scales (Fitbit Aria Wireless Activity Scale) to promote adherence to the inventions and to allow monitoring for compliance by study personnel. The pretreatment intervention will last 16 weeks. Both groups will aim to build up to 10,000 steps/day with a recommendation to increase steps from baseline by 500 steps/per week. We will monitor subjects monthly during this preconception intervention. All subjects randomized will receive after 16 weeks of lifestyle modification, regardless of adherence or

success in achieving treatment goals, a standardized empiric infertility treatment. This will consist of ovarian stimulation with an oral medication (clomiphene) followed by ultrasound follicular monitoring, human chorionic gonadotropin (hCG) trigger of ovulation, and a single partner intrauterine insemination (IUI) per treatment cycle for up to three treatment cycles. The goal for both treatment groups will be to maintain levels of physical activity and weight achieved during the pretreatment phase during the empiric infertility treatment phase. Subjects who conceive will be followed throughout pregnancy with the wireless activity monitor and wireless scale. Additionally there will be three brief onsite visits during pregnancy (per trimester at 16, 24, and 32 weeks) for onsite determination of weight, blood pressure, glycemic parameters, and collection of biospecimens. All pregnancy outcomes will be tracked. Subjects who deliver will be encouraged to donate placenta and cord blood to the study repository and then to enroll in our Pregnancy Registry for continued infant follow-up. We will also expand the number and variety of specimens we collect for the repository from both partners including urine and serum, semen, placenta and cord blood.

1.6 Primary efficacy parameter

Cumulative Good Birth Outcome during the study. Good Birth Outcome will be defined as a live birth of an infant born at ≥ 37 weeks, with a birth weight between 2500 and 4000g and without a major congenital anomaly.

1.7 Secondary efficacy parameters

We will assess secondary outcomes including live birth (birth after 20 weeks), time to pregnancy, pregnancy loss rate [including ectopics and Pregnancies of Unknown Location (PULs)], multiple pregnancy rate, pregnancy complication rate including development of gestational hypertension and diabetes, birth weight, neonatal complication rate, predictive factors for response including DNA polymorphisms, sleep quality, sexual function and psychosocial functioning (health-related quality of life and weight-related quality of life).

1.8 Statistical Analysis

The primary analysis will use an intent-to-treat approach examining differences in the good birth outcome rate in the two treatment arms. The study is powered with 80% power and an alpha of 0.05 to detect an absolute 15% difference in good birth outcome (a live birth of an infant born at ≥ 37 weeks, with a birth weight between 2500 and 4000g and without a major congenital anomaly) between the two treatment groups. We project the proportion of good birth outcomes to be 0.25 in the standard lifestyle group and 0.40 in the intensive lifestyle group. The sample size is inflated to reflect a 20% dropout rate, giving us 190 subjects in each group. A logistic regression model will be fit to compare the treatment arms with respect to the primary outcome of good birth outcome while adjusting for the randomization stratification factors of study site and baseline BMI. The analysis of other secondary (supplemental) outcomes measured over time will entail the application of statistical methods that have been developed for correlated data since repeated observations will be made over time on each individual. For secondary outcomes such as weight loss, a linear mixed-effects model will be fit where the main independent variables will be treatment group, time, and their interaction as well as the randomization stratification factors as covariates. Logistic regression models will be used in secondary analyses to evaluate the predictive value of treatment arm, clinical site, prior exposure to ovulation induction drugs, age, and other explanatory variables on binary outcomes

(e.g., singleton live birth, abortion). Cox proportional hazards models and a Kaplan-Meier method will be applied to compare time to pregnancy in the treatment groups.

1.9 Anticipated time to completion

A total of 2.6 years will be required to complete the study after start up; 15 month enrollment period (based on 2 subjects per site/month x 13 sites), 7 month treatment period (4 months lifestyle pretreatment and 3 months ovarian stimulation/IUI), with 9 month additional observation to determine pregnancy outcomes.

1.10 Regulatory Compliance

The DCC is working with The Eunice Kennedy Shriver National Institute of Child Health and Human Development (NICHD) to ensure that clinical study data and regulatory requirements are met regarding the Food and Drug Administration (FDA) code for federal regulations. This trial is registered on http://www.clinicaltrials.gov (NCT#02432209).

2 Study Objectives

2.1 Primary Aim

Our primary goal is to examine the efficacy of an intensive lifestyle modification intervention, which includes caloric restriction by using meal replacements, a weight loss medication (over the counter orlistat) and physical activity recommendations versus a standard lifestyle intervention consisting of increasing physical activity alone on improving good birth outcomes in obese women with unexplained infertility. Implicit in the primary aim is the goal of tracking safety of our interventions at all Phases of the study (through lifestyle modification to infertility treatment to pregnancy to infant).

2.2 Secondary Aim

We will assess secondary outcomes including live birth rate, time to pregnancy, pregnancy loss rate [including ectopics and Pregnancies of Unknown Location (PULs)], multiple pregnancy rate, pregnancy complication rate including development of gestational hypertension and diabetes, birth weight, mode of delivery, neonatal complication rate, predictive factors for response including DNA polymorphisms, stool and vaginal microbiome composition, psychosocial outcomes, and cost effectiveness.

2.3 Tertiary Aims

1. Is there a relationship between amount of weight loss or amount of physical activity during lifestyle modification and ovulatory response and pregnancy?
2. Does physical activity alone lead to healthier pregnancies (i.e. better gestational weight gain, blood pressure change, glycohemoglobin levels)?
3. Is there a biphasic effect of either weight loss or physical activity on conception and pregnancy?
4. Does lifestyle modification affect the menstrual cycle of regularly ovulating women?
5. Is this treatment more cost effective than immediately proceeding with empiric infertility treatment? (compare with AMIGOS data)
6. Does weight loss or physical activity increase sexual function and amount of intercourse?
7. Can sperm microarray identify couples more or less likely to conceive?
8. Does perceived stress and self-reported quality of life by husband and wife influence success? [Assess by SF-12 and PHQ-9]]
9. Is length of luteal phase predictive of pregnancy outcome?
10. Is there a relationship between length of infertility and conception?
11. Is there a difference in pregnancy rate in the first vs. second vs. third vs. fourth cycle?
12. Does weight loss or physical activity reduce the rate of metabolic syndrome?
13. Does the baseline vaginal or stool microbiome effect response to treatment and outcomes.
14. Does treatment effect the stool or vaginal microbiome?
15. Is the stool microbiome heritable to offspring?

16. Are there changes in stress as determined by salivary biomarker of stress according to treatments?

3 Background

3.1 Rationale

3.2 Obesity and Reproduction

Obesity is associated with poor reproductive outcomes in women. This includes a longer time to pregnancy,[1] an association with ovulatory dysfunction,[2] lower conception rates with infertility treatment from ovulation induction to ovarian stimulation to IVF,[3-6] an increased chance of pregnancy loss with conception,[7,8}9] and higher rates of major pregnancy complications such as pre-eclampsia, pre-term labor, and gestational diabetes leading to increased risk of maternal, fetal, and infant morbidity and mortality.[10-12] The epidemiologic evidence linking female obesity to poor reproductive outcomes is massive and can be overwhelming. Consequently experts, major medical societies and public health programs have endorsed or mandated weight loss in obese women before initiating infertility therapy.[13,14] For example, in the United Kingdom infertility treatment is discouraged in women with a BMI > 35,[15] and in New Zealand, it is forbidden to provide public assistance to treat infertility in women with a BMI > 32.[16,17] While obesity is associated with poor reproductive outcomes, it is not known if weight loss by obese women improves reproductive outcomes. Common sense supports this belief, as well as extrapolated data from lifestyle modification studies to prevent diabetes or to secondarily prevent cardiovascular events. *Unfortunately the clinical trial evidence that lessening obesity improves reproductive outcomes is less than underwhelming, it is non-existent.* A recent Cochrane meta-analysis highlighted the complete absence of randomized trials indicating a benefit of weight loss on pregnancy rates or pregnancy outcomes.[18]

Figure 1. Relationship between total calories at the time of conception and birth rate in the Netherlands during the post war famine 1944-46

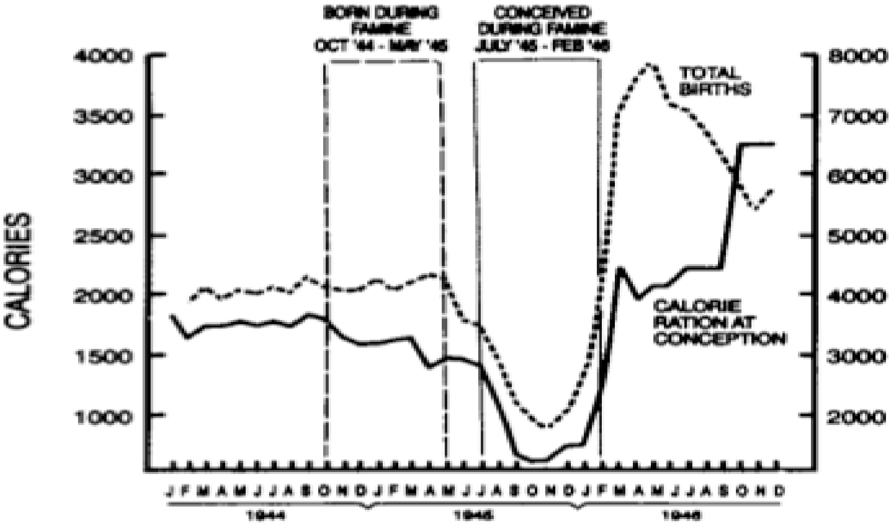

While no one can deny the health benefits of weight loss and lifestyle modification on overall health, the unique energy requirements for ovulation and pregnancy may create a scenario where weight loss, or the stress of exercise, may produce counterintuitive and adverse reproductive outcomes. Weight loss, or at least meaningful weight loss, requires a significant investment of

resources, effort, and time; therefore, it is imperative, especially where age remains the single most important predictor of pregnancy success in infertility treatment,[19] that we not waste time without clear evidence of benefit.

Why is weight and body fat critical to reproduction? A certain threshold of body fat is essential to reproductive maturation and menarche. Without it menarche is delayed. This concept known as the Frisch Hypothesis[20] has become widely accepted and supported by our own studies.[21] The rapid decline in the age of menarche over the last 100 years in most developed countries is attributed to improved food supplies and increased fat mass in prepubertal girls.[22] Further loss of fat mass due to disorders such as anorexia nervosa or among high performance athletes (such as gymnasts or runners) is associated with secondary amenorrhea. There does not appear to be an upper threshold of body fat that causes similar ovulatory dysfunction. The notion that obesity causes PCOS is debunked by many authors and certainly in many parts of the world such as East and South Asia where most women with PCOS are normal weight.[23] Further there is evidence from detailed studies of morbidly obese women undergoing bariatric surgery that their ovulatory function is normal, though they may have longer follicular phases or relatively inadequate luteal phases, both of which tend to normalize with massive weight loss following surgery.[24-26]

3.3 Adverse effects of lifestyle and weight loss on pregnancy

It is difficult to accept the hypothesis that weight loss and lifestyle modification may not have the desired effect on improving fertility in obese women. However there is evidence from the epidemiologic literature that forced caloric restriction is associated with decreased population fecundity. In developing nations, famines are often associated with marked declines in birth rates (ranging from 25-26%). One of the most closely studied famines in the developed world occurred in the Netherlands immediately after WWII from 1944-1946. The observation of this nutritionally challenged birth cohort formed the basis for the Barker Hypothesis regarding developmental origins. Interestingly the birth rate plummeted with the onset of the famine

Table 1: Effect of Duration of Exercise on IVF and pregnancy outcomes in a cohort of women undergoing IVF treatment

	No Exercise (n=859)	All Categories (n=1,305)	Category 1 1–3 h/wk for 1–9 y (n=221)	Category 2 4 h or more/wk for 1–9 y (n=230)
Cycle cancellation	1.0 (referent)	1.2 (0.8–1.8)	1.0 (0.5–1.9)	2.8 (1.5–5.3)*
Failed fertilization	1.0 (referent)	1.1 (0.7–1.6)	0.7 (0.4–1.6)	1.2 (0.6–2.6)
Implantation failure	1.0 (referent)	1.3 (1.0–1.6)†	1.0 (0.7–1.5)	2.0 (1.4–3.1)*
Pregnancy loss	1.0 (referent)	1.3 (1.0–1.8)	1.3 (0.8–2.2)	2.0 (1.2–3.4)*
Successful live birth	1.0 (referent)	0.8 (0.7–1.0)	0.9 (0.6–1.3)	0.6 (0.4–0.8)*

and rapidly climbed back following the end of the famine, implying that rather than fat supply, caloric restriction and restoration were central to the birth rate (**Figure 1**). The hazards of attempting conception and maintaining conception during periods of acute caloric restriction are also supported by the experience with bariatric surgery leading to recommendations to avoid conception in the 6-12 months after surgery during the period of greatest weight loss and gastric restriction. There are also examples from the infertility literature that acute and severe caloric restriction during the period of infertility treatment is associated with reduced fecundity.

For example, one trial severely calorie restricted (456-1200 kcal/d) obese infertile women during an IVF cycle prior to and during the IVF cycle up to oocyte retrieval and noted poor fertilization and implantation rates and was stopped prematurely.[27] We note here that publishing such adverse results of studies is rare in our field, and the absence of data regarding benefit and lack of harm may in part reflect a publication bias. There is also evidence that exercise, or at least relatively more exercise in a population of women undergoing IVF, may be associated with lower implantation rates, but once implanted higher delivery rates, implying a bi-phasic association with fecundity (**Table 1**).[28] There is also evidence from Norway that women who exercise more over a lifetime are more likely to be nulliparous.[29] Exercise, especially when associated with energy deficit, has been associated with inducing menstrual disturbances and anovulation in eumenorrheic women.[30-32] It is important to highlight these potential adverse effects of lifestyle modification to underscore the importance of a prospective randomized trial to better address the risk benefit ratio.

3.4 Current Status of Obesity Management

A program of diet, physical activity, and behavior therapy (i.e., lifestyle modification) is the cornerstone of treatment for most obese individuals.[33] In trials conducted in academic medical centers, persons treated by a 1200-1500 kcal/d diet, combined with regular exercise and a comprehensive program of group or individual lifestyle modification, lose approximately 7%-10% of initial weight in 20-26 weeks.[34-36]

<u>Effects of exercise alone in lifestyle modification on health</u> It is generally agreed that programs emphasizing exercise alone (as compared to or combined with weight loss) generally have no or little associated weight loss.[37] However exercise alone, with respect to the prevention and treatment of diabetes and cardiovascular disease has been found to have clear benefit with minimal risks. Programs of exercise alone (without dietary modification) have been found to significantly improve glucose and glycohemoglobin levels in patients with type 2 diabetes.[38] Further multiple large cohort studies have found that increasing amounts of habitual aerobic fitness and/or physical activity are associated with significantly lower cardiovascular and overall mortality to a much greater extent than could be explained by decreases in glucose lowering alone.[37-39] Exercise alone has also been associated with favorable changes in body composition,[40] i.e. decrease in centripetal obesity, which may favorably impact reproduction.[41] No significant effect on sex hormones was noted in an aerobic exercise intervention (150 mins/week) in a cohort of premenopausal women compared to control women.[42] In our FIT-PLESE study we will be proposing exercise alone as a comparison to weight loss through caloric restriction, anticipating minimal weight loss but an overall improvement in fitness and health which may favorably impact reproduction.

<u>Pharmacotherapy</u> Following the NHLBI's stepped care algorithm,[33,43] pharmacotherapy is an option for persons unable to lose 10% of initial weight with lifestyle modification alone and who have a BMI > 30 kg/m^2 (or > 27 kg/m^2 in the presence of co-morbid conditions). Pharmacotherapy has a checkered history with many medications being removed post FDA approval due to unacceptable cardiovascular complications, including fenfluramine and sibutramine, and others never achieving FDA approval, such as rimonabant, due to concerns about mood changes and suicidality. Although a number of weight loss medications have recently been approved by the FDA, these medications lack a proven track record of safety and efficacy, and this had discouraged us from incorporating them into a clinical trial. However, there is a medication that has survived the test of time and found to be both safe and

efficacious, which is orlistat. Orlistat is a gastric and pancreatic lipase inhibitor that induces weight loss by blocking the absorption of about one-third of the fat contained in a meal. [44-47] This medication combined with a modest program of lifestyle modification, is associated with mean loss of 8% - 10% of initial weight and improvements in metabolic risk factors.[44-53] As evidence of its safety, this medication is available in a reduced dose as an over the counter (OTC) medication (Alli). Further, as noted below in our preliminary data, we have successfully used this OTC medication without major adverse effects in a trial of lifestyle modification in obese women with PCOS seeking pregnancy. Overall the medication is well tolerated, and to patients it provides a "gut check" as excessive fat consumption will lead to a powerful wave of steatorrhea.

<u>Increasing initial weight loss</u> Although loss of as little as 5% of initial weight is associated with improvements in health, larger losses are generally associated with greater improvements in glycemic control, B/P, and lipids [43,54,55] and presumably reproductive function. The use of portion-controlled servings of conventional foods,[56,57] as well as liquid meal replacements,[58,59] is effective in increasing initial weight losses by approximately 3 kg, as compared with the prescription of a self-selected diet of conventional foods with the same calorie goal. Portion-controlled servings, by providing foods of pre-determined quantity and energy content, reduce the obese individual's tendency to underestimate his/her calorie intake,[60] which has been found to be as great as 50% when a self-selected diet of conventional foods is consumed.[61] The addition of pharmacotherapy to lifestyle modification also increases initial weight loss by approximately 4 to 6 percentage points (e.g., from 6% to 10%), compared with lifestyle modification alone.[46,62] This additive benefit is observed whether participants receive a modest program of lifestyle modification (e.g., a few visits with a dietitian)[62] or a comprehensive program (weekly group meetings).[63] We successfully used meal replacements in our OWL PCOS trial discussed below, and note that this contributed substantially to meeting our goal of a 7% weight loss in a 16 week pretreatment period in obese women with PCOS.

<u>Barriers to Treating Obesity in Medical Practice</u> Obesity is the most frequently encountered problem in primary care practice and the one least likely to be addressed. Primary care physicians, by their own report (or that of their patients) do not discuss weight management with 50% or more of their overweight and obese patients.[64,65] *Ob/Gyns, for instance, tend to not refer their patients for lifestyle modification interventions for weight loss or to use weight loss medications.[66]* Physicians' inactivity in this area appears to be attributable to multiple factors including providers' perceptions that: 1) obesity is a problem of willpower, not medicine (representing a bias against obesity); 2) broaching the subject of weight control is uncomfortable for both patient and provider; 3) most therapies are ineffective; and 4) treatment is not adequately reimbursed.[67,68] Practitioners also believe they lack the training and time to provide adequate weight counseling. Other countries with socialized medical systems have developed multi-specialty interventions to treat obesity in women seeking conception. The Fertility Fitness program, established in an urban setting in Australia, involved weekly dietetic and behavioral intervention in a group environment, and used a multidisciplinary team approach, including an obstetrician/gynecologist, psychiatrist, dietician, fitness professional, and nurse.[69] This program reported restoration of ovulation in women with PCOS, improvement in pregnancy, and reduction in miscarriage rates,[70] but the dropout rate was 30% due to the intensive monitoring and multiple weekly visits that were part of the program. These results, however, were obtained without randomization or alternative

treatment, and there was no standardized infertility treatment following the intervention. Further, we believe that such a program, given its expense and lack of a U.S. clinical practice model, is impractical and unlikely to result in a widespread adaption. We have instead sought to create a more streamlined approach to lifestyle modification that can be readily understood by patients and clinicians, and easily incorporated into clinical practice.

3.5 Good Birth Outcome

In this protocol we propose to narrow the primary outcome of a RMN trial from live birth, traditionally defined as any delivery beyond 20 weeks to one that reflects the desires of our patients and the demands of public health, i.e. a good perinatal outcome and specifically a good birth outcome. A sole focus on live birth as the metric of ART success in the U.S. has led to practices which encourage multiple embryo transfer to ensure high live birth rates, at the cost of an unacceptably high multiple pregnancy rate and among the multiple births a higher risk of adverse maternal (gestational hypertension, pre-eclampsia, gestational diabetes, cesarean delivery and hospital admission and neonatal (low birth weight, prematurity, NICU admission, birth defects) outcomes.[71,72] A recent study of the National ART Surveillance Group at the CDC defined a "good perinatal outcome" as "a singleton or twin term live birth with normal birth weight neonates".[73]

When "good perinatal outcome" was substituted for live birth, transfer of one embryo significantly improved the chance for this compared to multiple embryo transfers in women < 35 years old as well as women 35-37 years old. It is estimated that in the U.S. in 2011 a total of 36% of twin births and 77% of triplet and higher-order births resulted from conception assisted by fertility treatments.[75] Most of the twin births are thought to be due to ovarian stimulation and not IVF, and the rate of twin births in the U.S. has continued to increase over recent years, despite the dramatic reduction in triplet and other high order pregnancies (related to fewer embryos transferred during IVF).[75] We believe that the goals of any infertility treatment, either ovarian stimulation or IVF should be preferably a pregnancy with a good perinatal outcome.[75] We note a similar pattern with ovarian stimulation.[74] Gonadotropins for couples with unexplained infertility has a significantly higher live birth rate, but due to the high multiple rate, a lower proportion of good perinatal outcomes. In this study we have chosen a good perinatal outcome, revised here to exclude LGA babies (birth weight ≥ 4500g) as they pose a significantly greater risk for poor perinatal outcomes due to macrosomia such as operative delivery, perineal trauma, shoulder dystocia, fetal injury, NICU admissions etc., especially in obese mothers. Because we are focusing on livebirth, we have altered the name from "good perinatal outcome" to "good birth outcome" in our protocol. Good birth outcome is also a term more comprehensible to our target patient population.

3.6 Supporting Data

<u>Systematic Review and Meta-analyses</u>

A recent systematic review and meta-analysis was performed examining the effects of pretreatment weight loss on subsequent fertility.[1] Of note, these authors reviewed 499 published records and could locate only 2 randomized controlled trials, one of which was described as a pilot trial.[76] (An excerpt is found in **Table 2**) Both of these were small, with less than 100 total subjects in each trial, included various causes of infertility, not just unexplained, and utilized IVF for infertility treatment as opposed to less invasive strategies. The treatment effect was large in the Sim et al trial[77] (absolute difference in live birth rate of 30% favoring

weight loss), and less so in the Moran et al trial,[76] absolute difference in live birth rate of 5%, favoring weight loss. <u>We conclude that there have been a paucity of well designed RCTs in this field and that available data from cohort studies and the two RCTs support a benefit to weight loss prior to infertility treatment.</u>

Table 2 Summary of RCTs from recent systematic review ([1] examining interventions on weight and fertility outcomes in overweight or obese women).

Authors, year, reference	Study design	Type of weight loss intervention and description	Duration of weight loss intervention (follow-up)	Mean age in years and/or (range)	Sample size (intervention/control or drop-out)	Cause of infertility	Main findings for body mass index and fertility			Quality of study
							Weight loss	Pregnancy rate and live birth outcomes	Other	
Clark *et al.*, 1995 (23)	Cohort study (prospective)	Diet, exercise and behavioural support	6 months (12 months)	(21–40)	18 (13/5)	Anovulation with previous clomiphene resistance	6.3 ± 4.2 kg (intervention); 1.4 ± 1.8 kg (comparison); $P < 0.001$. No alteration in waist-to-hip ratio	Pregnancy rate: 85% (11/13), 55% (6/11) were naturally conceived (intervention); no pregnancies (comparison) Live births: 77% (10/13)	Ovulation: 92% (12/13) women were ovulating spontaneously (intervention); none (comparison)	Weak
Clark *et al.*, 1998 (24)	Cohort study (prospective)	Diet, exercise behavioural support	6 months (12 months)	(21–40)	87 (67/20)	Various, including anovulation and PCOS	10.2 ± 4.3 kg (intervention); 1.2 ± 3.6 kg (control); $P < 0.001$	Pregnancy rate: 78% (52/67), 35% (18/52) were naturally conceived (intervention); no pregnancies (comparison) Live births: 67% (45/67)	Ovulation: 90% (62/69) of the previously anovulatory women were ovulating spontaneously (intervention); none (comparison)	Weak
Galletly *et al.*, 1996 (25)	Cohort study (prospective)	Diet, exercise and behavioural support	6 months (21–36 months)	(22–40)	58 (37/21)	Various	6.2 ± 4.5 kg; $P < 0.001$. No comparative group data was reported for any parameter	Pregnancy rate: 78% (29/37), 24% (7/29) were naturally conceived (intervention) Live births: not reported		Weak
Sim *et al.*, 2014 (21)	RCT	Diet (including VLED), exercise and behavioural support	3 months (12 months)	(18–37)	49 (27/22)	Various	6.6 ± 4.6 kg (intervention); 1.8 ± 3.6 kg (control); $P = 0.001$ WC: 8.7 ± 5.6 cm (intervention); 1.0 ± 6.3 cm (control); $P = 0.001$	Pregnancy rate: 48% (13/27), 23% (3/13) were naturally conceived (intervention); 14% (3/22), of which none were naturally conceived (control); $P = 0.014$ for pregnancy rate Live births: 44% (12/27) (intervention), 14% (3/22) (control); $P = 0.02$	ART cycles taken to achieve pregnancy: 2.46 (intervention), 4.33 (control); $P = 0.01$	Moderate
Moran *et al.*, 2011 (22)	RCT	Diet and exercise	5–9 weeks prior to oocyte pick-up (not reported)	(18–40)	38 (18/20)	Various	3.8 ± 3.0 kg (intervention); 0.5 ± 1.2 kg (control); $P < 0.001$ WC: 5.3 ± 4.6 cm (intervention); 3.5 ± 3.5 cm (control); $P = 0.22$	Pregnancy rate: 67% (12/18) (intervention); 40% (8/20) (control); $P = 0.12$; natural conceptions not reported Live births: 39% (7/18) (intervention), 25% (5/20) (control); $P = 0.48$		Moderate

Preliminary Data from a Randomized Trial of Preconception Inverventions in women with Polycystic Ovary Syndrome (PCOS)

Figure 2: Live Birth probability by Treatment Arm in OWL PCOS trial

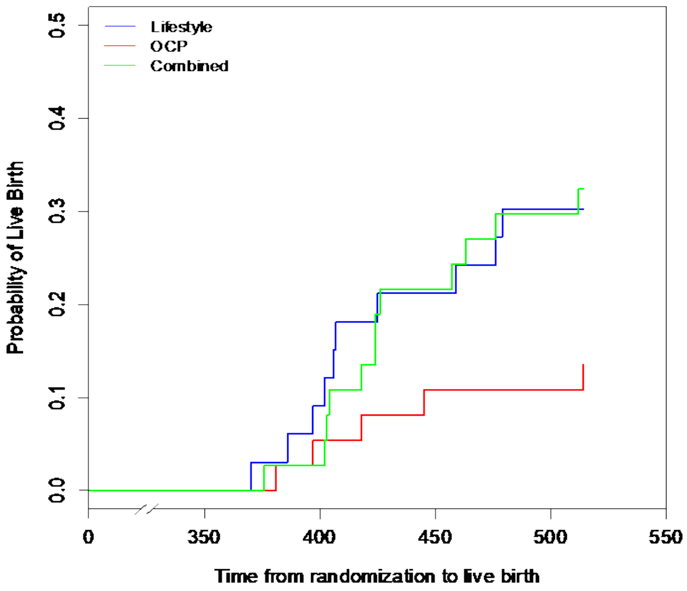

Overview We present now preliminary data from a randomized preconception lifestyle modification program that we are completing in obese women with PCOS. Though the target population in our FIT-PLESE trial is obese women with unexplained infertility, we believe the lessons from our preliminary trial are very relevant to this protocol. These lessons include that we can achieve substantial and projected weight loss in a finite period with meal replacements and orlistat, that our drop-out rates at all time points in in the trial (including lifestyle modification and infertility treatment phases) are low, that adverse event rates are minimal and serious adverse event rates absent, and that there may be a substantial treatment benefit in terms of pregnancy and live birth to lifestyle modification.

OWL PCOS study The OWL PCOS Study is a randomized, open-label clinical trial in overweight/obese PCOS women sponsored by an RO1 grant with Dr. Legro as PI and Drs. Dodson, Penny Kris-Etherton (Penn State) and Drs. Coutifaris and Sarwer (U Penn) as Co- Investigators (age 18-40 years old, BMI 27-42 km/m^2, and no other major infertility factors), of three preconception interventions (Phase I) followed by 4 cycles of ovulation induction with CC to all groups (Phase II), followed by pregnancy for those who conceive up to live birth (Phase III). The three treatment arms were: Lifestyle: weight loss through a regimen of weight loss drug/meal replacements/exercise enforced by behavioral modification with a target of 5-10% weight loss, OCP: Hormonal suppression through continuous oral contraceptive pills (OCP), and Combined: the simultaneous combination of both the Lifestyle Arm and the OCP arm, i.e. weight loss with hormonal suppression. The primary hypothesis is that Combined > OCP > Lifestyle at achieving live birth. It was powered for 246 subjects, and we randomized 149 subjects. After randomization, this was an open label study so that we were able to track the outcomes of our

participants. Based on these findings (**Table 3**) and the recommendations of our DSMB, we stopped enrollment in December 2012, and followed the final subjects through to live birth.

<u>Main Outcomes:</u> The primary outcome for OWL PCOS was live birth. As seen in the Kaplan Meier Curve in Figure 2 those women randomized to OCP (hormonal suppression, red curve) had the lowest probability of live birth, though it was not statistically significant (Table 3). Live birth rates were highest in Lifestyle and Combined arms (weight loss groups with or without hormonal suppression), which is less in Arm B-OCP compared to the other arms (**Figure 2**) These curves parallel the pregnancy rate curves with no differences in pregnancy loss between groups. (**Table 3**) Both Lifestyle and Combined treatments are superior to OCP, and also superior to extracted data from the PPCOS I and II studies (Live Birth Rate after 4 cycles CC/Placebo in PPCOS I =18.6% (29/209) and after 4 cycles CC in PPCOS II 16.2% (61/376). Compared to these results, both Lifestyle and Combined from our trial offered a significantly better ovulation rate[Lifestyle vs Clomiphene: relative risk=1.4; 95% confidence interval=(1.1, 1.7); p=0.005 and Combined vs Clomiphene: relative risk=1.5; 95% confidence interval=(1.2, 1.8); p<0.0001] and a significantly better live birth rate[Lifestyle vs Clomiphene: relative risk=2.5; 95% confidence interval=(1.3, 4.6); p=0.004 and Combined vs Clomiphene: relative risk=2.3; 95% confidence interval=(1.2, 4.3); p=0.01]. These data support the benefit of preconception weight loss in women with PCOS.

Table 3: Effect of preconception intervention on ovulation, pregnancy, and live birth

Outcome	OCP	Lifestyle	Combined	Lifestyle vs. OCP*			Combined vs. OCP*			Lifestyle vs. Combined*		
	no./total no. (%)	no./total no. (%)	no./total no. (%)	Absolute difference % (95% CI)	Risk ratio (95% CI)	p value$	Absolute difference % (95% CI)	Risk ratio (95% CI)	p value$	Absolute difference % (95% CI)	Risk ratio (95% CI)	p value$
**Ovulation**												
Ovulation (total number of ovulations/total treatment cycles)	71/154 (46.1)	82/136 (60.3)	94/140 (67.1)	14.1 (2.8, 25.3)	1.3 (1.0, 1.7)	0.06	20.7 (9.9, 31.6)	1.5 (1.1, 1.9)	0.002	-7.1 (-18.2, 4.1)	0.9 (0.7, 1.1)	0.28
**Pregnancy**												
Conception (serum hCG level>10 mIU/mL)	8/49 (16.3)	16/50 (32.0)	14/50 (28.0)	15.7 (-0.9, 32.2)	1.9 (0.9, 4.1)	0.09	11.7 (-4.2, 27.5)	1.7 (0.8, 3.7)	0.16	4.3 (-13.5, 22.2)	1.1 (0.6, 2.0)	0.72
Clinical pregnancy (fetal heart motion visualized on ultrasound)	7/49 (14.3)	13/50 (26.0)	13/50 (26.0)	11.7 (-3.9, 27.3)	1.8 (0.8, 4.2)	0.16	11.6 (-3.7, 27.0)	1.8 (0.8, 4.2)	0.15	0.3 (-16.8, 17.3)	1.0 (0.5, 1.9)	0.99
**Birth Outcomes**												
Live birth	5/49 (10.2)	13/50 (26.0)	12/50 (24.0)	15.9 (1.2, 30.6)	2.5 (1.0, 6.6)	0.06	13.8 (-0.5, 28.0)	2.3 (0.9, 6.1)	0.08	2.2 (-14.7, 19.0)	1.1 (0.5, 2.1)	0.82

We have also experienced excellent retention in this study. Our combined dropout rate is 19% which is lower than that for clomiphene in PPCOS I (23% and markedly higher for metformin alone) and in PPCOS II (20% overall), which is remarkable considering that the OWL PCOS study is much more intensive, of longer duration, and entails a lifestyle intervention which traditionally has higher dropout rates than a purely pharmaceutical intervention such as the PPCOS trials. (**Figure 2**).

<u>Other Secondary Outcomes:</u> We have collected various data to ascertain the effectiveness of our preconception interventions (**Table 4**). Indeed, we have achieved excellent weight loss in the lifestyle modification arms (A and C) and weight maintenance in Arm B (erasing the concern that study participation *per se* would lead to weight loss). We initially started the study with

sibutramine, an amphetamine-like appetite suppressant, as our weight loss medication, but removed this from our study in January 2010 just prior to the FDA ruling to remove it from the market because of concerns about increased cardiovascular event rates. We substituted over the counter (OTC) orlistat due to its lack of major safety concerns, including no known adverse pregnancy effects. We noted no difference in weight loss between the groups that received orlistat versus those who received sibutramine (data not shown). We examined a number of other parameters to show that the interventions have varied effects on the reproductive and metabolic phenotype of PCOS. These results show that greater suppression of AMH/ovarian volume with OCP suggests a mechanism for lower ovulation/pregnancy rates seen with Arm B (OCP). Our data show that weight loss erases metabolic exacerbations caused by OCP alone. High compliance with medical treatments was supported by pill counts: average compliance for OCP (Arm B) 97% and for orlistat (Arm A) 83%.

Table 4: Baseline values of select reproductive/metabolic parameters, and mean change at the end of Phase I (four months of treatment) from baseline. P-value is among the 3 groups for mean change

	Lifestyle		Continuous OCP		Combined Weight Loss/OCP		
Parameter	Base-line Mean (SD)	Mean Change (95% CI)	Base-line Mean (SD)	Mean Change (95% CI)	Base-line Mean (SD)	Mean Change (95% CI)	P-value
Weight (kg)	96 (16)	-6 (-7,-5)	95 (14)	-1 (-2,-0)	95 (15)	-6 (-7,-5)	<.0001
Waist Circumference (cm)	107 (14)	-6 (-9,-4)	106 (11)	-2 (-5,1)	107 (12)	-6 (-9,-3)	0.04
Mean Arterial Pressure (mmHg)	86 (9)	-2 (-5,2)	88 (8)	2 (-2,6)	89 (10)	-1 (-5,2)	0.29
F-G Score	19 (9)	-0 (-1,1)	17 (8)	-1 (-2,0)	18 (9)	-1 (-2,0)	0.62
Sebum (mcg/cm^3)	98 (50)	7 (-15,29)	101 (59)	-21 (-42,1)	107 (53)	-28 (-50,-7)	0.06
AMH (pmol/L)	9 (6)	-1 (-2,1)	9 (5)	-3 (-4,-2)	9 (5)	-3 (-4,-2)	0.03
Antral Follicle Count (AFC)	54 (34)	-5 (-14,5)	66 (39)	-25 (-34,-15)	58 (34)	-19 (-29,-9)	0.01
Total Ovarian Volume (cm^3)*	21 (14, 27)	1.0 0.9, 1.1)	22 (18, 30)	0.7 (0.6, 0.8)	19 (14, 25)	0.9 (0.8,1.0)	0.002
Fasting Glucose (mg/dL)	87 (9)	-3 (-7,2)	87 (10)	1 (-3,6)	90 (15)	-7 (-12,-3)	0.03
Fasting Insulin (mIU/mL)*	25 (18, 32)	0.9 0.8, 1.1)	24 (17, 32)	1.0 (0.9, 1.2)	24 (15, 33)	1.0 (0.8,1.1)	0.63
TTG (mg/dL)*	122 (99, 171)	1.0 (0.9, 1.1)	117 (94, 155)	1.2 (1.1,1.3)	120 (87, 165)	1.0 (0.9,1.2)	0.02

<u>Adverse Events (AEs)</u> We have had 4 serious AEs to date (all brief hospitalizations): 3 were likely unrelated to study medications/interventions and 1 was an ectopic pregnancy (**Table 5**). We had no congenital anomalies and one IUFD and one neonatal death from a set of twins who delivered at 20 weeks. Potential side effects related to orlistat use are steatorrhea and diarrhea as well as abdominal pain (Table 5). These side effects are common with this treatment. We found the treatments to be equally safe. The fact that both Lifestyle and Combined have similar ovulation and pregnancy rates suggest that weight loss appears to be the predominant effect modifier, and that OCP exposure *per se* is not harmful. We also eliminated the elective use of progestin to induce withdrawal bleeds if anovulatory after CC early in the study.

Table 5: Adverse events with significant differences between groups by Phase of Study and all Serious Adverse Events.

	OCP	Lifestyle	Combined
	**N subjects (%)**	**N subjects (%)**	**N subjects (%)**
**Phase 1: Lifestyle intervention**	**N=49(%)**	**N=50(%)**	**N=50(%)**
Steatorrhea/Diarrhea†‡	0 (0.0)	6 (12.0)	12 (24.0)
Breast Pain†	10 (20.4)	1 (2.0)	6 (12.0)
Abdominal Pain‡	1 (2.0)	5 (10.0)	10 (20.0)
Dysmenorrhea†	8 (16.3)	1 (2.0)	3 (6.0)
Abnormal uterine bleeding¶	4 (8.2)	0 (0.0)	6 (12.0)
**Phase 2: Ovulation induction**	**N=44(%)**	**N=44(%)**	**N=43(%)**
Headache¶	6 (13.6)	4 (9.1)	12 (27.9)
**Serious Adverse Events: All Phases**	3 (6.1)	1 (8.3)	0 (0.0)
Phase 1: Episode of menorrhagia leading to ER visit	1 (2.0)	N/A	N/A
Phase 3: Ectopic pregnancy requiring surgery	1 (2.0)	N/A	N/A
Phase 3: Preterm Delivery of twins at 20 weeks: 1 live birth and 1 IUFD secondary to infection	1 (2.0)	N/A	N/A
Phase 3: Postpartum hospitalization for perforated appendix	N/A	N/A	1 (2.0)

* Indicates Serious Adverse Event (SAE). In addition to the 4 SAE cases reported above, there was one addition al

SAE in a non-randomized subject. The subject developed a pelvic infection following the screening hysterosalpingogram andwas diagnosed with Pelvic Inflammatory Disease which requ ired hospitalization.

†P<0.05 for the comparison between Lifestyle and OCP.

‡ P<0.05 for the comparison between Combined and OCP.

¶ P<0.05 for the comparison between Lifestyle and Combined.

Summary of Results We have learned that our hypothesis about the treatment effects of OCP pretreatment versus lifestyle modification is reversed and analogous to our hypothesis in PPCOS I where we hypothesized that metformin was superior to CC at achieving live birth, and the combination superior to both. [78] In PPCOS I we also found the opposite, i.e. metformin markedly inferior to CC, and no clear benefit to the combination therapy. Similarly with OWL PCOS, we did not observe the trend we theorized, nor did we see that the combination is superior. Rather, lifestyle, alone or in combination with OCP, appeared superior to OCP alone. We do have solid data on which to power future studies about lifestyle modifications and effects on human fecundity. We also can accurately project recruitment rates, as we noted steady, though slower than anticipated recruiting. Although the study overlapped with PPCOS II at both of our centers, Penn State and University of Pennsylvania, there was the same rate of recruiting into OWL PCOS during the year after PPCOS II closed as when both studies were recruiting simultaneously. This indicates to us that this trial is possible in the context of other unexplained infertility trials in the RMN, and that until lifestyle becomes mainstream and open to all comers (not just below a certain BMI as the number one reason for screen failure in OWL PCOS was a BMI ≥ 42), this may not be the first choice therapy of patients.

Further and most importantly, we have demonstrated the feasibility and effectiveness of our preconception interventions on our main parameters and successfully validated our methods for collecting the primary/secondary outcomes of the study. Our pregnancy rates in this trial of overweight/obese women treated with weight loss and 4 cycles of CC is superior to that after 4 cycles of medication alone in either PPCOS I or PPCOS II. Our preliminary results of marked suppression of ovarian volume and AMH levels in the OCP arm and slightly lower ovulation rates suggests a possible ovarian mechanism of action for lower ovulation and pregnancy rates, and therefore we will not utilize this treatment as a control situation in the FIT-PLESE protocol.

Empiric Treatment of Infertility after Lifestyle Modification
We plan to proceed with a standardized empiric therapy for infertility after lifestyle modification that both treatment groups in this trial will receive. We plan to use our experience and data from the AMIGOS trial to power the sample size for this trial and aid in the design of the infertility treatments.

<u>AMIGOS Preliminary Results</u> The AMIGOS trial (Assessment of Multiple Intrauterine Gestations from Ovarian Stimulation) is a randomized trial of three empiric treatments for unexplained infertility: Clomiphene/IUI, letrozole/IUI, or human menopausal gonadotropins/IUI for up to four treatment cycles. This trial randomized 900 couples with unexplained infertility to these treatments and was completed in the last funding cycle of the RMN. We propose to adapt many of the inclusion/exclusion criteria of that study as well as some of the methodology for the empiric treatment phase. Based on the results of this trial, we have elected to use clomiphene/IUI as our empiric treatment for unexplained infertility after completion of the lifestyle modification phase. Although gonadotropin/IUI had the highest live birth rate, it also had a high rate of multiple pregnancy, including high order multiple pregnancy (triplets). Further, clomiphene/IUI had a higher cumulative live birth rate (23.3%) than letrozole/IUI (18.7%). We further extrapolated the data from the obese women (BMI ≥ 30) with unexplained infertility over the first three treatment cycles in the AMIGOS trial and found that clomiphene IUI trended better both in terms of live birth and healthy live birth than letrozole (**Table 6**). We opted for three treatment cycles here to reflect current practice (i.e. to conduct 3 cycles of empiric therapy before moving on) as well as the declining pregnancy and live birth rates we experienced in the fourth treatment cycle (data not shown).

3.7 Summary of AMIGOS data

The AMIGOS data provides reassurance that clomiphene/IUI is an effective empiric infertility treatment and is non-inferior to letrozole (and trending better in terms of live birth and a healthy live birth).

Table 6: Live birth per treatment cycles of AMIGOS patients with BMI>=30

Variable	Clomiphene group (N=70)	Letrozole group (N=90)	Gonadotropin group (N=81)	All (N=241)
		no./total no. (%)		
**Live birth per treatment cycle**				
**Treatment cycle 1**				
Total live birth	4/70(5.7)	9/90(10.0)	16/81(19.8)	29/241(12.0)
Live birth with EGA>=37 wks and 2500<=birth weight<=4000 g	3/70(4.3)	6/90(6.7)	10/81(12.4)	19/241(7.9)
**Treatment cycle 2**				
Total live birth	7/66(10.6)	5/81(6.2)	4/65(6.2)	16/212(7.6)
Live birth with EGA>=37 wks and 2500<=birth weight<=4000 g	6/66(9.1)	3/81(3.7)	3/65(4.6)	12/212(5.7)
**Treatment cycle 3**				
Total live birth	3/59(5.1)	1/76(1.3)	4/61(6.6)	8/196(4.1)
Live birth with EGA>=37 wks and 2500<=birth weight<=4000 g	3/59(5.1)	0/76(0)	3/61(4.9)	6/196(3.1)
**Cumulative Live Birth Rate after 3 cycles**				
Total live birth	14/70(20.0)	15/90(16.6)	24/81(29.6)	53/241(22.0)
Live birth with EGA>=37 wks and 2500<=birth weight<=4000 g	12/70(17.1)	9/90(10.0)	16/81(19.8)	37/241(15.4)

Implications for the FIT-PLESE Protocol

Changes in the proposed FIT-PLESE protocol from the OWL PCOS I Protocol – We propose changes to lower participant burden in terms of time and visits, ease the conduct of the trial for the RMN and RMN sites, and lower the overall cost of the trial. (NOTE: We have conducted the RO1 OWL PCOS trial with direct costs < $500,000 per year, i.e. within the limits of an RO1 grant, most of which goes toward salary support that would be covered by the infrastructure support of the RMN grant).

First we propose to modify the primary outcome from live birth, to a good birth outcome (GBO) which we will define as: Singleton or twin infant (s) born at ≥ 37 weeks between 2500 and 4000g without a major congenital anomaly. We are modifying this to meet both our patient's and public health expectation to produce healthy infants. No one considers a 24 week, 400g premature infant who dies 1 day after delivery an optimal outcome. This is an emerging concept in infertility treatment recently used to examine the outcomes from single embryo transfers in the SART database.[74]

Second we propose to eliminate intensive testing, that sought out mechanisms of action of the preconception interventions. These would not be necessary for a larger Phase III type clinical trial. We propose eliminating serial OGTTs for glucose tolerance changes as well as derived measures of insulin action, serial DXA scans to determine changes in body composition, and serial VO2 submaximal treadmill testing to detect changes in exercise tolerance. All of these require specialized oversight and research facilities, which might not be present at all of the RMN sites, and involve substantial time commitment on the part of research subjects and staff. Additionally some of these tests including OGTT and exercise testing became especially unwieldy in the third trimester, and finally, they are disproportionately expensive. We will continue to track related parameters including homeostatic measures of insulin action and add a glycohemoglobin (HgbA1C) as an integrated measure of glucose homeostasis at several time points in the study.

Third we propose to add both the option to participate in the RMN Repository and Pregnancy Registry to the protocol, and thus we will collect serum, urine, semen, and saliva on the participants who consent (i.e. females, males, and parents for their infants). Additionally on females we will collect vaginal and stool samples for microbiome analysis, and on infants a meconium analysis. We will also encourage collection of placental samples and cord blood.

Fourth we propose to eliminate the upper limit weight cutoff (BMI <42) imposed on us by the study section for the RO1 OWL PCOS study and instead utilize the upper limit utilized in the PPCOS II and AMIGOS trials (which was none). This lack of an upper weight limit for these studies was approved by the NIH program officers, the SC, the Advisory Board, the DSMB, and the site IRBs. This will ease enrollment, as excessive BMI was the number one reason for screen failure in the study (N=182). It also allows access to all who suffer from obesity, not a select few, and thus will increase the external study validity. It is inherently unfair to let an obese woman with unexplained infertility immediately enroll in an empiric infertility study (i.e. AMIGOS), yet deny her the opportunity to first participate in a preconception intervention to improve her reproductive fitness (i.e. the current FIT PLESE Female protocol). We have designed the lifestyle modification intervention such that we are modifying lifestyle through a combined approach of caloric restriction, pharmacotherapy, and increased physical activity.

4 Study Design

Figure 3: FIT-PLESE Study Flowchart

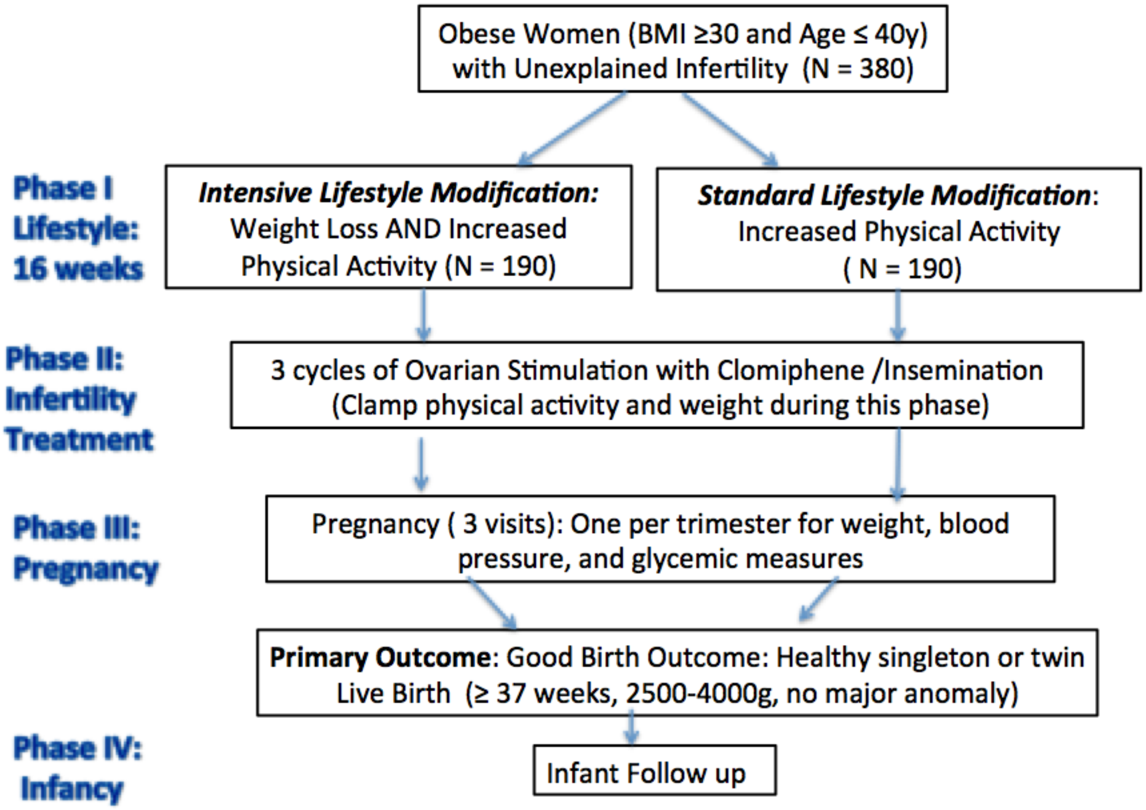

4.1 Overview

The flowchart (**Figure 3**) summarizes this study which will consist of infertility treatment commencing with randomization at equal allocation (1:1) to two interventions (Phase I), an intensive lifestyle modification which includes caloric restriction primarily with meal replacements (Nutrisystem), use of a weight loss medication (Alli), and physical activity recommendations, and a less intensive lifestyle modification intervention focused on increasing physical activity with weight maintenance. This will serve as the comparison group. After completion of the 16 week lifestyle intervention, subjects will receive 3 cycles of ovarian stimulation with an oral medication (clomiphene citrate), triggered ovulation with hCG, and a single intrauterine insemination per cycle (Phase II). During the infertility treatment phase, subjects will be encouraged to maintain their weight and activity levels achieved during the initial intervention. Subjects who conceive will be followed to the point of documented viable intrauterine pregnancy, and then will be seen once per trimester for a brief visit with serum and urine collection. All pregnancies will be followed until completion (Phase III). Subjects who deliver viable infants will be offered participation in our Pregnancy Registry (Phase IV). The details of the Pregnancy Registry protocol can be found in that protocol document.

4.1.1 Treatment Design

This will be a two-arm, multi-center, prospective randomized trial of two types of lifestyle modification in obese women with unexplained infertility who will be randomized to either an intensive lifestyle modification with weight loss and increased physical activity or a standard lifestyle modification consisting of increased physical activity for 16 weeks prior to initiating infertility treatment with an oral drug, clomiphene, to stimulate follicular development followed by intrauterine insemination (IUI). The lifestyle modifications will be open label with adequate allocation concealment and the randomization ratio will be equal between modifications (1:1).

The appropriate study candidates will be recruited from the main sites of the Reproductive Medicine Network and their ancillary partners. Recruited subjects will meet the inclusion and exclusion criteria detailed below. Monitoring of this trial at all sites will be conducted by the RMN Data Coordination Center, with progress reports provided to the RMN Data and Safety Monitoring Board (DSMB) no less than every three months in order to review trial progress and subject safety.

4.1.2 Study Population

Three hundred eighty (380) infertile obese ovulatory women actively seeking pregnancy (190 per treatment arm), age 18-40 years, will be enrolled in the participating sites. The overall goal of the inclusion/exclusion criteria is to identify a population of healthy infertile women who are regularly ovulating. These will include a normal uterine cavity with at least one patent tube and a partner with a motile sperm count of at least 5 million in the ejaculate. If existing medical records are used to verify inclusion or exclusion criteria, the site should keep a copy of these in the source documents. A general list of exclusionary medications requiring a washout period is found in the appendix. The list is not exhaustive and questionable medications can be looked up to see if they belong to one of the families of exclusionary medications, or the Project Leader (Dr. Richard Legro) and/or DCC can be queried.

5 Selection and Enrollment of Subjects

5.1 Inclusion Criteria

1. Women ≥ 18 to ≤ 40 years of age, with one or more years infertility history, desirous of conceiving, regularly ovulating (defined as 9 or more menses per year), at initiation of participation.
2. BMI ≥ 30 kg/m2 obtained at screening visit.
3. Normal uterine cavity and at least one open fallopian tube confirmed by hysterosalpingography (HSG), sonohysterography (SHG), or laparoscopy/hysteroscopy in the last three years preceding enrollment into the study. An uncomplicated intrauterine non-IVF pregnancy and uncomplicated delivery and postpartum course resulting in live birth within the last three years will also serve as sufficient evidence of a patent tube and normal uterine cavity as long as the subject did not have, during the pregnancy or subsequently, risk factors for Asherman's syndrome or tubal disease or other disorder leading to an increased suspicion for intrauterine abnormality or tubal occlusion.
4. Evidence of ovarian function/reserve as assessed by day 3 (+/-2 days) FSH ≤ 10 IU/L with Estradiol ≤ 70 pg/mL OR serum AMH ≥ 1 ng/mL within one year prior to study initiation.
5. Normal or corrected thyroid function within one year of study initiation.
6. Normal or corrected prolactin level within one year of study initiation.
7. In general good health, not taking any medications which could interfere with the study (e.g., FSH, insulin sensitizers - see Appendix for full list).
8. Ability to have inseminations following hCG administration
9. Male partner with total motile sperm in the ejaculate of at least 5 million sperm, within one year of study initiation
10. Able to have intercourse and collect semen for insemination

5.2 Exclusion Criteria

1. Currently pregnant or successful pregnancies within 12 months of initiating participation. Clinical intrauterine miscarriages prior to initiating participation, within ASRM guidelines: subjects 35 and over must wait six months, while subjects under 35 must wait 12 months. No exclusion for biochemical pregnancies.
2. Undiagnosed abnormal uterine bleeding.
3. Suspicious ovarian mass.
4. Subjects on oral contraceptives, depo-progestins, or hormonal implants (including Implanon). A two month washout period will be required prior to screening for patients on these agents. Longer washouts may be necessary for certain depot contraceptive forms or implants, especially when the implants are still in place. A one-month washout will be required for patients who have taken oral cyclic progestins.
5. Known 21-hydroxylase deficiency or other enzyme defect causing congenital adrenal hyperplasia.
6. Type I or Type II diabetes mellitus, or if receiving antidiabetic medications.
7. Known significant anemia (Hemoglobin <10 g/dL).
8. History of deep venous thrombosis, pulmonary embolus, or cerebrovascular event.
9. Known heart disease (New York Heart Association Class II or higher).

10. Known liver disease (defined as AST or ALT >2 times normal, or total bilirubin >2.5 mg/dL).
11. Known renal disease (defined as BUN >30 mg/dL or serum creatinine > 1.4 mg/dL).
12. History of, or suspected cervical carcinoma, endometrial carcinoma or breast carcinoma.
13. History of alcohol abuse (defined as >15 drinks/week) or binge drinking of ≥ 6 drinks at one time).
14. Known Cushing's disease.
15. Known or suspected adrenal or ovarian androgen secreting tumors.
16. Allergy, known hypersensitivity or contraindication to the treatment medications used in this study including orlistat (This will include patients with chronic malabsorption syndrome or cholestasis or clomiphene (previous change in vision).
17. Couples with previous sterilization procedures (e.g. vasectomy, tubal ligation) whether or not it has been reversed.
18. Subjects with untreated poorly controlled hypertension defined as a systolic blood pressure ≥ 160 mm Hg or a diastolic ≥ 100 mm Hg obtained on two measures obtained at least 60 minutes apart.
19. Subjects who have undergone a bariatric surgery procedure in the past or are in a period of acute weight loss (defined as a weight loss of greater than 5% body weight in the last 6 months).
20. Known severe endometriosis
21. Anovulation or oligo-ovulation including hypothalamic amenorrhea, polycystic ovary syndrome, etc.
22. Donated semen.
23. Couples in which either partner is legally married to someone else.
24. Medical conditions that are contraindications to pregnancy.
25. Presence of severe, untreated psychiatric illness (major depression, substance abuse, eating disorder, etc.) that would, in the opinion of the site investigator, interfere with the patient's ability to successfully complete the study.
26. Any additional medical conditions that would be a contraindication to orlistat.
27. Any contraindication to study requirements including diet recommendations and activity requirements.
28. Currently participating in a lifestyle intervention program (such as Weight Watchers, Atkins Diet, Curves)
29. History of Gout.
30. History of pelvic radiation

5.3 Study Termination Criteria

1. Development or suspicion of an allergic or serious adverse reaction to any of the medications in the study.
2. Uncontrolled hypertension.
3. Persistent ovarian cyst > 30 mm mean diameter that does not resolve in two cycles.
4. Serious or severe Ovarian Hyperstimulation Syndrome (OHSS).

5.4 Study Enrollment Procedures

5.4.1 Recruitment

Clinical Practices of Investigators

Infertile couples presenting for consultation with an obese female and unexplained infertility (after clinical confirmation of their eligibility for admission into the study based on inclusion and exclusion criteria) will be approached to participate by the research coordinator or non-care providing physician. In the recruitment visit, study details including the expectations of lifestyle modification, as well as the experimental nature of the preconception intervention will be explained. Risks and benefits will be thoroughly discussed and consents given to the patient and partner.

Hospital/Local Health Care Referrals

Subjects will be recruited at each site from individual practice(s) of the investigators as noted above as well as faculty/resident clinics. Ongoing contact with practice and faculty members as well as with residents will be made by the investigators and coordinators, reminding them of the inclusion criteria, importance of the study, etc. In addition, the investigators will describe the study to members of other departments in the hospital, primarily family practice, medical endocrinology, urology, and gynecology who also see and treat these patients. Contact with local physicians will be made and/or grand rounds will be given to disseminate information about the study.

Referrals from Study Participants

Study participants often refer friends, acquaintances and colleagues to be potential participants.

Local Publicity Office

Investigators will meet with their local Public Relations offices and plan a news release about the study. They will also make themselves available for any newspaper, radio, or TV stories that may increase public awareness of the study. The full gamut of local media sources should be utilized. Often there is greater yield with more extensive coverage in smaller local outlets as opposed to brief mentions in outlets with larger circulation. News releases will mention the uniqueness of the study.

Local Advertisements

Advertisements will be placed in local newspapers and will be continued on a regular basis if response is good.

Contact with infertility support groups

Contact will be made with both national and local support groups to spread information about the study through informational brochures and/or participation in local meetings. The American Infertility Association also may be helpful in promoting awareness of this study.

National Professional Organizations

Contact will be made with the publicity office of The American Society for Reproductive Medicine, and other potentially helpful organizations to solicit their support and potential informational releases.

Web sites

The study will be prominently displayed on the RMN web site. Additionally each RMN center should have a web page devoted to this study with general as well as contact information. Information should also be available at the NICHD web site with links to each RMN center. An ad should also be placed at "Center Watch" on the web.

National Advertising
We would consider placing a trial ad in the health section of a select or a series of selected national publications with all of our local numbers/contacts.

IRB Approval
It is expressly acknowledged that all informational material that could be construed to be advertising will be approved by the appropriate IRB prior to dissemination.

5.5 Procedures for Tracking Sources of Subjects and their Disposition

We will track all contacts from subjects interested in the study. We will develop a pre-screening list that documents date and point of contact, eligibility based on telephone screening, and follow-up if subject meets prescreening and is interested in further participation. The consent form is mailed or emailed prior to the screening visit which is the next point of contact.

5.6 Obtaining Informed Consent

Once potential couples have been prescreened, they will be referred to the site clinical coordinator or his/her designee for a screening visit The consent will be explained to potential subjects and their partners by the coordinator or possibly the physician investigator depending on the clinical circumstances.

Inclusion and exclusion criteria will be reviewed. After the study has been completely explained to the potential couple, they will be given the informed consent documents to review (sample consent forms, in the format of Penn State University, are included in Section 10). Some individuals may wish to complete the informed consent process at the time of this discussion. In these cases the informed consent documents will be signed once all questions are resolved. In other cases the subjects may wish to take the consent forms home for further consideration. In these cases the coordinator will confirm the couple's willingness to be contacted, and set up a tentative timeframe to be back in touch with the subjects. The consents can be signed either with the coordinator or with a physician once all questions have been answered to the satisfaction of the potential patient and partner. A signed informed consent document, approved by the IRB at the study site, will be confirmed on all subjects prior to the baseline evaluation. The PI at each RMN site should not simultaneously be managing the care of a patient in the study and be the primary caregiver for a study participant, as specified in the NICHD clinical Research Policy guidelines.

In order to be eligible for enrollment and randomization, each member of the couple must be confirmed to meet all inclusion and exclusion criteria described above.

5.7 Intervention Group Assignment

After screening is completed the information will be uploaded to the WebEZ system electronically, and randomization will be performed by the program at the time of the randomization visit. Subjects will be immediately informed of their treatment assignment and orientation will begin.

6 Study Interventions

6.1 Interventions, Administration and Duration

<u>Phase I – Preconception Treatment</u>

<u>Overview</u>
Phase 1 will involve randomization to either an Intensive Lifestyle Modification program or a Standard Physical Activity Program. Each will last 16 weeks. The Intensive Lifestyle Modifciation Program will consist of an over the counter anti-obesity drug, orlistat, caloric restriction assisted by the use of meal replacements, and increased physical activity. The Standard Physical activity Program will consist of increased physical activity alone. All subjects will receive folic acid supplementation throughout all phases of the study.

Our goal in designing the lifestyle modification program is to develop simple methods that are easily comprehended by our subjects and study personnel. Therefore we are dispensing with labor intensive recall questionnaires of diet and activity that are frequently utilized in such studies but have little correlation with change in hard outcomes such as weight or biochemical parameters. We will create identical on-site in-person study visits and self-monitoring schedules for both lifestyle modification groups. We will utilize in both groups wireless devices that monitor activity (Fitbit) and weight (Fitbit Aria Scale) and automatically upload data to a central website (maintained by the DCC with appropriate consultants). This will enable both self-monitoring by the subject and central monitoring by the study personnel (who will provide devices, a web account, and joint access to the website). This will allow both subjects and study personnel the ability to view integrated and time related data and will also save time for subjects and study personnel. Finally, it allows a seamless record of weight and activity from preconception to conception to pregnancy to delivery. We will develop algorithms to address poor compliance with activity recommendations in both groups based on Fitbit self-monitoring as well as lack of weight loss within the intensive lifestyle modification with caloric restriction group. We will pilot text-based automated responses on smart phones to achieve these goals, as well as using more traditional (phone or email) methods for those without access to smart phones. Both groups will have similar formal site visit schedules and interactions with study personnel.

<u>Intensive Lifestyle Modification:</u> The primary focus of this modification will be to achieve weight loss as well as to increase physical activity. We will use a multifocal approach to achieve weight loss, utilizing meal replacements as well as a gastric lipase inhibitor (orlistat OTC - brand name Alli at 60 mg per dose) to prevent fat absorption. Multifocal approaches have been shown to increase weight loss beyond single therapy based interventions.

Medication (Orlistat) will be initiated at a dose of 60 mg per meal at lunch and dinner. We will skip the morning dose, as subjects have a low fat meal replacement (MR) breakfast: *<u>MRs as basis of a hypocaloric diet (~1200-1500 kcal/d).</u>* We, led by Dr. Penny Kris-Etherton, will develop a complete daily diet plan for the 16 week intervention plan with meal replacements that includes two prepared entrees for lunch and dinner and a cereal bar or beverage. The meal replacements

will be provided without cost to the participants in this treatment group. In addition, subjects will be instructed to consume two servings of fruit, 3 servings of vegetables, and two servings of low fat dairy per day. This diet will provide about 1100 calories with this macronutrient profile: 30% calories from protein, 45% calories from carbohydrate; and 25% calories from fat. Since we want to provide 1200 calories per day, there are 100 calories that can be consumed as desired. This meal plan totals approximately 1200 kcal/d and is consistent with the lifestyle modification in the Look AHEAD study.[79] Participants will be provided full instructions on how to use MRs and will develop a daily schedule for consuming them. At the randomization visit in Phase I, participants will receive instructions on ordering MRs directly from the Nutrisystem web site which will be shipped directly to their house. They will receive a monthly code to order further meal replacements. They will also at the randomization visit receive a two month supply of medication. We will ask subjects to weigh themselves at home on a weekly basis and will capture results automatically with the Fitbit Aria scale.

Increased Physical Activity Subjects will be asked to use the Fitbit Physical Activity Monitor during the screening phase to establish a mean number of steps over a 7 day period. This will then form the baseline number of steps. This baseline average of steps will be used as the starting point for increasing average steps a day by 500 steps a day per week until the desired maximum of 10,000 steps is reached. Subjects will be asked to maintain that level of physical activity throughout the study.

6.1.1 Determine Baseline Physical Activity

Subjects will be dispensed a Fitbit Physical Activity monitor during the randomization visit which will be used to determine average daily steps over a week's time.

Standard Physical Activity Intervention: This will be identical to the physical activity described above for the intensive physical activity group. There will be no dietary recommendations, no use of meal replacements, and no use of anti-obesity drugs. Subjects will have the same monitoring schedule both on site and off site as subjects in the intensive lifestyle modification.

Conception during Lifestyle Modification We will encourage intercourse without barriers during the lifestyle modification phase and welcome any pregnancies which happen. They will be managed identically as though that occur during the empiric infertility treatment phase of this study (Phase II below). Of course such pregnancies will be analyzed by treatment group according to intention-to-treat (ITT) principles. We are using safe and widely utilized lifestyle modifications. We are using an OTC weight loss drug, Alli, that has no known early adverse pregnancy effects and we will immediately discontinue it if pregnancy occurs. The ability to conceive at all points in this study will increase the appeal to participate and also the external validity of our results. No one with infertility wants to stop attempting pregnancy as part of infertility treatment. This also led us to reject using the oral contraceptive pill as a control arm for unexplained infertility.

Phase II – Standardized infertility treatment

After completion of Phase I (or 16 weeks of pretreatment), all subjects will enter Phase II. This phase will be universally administered to all participants in an open label, but standardized fashion. In this part of the study, subjects will receive three cycles of ovarian stimulation/hCG

trigger for ovulation/ timed IUI and followed by midluteal visit. We will clamp weight and activity in both groups and continue to monitor with the Fitbit and the Aria scale. We will modify and streamline the study procedures from our AMIGOS study.

Phase III – Pregnancy

There will be no treatment intervention during pregnancy, though we will continue to monitor weight and have trimester study visits.

Phase IV – Pregnancy Registry

All subjects who deliver will be encouraged to participate in our Pregnancy Registry which currently provides infant follow up till age 3. This is currently a separate protocol and we will also obtain separate informed consent for participation in this protocol.

6.2 Handling of Study Interventions

All study interventions are FDA approved for the indication (i.e. orlistat to treat obesity), clomiphene to treat infertility or are common practices, i.e. prenatal and folic acid supplementation, meal replacements to achieve caloric restriction. They will be dispensed open label in concordance with policies at each site (i.e. some may require orlistat, though available over the counter to be dispensed from a research pharmacy). We will also use meal replacement gift certificates for purchasing meal replacements. Further details can be found in the Manual of Operations and Procedures.

6.3 Concomitant Interventions

No other interventions, other than those specified in the protocol are allowed to treat obesity or infertility during participation in the study. Exclusionary medications and conditions for randomization are also excluded during participation in the study.

6.4 Adherence Assessment

Adherence to the interventions during Phase I will be monitored by the use of the FitBit activity monitor and Aria Scale off site, and by monthly visits on site. Additionally pill counts and dietary logs will confirm adherence to the Intensive Lifestyle Modification program. We will also perform pill counts and collect the used hCG vial during the Phase II infertility treatment to confirm compliance. We will also collect intercourse and menstrual diaries during the study. We will make accommodations for those subjects who do not have regular access to the internet or a smart phone by arranging regular downloads of FITBIT data through alternate means at regular intervals (2 weeks).

7 Clinical and Laboratory Evaluations

7.1 Schedule of Evaluations

The table below (**Table 7**) summarizes the clinical and laboratory evaluations during the trial. A description of the visits follows.

Table 7: Study Visits and Procedures

Phases	Phase 1: Lifestyle Intervention (16 weeks)				Phase 2: Infertility Treatment						Phase 3: Pregnancy
Visit Titles	*Screen (can be done in 2 visits)*	*Baseline /Drug Assign*	*Month Visit 1, 2 & 3*	*End of Phase 1 (week 16)*	*Cycle 1 Baseline (day1-5 of menses)*	*Monitor Visit *(cycle day 8-12)*	*IUI*	*Urine Preg Test *****	*Serum hCG ******	*End of Phase 2*	*16, 24, 32 week Pregnancy Visit*
Informed Consent	X										
Medical Assessment	X										
Vital Signs & Biometrics	X	X	X	X	X					X	X
Complete Physical Exam	X										
Gynecological Exam and pap smear	X										
Acne/Hirsutism Assessment	X			X						X	
Transvaginal ultrasound	X				X	X					
Sonohysterogram or Hysterosalpingogram	X										
Questionnaires & QOL surveys	X			X						X	
Urine pregnancy test	X		X	X	X			X (@home)			
Serum pregnancy test									X		
Blood work for Rubella, Varicella, HIV, genetic tests and hgb electrophoresis (optional)	X										
Blood work for female hormones	X										
Blood work for safety labs, lipid panel and HgbA1C	X			X						X	
Blood work for central core	X	X	X	X	X					X	X
Blood work and urine for reserve ancillary studies (optional)	X	X	X	X	X					X	X
Blood work for Whole blood (optional)		X									
Blood work for RMN Repository (optional)		X									
Vaginal Swab	X				X						X
Rectal swab	X				X						X
Saliva sample (optional)		X		X							
Dispense meds		X	X		X		X				
Collect meds			X	X		X				X	
Order meal replacements (if applicable)		X	X								
Dispense Fitbit and Aria scale		X									
Dispense diaries		X	X		X		X				
Collect diaries			X	X		X				X	
Adverse Events & Concomitant Meds		X	X	X	X	X		X		X	X
Intrauterine Insemination							X				
MALE PROCEDURES											
Informed Consent	X										
Medical Assessment	X										
Vital signs & Biometrics	X									X	
Questionnaires & QOL surveys	X									X	
Semen analysis/collection	X						X				
Semen for RMN Repository (optional)	X										

Blood work and urine for RMN repository (optional)	X										
Saliva sample (optional)	X										

*Monitor visits will be repeated on an individual basis until hCG administration
**To be done 2 weeks after insemination at home. Repeat cycles until pregnancy occurs or treatment cycles end; may have hCG up to 3 times (cycles). If positive pregnancy, will schedule visit for serum pregnancy test and move to Phase 3 for follow-up. If negative, start new cycle.
***Will repeat hCG 2 days after + result. OB ultrasound will be scheduled 14-21 days from + result.

7.2 Timing of Evaluations

<u>Screening visit(s):</u>

Although we anticipate that the items described below could be completed in a single visit, we will allow coordinators to divide them into two visits. For example, if the subject or couple has had little to no prior evaluation, it may be time and cost effective to obtain parameters in a stepwise fashion. We anticipate that consent, biometrics, and medical history would be obtained at first visit, whereas we would distribute kits to collect saliva, stool and dispense the FitBit activity monitor at the second visit for subjects with a high probability of randomization.

1. Obtain informed, signed consent from female and male partner.
   Female and partner must specifically be made aware of potential drug complications and adverse outcomes, and partner must sign the Male Consent Form.

2. Complete medical history and counseling of the couple
   Pre-conception counseling
   Female and partner must complete a Genetic Risk Factors questionnaire and RMN Medical History questionnaire

3. Complete Questionnaires.
   - Female: Female Sexual Function Index (FSFI), SF-12, PHQ-9, FertiQoL, Epworth Sleepiness, Stop-Bang (Sleep apnea), Female Sexual Distress Scale (FSDS), Automated Self-Administered 24-hour Recall (ASA24), and Diet History Questionnaire (DHQ II)
   - Male Partner: SF-12, PHQ-9, FertiQoL, Epworth Sleepiness, Stop-Bang (Sleep apnea), International Index of Erectile Function (IIEF), ASA24, and Diet History Questionnaire (DHQ II), Sun Exposure and Behaviour Inventory (SEBI), qADAM Questionnaire, and Psychosexual Daily Questionnaire (PDQ)

4. Physical Exams of Male and Female Subject
   - Female
     - Vital signs, height, weight, hip and abdominal circumference, BMI
     - Pap smear if necessary per current ACOG time-frame guidelines
     - Standard pelvic and breast exam conducted by physician (or within past 12 months)
     - Ferriman-Gallwey hirsutism scoring, acne lesion count

   - Male Partner

- Vital signs, height, weight, hip and abdominal circumference, BMI

5. Perform radiological exams.
   Transvaginal ultrasound measuring uterine and ovarian characteristics

   Sonohysterogram or hysterosalpingogram to verify patency in at least one tube, and normal uterine cavity (or documentation within past 3 years). An uncomplicated intrauterine non-IVF pregnancy and uncomplicated delivery and postpartum course resulting in live birth within the last three years will also serve as sufficient evidence of a patent tube and normal uterine cavity as long as the subject did not have, during the pregnancy or subsequently, risk factors for Asherman's syndrome or tubal disease or other disorder leading to an increased suspicion for intrauterine abnormality or tubal occlusion.

6. Laboratory tests.
   Females:
   -Collect blood for the following tests for inclusion/exclusion (Table 8) (if not already obtained)
   -Offer optional blood test for Rubella, Varicella, and HIV. The costs for these blood tests are not included in determining the patient care budget for this protocol.
   -Offer optional recommended genetic tests and hemoglobin electrophoresis per ACOG guidelines. The costs for these blood tests are not included in determining the patient care budget for this protocol.
   -Collect central core blood specimen to be sent to a central laboratory for testing
   -At each blood draw during the study, retain a reserve blood and urine specimen (aliquotted to at least 3 cc each) for banking on site for future ancillary studies as plasma and serum, if subject has consented for optional blood and urine collection.
   -Collect vaginal microbiome swab and rectal microbiome swab.

Table 8: Female Local Laboratory Screening Blood Tests

**FEMALE LOCAL LABORATORY SCREENING BLOOD TESTS**	
**Eligibility Labs (within 1 year)**	**Safety Screen (fasting) Labs**
AMH*	Urine pregnancy test
Prolactin	Hemoglobin
TSH	Hematocrit
HgbA1c	WBC
	Platelets
	BUN
	Creatinine
	Total Bilirubin
	ALT/AST
	Total Cholesterol
	HDL-C
	Triglycerides

- In lieu of AMH to determine ovarian reserve to meet inclusion criteria, investigators can obtain or use a prior Day 3 (+/-2 day) FSH and Estradiol
- Eligibility tests in Column 1 are valid for one year prior to randomization.
- Safety Screen tests, except the lipid panel must be obtained within 60 days prior to randomization.
- Lipid Panel (cholesterol, triglycerides, HDL) results are valid for one year prior to randomization.

Males: obtain a semen specimen.

- Local lab semen analysis (or documentation within 1 year)
- Retain semen sample for future microarray analysis if RMN Biologic Repository consent is signed
- Collect urine and blood sample for RMN Biologic Repository if consent is signed
- Collect saliva sample if consented

7. Distribute Testing and Study Supplies
    a. Distribute salivary collection kit, if consented.

<u>Randomization Visit:</u>

Randomization visit can take place regardless of menstrual cycle day and as soon after screening visit as subject and couple meet inclusion/exclusion criteria. Subjects will be informed in person of their randomization assignment. This is to allow for in person counseling of the lifestyle modification and answer face to face any concerns about their treatment assignment. Subjects will undergo during this visit: extensive orientation to the goals of the lifestyle modification, dispensing of all supplies and medications, and review of tracking and self-reporting requirements.

1. Perform limited physical exam, including weight and vital signs.

2. Orient subject to study treatment group including activity recommendations and use of meal replacements and orlistat.

3. Dispense study supplies such as Aria Wifi scale and Fitbit Activity tracker.

4. Dispense weight loss medication (orlistat) and provide instructions for ordering meal replacements on Nutrisystem’s website to subjects randomized to the extensive lifestyle modification arm.
5. Dispense intercourse and menstrual diaries.

6. Prenatal vitamins with folic acid dispensed.

7. Collect central core blood specimen as well as urine and blood reserve sample for ancillary studies (to be stored on site) (with consent). This will include hormonal assays for sex steroids (E2, P4, Testosterone), other reproductive hormones (SHBG), adipokines (leptin, adiponectin) and other hormonal and metabolic parameters that are determined relevant to the study.

8. Collect blood for DNA for the pharmacogenomic studies (with consent) to be stored on site.

9. Collect all available biospecimens from subject and male partner for RMN Biologic Repository (with consent).

10. Collect saliva specimen (with consent).

<u>Monthly Visit during Lifestyle Modification Phase (Phase 1) (N = 3)</u>

1. Perform limited physical exam, including weight, waist circumference, and vital signs (respiratory rate, pulse, and blood pressure).

2. Perform urine pregnancy test.

3. Collect menstrual and intercourse diaries, dispense new for next month.

4. Collect remaining study medications and perform pill count.

5. Dispense study supplies and medications, Prenatal vitamin, if applicable, orlistat.

6. Ensure that meal replacements for the next month have been ordered through Nutrisystem.

7. Collect adverse events.

8. Collect blood and urine reserve sample for ancillary studies (to be stored on site) and central core specimens which will be run at a core lab facility.

9. During the 3rd monthly visit, dispense saliva kit for collection to be returned Phase 1 closeout, if consented.

<u>Lifestyle Modification Closeout Visit (Week 16)</u>

Subjects will undergo a repeat baseline visit at the end of their 16 week lifestyle modification phase. This will again be independent of cycle day. We will not repeat breast and pelvic exam, pap smear or other items required for inclusion in the study that were obtained at the baseline or screening visits. Intercourse and menstrual diaries will be collected. Female study participants will have a limited physical exam. We will defer transvaginal ultrasound exam until the baseline visit of the infertility treatment cycle. We will obtain questionnaires and biospecimens.

1. Perform limited physical exam, including weight, waist circumference and vital signs (as above).

2. Perform urine pregnancy test.

3. Collect menstrual and intercourse diaries.

4. Collect remaining study medications and perform pill count.

5. Collect adverse events.

6. Collect blood and urine reserve sample for ancillary studies (to be stored on site) and central core specimens which will be run at a core lab facility. Collect specimens for repeat safety screening (Table 8).

7. Collect saliva sample.

8. Repeat full screening visit including hirsutism and acne scoring, QOL questionnaires (excluding DHQII) (See above screening visit) with the exception of full physical exam and transvaginal ultrasound (which will be performed at the start of the subsequent infertility treatment baseline visit).

Baseline Ultrasound Visit for Empirical Infertility Treatment (Phase 2)

This baseline visit will be performed at the start of Cycle 1 only. The initial dose of clomiphene citrate will be 100 mg/day, which may be adjusted upwards or downward depending on response in subsequent cycles. Women may undergo a total of up to 3 cycles culminating in hCG administration. Subjects may begin subsequent cycles immediately following failed cycles, as long as they meet baseline criteria of a negative urine pregnancy test. Subjects may continue in the study after one failed cycle (i.e. either no development of follicles on study medication). If a subject experiences a second failed cycle, it is recommended to discontinue the subject from the study.

1. Subject will call with menses and come in between days 1-5 of their menstrual cycle (Preferably day 3 for baseline monitoring visit).

2. Check Urine Pregnancy test.

3. Obtain weight.

4. Perform baseline ultrasound. If subject has a cyst with a mean diameter of >30mm, do not continue the visit. Have the subject return with her next menses for a repeat baseline visit.

5. Collect vaginal microbiome and rectal microbiome swab.

6. Dispense Infertility Treatment Study Medication (see Table 9). Please note, if the female has a prior history of over-response to clomiphene citrate at the 100mg dose, the starting dose may be adjusted downward.

7. Obtain central core lab specimens and blood and urine reserve (to be stored on site) for ancillary studies (with consent).

8. Dispense menstrual and intercourse diaries.

9. Schedule Day 8-12 visit for follicular monitoring.

Table 9: Study Medication and Dosing

Protocol	interval	dose	method	start	finish	future cycles
CC	Daily	100 mg	pill	Day 3*	Day 7	Can be started at 50-150 mg/d= (1 to 3 pills)

*(+/- 2 days)

<u>Empirical treatment Cycle Visit number 2:</u>
Within 3 days after completing 5-day drug cycle (Cycle Day 8-12)

1. Transvaginal ultrasound for endometrial and follicular monitoring.
2. Adverse effects and concomitant medications query.
3. During cycle 2 & 3, medication bottles and menstrual diaries from previous cycle will be collected.

<u>Study treatment Cycle visits number 3 and beyond:</u>
Visits for subjects will be conducted on an individualized basis until hCG administration. When a decision has been made to give hCG on the next day, a follow up monitoring visit on that day is unnecessary.

1. Transvaginal ultrasound examination for endometrial and follicular monitoring.
2. Adverse effects and concomitant medications query.

<u>hCG administration day:</u>
1. 10,000 IU in 1 cc diluent delivered IM in clinic if applicable, or subjects will be instructed in the use of intramuscular hCG and a written plan for its use will be provided.
2. Transvaginal ultrasound examination for endometrial and follicular monitoring is not necessary if one has been performed within 2 days.

Criteria for hCG administration:
1. First occurrence of lead follicle reaching 20 mm (in average diameter in two dimensions), or
2. First occurrence of two lead follicles greater than 18 mm diameter (in average diameter in two dimensions), or
3. The day after the lead follicle reaching 18 mm (in average diameter in two dimensions), or
4. The day of detection of presumptive ovulation by ultrasound.

Criteria for withholding hCG:
1. If a leading follicle does not reach a mean diameter of 18 mm after 18 days of treatment, or

2. Endogenous LH surge happens [which can lead to premature luteinization], or
3. Increased risk for OHSS and/or high-order multiple gestational pregnancy exists when more than 4 growing follicles develop (mean diameter >18 mm)

**Protocol medication adjustment:*
1. If no ovulation or development of at least one follicle ≥ 18 mm in average diameter on treatment day 18, then bring subject in for a progesterone level and serum hCG. If the progesterone is less than 2.5 ng/ml and the hCG is negative, medication will be increased in a stair-step fashion to the maximum dose of 150mg. Progestin will not be used.
2. If only one follicle ≥ 18 mm in average diameter, the dosage increase for subsequent cycles will be left to physician's discretion.
3. If more than four follicles are ≥ 18 mm in average diameter, dosage will be reduced in the subsequent cycle. Dosage may also be reduced in a subsequent cycle if excessive follicular recruitment leads to cycle cancellation before follicles reach maturity
4. If the subject has an adverse reaction to the medication, the medication may be withheld for the remaining cycles.

**Cancellation criteria:* Cycles will be cancelled if significant adverse reactions develop in response to administered medications, if criteria for withholding hCG administration are encountered, or per patient's request.

<u>Insemination protocol:</u>

One insemination will be performed within 44 hours after hCG administration, and time of insemination from hCG administration will be recorded. For inseminations, each site will utilize its standard semen preparation method and catheter.

<u>Insemination day visit:</u>
1. Each site will perform its standard insemination procedure.
2. Record parameters of semen analysis before and after sperm preparation.
3. Record time of insemination.
4. During cycle 1 & 2, medication for the next cycle will be dispensed along with menstrual diaries. The subject will be instructed that this medication SHOULD NOT be started until the current cycle is completed, either pregnancy or no pregnancy has been confirmed with a pregnancy test and the subject has spoken with the site. The subject will be instructed to perform a urine pregnancy test (see below under Urine pregnancy test) two weeks after the insemination.

<u>Urine Pregnancy test:</u>

To be conducted 2 weeks after insemination at home or at start of menses.

Patient will perform a urine pregnancy test at home and call the site with the results. If the test is positive, the patient will be scheduled for a serum hCG test. If the urine pregnancy test is negative, and menses have begun, instructions will be provided to begin the next cycle. If the urine test is negative and menses has not begun, the subject should come in for a serum pregnancy test and the site should follow standard practice for a patient with no menses following IUI.

Serum hCG Visits:

To be conducted after + urine pregnancy test

1. Obtain blood sample for qHCG. If positive result, will schedule for repeat 2 days later.

Three possible outcomes:

A. No pregnancy

B. Positive test→ retest for rising level in 2 days→ no pregnancy

C. Positive test→ retest for rising level in 2 days→ positive pregnancy

A. Negative Serum or Urine Pregnancy Test/No pregnancy

Repeat cycles until pregnancy occurs or treatment cycles conclude; women may receive hCG up to 3 times (cycles). There may be anticipated or unanticipated breaks in protocol due to medical necessity, patient request or due to site holiday recess, but none of the breaks should exceed approximately 4 weeks (allowing the subject approximately 16 weeks to complete Phase 2 of the protocol).

Criteria to initiate Cycles 2 or 3 (Menstrual Cycle Day 3 +/- 2 days):

1. Negative urine pregnancy test (within 2 days)

B. Positive Pregnancy Test (no pregnancy)

Patient will return in 2 days to check for a rising level in serum βhCG; if there is not a rising level, patient will initiate Cycles 2, or 3 (see above)

C. Positive Pregnancy Test (pregnancy)

Biochemical pregnancy will be defined as serum hCG >5 units/ml (or >10 depending on local laboratory) 2 weeks after insemination followed by a rising level two days later.

- Patient will return in 2 days to check a rising level in serum βhCG; threshold level of 2,000-4000 mIU/mL will be obtained
- Schedule Pregnancy Ultrasound Visit
- Schedule End of Study Drug Visit

Pregnancy ultrasound(s):

To be done 14-21 days after a positive pregnancy test

Clinical pregnancy rate will be defined as the identification of intrauterine sac(s) with positive fetal cardiac activity in at least one sac, 2 weeks after biochemical pregnancy identification.

1. Use to determine location of pregnancy and number of implantation sites
2. Repeat in 7-14 days if no cardiac activity detected
3. Arrange obstetrical follow-up as soon as cardiac activity is observed and there are no pregnancy abnormalities requiring further monitoring.

Pregnancy Follow up:

1. Prenatal records will be requested from the treating physician.
2. Copy of each prenatal visit records to be kept until delivery
3. Hospital record of delivery outcome will be obtained from patient's labor & delivery hospital (these will include both maternal and infant hospital records).

4. Obtain a separate consent form to enter the patient into the pregnancy registry.

End of Empirical Infertility treatment study (Phase 2) Drug Visit:

This visit will mirror the end of Phase I visit with the exception that we will repeat biometric measurements and QOL surveys in the male. We will repeat most of the parameters obtained at the baseline visit:

If pregnant: perform as early in the visit as possible, skip the FertiQoL questionnaire.

If no conception within 3 cycles: perform after the pregnancy test from the third cycle of therapy is returned negative, showing the subject is not pregnant

Other: Study withdrawal

1. Physical exam.
    a. Height, weight, waist and hip circumference
    b. Vital signs: respiratory rate, pulse and blood pressure
    c. Ferriman-Gallwey hirsutism assessment, acne lesion count.
2. Administration.
    a. Subjects will return remaining study drug.
    b. Subjects will turn in journal logs; physician will review data.
    c. Query for adverse events and concomitant medications.
    d. Females will repeat QOL surveys (excluding DHQII).
        - Pregnant women will not repeat FertiQoL.

    e. Males use Aria Scale to record weight and repeat QOL surveys (excluding DHQII).
3. Laboratory tests.
    a. Repeat the same panel of serum tests including safety labs.
    b. Obtain Biospecimens for ancillary studies, if consented, and blood for central core lab.

Pregnancy Visits per trimester (Phase 3) (at 16, 24, and 32 weeks)

1. Perform limited exam including weight, waist circumference, and vital signs (as above).
2. Obtain central core blood and urine and blood reserve for ancillary studies (store on site) (with consent).
3. Obtain vaginal microbiome and rectal microbiome swab.
4. Collect adverse pregnancy events.

Phase 4: Pregnancy Registry

All subjects who deliver will be encouraged to participate in our Pregnancy Registry which currently provides infant follow up till age 3. This is currently a separate protocol and we will also obtain separate informed consent for participation in this protocol.

7.3 Special Instructions and Definitions of Evaluations

7.3.1 History and Demographics

Medical history, including infertility treatment history, and concomitant medicines will be obtained using standardized procedures and case report forms (CRFs) developed by the RMN.

7.3.2 Physical Exam

A physical exam with a standard pelvic and breast exam will be performed on all patients by a study physician. Height, weight and waist and hip circumferences will be recorded to the nearest 0.1 cm, 0.1 kg and 1 cm, respectively. Waist will be measured at the level of the umbilicus and hip circumference will be measured at the widest diameter. Participants will be weighed while dressed in light clothing, without shoes. Weight and waist circumference will be collected at each site visit. Blood pressure will be determined in the right arm in the sitting position, and a large cuff will be used as necessary. Blood pressure will be assessed at each visit. Elevated blood pressures (≥160/100) will be repeated following acclimation to the study environment. All patients aged 21 and older should have had a normal Pap smear in accordance with current ACOG guidelines[80]; otherwise, one should be performed at the screening exam. Subjects with cytological abnormalities will need to have these resolved prior to study entry.

A hirsutism assessment will be made via the modified Ferriman-Gallwey hirsutism score (**Figure 4**) by trained study personnel [81].

Figure 4: Modified Ferriman-Gallwey Hirsutism Scale

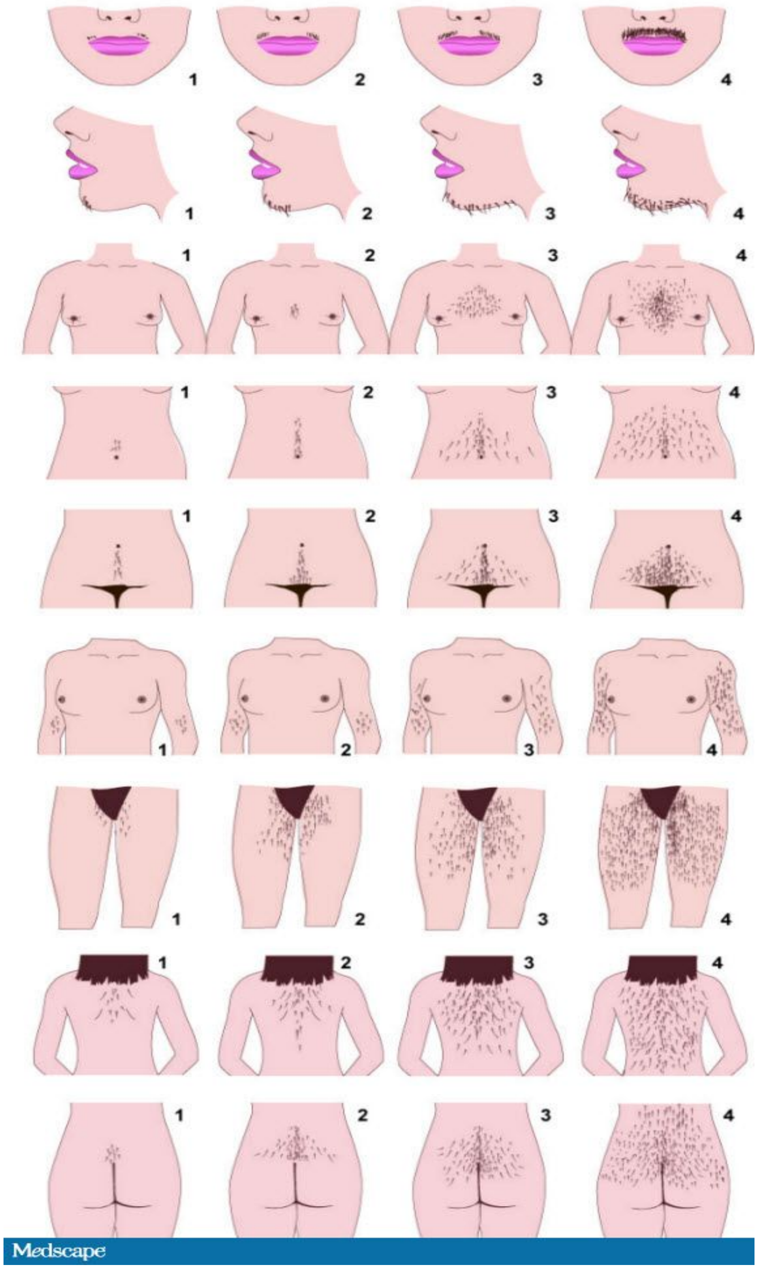

An acne assessment will be made by trained personnel using a standard acne lesion assessment (count) diagram and definitions (**Table 10**). Photographic examples of each grade will be provided to investigators as well as training to study personnel. When counting facial acne lesions, it is important that all lesions be counted, both non-inflammatory and inflammatory, examining areas of the forehead, cheeks, and chin and avoiding the nose.

Table 10: Acne Grading Method

Name	Description
Open Comedone (Blackhead)	Results when residual skin oil, makeup, dirt, dead skin, and small hairs impact a sebaceous follicle and prevent the pore from functioning correctly. If the pore is open, the comedone will be dark in appearance and is thus called a blackhead.

Closed Comedone (Whitehead)	Non-inflammatory comedone with a white center.
Papule	An inflammatory comedone that resembles a small red bump on the skin.
Pustule	An active infection of the skin that consists of dead skin cells and bacteria. These lesions are spherical in appearance and are filled with pus. Often reddish in color, pustules may be painful and will break open easily if scratched or bumped.
Nodule	A natural progression of a papule. They appear very similar to papules, but are inflamed and penetrate deep into the skin. They are often very painful.

7.3.3 Ultrasound and Imaging Exams

An ultrasound exam will be performed with a transvaginal probe. The following measures will be obtained: uterine dimensions, leiomyoma presence size and location, other uterine abnormalities (Mullerian or vascular), endometrial thickness, ovarian size in three dimensions, the size of the largest ovarian follicle, antral follicle count in each ovary (complete ovary) and ovarian morphology. Endometrial thickness is the largest anterior-posterior measurement of the endometrium in the sagittal plane. Ovarian size is determined by measuring the largest plane of the ovary in two dimensions and then turning the vaginal probe 90 degrees and obtaining a third measurement. Ovarian volume is determined by the formula for a prolate ellipsoid (length x width x height x π/6)[82]. This same ultrasound will be performed at each subsequent ultrasound visit (including follicular monitoring visits during Phase II). If the patient has had no prior test of tubal patency, this may be the desired time to perform a sonohysterogram to determine tubal patency.

7.3.4 Biospecimens

Serum: A phlebotomy will be performed at the appropriate visit. Blood will be obtained in the appropriate tubes for specimen processing and eventual aliquoting. If a subject has been fasting, this will be the first order of the study visit agenda, and subjects will be given an appropriate snack or drink after to ease hunger.

Blood for DNA: Whole blood will be collected (if consented) in an EDTA tube for eventual extraction of DNA from WBCs. This will be frozen after collection.

Urine: A urine specimen will be obtained at the visit and aliquoted into the approiate storage units.

Stool Microbiome: During a pelvic exam, a Dacron swab will be used to sample the rectum and placed in a NAT (Nucleic Acid Transport) collection tube. After collection, the samples will be refrigerated until processing. This collection method will allow for DNA analysis of the stool microbiome.

Vaginal Microbiome: During a pelvic exam with speculum in place in the vagina, Two Dacron swabs will be used to sample the posterior fornix and cervix and placed in a NAT (Nucleic Acid Transport, CentraCare Laboratory Services, St. Cloud, MN) collection tube. After collection the samples will be refrigerated until processing to preserve the DNA material from degradation. This collection method will allow for DNA analysis of the vaginal microbiome.

Salivary Analytes: A salivary collection kit will be used (SalivaBio Oral Swab (SOS)). This will be dispensed on the visit prior to the collection visit. A swab will be collected and placed in the collection tube for preservation and processing according to standard operating procedures (https://www.salimetrics.com/collection-systems). This will be collected at home at 9pm on the evening before the visit, and at 9am the day of the visit (in clinic if an am visit). The goal is to measure biomarkers for stress at the respective peak and nadir of the diurnal cycle.

Biospecimen Disposition: Specimens obtained under consent for the FIT-PLESE study will be aliquoted for storage at the study site, shipment to the RMN Central Core Laboratories, or shipment to the RMN Biologic Repository. Additionally subjects will be offered the possiblility to sign a separate consent to participate in our Registry Protocol, and will contribute additional specimens as indicated by that protocol. Further details of specimen processing can be found in the Manual of Operations and Procedures.

7.3.5 Questionnaires

Mood, quality of life, and sexual function will be assessed at baseline and at the end of the study visit. Quality of life and mood will be assessed by the PHQ-9[83] and Short Form 12 (SF-12).[84] Female sexual function will be assessed by the Female Sexual Function Inventory (FSFI) along with the Female Sexual Distress Scale (FSDS).[85] This measure is considered the "gold standard" paper and pencil assessments of sexual function and has excellent psychometric properties.[85] Male sexual response will be assessed by the International Index of Erectile Function (IIEF), a multidimensional scale for assessment of erectile dysfunction. The measure addresses the relevant domains of male sexual function (erectile function, orgasmic function, sexual desire, intercourse satisfaction, and overall satisfaction).[86] We will assess hypogonadal symptoms with the Androgen Deficiency and the Aging Male quantitative (qADAM) questionnaire and psychosexual function with the Psychosexual Daily Questionnaire (PDQ). We will assess quality of life relating to infertility and treatment with the FertiQoL survey.[87] There may also be some impact on fertility and we will investigate this in our study. We will use two simple scales to evaluate sleep, the STOP BANG questionnaire for sleep apnea,[88] and the Epworth Sleepiness Scale.[89] The Automated Self-Administered 24-hour Recall (ASA24) and Diet History Questionnaire II (DHQ II) will be used to assess past and current dietary intake. Male sunscreen exposure will be assessed by the Sun Exposure and Behaviour Inventory (SEBI v2).

8 Management of Adverse Experiences

8.1 Expected Adverse Experiences

All medications hold the possibility of an allergic reaction.

8.1.1 Phase I

There are no known side effects from meal replacements, except perhaps increased hunger. Orlistat is associated with gastrointestinal symptoms, most commonly flatulence, steatorrhea, and diarrhea. Prenatal vitamins may be associated with nausea or constipation (especially if they include iron). Increased physical activity may be associated with musculoskeletal pain. Pregnancy during Phase I is not an adverse event.

8.1.2 Phase II

The expected side effects with clomiphene include hot flashes, nausea, abdominal/pelvic pain and bloating, and mood changes. Rarely visual changes are noted. hCG given parenterally is associated with the risks of an intramuscular injection including pain at the injection site, bleeding, and infection. Intrauterine insemination is associated with the risk of pain and cramping after the procedure, spotting and less frequently bleeding and rarely pelvic infection. Both clomiphene and hCG alone and in combination are associated with an increased risk of multiple pregnancy. In the AMIGOS trial the rate of multiple pregnancy after conception was 7.6%.

8.1.3 Expected Serious Adverse Events

We do not anticipate serious adverse events during the first phase of this trial with lifestyle modification. These are more likely during the second and third phases of the trial. The expected serious adverse events are likely related to ovarian cyst formation which can result in hospitalization or surgery. Pregnancy and especially multiple pregnancy may also be a source of ectopic pregnancy, prematurity and maternal complications such as pre-eclampsia or eclampsia requiring hospital admission and induction of labor. None of the drugs or interventions are associated with increased risk of congenital anomalies.

9 Criteria for Modification or Discontinuation of Study Interventions

9.1 Phase I

There are no predetermined criteria for discontinuation during Phase I. Subjects will be allowed to continue in the trial even if they do not achieve the goals of the two interventions and eventually receive the infertility treatment in Phase II. However each subject will have to complete the 16 week pretreatment period before receving the Phase II treatments. Subjects can not be accelerated into Phase II.

9.1.1 Intensive Lifestyle Modification

Orlistat intolerance: Subjects who are unable to tolerate the dose of orlistat will be allowed to skip doses at specific mealtimes to ease symptoms or to discontinue altogether with continued intolerance based on the decision of the site PI.

Algorithm for poor responders: Subjects who fail to lose appropriate weight in the intensive lifestyle modification group or fail to meet physical activity goals in either group will be contacted by the study coordinator on a weekly basis to review reasons. We will pilot smart phone automated responses also within this trial. Drs. Kris-Etherton and Sarwer will lead the training (prior to the start of the study) and supervision (through monthly conference calls) of the interventions as well as review on a weekly basis of persistent poor responders and excessive responders (see below).

Algorithm for excessive responders: Excessive and rapid weight loss can be associated with health risks including formation of gall stones, development of hypotension, loss of energy, but rarely by an attack of gout. Subjects will be counseled at the onset about these health risks and to avoid excessive weight loss. Excessive response will be defined as a weight loss exceeding an average of 2.5 pounds per week or 10 pounds in a 4 week period. Subjects who exceed this at a monthly visit will be counseled to increase their caloric intake, by approximately 300-500 kcal/d, to slow the weight loss to our prescribed limits. We will follow subjects with their weekly weights collected by their Fitbit Aria wireless scales, and discuss them at both monthly investigator conferences and weekly coordinator conferences until they meet our guidelines. We will monitor the Fitbit steps during the first month of the study online to ensure that subjects do not exceed our guidelines of increasing 500 steps a day. We will also review monthly Fitbit records to ensure that subjects do not exceed the 10,000 steps on a regular basis. Subjects who consistently exceed our guidelines (more than 7 days in a 28 day period) will be advised to lower their activity to meet guidelines, and this will become a topic for weekly study coordinator and monthly investigator phone conferences

9.1.2 Standard Lifestyle Intervention

Algorithm for poor responders: Subjects who fail to meet physical activity goals or who lose or gain excessive weight will be contacted by the study coordinator on a weekly basis to review reasons. We will pilot smart phone automated responses also within this trial. Drs. Kris-Etherton and Sarwer will lead the training (prior to the start of the study) and supervision (through monthly conference calls) of the interventions as well as review of persistent poor responders.

Algorithm for excessive responders: We will monitor the FitBit steps during the first month of the study online to ensure that subjects do not exceed our guidelines of increasing 500 steps a

day. We will also review monthly Fitbit records to ensure that subjects do not exceed the 10,000 steps on a regular basis. Subjects who consistently exceed our guidelines (more than 7 days in a 28 day period) will be advised to lower their activity to meet guidelines, and this will become a topic for weekly study coordinator and monthly investigator phone conferences.

9.2 Phase II

Criteria for altering the clomiphene dose and withholding hCG are noted above in description of the visits. Additionally a change in visual symptoms determined by the site investigator to be caused by clomiphene administration should be considered as a reason for discontinuing further challenge with clomiphene.

9.3 Phase III and IV

There are no study interventions during this part of the study.

10 Statistical Considerations

10.1 General Design Issues

This will be a randomized open label clinical trial of two types of lifestyle modification: one intensive focusing on weight loss through caloric restriction and anti-obesity medication, and the other less intensive ("standard") focused on increasing physical activity for 16 weeks followed by three cycles of empiric ovarian stimulation combined with intrauterine insemination in obese women with unexplained infertility. We will track all pregnancies to completion. Subjects will be randomized 1:1 to the two treatments and stratified by site.

10.2 Rationale for Design

Obesity is associated with subfertility through multiple mechanisms and lifestyle modification is frequently recommended as primary treatment before initiating infertility therapy. However this common sense recommendation is tempered by data that suggest that excessive or rapid weight loss as well as excessive physical activity may be associated with lower conception rates. We propose to focus on obtaining reproductive fitness through two types of lifestyle modification prior to infertility treatment. The first will be an intensive lifestyle modification designed to improve fitness through weight loss (with a dual approach of meal replacements combined with an anti-obesity medication) AND by increasing physical activity and the second arm will be a standard lifestyle modification which will focus on increasing physical activity alone. The meal replacements will be considered a prescribed medical treatment and will be dispensed and tracked to the intensive lifestyle intervention group throughout the pre-conception intervention. Both groups will be encouraged and monitored to achieve the same amount of physical activity. We hypothesize that weight loss as achieved by the more intensive intervention will be superior to the likely weight maintenance achieved with physical activity alone. However as noted in the background there are few data beyond our preliminary data to support that hypothesis. We do not have a control arm of no treatment as currently lifestyle modification is recommended universally for obese women.

10.3 Outcomes

10.3.1 Primary Outcome Measurements

The primary outcome for this trial is the cumulative good birth outcome defined as a singleton or twin infant born at ≥ 37 weeks between 2500 and 4000g without a major congenital anomaly.

10.3.2 Secondary Outcome Measurements

We will assess secondary outcomes including live birth, time to pregnancy, pregnancy loss rate [including ectopics and Pregnancies of Unknown Location (PULs)], multiple pregnancy rate, pregnancy complication rate including development of gestational hypertension and diabetes, mode of delivery, birth weight, neonatal complication rate, predictive factors for response including DNA polymorphisms, salivary biomarkers of stress, quality of life, and cost effectiveness. Additionally we will be examining changes in the microbiome and association with outcomes.

10.4 Sample Size and Accruals

10.4.1 Sample Size and Power Calculations

Estimates used for the power calculation are based on our experience from the OWL PCOS and AMIGOS trials as provided in the preliminary data section. The primary outcome for this trial is a good birth outcome defined as an infant (singleton or twin) born at ≥ 37 weeks between 2500 and 4000g without a major congenital anomaly. We will examine the cumulative rates between groups. We anticipate the proportion of good birth outcomes to be 0.25 in the standard lifestyle intervention (physical activity and weight maintenance) arm and 0.40 in the Intensive lifestyle modification arm. We anticipate that there will be an increased proportion of good birth outcomes in each group beyond our preliminary data, as we will not actively prevent pregnancy during the lifestyle intervention; therefore, they will have an additional 16 weeks to conceive prior to initiating empiric treatment. A sample size of 190 per treatment arm will provide 80% power to detect a 0.15 absolute difference in the proportion of good perinatal births (0.25 in the standard lifestyle modification and 0.40 in the intensive group using a two-sided test having a significance level of 0.05. Although the randomization is stratified by two factors (recruitment site and BMI status), this factor was not taken into consideration for the power analysis to be conservative because accounting for the correlation of these covariates with the outcome would simply increase the precision, and hence, increase the power. The sample size has been inflated to a total of 380 participants to allow for a dropout rate of 20%. We believe this 20% dropout rate is a reasonable estimate based on current experience in our PPCOS I, PPCOS II, OWL PCOS, and AMIGOS trials. The table below summarizes other possible sample size scenarios having a 0.15 absolute difference in the proportion of good birth outcomes between the two treatment arms, all of which we would have excellent power to detect (**Table 11**).

Table 11: Sample size scenarios based on varying assumptions

Standard Lifestyle Modification: Physical Activity Arm having a Good Birth Outcome	Intensive Lifestyle Modification Weight Loss and Physical Activity Arm: Proportion having a Good Birth Outcome		Significance Level (alpha)	Power (%)	N per Arm (0% Dropout)	N per Arm (20% Dropout)
0.15	0.30		0.05	80	121	152
			0.05	90	161	202
0.20	0.35		0.05	80	138	173
			0.05	90	185	232
0.25	0.40		0.05	80	152	190
			0.05	90	203	254

10.4.2 Accrual

A total of 2.6 years will be required to complete the study after start up; 15 month enrollment period (based on 2 subjects per site/month x 13 sites), 7 month treatment period (4 months lifestyle pretreatment and 3 months ovarian stimulation/IUI), with 9 month additional observation to determine pregnancy outcomes.

10.5 Data Monitoring

There will be no pre-determined stopping rules and no interim analysis.

10.6 Study Monitoring

A monitoring plan that satisfies the ICH/GCP guidelines for clinical monitoring will be used. A Project Manager from the DCC will lead this effort, and report findings to the DCC PI. The Project Manager will have full knowledge of the study protocol, Manuals of Procedures, and is familiar with the database system and is trained to review patient charts. The Project Manager will be responsible for training and supervising other personnel.

Once personnel at participating site are trained to recruit patients, the Project Manager will be sent to the site to help initiate the study according to the study protocol, and to ensure that the clinical site meets the scientific, clinical, and regulatory requirements. For example, the Project Manager will review all signed and dated forms (such as financial disclosure forms), the curriculum vitae and certifications of the investigators and personnel, CRF training, and the written IRB approval of the protocol and consent form.

The on-site monitor will return to the clinical site after a defined number of patients are recruited (can be as early as the recruitment of the 2nd patient) or a certain time period has passed, depending on the duration of the protocol execution. The schedule of visits will be discussed and agreed in the Steering Committee and we anticipate that the Project Manager will visit each participating site at least once.

During the site visit, the clinical sites should provide to the monitor a space and access to all relevant records including medical records and regulatory binders, and there would be immediate verbal feedback provided to the site after original source documents are compared to entries in the CRF. The clinical sites must agree to cooperate with the monitor to ensure that any problems detected in the course of these monitoring visits are resolved. The on-site monitor will conduct an audit of a random sample of entered information against the source documents, a review of all regulatory documents, a review of all informed consents, and a review of all pharmacy logs. The clinical site PI and coordinator should be available to meet the monitor during the visit. The monitor will review electronic data from all sites, providing a method for identifying systematic errors or problems.

To assure Good Clinical/Laboratory Practice, the monitor will control adherence to the protocol at the clinical sites and evaluate the competence of the personnel at the clinical sites including the ability to obtain written informed consents and record data correctly. The monitor will inform the DCC PI, the Steering Committee, and NICHD regarding problems relating to facilities, technical equipment, or medical staff. A thorough written report will follow each site-visit and will include a detailed itemization of discrepancies and items requiring follow-up or reconciliation. This report will also be forwarded to the NICHD Research Scientist for review. The monitor will be responsible for maintaining regular contacts between the investigators in the clinical sites and the RMN. When the study ends, the monitor will also visit the clinical site to provide assistance for close-out.

10.7 Data and Safety Monitoring Board

The NICHD has established a DSMB to review and interpret data generated from RMN studies and to review protocols prior to their implementation. Its primary objectives are to ensure the safety of study subjects, the integrity of the research data and to provide NICHD with advice on

the ethical and safe progression of studies conducted in the RMN. The DSMB advises on research design issues, data quality and analysis, and research participant protections for each prospective and on-going study. A copy of the DSMB Charter can be found in the appendix.

The DSMB members are appointed by the Director of NICHD in accordance with established NIH and NICHD policies. DSMB members are experts in and represent the following fields: biostatistics, epidemiology, infertility, gynecology, andrology and ethics. The NICHD Committee Coordinator is responsible for scheduling regular committee meetings, recording all meeting minutes and summarizing the committee recommendations for the Steering Committee and NICHD. Steering Committee members are prohibited from attending closed sessions of the DSMB. Open sessions may be attended by Steering Committee members or Chairperson when requested by NICHD and the DSMB.

The DSMB meets regularly at a time and place of their choosing to review Network randomized trial protocols with respect to ethical and safety standards, monitors the safety of on-going clinical trials, monitors the integrity of the data with respect to original study design, and provides advice on study conduct. The DSMB periodically monitors data quality, including protocol adherence and adverse events. As outlined in the protocols, the DSMB will conduct interim evaluations of the data. It may recommend protocol modifications based on concern for subject welfare and scientific integrity.

10.8 Reporting

Administrative Reports will be prepared by the DCC, and they include monthly and quarterly reports to the RMN on accrual, data quality and study compliance and reports presented in the packet produced for each Steering Committee and DSMB. Statistical reports include reports to the SC and AB from the data analysis, and special reports for scientific manuscripts.

Statistical Reports will be generated in SAS. Reports are provided for DSMB reviews, and for final analysis of study results in preparation for scientific publications. The content of the interim reports will be very complete, and will serve as the template for the final report of each study, which in turn will form the basis of the publication of the results. Our proposed reports to the DSMB would include the following: a protocol description and history; accrual rates; site performance in terms of accrual; eligibility; protocol violations; data accuracy and minority representation; patient characteristics by treatment and site; and the rate of adverse experiences.

10.9 Data Analysis

Primary analyses will use an intent-to-treat paradigm, wherein all randomized subjects are included according to their randomized treatment assignment, regardless of actual treatment received, protocol violations, etc. for the primary outcome. The data will be summarized using descriptive statistics for continuous variables (mean, standard deviation, number of observations, and percentiles) and frequency statistics (frequencies and percentages) for categorical variables. All hypothesis tests will be two-sided and all analyses will be performed using SAS software, (SAS Institute, Inc., Cary, NC) or R (open source).

Primary efficacy analysis will be done using a logistic regression model to compare the two treatment arms with respect to the primary outcome of a good birth outcome while adjusting for recruitment site and baseline BMI. The proportion of good birth outcomes in each treatment

arm will be reported, as well as the odds ratio (OR) and corresponding 95% confidence interval (CI). We propose a number of secondary analyses and acknowledge that the power of our study may be low for some of these outcomes. However, even if not significant, examination of these secondary endpoints will guide future research in the area of infertility treatment. Logistic regression models will be fit to binary outcomes, such as pregnancy status (yes/no), to assess differences in the treatment arms while adjusting for recruitment site and baseline BMI. For rare binary events, e.g., multiple pregnancy births or other adverse events, Firth's penalized likelihood approach to logistic regression will be used to address the issue of having a small number of events.[90] Effect sizes from the logistic regression models will be quantified using the OR and 95% CI.

The analysis of other secondary outcomes will entail the application of statistical methods that have been developed for correlated data since repeated observations will be made over time on each individual, and these methods allow for both within-group and between-group comparisons to be assessed. For secondary outcomes such as weight loss or ultrasound parameters, a linear mixed-effects model will be fit where the main independent variables will be treatment arm, cycle number, and their interaction, as well as the randomization stratification factors of recruitment site and baseline BMI as covariates.[91] The linear mixed-effects model is an extension of linear regression that accounts for the within-subject variability inherent in longitudinal trials and has the flexibility to adjust for potentially confounding covariates if deemed necessary. Potential additional covariates in the models may include prior exposure to study medication, the baseline value of the outcome, and age. The effect size will be quantified from the mixed-effects models using the difference in the means between the treatment arms and corresponding 95% CI. For binary outcomes measured over time, e.g., ovulation rates, generalized estimating equations with a logit link, an extension of logistic regression that accounts for correlated data, will be fit where the main independent variables will be treatment arm, cycle number, and their interaction, as well as the randomization stratification factors of recruitment center and baseline BMI as covariates.[92] Cox proportional hazards models and the Kaplan-Meier method will be applied to compare time to pregnancy and time to live birth in the treatment groups.

<u>Participant Drop-out and Other Missing Data</u>: While every attempt will be made to collect complete data and prevent drop-out, missing data are inevitable. The sample size has been inflated by 20% in anticipation of some drop-out such as participant retention issues (based on experience in the PPCOS I, PPCOS II, OWL PCOS, and AMIGOS studies). The mechanism for missing data (missing completely at random, missing at random, or missing not at random) will be examined prior to beginning the analyses. We will compare the available characteristics of those with missing data to those with complete data and attempt to predict missingness with available data using multiple logistic regression and calculating the area under the receiver operating characteristic curve.[93] If necessary, imputation techniques may be used,[94] and shared parameter models or random pattern-mixture models considered if the preliminary analyses suggest that the data are missing not at random so that individuals with missing data can be included in the analyses.

<u>Statistical versus Clinical Significance and Multiple Testing</u>*:* As our analyses are based upon defined hypotheses, we have not included corrections for multiple testing. While the issue of p-value adjustment for multiple testing has long been a topic of discussion in observational

epidemiology and clinical trials, it generally is accepted that p-values should not be the sole criterion for assessing relationships. Conclusions will be based on the preponderance of scientific evidence related to each hypothesis, considering point estimates and confidence intervals, as well as statistical significance. Nonetheless, findings with marginal statistical significance will be interpreted cautiously, taking into consideration whether multiple testing could have contributed to a type I error.

11 Data Collection, Site Monitoring and Adverse Experience Reporting

11.1 Records to be kept

Data will be collected prospectively by designated research personnel at each study site, supervised by the site PI. Subject data will be entered into a web-based data management system created by the Data Coordination Center, using only a study ID number. Original source documents will be kept in the study subject folder.

11.1.1 Maintenance/Retention of site records

In order to comply with Good Clinical Practice (GCP) requirements, the investigators must maintain the master patient log that identifies all patients entered into the study for a period of two years after the study ends so that the subjects can be identified by audit. The PI must maintain adequate records pertaining to subjects' files and other source data for a minimum of 5 years after completion of the study.

11.2 Role of Data Management

Each clinical site and the DCC will be responsible for ensuring study personnel are trained and follow the data management guidelines of Good Clinical Practice and RMN policies.

11.3 Quality Assurance

The DCC will perform regular clinical site monitoring to assure protocol compliance, ethical standards, regulatory compliance and dat quality at the clinical sites, including review or records available for inspection by monitoring authorities. These data will be shared with the Steering committee and the DSMB as needed. Regular face to face meetings, monthly conference calls, and phone conferences of the protocol committees and recruitment committees will be forums for addressing quality assurance issues.

11.3.1 Data Entry and Forms

Case Report Forms (CRFs) will be developed as the protocol is developed. They will also be implemented in a Web-based Oracle data management system. The Web data entry forms will be similar to the paper forms with the same questions. However, the Web forms usually have more flexibility than the paper forms, such as pull-down menus.

11.3.2 Features of Data Management System

Features of the data management system include study definition; different types of data entry (and complete audit trail); forms control; query capture, reporting, and resolution; dictionary coding of Adverse Events (AEs) and medical terms; and clinical data review Tools. The end-user/reporting/ad hoc query front-end uses a standard Web browser, so that data entry and browsing can be done from any machine with Internet access, without purchase of special software. Login to this system will be through a secured Web server with the security under the protection of Yale Center for Clinical Investigations.

11.3.3 Data Security

A data server and Web server will be used. The data server will be managed by YNHH IT center and the servers will be managed by Forte Research Systems. The web server will be accessible through a secured login, but the data server can only be accessed through the web server. For security purposes, no login to the data server will be permitted, and access to the back end is

limited to authorized individuals. PHI, including patient names and addresses, will be locked and secured at the participating sites, and data will be linked through a unique identification number, which will be assigned after a patient is screened or enrolled. Access will be limited to authorized individuals (21 CFR 11.10(d)). Each user of the system will have an individual account. The user will log into the account at the beginning of a data entry session, input information (include changes) on the electronic record, and log out at the completion of the data entry session. The system will be designed to limit the number of log-in attempts and record unauthorized access log-in attempts. Individuals will work only under their own access key, and not share these with others. The system will not allow an individual to log onto the system to provide another person access to the system. Users will be asked to change their passwords at established intervals commensurate with a documented risk assessment. This plan has been adapted from the guidelines for computerized systems used in clinical investigations established by the U.S Department of Health and Human Services Food and Drug Administration.

11.3.4 Data Quality Control

11.3.4.1 Competency to perform procedure/tests in the protocol

The site PI will be responsible for ensuring that study related tests are performed by competent personnel. The criteria for determination of competency may vary between sites in the study. Attempts will be made to standardize protocols whenever possible to minimize inter-site variation.

11.3.4.2 Quality Control Steps

Quality control of data will be handled on three different levels. The first level is the real-time logical and range checking built into the web-based data entry system. The research coordinators and data entry clerks at the participating sites are required to ensure the data accuracy as the first defense. The second is the remote data monitoring and validation that is the primary responsibility of the data manager and programmer at the DCC. The data manager will conduct monthly comprehensive data checks (SAS programs run on a regular basis as a systematic search for common errors and omissions), as well as regular manual checks (within the database system). Manual checks will identify more complicated and less common errors. The data manager will query sites until each irregularity is resolved. The third level of quality control will be the site visits, where data in our database will be compared against source documents. Identified errors will be resolved between our center and clinical sites. The visits will assure data quality and patient protection.

An audit trail will be added as another security measure. This will ensure that only authorized additions, deletions, or alterations of information in the electronic record have occurred and allows a means to reconstruct significant details about study conduct and source data collection necessary to verify the quality and integrity of data. Computer generated, time-stamped audit trails will be implemented for tracking changes to electronic source documentation.

Controls will be established to ensure that the system's date and time are correct. This is a multi-center clinical trial and will be located in different time zones. System documentation will explain time zone references as well as zone acronyms. Dates and times will include the year, month, day, hour, and minute to the date provided by international standard-setting agencies (e.g. US National Institute of Standards and Technology). The ability to change the date or time

will be limited to authorized personnel, and such personnel will be notified if a system date or time discrepancy is detected.

In addition to internal safeguards built into the computerized system, external safeguards will be implemented. Data will be stored at the Data Coordination Center. Records will be regularly backed up, and record logs maintained to prevent a catastrophic loss and ensure the quality and integrity of the data. This plan has been adapted from the guidelines for computerized systems used in clinical investigations established by the U.S Department of Health and Human Services Food and Drug Administration.

11.3.5 Obligation of the Investigator

11.3.5.1 IRB Review

The site PI is responsible for submitting the approved protocol and consent form to the local IRB for review. The IRB must approve all aspects of the study as detailed in the protocol, including the patient informed consent form. It is anticipated that there will be minor site-specific changes in the consent form. The IRB must periodically review the status of the study at appropriate intervals not exceeding one year. The site PI will also be responsible for submitting revisions to the protocol to the IRB, as directed by the DCC, and promptly communicating serious adverse events that result during the study, to both the local IRB and the DCC. After the approval, the informed consent and IRB approval (or amendment) letters must be forwarded to the DCC.

11.3.6 Establishment of Pregnancy Registry

We intend to establish a pregnancy registry for this trial to establish outcomes of pregnancy. Couples who conceive will be offered participation in the Pregnancy Registry and a separate informed consent form will be obtained if they choose to participate. We will track the outcomes of all randomized subjects who have a positive serum pregnancy screen during the course of this study. We will record biochemical pregnancies (defined as positive serum pregnancy screens without ultrasonically detected pregnancies), ectopic pregnancies, and all intrauterine pregnancy losses both before and after 20 weeks including missed abortions, spontaneous abortions, elective abortions, fetal demises, and stillbirths. We will review pregnancy and birth records of the mother and of the fetus to establish neonatal morbidity and mortality and the presence of fetal anomalies. This will be done for all subjects who conceive.

If they participate in the Pregnancy Registry, we will extract from these records concomitant medical and obstetrical conditions, exposure information on all other medical products used, including prescription products, over-the-counter (OTC) products, dietary supplements, vaccines, and insertable or implantable medical devices. We will file individual case reports for all congenital anomalies which will be considered a serious adverse event. Additionally we will provide to the FDA a written annual status report of the pregnancy registry as specified in the guidelines above. We intend at a minimum to perform an annual parent directed screening questionnaire to assess the infant's developmental milestones for the first three years after birth as recommended by the FDA and to review the child's CDC growth curve and medical records. The registry will follow children until age 3. A survey will be administered to the mother annually that will screen for cognitive, and neurodevelopment up to age 3. If medical issues arise from the survey, a medical record review will be performed for more extensive assessment.

11.3.7 Regulatory Requirements

The DCC will work with NICHD staff by providing them with clinical study data, reports, and other support as required for AE Reporting. The Project Managers will work with NICHD colleagues in meeting all regulatory requirements including compliance with ICH and HIPAA requirements, FDA code for federal regulations (Title 21). For example, the DCC Project Managers have registered this clinical trial with ClinicalTrials.gov via a web based data entry system called the Protocol Registration System (PRS) (NCT02432209).

11.3.8 Protocol Amendments

Once the protocol is approved by the Steering Committee, it is then reviewed by an Advisory Board and Data and Safety Monitoring Board (DSMB). After all approvals, the DCC will finalize the protocol document that serves as the agreement among all members of the Network. In the meantime, because the DCC administers all patient care costs for the RMN, the DCC will promptly issue subcontracts to the participating sites based on the cost agreements made by the Steering Committee and NICHD.

After the protocols are approved by the RMN and the Steering Committee decides that changes are necessary for scientific or clinical reasons, the DCC will facilitate the procedure in a timely and diligent fashion. The RMN investigators and key personnel will participate in teleconferences and meetings, discuss, vote, and document circumstance and rationales for the changes and the implementation procedure for the changes. These include revising study hypotheses, designs, sample sizes, data entry forms, and appropriate statistical analyses. Once the amendments are finalized and agreed to by the RMN, they will be submitted for IRB and DSMB reviews and approvals.

11.4 Adverse Event Reporting

11.4.1 Serious Adverse Events

All serious adverse events (SAEs) that occur from randomization at Phase I through thirty days after the last dose of study medication (orlistat, clomiphene, or hCG) must be reported or if the patient is pregnant, 6 weeks following delivery. A serious adverse event is defined as: fatal or immediately life-threatening; severely or permanently disabling; requiring or prolonging inpatient hospitalization; overdose (intentional or accidental); congenital anomaly; pregnancy loss after 20 weeks gestation; neonatal death up to 6 weeks after delivery; or, any event adversely affecting the study's risk/benefit ratio. Additionally, any event that, based on appropriate medical judgment, may jeopardize the subject's health and may require medical or surgical intervention to prevent one of the outcomes listed above is considered an SAE.

If an SAE occurs and is thought to be related to the study medication, the study medication will be discontinued.

The site PI will report the SAE by completing and signing the Serious Adverse Event Report Form [available in the "Study Forms" section of the RMN members-only website], and then emailing the document in PDF format to rmn.dcc@mailman.yale.edu. Subjects will be identified by study number only. No other identifying information will be included on the form. The site PI must

determine and record on the SAE form whether the SAE is unanticipated or anticipated, and if it is related, possibly related, or unrelated to participation in the research.

DCC staff will enter the SAE information in the central database. The Safety Surveillance team, consisting of the DCC, NICHD research scientist and lead PI of the protocol, will analyze the SAE to determine if it meets the criteria listed in the OHRP 45CFR46 and/or FDA 21CFR312.32 & 3.14.80.

These determinations will dictate timeframes for sites' submission to the DCC, and the DCC's submission to the DSMB (**Table 12**):

Table 12: Types of Serious Adverse Events and their reporting requirements

TYPE	SITE	DCC
Unanticipated and related/possibly related SAE, fatal or life-threatening	Report to DCC within 1 business day of discovery	Notify DSMB by end of next business day of receiving site report
Other unanticipated and related/possibly related SAE	Report to DCC within 1 business day of discovery	Notify DSMB 5 business days of receiving site report
Anticipated and related/possibly related SAE	Report to DCC within 5 business days of discovery	Notify DSMB 5 business days of receiving site report
Unrelated SAE (anticipated or unanticipated	Report to DCC within 10 business days (no more than 3 weeks) of discovery	Notify DSMB within 10 business days (no more than 3 weeks) of receiving site report

Upon receiving notification of an SAE, the DSMB will review it via a closed-session email or conference-call discussion arranged by the NICHD RMN Committee Coordinator [RMN CC] (**Figure 5**). The DSMB will send a report to the RMN CC within two weeks; reports for life-threatening SAEs will be submitted in one week. The DSMB report will include: statement indicating what related information the DSMB reviewed; the review date; the DSMB's assessment of the information reviewed; and the DSMB's recommendation, if any, for the DCC. The RMN CC will then forward the DSMB report to the DCC for the record and appropriate disbursal. The DCC will forward reportable events to all RMN investigators, NIHCD, and the FDA on behalf of the NIHCD if the protocol is under IND. The NIHCD Project Scientist will review, sign, and return the IND safety report to the DCC within 2 business days, and will follow up with the site PI and DCC on the SAE until it is resolved. The Protocol PI will evaluate the frequency and severity of the SAEs and determine if modifications to the protocol and consent form are required. Site PIs will report the SAE to their site IRB according to local IRB requirements. For more information, please see the RMN/DSMB Communication Procedure.

Adverse events deemed non-serious will also be recorded throughout study participation from the start of study drug through one week after the last dose of study medication, and reported to the DCC. If an anticipated serious adverse event occurs at a frequency greater than expected, the DCC will notify the DSMB by the end of the next business day of discovery and follow the procedures for reporting serious and unanticipated and related adverse events. The DCC will forward relevant safety information to the DSMB. If an adverse event not initially determined to be reportable to the FDA under 21CFR312.32 is so reportable, the DCC will report the adverse event to the FDA within 15 calendar days after the determination is made.

Figure 5: Serious Adverse Event Flow Chart

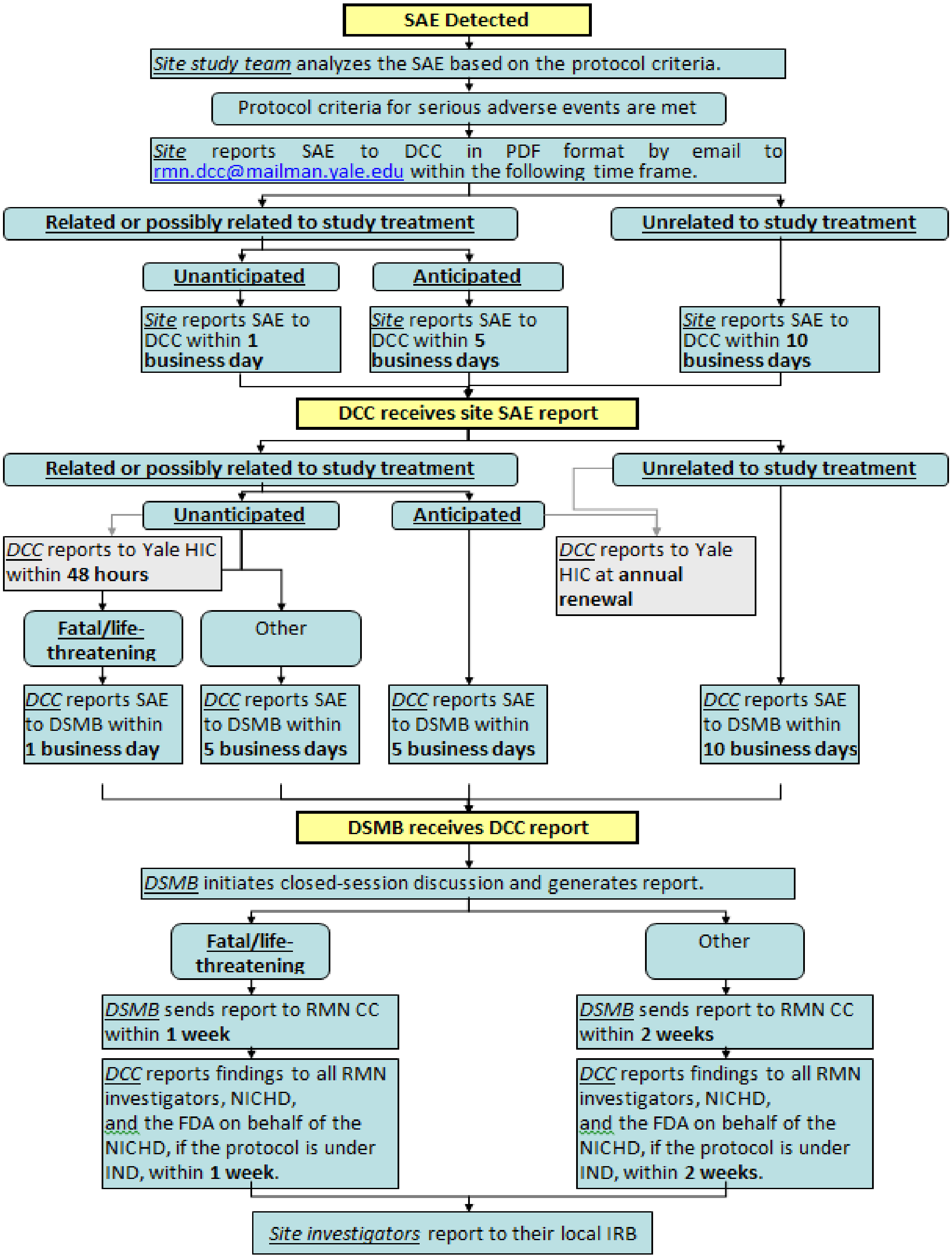

CFR= Code of Federal Regulations

12 Human Subjects

12.1 Institutional Review Board (IRB) Review and Informed Consent

This protocol and the informed consent document (see Appendix) and any subsequent modifications will be reviewed and approved by the IRB responsible for oversight of the study. A signed consent form will be obtained from the subject. The consent form will describe the purpose of the study, the procedures to be followed, and the risks and benefits of participation. A copy of the consent form will be given to the subject, parent, or legal guardian, and this fact will be documented in the subject's record.

12.2 Subject Confidentiality

All laboratory specimens, evaluation forms, reports, video recordings, and other records that leave the site will be identified only by the Study Identification Number (SID) to maintain subject confidentiality. All records will be kept in a locked file cabinet. All computer entry and networking programs will be done using SIDs only. Clinical information will not be released without written permission of the subject, except as necessary for monitoring by IRB, the NINDS, the OHRP, the sponsor, or the sponsor's designee.

12.3 Study Modification/Discontinuation

The study may be modified or discontinued at any time by the IRB, the NICHD, the OHRP, or other government agencies as part of their duties to ensure that research subjects are protected.

13 Publication of Research Findings

13.1 Overall Policy

The publications policy proposes guidelines for publications that originate from our collaborative Reproductive Medicine Network. Decisions concerning publications shall be determined by consensus (majority vote) of the collaborating principal investigators (or designate) noted below as the "Network". This policy is designed to promote prompt, exact, quality publications and presentations of Network studies with appropriate academic recognition of those with significant contributions. Protocols are classified into three types: 'Main Study' (which may include major and minor publications), 'Ancillary Study', and 'Pilot Study'. Additionally there may be publications from concepts or ideas generated by the RMN ("Related Publications") or from other groups utilizing RMN data and/or specimens "Outside Studies" (those utilizing data and/or specimens from the RMN studies). Abstract submissions to national meetings will also follow the publications policy below. The progress of publications (including presentations) will be a standing agenda note on all phone conferences and meetings. The Steering Committee will make the final disposition regarding disputes with respect to analysis request approval, prioritization, presentation, authorship and/or manuscript submission.

13.2 Main Study

A Main Study is a Network study designed prospectively by an investigator independent of other studies. Generally that investigator becomes Lead investigator of the protocol and Chair of the Protocol Subcommittee. At the end of each Main Study, a primary analysis resulting in the primary manuscript and a number of secondary analyses is produced based on the research questions stated in the protocol. The Protocol Subcommittee Chair is the primary author of the primary analysis. A main study can generate major (related to the major hypotheses) and minor publications (relating to secondary hypotheses).

13.2.1 Major Publications (Tables 13-14)

A major publication is defined as one reporting results of the major hypotheses tested. (For example, does hMG/IUI increase cyclic fecundity in couples with unexplained or male factor infertility?).

<u>*1. Authorship:*</u> Publications will include the names of investigators from each RMU and the DCC rather than merely identify the "Reproductive Medicine Network". Each RMU and DCC will have up to two authors per publication, ordinarily the PI and the Co-PI, but this may at times involve another investigator who has contributed to the study at their site, in lieu of the PI or Co-PI. The principal investigator at each RMU will be responsible for submitting the names of the two authors from that unit and designating them as either the primary and secondary authors of the unit. No more than 2 authors may represent a RMN site. An ancillary site (such as a SCCPIR) may only have 1 investigator. The Steering Committee Chair and NIH Project Scientist will be authors. Occasionally, additional authors, both within and outside the RMN may be appropriate. In these cases, the final decision will be by Network consensus (majority vote of the steering committee required).

<u>*2. First Author:*</u> The lead investigator initiating the protocol, chairing the Protocol Subcommittee will be the first author. The first author would always be expected to prepare the initial draft of the manuscript, after receiving approval from the Network to proceed. The author will prepare

the first draft of the manuscript in a timely fashion after receiving all the relevant data analyses from the DCC. The primary author will circulate the final draft to all authors prior to submission, with a timely turnaround of comments from other authors expected. Final decision of the manuscript content will be determined by the Protocol Subcommittee. In the event that the initiating protocol investigator declines first authorship or fails to meet the timeline determined by the Steering Committee (as determined by majority vote) and monitored monthly, the next RMU investigator in the rank order of authors (described below) will be the first author.

3. Authorship Order: All authorships are expected to meet reasonable criteria as set forth by the International Committee of Medical Journal Editors. Uniform requirements for manuscripts submitted to biomedical journals. http://www.icmje.org. Updated February 2006. Accessed April 4, 2007. The overall authorship order will be 1) the primary author, 2) RMU investigators, additional outside investigators with a limit of one author per site (e.g. SCCPIR investigators if applicable), followed by the Steering Committee Chair, NIH Project Scientist, and then the authors of the DCC.

Table 13: RMN Publication Authorship

Authorship Order Category	Description
1	Lead Investigator of the Protocol (N =1)
2	Primary RMN Investigators of the Protocol (N = 6); DCC investigator (N = 1)
3	Outside Investigators (i.e. Primary Investigator of SCCPIR sites) (N to be determined)
4	Additional Investigators (by Steering Committee vote) (N to be determined)
5	Secondary RMN Investigators (N = 7)
6	Steering Committee Chair (N = 1)
7	NIH Project Scientist (N = 1)
8	DCC PI (N = 1)
9	"for the NICHD Reproductive Medicine Network"

It is anticipated there will be up to 18-25 authors per major manuscript. The authorship order of the RMUs and outside sites will be based upon subject recruitment, data accuracy and promptness of data report according to the chart below:

Table 14: Authorship Order of the RMUs and Outside Sites

Investigative Sites	# Subjects Rank	Accuracy Rank	Total Rank	Authorship Order
A	1	4	5	3
B	2	7	9	6
C	3	1	4	2
D	4	2	6	4

E	5	3	8	5
F	6	5	11	7

Data accuracy will be ranked according to the rate of missing or false data entries/randomized subject at each site. Inquires that show data was accurately entered will not count against this rate of data inaccuracy. Each site's PI will be responsible to document the contributions to the study of that site's authors. In the event the journal editor requires fewer authors even after written documentation of the authors' contribution has been provided, the steering committee will vote on the authorship order which will include at a minimum the Lead Investigator and PI of the DCC (or his/her designate) in the positions listed above with the authorship order ending with the footnoted statement "for the Reproductive Medicine Network". The other authors will be referenced in the footnote and listed in the title page.

4. Acknowledgement Section: The acknowledgement section will include other investigators and study personnel who contributed substantially to the study by site, as well as members of the Advisory Board and the Data and Safety Monitoring Board. The designation will list the initials of the individual followed by their highest degree (e.g. C. L. Gnatuk, J.A. Ober, R.N., etc.) Significant contributions include but are not limited to protocol review, initiation and participation at each site, subject recruitment and enrollment, study conduct, data analysis, and preparation of the manuscript.

13.2.2 Minor Publications

Minor studies are defined as those in which the hypotheses would not be the main elements of Network studies, but in which the study data base would be utilized to test secondary hypotheses. (One example would be testing whether metformin use spares the dose of clomiphene resulting in lower dose needs.) Ideas for "minor studies" will, in general, be proposed by a single individual, who would direct all efforts leading to publication and representation. The results from minor studies would be handled similarly to those from major studies. The "Protocol" is defined as the Concept Protocol/study design of the hypothesis resulting in the publication.

Authorship will follow the Major publications guidelines above with the exception that the individual leading the minor study would be the first author, followed by the ranked primary RMN investigators involved in developing the Concept Protocol. The Lead Investigator of the minor publication can propose additional investigators who contributed to the study, whose inclusion in the authorship will be voted on by the Steering Committee (majority vote of SC required for inclusion in authorship. Centers may wish to withdraw inclusion from authorship of publications of minor studies in which only data are contributed, and this will be the decision of the individual site (RMU) PI.

13.2.3 Ancillary Study

An Ancillary Study is an observational study, conducted as a supplement to a Main Study. By definition, an Ancillary Study involves all or a subset of patients enrolled in a Main Study. An Ancillary Study does not involve any additional participants. To be defined as an Ancillary Study, there must be a need for collection of additional data not already collected in the Main Study. An Ancillary Study may also be designed by another Network investigator, who would serve as

the lead investigator and primary author of the paper. Ancillary Studies may be a “single-center” or “multi-center”.

A “single-center” Ancillary Study is a study in which all data are collected, stored and analyzed at a single center. The center bears the additional cost of such a study. The study requires approval of the Main Study Protocol Subcommittee and the Steering Committee. The center conducting the study is responsible for the analysis and reporting of the results. Abstracts and manuscripts resulting from data from the single-center Ancillary Study are not subject to the RMN Publications Policies.

A “multi-center” Ancillary Study is defined as one for which data or material (such as specimens) are collected at more than one center, or additional funds for conduct of the study are provided by the NICHD RMN and the DCC provides data analysis. Multi-center Ancillary Studies require the approval of the Main Study Protocol Subcommittee and the Steering Committee.

Authorship will be as per Major publications above with the exception that the individual leading the ancillary study and writing the paper would be the first author, followed by ranked RMN primary investigators, etc. A center not participating in the ancillary study would not receive authorship unless by majority vote of the steering committee.

13.2.4 Pilot Study

A Pilot Study is a preliminary study that generates data to help in the design of a Main Study and is the responsibility of the Main Study Protocol Subcommittee. The DCC collaborates with the Protocol Committee to complete the analysis, which may or may not generate an abstract for presentation and/or a manuscript for publication. The DCC writes a Final Report if there is sufficient data to justify one. It is not expected there will be any secondary analyses resulting from a Pilot Study.

13.2.5 Related Publications

A related publication is one that has had significant input from members of the RMN Steering Committee at formal meetings in terms of study significance and design. It is distinct from an ancillary publication in that a related publication reports on a study, concept or new methodology that has not been subjected to formal DSMB review and approval. Generally, “Related Publications” will arise from ideas and studies discussed with the Steering Committee, but not voted upon to become formal protocols.

The investigator who initiates, conducts and writes the study and those who (s)he names will be the sole authors. The authors should acknowledge the contribution of the NICHD Reproductive Medicine Network in the author line of the publication according to the format of the journal.

13.2.6 Outside Studies

Outside studies will result from the sharing of data and/or specimens with investigators whose protocols have been approved by the steering committee, and who comply with all components of those policies. All publications will acknowledge the assistance of NICHD, the RMN, and the Protocol Subcommittee in making the database available on behalf of the project. In addition, however, a disclaimer will need to be included stating, “the contents of this report represent the views of the authors and do not represent the views of the NICHD Reproductive Medicine Network.” The authors will be requested to cc the submitted manuscript to the NICHD program official to ensure compliance. These policies apply to both Network centers and outside centers.

13.2.7 Presentations

Network data should be presented before national organizations by the lead investigators of Main Studies, Ancillary, and Pilot studies. Organizations that might be appropriate include the American Society of Reproductive Medicine, the Society for Gynecologic Investigation, the American College of Obstetricians and Gynecologists, the American Urology Society and other urology or andrology societies. All presentations will be approved by the P & P committee. Once data are published in at least abstract form, all members of the Network can cite them publicly in lectures.

However, investigators should avoid citing specific numbers in review articles and chapters, for this could jeopardize peer review publication. Authorship, First Author, and Author Order are as described for Major Publications, and if there is an authorship limit to the abstract we will follow the plan above under Major Publications. Oral and poster presentations, including those resulting from secondary analyses at professional societies, must list all authors and participating institutions. In addition, they must include both the NICHD RMN logo and NIH Department of Health and Human Services logos that can be found on the Network web site.

14 References

1. Sim KA, Partridge SR, Sainsbury A. Does weight loss in overweight or obese women improve fertility treatment outcomes? A systematic review. Obesity reviews : an official journal of the International Association for the Study of Obesity 2014;15:839-50.
2. Bolumar F, Olsen J, Rebagliato M, Saez-Lloret I, Bisanti L. Body mass index and delayed conception: a european multicenter study on infertility and subfecundity. American journal of epidemiology 2000 Jun 1;151:1072-9.
3. Pasquali R, Pelusi C, Genghini S, Cacciari M, Gambineri A. Obesity and reproductive disorders in women. Human reproduction update 2003;9:359-72.
4. Imani B, Eijkemans MJ, te Velde ER, Habbema JD, Fauser BC. A nomogram to predict the probability of live birth after clomiphene citrate induction of ovulation in normogonadotropic oligoamenorrheic infertility. Fertility & Sterility 2002 January;77:91-7.
5. Imani B, Eijkemans MJ, Faessen GH, Bouchard P, Giudice LC, Fauser BC. Prediction of the individual follicle-stimulating hormone threshold for gonadotropin induction of ovulation in normogonadotropic anovulatory infertility: an approach to increase safety and efficiency. Fertility & Sterility 2002 January;77:83-90.
6. Rausch ME, Legro RS, Barnhart HX, et al. Predictors of pregnancy in women with polycystic ovary syndrome. The Journal of clinical endocrinology and metabolism 2009;94:3458-66.
7. Luke B, Brown MB, Stern JE, Missmer SA, Fujimoto VY, Leach R. Female obesity adversely affects assisted reproductive technology (ART) pregnancy and live birth rates. Human Reproduction 2011;26:245-52.
8. Winter E, Wang J, Davies MJ, Norman R. Early pregnancy loss following assisted reproductive technology treatment. Hum Reprod 2002;17:3220-3.
9. Metwally M, Ong KJ, Ledger WL, Li TC. Does high body mass index increase the risk of miscarriage after spontaneous and assisted conception? A meta-analysis of the evidence. Fertility and sterility 2008;90:714-26.
10. Wang JX, Davies MJ, Norman RJ. Obesity increases the risk of spontaneous abortion during infertility treatment. Obesity research 2002;10:551-4.
11. Garbaciak JA, Jr., Richter M, Miller S, Barton JJ. Maternal weight and pregnancy complications. American journal of obstetrics and gynecology 1985;152:238-45.
12. Guelinckx I, Devlieger R, Beckers K, Vansant G. Maternal obesity: pregnancy complications, gestational weight gain and nutrition. Obesity reviews : an official journal of the International Association for the Study of Obesity 2008;9:140-50.
13. Sarwer DB, Allison KC, Gibbons LM, Markowitz JT, Nelson DB. Pregnancy and obesity: a review and agenda for future research. J Womens Health (Larchmt) 2006;15:720-33.
14. Consensus on infertility treatment related to polycystic ovary syndrome. Fertility and sterility 2008;89:505-22.
15. Consensus on infertility treatment related to polycystic ovary syndrome. Hum Reprod 2008;23:462-77.
16. Balen AH, Anderson RA, Policy, Practice Committee of the BFS. Impact of obesity on female reproductive health: British Fertility Society, Policy and Practice Guidelines. Hum Fertil (Camb) 2007;10:195-206.
17. Johnson NP, Stewart AW, Falkiner J, et al. PCOSMIC: a multi-centre randomized trial in women with PolyCystic Ovary Syndrome evaluating Metformin for Infertility with Clomiphene. Hum Reprod 2010;25:1675-83.

18. Farquhar CM, Gillett WR. Prioritising for fertility treatments--should a high BMI exclude treatment? BJOG : an international journal of obstetrics and gynaecology 2006;113:1107-9.
19. Anderson K, Norman RJ, Middleton P. Preconception lifestyle advice for people with subfertility. Cochrane Database of Systematic Reviews 2010:CD008189.
20. Hull MG, Glazener CM, Kelly NJ, et al. Population study of causes, treatment, and outcome of infertility. Br Med J (Clin Res Ed) 1985;291:1693-7.
21. Frisch RE, Revelle R. Height and weight at menarche and a hypothesis of critical body weights and adolescent events. . Science 1970 169:397-9.
22. Legro RS, Lin HM, Demers LM, Lloyd T. Rapid maturation of the reproductive axis during perimenarche independent of body composition. The Journal of clinical endocrinology and metabolism 2000;85:1021-5.
23. Wang Y. Is obesity associated with early sexual maturation? A comparison of the association in American boys versus girls. Pediatrics 2002;110:903-10.
24. Chen ZJ, Zhao H, He L, et al. Genome-wide association study identifies susceptibility loci for polycystic ovary syndrome on chromosome 2p16.3, 2p21 and 9q33.3. Nature genetics 2011;43:55-9.
25. Rochester D, Jain A, Polotsky AJ, et al. Partial recovery of luteal function after bariatric surgery in obese women. Fertility and sterility 2009;92:1410-5.
26. Legro RS, Dodson WC, Gnatuk CL, et al. Effects of gastric bypass surgery on female reproductive function. The Journal of clinical endocrinology and metabolism 2012;97:4540-8.
27. Sarwer DB, Spitzer JC, Wadden TA, et al. Changes in sexual functioning and sex hormone levels in women following bariatric surgery. JAMA surgery 2014;149:26-33.
28. Tsagareli V, Noakes M, Norman RJ. Effect of a very-low-calorie diet on in vitro fertilization outcomes. Fertility and sterility 2006;86:227-9.
29. Morris SN, Missmer SA, Cramer DW, Powers RD, McShane PM, Hornstein MD. Effects of lifetime exercise on the outcome of in vitro fertilization. Obstetrics and gynecology 2006;108:938-45.
30. Gudmundsdottir SL, Flanders WD, Augestad LB. Physical activity and fertility in women: the North-Trondelag Health Study. Hum Reprod 2009;24:3196-204.
31. De Souza MJ, Toombs RJ, Scheid JL, O'Donnell E, West SL, Williams NI. High prevalence of subtle and severe menstrual disturbances in exercising women: confirmation using daily hormone measures. Hum Reprod 2010;25:491-503.
32. Loucks AB, Verdun M, Heath EM. Low energy availability, not stress of exercise, alters LH pulsatility in exercising women. Journal of applied physiology 1998;84:37-46.
33. De Souza MJ, Lee DK, VanHeest JL, Scheid JL, West SL, Williams NI. Severity of energy-related menstrual disturbances increases in proportion to indices of energy conservation in exercising women. Fertility and sterility 2007;88:971-5.
34. Anonymous. Clinical guidelines on the identification, evaluation, and treatment of overweight and obesity in adults--the evidence report. National institutes of health. [See comments.] [Erratum appears in obes res 1998 nov;6(6):464.]. [Review] [769 refs]. Obesity Research 6 Suppl 2:51S-209S, 1998 Sep.
35. Heshka S, Anderson JW, Atkinson RL, et al. Weight loss with self-help compared with a structured commercial program: a randomized trial. JAMA : the journal of the American Medical Association 2003;289:1792-8.
36. Tate DF, Wing RR, Winett RA. Using Internet technology to deliver a behavioral weight loss program. JAMA : the journal of the American Medical Association 2001;285:1172-7.

37. Tate DF, Jackvony EH, Wing RR. Effects of Internet behavioral counseling on weight loss in adults at risk for type 2 diabetes: a randomized trial. JAMA : the journal of the American Medical Association 2003;289:1833-6.
38. Sigal RJ, Kenny GP, Wasserman DH, Castaneda-Sceppa C, White RD. Physical activity/exercise and type 2 diabetes: a consensus statement from the American Diabetes Association. Diabetes care 2006;29:1433-8.
39. Stofan JR, DiPietro L, Davis D, Kohl HW, 3rd, Blair SN. Physical activity patterns associated with cardiorespiratory fitness and reduced mortality: the Aerobics Center Longitudinal Study. American journal of public health 1998;88:1807-13.
40. Hu FB, Stampfer MJ, Solomon C, et al. Physical activity and risk for cardiovascular events in diabetic women. Annals of internal medicine 2001;134:96-105.
41. Foster-Schubert KE, Alfano CM, Duggan CR, et al. Effect of diet and exercise, alone or combined, on weight and body composition in overweight-to-obese postmenopausal women. Obesity (Silver Spring) 2012;20:1628-38.
42. Pasquali R, Casimirri F, Venturoli S, et al. Body fat distribution has weight-independent effects on clinical, hormonal, and metabolic features of women with polycystic ovary syndrome. Metabolism 1994;43:Clinical &-Clinical 13.
43. Smith AJ, Phipps WR, Arikawa AY, et al. Effects of aerobic exercise on premenopausal sex hormone levels: results of the WISER study, a randomized clinical trial in healthy, sedentary, eumenorrheic women. Cancer epidemiology, biomarkers & prevention : a publication of the American Association for Cancer Research, cosponsored by the American Society of Preventive Oncology 2011;20:1098-106.
44. Jensen MD, Ryan DH, Apovian CM, et al. 2013 AHA/ACC/TOS Guideline for the Management of Overweight and Obesity in Adults: A Report of the American College of Cardiology/American Heart Association Task Force on Practice Guidelines and The Obesity Society. Circulation 2013.
45. Hollander PA, Elbein SC, Hirsch IB, et al. Role of orlistat in the treatment of obese patients with type 2 diabetes. A 1-year randomized double-blind study. Diabetes Care 1998;21:1288-94.
46. Hill JO, Hauptman J, Anderson JW, et al. Orlistat, a lipase inhibitor, for weight maintenance after conventional dieting: a 1-y study. The American journal of clinical nutrition 1999;69:1108-16.
47. Davidson MH, Hauptman J, DiGirolamo M, et al. Weight control and risk factor reduction in obese subjects treated for 2 years with orlistat: a randomized controlled trial. JAMA : the journal of the American Medical Association 1999;281:235-42.
48. Sjostrom L, Rissanen A, Andersen T, et al. Randomised placebo-controlled trial of orlistat for weight loss and prevention of weight regain in obese patients. European Multicentre Orlistat Study Group. Lancet 1998;352:167-72.
49. Bray GA, Ryan DH, Gordon D, Heidingsfelder S, Cerise F, Wilson K. A double-blind randomized placebo-controlled trial of sibutramine. Obesity research 1996;4:263-70.
50. Rolls BJ, Shide DJ, Thorwart ML, Ulbrecht JS. Sibutramine reduces food intake in non-dieting women with obesity. Obesity research 1998;6:1-11.
51. Hansen DL, Toubro S, Stock MJ, Macdonald IA, Astrup A. The effect of sibutramine on energy expenditure and appetite during chronic treatment without dietary restriction. International journal of obesity and related metabolic disorders : journal of the International Association for the Study of Obesity 1999;23:1016-24.
52. Arterburn DE, Crane PK, Veenstra DL. The efficacy and safety of sibutramine for weight loss: a systematic review. Archives of internal medicine 2004;164:994-1003.

53. Kim SH, Lee YM, Jee SH, Nam CM. Effect of sibutramine on weight loss and blood pressure: a meta-analysis of controlled trials. Obesity research 2003;11:1116-23.
54. Yanovski SZ, Yanovski JA. Obesity. The New England journal of medicine 2002;346:591-602.
55. Stevens VJ, Corrigan SA, Obarzanek E, et al. Weight loss intervention in phase 1 of the Trials of Hypertension Prevention. The TOHP Collaborative Research Group. Archives of internal medicine 1993;153:849-58.
56. Dattilo AM, Kris-Etherton PM. Effects of weight reduction on blood lipids and lipoproteins: a meta-analysis. The American journal of clinical nutrition 1992;56:320-8.
57. Jeffery RW, Wing RR, Thorson C, et al. Strengthening behavioral interventions for weight loss: a randomized trial of food provision and monetary incentives. Journal of Consulting & Clinical Psychology 1993;61:1038-45.
58. Metz JA, Stern JS, Kris-Etherton P, et al. A randomized trial of improved weight loss with a prepared meal plan in overweight and obese patients: impact on cardiovascular risk reduction. Archives of internal medicine 2000;160:2150-8.
59. Ditschuneit HH, Flechtner-Mors M, Johnson TD, Adler G. Metabolic and weight-loss effects of a long-term dietary intervention in obese patients. American Journal of Clinical Nutrition 1999;69:198-204.
60. Heymsfield SB, van Mierlo CA, van der Knaap HC, Heo M, Frier HI. Weight management using a meal replacement strategy: meta and pooling analysis from six studies. International journal of obesity and related metabolic disorders : journal of the International Association for the Study of Obesity 2003;27:537-49.
61. Wadden TA, Butryn ML, Byrne KJ. Efficacy of lifestyle modification for long-term weight control. Obesity research 2004;12 Suppl:151S-62S.
62. Lichtman SW, Pisarska K, Berman ER, et al. Discrepancy between self-reported and actual caloric intake and exercise in obese subjects. New England Journal of Medicine 1992;327:1893-8.
63. Bray GA, Blackburn GL, Ferguson JM, et al. Sibutramine produces dose-related weight loss. Obesity research 1999;7:189-98.
64. Wadden TA, Berkowitz RI, Sarwer DB, Prus-Wisniewski R, Steinberg C. Benefits of lifestyle modification in the pharmacologic treatment of obesity: a randomized trial. Archives of internal medicine 2001;161:218-27.
65. Stafford RS, Farhat JH, Misra B, Schoenfeld DA. National patterns of physician activities related to obesity management. Arch Fam Med 2000;9:631-8.
66. Wadden TA, Anderson DA, Foster GD, Bennett A, Steinberg C, Sarwer DB. Obese women's perceptions of their physicians' weight management attitudes and practices. Arch Fam Med 2000;9:854-60.
67. Power ML, Cogswell, M.E., Schulkin J. Obesity Prevention and Treatment Practices of U.S. Obstetrician-Gynecologists. Obstetrics and gynecology 2006;108:961-8.
68. Frank A. Futility and avoidance. Medical professionals in the treatment of obesity. JAMA : the journal of the American Medical Association 1993;269:2132-3.
69. Kushner RF. Barriers to providing nutrition counseling by physicians: a survey of primary care practitioners. Prev Med 1995;24:546-52.
70. Moran LJ, Brinkworth G, Noakes M, Norman RJ. Effects of lifestyle modification in polycystic ovarian syndrome. Reproductive biomedicine online 2006;12:569-78.

71. Clark AM, Thornley B, Tomlinson L, Galletley C, Norman RJ. Weight loss in obese infertile women results in improvement in reproductive outcome for all forms of fertility treatment. Hum Reprod 1998;13:1502-5.
72. Reddy UM, Wapner RJ, Rebar RW, Tasca RJ. Infertility, assisted reproductive technology, and adverse pregnancy outcomes: executive summary of a National Institute of Child Health and Human Development workshop. Obstetrics and gynecology 2007;109:967-77.
73. Schieve LA, Meikle SF, Ferre C, Peterson HB, Jeng G, Wilcox LS. Low and very low birth weight in infants conceived with use of assisted reproductive technology. The New England journal of medicine 2002;346:731-7.
74. Kissin DM, Kulkarni AD, Kushnir VA, Jamieson DJ, National ARTSSG. Number of embryos transferred after in vitro fertilization and good perinatal outcome. Obstetrics and gynecology 2014;123:239-47.
75. Kulkarni AD, Jamieson DJ, Jones HW, Jr., et al. Fertility treatments and multiple births in the United States. The New England journal of medicine 2013;369:2218-25.
76. Moran L, Tsagareli V, Norman R, Noakes M. Diet and IVF pilot study: short-term weight loss improves pregnancy rates in overweight/obese women undertaking IVF. The Australian & New Zealand journal of obstetrics & gynaecology 2011;51:455-9.
77. Sim KA, Dezarnaulds GS, Denyer MR, Skilton MR, Carterson ID. Weight loss improves reproductive outcomes in obese women undergoing fertility treatment: a randomized controlled trial. Clinical Obesity 2014;4:61-8.
78. Legro RS, Barnhart HX, Schlaff WD, et al. Clomiphene, metformin, or both for infertility in the polycystic ovary syndrome. The New England journal of medicine 2007;356:551-66.
79. Group LAR. The Look AHEAD study: a description of the lifestyle intervention and the evidence supporting it. Obesity research 2006:In Press.
80. Committee on Practice B-G. ACOG Practice Bulletin Number 131: Screening for cervical cancer. Obstetrics and gynecology 2012;120:1222-38.
81. Hatch R, Rosenfield RL, Kim MH, Tredway D. Hirsutism: implications, etiology, and management. . American Journal of Obstetrics & Gynecology 1981 Aug 1;140:815-30.
82. Pache TD, Hop WC, Wladimiroff JW, Schipper J, Fauser BC. Transvaginal sonography and abnormal ovarian appearance in menstrual cycle disturbances. Ultrasound in Medicine & Biology 1991;17:589-93.
83. Kroenke K, Spitzer RL, Williams JB. The PHQ-9: validity of a brief depression severity measure. Journal of general internal medicine 2001;16:606-13.
84. Ware JE SK, Kosinski M, Gandek B. SF-36 health survey manual and interpretation guide. Boston: New England Medical Center,: The Health Institute; 1993.
85. Rosen R, Brown C, Heiman J, et al. The Female Sexual Function Index (FSFI): a multidimensional self-report instrument for the assessment of female sexual function. Journal of sex & marital therapy 2000;26:191-208.
86. Rosen RC, Riley A, Wagner G, Osterloh IH, Kirkpatrick J, Mishra A. The international index of erectile function (IIEF): a multidimensional scale for assessment of erectile dysfunction. Urology 1997;49:822-30.
87. Boivin J, Takefman J, Braverman A. The fertility quality of life (FertiQoL) tool: development and general psychometric properties. Hum Reprod 2011;26:2084-91.
88. Chung F, Yang Y, Liao P. Predictive performance of the STOP-Bang score for identifying obstructive sleep apnea in obese patients. Obesity surgery 2013;23:2050-7.
89. Johns MW. A new method for measuring daytime sleepiness: the Epworth sleepiness scale. Sleep 1991;14:540-5.

90. Heinze G, Schemper M. A solution to the problem of separation in logistic regression. Statistics in medicine 2002;21:2409-19.
91. Laird NM, Ware JH. Random-effects models for longitudinal data. Biometrics 1982;38:963-74.
92. Zeger SL, Liang KY, Albert PS. Models for longitudinal data: a generalized estimating equation approach. Biometrics 1988;44:1049-60.
93. Donders AR, van der Heijden GJ, Stijnen T, Moons KG. Review: a gentle introduction to imputation of missing values. Journal of clinical epidemiology 2006;59:1087-91.
94. Little RR, DB. Chapter 15: Nonignorable Missing-Data Models. Statistical Analysis with Missing Data,. 2nd ed. ed. New York, NY: Wiley; 2002.

15 Human Subjects/Informed Consent/Female

COMBINED INFORMED CONSENT AND
HIPAA AUTHORIZATION FORM
Penn State College of Medicine
The Milton S. Hershey Medical Center

**Protocol Title:**	Improving Reproductive Fitness with Pretreatment with Lifestyle Modification in Obese Women with Unexplained Infertility: FIT-PLESE
**Principal Investigator:**	Richard Legro, M.D.<br>Obstetrics & Gynecology Department, Division of Reproductive Endocrinology<br>500 University Drive, Room C3604, MCH103<br>Hershey, PA 17033
**Emergency Contact:**	Weekdays: 8:00 a.m. to 5:00 p.m. (717) 531-8478.<br>After hours call (717) 531-8521 and ask for the Ob-Gyn doctor on 24-hour call.

Why am I being asked to volunteer?

We are asking you to be in this research because you have been diagnosed as being overweight with unexplained infertility and are seeking pregnancy. Your participation is voluntary which means you can choose whether or not you want to participate. If you choose not to participate, there will be no loss of benefits to which you are otherwise entitled. Before you can make your decision, you will need to know what the study is about, the possible risks and benefits of being in this study, and what you will have to do in this study. The research team is going to talk to you about the research study, and they will give you this consent form to read. You may also decide to discuss it with your family, friends, or family doctor. You may find some of the medical language difficult to understand. Please ask the study doctor and/or the research team about this form. If you decide to participate, you will be asked to sign this form.

What is the purpose of this research study?

We are asking you to be in this research because you have been diagnosed as being overweight with unexplained infertility and are seeking pregnancy.

This research is being done to find out the effects of lifestyle modification in obese women with unexplained infertility. Phase 1 of the study is 16 weeks and includes two types of lifestyle modifications: 1) increased physical activity by increasing steps per day OR 2) a calorie restricted diet using meal replacements and a weight loss medication called Orlistat (Alli) with increased physical activity. Orlistat 60mg is a United States Food and Drug Administration (FDA) approved medication to be taken with meals and works by reducing the absorption of fat in a meal.

After completion of Phase 1 lifestyle modification, you will move to Phase 2 of the study. This will involve 3 cycles of infertility treatment involving ovarian stimulation with an oral medication (clomiphene citrate) followed by triggered ovulation using hCG injection and single intrauterine insemination. Clomiphene Citrate is approved by the FDA for the treatment of infertility. hCG (human chorionic gonadotropin) is an injectable medicine to trigger ovulation and is also FDA approved.

How long will I be in the study?

If you agree to take part, it will take you about 18 months to complete this research study. Below is a time table that lists the visits and their length of times:

Visit Name	# of Visits	Approximate Length of time
Screening	1	2-3 hours
Baseline	1	1 hour
Phase 1-Lifestyle Monthly Visit 1, 2, & 3	3	1 hour each
Lifestyle Modification Close-out/End of Phase 1	1	1 ½ hours
Phase 2- Infertility Treatment Baseline Cycle 1	1	1 hour
Monitoring	1+ additional as required(varies)	30 minutes
Insemination	Up to 3	1 hour
Serum Pregnancy Test	2 + additional as required	10 minutes
Infertility Treatment Close-out/End of Phase 2	1	1 ½ hours
Pregnancy 16 weeks	1	30 minutes
Pregnancy 24 weeks	1	30 minutes
Pregnancy 32 weeks	1	30 minutes
POSSIBLE TOTALS=	***17 visits***	***13 hours***

How many subjects will be in the study?

Approximately 380 women and their partner/spouse will take part in this research study nationwide as part of the Reproductive Medicine Network (RMN). Approximately 30 will be from the Penn State Hershey Medical Center.

What am I being asked to do?

At initial contact either by email, telephone, or in clinic, you will be screened with a brief eligibility questionnaire to determine if you qualify to take part in this research. If you are determined to be eligible, you will be scheduled for a screening visit. If you have had any of these tests performed recently within the time limitations set forth in the study, the results of the tests will be recorded and the tests will not be repeated for this study. This consent form will be reviewed by you and the study coordinator. The purpose of the study, all procedures involved, the risks, and the time commitment related to this study will be discussed. Once all questions have been answered, you will sign this consent form. A copy of this signed consent will be given to you for your records.

Below is a brief chart explaining the visits and procedures to be completed for the study. Following this chart, you will find explanations of the visits and each procedure in more detail.

Phases	Phase 1: Lifestyle Intervention (16 weeks)				Phase 2: Infertility Treatment						Phase 3: Pregnancy
Visit Titles	*Screen (can be done in 2 visits)*	*Baseline/Drug Assign*	*Month Visit 1, 2 & 3*	*End of Phase 1 (week 16)*	*Cycle 1 Baseline (day1-3 of menses)*	*Monitor Visit *(cycle day 8-12)*	*IUI*	*UrinePreg Test ***	*Serum hCG ****	*End of Phase 2*	*16, 24, 32 week Pregnancy Visit*
Informed Consent	X										
Medical Assessment	X										
Vital Signs & Biometrics	X	X	X	X	X					X	X
Complete Physical Exam	X										
Gynecological Exam and pap smear	X										
Acne/Hirsutism Assessment	X			X						X	
Transvaginal ultrasound	X				X	X					
Sonohysterogram or Hysterosalpingogram	X										
Questionnaires & QOL surveys	X			X						X	
Urine pregnancy test	X		X	X	X			X (@ home)			
Serum pregnancy test									X		
Blood work for Rubella, Varicella, HIV, genetic tests and hgb electrophoresis (optional)	X										
Blood work for female hormones and HgbA1C	X										
Blood work for safety labsand lipid panel	X			X						X	
Blood work for central core	X	X	X	X	X					X	X
Blood work and urine for reserve ancillary studies (optional)	X	X	X	X	X					X	X
Blood work for Whole blood (optional)		X									
Blood work for RMN Repository (optional)		X									
Vaginal Swab	X				X						X
Rectal swab	X				X						X
Saliva sample (optional)		X		X							
Dispense meds		X	X		X	X					
Collect meds			X	X		X				X	
Order meal replacements (if applicable)		X	X								
Dispense Fitbit and Aria scale	X	X									
Dispense diaries		X	X		X	X					
Collect diaries			X	X		X				X	
Adverse Events & Concomitant Meds		X	X	X	X	X		X		X	X
Intrauterine Insemination							X				
MALE PROCEDURES											
Informed Consent	X										
Medical Assessment	X										
Vital signs & Biometrics	X									X	

Questionnaires & QOL surveys	X									X	
Semen analysis/collection	X						X				
Semen for RMN Repository (optional)	X										
Blood work and urine for RMN repository (optional)	X										
Saliva sample (optional)	X										

*Monitor visits will be repeated on an individual basis until hCG administration
**To be done 2 weeks after insemination at home. Repeat cycles until pregnancy occurs or treatment cycles end; may have hCG up to 3 times (cycles). If positive pregnancy, will schedule visit for serum pregnancy test and move to Phase 3 for follow-up. If negative, start new cycle.
***Will repeat hCG 2 days after + result. OB ultrasound will be scheduled 14-21 days from + result.

<u>Screening Visit -</u> This visit will take approximately 2 hours to complete. You will report to the medical center after you have had *<u>nothing to eat or drink 8 hours prior to your visit</u>*. The following procedures are research related and will take place during this visit:

- This consent form will be reviewed by you and the study coordinator. You will have an opportunity to read this consent form in full and ask any questions you may have about the procedures involved, risks and time commitments related to this study. Once all of your questions have been answered, you will sign this consent form. A copy will be provided to you for your records.
- Your past medical and menstrual history will be recorded. This form will ask a series of questions about your medical health, family health history, reproductive and gynecological history, pregnancy history, and current use of medications.
- Your height, weight, vital signs (blood pressure and pulse) and hip and abdominal circumference will be collected and your BMI (body mass index) will be calculated.
- A urine pregnancy test will be performed.
- A physical exam including breast and pelvic exam will be performed by the physician if not done within the past 12 months. A pap smear will be collected if you are 21 or older and have not had one within the time period specified by current guidelines. A vaginal swab will also be collected and tested for vaginal microbiome (normal organisms found in the vagina). This is done by swabbing the vaginal wall using a cotton swab and then placed in a special media. A rectal swab will also be collected and tested for stool microbiome (normal organisms found in the gut). This is done by swabbing the rectal wall using a cotton swab and then placed in a special media.
- The amount of acne lesions on your face will be counted and your hirsutism (excessive hair growth) assessment will be completed.
- You will be given 9-10 questionnaires to complete. A Risk Factor for Genetic Disorders questionnaire is completed to determine if you have an increased risk of having a baby with a genetic disorder. Any potential problems that may lead to complications during pregnancy will be addressed at this time and we may refer you to a genetics counselor prior to starting the study. We will also give you questionnaires concerning your sexual function, feelings of depression, daily activities, sleep habits, and past and current dietary history. You will be free to skip any questions that you would prefer not to answer or that make you uncomfortable.

- Blood work will be drawn to determine your eligibility for the study. This blood will consist of checking hormone levels to evaluate you for specific causes of infertility. If no reason for your infertility is identified, then you will be considered to have unexplained infertility. You will also have blood work to be sure your liver and kidneys are working normally and that you are not anemic (a condition where your blood lacks healthy red blood cells). Approximately 8 tablespoons(6 tablespoons if not consented to optional section of study) of blood will be drawn at this visit for the following:

  - Checking hormone levels

  - Checking safety labs

    **If you have already had some of these hormone tests and a lipid panel done within the past year and the results were within normal range and we can obtain a copy of these results, you will not need to have these tests repeated.*

  - Blood will be collected for testing at the central core laboratory. Each site participating in this study will collect a blood sample from every participant at screening and each visit thereafter to be tested by this central core lab for hormone levels and other tests as decided by the RMN.

  - Blood (2 tablespoons) and urine reserve samples will also be collected at this time and stored on site to be used for future testing if you consent to this optional section of the study.

  - Additionally you will be offered optional blood test to determine if you are immune to rubella (German Measles) and Varicella (Chicken Pox), or to see if you are infected with the HIV virus. If any of these tests are abnormal, you will be referred for appropriate treatment and counseling, however, they will not prevent you from being eligible for the study. If the HIV test is positive, we must report it to the State Health Department and someone from the department may contact you. These tests are <u>NOT</u> included or required for the study and will be at your cost.

  -You will also be offered optional recommended genetic tests and hemoglobin electrophoresis (test performed to test different types of hemoglobin, which transfers oxygen in the blood) per standard guidelines. If any of these tests are abnormal, you will be referred for appropriate treatment and counseling, however, they will not prevent you from being eligible for the study. These tests are <u>NOT</u> included or required for the study and will be at your cost.

The following procedures are a standard form of care and would be performed as part of an infertility work up even if you were not a participant in the study.

- A transvaginal ultrasound will be performed. This involves inserting an ultrasound probe into your vagina to visualize your ovaries and uterus. Measurements and ultrasound pictures will be recorded of your ovaries and uterus.
- At this time, if you have had not had a test within the past 3 years to determine that your fallopian tubes are open, a sonohysterogram (SHG) or hysterosalpingogram (HSG) will be completed. During the SHG, sterile saline fluid is injected through an intrauterine catheter

that contains a balloon. The balloon is inflated and the shape of your uterus can be seen and the fluid that accumulates in the back area of your uterus determines that at least one fallopian tube is open. If it can't be determined by the SHG, it will be necessary for a different procedure to be performed. An HSG procedure is done in the radiology department using radio-opaque contrast dye that is injected into the uterus and is visualized flowing through the fallopian tube. If you have been pregnant within the last three years and your pregnancy and delivery were uncomplicated, the SHG or HSG may not be necessary. You will be separately consented for these procedures if you need one of them.

- Your partner will need to provide a semen sample for analysis. A semen analysis is required to determine a sperm concentration of greater than 5 million total motile sperm (percent of sperm swimming forward) in an ejaculate for entry into the study. If your partner/spouse has documentation with the past year of a semen analysis, he will be asked to sign a medical release form permitting the coordinator to obtain his semen analysis records, and he will not need to repeat the semen analysis testing if results are within study limits.

<u>Randomization/Beginning of Phase 1 Lifestyle Modification</u>

After the screening visit, if you are eligible to continue in the research, you will be scheduled for your baseline visit to begin Phase 1 of the study - Lifestyle Modification. You will be randomly assigned to receive one of the two study treatments. Randomization means whichever study treatment you receive it will be determined purely by chance, like a flip of a coin. You will have an equal chance to receive either increased physical activity only or increased physical activity with dietary modifications and medication. Neither you nor your physician will be able to decide which group you are assigned to. The following is a detailed description of the lifestyle treatments you could receive:

<u>-Lifestyle Arm A: Increased Physical Activity</u>

In this arm, you will be required to increase your physical activity. We will use your Fitbit measurements from screening to determine your baseline average number of steps per day. You will be asked to increase your amount of steps by 500 per day per week until you have reached the upper limit of 10,000 steps per day. You will be asked to then maintain that amount of steps over the 16 week period. There will be no restrictions on your diet. You will be given a prenatal vitamin with 800mcg of folic acid (a B vitamin) to be taken once a day.

<u>-Lifestyle Arm B: Increased Physical Activity with weight loss</u>

In this arm, you will be required to achieve weight loss through restricting/decreasing your calories, increasing your physical activity and taking weight loss medication. You will receive information on ordering meal replacements and also receive a medication during this 16 week treatment period. You will take one tablet of Orlistat 60 mg (brand name Alli) twice a day at lunch and dinner. You will be instructed by the study coordinator when to begin your medication. You will continue this medication for 14 weeks with a 2 week break from medication before moving on to the next phase of the study. You will be given a prenatal vitamin with folic acid (a B vitamin) to be taken once a day at least 2 hours before or after Orlistat to ensure adequate vitamin effects because the Orlistat can interfere with the absorption of fat soluable vitamins, such as Vitamin D. You will also receive a complete daily diet plan for the 16 weeks with meal replacements (MR) which will include two prepared entrees for lunch and dinner and

a cereal bar or beverage. You will be provided with the meal replacements through participation in the study as we will be considering the meal replacements as a prescribed medical treatment. In addition, you will be instructed to also consume 2 servings of fruit (1 serving= 1 small-medium fresh fruit, ½ canned or fresh fruit, 1 cup berries), 3 servings of vegetables (1 serving= ½ cooked vegetables, 1 cup raw vegetables), and 2 servings of low fat dairy (1 serving= 1 cup skim milk, 1 container 6 or 8 oz yogurt, 1 ½ oz natural cheese) per day. This will provide you about 1100 calories. You will be required to consume a total of 1200 calories per day so 100 calories can be consumed with something of your choice. You will also be required to follow the increased physical activity plan as described in Lifestyle A.

Both groups will receive the following during their baseline visit:

- A weight measurement and vital signs will be recorded.
- You will be dispensed a Fitbit Wireless Activity monitor. This will be used throughout the study to determine your daily steps. You will be instructed to download the Fitbit app to your smartphone and sync your Fitbit to the app. If you are unable to use the smart phone app, instructions will be provided to sync to your home computer. All data will be sent via Wi-Fi to the secure database for review.
- You will be dispensed an Aria Scale to monitor your weight. You will be required to weigh yourself weekly. This Wi-Fi smart scale will send your data to the secure database for review. If you are unable to use the Aria scale wirelessly, you will be required to contact the study coordinator via email, text or phone call to report your result.
- You will be given daily journal logs to complete. On these logs you will record menstrual cycles, dates when you took your study medication, intercourse frequency and side effects you have from the medications. You will also keep a list of all medications such as over-the-counter, herbal, or vitamins you are taking.
- If you are assigned to Arm B you will receive your medication, vitamins, and instructions on ordering your meal replacements.
- You will have approximately 6 tablespoons of blood drawn for the following:

  - Blood will be drawn for the central core laboratory.

  - Blood and urine for reserve will be collected (if consented).

  - If you have consented for your blood to be drawn for the study whole blood collection and/or the RMN Biologic Repository, this will be drawn at this time. The purpose of the whole blood collection is to identify certain key genes in your DNA (called polymorphisms) that may predict a response to the medications used in this trial. The repository collection will be used for future studies. These specimens will only be drawn if you have provided consent to do so.
- If you have consented to the collection of saliva for testing for biomarkers of stress, this will be collected at this time. This will be done by removing a cotton swab from the package and placing the swab under your tongue for approximately 60-120 seconds. Once the time is complete, the swab is inserted into the tube and sealed for storage in a freezer.

<u>Monthly Visit (1, 2, & 3)-</u>

You will return at 4, 8 and 12 weeks for a monthly visit. The following procedures will occur at each monthly visit:

- A weight measurement and vital signs will be recorded.
- A urine pregnancy test will be done.
- Approximately 3 tablespoons of blood and a urine specimen will be collected for the ancillary reserve (if consented) and central core laboratory.
- Assessment of your adverse events and concomitant medications will be collected.
- The study coordinator will collect all menstrual/intercourse diaries and any leftover medication and a pill count will be performed for compliance, if applicable.
- New menstrual/intercourse diaries will be re-distributed.
- If applicable, medication will be dispensed and you will be instructed to order your meal replacements.
- During the 3rd monthly visit, you will be dispensed kits for saliva collection to be returned at Closeout Visit (week 16), if consented.

Lifestyle Modification Close-out/End of Phase 1-

At approximately 16 weeks after completing your lifestyle modification phase, you will have a closeout visit for this phase. You will need to be fasting for this visit. The following procedures will occur at this visit:

- Measurements of height, weight, abdominal circumference and vital signs will be recorded.
- A urine pregnancy test will be done.
- Hirsutism and acne assessments will be completed.
- Approximately 4 tablespoons of blood will be collected for safety labs, central core labs and blood and urine reserve for the ancillary storage (if consented).
- If you consented to the collection of saliva for testing for biomarkers of stress, this sample will again be collected at this visit.
- You will be asked to complete the quality of life questionnaires as you did at the screening visit.
- Your menstrual and diary logs will be collected.
- Any remaining medications will be collected and pill count will be performed for compliance.
- Assessment of your adverse events and concomitant medications will be collected.

Baseline Visit Infertility Treatment Phase 2-

You will call on the first day of your next menstrual period (menstrual cycle day 1) after completing Phase 1 to schedule your baseline visit for infertility treatment between menstrual cycle day 1-5. At this visit the following procedures will occur:

- A weight measurement will be collected.
- You will have a transvaginal ultrasound and a repeat vaginal and rectal swab for microbiome testing.
- A urine pregnancy test will be done.
- You will have 3 tablespoons of blood drawn for the central core and blood and urine reserve for the ancillary storage (if consented).
- You will be dispensed menstrual and intercourse diaries.
- Your study medications will be dispensed to you at this time. You will receive capsules containing Clomiphene Citrate. You will be instructed to begin your medications if your urine pregnancy test is negative and your ultrasound results are within a normal range. You will also receive hCG injection medication to be used when directed by the study

physician prior to your insemination. You and your partner will be taught the technique for administering an intramuscular injection. Below is a table describing the medication Clomiphene Citrate and its dosages:

**Study Medication**	**Interval**	**Dose**	**Method**	**Start**	**Finish**	**Future Cycles**
Clomiphene Citrate	Daily	100mg	Capsule	Day 3*	Day 7	Can be started at 50-150 mg/day= 1 to 3 pills

**Can be started +/- 2 days (menstrual cycle day 1 to 5)*

<u>Monitor Visits (Cycle day 8 to 12 and then beyond as required)-</u>

You will be required to return to the clinic within 3 days after completing your 5 day treatment of Clomiphene Citrate. A transvaginal ultrasound will be performed. You will report any adverse events or concomitant medications you are taking. You may be required to return to the clinic regularly for additional monitoring visits until you have met the criteria for hCG administration. These visits will be individualized and based on the physician's decision. At this visit during cycle 2 and 3, you will return your medication bottles and menstrual diaries from the previous cycle.

When your main follicle (cyst which contains an egg) gets to a certain size, you will be instructed to self- or partner- administer, as previously instructed, an injection of hCG at a dose of 10,000 IU. A cycle may be cancelled if the lead follicles become too large or too small after 18 days of treatment; the study doctor will decide this. If your lead follicles are too small after 18 days of treatment, your doctor may ask you to come in for some blood tests to see if you can start your next dose of study medication right away.

<u>Intrauterine Insemination Visit-</u>

You must return to the clinic within 44 hours of the hCG shot. Your partner will provide a semen sample. This sample will be washed and prepared for the insemination. A speculum will be placed in your vagina and a thin flexible catheter will be placed into your cervix. The sperm will be injected through the catheter and into your uterus. During cycle 1 and cycle 2, at this visit you will be dispensed medication and your menstrual diaries for the next cycle. You will be instructed to NOT start your medications until the end of the cycle after a confirmed negative urine pregnancy test.

<u>Urine Pregnancy Test-</u>

Two weeks after insemination you will do a urine pregnancy test at home and call the study coordinator with your results.

If you have a *<u>positive urine pregnancy test</u>*, you will be required to return to the clinic for a blood pregnancy test (quantitative bhCG). If your bhCG is positive you will return 2 days later for repeat bhCG to check for a rising level. If the level is not rising, you will begin the next cycle of treatment with the start of your menstrual cycle. If the bhCG level has risen, you will be scheduled for an ultrasound approximately 14-21 days after the positive result. This will determine the location of your pregnancy and number of gestational sacs. A repeat pregnancy ultrasound will be done

7-14 days after the first one to assess fetal heart beat. You may need additional ultrasounds done to evaluate the progress of the pregnancy if necessary.

Pregnancy care is not part of the study. You will be scheduled with your obstetrician for follow up prenatal care. If you do not have a doctor who delivers babies, you will be referred to one. You will be asked to sign a medical release form so the study coordinator can obtain your pregnancy and delivery information.

Follow up Initiation of Cycle 2 & 3-

If your urine pregnancy test is negative after a cycle, you may start another cycle of treatment. You may participate for a maximum of 3 completed cycles. A cycle may be cancelled if any significant adverse reactions develop in response to the medications, if you are unable to have the hCG injection, or if you request the cycle to be cancelled. The study coordinator will provide you with instructions to start your medication on cycle day 3 (+ or – 2 days). The study physician may change the start dose of your Clomiphene Citrate. You will be scheduled for a return monitoring visit during menstrual cycle day 8-12.

Infertility Treatment Close-out/End of Phase 2-

You will return for an end of study drug visit and will need to be fasting for this visits. If you do not conceive, this visit will take place at the end of the 3rd cycle. If you do conceive, the visit will take place as soon as possible after pregnancy is confirmed. The following procedures will be done:

- Measurements including height, weight, neck, waist and hip circumference will be collected.
- Hirsutism and acne assessments will be done.
- You will return remaining study drug and diaries.
- Assessment of adverse events and concomitant medications will be done.
- You will be asked to repeat the quality of life questionnaires as you did at the screening visit, excluding the DHQII. If you are pregnant, the FertiQOL questionnaire will be skipped.
- Approximately 4 tablespoons of blood will be collected for safety labs, central core labs and blood and urine reserve for ancillary storage (if consented).

Pregnancy Follow-up/Phase 3-

If you become pregnant during the study, you will participate in Phase 3. You will be seen for 3 visits at 16 weeks, 24 weeks and 32 weeks of pregnancy. You will return to the clinic for a brief visit and will need to be fasting for these visits. The following will occur:

- Measurements including waist and hip measurements, vital signs and weight will be obtained.
- You will have about 4 tablespoons of blood for central core labs, and blood and urine reserve for ancillary storage (if consented).
- Assessment of pregnancy adverse events will be done.
- A vaginal and rectal swab for stool microbiome will be collected.
- A medical release form will be signed to obtain copies of your prenatal record and delivery records.

- You will be offered the opportunity to participate in the Reproductive Medicine Network (RMN) Pregnancy Registry. The Registry is a separate study, so we will ask you to sign a separate consent form if you are interested.

Other visits which may occur

Unexpected visits, blood tests and procedures may occur during the study. These could include, for example, if your menses are late during Phase 2, an additional visit to the clinic or additional blood tests.

What are my responsibilities if I take part in this research?

If you take part in this research, your major responsibilities will include:

- Compliance with study visits
- Reviewing all of your medications and side effects with your study doctor
- Participation in study related procedures
- Participation in the collection of all blood work
- Taking and storing your study medication as instructed and returning all of the unused study drug and empty containers to the study site at each visit
- Compliance with the documentation and collection of menstrual and intercourse daily logs
- Compliance with the collection of all data for your exercise and meal replacement use

What are the possible risks or discomforts?

Below is a table listing all the tests and procedures involved in this research and their related discomforts or risks. You should check with the study doctor before starting any new prescriptions, over the counter medications, vitamins or herbal supplements.

Test or Procedure	Discomforts and Risks Associated
Standard venipuncture for blood work	Slight pinch or pin prick, discomfort, black and blue mark at the site of puncture, small blood clot, infection or bleeding at the site, and fainting during the procedure
Transvaginal ultrasound	Abdominal or pelvic discomfort
Sonohysterogram/Hysterosalpingogram	Pain, bleeding, damage to the uterus, pelvic infection, interrupting an unrecognized pregnancy, small amount of radiation exposure, allergic reaction to the radio-opaque dye
Clomiphene Citrate	Visual changes (such as blurring of vision, double vision, light sensitivity waves), abdominal pain, nausea, vomiting, constipation, mood changes, headache, hot flashes, respiratory difficulty, fatigue, abnormal endometrial

	thickening, multiple pregnancies, formation of ovarian cyst causing ovarian hyperstimulation syndrome, ovarian enlargement, breast discomfort, abnormal uterine bleeding, bloating, hair loss, arrhythmias, chest pain, allergic reaction, palpitations, and stroke or pulmonary embolism
Low calorie diet	Hunger, weakness, changes in your electrolytes, mood changes, depression, gall stone formation from rapid weight loss, lowered blood pressure with dizziness, lack of energy, and new onset attack of gout (presents with joint pain)
Daily exercising	Fatigue, muscle strain, joint pain, or injury from repetitive exercise, stroke or heart attack in susceptible individuals
Orlistat (Alli)	Headache, oily spotting, stomach pain/discomfort, gas with discharge, bowel movement urgency, fatty/oily stools, increased bowl movements, back pain, upper respiratory infection, fatigue, anxiety, sleep disorder, dry skin, menstrual irregularities, nausea, rectal pain/discomfort, tooth disorder, rash. Rarely liver injury has occurred. Symptoms include jaundice (yellow color in skin, gums, eye and other tissues due to too much bile in the blood), weakness and abdominal pain
Insemination	Vasovagal response (feeling lightheaded, dizzy, sweating), possible infection, pelvic discomfort, mild vaginal bleeding
Infertility treatments	Emotional distress at various degrees
Questionnaires	Some parts of the questionnaires may make you feel uncomfortable

It is not expected that patients will have any/all of these side effects. If you are feeling fatigue or dizziness associated with any of the medications, caution should be taken while driving or operating machinery. Side effects are usually temporary and manageable. However, it is possible they could be more serious.

The study treatment you receive may prove to be less effective or to have more side effects than the other research treatments or other available treatments.

There is a risk associated with loss of confidentiality if your medical information or your identity are obtained by someone other than the investigators, but precautions will be taken to prevent this from happening.

Although these drugs may result in pregnancy, there is no guarantee that this will result in live birth. Clomiphene has been associated with a 12.5% multiple pregnancy rate. Multiple pregnancies are more likely to end early in preterm labor and are more likely to experience most pregnancy complications including diabetes and hypertensive disorders of pregnancy. It is possible that your pregnancy may be nonviable or a pregnancy is detected in your fallopian tube (ectopic pregnancy) and will require further medical or surgical treatment. The risk for ectopic pregnancy is the same even if you were not participating in the study.

The particular treatments being used in this study may involve risks to you or to the embryo or fetus if you become pregnant and these risks are currently unforeseeable. You will be given pre-conceptional counseling for healthy behaviors in pregnancy throughout this study. You will be followed through study, after a positive pregnancy test, to confirm the location and viability of the pregnancy before being released to your Ob-Gyn physician. The use of these drugs may be associated with an increased risk for fetal malformations, though no specific pattern has been associated with either drug. Clomiphene Citrate has been shown to be genotoxic (cause abnormalities to genes) in in-vitro laboratory tests.

Risks of Genetic Testing:
A Federal law, called the Genetic Information Nondiscrimination Act (GINA), generally makes it illegal for health insurance companies, group health plans, and most employers to discriminate against you based on your genetic information.
This law generally will protect you in the following ways:

- Health insurance companies and group health plans may not request your genetic information that we get from this research.
- Health insurance companies and group health plans may not use your genetic information when making decisions regarding your eligibility or premiums.
- Employers with 15 or more employees may not use your genetic information that we get from this research when making a decision to hire, promote, or fire you or when setting the terms of your employment.

Be aware that this Federal law does not protect you or your family against genetic discrimination by companies that sell life insurance, disability insurance, or long-term care insurance. Also, GINA does not prohibit discrimination of individuals with a genetic disorder that has been diagnosed. However, in order to do everything possible to keep this from happening, the results of this test will NOT be given to anyone outside the study staff. This means that it will not be made available to you, your family members, your private physician, your employer, your insurance company or any other party as allowed by law.

In addition to the risks described above, there may be unknown risks we cannot predict while participating in this research. It is important that you notify your study coordinator if you experience any of these symptoms listed above and keep accurate documentation of these side effects in your daily journal logs. The investigators will let you know if they learn anything that might make you change your mind about participating in this research.

What if new information becomes available about the study?

During the course of this study, we may find more information that could be important to you. This includes information that, once learned, might cause you to change your mind about being in the study. We will notify you as soon as possible if such information becomes available.

What are the possible benefits of the study?

There is no guarantee that you will benefit from this research. The possible benefits you may experience from this research study include weight loss and a better chance for a healthier pregnancy. The information from the blood tests will let you know more about your health such as the levels of hormones in your blood involved in reproduction. The initial ultrasound examination will evaluate your pelvic anatomy, which may identify abnormalities such as ovarian cysts or abnormalities in the uterine lining. The infertility medications and intrauterine insemination may help you become pregnant.

The results of this research may guide the future of treatment of obesity for women with unexplained infertility by establishing effective lifestyle changes and weight loss prior to attempting pregnancy. This research may also help discover a better way to achieve pregnancy and a goal of a healthy pregnancy with live birth by the use of Clomiphene Citrate and intrauterine insemination for women with unexplained infertility.

What other choices do I have if I do not participate?

You do not have to take part in this study to be treated for your condition. Instead of participating in this research, you could:

- Receive commercially available treatments, including: over-the-counter orlistat or other weight loss medication; Clomid or other form of infertility medication along with intrauterine inseminations through your regular doctor.
- Be part of a different research study, if one is available.
- Choose not to be treated for your medical condition

Before you decide if you want to be in this research, we will discuss the other choices that are available to you. We will tell you about the possible benefits and risks of these choices.

The therapy offered in this research is available to you without taking part in this research study. Choosing not to participate will not affect your standard of care treatment.

Will I be paid for being in this study?

You will not receive any payment or compensation for being in this research study. You will keep the FitBit and Aria scale.

Will I have to pay for anything?

For costs of tests and procedures that are only being done for the research study:

- The orlistat, Clomiphene Citrate, hCG injection, and vitamins will be provided by RMN at no cost to you.

- You and/or your insurance company will not be charged for the cost of any tests or procedures that are required as part of the research and are within the standard of care (what is normally done) for your condition.
- The research-related tests and procedures that will be provided at no cost to you include:
    - All study required blood tests
    - Physical exam
    - Transvaginal ultrasound (s)
    - Pap smear
    - Sonohysterogram (SHG) and any required blood work needed to perform the test
    - Semen Analysis
    - Folic acid supplement and prenatal vitamins
    - Meal replacements
    - Fitbit
    - Aria scale
    - Intrauterine inseminations
    - Supplies for injection medication such as: syringes, alcohol swabs, gauze, and band-aids
    - 2 Obstetrical ultrasounds

For costs of medical services for care you would receive even if you were not in this research study:

- You and/or your insurance company will be responsible for the cost of routine medications, tests and procedures that you would receive even if you were not in this research that are not mentioned above as covered.
- You and/or your insurance company will be billed for the costs of these routine tests and procedures in the usual manner.
- You will be responsible for any co-payments, co-insurance and deductibles that are standard for your insurance coverage.
- You will be responsible for any charges not reimbursed by your insurance company.
- Some insurance companies will not pay for routine costs for people taking part in research studies. Before deciding to be in this research you should check with your insurance company to find out what they will pay for.

The following is a list of the non-covered services related to this study:

- Rubella, Varicella, & HIV testing
- Any genetic tests and hemoglobin electrophoresis
- Hysterosalpingogram (HSG) and any required blood work to perform the test
- Doxycycline prescription for after the HSG procedure
- Remaining obstetrical ultrasounds after the first 2
- Pregnancy care and delivery costs
- Any additional test which the doctor may order outside of the research procedures while you are in the study

If you have any questions about costs and insurance, ask the research study doctor or a member of the research team.

Who is paying for this research study?

The institution and investigators are receiving a grant from the National Institute of Health to support this research.

What happens if I am injured from being in the study?

It is possible that you could develop complications or injuries as a result of being in this research study. If you experience a side effect or injury and emergency medical treatment is required, seek treatment immediately at any medical facility. If you experience a side effect or injury and you believe that emergency treatment is not necessary, you should contact the principal investigator listed on the first page of this consent form as soon as possible and he/she will arrange for medical treatment.

<u>The Milton S. Hershey Medical Center (HMC)/Penn State College of Medicine (PSU) compensation for injury</u>

- There are no plans for HMC/PSU to provide financial compensation or free medical treatment for research-related injury.
- If an injury occurs, medical treatment is available at the usual charge.
- Costs will be charged to your insurance carrier or to you.
- Some insurance companies may not cover costs associated with research injuries.
- If these costs are not covered by your insurance, they will be your responsibility.

When you sign this form you are not giving up any legal right to seek compensation for injury.

When is the Study over? Can I leave the Study before it ends?

Taking part in this research study is voluntary.

- You do not have to be in this research.
- If you choose to be in this research, you have the right to stop at any time.
- If you decide not to be in this research or if you decide to stop at a later date, there will be no penalty or loss of benefits to which you are entitled.

If you stop being in the research, already collected data may not be removed from the study database. You will be asked whether the investigator can collect medical information from your routine medical care. If you agree, this data will be handled the same as research data. If you withdraw completely from the research study, no further information will be collected and your participation will end. You may discontinue taking part at any time without penalty or loss of benefits to which you are otherwise entitled. Your regular medical care at the facility will not be impacted in any way.

Your research doctor may take you out of the research study without your permission.

- Some possible reasons for this are: you experience serious side effects and continuing the research would be harmful, your condition has become worse, and you did not follow the instructions of the study doctor.
- Also, the sponsor of the research may end the research study early.
- If your participation ends early, you may be asked to visit the research doctor for a final visit.

If you will be in another clinical research study at the HMC/PSU or elsewhere while in this research, you should discuss the procedures and/or treatments with your physician or the study doctors. This precaution is to protect you from possible side effects from interactions of research drugs, treatments or testing.

Who can see or use my information? How will my personal information be protected?

Efforts will be made to limit the use and sharing of your personal research information. In our research files at HMC/PSU we will include these identifiers:

- Study identification number
- Name & Initials
- Address
- Phone number, cell phone number, work number
- Email address
- Social Security number
- Medical Record number
- Date of birth
- Visit date

A list that matches your name with your code number will be kept in a locked file.

Your research records will be labeled with your study identification number, your initials, date of birth and will be kept in a secure area. These records will be kept in the office for the amount of time necessary to complete data analysis and for a minimum of 5 years after completion of the study as required by law.

Results of some of the research-related tests including but not limited to your hormone, safety labs, and lipid blood results, pregnancy test results, ultrasound results, SHG/HSG results, pap smear results and intrauterine insemination information will be kept in your HMC/PSU medical record.

For research records sent to Yale University (the Data Coordinating Center), you will be identified by your study identification number only. The research records are entered into an electronic password protected database. Only HMC/PSU and RMN/Yale University investigators as well as investigators approved by the RMN to use the data will have access to these de-identified electronic records. This data is managed by the RMN/Yale University Data Coordinating Center and will be kept indefinitely.

Your research samples for the <u>central core laboratory</u> will be labeled with your study identification number, visit name and visit date and will be stored in the research laboratory in a -80 degree freezer until shipment. These samples are analyzed by the core lab and will be stored there until the sample is exhausted. If any remaining sample is leftover, they will eventually be sent to the RMN Biologic Repository and be owned by the RMN for future use in research until it has been exhausted. The list linking your sample to your identification number will be kept in a password protected computer file. Only investigators involved in this research will have access to this list.

For optional specimens collected as <u>reserve for ancillary studies</u>, you will identified by your study identification number, visit name and visit date. These samples are stored in the research laboratory in a -80 freezer. These samples will remain there until the RMN has decided on additional testing to be completed. These samples will be analyzed by the laboratory designated by the RMN and will be stored at that laboratory until the sample is exhausted. If any remaining sample is

leftover, they will eventually be sent to the RMN Biologic Repository and be owned by the RMN for future use in research until it has been exhausted.

For vaginal and rectal microbiome samples, and optional whole blood, stool and saliva samples you will not be identified. The sample will be labeled with a bar code label and a unique identifier. The sample will be stored in the research laboratory until shipment. These samples will remain there until the RMN has decided on the laboratory performing the testing. The samples will be sent to the laboratory and will be stored there until the sample is exhausted. If remaining sample is leftover, they will eventually be sent to the RMN Biologic Repository and be owned by the RMN for future use in research until it is exhausted.

For optional specimens collected for the RMN Biologic Repository, you will not be identified. The sample will be labeled with a bar code label and a unique identifier. The sample will be stored in the Repository until exhausted by the RMN.

To help us protect your privacy, we have obtained a Certificate of Confidentiality from the National Institute of Health. With this Certificate, the researchers cannot be forced to disclose information that may identify you, even by a court subpoena, in any federal, state, or local, civil, criminal, administrative, legislative, or other proceedings. The researchers will use the Certificate to resist any demands for information that would identify you, except as explained below.
The Certificate cannot be used to resist a demand for information from personnel of the United States Government that is used for auditing or evaluation of federally funded projects or for information that must be disclosed in order to meet the requirements of the federal Food and Drug Administration (FDA).

You should understand that a Certificate of Confidentiality does not prevent you or a member of your family from voluntarily releasing information about yourself or our involvement in this research. If an insurer, employer, or other person obtains your written consent to receive research information, then the researchers may not use the Certificate to withhold that information.

The Certificate of Confidentiality does not prevent researchers from disclosing voluntarily, without your consent, information that would identify you as a participant in the research project under the following circumstances: child abuse, intent to harm yourself or others, or if you have reportable communicable disease that state or federal law requires us to report such as Tuberculosis, HIV infection, or Syphilis.

In the event of any publication or presentation resulting from the research, no personally identifiable information will be shared. Your de-identified information may be used in the future for other research. Your de-identified data will be shared with the National Institute of Child Health and Human Development on the Data and Specimen Hub which is a resource for investigators to share de-identified research data from studies.

Some of these records could contain information that personally identifies you. Reasonable efforts will be made to keep the personal information in your research record private. However, absolute confidentiality cannot be guaranteed.

A description of this clinical trial will be available on http://www.ClinicalTrials.gov, as required by U.S. Law. This Web site will not include information that can identify you. At most, the Web site will include a summary of the results. You can search this Web site at any time.

Electronic Medical Records and Research Results

What is an Electronic Medical Record?
An Electronic Medical Record (EMR) is an electronic version of the record of your care within a health system. An EMR is simply a computerized version of a paper medical record.

If you are receiving care or have received care within HMC (outpatient or inpatient) and are participating in a HMC/PSU research study, results of research-related procedures (i.e. laboratory tests, imaging studies and clinical procedures) may be placed in your existing EMR maintained by HMC.

If you have never received care within HMC and are participating in a HMC/PSU research study that uses HMC services, an EMR will be created for you for the purpose of maintaining any results of procedures performed as part of this research study. The creation of this EMR is required for your participation in this study. In order to create your EMR, the study team will need to obtain basic information about you that would be similar to the information you would provide the first time you visit a hospital or medical facility (i.e. your name, the name of your primary doctor, the type of insurance you have). Results of research procedures performed as part of your participation in the study (i.e. laboratory tests, imaging studies and clinical procedures) may be placed in this EMR.

Once placed in your EMR, these results are accessible to appropriate HMC workforce members that are not part of the research team. Information within your EMR may also be shared with others who are determined by HMC to be appropriate to have access to your EMR (e.g. health insurance company, disability provider, etc).

What information about me may be collected, used or shared with others?

- Name
- Address
- Phone number, cell phone number, work number
- Email address
- Social Security number
- Medical Record number
- Date of birth
- Past, present, and future medical records
- New health information from tests, procedures, visits, interviews, or forms filled out as part of this research study. Any information from study visits, interviews or forms completed by you as part of the study will only be shared with the study team, and not to other entities without your prior authorization and approval.

Why is my information being used?

Your information is used by the research team to contact you during the study. Your information and results of tests and procedures are used to:

- do the research
- oversee the research
- to see if the research was done right.

Who may use and share information about me?

The following people/groups may see, use, and share your identifiable health information:

- HMC/PSU research staff involved in this study
- The University of Pennsylvania Institutional Review Board, a group of people who review the research study to protect subjects' rights and welfare
- The University of Pennsylvania Office of Regulatory Affairs
- The HMC/PSU Human Subjects Protection Office
- The HMC/PSU Research Quality Assurance Office
- Non-research staff within HMC/PSU who need this information to do their jobs (such as for treatment, payment (billing), or health care operations)
- Federal and state agencies (such as the U.S. Food and Drug Administration, the Office for Human Research Protections, the Department of Health and Human Services, the National Institutes of Health, and other U.S. or foreign government bodies that oversee or review research)
- Other researchers and medical centers that are part of this study and their IRBs
- The Data Coordinating Center at Yale University
- Reproductive Medicine Network of the The Eunice Kennedy Shriver National Institute of Child Health and Human Development
- A group that oversees the data (study information) and safety of this research
- Organizations that provide independent accreditation and oversight of hospitals and research
- Public health and safety authorities (for example, if we learn information that could mean harm to you or others, we may need to report this, as required by law)

These groups may also review and/or copy your original PSU/HMC records while looking at the results of the research study. It is possible that some of the other people/groups who receive your health information may not be required by Federal privacy laws to protect your information. We share your information only when we must, and we ask anyone who receives it from us to protect your privacy.

You have the right to see and get a copy of your health information that is used or shared for treatment or for payment. However, you may not be allowed to see or copy certain health information that is a part of this research study. This is only for the period of the study. You will be allowed to see that information when the entire research study is complete.

How long may HMC/PSU use or disclose my personal health information?

Your authorization for use of your personal health information for this specific study does not expire.

Your information may be held in a research database. However, HMC/PSU may not re-use or re-disclose information collected in this study for a purpose other than this study unless:

- You have given written authorization
- The University of Pennsylvania's Institutional Review Board grants permission
- As permitted by law

Can I change my mind about giving permission for use of my information?

You have the right to withdraw your permission for us to use or share your health information for this research study. If you want to withdraw your permission, you must notify the person in charge of this research study in writing using the address on the front of this form. Once permission is withdrawn, you cannot continue to take part in the study.

If you withdraw your permission, we will stop collecting health information about you for this study; we may continue to use and share your health information that we already have if it is necessary for safety and scientific soundness of the research study; and we will not be able to take back information that has already been used or shared with others.

What if I decide not to give permission to use and give out my health information?

You have the right to refuse to sign this form that allows us to use and share your health information for research; however, if you don't sign it, you will not be able to take part in this research study. Please refer to above information regarding other options available to you other than participating in this study.

Who can I call with questions, complaints or if I'm concerned about my rights as a research subject?

If you have questions, concerns or complaints regarding your participation in this research study or if you have any questions about your rights as a research subject, you should speak with the Principal Investigator listed on page one of this form. If a member of the research team cannot be reached or you want to talk to someone other than those working on the study, you may contact the Office of Regulatory Affairs at the University of Pennsylvania by calling (215) 898-2614. The Office of Regulatory Affairs at the University of Pennsylvania is the central human subjects protection oversight body for this study.

When you sign this form, you are agreeing to take part in this research study. This means that you have read the consent form, your questions have been answered, and you have decided to volunteer. Your signature also means that you are permitting the HMC/PSU to use your personal health information collected about you for research purposes within our institution. You are also allowing the HMC/PSU to disclose that personal health information to outside organizations or people involved with the operations of this study.

A copy of this consent and HIPAA authorization form will be given to you.

_________________________ ______________________________________

Name of Subject (Please Print) **Signature of Subject** **Date**

____

Name of Person Obtaining Consent (Please Print) **Signature** **Date**

Optional part(s) of the study

In addition to the main part of the research study, there are other parts of the research in which you may participate. You can be in the main part of the research without agreeing to be in some or all of these optional parts. Each optional section is listed below.

Optional Storage of Blood & Urine Reserve for Future Research

As part of an optional study, we want to obtain a sample of your blood and urine to be indefinitely stored by the RMN for future use. If you agree, your blood and urine will be collected at each visit. Approximately 2 tablespoons of blood will be collected and a urine sample. The sample will be labeled with your study identification number, visit name and visit date. The samples will be stored in a locked laboratory at the HMC/PSU until the RMN has decided on further testing to be done to the specimens. When the RMN has decided to do further testing, your sample will be shipped to the designated laboratory as decided by the RMN. If there is any remaining specimen after the testing is completed, your sample will then be shipped to the RMN Biologic Repository for long term storage. The samples will be tested for hormones and other substances in your blood and urine. These samples can be used for other research in the future after this study is over and also be shared with other investigators who collaborate with the RMN. Once your blood has left the HMC/PSU, there is no way to have it returned or withdrawn for testing. You will not be able to withdraw your permission for the use of your blood sample for future use as this time.

- These future studies may be helpful in understanding obesity and unexplained infertility.
- It is unlikely that these studies will have a direct benefit to you.

- The results of these tests will not have an effect on your care.
- Neither your doctor nor you will receive results of these future research tests, nor will the results be put in your health record.
- It is possible that your blood/urine might be used to develop products or tests that could be patented and licensed. There are no plans to provide financial compensation to you should this occur. If you have any questions, you should contact a member of the research team.

You should ***initial*** below to indicate your preference for the collection of your blood and urine reserve sample for ancillary studies in the future.

________ I **give my permission** for my blood and urine reserve sample to be collected and stored for future testing.

________ I **decline my permission** for my blood and urine reserve sample to be collected and stored for future testing.

Optional Study Whole Blood Collection

As part of an optional study, we would like to obtain, prior to you starting medication, a sample of your whole blood for DNA testing and to measure other substances in the blood. If you agree, less than 2 tablespoons of blood will collected at the randomization visit. The researchers would like to use your DNA to identify certain sequences of key genes (called polymorphisms) that may predict a response to the medications used in this study. If genes are identified, which are related to the medications used in the study, the sample may also be used in the development of diagnostic or prognostic testes to identify those who will or will not respond to these medications. We also may use this DNA to identify genes that cause infertility. There is no normal or abnormal result produced by the DNA and the researchers will not use the DNA to try to see if you have any genetic diseases or conditions. If you agree to allow us to collect and store a DNA sample from you for future use, your sample will be labeled with a bar code label and a unique identifier. These samples will be stored in in a locked laboratory at the HMC/PSU until shipment to the laboratory analyzing the DNA. The laboratory will be designated by the RMN. Once the DNA has been analyzed, and if there is leftover specimen, your sample will be shipped to the RMN Biologic Repository where it will be stored indefinitely or until the sample is exhausted by the RMN. These samples can be used for other research in the future after the study is over and also be shared with other investigators who collaborate with the RMN. Once your blood has left the HMC/PSU, there is no way to have it returned or withdrawn for testing. You will not be able to withdraw your permission for the use of your blood sample for future use as this time.

- The testing of DNA may provide additional information that will be helpful in understanding the medications used in this trial and the effects on infertility.
- It is unlikely that these studies will have a direct benefit to you.
- The results of these tests will not have an effect on your care.
- Neither your doctor nor you will receive results of these tests, nor will the results be put in your health record.

You should ***initial*** below to indicate your preference for the collection of your DNA sample:

________ I **give my permission** for my DNA sample to be collected and tested under the study.

________ I **decline my permission** for my DNA to be collected and tested under the study.

Optional Study Saliva for Biomarkers of Stress Collection

As part of an optional study, we would like to obtain a sample of your saliva to test for biomarkers of stress such as amylase and cortisol. Cortisol and amylase arestress hormones found in saliva. If you agree, a sample of your saliva will be collected by placing a cotton swab under your tongue for 60-120 seconds and then placed in a tube and sealed for storage in a freezer until testing. If you agree to allow us to collect your saliva, your sample will be labeled with a bar code label and a unique identifier. These samples will be stored in a locked laboratory at the HMC/PSU, until shipment to the laboratory analyzing the saliva. The laboratory will be designated by the RMN. Once the saliva has been analyzed, and if there is leftover sample, your sample will be shipped to the RMN Biologic Repository where it will be stored indefinitely or until the sample is exhausted by the RMN. These samples can be used for other research in the future after the study is over and also be shared with other investigators who collaborate with the RMN. Once your saliva has left the HMC/PSU, there is no way to have it returned or withdrawn for testing. You will not be able to withdraw your permission for the use of your saliva for future use as this time.

- The testing of saliva may provide additional information that will be helpful in understanding the factors of stress in infertility.
- It is unlikely that these studies will have a direct benefit to you.
- The results of these tests will not have an effect on your care.
- Neither your doctor nor you will receive results of these tests, nor will the results be put in your health record.

You should ***initial*** below to indicate your preference for the collection of your saliva sample:

________ I **give my permission** for my saliva sample to be collected and tested under the study.

________ I **decline my permission** for my saliva to be collected and tested under the study.

Optional Blood and Urine for RMN Biologic Repository

As part of an optional study, we are obtaining a sample of your blood to be stored in a Biologic Repository by the Reproductive Medicine Network for future use. If you agree, a urine sample and approximately 2 tablespoons of your blood will be collected at the randomization visit and shipped to the RMN Biologic Repository (a central location). Your sample will be tested for DNA and to measure other substances in your blood. If you agree to allow us to collect and store a blood sample from you for future use in the repository, your sample will be labeled with a bar code label and unique identifier. These samples will be stored in a locked laboratory at the HMC/PSU until shipment to the repository. If you consent to the collection of your blood for the repository, it will be kept indefinitely or until the sample is exhausted by the Reproductive Medicine Network. Once your blood has left the HMC/PSU, there is no way to have it returned or withdrawn for testing. You will not be able to withdraw your permission for the use of your blood sample for future use as this time.

We are also collecting blood and urine as a part of this study. If you agree, we would like to retain any leftovers after their use in the study for future use, including measuring other substances from your blood and urine. If you agree to let us retain these samples they will be stored in a locked laboratory

at the HMC/PSU until shipment to the repository. If you consent to the retention of these samples blood and urine for the repository, they will be kept indefinitely or until the sample is exhausted by the Reproductive Medicine Network. Once your blood and urine has left the HMC/PSU, there is no way to have it returned or withdrawn for testing. You will not be able to withdraw your permission for the use of your blood and urine sample for future use at this time.

- The testing of your blood and urine may provide additional information that will be helpful in understanding the medications used in this trial, obesity and unexplained infertility.
- It is unlikely that these studies will have a direct benefit to you.
- The results of these tests will not have an effect on your care.
- Neither your doctor nor you will receive results of these tests, nor will the results be put in your health record.
- It is possible that your blood/urine might be used to develop products or tests that could be patented and licensed. There are no plans to provide financial compensation to you should this occur. If you have any questions, you should contact a member of the research team.

You should ***initial*** below to indicate your preference for the collection of your blood and urine sample for the RMN Biologic Repository:

______ I **give my permission** for my blood and urine sample to be collected and sent to the repository for future testing.
______ I **decline my permission** for my blood and urine sample to be collected and sent to the repository for future testing.

You should ***initial*** below to indicate your preference for the storage of your leftover blood and urine sample for the RMN Biologic Repository:

______ **I give my permission** for my leftover blood and urine sample(s) to be retained and sent to the repository for future testing.

______ **I decline my permission** my leftover blood and urine sample(s) to be retained and sent to the repository for future testing.

Signature of Person Giving Informed Consent

Signature of Subject

By signing below, you indicate that you have read the information written above and have indicated your choices for the optional part(s) of the research study.

_________________________ _____________________________________

Name of Subject (Please Print) **Signature of Subject** **Date**

Signature of Person Obtaining Informed Consent

Your signature below means that you have explained the optional part(s) to the research to the subject or subject representative and have answered any questions he/she has about the research.

___________________________	____________________________________	
**Name of Person Obtaining Consent** (Please Print)	**Signature**	**Date**

16 Human Subjects/Informed Consent/Male

COMBINED INFORMED CONSENT AND
HIPAA AUTHORIZATION FORM
Penn State College of Medicine
The Milton S. Hershey Medical Center

**Protocol Title:**	Improving Reproductive Fitness with Pretreatment with Lifestyle Modification in Obese Women with Unexplained Infertility: FIT-PLESE
**Principal Investigator:**	Richard Legro, M.D.<br>Obstetrics & Gynecology Department, Division of Reproductive Endocrinology<br>500 University Drive, Room C3604, MC H103<br>Hershey, PA 17033
**Emergency Contact:**	Weekdays: 8:00 a.m. to 5:00 p.m. (717) 531-8478.<br>After hours call (717) 531-8521 and ask for the Ob-Gyn doctor on 24-hour call.

Why am I being asked to volunteer?

You are being invited to participate in a research study. Your participation is voluntary which means you can choose whether or not you want to participate. If you choose not to participate, there will be no loss of benefits to which you are otherwise entitled. Before you can make your decision, you will need to know what the study is about, the possible risks and benefits of being in this study, and what you will have to do in this study. The research team is going to talk to you about the research study, and they will give you this consent form to read. You may also decide to discuss it with your family, friends, or family doctor. You may find some of the medical language difficult to understand. Please ask the study doctor and/or the research team about this form. If you decide to participate, you will be asked to sign this form.

What is the purpose of this research study?

We are asking you to be in this research because you and your partner/spouse have a diagnosis of unexplained infertility and are seeking pregnancy and your partner is overweight.

This research is being done to find out the effects of lifestyle modification in obese women with unexplained infertility. Phase 1 of the study is 16 weeks and includes one of two types of lifestyle modifications for your partner, either increased physical activity by increasing steps per day OR a calorie restricted diet using meal replacements, use of a weight loss medication called Orlistat

(Alli) and increased physical activity. Orlistat 60mg is a United States Food and Drug Administration (FDA) approved medication to be taken with meals and works by reducing the absorption of fat in a meal.

After completion of Phase 1 lifestyle modification, your partner/spouse will move to Phase 2 of the study. This will involve 3 cycles of infertility treatment involving ovarian stimulation with an oral medication (clomiphene citrate) followed by triggered ovulation using hCG injection and single intrauterine insemination. Clomiphene Citrate is approved by the FDA for the treatment of infertility. hCG, a combination medication of Follicle Stimulating Hormone and Luteinizing Hormone. It is an injectable medicine to be used to trigger ovulation and is also FDA approved.

How long will I be in the study?

If you agree to take part, it will take you up to 8 months to complete this research study. This includes a screening visit for semen analysis and then up to 3 cycles of inseminations for your partner/spouse where you will need to provide semen specimens. You will be asked to return to the research site 3 times. Each visit will take as long as you need for collection of semen for analysis and insemination. Your partner would not be able to have the intrauterine insemination without these specimens.

How many subjects will be in the study?

Approximately 380 women and their partner/spouse will take part in this research study nationwide as part of the Reproductive Medicine Network (RMN).

What am I being asked to do?

At initial contact either by email, telephone, or in clinic, your partner/spouse will be screened with a brief eligibility questionnaire to determine if she qualifies to take part in this research. If she is determined to be eligible, you and your partner/spouse will be scheduled for a screening visit. This consent form will be reviewed by you and the study coordinator. The purpose of the study, all procedures involved, the risks, and the time commitment related to this study will be discussed. Once all questions have been answered, you will sign this consent form. A copy of this signed consent will be given to you for your records. Your partner/spouse will also sign a separate informed consent for participation.

Medical Assessment & Questionnaires

During the screening visit your height, weight, vital signs (blood pressure and pulse) and neck, hip and abdominal circumference will be collected and your BMI will be calculated. You will be required to complete a medical history questionnaire. This questionnaire will ask you questions about your medical health, infertility history, and demographic information. “We will also give you questionnaires concerning your sexual function, feelings of depression, daily activities, sleep habits, sunscreen usage, and past and current dietary history. You will be free to skip any questions that you would prefer not to answer or that make you uncomfortable.

When your partner is randomized, you will use your partner’s Aria Scale to have your weight recorded. You will be required to weigh yourself once at the time your partner is randomized. This Wi-Fi smart scale will send your data to the secure database for review. If you are unable

to use the Aria scale wirelessly, you will be required to contact the study coordinator via email, text or phone call to report your result.

When your partner has her End of Study visit, you will be required to use the Aria Scale again to have your weight recorded. You will also repeat the quality of life questionnaires as you did during the screening visit, excluding the DHQII. If your partner is pregnant, the FertiQOL questionnaire will be skipped.

Collection of Semen Sample

If you agree to take part in this research study, you will be asked to provide a semen sample if you have not given one within the past 12 months. The study coordinator will schedule you for an appointment in the andrology laboratory. Instructions will be given to you prior to the collection appointment. For this semen analysis test, it is important to not have sex or masturbate for 48 hours prior to collecting your samples.

The following are the instructions you will follow to collect your sample. There is a private room near the lab where the specimen can be collected.

1. Wash your hands and genitals in the normal way and dry thoroughly.
2. Use the cup provided by the study coordinator or the lab. Do not open the container until you are ready to produce the sample.
3. Collect the sample by masturbation, putting the entire specimen directly into the cup.
4. Do not use lubricants or condoms, as these are harmful to sperm.
5. Seal the cup immediately with the lid.
6. Write your name on the label of the cup. Fill out the bottom of the lab form. Return the specimen and form to the laboratory staff.

Once you have completed your collection, a semen analysis will be performed to determine the concentration of sperm, motility (moving) and morphology (appearance). Once your specimen has been tested, the study coordinator will be given your results. Your results will be reviewed with principal investigator of the study. You will be notified of your results. If there are more than 5 million motile (moving) sperm in the ejaculate, your partner/spouse will be eligible to participate in the main study. If the results of your semen analysis are abnormal, you will be provided with the information and referred to your physician for further follow-up. If the number of motile sperm in an ejaculate is less than 5 million, your partner/spouse will be ineligible to participate in the main study at this time. If you have previously had a semen analysis test done within the past 12 months, we will ask you to sign a medical release form so we can obtain your results.

Trigger injection & Insemination

You will be asked to assist in the giving of an injection of the hCG trigger medication to your partner/spouse. The proper technique of the injection will be taught to you and your partner/spouse by the study coordinator. You may decline to assist in the injections and remain in the study, if your partner/spouse is going to self-administer the injections.

As part of the inclusion criteria for participation in this study, inseminations (placing your sperm into your partner's/spouse's uterus) of your partner must take place at least once each cycle for up to 3 cycles. Both you and your partner must agree to this requirement to participate in the study.

For the inseminations, the semen specimen will be washed to concentrate the sperm and remove other components of the ejaculate specimen. The sperm will then be used for the insemination of your partner/spouse.

Repository Semen, Blood and Urine Specimen Collection
If you consent to the optional part of the study, you will provide a urine sample and have approximately 2 tablespoons of blood drawn for future testing. No blood or urine will be collected if you have not consented to this option. A portion of your semen will also be collected and stored as part of the repository. Genetic testing may be performed on some of these samples. Additional information about these optional samples is included at the end of this consent form. These samples will only be collected if you have consented to the optional part of the study.

Saliva Collection for Testing for Biomarkers of Stress
If you have consented to the collection of saliva for testing for biomarkers of stress, such as cortisol or amylase, this will be collected at this time. Cortisol and amylase are stress hormones. This will be done by removing a cotton swab from the package and placing the swab under your tongue for approximately 60-120 seconds. Once the time is complete, the swab is inserted into the tube and sealed for storage in a freezer.

What are my responsibilities if I take part in this research?

If you take part in this research, your major responsibilities will include:

- Providing a semen sample for screening and for each insemination cycle up to 3 cycles
- Assisting in injections of hCG trigger medication to your partner/spouse
- Completing medical and quality of life questionnaires
- Provide blood, urine and saliva for optional portion of the study if you have consented to this part of the study.

What are the possible risks or discomforts?

By taking part in this study, you may experience the following risks:

Risks of standard venipuncture:
A slight pinch or pin prick, discomfort, black and blue mark at the site of puncture, small blood clot, infection or bleeding at the site and fainting during the procedure.

Risks of loss of Confidentiality:
There is a risk of loss of confidentiality if your medical information or your identity are obtained by someone other than the investigators, but precautions will be taken to prevent this from happening.

Risks of Genetic Testing:
A Federal law, called the Genetic Information Nondiscrimination Act (GINA), generally makes it illegal for health insurance companies, group health plans, and most employers to discriminate against you based on your genetic information.
This law generally will protect you in the following ways:

- Health insurance companies and group health plans may not request your genetic information that we get from this research.
- Health insurance companies and group health plans may not use your genetic information when making decisions regarding your eligibility or premiums.
- Employers with 15 or more employees may not use your genetic information that we get from this research when making a decision to hire, promote, or fire you or when setting the terms of your employment.

Be aware that this Federal law does not protect you or your family against genetic discrimination by companies that sell life insurance, disability insurance, or long-term care insurance. Also, GINA does not prohibit discrimination of individuals with a genetic disorder that has been diagnosed. However, in order to do everything possible to keep this from happening, the results of this test will NOT be given to anyone outside the study staff. This means that it will not be made available to you, your family members, your private physician, your employer, your insurance company or any other party as allowed by law.

What if new information becomes available about the study?

During the course of this study, we may find more information that could be important to you. This includes information that, once learned, might cause you to change your mind about being in the study. We will notify you as soon as possible if such information becomes available.

What are the possible benefits of the study?

There is no guarantee that you will benefit from this research. The possible benefits you may experience from this research study include:

- You will be provided with your semen analysis results. Any abnormalities will be assessed by the principal investigator and followed through by your physician. The results of your semen analysis will determine eligibility for your partner/spouse to participate in the main study.
- The study interventions and medications your partner/spouse is taking may help her to become pregnant.

What are the possible benefits to others?

The results of this research may guide the future of treatment of obesity for women with unexplained infertility by establishing effective lifestyle changes and weight loss prior to attempting pregnancy. This research may also help discover a better way to achieve pregnancy and a goal of a healthy pregnancy with live birth by the use of Clomiphene Citrate and intrauterine insemination for women with unexplained infertility.

What other choices do I have if I do not participate?

You do not have to take part in this study. Choosing not to participate will not have any effect on your clinical care.

The therapy offered in this research is available to you without taking part in this research study. Choosing not to participate will not affect your standard of care treatment.

Will I be paid for being in this study?

You will not receive any payment or compensation for being in this research study.

Will I have to pay for anything?

The study will provide the following:

- The semen analysis will be provided by the Reproductive Medicine Network at no cost to you.
- You and/or your insurance company will not be charged for the cost of any tests or procedures that are required as part of the research and are within the standard of care (what is normally done) for your condition.
- The research-related tests and procedures that will be provided at no cost to you include: semen analysis, blood work and urine collection for RMN Biologic Repository and saliva collection.

For costs of medical services for care you would receive even if you were not in this research study:

- You and/or your insurance company will be responsible for the cost of routine medications, tests and procedures that you would receive even if you were not in this research that are not mentioned above as covered.
- You and/or your insurance company will be billed for the costs of these routine tests and procedures in the usual manner.
- You will be responsible for any co-payments, co-insurance and deductibles that are standard for your insurance coverage. You will be responsible for any charges not reimbursed by your insurance company.
- Some insurance companies will not pay for routine costs for people taking part in research studies. Before deciding to be in this research you should check with your insurance company to find out what they will pay for.

If you have any questions about costs and insurance, ask the research study doctor or a member of the research team about putting you in touch with a financial counselor to determine exactly what the deductible and co-pay will be for you; this is highly variable depending on your type of insurance."

Who is paying for this research study?

The institution and investigators are receiving a grant from the National Institute of Health to support this research.

What happens if I am injured from being in the study?

It is possible that you could develop complications or injuries as a result of being in this research study. If you experience a side effect or injury and emergency medical treatment is required, seek treatment immediately at any medical facility. If you experience a side effect or injury and you believe that emergency treatment is not necessary, you should contact the principal

investigator listed on the first page of this consent form as soon as possible and he/she will arrange for medical treatment.

The Milton S. Hershey Medical Center (HMC)/Penn State College of Medicine (PSU) compensation for injury

- There are no plans for HMC/PSU to provide financial compensation or free medical treatment for research-related injury.
- If an injury occurs, medical treatment is available at the usual charge.
- Costs will be charged to your insurance carrier or to you.
- Some insurance companies may not cover costs associated with research injuries.
- If these costs are not covered by your insurance, they will be your responsibility.

When you sign this form you are not giving up any legal right to seek compensation for injury.

When is the Study over? Can I leave the Study before it ends?

This study is expected to end after all participants have completed all visits, and all information has been collected. This study may also be stopped at any time by your physician, the study Sponsor, or the Food and Drug Administration (FDA) without your consent because:

- The Primary Investigator feels it is necessary for your health or safety. Such an action would not require your consent, but you will be informed if such a decision is made and the reason for this decision.
- You have not followed study instructions.
- The Sponsor, the study Principal Investigator, or the Food and Drug Administration (FDA) has decided to stop the study.

If you decide to participate, you are free to leave the study at anytime. Withdrawal will not interfere with your future care. Your regular medical care at the facility will not be impacted in any way.

If you stop being in the research, already collected data may not be removed from the study database. You will be asked whether the investigator can collect medical information from your routine medical care. If you agree, this data will be handled the same as research data. If you withdraw completely from the research study, no further information will be collected and your participation will end. You may discontinue taking part at any time without penalty or loss of benefits to which you are otherwise entitled.

Who can see or use my information? How will my personal information be protected?

Efforts will be made to limit the use and sharing of your personal research information. In our research files at HMC/PSU we will include these identifiers:

- Study identification number
- Name & Initials
- Address
- Phone number, cell phone number, work number
- Email address
- Social Security number

- Medical Record number
- Date of birth
- Visit date

A list that matches your name with your code number will be kept in a locked file.

Your records that are used in the research at HMC/PSU will include your study identification number, your initials, and visit date and will be kept in a secured area in a locked file cabinet. Your samples collected for research purposes will be labeled with your study identification number, initials, and visit date and will be stored in a -80 degree freezer.

For research records sent to the Data Coordination Center at Yale University, you will be identified by your study identification number and study visit date. The research records are entered into an electronic password protected database. Only HMC/PSU and RMN/Yale University investigators as well as investigators approved by the RMN to use the data will have access to these de-identified electronic records. This data is managed by the RMN/Yale University Data Coordinating Center and will be kept indefinitely. Your blood specimens will not identify you and you cannot be linked to your specimen. The list that matches your name with your code number will be kept in a secured area in a locked file cabinet at HMC/PSU.

For specimens sent to the RMN Biologic Repository and the saliva collection, you will not be identified and you cannot be linked to your sample. The sample will be labeled with a bar code label. The sample will be stored in the Repository until exhausted by the RMN.

To help protect your privacy, a Certificate of Confidentiality will be obtained from the federal government. This Certificate means that the researchers cannot be forced (for example by court subpoena) to share information that may identify you in any federal, state, or local civil, criminal, administrative, legislative, or other proceedings. The researchers will use the Certificate to resist any demands for information that would identify you, except as explained below.

The Certificate cannot be used to resist a demand for information from personnel of the U.S. government that is used for checking or evaluation of federally funded projects or for information that must be disclosed in order to meet the requirements of the federal Food and Drug Administration (FDA).

The Certificate, however, does not prevent you or a member of your family from voluntarily releasing information about yourself or your involvement in this research. If an insurer, employer, or other person obtains your written consent to receive research information, then the researchers may not use the Certificate to withhold that information. This means that you and your family should actively protect your own privacy.

In the event of any publication or presentation resulting from the research, no personally identifiable information will be shared. Your de-identified information may be used in the future for other research. Your de-identified data will be shared with the National Institute of Child Health and Human Development on the Data and Specimen Hub which is a resource for investigators to share de-identified research data from studies.

A copy of this consent form will go in to your medical record. This will allow the doctors caring for you to know what study medications or tests you may be receiving as a part of the study and know how to take care of you if you have other health problems or needs during the study.

We will do our best to make sure that the personal information obtained during the course of this research study will be kept private. However, we cannot guarantee total privacy. Your personal information may be given out if required by law. If information from this study is published or presented at scientific meetings, your name and other personal information will not be used. If this study is being overseen by the Food and Drug Administration (FDA), they may review your research records.

If you test positive for reportable infectious diseases, by law we have to report the infection to the PA Department of Health. We would report your name, gender, racial/ethnic background, and the month and year you were born.

Electronic Medical Records and Research Results

Results of some of the research-related tests including but not limited to your screening semen analysis and semen analysis for insemination information will be kept in your electronic medical record.

What is an Electronic Medical Record?
An Electronic Medical Record (EMR) is an electronic version of the record of your care within a health system. An EMR is simply a computerized version of a paper medical record.

If you are receiving care or have received care within the HMC (outpatient or inpatient) and are participating in a HMC/PSU research study, results of research-related procedures (i.e. laboratory tests, imaging studies and clinical procedures) may be placed in your existing EMR maintained by HMC.

If you have never received care within HMC and are participating in a HMC/PSU research study that uses HMC services, an EMR will be created for you for the purpose of maintaining any results of procedures performed as part of this research study. The creation of this EMR is required for your participation in this study. In order to create your EMR, the study team will need to obtain basic information about you that would be similar to the information you would provide the first time you visit a hospital or medical facility (i.e. your name, the name of your primary doctor, the type of insurance you have). Results of research procedures performed as part of your participation in the study (i.e. laboratory tests, imaging studies and clinical procedures) may be placed in this EMR.

Once placed in your EMR, these results are accessible to appropriate HMC/PSU workforce members that are not part of the research team. Information within your EMR may also be shared with others who are determined by HMC/PSU to be appropriate to have access to your EMR (e.g. health insurance company, disability provider, etc.).

What information about me may be collected, used or shared with others?

- Name

- Address
- Phone number, cell phone number, work number
- Email address
- Social Security number
- Medical Record number
- Date of birth
- Past, present, and future medical records
- New health information from tests, procedures, visits, interviews, or forms filled out as part of this research study. Any information from study visits, interviews or forms completed by you as part of the study will only be shared with the study team, and not to other entities without your prior authorization and approval.

Why is my information being used?

Your information is used by the research team to contact you during the study. Your information and results of tests and procedures are used to:

- do the research
- oversee the research
- see if the research was done right.

Who may use and share information about me?

The following people/groups may see, use, and share your identifiable health information:

- HMC/PSU research staff involved in this study
- The University of Pennsylvania Institutional Review Board, a group of people who review the research study to protect subjects' rights and welfare
- The University of Pennsylvania Office of Regulatory Affairs
- The HMC/PSU Human Subjects Protection Office
- The HMC/PSU Research Quality Assurance Office
- Non-research staff within HMC/PSU who need this information to do their jobs (such as for treatment, payment (billing), or health care operations)
- Federal and state agencies (such as the U.S. Food and Drug Administration, the Office for Human Research Protections, the Department of Health and Human Services, the National Institutes of Health, and other U.S. or foreign government bodies that oversee or review research)
- Other researchers and medical centers that are part of this study and their IRBs
- The Data Coordinating Center at Yale University
- Reproductive Medicine Network of the Eunice Kennedy Shriver National Institute of Child Health and Human Development
- A group that oversees the data (study information) and safety of this research

- Organizations that provide independent accreditation and oversight of hospitals and research
- Public health and safety authorities (for example, if we learn information that could mean harm to you or others, we may need to report this, as required by law)

These groups may also review and/or copy your original PSU/HMC records while looking at the results of the research study. It is possible that some of the other people/groups who receive your health information may not be required by Federal privacy laws to protect your information. We share your information only when we must, and we ask anyone who receives it from us to protect your privacy

You have the right to see and get a copy of your health information that is used or shared for treatment or for payment. However, you may not be allowed to see or copy certain health information that is a part of this research study. This is only for the period of the study. You will be allowed to see that information when the entire research study is complete.

How long may the HMC/PSU use or disclose my personal health information?

Your authorization for use of your personal health information for this specific study does not expire.

Your information may be held in a research database. However, HMC/PSU may not re-use or re-disclose information collected in this study for a purpose other than this study unless:

- You have given written authorization
- The University of Pennsylvania's Institutional Review Board grants permission
- As permitted by law

Can I change my mind about giving permission for use of my information?

You have the right to withdraw your permission for us to use or share your health information for this research study. If you want to withdraw your permission, you must notify the person in charge of this research study in writing using the address on the front of this form. Once permission is withdrawn, you cannot continue to take part in the study.

If you withdraw your permission, we will stop collecting health information about you for this study; we may continue to use and share your health information that we already have if it is necessary for safety and scientific soundness of the research study; and we will not be able to take back information that has already been used or shared with others.

What if I decide not to give permission to use and give out my health information?

You have the right to refuse to sign this form that allows us to use and share your health information for research; however, if you don't sign it, you will not be able to take part in this research study.
Please refer to above information regarding other options available to you other than participating in this study.

Who can I call with questions, complaints or if I'm concerned about my rights as a research subject?

If you have questions, concerns or complaints regarding your participation in this research study or if you have any questions about your rights as a research subject, you should speak with the Principal Investigator listed on page one of this form. If a member of the research team cannot be reached or you want to talk to someone other than those working on the study, you may contact the Office of Regulatory Affairs at the University of Pennsylvania by calling (215) 898-2614. The Office of Regulatory Affairs at the University of Pennsylvania is the central human subjects protection oversight body for this study.

When you sign this form, you are agreeing to take part in this research study. This means that you have read the consent form, your questions have been answered, and you have decided to volunteer. Your signature also means that you are permitting HMC/PSU to use your personal health information collected about you for research purposes within our institution. You are also allowing HMC/PSU to disclose that personal health information to outside organizations or people involved with the operations of this study.

A copy of this consent form will be given to you.

_________________________ _____________________________________

Name of Subject (Please Print) **Signature of Subject** **Date**

____

Name of Person Obtaining Consent (Please Print) **Signature** **Date**

Optional part(s) of the study

In addition to the main part of the research study, there is another part of the research. You can be in the main part of the research without agreeing to be in this optional part.

Optional Blood and Urine for RMN Biologic Repository

As part of an optional study, we are obtaining a sample of your blood to be stored in a Biologic Repository by the Reproductive Medicine Network for future use. If you agree, a urine sample and approximately 2 tablespoons of your blood will be collected at the randomization visit and shipped to the RMN Biologic Repository (a central location). Your sample will be tested for DNA and to measure other substances in your blood. If you agree to allow us to collect and store a blood sample from you for future use in the repository, your sample will be labeled with a bar code label and unique identifier. If you consent to the collection of your blood for the repository, it will be kept indefinitely or until the

sample is exhausted by the Reproductive Medicine Network. These samples can be used for other research in the future after the study is over and also be shared with other investigators who collaborate with the RMN. Once your blood has left the HMC/PSU, there is no way to have it returned or withdrawn for testing. You will not be able to withdraw your permission for the use of your blood sample for future use as this time. No blood or urine will be collected if you have not agreed to this option.

- These future studies may be helpful in understanding testicular physiology and unexplained infertility.
- It is unlikely that these studies will have a direct benefit to you.
- The results of these tests will not have an effect on your care.
- Neither your doctor nor you will receive results of these tests, nor will the results be put in your health record.
- It is possible that your blood/urine might be used to develop products or tests that could be patented and licensed. There are no plans to provide financial compensation to you should this occur. If you have any questions, you should contact a member of the research team.

You should ***initial*** below to indicate your preference for the collection of your blood and urine sample for the RMN Biologic Repository:

______ I **give my permission** for my blood and urine sample to be collected and sent to the repository for future testing.

______ I **decline my permission** for my blood and urine sample to be collected and sent to the repository for future testing.

Optional Study Saliva for Biomarkers of Stress Collection

As part of an optional study, we would like to obtain a sample of your saliva for testing for biomarkers of stress, including cortisol and amylase. Cortisol and amylaseare stress hormones found in saliva. If you agree, a sample of your saliva will be collected by placing a cotton swab under your tongue for 60-120 seconds and then placed in a tube and sealed for storage in a freezer until testing. If you agree to allow us to collect your saliva, your sample will be labeled with a bar code label and a unique identifier. These samples will be stored in Dr. Legro's locked laboratory, Room C3611 until shipment to the laboratory analyzing the saliva. The laboratory will be designated by the RMN. Once the saliva has been analyzed, and if there is leftover sample, your sample will be shipped to the RMN Biologic Repository where it will be stored indefinitely or until the sample is exhausted by the RMN. These samples can be used for other research in the future after the study is over and also be shared with other investigators who collaborate with the RMN. Once your saliva has left the Hershey Medical Center, there is no way to have it returned or withdrawn for testing. You will not be able to withdraw your permission for the use of your saliva for future use as this time.

- The testing of saliva may provide additional information that will be helpful in understanding the factors of stress in infertility.
- It is unlikely that these studies will have a direct benefit to you.
- The results of these tests will not have an effect on your care.

- Neither your doctor nor you will receive results of these tests, nor will the results be put in your health record.

You should ***initial*** below to indicate your preference for the collection of your saliva sample:

________ I **give my permission** for my saliva sample to be collected and tested under the study.

________ I **decline my permission** for my saliva to be collected and tested under the study.

Optional Storage of Semen for Future Use in Research

As part of an optional study, we want to obtain a sample of your semen to be stored by the RMN for future use in the RMN Biologic Repository. If you agree, your semen sample will be collected at the screening visit after informed consent. If you are collecting a semen sample at the screening visit for analysis as part of the screening requirement, a portion of that sample can be used for this optional collection. A separate collection would not be needed in this instance. If you have a semen analysis on file that meets the eligibility requirements, and do not need to collect for eligibility, then a semen sample would need to be collected for this optional part of the study. The sample will be stored at the HMC/PSU until shipment to the RMN Biologic Repository. Your sample will be tested for the chemicals that make up all of your genes and contain your genetic information. These samples can be used for other research in the future after this study is over. Your sample will not be labeled with any of your personal information, such as your name or a code number. The sample for the repository is labeled with a bar code and cannot be directly linked back to you. They will be available for use in future research studies indefinitely and cannot be removed due to the inability to identify them.

- These future studies may be helpful in understanding testicular physiology and unexplained infertility.
- It is unlikely that these studies will have a direct benefit to you.
- The results of these tests will not have an effect on your care.
- Neither your doctor nor you will receive results of these future research tests, nor will the results be put in your health record.
- It is possible that your semen sample might be used to develop products or tests that could be patented and licensed. There are no plans to provide financial compensation to you should this occur. If you have any questions, you should contact a member of the research team.

You should ***initial*** below to indicate your preference for the collection of your semen sample for the repository:

________ I **give my permission** for my semen sample to be collected and sent to the repository for future testing.

________ I **decline my permission** for my semen sample to be collected and sent to the repository for future testing.

Signature of Person Giving Informed Consent

Signature of Subject

By signing below, you indicate that you have read the information written above and have indicated your choices for the optional part(s) of the research study.

__________________________ _______________________________________

Name of Subject (Please Print) **Signature of Subject** **Date**

Signature of Person Obtaining Informed Consent

Your signature below means that you have explained the optional part(s) to the research to the subject or subject representative and have answered any questions he/she has about the research.

___________________________ _______________________________________

Name of Person Obtaining Consent (Please Print) **Signature** **Date**

17 Appendix A: Risk Factors for Genetic Disorders

The following questions are designed to determine if you have an increased risk of having a baby with a genetic disorder. Sometimes genetic disorders occur even when there is no history of problems in your family. The following questions are related to you and your immediate family including mother, father, sister, brother, child, or grandparent. If you answer "yes" to any of the following questions and would like more information, please discuss this with your physician.

Questions	Yes	No	Unknown	N/A
1. If you conceive during this study, will you be 35 or older when your baby is due?				
2. If you are of Mediterranean or Asian descent, does anyone in your family have thalassemia? (a blood disorder that causes anemia)				
3. Is there a family history of neural tube defects?				
4. Have you had a child with a neural tube defect?				
5. Does anyone in your family have a history of congenital heart defects? (heart problems when they were born)				
6. Does anyone in your family have Down Syndrome?				
7. Have you ever had a child with Down Syndrome?				
8. If you are of Eastern European Jewish or French Canadian descent, does anyone in your family have a history of Tay-Sachs disease? (a disorder of the central nervous system)				
9. If you are of Eastern European Jewish descent, does anyone in your family have a history of Canavan disease? (a disorder of the central nervous system that leads to blindness and muscle weakness)				
10. If you are of African American descent, is there any history of sickle cell trait? (a type of anemia)				
11. If you are of African American descent, is there any history of sickle cell disease? (a type of anemia)				
12. Do you or anyone in your family have a history of hemophilia? (a disorder that causes bleeding)				
13. Do you or anyone in your family have a history of muscular dystrophy? (a neuromuscular disorder)				
14. Do you or anyone in your family have a history of cystic fibrosis? (a disorder that causes thick mucous in the lungs and other organs)				
15. Do you or anyone in your family have a history of Huntington's disease? (a degenerative brain disease)				
16. Do you or anyone in your family have a history of alpha-1 antitrypsin deficiency? (lack of a liver protein)				
17. Do you or anyone in your family have a history of mental retardation?				

17a. If yes to Question #17, was the person ever tested for fragile-x syndrome? (a condition which can cause mild to severe mental retardation)				
18. Do you or anyone in your family have a history of any other genetic disease, chromosomal disorder or birth defect?				
19. Do you have any metabolic disorders such as diabetes or phenylketonuria (PKU)? (a disorder which prevents the normal use of protein foods)				
20. Have you ever had 3 or more miscarriages in a row?				
21. Have you ever had a baby that was stillborn?				

Partner Family History

The following questions are designed to determine if you have an increased risk of having a baby with a genetic disorder. Sometimes genetic disorders occur even when there is no history of problems in your family. The following questions are related to you and your immediate family including mother, father, sister, brother, child, or grandparent. If you answer "yes" to any of the following questions and would like more information, please discuss this with your physician.

Questions	Yes	No	Unknown	N/A
1. If you are of Mediterranean or Asian descent, does anyone in your family have thalassemia? (a blood disorder that causes anemia)				
2. Is there a family history of neural tube defects?				
3. Have you had a child with a neural tube defect?				
4. Does anyone in your family have a history of congenital heart defects? (heart problems when they were born)				
5. Does anyone in your family have Down Syndrome?				
6. Have you ever had a child with Down Syndrome?				
7. If you are of Eastern European Jewish or French Canadian descent, does anyone in your family have a history of Tay-Sachs disease? (a disorder of the central nervous system)				
8. If you are of Eastern European Jewish descent, does anyone in your family have a history of Canavan disease? (a disorder of the central nervous system that leads to blindness and muscle weakness)				
9. If you are of African American descent, is there any history of sickle cell trait? (a type of anemia)				
10. If you are of African American descent, is there any history of sickle cell disease? (a type of anemia)				
11. Do you or anyone in your family have a history of hemophilia? (a disorder that causes bleeding)				
12. Do you or anyone in your family have a history of muscular dystrophy? (a neuromuscular disorder)				
13. Do you or anyone in your family have a history of cystic fibrosis? (a disorder that causes thick mucous in the lungs and other organs)				
14. Do you or anyone in your family have a history of Huntington's disease? (a degenerative brain disease)				
15. Do you or anyone in your family have a history of alpha-1 antitrypsin deficiency? (lack of a liver protein)				
16. Do you or anyone in your family have a history of mental retardation?				
16a. If yes to Question #17, was the person ever tested for fragile-x syndrome? (a condition which can cause mild to severe mental retardation)				

17. Do you or anyone in your family have a history of any other genetic disease, chromosomal disorder or birth defect?				
18. Do you have any metabolic disorders such as diabetes or phenylketonuria (PKU)?<br>(a disorder which prevents the normal use of protein foods)				

18 Appendix B: List of common medications excluded or requiring wash-out period

Excluded Medications

A patient will be excluded from study if they are taking any medication that should not be discontinued and this medication would affect reproductive function or metabolism, or would interact with either study medication.

Hormonal Medications Requiring 1 month wash-out:

Progestins (Oral or Cyclic)

- medroxyprogesterone acetate (Provera, Cycrin, Amen, Curretab)
- megestrol (Megase)
- norethindrone (Aygestin)
- progesterone gel (Crinone)
- Micronized progesterone (Prometrium)

Hormonal Medications Requiring 2 month Wash-Out Period:

GnRH Agonists/Antagonists

- Leuprolide (Lupron)
- nafarelin (Synarel)
- buserelin
- gosarelin (Zoladex)
- ganarelix (Antagon)
- cetrorelix (Cetrotide)

Gonadotropins

- Pergonal
- Repronex
- Follistim
- Gonal-F
- Fertinex
- Metrodin

Injectable Contraceptives

- medroxyprogesterone acetate (Depo Provera)

Oral Contraceptives

- Any Brand

Continuous Progestins (Not including cyclic)

- Any Brand

Other Medications Requiring 2 month Wash-out Period:

Somatostatin

- octreotide (Sandostatin)
- lanreaotide

Anti-Acne

- isotretinoin (Accutane)

Anti-androgens

- cyproterone (Cyprostat)
- spironolactone (Aldactone)
- flutamide (Eulexin)
- finasteride (Proscar, Propecia)

Anti-diabetic

Insulin

Gastric inhibitors

- acarbose (Precose)

Thiazolidinediones

- Rosiglitazone (Avandia)
- Pioglitazone (Actos)

Sulfonylureas/Beta cell stimulators

acetahexamide (Dymelor)
chlorpropamide (Diabinese)
tolazamide (Tolinase)
tolbutamide (Orinase)
glimepiride (Amaryl)
glipizide (Glucotrol)
glyburide (DiaBeta Micronase)

Biguanides

metformin (Glucophage)

Incretins : GLP-1 Analogues/DPP-IV inhibitors

sitagliptin (Januvia)
vidagliptin (Glavus)
exenatide (Byetta)
liraglutide (Victoza)

Amylin Analogue

Pramlintide (Symlin)

Anti-obesity

diazoxide (Proglycem)
orlistat (Xenical
diethylpropion (Tenuate)
phendimetrazine (Bontril)
phentermine (Adipex-P, Fastin, Ionamin)
lorcarserin (Belviq)
Phentermine/Topiramate (Osymia)

Other drugs with weight loss as a side effect

Buproprion (Wellbutrin, Zyban)
Topiramate (Topamax)
Desfenlafaxine (Pristiq)

Medications with potential longer washouts (Contact DCC)

Contraceptive Implants
Norplant (levonorgestrel implants)
Implanon
Previous clomiphene and letrozole use requires a two-month washout

19 Appendix C: SF 12 Health Survey

Your Health and Well-Being

This survey asks for your views about your health. This information will help keep track of how you feel and how well you are able to do your usual activities. *Thank you for completing this survey!*

For each of the following questions, please mark an ☒ in the one box that best describes your answer.

1. **In general, would you say your health is:**

Excellent	Very good	Good	Fair	Poor
☐ 1	☐ 2	☐ 3	☐ 4	☐ 5

2. **The following questions are about activities you might do during a typical day. Does <u>your health now limit you</u> in these activities? If so, how much?**

	Yes, limited a lot	Yes, limited a little	No, not limited at all
a Moderate activities, such as moving a table, pushing a vacuum cleaner, bowling, or playing golf	☐ 1	☐ 2	☐ 3
b Climbing several flights of stairs	☐ 1	☐ 2	☐ 3

SAMPLE

3. **During the <u>past 4 weeks</u>, how much of the time have you had any of the following problems with your work or other regular daily activities <u>as a result of your physical health</u>?**

	All of the time	Most of the time	Some of the time	A little of the time	None of the time
a Accomplished less than you would like	☐ 1	☐ 2	☐ 3	☐ 4	☐ 5
b Were limited in the kind of work or other activities	☐ 1	☐ 2	☐ 3	☐ 4	☐ 5

4. **During the <u>past 4 weeks</u>, how much of the time have you had any of the following problems with your work or other regular daily activities <u>as a result of any emotional problems</u> (such as feeling depressed or anxious)?**

	All of the time	Most of the time	Some of the time	A little of the time	None of the time
a Accomplished less than you would like	☐ 1	☐ 2	☐ 3	☐ 4	☐ 5
b Did work or other activities less carefully than usual	☐ 1	☐ 2	☐ 3	☐ 4	☐ 5

5. **During the <u>past 4 weeks</u>, how much did <u>pain</u> interfere with your normal work (including both work outside the home and housework)?**

Not at all	A little bit	Moderately	Quite a bit	Extremely
☐ 1	☐ 2	☐ 3	☐ 4	☐ 5

SAMPLE

6. **These questions are about how you feel and how things have been with you <u>during the past 4 weeks</u>. For each question, please give the one answer that comes closest to the way you have been feeling. How much of the time during the <u>past 4 weeks</u>…**

	All of the time	Most of the time	Some of the time	A little of the time	None of the time
a Have you felt calm and peaceful?	☐ 1	☐ 2	☐ 3	☐ 4	☐ 5
b Did you have a lot of energy?	☐ 1	☐ 2	☐ 3	☐ 4	☐ 5
c Have you felt downhearted and depressed?	☐ 1	☐ 2	☐ 3	☐ 4	☐ 5

7. **During the <u>past 4 weeks</u>, how much of the time has your <u>physical health or emotional problems</u> interfered with your social activities (like visiting with friends, relatives, etc.)?**

All of the time	Most of the time	Some of the time	A little of the time	None of the time
☐ 1	☐ 2	☐ 3	☐ 4	☐ 5

SAMPLE

Thank you for completing these questions!

20 Appendix D: Female Sexual Function Index

Female Sexual Function Index (FSFI) ©

Subject Identifier ______________________ Date _______________

INSTRUCTIONS: These questions ask about your sexual feelings and responses during the past 4 weeks. Please answer the following questions as honestly and clearly as possible. Your responses will be kept completely confidential. In answering these questions the following definitions apply:

Sexual activity can include caressing, foreplay, masturbation and vaginal intercourse.

Sexual intercourse is defined as penile penetration (entry) of the vagina.

Sexual stimulation includes situations like foreplay with a partner, self-stimulation (masturbation), or sexual fantasy.

CHECK ONLY ONE BOX PER QUESTION.

Sexual desire or interest is a feeling that includes wanting to have a sexual experience, feeling receptive to a partner's sexual initiation, and thinking or fantasizing about having sex.

1. Over the past 4 weeks, how **often** did you feel sexual desire or interest?

   - ☐ Almost always or always
   - ☐ Most times (more than half the time)
   - ☐ Sometimes (about half the time)
   - ☐ A few times (less than half the time)
   - ☐ Almost never or never

2. Over the past 4 weeks, how would you rate your **level** (degree) of sexual desire or interest?

   - ☐ Very high
   - ☐ High
   - ☐ Moderate
   - ☐ Low
   - ☐ Very low or none at all

Sexual arousal is a feeling that includes both physical and mental aspects of sexual excitement. It may include feelings of warmth or tingling in the genitals, lubrication (wetness), or muscle contractions.

3. Over the past 4 weeks, how **often** did you feel sexually aroused ("turned on") during sexual activity or intercourse?

- ☐ No sexual activity
- ☐ Almost always or always
- ☐ Most times (more than half the time)
- ☐ Sometimes (about half the time)
- ☐ A few times (less than half the time)
- ☐ Almost never or never

4. Over the past 4 weeks, how would you rate your **level** of sexual arousal ("turn on") during sexual activity or intercourse?

- ☐ No sexual activity
- ☐ Very high
- ☐ High
- ☐ Moderate
- ☐ Low
- ☐ Very low or none at all

5. Over the past 4 weeks, how **confident** were you about becoming sexually aroused during sexual activity or intercourse?

- ☐ No sexual activity
- ☐ Very high confidence
- ☐ High confidence
- ☐ Moderate confidence
- ☐ Low confidence
- ☐ Very low or no confidence

6. Over the past 4 weeks, how **often** have you been satisfied with your arousal (excitement) during sexual activity or intercourse?

- ☐ No sexual activity
- ☐ Almost always or always
- ☐ Most times (more than half the time)
- ☐ Sometimes (about half the time)
- ☐ A few times (less than half the time)
- ☐ Almost never or never

7. Over the past 4 weeks, how **often** did you become lubricated ("wet") during sexual activity or intercourse?

- ☐ No sexual activity
- ☐ Almost always or always
- ☐ Most times (more than half the time)
- ☐ Sometimes (about half the time)
- ☐ A few times (less than half the time)
- ☐ Almost never or never

8. Over the past 4 weeks, how **difficult** was it to become lubricated ("wet") during sexual activity or intercourse?

- ☐ No sexual activity
- ☐ Extremely difficult or impossible
- ☐ Very difficult
- ☐ Difficult
- ☐ Slightly difficult
- ☐ Not difficult

9. Over the past 4 weeks, how often did you **maintain** your lubrication ("wetness") until completion of sexual activity or intercourse?

- ☐ No sexual activity
- ☐ Almost always or always
- ☐ Most times (more than half the time)
- ☐ Sometimes (about half the time)
- ☐ A few times (less than half the time)
- ☐ Almost never or never

10. Over the past 4 weeks, how **difficult** was it to maintain your lubrication ("wetness") until completion of sexual activity or intercourse?

- ☐ No sexual activity
- ☐ Extremely difficult or impossible
- ☐ Very difficult
- ☐ Difficult
- ☐ Slightly difficult
- ☐ Not difficult

11. Over the past 4 weeks, when you had sexual stimulation or intercourse, how **often** did you reach orgasm (climax)?

- ☐ No sexual activity
- ☐ Almost always or always
- ☐ Most times (more than half the time)
- ☐ Sometimes (about half the time)
- ☐ A few times (less than half the time)
- ☐ Almost never or never

12. Over the past 4 weeks, when you had sexual stimulation or intercourse, how **difficult** was it for you to reach orgasm (climax)?

- ☐ No sexual activity
- ☐ Extremely difficult or impossible
- ☐ Very difficult
- ☐ Difficult
- ☐ Slightly difficult
- ☐ Not difficult

13. Over the past 4 weeks, how **satisfied** were you with your ability to reach orgasm (climax) during sexual activity or intercourse?

- ☐ No sexual activity
- ☐ Very satisfied
- ☐ Moderately satisfied
- ☐ About equally satisfied and dissatisfied
- ☐ Moderately dissatisfied
- ☐ Very dissatisfied

14. Over the past 4 weeks, how **satisfied** have you been with the amount of emotional closeness during sexual activity between you and your partner?

- ☐ No sexual activity
- ☐ Very satisfied
- ☐ Moderately satisfied
- ☐ About equally satisfied and dissatisfied
- ☐ Moderately dissatisfied
- ☐ Very dissatisfied

15. Over the past 4 weeks, how **satisfied** have you been with your sexual relationship with your partner?

- ☐ Very satisfied
- ☐ Moderately satisfied
- ☐ About equally satisfied and dissatisfied
- ☐ Moderately dissatisfied
- ☐ Very dissatisfied

16. Over the past 4 weeks, how **satisfied** have you been with your overall sexual life?

- ☐ Very satisfied
- ☐ Moderately satisfied
- ☐ About equally satisfied and dissatisfied
- ☐ Moderately dissatisfied
- ☐ Very dissatisfied

17. Over the past 4 weeks, how **often** did you experience discomfort or pain during vaginal penetration?

- ☐ Did not attempt intercourse
- ☐ Almost always or always
- ☐ Most times (more than half the time)
- ☐ Sometimes (about half the time)
- ☐ A few times (less than half the time)
- ☐ Almost never or never

18. Over the past 4 weeks, how **often** did you experience discomfort or pain following vaginal penetration?

- ☐ Did not attempt intercourse
- ☐ Almost always or always
- ☐ Most times (more than half the time)
- ☐ Sometimes (about half the time)
- ☐ A few times (less than half the time)
- ☐ Almost never or never

19. Over the past 4 weeks, how would you rate your **level** (degree) of discomfort or pain during or following vaginal penetration?

- ☐ Did not attempt intercourse
- ☐ Very high
- ☐ High
- ☐ Moderate
- ☐ Low
- ☐ Very low or none at all

Thank you for completing this questionnaire

21 Appendix E: PHQ-9

PATIENT HEALTH QUESTIONNAIRE (PHQ-9)

NAME:______________________ DATE:______________

Over the last *2 weeks,* how often have you been bothered by any of the following problems?
(use "✓" to indicate your answer)

	Not at all	Several days	More than half the days	Nearly every day
**1.** Little interest or pleasure in doing things	0	1	2	3
**2.** Feeling down, depressed, or hopeless	0	1	2	3
**3.** Trouble falling or staying asleep, or sleeping too much	0	1	2	3
**4.** Feeling tired or having little energy	0	1	2	3
**5.** Poor appetite or overeating	0	1	2	3
**6.** Feeling bad about yourself—or that you are a failure or have let yourself or your family down	0	1	2	3
**7.** Trouble concentrating on things, such as reading the newspaper or watching television	0	1	2	3
**8.** Moving or speaking so slowly that other people could have noticed. Or the opposite —being so figety or restless that you have been moving around a lot more than usual	0	1	2	3
**9.** Thoughts that you would be better off dead, or of hurting yourself	0	1	2	3
	add columns		+	+
(Healthcare professional: For interpretation of TOTAL, please refer to accompanying scoring card).	TOTAL:			

10. If you checked off *any problems,* how *difficult* have these problems made it for you to do your work, take care of things at home, or get along with other people?

Not difficult at all ________
Somewhat difficult ________
Very difficult ________
Extremely difficult ________

PHQ-9 Patient Depression Questionnaire

For initial diagnosis:

1. Patient completes PHQ-9 Quick Depression Assessment.
2. If there are at least 4 ✓s in the shaded section (including Questions #1 and #2), consider a depressive disorder. Add score to determine severity.

Consider Major Depressive Disorder

- if there are at least 5 ✓s in the shaded section (one of which corresponds to Question #1 or #2)

Consider Other Depressive Disorder

- if there are 2-4 ✓s in the shaded section (one of which corresponds to Question #1 or #2)

Note: Since the questionnaire relies on patient self-report, all responses should be verified by the clinician, and a definitive diagnosis is made on clinical grounds taking into account how well the patient understood the questionnaire, as well as other relevant information from the patient.
Diagnoses of Major Depressive Disorder or Other Depressive Disorder also require impairment of social, occupational, or other important areas of functioning (Question #10) and ruling out normal bereavement, a history of a Manic Episode (Bipolar Disorder), and a physical disorder, medication, or other drug as the biological cause of the depressive symptoms.

To monitor severity over time for newly diagnosed patients or patients in current treatment for depression:

1. Patients may complete questionnaires at baseline and at regular intervals (eg, every 2 weeks) at home and bring them in at their next appointment for scoring or they may complete the questionnaire during each scheduled appointment.
2. Add up ✓s by column. For every ✓: Several days = 1 More than half the days = 2 Nearly every day = 3
3. Add together column scores to get a TOTAL score.
4. Refer to the accompanying **PHQ-9 Scoring Box** to interpret the TOTAL score.
5. Results may be included in patient files to assist you in setting up a treatment goal, determining degree of response, as well as guiding treatment intervention.

Scoring: add up all checked boxes on PHQ-9

For every ✓ Not at all = 0; Several days = 1;
More than half the days = 2; Nearly every day = 3

Interpretation of Total Score

Total Score	Depression Severity
1-4	Minimal depression
5-9	Mild depression
10-14	Moderate depression
15-19	Moderately severe depression
20-27	Severe depression

22 Appendix F: Female Sexual Distress Scale (FSDS - Revised 2005)

INSTRUCTIONS
Below is a list of feelings and problems that women sometimes have concerning their sexuality. Please read each item carefully, and circle the number that best describes HOW OFTEN THAT PROBLEM HAS BOTHERED YOU OR CAUSED YOU DISTRESS DURING THE PAST 7 DAYS INCLUDING TODAY. Circle only one number for each item, and take care not to skip any items. If you change your mind, erase your first circle carefully. Read the example before beginning, and if you have any questions please ask about them.
Example: How often did you feel: **Personal responsibility for your sexual problems.**

NEVER	RARELY	OCCASIONALLY	FREQUENTLY	ALWAYS
0	1	2	3	4

**HOW OFTEN DID YOU FEEL:**					
1. Distressed about your sex life	0	1	2	3	4
2. Unhappy about your sexual relationship	0	1	2	3	4
3. Guilty about sexual difficulties	0	1	2	3	4
4. Frustrated by your sexual problems	0	1	2	3	4
5. Stressed about sex	0	1	2	3	4
6. Inferior because of sexual problems	0	1	2	3	4
7. Worried about sex	0	1	2	3	4
8. Sexually inadequate	0	1	2	3	4
9. Regrets about your sexuality	0	1	2	3	4
10. Embarrassed about sexual problems	0	1	2	3	4
11. Dissatisfied with your sex life	0	1	2	3	4
12. Angry about your sex life	0	1	2	3	4
13. Bothered by low sexual desire	0	1	2	3	4

Copyright □□2000 by American Foundation for Urological Disease Inc.

23 Appendix G: FertiQoL

FertiQoL International

Fertility Quality of Life Questionnaire (2008)

For each question, kindly check (tick the box) for the response that most closely reflects how you think and feel. Relate your answers to your current thoughts and feelings. Some questions may relate to your private life, but they are necessary to adequately measure all aspects of your life.

Please complete the items marked with an asterisk (*) only if you have a partner.

	For each question, check the response that is closest to your current thoughts and feelings	Very Poor	Poor	Nor good nor poor	Good	Very Good
A	How would you rate your health?	☐	☐	☐	☐	☐
	**For each question, check the response that is closest to your current thoughts and feelings**	**Very Dissatisfied**	**Dissatisfied**	**Neither Satisfied Nor Dissatisfied**	**Satisfied**	**Very Satisfied**
B	Are you satisfied with your quality of life?	☐	☐	☐	☐	☐
	**For each question, check the response that is closest to your current thoughts and feelings**	**Completely**	**A Great Deal**	**Moderately**	**Not Much**	**Not At All**
Q1	Are your attention and concentration impaired by thoughts of infertility?	☐	☐	☐	☐	☐
Q2	Do you think you cannot move ahead with other life goals and plans because of fertility problems?	☐	☐	☐	☐	☐
Q3	Do you feel drained or worn out because of fertility problems?	☐	☐	☐	☐	☐
Q4	Do you feel able to cope with your fertility problems?	☐	☐	☐	☐	☐
	**For each question, check the response that is closest to your current thoughts and feelings**	**Very Dissatisfied**	**Dissatisfied**	**Neither Satisfied Nor Dissatisfied**	**Satisfied**	**Very Satisfied**
Q5	Are you satisfied with the support you receive from friends with regard to your fertility problems?	☐	☐	☐	☐	☐
*Q6	Are you satisfied with your sexual relationship even though you have fertility problems?	☐	☐	☐	☐	☐
	**For each question, check the response that is closest to your current thoughts and feelings**	**Always**	**Very Often**	**Quite Often**	**Seldom**	**Never**
Q7	Do your fertility problems cause feelings of jealousy and resentment?	☐	☐	☐	☐	☐
Q8	Do you experience grief and/or feelings of loss about not being able to have a child (or more children)?	☐	☐	☐	☐	☐
Q9	Do you fluctuate between hope and despair because of fertility problems?	☐	☐	☐	☐	☐
Q10	Are you socially isolated because of fertility problems?	☐	☐	☐	☐	☐
*Q11	Are you and your partner affectionate with each other even though you have fertility problems?	☐	☐	☐	☐	☐
Q12	Do your fertility problems interfere with your day-to-day work or obligations?	☐	☐	☐	☐	☐
Q13	Do you feel uncomfortable attending social situations like holidays and celebrations because of your fertility problems?	☐	☐	☐	☐	☐
Q14	Do you feel your family can understand what you are going through?	☐	☐	☐	☐	☐
	**For each question, check the response that is closest to your current thoughts and feelings**	**An Extreme Amount**	**Very Much**	**A Moderate Amount**	**A Little**	**Not At All**
*Q15	Have fertility problems strengthened your commitment to your partner?	☐	☐	☐	☐	☐
Q16	Do you feel sad and depressed about your fertility problems?	☐	☐	☐	☐	☐
Q17	Do your fertility problems make you inferior to people with children?	☐	☐	☐	☐	☐
Q18	Are you bothered by fatigue because of fertility problems?	☐	☐	☐	☐	☐
*Q19	Have fertility problems had a negative impact on your relationship with your partner?	☐	☐	☐	☐	☐
*Q20	Do you find it difficult to talk to your partner about your feelings related to infertility?	☐	☐	☐	☐	☐
*Q21	Are you content with your relationship even though you have fertility problems?	☐	☐	☐	☐	☐
Q22	Do you feel social pressure on you to have (or have more) children?	☐	☐	☐	☐	☐
Q23	Do your fertility problems make you angry?	☐	☐	☐	☐	☐
Q24	Do you feel pain and physical discomfort because of your fertility problems?	☐	☐	☐	☐	☐

24 Appendix H: Stop Bang Questionnaire

STOP BANG Questionnaire

Height _____ inches/cm Weight _____ lb/kg
Age _____
Male/Female
BMI _____
Collar size of shirt: S, M, L, XL, or _____ inches/cm
Neck circumference* _____ cm

1. *S*noring
Do you *s*nore loudly (louder than talking or loud enough to be heard through closed doors)?
Yes No

2. *T*ired
Do you often feel *t*ired, fatigued, or sleepy during daytime?
Yes No

3. *O*bserved
Has anyone *o*bserved you stop breathing during your sleep?
Yes No

4. Blood *p*ressure
Do you have or are you being treated for high blood *p*ressure?
Yes No

5. *B*MI
*B*MI more than 35 kg/m^2?
Yes No

6. *A*ge
*A*ge over 50 yr old?
Yes No

7. *N*eck circumference
*N*eck circumference greater than 40 cm?
Yes No

8. *G*ender
*G*ender male?
Yes No

* Neck circumference is measured by staff

High risk of OSA: answering yes to three or more items
Low risk of OSA: answering yes to less than three items

Adapted from:
STOP Questionnaire
A Tool to Screen Patients for Obstructive Sleep Apnea
Frances Chung, F.R.C.P.C., Balaji Yegneswaran, M.B.B.S.,† Pu Liao, M.D.,‡ Sharon A. Chung, Ph.D.,§ Santhira Vairavanathan, M.B.B.S.,_ Sazzadul Islam, M.Sc.,_ Ali Khajehdehi, M.D.,† Colin M. Shapiro, F.R.C.P.C.#*

25 Appendix I: Epworth Sleepiness Scale

EPWORTH SLEEPINESS SCALE (ESS)

The following questionnaire will help you measure your general level of daytime sleepiness. You are to rate the chance that you would *doze off or fall asleep* during different routine daytime situations. Answers to the questions are rated on a reliable scale called the Epworth Sleepiness Scale (ESS). Each item is rated from 0 to 3: with 0 meaning you would never *doze or fall asleep* in a given situation; and 3 meaning that there is a very high chance that you would *doze or fall asleep* in that situation.

How likely are you to *doze off or fall asleep* in the following situations, in contrast to just feeling tired? Even if you haven't done some of the activities recently, think about how they would have affected you.

Use this scale to choose the most appropriate number for each situation:

0 = would never doze | **2 = moderate chance of dozing**
1 = slight chance of dozing | **3 = high chance of dozing**
It is important that you circle a number (0 to 3) for EACH situation.

SITUATION	CHANCE OF DOZING			
Sitting and Reading	0	1	2	3
Watching Television	0	1	2	3
Sitting inactive in a public place (theater/meeting)	0	1	2	3
As a passenger in a car for an hour without a break	0	1	2	3
Lying down to rest in the afternoon	0	1	2	3
Sitting and talking to someone	0	1	2	3
Sitting quietly after lunch (with no alcohol)	0	1	2	3
In a car, while stopped in traffic	0	1	2	3

TOTAL SCORE _________

Name: ___________________________________

Date: ______________________________

Revised 09/25/08

26 Appendix J: International Index of Erectile Function (IIEF)

(Write the number that best describes your erectile function for the past 4 weeks in the spaces provided.)

**Over the past four weeks:**<br>1. How often were you able to get an erection during sexual activity? _______	0 = No sexual activity<br>1 = Almost never/never<br>2 = A few times (much less than half the time)<br>3 = Sometimes (about half the time)<br>4 = Most times (much more than half the time)<br>5 = Almost always/always
2. When you had erections with sexual stimulation, how often were your erections hard enough for penetration? _______	0 = No sexual activity<br>1 = Almost never/never<br>2 = A few times (much less than half the time)<br>3 = Sometimes (about half the time)<br>4 = Most times (much more than half the time)<br>5 = Almost always/always
3. When you attempted sexual intercourse, how often were you able to penetrate (enter) your partner? _______	0 = Did not attempt intercourse<br>1 = Almost never/never<br>2 = A few times (much less than half the time)<br>3 = Sometimes (about half the time)<br>4 = Most times (much more than half the time)<br>5 = Almost always/always
4. During intercourse, how often were you able to maintain your erection after you had penetrated (entered) your partner? _______	0 = Did not attempt intercourse<br>1 = Almost never/never<br>2 = A few times (much less than half the time)<br>3 = Sometimes (about half the time)<br>4 = Most times (much more than half the time)<br>5 = Almost always/always
5. During sexual intercourse, how difficult was it to maintain your erection to completion of intercourse? _______	0 = Did not attempt intercourse<br>1 = Extremely difficult<br>2 = Very difficult<br>3 = Difficult<br>4 = Slightly difficult<br>5 = Not difficult
6. How many times have you attempted sexual intercourse? _______	0 = No attempts<br>1 = One to two attempts<br>2 = Three to four attempts<br>3 = Five to six attempts<br>4 = Seven to ten attempts<br>5 = Eleven or more attempts
7. When you attempted sexual intercourse, how often was it satisfactory for you? _______	0 = Did not attempt intercourse<br>1 = Almost never/never<br>2 = A few times (much less than half the time)<br>3 = Sometimes (about half the time)

	4 = Most times (much more than half the time)<br>5 = Almost always/always
8. How much have you enjoyed sexual intercourse? _______	0 = No intercourse<br>1 = No enjoyment<br>2 = Not very enjoyable<br>3 = Fairly enjoyable<br>4 = Highly enjoyable<br>5 = Very highly enjoyable
9. When you had sexual stimulation <u>or</u> intercourse, how often did you ejaculate? _______	0 = No sexual stimulation/intercourse<br>1 = Almost never/never<br>2 = A few times (much less than half the time)<br>3 = Sometimes (about half the time)<br>4 = Most times (much more than half the time)<br>5 = Almost always/always
10. When you had sexual stimulation <u>or</u> intercourse, how often did you have the feeling of orgasm or climax? _______	0 = No sexual stimulation/intercourse<br>1 = Almost never/never<br>2 = A few times (much less than half the time)<br>3 = Sometimes (about half the time)<br>4 = Most times (much more than half the time)<br>5 = Almost always/always
11. How often have you felt sexual desire? _______	1 = Almost never/never<br>2 = A few times (much less than half the time)<br>3 = Sometimes (about half the time)<br>4 = Most times (much more than half the time)<br>5 = Almost always/always
12. How would you rate your sexual desire? _______	1 = Very low/none at all<br>2 = Low<br>3 = Moderate<br>4 = High<br>5 = Very high
13. How satisfied have you been with your overall sex life? _______	1 = Very dissatisfied<br>2 = Moderately dissatisfied<br>3 = About equally satisfied and dissatisfied<br>4 = Moderately satisfied<br>5 = Very satisfied
14. How satisfied have you been with your <u>sexual relationship</u> with your partner? _______	1 = Very dissatisfied<br>2 = Moderately dissatisfied<br>3 = About equally satisfied and dissatisfied<br>4 = Moderately satisfied<br>5 = Very satisfied
15. How would you rate your <u>confidence</u> that you could get and keep an erection? _______	1 = Very low<br>2 = Low<br>3 = Moderate

	4 = High<br>5 = Very high

*RC Rosen, A Riley, G Wagner et al. The international index of erectile dysfunction (IIEF): a multidimensional scale for assessment of erectile dysfunction. Urology 1997 49: 822-30.

Copyright 2004 Urology Specialists, S.C. All rights reserved. Last Updated: 18 May 2004

27 Appendix K: Diet History Questionnaire II (DHQ II)

This is a sample form. Do not use for scanning.

NATIONAL INSTITUTES OF HEALTH

Diet History Questionnaire II

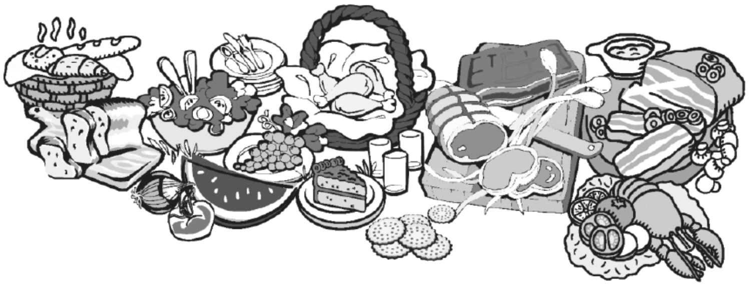

GENERAL INSTRUCTIONS

- Answer each question as best you can. Estimate if you are not sure. A guess is better than leaving a blank.
- Use only a black ball-point pen. Do not use a pencil or felt-tip pen. Do not fold, staple, or tear the pages.
- Put an X in the box next to your answer.
- If you make any changes, cross out the incorrect answer and put an X in the box next to the correct answer. Also draw a circle around the correct answer.
- If you mark NEVER, NO, or DON'T KNOW for a question, please follow any arrows or instructions that direct you to the next question.

BEFORE TURNING THE PAGE, PLEASE COMPLETE THE FOLLOWING QUESTIONS.

Today's date:

MONTH	DAY		YEAR
☐ Jan	|___|___|		☐ 2010
☐ Feb	☐0	☐0	☐ 2011
☐ Mar	☐1	☐1	☐ 2012
☐ Apr	☐2	☐2	☐ 2013
☐ May	☐3	☐3	☐ 2014
☐ Jun		☐4	☐ 2015
☐ Jul		☐5	☐ 2016
☐ Aug		☐6	☐ 2017
☐ Sep		☐7	☐ 2018
☐ Oct		☐8	☐ 2019
☐ Nov		☐9	☐ 2020
☐ Dec			

In what month were you born?

☐ Jan
☐ Feb
☐ Mar
☐ Apr
☐ May
☐ Jun
☐ Jul
☐ Aug
☐ Sep
☐ Oct
☐ Nov
☐ Dec

In what year were you born?

19 |___|___|

☐0	☐0
☐1	☐1
☐2	☐2
☐3	☐3
☐4	☐4
☐5	☐5
☐6	☐6
☐7	☐7
☐8	☐8
☐9	☐9

Are you male or female?

☐Male
☐Female

BAR CODE LABEL OR SUBJECT ID HERE

|___|___|___|___|___|___|___|___|

DHQ II PastYear

This is a sample form. Do not use for scanning.

1. Over the <u>past 12 months</u>, how often did you drink **carrot juice**?

☐ NEVER (GO TO QUESTION 2)

☐ 1 time per month or less
☐ 2–3 times per month
☐ 1–2 times per week
☐ 3–4 times per week
☐ 5–6 times per week
☐ 1 time per day
☐ 2–3 times per day
☐ 4–5 times per day
☐ 6 or more times per day

1a. Each time you drank **carrot juice**, how much did you usually drink?

☐ Less than ½ cup (4 ounces)
☐ ½ to 1¼ cups (4 to 10 ounces)
☐ More than 1¼ cups (10 ounces)

2. Over the <u>past 12 months</u>, how often did you drink **tomato juice** or **other vegetable juice**? *(Please do not include carrot juice.)*

☐ NEVER (GO TO QUESTION 3)

☐ 1 time per month or less
☐ 2–3 times per month
☐ 1–2 times per week
☐ 3–4 times per week
☐ 5–6 times per week
☐ 1 time per day
☐ 2–3 times per day
☐ 4–5 times per day
☐ 6 or more times per day

2a. Each time you drank **tomato juice** or **other vegetable juice**, how much did you usually drink?

☐ Less than ¾ cup (6 ounces)
☐ ¾ to 1¼ cups (6 to 10 ounces)
☐ More than 1¼ cups (10 ounces)

3. Over the <u>past 12 months</u>, how often did you drink **orange juice** or **grapefruit juice?**

☐ NEVER (GO TO QUESTION 4)

☐ 1 time per month or less
☐ 2–3 times per month
☐ 1–2 times per week
☐ 3–4 times per week
☐ 5–6 times per week
☐ 1 time per day
☐ 2–3 times per day
☐ 4–5 times per day
☐ 6 or more times per day

3a. Each time you drank **orange juice** or **grapefruit juice**, how much did you usually drink?

☐ Less than ¾ cup (6 ounces)
☐ ¾ to 1¼ cups (6 to 10 ounces)
☐ More than 1¼ cups (10 ounces)

Question 4 appears in the next column

3b. How often was the orange juice or grapefruit juice you drank **calcium-fortified**?

☐ Almost never or never
☐ About ¼ of the time
☐ About ½ of the time
☐ About ¾ of the time
☐ Almost always or always

4. Over the <u>past 12 months</u>, how often did you drink **other 100% fruit juice** or **100% fruit juice mixtures** (such as apple, grape, pineapple, or others)?

☐ NEVER (GO TO QUESTION 5)

☐ 1 time per month or less
☐ 2–3 times per month
☐ 1–2 times per week
☐ 3–4 times per week
☐ 5–6 times per week
☐ 1 time per day
☐ 2–3 times per day
☐ 4–5 times per day
☐ 6 or more times per day

4a. Each time you drank **other 100% fruit juice** or **100% fruit juice mixtures**, how much did you usually drink?

☐ Less than ¾ cup (6 ounces)
☐ ¾ to 1½ cups (6 to 12 ounces)
☐ More than 1½ cups (12 ounces)

4b. How often were the other 100% fruit juice or 100% fruit juice mixtures you drank **calcium-fortified**?

☐ Almost never or never
☐ About ¼ of the time
☐ About ½ of the time
☐ About ¾ of the time
☐ Almost always or always

5. How often did you drink **other fruit drinks** (such as cranberry cocktail, Hi-C, lemonade, or Kool-Aid, diet or regular)?

☐ NEVER (GO TO QUESTION 6)

☐ 1 time per month or less
☐ 2–3 times per month
☐ 1–2 times per week
☐ 3–4 times per week
☐ 5–6 times per week
☐ 1 time per day
☐ 2–3 times per day
☐ 4–5 times per day
☐ 6 or more times per day

Question 6 appears on the next page

This is a sample form. Do not use for scanning.

Over the past 12 months...

5a. Each time you drank **fruit drinks**, how much did you usually drink?

- ☐ Less than 1 cup (8 ounces)
- ☐ 1 to 2 cups (8 to 16 ounces)
- ☐ More than 2 cups (16 ounces)

5b. How often were your fruit drinks **diet** or **sugar-free**?

- ☐ Almost never or never
- ☐ About ¼ of the time
- ☐ About ½ of the time
- ☐ About ¾ of the time
- ☐ Almost always or always

6. How often did you drink **milk as a beverage** (NOT in coffee, NOT in cereal)? *(Please do not include chocolate milk and hot chocolate.)*

- ☐ NEVER (GO TO QUESTION 7)

- ☐ 1 time per month or less
- ☐ 2–3 times per month
- ☐ 1–2 times per week
- ☐ 3–4 times per week
- ☐ 5–6 times per week
- ☐ 1 time per day
- ☐ 2–3 times per day
- ☐ 4–5 times per day
- ☐ 6 or more times per day

6a. Each time you drank **milk as a beverage**, how much did you usually drink?

- ☐ Less than 1 cup (8 ounces)
- ☐ 1 to 1½ cups (8 to 12 ounces)
- ☐ More than 1½ cups (12 ounces)

6b. What kind of **milk** did you usually drink?

- ☐ Whole milk
- ☐ 2% fat milk
- ☐ 1 % fat milk
- ☐ Skim, nonfat, or ½% fat milk
- ☐ Soy milk
- ☐ Rice milk
- ☐ Other

7. How often did you drink **chocolate milk** (including hot chocolate)?

- ☐ NEVER (GO TO QUESTION 8)

- ☐ 1 time per month or less
- ☐ 2–3 times per month
- ☐ 1–2 times per week
- ☐ 3–4 times per week
- ☐ 5–6 times per week
- ☐ 1 time per day
- ☐ 2–3 times per day
- ☐ 4–5 times per day
- ☐ 6 or more times per day

Question 8 appears in the next column

7a. Each time you drank **chocolate milk**, how much did you usually drink?

- ☐ Less than 1 cup (8 ounces)
- ☐ 1 to 1½ cups (8 to 12 ounces)
- ☐ More than 1½ cups (12 ounces)

7b. How often was the chocolate milk **reduced-fat** or **fat-free**?

- ☐ Almost never or never
- ☐ About ¼ of the time
- ☐ About ½ of the time
- ☐ About ¾ of the time
- ☐ Almost always or always

8: How often did you drink **meal replacement** or **high-protein beverages** (such as Instant Breakfast, Ensure, Slimfast, Sustacal or others)?

- ☐ NEVER (GO TO QUESTION 9)

- ☐ 1 time per month or less
- ☐ 2–3 times per month
- ☐ 1–2 times per week
- ☐ 3–4 times per week
- ☐ 5–6 times per week
- ☐ 1 time per day
- ☐ 2–3 times per day
- ☐ 4–5 times per day
- ☐ 6 or more times per day

8a. Each time you drank **meal replacement** or **high-protein beverages**, how much did you usually drink?

- ☐ Less than 1 cup (8 ounces)
- ☐ 1 to 1½ cups (8 to 12 ounces)
- ☐ More than 1½ cups (12 ounces)

9. Over the past 12 months, did you drink **soda** or **pop**?

- ☐ NO (GO TO QUESTION 10)
- ☐ YES

9a. How often did you drink **soda** or **pop IN THE SUMMER**?

- ☐ NEVER

- ☐ 1 time per month or less
- ☐ 2–3 times per month
- ☐ 1–2 times per week
- ☐ 3–4 times per week
- ☐ 5–6 times per week
- ☐ 1 time per day
- ☐ 2–3 times per day
- ☐ 4–5 times per day
- ☐ 6 or more times per day

Question 10 appears on the next page

This is a sample form. Do not use for scanning.

Over the past 12 months…

9b. How often did you drink **soda** or **pop** **DURING THE REST OF THE YEAR**?

☐ NEVER

☐ 1 time per month or less
☐ 2–3 times per month
☐ 1–2 times per week
☐ 3–4 times per week
☐ 5–6 times per week
☐ 1 time per day
☐ 2–3 times per day
☐ 4–5 times per day
☐ 6 or more times per day

9c. Each time you drank **soda** or **pop,** how much did you usually drink?

☐ Less than 12 ounces or less than 1 can or bottle
☐ 12 to 16 ounces or 1 can or bottle
☐ More than 16 ounces or more than 1 can or bottle

9d. How often were these sodas or pop **diet** or **sugar-free**?

☐ Almost never or never
☐ About ¼ of the time
☐ About ½ of the time
☐ About ¾ of the time
☐ Almost always or always

9e. How often were these sodas or pop **caffeine-free**?

☐ Almost never or never
☐ About ¼ of the time
☐ About ½ of the time
☐ About ¾ of the time
☐ Almost always or always

10. Over the past 12 months, did you drink **sports drinks** (such as Propel, PowerAde, or Gatorade)?

☐ NO (GO TO QUESTION 11)

☐ YES

10a. How often did you drink **sports drinks IN THE SUMMER**?

☐ NEVER

☐ 1 time per month or less
☐ 2–3 times per month
☐ 1–2 times per week
☐ 3–4 times per week
☐ 5–6 times per week
☐ 1 time per day
☐ 2–3 times per day
☐ 4–5 times per day
☐ 6 or more times per day

Question 11 appears in the next column

10b. How often did you drink **sports drinks DURING THE REST OF THE YEAR**?

☐ NEVER

☐ 1 time per month or less
☐ 2–3 times per month
☐ 1–2 times per week
☐ 3–4 times per week
☐ 5–6 times per week
☐ 1 time per day
☐ 2–3 times per day
☐ 4–5 times per day
☐ 6 or more times per day

10c. Each time you drank **sports drinks**, how much did you usually drink?

☐ Less than 12 ounces or less than 1 bottle
☐ 12 to 24 ounces or 1 to 2 bottles
☐ More than 24 ounces or more than 2 bottles

11. Over the past 12 months, did you drink **energy drinks** (such as Red Bull or Jolt)?

☐ NO (GO TO QUESTION 12)

☐ YES

11a. How often did you drink **energy drinks IN THE SUMMER**?

☐ NEVER

☐ 1 time per month or less
☐ 2–3 times per month
☐ 1–2 times per week
☐ 3–4 times per week
☐ 5–6 times per week
☐ 1 time per day
☐ 2–3 times per day
☐ 4–5 times per day
☐ 6 or more times per day

11b. How often did you drink **energy drinks DURING THE REST OF THE YEAR**?

☐ NEVER

☐ 1 time per month or less
☐ 2–3 times per month
☐ 1–2 times per week
☐ 3–4 times per week
☐ 5–6 times per week
☐ 1 time per day
☐ 2–3 times per day
☐ 4–5 times per day
☐ 6 or more times per day

11c. Each time you drank **energy drinks**, how much did you usually drink?

☐ Less than 8 ounces or less than 1 cup
☐ 8 to 16 ounces or 1 to 2 cups
☐ More than 16 ounces or more than 2 cups

Question 12 appears on the next page

This is a sample form. Do not use for scanning.

Over the past 12 months…

12. Over the past 12 months, did you drink **beer**?

☐ NO (GO TO QUESTION 13)
☐ YES

12a. How often did you drink **beer IN THE SUMMER**?

☐ NEVER

☐ 1 time per month or less
☐ 2–3 times per month
☐ 1–2 times per week
☐ 3–4 times per week
☐ 5–6 times per week
☐ 1 time per day
☐ 2–3 times per day
☐ 4–5 times per day
☐ 6 or more times per day

12b. How often did you drink **beer DURING THE REST OF THE YEAR**?

☐ NEVER

☐ 1 time per month or less
☐ 2–3 times per month
☐ 1–2 times per week
☐ 3–4 times per week
☐ 5–6 times per week
☐ 1 time per day
☐ 2–3 times per day
☐ 4–5 times per day
☐ 6 or more times per day

12c. Each time you drank **beer**, how much did you usually drink?

☐ Less than a 12-ounce can or bottle
☐ 1 to 3 12-ounce cans or bottles
☐ More than 3 12-ounce cans or bottles

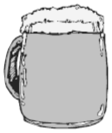

13. Over the past 12 months, did you drink **water** (including tap, bottled, and carbonated water)?

☐ NO (GO TO QUESTION 14)
☐ YES

13a. How often did you drink **water** (including tap, bottled, and carbonated water) **IN THE SUMMER**?

☐ NEVER

☐ 1 time per month or less
☐ 2–3 times per month
☐ 1–2 times per week
☐ 3–4 times per week
☐ 5–6 times per week
☐ 1 time per day
☐ 2–3 times per day
☐ 4–5 times per day
☐ 6 or more times per day

Question 14 appears in the next column

13b. How often did you drink **water** (including tap, bottled, and carbonated water) **DURING THE REST OF THE YEAR**?

☐ NEVER

☐ 1 time per month or less
☐ 2–3 times per month
☐ 1–2 times per week
☐ 3–4 times per week
☐ 5–6 times per week
☐ 1 time per day
☐ 2–3 times per day
☐ 4–5 times per day
☐ 6 or more times per day

13c. Each time you drank **water**, how much did you usually drink?

☐ Less than 12 ounces or less than 1 bottle
☐ 12 to 24 ounces or 1 to 2 bottles
☐ More than 24 ounces or more than 2 bottles

13d. How often was the water you drank **tap water**?

☐ Almost never or never
☐ About ¼ of the time
☐ About ½ of the time
☐ About ¾ of the time
☐ Almost always or always

13e. How often was the water you drank **bottled, sweetened water** (with low or no-calorie sweetener, including carbonated water)?

☐ Almost never or never
☐ About ¼ of the time
☐ About ½ of the time
☐ About ¾ of the time
☐ Almost always or always

13f. How often was the water you drank **bottled, unsweetened water** (including carbonated water)?

☐ Almost never or never
☐ About ¼ of the time
☐ About ½ of the time
☐ About ¾ of the time
☐ Almost always or always

14. How often did you drink **wine** or **wine coolers**?

☐ NEVER (GO TO QUESTION 15)

☐ 1 time per month or less
☐ 2–3 times per month
☐ 1–2 times per week
☐ 3–4 times per week
☐ 5–6 times per week
☐ 1 time per day
☐ 2–3 times per day
☐ 4–5 times per day
☐ 6 or more times per day

Question 15 appears on the next page

This is a sample form. Do not use for scanning.

Over the past 12 months…

14a. Each time you drank **wine** or **wine coolers**, how much did you usually drink?

- ☐ Less than 5 ounces or less than 1 glass
- ☐ 5 to 12 ounces or 1 to 2 glasses
- ☐ More than 12 ounces or more than 2 glasses

15. How often did you drink **liquor** or **mixed drinks**?

- ☐ NEVER (GO TO QUESTION 16)

- ☐ 1 time per month or less
- ☐ 2–3 times per month
- ☐ 1–2 times per week
- ☐ 3–4 times per week
- ☐ 5–6 times per week
- ☐ 1 time per day
- ☐ 2–3 times per day
- ☐ 4–5 times per day
- ☐ 6 or more times per day

15a. Each time you drank **liquor** or **mixed drinks**, how much did you usually drink?

- ☐ Less than 1 shot of liquor
- ☐ 1 to 3 shots of liquor
- ☐ More than 3 shots of liquor

16. Over the past 12 months, did you eat **oatmeal, grits,** or **other cooked cereal?**

- ☐ NO (GO TO QUESTION 17)
- ☐ YES

16a. How often did you eat **oatmeal**, **grits**, or **other cooked cereal IN THE WINTER**?

- ☐ NEVER

- ☐ 1–6 times per winter
- ☐ 7–11 times per winter
- ☐ 1 time per month
- ☐ 2–3 times per month
- ☐ 1 time per week
- ☐ 2 times per week
- ☐ 3–4 times per week
- ☐ 5–6 times per week
- ☐ 1 time per day
- ☐ 2 or more times per day

16b. How often did you eat **oatmeal**, **grits**, or **other cooked cereal DURING THE REST OF THE YEAR**?

- ☐ NEVER

- ☐ 1–6 times per year
- ☐ 7–11 times per year
- ☐ 1 time per month
- ☐ 2–3 times per month
- ☐ 1 time per week
- ☐ 2 times per week
- ☐ 3–4 times per week
- ☐ 5–6 times per week
- ☐ 1 time per day
- ☐ 2 or more times per day

Question 17 appears in the next column

16c. Each time you ate **oatmeal, grits,** or **other cooked cereal,** how much did you usually eat?

- ☐ Less than ¾ cup
- ☐ ¾ to 1¼ cups
- ☐ More than 1¼ cups

16d. How often was **butter** or **margarine** added to your oatmeal, grits or other cooked cereal?

- ☐ Almost never or never
- ☐ About ¼ of the time
- ☐ About ½ of the time
- ☐ About ¾ of the time
- ☐ Almost always or always

17. How often did you eat **cold cereal**?

- ☐ NEVER (GO TO QUESTION 18)

- ☐ 1–6 times per year
- ☐ 7–11 times per year
- ☐ 1 time per month
- ☐ 2–3 times per month
- ☐ 1 time per week
- ☐ 2 times per week
- ☐ 3–4 times per week
- ☐ 5–6 times per week
- ☐ 1 time per day
- ☐ 2 or more times per day

17a. Each time you ate **cold cereal**, how much did you usually eat?

- ☐ Less than 1 cup
- ☐ 1 to 2½ cups
- ☐ More than 2½ cups

17b. How often was the cold cereal you ate **Total Raisin Bran**, **Total Cereal**, or **Product 19**?

- ☐ Almost never or never
- ☐ About ¼ of the time
- ☐ About ½ of the time
- ☐ About ¾ of the time
- ☐ Almost always or always

17c. How often was the cold cereal you ate **All Bran**, **Fiber One**, **100% Bran**, or **All-Bran Bran Buds**?

- ☐ Almost never or never
- ☐ About ¼ of the time
- ☐ About ½ of the time
- ☐ About ¾ of the time
- ☐ Almost always or always

Question 18 appears on the next page

This is a sample form. Do not use for scanning.

Over the past 12 months…

17d. How often was the cold cereal you ate **some other bran** or **fiber cereal** (such as Cheerios, Shredded Wheat, Raisin Bran, Bran Flakes, Grape-Nuts, Granola, Wheaties, or Healthy Choice)?

- ☐ Almost never or never
- ☐ About ¼ of the time
- ☐ About ½ of the time
- ☐ About ¾ of the time
- ☐ Almost always or always

17e. How often was the cold cereal you ate any **other type of cold cereal** (such as Corn Flakes, Rice Krispies, Frosted Flakes, Special K, Froot Loops, Cap'n Crunch, or others)?

- ☐ Almost never or never
- ☐ About ¼ of the time
- ☐ About ½ of the time
- ☐ About ¾ of the time
- ☐ Almost always or always

17f. Was **milk** added to your cold cereal?

- ☐ NO (GO TO QUESTION 18)
- ☐ YES

17g. What kind of **milk** was usually added?

- ☐ Whole milk
- ☐ 2% fat milk
- ☐ 1% fat milk
- ☐ Skim, nonfat, or ½% fat milk
- ☐ Soy milk
- ☐ Rice milk
- ☐ Other

17h. Each time **milk was added to your cold cereal**, how much was usually added?

- ☐ Less than ½ cup
- ☐ ½ to 1 cup
- ☐ More than 1 cup

18. How often did you eat **applesauce**?

- ☐ NEVER (GO TO QUESTION 19)

☐ 1–6 times per year	☐ 2 times per week
☐ 7–11 times per year	☐ 3–4 times per week
☐ 1 time per month	☐ 5–6 times per week
☐ 2–3 times per month	☐ 1 time per day
☐ 1 time per week	☐ 2 or more times per day

Question 19 appears in the next column

18a. Each time you ate **applesauce**, how much did you usually eat?

- ☐ Less than ½ cup
- ☐ ½ to 1 cup
- ☐ More than 1 cup

19. How often did you eat **apples**?

- ☐ NEVER (GO TO QUESTION 20)

☐ 1–6 times per year	☐ 2 times per week
☐ 7–11 times per year	☐ 3–4 times per week
☐ 1 time per month	☐ 5–6 times per week
☐ 2–3 times per month	☐ 1 time per day
☐ 1 time per week	☐ 2 or more times per day

19a. Each time you ate **apples**, how many did you usually eat?

- ☐ Less than 1 apple
- ☐ 1 apple
- ☐ More than 1 apple

20. How often did you eat **pears** (fresh, canned, or frozen)?

- ☐ NEVER (GO TO QUESTION 21)

☐ 1–6 times per year	☐ 2 times per week
☐ 7–11 times per year	☐ 3–4 times per week
☐ 1 time per month	☐ 5–6 times per week
☐ 2–3 times per month	☐ 1 time per day
☐ 1 time per week	☐ 2 or more times per day

20a. Each time you ate **pears**, how many did you usually eat?

- ☐ Less than 1 pear
- ☐ 1 pear
- ☐ More than 1 pear

21. How often did you eat **bananas?**

- ☐ NEVER (GO TO QUESTION 22)

☐ 1–6 times per year	☐ 2 times per week
☐ 7–11 times per year	☐ 3–4 times per week
☐ 1 time per month	☐ 5–6 times per week
☐ 2–3 times per month	☐ 1 time per day
☐ 1 time per week	☐ 2 or more times per day

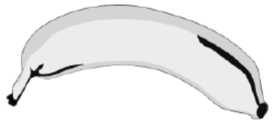

Question 22 appears on the next page

This is a sample form. Do not use for scanning.

Over the past 12 months…

21a. Each time you ate **bananas**, how many did you usually eat?

- ☐ Less than 1 banana
- ☐ 1 banana
- ☐ More than 1 banana

22. How often did you eat **dried fruit** (such as prunes or raisins)? *(Please do not include dried apricots.)*

- ☐ NEVER (GO TO QUESTION 23)

- ☐ 1–6 times per year
- ☐ 7–11 times per year
- ☐ 1 time per month
- ☐ 2–3 times per month
- ☐ 1 time per week
- ☐ 2 times per week
- ☐ 3–4 times per week
- ☐ 5–6 times per week
- ☐ 1 time per day
- ☐ 2 or more times per day

22a. Each time you ate **dried fruit**, how much did you usually eat?

- ☐ Less than 2 tablespoons
- ☐ 2 to 5 tablespoons
- ☐ More than 5 tablespoons

23. Over the past 12 months, did you eat **peaches, nectarines,** or **plums**?

- ☐ NO (GO TO QUESTION 24)
- ☐ YES

23a. How often did you eat **fresh peaches, nectarines**, or **plums WHEN IN SEASON**?

- ☐ NEVER

- ☐ 1–6 times per season
- ☐ 7–11 times per season
- ☐ 1 time per month
- ☐ 2–3 times per month
- ☐ 1 time per week
- ☐ 2 times per week
- ☐ 3–4 times per week
- ☐ 5–6 times per week
- ☐ 1 time per day
- ☐ 2 or more times per day

23b. How often did you eat **peaches, nectarines,** or **plums** (fresh, canned, or frozen) **DURING THE REST OF THE YEAR**?

- ☐ NEVER

- ☐ 1–6 times per year
- ☐ 7–11 times per year
- ☐ 1 time per month
- ☐ 2–3 times per month
- ☐ 1 time per week
- ☐ 2 times per week
- ☐ 3–4 times per week
- ☐ 5–6 times per week
- ☐ 1 time per day
- ☐ 2 or more times per day

Question 24 appears in the next column

23c. Each time you ate **peaches, nectarines,** or **plums,** how much did you usually eat?

- ☐ Less than 1 fruit or less than ½ cup
- ☐ 1 to 2 fruits or ½ to ¾ cup
- ☐ More than 2 fruits or more than ¾ cup

24. How often did you eat **grapes**?

- ☐ NEVER (GO TO QUESTION 25)

- ☐ 1–6 times per year
- ☐ 7–11 times per year
- ☐ 1 time per month
- ☐ 2–3 times per month
- ☐ 1 time per week
- ☐ 2 times per week
- ☐ 3–4 times per week
- ☐ 5–6 times per week
- ☐ 1 time per day
- ☐ 2 or more times per day

24a. Each time you ate **grapes**, how much did you usually eat?

- ☐ Less than ½ cup or less than 10 grapes
- ☐ ½ to 1 cup or 10 to 30 grapes
- ☐ More than 1 cup or more than 30 grapes

25. Over the past 12 months, did you eat **cantaloupe**?

- ☐ NO (GO TO QUESTION 26)
- ☐ YES

25a. How often did you eat **fresh cantaloupe WHEN IN SEASON**?

- ☐ NEVER

- ☐ 1–6 times per season
- ☐ 7–11 times per season
- ☐ 1 time per month
- ☐ 2–3 times per month
- ☐ 1 time per week
- ☐ 2 times per week
- ☐ 3–4 times per week
- ☐ 5–6 times per week
- ☐ 1 time per day
- ☐ 2 or more times per day

25b. How often did you eat **cantaloupe** (fresh or frozen) **DURING THE REST OF THE YEAR**?

- ☐ NEVER

- ☐ 1–6 times per year
- ☐ 7–11 times per year
- ☐ 1 time per month
- ☐ 2–3 times per month
- ☐ 1 time per week
- ☐ 2 times per week
- ☐ 3–4 times per week
- ☐ 5–6 times per week
- ☐ 1 time per day
- ☐ 2 or more times per day

Question 26 appears on the next page

This is a sample form. Do not use for scanning.

Over the past 12 months…

25c. Each time you ate **cantaloupe**, how much did you usually eat?

- ☐ Less than ¼ melon or less than ½ cup
- ☐ ¼ melon or ½ to 1 cup
- ☐ More than ¼ melon or more than 1 cup

26. Over the past 12 months, did you eat **melon, other than cantaloupe** (such as watermelon or honeydew)?

- ☐ NO (GO TO QUESTION 27)
- ☐ YES

26a. How often did you eat **fresh melon, other than cantaloupe, WHEN IN SEASON**?

- ☐ NEVER
- ☐ 1–6 times per season
- ☐ 7–11 times per season
- ☐ 1 time per month
- ☐ 2–3 times per month
- ☐ 1 time per week
- ☐ 2 times per week
- ☐ 3–4 times per week
- ☐ 5–6 times per week
- ☐ 1 time per day
- ☐ 2 or more times per day

26b. How often did you eat **melon other than cantaloupe** (fresh or frozen) **DURING THE REST OF THE YEAR**?

- ☐ NEVER
- ☐ 1–6 times per year
- ☐ 7–11 times per year
- ☐ 1 time per month
- ☐ 2–3 times per month
- ☐ 1 time per week
- ☐ 2 times per week
- ☐ 3–4 times per week
- ☐ 5–6 times per week
- ☐ 1 time per day
- ☐ 2 or more times per day

26c. Each time you ate **melon other than cantaloupe**, how much did you usually eat?

- ☐ Less than ½ cup or 1 small wedge
- ☐ ½ to 2 cups or 1 medium wedge
- ☐ More than 2 cups or 1 large wedge

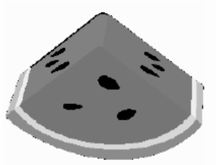

Question 27 appears in the next column

27. Over the past 12 months, did you eat **strawberries**?

- ☐ NO (GO TO QUESTION 28)
- ☐ YES

27a. How often did you eat **fresh strawberries WHEN IN SEASON**?

- ☐ NEVER
- ☐ 1–6 times per season
- ☐ 7–11 times per season
- ☐ 1 time per month
- ☐ 2–3 times per month
- ☐ 1 time per week
- ☐ 2 times per week
- ☐ 3–4 times per week
- ☐ 5–6 times per week
- ☐ 1 time per day
- ☐ 2 or more times per day

27b. How often did you eat **strawberries** (fresh or frozen) **DURING THE REST OF THE YEAR**?

- ☐ NEVER
- ☐ 1–6 times per year
- ☐ 7–11 times per year
- ☐ 1 time per month
- ☐ 2–3 times per month
- ☐ 1 time per week
- ☐ 2 times per week
- ☐ 3–4 times per week
- ☐ 5–6 times per week
- ☐ 1 time per day
- ☐ 2 or more times per day

27c. Each time you ate **strawberries**, how much did you usually eat?

- ☐ Less than ¼ cup or less than 3 berries
- ☐ ¼ to ¾ cup or 3 to 8 berries
- ☐ More than ¾ cup or more than 8 berries

28. Over the past 12 months, did you eat **oranges, tangerines,** or **clementines**?

- ☐ NO (GO TO QUESTION 29)
- ☐ YES

28a. How often did you eat **fresh oranges, tangerines**, or **clementines WHEN IN SEASON**?

- ☐ NEVER
- ☐ 1–6 times per season
- ☐ 7–11 times per season
- ☐ 1 time per month
- ☐ 2–3 times per month
- ☐ 1 time per week
- ☐ 2 times per week
- ☐ 3–4 times per week
- ☐ 5–6 times per week
- ☐ 1 time per day
- ☐ 2 or more times per day

Question 29 appears on the next page

This is a sample form. Do not use for scanning.

Over the past 12 months…

28b. How often did you eat **oranges**, **tangerines**, or **clementines** (fresh or canned) **DURING THE REST OF THE YEAR**?

☐ NEVER

☐ 1–6 times per year
☐ 7–11 times per year
☐ 1 time per month
☐ 2–3 times per month
☐ 1 time per week
☐ 2 times per week
☐ 3–4 times per week
☐ 5–6 times per week
☐ 1 time per day
☐ 2 or more times per day

28c. Each time you ate **oranges, tangerines,** or **clementines,** how many did you usually eat?

☐ Less than 1 fruit
☐ 1 fruit
☐ More than 1 fruit

29. Over the past 12 months, did you eat **grapefruit**?

☐ NO (GO TO QUESTION 30)
☐ YES

29a. How often did you eat **fresh grapefruit WHEN IN SEASON**?

☐ NEVER

☐ 1–6 times per season
☐ 7–11 times per season
☐ 1 time per month
☐ 2–3 times per month
☐ 1 time per week
☐ 2 times per week
☐ 3–4 times per week
☐ 5–6 times per week
☐ 1 time per day
☐ 2 or more times per day

29b. How often did you eat **grapefruit** (fresh or canned) **DURING THE REST OF THE YEAR?**

☐ NEVER

☐ 1–6 times per year
☐ 7–11 times per year
☐ 1 time per month
☐ 2–3 times per month
☐ 1 time per week
☐ 2 times per week
☐ 3–4 times per week
☐ 5–6 times per week
☐ 1 time per day
☐ 2 or more times per day

Question 30 appears in the next column

29c. Each time you ate **grapefruit**, how much did you usually eat?

☐ Less than ½ grapefruit
☐ ½ grapefruit
☐ More than ½ grapefruit

30. How often did you eat **pineapple**?

☐ NEVER (GO TO QUESTION 31)

☐ 1–6 times per year
☐ 7–11 times per year
☐ 1 time per month
☐ 2–3 times per month
☐ 1 time per week
☐ 2 times per week
☐ 3–4 times per week
☐ 5–6 times per week
☐ 1 time per day
☐ 2 or more times per day

30a. Each time you ate **pineapple**, how much did you usually eat?

☐ Less than ¼ cup or less than 1 medium slice
☐ ¼ to ¾ cup or 1 medium slice
☐ More than ¾ cup or more than 1 medium slice

31. How often did you eat **other kinds of fruit**?

☐ NEVER (GO TO QUESTION 32)

☐ 1–6 times per year
☐ 7–11 times per year
☐ 1 time per month
☐ 2–3 times per month
☐ 1 time per week
☐ 2 times per week
☐ 3–4 times per week
☐ 5–6 times per week
☐ 1 time per day
☐ 2 or more times per day

31a. Each time you ate **other kinds of fruit**, how much did you usually eat?

☐ Less than ¼ cup
☐ ¼ to ¾ cup
☐ More than ¾ cup

32. How often did you eat **COOKED greens** (such as spinach, turnip, collard, mustard, chard, or kale)?

☐ NEVER (GO TO QUESTION 33)

☐ 1–6 times per year
☐ 7–11 times per year
☐ 1 time per month
☐ 2–3 times per month
☐ 1 time per week
☐ 2 times per week
☐ 3–4 times per week
☐ 5–6 times per week
☐ 1 time per day
☐ 2 or more times per day

Question 33 appears on the next page

This is a sample form. Do not use for scanning.

Over the past 12 months…

32a. Each time you ate **COOKED greens**, how much did you usually eat?

- ☐ Less than ½ cup
- ☐ ½ to 1 cup
- ☐ More than 1 cup

33. How often did you eat **RAW greens** (such as spinach, turnip, collard, mustard, chard, or kale)? *(We will ask about lettuce later.)*

- ☐ NEVER (GO TO QUESTION 34)
- ☐ 1–6 times per year
- ☐ 7–11 times per year
- ☐ 1 time per month
- ☐ 2–3 times per month
- ☐ 1 time per week
- ☐ 2 times per week
- ☐ 3–4 times per week
- ☐ 5–6 times per week
- ☐ 1 time per day
- ☐ 2 or more times per day

33a. Each time you ate **RAW greens**, how much did you usually eat?

- ☐ Less than ½ cup
- ☐ ½ to 1 cup
- ☐ More than 1 cup

34. How often did you eat **coleslaw**?

- ☐ NEVER (GO TO QUESTION 35)
- ☐ 1–6 times per year
- ☐ 7–11 times per year
- ☐ 1 time per month
- ☐ 2–3 times per month
- ☐ 1 time per week
- ☐ 2 times per week
- ☐ 3–4 times per week
- ☐ 5–6 times per week
- ☐ 1 time per day
- ☐ 2 or more times per day

34a. Each time you ate **coleslaw**, how much did you usually eat?

- ☐ Less than ¼ cup
- ☐ ¼ to ¾ cup
- ☐ More than ¾ cup

35. How often did you eat **sauerkraut** or **cabbage** (other than coleslaw)?

- ☐ NEVER (GO TO QUESTION 36)
- ☐ 1–6 times per year
- ☐ 7–11 times per year
- ☐ 1 time per month
- ☐ 2–3 times per month
- ☐ 1 time per week
- ☐ 2 times per week
- ☐ 3–4 times per week
- ☐ 5–6 times per week
- ☐ 1 time per day
- ☐ 2 or more times per day

Question 36 appears in the next column

35a. Each time you ate **sauerkraut** or **cabbage**, how much did you usually eat?

- ☐ Less than ¼ cup
- ☐ ¼ to 1 cup
- ☐ More than 1 cup

36. How often did you eat **carrots** (fresh, canned, or frozen)?

- ☐ NEVER (GO TO QUESTION 37)
- ☐ 1–6 times per year
- ☐ 7–11 times per year
- ☐ 1 time per month
- ☐ 2–3 times per month
- ☐ 1 time per week
- ☐ 2 times per week
- ☐ 3–4 times per week
- ☐ 5–6 times per week
- ☐ 1 time per day
- ☐ 2 or more times per day

36a. Each time you ate **carrots**, how much did you usually eat?

- ☐ Less than ¼ cup or less than 2 baby carrots
- ☐ ¼ to ½ cup or 2 to 5 baby carrots
- ☐ More than ½ cup or more than 5 baby carrots

37. How often did you eat **string beans** or **green beans** (fresh, canned, or frozen)?

- ☐ NEVER (GO TO QUESTION 38)
- ☐ 1–6 times per year
- ☐ 7–11 times per year
- ☐ 1 time per month
- ☐ 2–3 times per month
- ☐ 1 time per week
- ☐ 2 times per week
- ☐ 3–4 times per week
- ☐ 5–6 times per week
- ☐ 1 time per day
- ☐ 2 or more times per day

37a. Each time you ate **string beans** or **green beans**, how much did you usually eat?

- ☐ Less than ½ cup
- ☐ ½ to 1 cup
- ☐ More than 1 cup

38. How often did you eat **peas** (fresh, canned, or frozen)?

- ☐ NEVER (GO TO QUESTION 39)
- ☐ 1–6 times per year
- ☐ 7–11 times per year
- ☐ 1 time per month
- ☐ 2–3 times per month
- ☐ 1 time per week
- ☐ 2 times per week
- ☐ 3–4 times per week
- ☐ 5–6 times per week
- ☐ 1 time per day
- ☐ 2 or more times per day

Question 39 appears on the next page

This is a sample form. Do not use for scanning.

Over the past 12 months…

38a. Each time you ate **peas**, how much did you usually eat?

- ☐ Less than ¼ cup
- ☐ ¼ to ¾ cup
- ☐ More than ¾ cup

39. Over the past 12 months, did you eat **corn**?

- ☐ NO (GO TO QUESTION 40)
- ☐ YES

39a. How often did you eat **fresh corn WHEN IN SEASON**?

- ☐ NEVER
- ☐ 1–6 times per season
- ☐ 7–11 times per season
- ☐ 1 time per month
- ☐ 2–3 times per month
- ☐ 1 time per week
- ☐ 2 times per week
- ☐ 3–4 times per week
- ☐ 5–6 times per week
- ☐ 1 time per day
- ☐ 2 or more times per day

39b. How often did you eat **corn** (fresh, canned, or frozen) **DURING THE REST OF THE YEAR**?

- ☐ NEVER
- ☐ 1–6 times per year
- ☐ 7–11 times per year
- ☐ 1 time per month
- ☐ 2–3 times per month
- ☐ 1 time per week
- ☐ 2 times per week
- ☐ 3–4 times per week
- ☐ 5–6 times per week
- ☐ 1 time per day
- ☐ 2 or more times per day

39c. Each time you ate **corn**, how much did you usually eat?

- ☐ Less than 1 ear or less than ½ cup
- ☐ 1 ear or ½ to 1 cup
- ☐ More than 1 ear or more than 1 cup

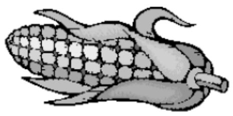

Question 40 appears in the next column

40. How often did you eat **broccoli** (fresh or frozen)?

- ☐ NEVER (GO TO QUESTION 41)
- ☐ 1–6 times per year
- ☐ 7–11 times per year
- ☐ 1 time per month
- ☐ 2–3 times per month
- ☐ 1 time per week
- ☐ 2 times per week
- ☐ 3–4 times per week
- ☐ 5–6 times per week
- ☐ 1 time per day
- ☐ 2 or more times per day

40a. Each time you ate **broccoli**, how much did you usually eat?

- ☐ Less than ¼ cup
- ☐ ¼ to 1 cup
- ☐ More than 1 cup

41. How often did you eat **cauliflower** or **Brussels sprouts** (fresh or frozen)?

- ☐ NEVER (GO TO QUESTION 42)
- ☐ 1–6 times per year
- ☐ 7–11 times per year
- ☐ 1 time per month
- ☐ 2–3 times per month
- ☐ 1 time per week
- ☐ 2 times per week
- ☐ 3–4 times per week
- ☐ 5–6 times per week
- ☐ 1 time per day
- ☐ 2 or more times per day

41a. Each time you ate **cauliflower** or **Brussels sprouts**, how much did you usually eat?

- ☐ Less than ¼ cup
- ☐ ¼ to ½ cup
- ☐ More than ½ cup

42. How often did you eat **asparagus** (fresh or frozen)?

- ☐ NEVER (GO TO QUESTION 43)
- ☐ 1–6 times per year
- ☐ 7–11 times per year
- ☐ 1 time per month
- ☐ 2–3 times per month
- ☐ 1 time per week
- ☐ 2 times per week
- ☐ 3–4 times per week
- ☐ 5–6 times per week
- ☐ 1 time per day
- ☐ 2 or more times per day

42a. Each time you ate **asparagus**, how much did you usually eat?

- ☐ Less than ⅓ cup or less than 4 spears
- ☐ ⅓ to ⅔ cup or 4 to 7 spears
- ☐ More than ⅔ cup or more than 7 spears

Question 43 appears on the next page

This is a sample form. Do not use for scanning.

Over the past 12 months…

43. How often did you eat **winter squash** (such as pumpkin, butternut, or acorn)?

- ☐ NEVER (GO TO QUESTION 44)
- ☐ 1–6 times per year
- ☐ 7–11 times per year
- ☐ 1 time per month
- ☐ 2–3 times per month
- ☐ 1 time per week
- ☐ 2 times per week
- ☐ 3–4 times per week
- ☐ 5–6 times per week
- ☐ 1 time per day
- ☐ 2 or more times per day

43a. Each time you ate **winter squash**, how much did you usually eat?

- ☐ Less than ½ cup
- ☐ ½ to ¾ cup
- ☐ More than ¾ cup

44. How often did you eat **mixed vegetables**?

- ☐ NEVER (GO TO QUESTION 45)
- ☐ 1–6 times per year
- ☐ 7–11 times per year
- ☐ 1 time per month
- ☐ 2–3 times per month
- ☐ 1 time per week
- ☐ 2 times per week
- ☐ 3–4 times per week
- ☐ 5–6 times per week
- ☐ 1 time per day
- ☐ 2 or more times per day

44a. Each time you ate **mixed vegetables**, how much did you usually eat?

- ☐ Less than ½ cup
- ☐ ½ to 1 cup
- ☐ More than 1 cup

45. How often did you eat **onions**?

- ☐ NEVER (GO TO QUESTION 46)
- ☐ 1–6 times per year
- ☐ 7–11 times per year
- ☐ 1 time per month
- ☐ 2–3 times per month
- ☐ 1 time per week
- ☐ 2 times per week
- ☐ 3–4 times per week
- ☐ 5–6 times per week
- ☐ 1 time per day
- ☐ 2 or more times per day

45a. Each time you ate **onions**, how much did you usually eat?

- ☐ Less than 1 slice or less than 1 tablespoon
- ☐ 1 slice or 1 to 4 tablespoons
- ☐ More than 1 slice or more than 4 tablespoons

Question 46 appears in the next column

46. Now think about all the **cooked vegetables** you ate in the past 12 months and how they were prepared. How often were your vegetables **COOKED WITH** some sort of **fat**, including oil spray? *(Please do not include potatoes.)*

- ☐ NEVER (GO TO QUESTION 47)
- ☐ 1–6 times per year
- ☐ 7–11 times per year
- ☐ 1 time per month
- ☐ 2–3 times per month
- ☐ 1 time per week
- ☐ 2 times per week
- ☐ 3–4 times per week
- ☐ 5–6 times per week
- ☐ 1 time per day
- ☐ 2 or more times per day

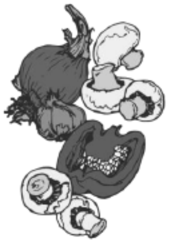

46a. Which fats were usually added to your vegetables **DURING COOKING**? *(Please do not include potatoes.* ***Mark all that apply.****)*

- ☐ Margarine (including low-fat)
- ☐ Butter (including low-fat)
- ☐ Lard, fatback, or bacon fat
- ☐ Olive oil
- ☐ Corn oil
- ☐ Canola or rapeseed oil
- ☐ Oil spray, such as Pam or others
- ☐ Other kinds of oils
- ☐ None of the above

47. Now, thinking again about all the **cooked vegetables** you ate in the past 12 months, how often was some sort of fat, sauce, or dressing added **AFTER COOKING OR AT THE TABLE**? *(Please do not include potatoes.)*

- ☐ NEVER (GO TO QUESTION 48)
- ☐ 1–6 times per year
- ☐ 7–11 times per year
- ☐ 1 time per month
- ☐ 2–3 times per month
- ☐ 1–2 times per week
- ☐ 3–4 times per week
- ☐ 5–6 times per week
- ☐ 1 time per day
- ☐ 2 times per day
- ☐ 3 or more times per day

Question 48 appears on the next page

This is a sample form. Do not use for scanning.

Over the past 12 months…

47a. Which fats, sauces, or dressings were usually added **AFTER COOKING OR AT THE TABLE**? *(Please do not include potatoes.* ***Mark all that apply.****)*

- ☐ Margarine (including low-fat)
- ☐ Butter (including low-fat)
- ☐ Lard, fatback, or bacon fat
- ☐ Salad dressing
- ☐ Cheese sauce
- ☐ White sauce
- ☐ Other

47b. If margarine, butter, lard, fatback, or bacon fat was added to your cooked vegetables **AFTER COOKING OR AT THE TABLE**, how much did you usually add?

- ☐ Did not usually add these
- ☐ Less than 1 teaspoon
- ☐ 1 to 3 teaspoons
- ☐ More than 3 teaspoons

47c. If salad dressing, cheese sauce, or white sauce was added to your cooked vegetables **AFTER COOKING OR AT THE TABLE**, how much did you usually add?

- ☐ Did not usually add these
- ☐ Less than 1 tablespoon
- ☐ 1 to 3 tablespoons
- ☐ More than 3 tablespoons

48. How often did you eat **sweet peppers** (green, red, or yellow)?

- ☐ NEVER (GO TO QUESTION 49)
- ☐ 1–6 times per year
- ☐ 7–11 times per year
- ☐ 1 time per month
- ☐ 2–3 times per month
- ☐ 1 time per week
- ☐ 2 times per week
- ☐ 3–4 times per week
- ☐ 5–6 times per week
- ☐ 1 time per day
- ☐ 2 or more times per day

48a. Each time you ate **sweet peppers**, how much did you usually eat?

- ☐ Less than ⅛ pepper
- ☐ ⅛ to ¼ pepper
- ☐ More than ¼ pepper

Question 49 appears in the next column

49. Over the past 12 months, did you eat **fresh tomatoes** (including those in salads)?

- ☐ NO (GO TO QUESTION 50)
- ☐ YES

49a. How often did you eat **fresh tomatoes** (including those in salads) **WHEN IN SEASON**?

- ☐ NEVER
- ☐ 1–6 times per season
- ☐ 7–11 times per season
- ☐ 1 time per month
- ☐ 2–3 times per month
- ☐ 1 time per week
- ☐ 2 times per week
- ☐ 3–4 times per week
- ☐ 5–6 times per week
- ☐ 1 time per day
- ☐ 2 or more times per day

49b. How often did you eat **fresh tomatoes** (including those in salads) **DURING THE REST OF THE YEAR**?

- ☐ NEVER
- ☐ 1–6 times per year
- ☐ 7–11 times per year
- ☐ 1 time per month
- ☐ 2–3 times per month
- ☐ 1 time per week
- ☐ 2 times per week
- ☐ 3–4 times per week
- ☐ 5–6 times per week
- ☐ 1 time per day
- ☐ 2 or more times per day

49c. Each time you ate **fresh tomatoes**, how much did you usually eat?

- ☐ Less than ¼ tomato
- ☐ ¼ to ½ tomato
- ☐ More than ½ tomato

50. How often did you eat **lettuce salads** (with or without other vegetables)?

- ☐ NEVER (GO TO QUESTION 51)
- ☐ 1–6 times per year
- ☐ 7–11 times per year
- ☐ 1 time per month
- ☐ 2–3 times per month
- ☐ 1 time per week
- ☐ 2 times per week
- ☐ 3–4 times per week
- ☐ 5–6 times per week
- ☐ 1 time per day
- ☐ 2 or more times per day

Question 51 appears on the next page

This is a sample form. Do not use for scanning.

Over the past 12 months…

50a. Each time you ate **lettuce salads**, how much did you usually eat?

- ☐ Less than ¼ cup
- ☐ ¼ to 1¼ cups
- ☐ More than 1¼ cups

50b. How often did the lettuce salads you ate include **dark green lettuce**?

- ☐ Almost never or never
- ☐ About ¼ of the time
- ☐ About ½ of the time
- ☐ About ¾ of the time
- ☐ Almost always or always

51. How often did you eat **salad dressing** (including low-fat) **on salads**?

☐ NEVER (GO TO QUESTION 52)

☐ 1–6 times per year	☐ 2 times per week
☐ 7–11 times per year	☐ 3–4 times per week
☐ 1 time per month	☐ 5–6 times per week
☐ 2–3 times per month	☐ 1 time per day
☐ 1 time per week	☐ 2 or more times per day

51a. Each time you ate **salad dressing on salads**, how much did you usually eat?

- ☐ Less than 2 tablespoons
- ☐ 2 to 4 tablespoons
- ☐ More than 4 tablespoons

52. How often did you eat **sweet potatoes** or **yams**?

☐ NEVER (GO TO QUESTION 53)

☐ 1–6 times per year	☐ 2 times per week
☐ 7–11 times per year	☐ 3–4 times per week
☐ 1 time per month	☐ 5–6 times per week
☐ 2–3 times per month	☐ 1 time per day
☐ 1 time per week	☐ 2 or more times per day

52a. Each time you ate **sweet potatoes** or **yams**, how much did you usually eat?

- ☐ 1 small potato or less than ¼ cup
- ☐ 1 medium potato or ¼ to ¾ cup
- ☐ 1 large potato or more than ¾ cup

Question 53 appears in the next column

53. How often did you eat **French fries**, **home fries**, **hash browned potatoes**, or **tater tots**?

☐ NEVER (GO TO QUESTION 54)

☐ 1–6 times per year	☐ 2 times per week
☐ 7–11 times per year	☐ 3–4 times per week
☐ 1 time per month	☐ 5–6 times per week
☐ 2–3 times per month	☐ 1 time per day
☐ 1 time per week	☐ 2 or more times per day

53a. Each time you ate **French fries**, **home fries**, **hash browned potatoes**, or **tater tots** how much did you usually eat?

- ☐ Less than 10 fries or less than ½ cup
- ☐ 10 to 25 fries or ½ to 1 cup
- ☐ More than 25 fries or more than 1 cup

54. How often did you eat **potato salad**?

☐ NEVER (GO TO QUESTION 55)

☐ 1–6 times per year	☐ 2 times per week
☐ 7–11 times per year	☐ 3–4 times per week
☐ 1 time per month	☐ 5–6 times per week
☐ 2–3 times per month	☐ 1 time per day
☐ 1 time per week	☐ 2 or more times per day

54a. Each time you ate **potato salad**, how much did you usually eat?

- ☐ Less than ½ cup
- ☐ ½ to 1 cup
- ☐ More than 1 cup

55. How often did you eat **baked, boiled,** or **mashed potatoes**?

☐ NEVER (GO TO QUESTION 56)

☐ 1–6 times per year	☐ 2 times per week
☐ 7–11 times per year	☐ 3–4 times per week
☐ 1 time per month	☐ 5–6 times per week
☐ 2–3 times per month	☐ 1 time per day
☐ 1 time per week	☐ 2 or more times per day

55a. Each time you ate **baked, boiled,** or **mashed potatoes**, how much did you usually eat?

- ☐ 1 small potato or less than ½ cup
- ☐ 1 medium potato or ½ to 1 cup
- ☐ 1 large potato or more than 1 cup

Question 56 appears on the next page

This is a sample form. Do not use for scanning.

Over the past 12 months…

55b. How often was **sour cream** (including low-fat) added to your potatoes, **EITHER IN COOKING OR AT THE TABLE**?

- ☐ Almost never or never (GO TO QUESTION 55d)
- ☐ About ¼ of the time
- ☐ About ½ of the time
- ☐ About ¾ of the time
- ☐ Almost always or always

55c. Each time **sour cream** was added to your potatoes, how much was usually added?

- ☐ Less than 1 tablespoon
- ☐ 1 to 3 tablespoons
- ☐ More than 3 tablespoons

55d. How often was **margarine** (including low-fat) added to your potatoes, **EITHER IN COOKING OR AT THE TABLE**?

- ☐ Almost never or never
- ☐ About ¼ of the time
- ☐ About ½ of the time
- ☐ About ¾ of the time
- ☐ Almost always or always

55e. How often was **butter** (including low-fat) added to your potatoes, **EITHER IN COOKING OR AT THE TABLE**?

- ☐ Almost never or never
- ☐ About ¼ of the time
- ☐ About ½ of the time
- ☐ About ¾ of the time
- ☐ Almost always or always

55f. Each time **margarine** or **butter** was added to your potatoes, how much was usually added?

- ☐ Never added
- ☐ Less than 1 teaspoon
- ☐ 1 to 3 teaspoons
- ☐ More than 3 teaspoons

55g. How often was **cheese** or **cheese sauce** added to your potatoes, **EITHER IN COOKING OR AT THE TABLE**?

- ☐ Almost never or never (GO TO QUESTION 56)
- ☐ About ¼ of the time
- ☐ About ½ of the time
- ☐ About ¾ of the time
- ☐ Almost always or always

Question 56 appears in the next column

55h. Each time **cheese** or **cheese sauce** was added to your potatoes, how much was usually added?

- ☐ Less than 1 tablespoon
- ☐ 1 to 3 tablespoons
- ☐ More than 3 tablespoons

56. How often did you eat **salsa**?

- ☐ NEVER (GO TO QUESTION 57)

☐ 1–6 times per year	☐ 2 times per week
☐ 7–11 times per year	☐ 3–4 times per week
☐ 1 time per month	☐ 5–6 times per week
☐ 2–3 times per month	☐ 1 time per day
☐ 1 time per week	☐ 2 or more times per day

56a. Each time you ate **salsa**, how much did you usually eat?

- ☐ Less than 1 tablespoon
- ☐ 1 to 5 tablespoons
- ☐ More than 5 tablespoons

57. How often did you eat **catsup**?

- ☐ NEVER (GO TO QUESTION 58)

☐ 1–6 times per year	☐ 2 times per week
☐ 7–11 times per year	☐ 3–4 times per week
☐ 1 time per month	☐ 5–6 times per week
☐ 2–3 times per month	☐ 1 time per day
☐ 1 time per week	☐ 2 or more times per day

57a. Each time you ate **catsup**, how much did you usually eat?

- ☐ Less than 1 teaspoon
- ☐ 1 to 6 teaspoons
- ☐ More than 6 teaspoons

58. How often did you eat **stuffing, dressing**, or **dumplings**?

- ☐ NEVER (GO TO QUESTION 59)

☐ 1–6 times per year	☐ 2 times per week
☐ 7–11 times per year	☐ 3–4 times per week
☐ 1 time per month	☐ 5–6 times per week
☐ 2–3 times per month	☐ 1 time per day
☐ 1 time per week	☐ 2 or more times per day

58a. Each time you ate **stuffing, dressing**, or **dumplings**, how much did you usually eat?

- ☐ Less than ½ cup
- ☐ ½ to 1 cup
- ☐ More than 1 cup

Question 59 appears on the next page

Over the past 12 months…

59. How often did you eat **chili**?

- ☐ NEVER (GO TO QUESTION 60)
- ☐ 1–6 times per year
- ☐ 7–11 times per year
- ☐ 1 time per month
- ☐ 2–3 times per month
- ☐ 1 time per week
- ☐ 2 times per week
- ☐ 3–4 times per week
- ☐ 5–6 times per week
- ☐ 1 time per day
- ☐ 2 or more times per day

59a. Each time you ate **chili**, how much did you usually eat?

- ☐ Less than ½ cup
- ☐ ½ to 1¾ cups
- ☐ More than 1¾ cups

60. How often did you eat **Mexican foods** (such as tacos, tostados, burritos, tamales, fajitas, enchiladas, quesadillas, and chimichangas)?

- ☐ NEVER (GO TO QUESTION 61)
- ☐ 1–6 times per year
- ☐ 7–11 times per year
- ☐ 1 time per month
- ☐ 2–3 times per month
- ☐ 1 time per week
- ☐ 2 times per week
- ☐ 3–4 times per week
- ☐ 5–6 times per week
- ☐ 1 time per day
- ☐ 2 or more times per day

60a. Each time you ate **Mexican foods**, how much did you usually eat?

- ☐ Less than 1 taco, burrito, etc.
- ☐ 1 to 2 tacos, burritos, etc.
- ☐ More than 2 tacos, burritos, etc.

61. How often did you eat **cooked dried beans** (such as baked beans, pintos, kidney, blackeyed peas, lima, lentils, soybeans, or refried beans)? *(Please do not include bean soups or chili.)*

- ☐ NEVER (GO TO QUESTION 62)
- ☐ 1–6 times per year
- ☐ 7–11 times per year
- ☐ 1 time per month
- ☐ 2–3 times per month
- ☐ 1 time per week
- ☐ 2 times per week
- ☐ 3–4 times per week
- ☐ 5–6 times per week
- ☐ 1 time per day
- ☐ 2 or more times per day

61a. Each time you ate **beans**, how much did you usually eat?

- ☐ Less than ½ cup
- ☐ ½ to 1 cup
- ☐ More than 1 cup

Question 62 appears in the next column

61b. How often were the beans you ate **refried beans**, **beans prepared with any type of fat**, or **with meat added**?

- ☐ Almost never or never
- ☐ About ¼ of the time
- ☐ About ½ of the time
- ☐ About ¾ of the time
- ☐ Almost always or always

62. How often did you eat **other kinds of vegetables**?

- ☐ NEVER (GO TO QUESTION 63)
- ☐ 1–6 times per year
- ☐ 7–11 times per year
- ☐ 1 time per month
- ☐ 2–3 times per month
- ☐ 1 time per week
- ☐ 2 times per week
- ☐ 3–4 times per week
- ☐ 5–6 times per week
- ☐ 1 time per day
- ☐ 2 or more times per day

62a. Each time you ate **other kinds of vegetables**, how much did you usually eat?

- ☐ Less than ¼ cup
- ☐ ¼ to ½ cup
- ☐ More than ½ cup

63. How often did you eat **rice** or **other cooked grains** (such as bulgur, cracked wheat, or millet)?

- ☐ NEVER (GO TO QUESTION 64)
- ☐ 1–6 times per year
- ☐ 7–11 times per year
- ☐ 1 time per month
- ☐ 2–3 times per month
- ☐ 1 time per week
- ☐ 2 times per week
- ☐ 3–4 times per week
- ☐ 5–6 times per week
- ☐ 1 time per day
- ☐ 2 or more times per day

63a. Each time you ate **rice** or **other cooked grains**, how much did you usually eat?

- ☐ Less than ½ cup
- ☐ ½ to 1½ cups
- ☐ More than 1½ cups

63b. How often was **butter**, **margarine**, or **oil** added to your rice or other cooked grains **IN COOKING OR AT THE TABLE**?

- ☐ Almost never or never
- ☐ About ¼ of the time
- ☐ About ½ of the time
- ☐ About ¾ of the time
- ☐ Almost always or always

Question 64 appears on the next page

This is a sample form. Do not use for scanning.

Over the past 12 months…

64. How often did you eat **pancakes, waffles,** or **French toast**?

- ☐ NEVER (GO TO QUESTION 65)

☐ 1–6 times per year	☐ 2 times per week
☐ 7–11 times per year	☐ 3–4 times per week
☐ 1 time per month	☐ 5–6 times per week
☐ 2–3 times per month	☐ 1 time per day
☐ 1 time per week	☐ 2 or more times per day

64a. Each time you ate **pancakes, waffles,** or **French toast**, how much did you usually eat?

- ☐ Less than 1 medium piece
- ☐ 1 to 3 medium pieces
- ☐ More than 3 medium pieces

64b. How often was **margarine** (including low-fat) added to your pancakes, waffles, or French toast **AFTER COOKING OR AT THE TABLE**?

- ☐ Almost never or never
- ☐ About ¼ of the time
- ☐ About ½ of the time
- ☐ About ¾ of the time
- ☐ Almost always or always

64c. How often was **butter** (including low-fat) added to your pancakes, waffles, or French toast **AFTER COOKING OR AT THE TABLE**?

- ☐ Almost never or never
- ☐ About ¼ of the time
- ☐ About ½ of the time
- ☐ About ¾ of the time
- ☐ Almost always or always

64d. Each time **margarine** or **butter** was added to your pancakes, waffles, or French toast, how much was usually added?

- ☐ Never added
- ☐ Less than 1 teaspoon
- ☐ 1 to 3 teaspoons
- ☐ More than 3 teaspoons

64e. How often was **syrup** added to your pancakes, waffles, or French toast?

- ☐ Almost never or never (GO TO QUESTION 65)
- ☐ About ¼ of the time
- ☐ About ½ of the time
- ☐ About ¾ of the time
- ☐ Almost always or always

Question 65 appears in the next column

64f. Each time **syrup** was added to your pancakes, waffles, or French toast, how much was usually added?

- ☐ Less than 1 tablespoon
- ☐ 1 to 4 tablespoons
- ☐ More than 4 tablespoons

65. How often did you eat **lasagna, stuffed shells, stuffed manicotti, ravioli,** or **tortellini**? *(Please do not include spaghetti or other pasta.)*

- ☐ NEVER (GO TO QUESTION 66)

☐ 1–6 times per year	☐ 2 times per week
☐ 7–11 times per year	☐ 3–4 times per week
☐ 1 time per month	☐ 5–6 times per week
☐ 2–3 times per month	☐ 1 time per day
☐ 1 time per week	☐ 2 or more times per day

65a. Each time you ate **lasagna, stuffed shells, stuffed manicotti, ravioli,** or **tortellini**, how much did you usually eat?

- ☐ Less than 1 cup
- ☐ 1 to 2 cups
- ☐ More than 2 cups

66. How often did you eat **macaroni and cheese**?

- ☐ NEVER (GO TO QUESTION 67)

☐ 1–6 times per year	☐ 2 times per week
☐ 7–11 times per year	☐ 3–4 times per week
☐ 1 time per month	☐ 5–6 times per week
☐ 2–3 times per month	☐ 1 time per day
☐ 1 time per week	☐ 2 or more times per day

66a. Each time you ate **macaroni and cheese**, how much did you usually eat?

- ☐ Less than 1 cup
- ☐ 1 to 1½ cups
- ☐ More than 1½ cups

67. How often did you eat **pasta salad** or **macaroni salad**?

- ☐ NEVER (GO TO QUESTION 68)

☐ 1–6 times per year	☐ 2 times per week
☐ 7–11 times per year	☐ 3–4 times per week
☐ 1 time per month	☐ 5–6 times per week
☐ 2–3 times per month	☐ 1 time per day
☐ 1 time per week	☐ 2 or more times per day

Question 68 appears on the next page

This is a sample form. Do not use for scanning.

Over the past 12 months…

67a. Each time you ate **pasta salad** or **macaroni salad**, how much did you usually eat?

- ☐ Less than ½ cup
- ☐ ½ to 1 cup
- ☐ More than 1 cup

68. Other than the pastas listed in Questions 65, 66, and 67, how often did you eat **pasta, spaghetti**, or **other noodles**?

- ☐ NEVER (GO TO QUESTION 69)
- ☐ 1–6 times per year
- ☐ 7–11 times per year
- ☐ 1 time per month
- ☐ 2–3 times per month
- ☐ 1 time per week
- ☐ 2 times per week
- ☐ 3–4 times per week
- ☐ 5–6 times per week
- ☐ 1 time per day
- ☐ 2 or more times per day

68a. Each time you ate **pasta, spaghetti, or other noodles**, how much did you usually eat?

- ☐ Less than 1 cup
- ☐ 1 to 3 cups
- ☐ More than 3 cups

68b. How often did you eat your pasta, spaghetti, or other noodles with **tomato sauce** or **spaghetti sauce made WITH meat**?

- ☐ Almost never or never
- ☐ About ¼ of the time
- ☐ About ½ of the time
- ☐ About ¾ of the time
- ☐ Almost always or always

68c. How often did you eat your pasta, spaghetti, or other noodles with **tomato sauce** or **spaghetti sauce made WITHOUT meat**?

- ☐ Almost never or never
- ☐ About ¼ of the time
- ☐ About ½ of the time
- ☐ About ¾ of the time
- ☐ Almost always or always

68d. How often did you eat your pasta, spaghetti, or other noodles with **margarine, butter, oil**, or **cream sauce**?

- ☐ Almost never or never
- ☐ About ¼ of the time
- ☐ About ½ of the time
- ☐ About ¾ of the time
- ☐ Almost always or always

Question 69 appears in the next column

69. How often did you eat **bagels** or **English muffins**?

- ☐ NEVER (GO TO INTRODUCTION TO QUESTION 70)
- ☐ 1–6 times per year
- ☐ 7–11 times per year
- ☐ 1 time per month
- ☐ 2–3 times per month
- ☐ 1 time per week
- ☐ 2 times per week
- ☐ 3–4 times per week
- ☐ 5–6 times per week
- ☐ 1 time per day
- ☐ 2 or more times per day

69a. How often were the bagels or English muffins you ate **whole wheat**?

- ☐ Almost never or never
- ☐ About ¼ of the time
- ☐ About ½ of the time
- ☐ About ¾ of the time
- ☐ Almost always or always

69b. Each time you ate **bagels** or **English muffins**, how many did you usually eat?

- ☐ Less than 1 bagel or English muffin
- ☐ 1 bagel or English muffin
- ☐ More than 1 bagel or English muffin

69c. How often was **margarine** (including low-fat) added to your bagels or English muffins?

- ☐ Almost never or never
- ☐ About ¼ of the time
- ☐ About ½ of the time
- ☐ About ¾ of the time
- ☐ Almost always or always

69d. How often was **butter** (including low-fat) added to your bagels or English muffins?

- ☐ Almost never or never
- ☐ About ¼ of the time
- ☐ About ½ of the time
- ☐ About ¾ of the time
- ☐ Almost always or always

69e. Each time **margarine** or **butter** was added to your bagels or English muffins, how much was usually added?

- ☐ Never added
- ☐ Less than 1 teaspoon
- ☐ 1 to 2 teaspoons
- ☐ More than 2 teaspoons

Introduction to Question 70 appears on the next page

This is a sample form. Do not use for scanning.

Over the past 12 months…

69f. How often was **cream cheese** (including low-fat) spread on your bagels or English muffins?

- ☐ Almost never or never (GO TO INTRODUCTION TO QUESTION 70)
- ☐ About ¼ of the time
- ☐ About ½ of the time
- ☐ About ¾ of the time
- ☐ Almost always or always

69g. Each time **cream cheese** was added to your bagels or English muffins, how much was usually added?

- ☐ Less than 1 tablespoon
- ☐ 1 to 2 tablespoons
- ☐ More than 2 tablespoons

The next questions ask about your intake of breads other than bagels or English muffins. First, we will ask about bread you ate as part of sandwiches only. Then we will ask about all other bread you ate.

70. How often did you eat **breads** or **rolls AS PART OF SANDWICHES** (including burger and hot dog rolls)?
(Please do not include fast food sandwiches.)

- ☐ NEVER (GO TO QUESTION 71)
- ☐ 1–6 times per year
- ☐ 7–11 times per year
- ☐ 1 time per month
- ☐ 2–3 times per month
- ☐ 1 time per week
- ☐ 2 times per week
- ☐ 3–4 times per week
- ☐ 5–6 times per week
- ☐ 1 time per day
- ☐ 2 or more times per day

70a. Each time you ate **breads** or **rolls AS PART OF SANDWICHES**, how many did you usually eat?

- ☐ 1 slice or ½ roll
- ☐ 2 slices or 1 roll
- ☐ More than 2 slices or more than 1 roll

70b. How often were the breads or rolls that you used for your sandwiches **white bread** (including burger and hot dog rolls)?

- ☐ Almost never or never
- ☐ About ¼ of the time
- ☐ About ½ of the time
- ☐ About ¾ of the time
- ☐ Almost always or always

Question 71 appears in the next column

70c. How often was **mayonnaise** or **mayonnaise-type dressing** (including low-fat) added to the breads or rolls used for your sandwiches?

- ☐ Almost never or never (GO TO QUESTION 70e)
- ☐ About ¼ of the time
- ☐ About ½ of the time
- ☐ About ¾ of the time
- ☐ Almost always or always

70d. Each time **mayonnaise** or **mayonnaise-type dressing** was added to the breads or rolls used for your sandwiches, how much was usually added?

- ☐ Less than 1 teaspoon
- ☐ 1 to 3 teaspoons
- ☐ More than 3 teaspoons

70e. How often was **margarine** (including low-fat) added to the breads or rolls used for your sandwiches?

- ☐ Almost never or never
- ☐ About ¼ of the time
- ☐ About ½ of the time
- ☐ About ¾ of the time
- ☐ Almost always or always

70f. How often was **butter** (including low-fat) added to the breads or rolls used for your sandwiches?

- ☐ Almost never or never
- ☐ About ¼ of the time
- ☐ About ½ of the time
- ☐ About ¾ of the time
- ☐ Almost always or always

70g. Each time **margarine** or **butter** was added to the breads or rolls used for your sandwiches, how much was usually added?

- ☐ Never added
- ☐ Less than 1 teaspoon
- ☐ 1 to 2 teaspoons
- ☐ More than 2 teaspoons

71. How often did you eat **breads** or **dinner rolls**, **NOT AS PART OF SANDWICHES**?

- ☐ NEVER (GO TO QUESTION 72)
- ☐ 1–6 times per year
- ☐ 7–11 times per year
- ☐ 1 time per month
- ☐ 2–3 times per month
- ☐ 1 time per week
- ☐ 2 times per week
- ☐ 3–4 times per week
- ☐ 5–6 times per week
- ☐ 1 time per day
- ☐ 2 or more times per day

Question 72 appears on the next page

This is a sample form. Do not use for scanning.

Over the past 12 months...

71a. Each time you ate **breads** or **dinner rolls, NOT AS PART OF SANDWICHES**, how much did you usually eat?

- ☐ 1 slice or 1 dinner roll
- ☐ 2 slices or 2 dinner rolls
- ☐ More than 2 slices or 2 dinner rolls

71b. How often were the breads or rolls you ate **white bread**?

- ☐ Almost never or never
- ☐ About ¼ of the time
- ☐ About ½ of the time
- ☐ About ¾ of the time
- ☐ Almost always or always

71c. How often was **margarine** (including low-fat) added to your breads or rolls?

- ☐ Almost never or never
- ☐ About ¼ of the time
- ☐ About ½ of the time
- ☐ About ¾ of the time
- ☐ Almost always or always

71d. How often was **butter** (including low-fat) added to your breads or rolls?

- ☐ Almost never or never
- ☐ About ¼ of the time
- ☐ About ½ of the time
- ☐ About ¾ of the time
- ☐ Almost always or always

71e. Each time **margarine** or **butter** was added to your breads or rolls, how much was usually added?

- ☐ Never added
- ☐ Less than 1 teaspoon
- ☐ 1 to 2 teaspoons
- ☐ More than 2 teaspoons

71f. How often was **cream cheese** (including low-fat) added to your breads or rolls?

- ☐ Almost never or never (GO TO QUESTION 72)
- ☐ About ¼ of the time
- ☐ About ½ of the time
- ☐ About ¾ of the time
- ☐ Almost always or always

Question 72 appears in the next column

71g. Each time **cream cheese** was added to your breads or rolls, how much was usually added?

- ☐ Less than 1 tablespoon
- ☐ 1 to 2 tablespoons
- ☐ More than 2 tablespoons

72. How often did you eat **jam, jelly,** or **honey** on bagels, muffins, bread, rolls, or crackers?

- ☐ NEVER (GO TO QUESTION 73)

☐ 1–6 times per year	☐ 2 times per week
☐ 7–11 times per year	☐ 3–4 times per week
☐ 1 time per month	☐ 5–6 times per week
☐ 2–3 times per month	☐ 1 time per day
☐ 1 time per week	☐ 2 or more times per day

72a. Each time you ate **jam, jelly,** or **honey**, how much did you usually eat?

- ☐ Less than 1 teaspoon
- ☐ 1 to 3 teaspoons
- ☐ More than 3 teaspoons

73. How often did you eat **peanut butter** or **other nut butter**?

- ☐ NEVER (GO TO QUESTION 74)

☐ 1–6 times per year	☐ 2 times per week
☐ 7–11 times per year	☐ 3–4 times per week
☐ 1 time per month	☐ 5–6 times per week
☐ 2–3 times per month	☐ 1 time per day
☐ 1 time per week	☐ 2 or more times per day

73a. Each time you ate **peanut butter** or **other nut butter**, how much did you usually eat?

- ☐ Less than 1 tablespoon
- ☐ 1 to 2 tablespoons
- ☐ More than 2 tablespoons

74. How often did you eat **roast beef** or **steak IN SANDWICHES**?

- ☐ NEVER (GO TO QUESTION 75)

☐ 1–6 times per year	☐ 2 times per week
☐ 7–11 times per year	☐ 3–4 times per week
☐ 1 time per month	☐ 5–6 times per week
☐ 2–3 times per month	☐ 1 time per day
☐ 1 time per week	☐ 2 or more times per day

Question 75 appears on the next page

This is a sample form. Do not use for scanning.

Over the <u>past 12 months</u>...

74a. Each time you ate **roast beef** or **steak IN SANDWICHES**, how much did you usually eat?

- ☐ Less than 1 slice or less than 2 ounces
- ☐ 1 to 2 slices or 2 to 4 ounces
- ☐ More than 2 slices or more than 4 ounces

75. How often did you eat **turkey** or **chicken COLD CUTS** (such as loaf, luncheon meat, turkey ham, turkey salami, or turkey pastrami)? *(We will ask about other turkey or chicken later.)*

- ☐ NEVER (GO TO QUESTION 76)

☐ 1–6 times per year	☐ 2 times per week
☐ 7–11 times per year	☐ 3–4 times per week
☐ 1 time per month	☐ 5–6 times per week
☐ 2–3 times per month	☐ 1 time per day
☐ 1 time per week	☐ 2 or more times per day

75a. Each time you ate **turkey or chicken COLD CUTS**, how much did you usually eat?

- ☐ Less than 1 slice
- ☐ 1 to 3 slices
- ☐ More than 3 slices

76. How often did you eat **luncheon** or **deli-style ham**? *(We will ask about other ham later.)*

- ☐ NEVER (GO TO QUESTION 77)

☐ 1–6 times per year	☐ 2 times per week
☐ 7–11 times per year	☐ 3–4 times per week
☐ 1 time per month	☐ 5–6 times per week
☐ 2–3 times per month	☐ 1 time per day
☐ 1 time per week	☐ 2 or more times per day

76a. Each time you ate **luncheon** or **deli-style ham**, how much did you usually eat?

- ☐ Less than 1 slice
- ☐ 1 to 3 slices
- ☐ More than 3 slices

76b. How often was the luncheon or deli-style ham you ate **light**, **low-fat**, or **fat-free**?

- ☐ Almost never or never
- ☐ About ¼ of the time
- ☐ About ½ of the time
- ☐ About ¾ of the time
- ☐ Almost always or always

Question 77 appears in the next column

77. How often did you eat **other cold cuts** or **luncheon meats** (such as bologna, salami, corned beef, pastrami, or others, including low-fat)? *(Please do not include ham, turkey, or chicken cold cuts.)*

- ☐ NEVER (GO TO QUESTION 78)

☐ 1–6 times per year	☐ 2 times per week
☐ 7–11 times per year	☐ 3–4 times per week
☐ 1 time per month	☐ 5–6 times per week
☐ 2–3 times per month	☐ 1 time per day
☐ 1 time per week	☐ 2 or more times per day

77a. Each time you ate **other cold cuts** or **luncheon meats**, how much did you usually eat?

- ☐ Less than 1 slice
- ☐ 1 to 3 slices
- ☐ More than 3 slices

77b. How often were the other cold cuts or luncheon meats you ate **light**, **low-fat**, or **fat-free**? *(Please do not include ham, turkey, or chicken cold cuts.)*

- ☐ Almost never or never
- ☐ About ¼ of the time
- ☐ About ½ of the time
- ☐ About ¾ of the time
- ☐ Almost always or always

78. How often did you eat **canned tuna** (including in salads, sandwiches, or casseroles)?

- ☐ NEVER (GO TO QUESTION 79)

☐ 1–6 times per year	☐ 2 times per week
☐ 7–11 times per year	☐ 3–4 times per week
☐ 1 time per month	☐ 5–6 times per week
☐ 2–3 times per month	☐ 1 time per day
☐ 1 time per week	☐ 2 or more times per day

78a. Each time you ate **canned tuna**, how much did you usually eat?

- ☐ Less than ¼ cup or less than 2 ounces
- ☐ ¼ to ½ cup or 2 to 3 ounces
- ☐ More than ½ cup or more than 3 ounces

78b. How often was the canned tuna you ate **water-packed**?

- ☐ Almost never or never
- ☐ About ¼ of the time
- ☐ About ½ of the time
- ☐ About ¾ of the time
- ☐ Almost always or always

Question 79 appears on the next page

This is a sample form. Do not use for scanning.

Over the past 12 months…

78c. How often was the canned tuna you ate **prepared with mayonnaise** or **other dressing** (including low-fat)?

- ☐ Almost never or never
- ☐ About ¼ of the time
- ☐ About ½ of the time
- ☐ About ¾ of the time
- ☐ Almost always or always

79. How often did you eat **GROUND chicken** or **turkey**? *(We will ask about other chicken and turkey later.)*

- ☐ NEVER (GO TO QUESTION 80)

- ☐ 1–6 times per year
- ☐ 7–11 times per year
- ☐ 1 time per month
- ☐ 2–3 times per month
- ☐ 1 time per week
- ☐ 2 times per week
- ☐ 3–4 times per week
- ☐ 5–6 times per week
- ☐ 1 time per day
- ☐ 2 or more times per day

79a. Each time you ate **GROUND chicken** or **turkey**, how much did you usually eat?

- ☐ Less than 2 ounces or less than ½ cup
- ☐ 2 to 4 ounces or ½ to 1 cup
- ☐ More than 4 ounces or more than 1 cup

80. How often did you eat **beef hamburgers** or **cheeseburgers** from a **FAST FOOD** or **OTHER RESTAURANT**?

- ☐ NEVER (GO TO QUESTION 81)

- ☐ 1–6 times per year
- ☐ 7–11 times per year
- ☐ 1 time per month
- ☐ 2–3 times per month
- ☐ 1 time per week
- ☐ 2 times per week
- ☐ 3–4 times per week
- ☐ 5–6 times per week
- ☐ 1 time per day
- ☐ 2 or more times per day

80a. Each time you ate **beef hamburgers** or **cheeseburgers** from a **FAST FOOD** or **OTHER RESTAURANT**, what size did you usually eat?

- ☐ Small hamburger (such as a regular Burger King or McDonald's Hamburger)
- ☐ Medium (such as McDonald's or Burger King Double Burger or Cheeseburger)
- ☐ Large (such as Burger King Whopper or Double Whopper or a McDonald's Double Quarter Pounder)

Question 81 appears in the next column

80b. Each time you ate **beef hamburgers** or **cheeseburgers** from a **FAST FOOD** or **OTHER RESTAURANT**, how much did you usually eat?

- ☐ Less than 1 burger
- ☐ 1 burger
- ☐ More than 1 burger

80c. How often did you have **cheeseburgers** rather than **hamburgers**?

- ☐ Almost never or never
- ☐ About ¼ of the time
- ☐ About ½ of the time
- ☐ About ¾ of the time
- ☐ Almost always or always

81. How often did you eat **beef hamburgers** or **cheeseburgers** that were **NOT FROM A FAST FOOD** or **OTHER RESTAURANT**?

- ☐ NEVER (GO TO QUESTION 82)

- ☐ 1–6 times per year
- ☐ 7–11 times per year
- ☐ 1 time per month
- ☐ 2–3 times per month
- ☐ 1 time per week
- ☐ 2 times per week
- ☐ 3–4 times per week
- ☐ 5–6 times per week
- ☐ 1 time per day
- ☐ 2 or more times per day

81a. Each time you ate **beef hamburgers** or **cheeseburgers** that were **NOT FROM A FAST FOOD** or **OTHER RESTAURANT**, how much did you usually eat?

- ☐ Less than 1 patty or less than 2 ounces
- ☐ 1 patty or 2 to 4 ounces
- ☐ More than 1 patty or more than 4 ounces

81b. How often were these beef hamburgers or cheeseburgers made with **lean ground beef**?

- ☐ Almost never or never
- ☐ About ¼ of the time
- ☐ About ½ of the time
- ☐ About ¾ of the time
- ☐ Almost always or always

82. How often did you eat **ground beef in mixtures** (such as meatballs, casseroles, chili, or meatloaf)?

- ☐ NEVER (GO TO QUESTION 83)

- ☐ 1–6 times per year
- ☐ 7–11 times per year
- ☐ 1 time per month
- ☐ 2–3 times per month
- ☐ 1 time per week
- ☐ 2 times per week
- ☐ 3–4 times per week
- ☐ 5–6 times per week
- ☐ 1 time per day
- ☐ 2 or more times per day

Question 83 appears on the next page

This is a sample form. Do not use for scanning.

Over the past 12 months…

82a. Each time you ate **ground beef in mixtures**, how much did you usually eat?

- ☐ Less than 3 ounces or less than ½ cup
- ☐ 3 to 8 ounces or ½ to 1 cup
- ☐ More than 8 ounces or more than 1 cup

83. How often did you eat **hot dogs** or **frankfurters**? *(Please do not include sausages or vegetarian hot dogs.)*

- ☐ NEVER (GO TO QUESTION 84)

☐ 1–6 times per year	☐ 2 times per week
☐ 7–11 times per year	☐ 3–4 times per week
☐ 1 time per month	☐ 5–6 times per week
☐ 2–3 times per month	☐ 1 time per day
☐ 1 time per week	☐ 2 or more times per day

83a. Each time you ate **hot dogs** or **frankfurters**, how many did you usually eat?

- ☐ Less than 1 hot dog
- ☐ 1 to 2 hot dogs
- ☐ More than 2 hot dogs

83b. How often were the hot dogs or frankfurters you ate **light** or **low-fat**?

- ☐ Almost never or never
- ☐ About ¼ of the time
- ☐ About ½ of the time
- ☐ About ¾ of the time
- ☐ Almost always or always

84. How often did you eat **beef mixtures** (such as beef stew, beef pot pie, beef and noodles, or beef and vegetables)?

- ☐ NEVER (GO TO QUESTION 85)

☐ 1–6 times per year	☐ 2 times per week
☐ 7–11 times per year	☐ 3–4 times per week
☐ 1 time per month	☐ 5–6 times per week
☐ 2–3 times per month	☐ 1 time per day
☐ 1 time per week	☐ 2 or more times per day

84a. Each time you ate **beef mixtures**, how much did you usually eat?

- ☐ Less than 1 cup
- ☐ 1 to 2 cups
- ☐ More than 2 cups

Question 85 appears in the next column

85. How often did you eat **roast beef** or **pot roast**? *(Please do not include roast beef or pot roast in sandwiches.)*

- ☐ NEVER (GO TO QUESTION 86)

☐ 1–6 times per year	☐ 2 times per week
☐ 7–11 times per year	☐ 3–4 times per week
☐ 1 time per month	☐ 5–6 times per week
☐ 2–3 times per month	☐ 1 time per day
☐ 1 time per week	☐ 2 or more times per day

85a. Each time you ate **roast beef** or **pot roast**, how much did you usually eat?

- ☐ Less than 2 ounces
- ☐ 2 to 5 ounces
- ☐ More than 5 ounces

86. How often did you eat **steak** (beef)? (*Please do not include steak in sandwiches*)

- ☐ NEVER (GO TO QUESTION 87)

☐ 1–6 times per year	☐ 2 times per week
☐ 7–11 times per year	☐ 3–4 times per week
☐ 1 time per month	☐ 5–6 times per week
☐ 2–3 times per month	☐ 1 time per day
☐ 1 time per week	☐ 2 or more times per day

86a. Each time you ate **steak** (beef), how much did you usually eat?

- ☐ Less than 3 ounces
- ☐ 3 to 7 ounces
- ☐ More than 7 ounces

86b. How often was the steak you ate **lean steak**?

- ☐ Almost never or never
- ☐ About ¼ of the time
- ☐ About ½ of the time
- ☐ About ¾ of the time
- ☐ Almost always or always

87. How often did you eat **pork** or **beef spareribs**?

- ☐ NEVER (GO TO QUESTION 88)

☐ 1–6 times per year	☐ 2 times per week
☐ 7–11 times per year	☐ 3–4 times per week
☐ 1 time per month	☐ 5–6 times per week
☐ 2–3 times per month	☐ 1 time per day
☐ 1 time per week	☐ 2 or more times per day

Question 88 appears on the next page

This is a sample form. Do not use for scanning.

Over the past 12 months...

87a. Each time you ate **pork** or **beef spareribs**, how much did you usually eat?

- ☐ Less than 4 ribs
- ☐ 4 to 12 ribs
- ☐ More than 12 ribs

88. How often did you eat **roast turkey, turkey cutlets**, or **turkey nuggets** (including in sandwiches)?

- ☐ NEVER (GO TO QUESTION 89)

- ☐ 1–6 times per year
- ☐ 7–11 times per year
- ☐ 1 time per month
- ☐ 2–3 times per month
- ☐ 1 time per week
- ☐ 2 times per week
- ☐ 3–4 times per week
- ☐ 5–6 times per week
- ☐ 1 time per day
- ☐ 2 or more times per day

88a. Each time you ate **roast turkey, turkey cutlets**, or **turkey nuggets**, how much did you usually eat? *(Please note: 4 to 8 turkey nuggets = 3 ounces.)*

- ☐ Less than 2 ounces
- ☐ 2 to 4 ounces
- ☐ More than 4 ounces

89. How often did you eat **chicken mixtures** (such as salads, sandwiches, casseroles, stews, or other mixtures)?

- ☐ NEVER (GO TO QUESTION 90)

- ☐ 1–6 times per year
- ☐ 7–11 times per year
- ☐ 1 time per month
- ☐ 2–3 times per month
- ☐ 1 time per week
- ☐ 2 times per week
- ☐ 3–4 times per week
- ☐ 5–6 times per week
- ☐ 1 time per day
- ☐ 2 or more times per day

89a. Each time you ate **chicken mixtures**, how much did you usually eat?

- ☐ Less than ½ cup
- ☐ ½ to 1½ cups
- ☐ More than 1½ cups

Question 90 appears in the next column

90. How often did you eat **baked, broiled, roasted, stewed**, or **fried chicken** (including nuggets)? *(Please do not include chicken in mixtures.)*

- ☐ NEVER (GO TO QUESTION 91)

- ☐ 1–6 times per year
- ☐ 7–11 times per year
- ☐ 1 time per month
- ☐ 2–3 times per month
- ☐ 1 time per week
- ☐ 2 times per week
- ☐ 3–4 times per week
- ☐ 5–6 times per week
- ☐ 1 time per day
- ☐ 2 or more times per day

90a. Each time you ate **baked, broiled, roasted, stewed**, or **fried chicken** (including nuggets), how much did you usually eat?

- ☐ Less than 2 drumsticks or wings, less than 1 breast or thigh, or less than 4 nuggets
- ☐ 2 drumsticks or wings, 1 breast or thigh, or 4 to 8 nuggets
- ☐ More than 2 drumsticks or wings, more than 1 breast or thigh, or more than 8 nuggets

90b. How often was the chicken you ate **fried chicken** (including deep fried) or **chicken nuggets**?

- ☐ Almost never or never
- ☐ About ¼ of the time
- ☐ About ½ of the time
- ☐ About ¾ of the time
- ☐ Almost always or always

90c. How often was the chicken you ate **WHITE meat**?

- ☐ Almost never or never
- ☐ About ¼ of the time
- ☐ About ½ of the time
- ☐ About ¾ of the time
- ☐ Almost always or always

90d. How often did you eat chicken **WITH skin**?

- ☐ Almost never or never
- ☐ About ¼ of the time
- ☐ About ½ of the time
- ☐ About ¾ of the time
- ☐ Almost always or always

91. How often did you eat **baked ham** or **ham steak**?

- ☐ NEVER (GO TO QUESTION 92)

- ☐ 1–6 times per year
- ☐ 7–11 times per year
- ☐ 1 time per month
- ☐ 2–3 times per month
- ☐ 1 time per week
- ☐ 2 times per week
- ☐ 3–4 times per week
- ☐ 5–6 times per week
- ☐ 1 time per day
- ☐ 2 or more times per day

Question 92 appears on the next page

This is a sample form. Do not use for scanning.

Over the past 12 months...

91a. Each time you ate **baked ham** or **ham steak**, how much did you usually eat?

- ☐ Less than 1 ounce
- ☐ 1 to 3 ounces
- ☐ More than 3 ounces

92. How often did you eat **pork** (including chops, roasts, and in mixed dishes)? *(Please do not include ham, ham steak, or sausage.)*

- ☐ NEVER (GO TO QUESTION 93)

☐ 1–6 times per year	☐ 2 times per week
☐ 7–11 times per year	☐ 3–4 times per week
☐ 1 time per month	☐ 5–6 times per week
☐ 2–3 times per month	☐ 1 time per day
☐ 1 time per week	☐ 2 or more times per day

92a. Each time you ate **pork**, how much did you usually eat?

- ☐ Less than 2 ounces or less than 1 chop
- ☐ 2 to 5 ounces or 1 chop
- ☐ More than 5 ounces or more than 1 chop

93. How often did you eat **gravy** on meat, chicken, potatoes, rice, etc.?

- ☐ NEVER (GO TO QUESTION 94)

☐ 1–6 times per year	☐ 2 times per week
☐ 7–11 times per year	☐ 3–4 times per week
☐ 1 time per month	☐ 5–6 times per week
☐ 2–3 times per month	☐ 1 time per day
☐ 1 time per week	☐ 2 or more times per day

93a. Each time you ate **gravy** on meat, chicken, potatoes, rice, etc., how much did you usually eat?

- ☐ Less than ⅛ cup
- ☐ ⅛ to ½ cup
- ☐ More than ½ cup

94. How often did you eat **liver** (all kinds) or **liverwurst**?

- ☐ NEVER (GO TO QUESTION 95)

☐ 1–6 times per year	☐ 2 times per week
☐ 7–11 times per year	☐ 3–4 times per week
☐ 1 time per month	☐ 5–6 times per week
☐ 2–3 times per month	☐ 1 time per day
☐ 1 time per week	☐ 2 or more times per day

Question 95 appears in the next column

94a. Each time you ate **liver** or **liverwurst**, how much did you usually eat?

- ☐ Less than 1 ounce
- ☐ 1 to 4 ounces
- ☐ More than 4 ounces

95. How often did you eat **bacon** (including low-fat)?

- ☐ NEVER (GO TO QUESTION 96)

☐ 1–6 times per year	☐ 2 times per week
☐ 7–11 times per year	☐ 3–4 times per week
☐ 1 time per month	☐ 5–6 times per week
☐ 2–3 times per month	☐ 1 time per day
☐ 1 time per week	☐ 2 or more times per day

95a. Each time you ate **bacon**, how much did you usually eat?

- ☐ Fewer than 2 slices
- ☐ 2 to 3 slices
- ☐ More than 3 slices

95b. How often was the bacon you ate **light, low-fat**, or **lean**?

- ☐ Almost never or never
- ☐ About ¼ of the time
- ☐ About ½ of the time
- ☐ About ¾ of the time
- ☐ Almost always or always

96. How often did you eat **sausage** (including low-fat)?

- ☐ NEVER (GO TO QUESTION 97)

☐ 1–6 times per year	☐ 2 times per week
☐ 7–11 times per year	☐ 3–4 times per week
☐ 1 time per month	☐ 5–6 times per week
☐ 2–3 times per month	☐ 1 time per day
☐ 1 time per week	☐ 2 or more times per day

96a. Each time you ate **sausage**, how much did you usually eat?

- ☐ Less than 1 patty or 2 links
- ☐ 1 to 3 patties or 2 to 5 links
- ☐ More than 3 patties or 5 links

96b. How often was the sausage you ate **light, low-fat**, or **lean**?

- ☐ Almost never or never
- ☐ About ¼ of the time
- ☐ About ½ of the time
- ☐ About ¾ of the time
- ☐ Almost always or always

Question 97 appears on the next page

This is a sample form. Do not use for scanning.

Over the past 12 months…

97. How often did you eat **fried shellfish** (such as crab, lobster, shrimp)?

- ☐ NEVER (GO TO QUESTION 98)
- ☐ 1–6 times per year
- ☐ 7–11 times per year
- ☐ 1 time per month
- ☐ 2–3 times per month
- ☐ 1 time per week
- ☐ 2 times per week
- ☐ 3–4 times per week
- ☐ 5–6 times per week
- ☐ 1 time per day
- ☐ 2 or more times per day

97a. Each time you ate **fried shellfish**, how much did you usually eat?

- ☐ Less than 2 ounces
- ☐ 2 to 4 ounces
- ☐ More than 4 ounces

98. How often did you eat **shellfish** (such as crab, lobster, shrimp) **that was NOT FRIED**?

- ☐ NEVER (GO TO QUESTION 99)
- ☐ 1–6 times per year
- ☐ 7–11 times per year
- ☐ 1 time per month
- ☐ 2–3 times per month
- ☐ 1 time per week
- ☐ 2 times per week
- ☐ 3–4 times per week
- ☐ 5–6 times per week
- ☐ 1 time per day
- ☐ 2 or more times per day

98a. Each time you ate **shellfish that was NOT FRIED**, how much did you usually eat?

- ☐ Less than 1 ounce
- ☐ 1 to 4 ounces
- ☐ More than 4 ounces

99. How often did you eat **salmon, fresh tuna or trout**?

- ☐ NEVER (GO TO QUESTION 100)
- ☐ 1–6 times per year
- ☐ 7–11 times per year
- ☐ 1 time per month
- ☐ 2–3 times per month
- ☐ 1 time per week
- ☐ 2 times per week
- ☐ 3–4 times per week
- ☐ 5–6 times per week
- ☐ 1 time per day
- ☐ 2 or more times per day

99a. Each time you ate **salmon, fresh tuna or trout**, how much did you usually eat?

- ☐ Less than 2 ounces
- ☐ 2 to 6 ounces
- ☐ More than 6 ounces

Question 100 appears in the next column

100. How often did you eat **fish sticks** or other **fried fish** (not including shellfish)?

- ☐ NEVER (GO TO QUESTION 101)
- ☐ 1–6 times per year
- ☐ 7–11 times per year
- ☐ 1 time per month
- ☐ 2–3 times per month
- ☐ 1 time per week
- ☐ 2 times per week
- ☐ 3–4 times per week
- ☐ 5–6 times per week
- ☐ 1 time per day
- ☐ 2 or more times per day

100a. Each time you ate **fish sticks** or **other fried fish**, how much did you usually eat?

- ☐ Less than 2 ounces or less than 1 fillet
- ☐ 2 to 7 ounces or 1 fillet
- ☐ More than 7 ounces or more than 1 fillet

101. How often did you eat **other fish that was NOT FRIED** (not including shellfish)?

- ☐ NEVER (GO TO INTRODUCTION TO QUESTION 102)
- ☐ 1–6 times per year
- ☐ 7–11 times per year
- ☐ 1 time per month
- ☐ 2–3 times per month
- ☐ 1 time per week
- ☐ 2 times per week
- ☐ 3–4 times per week
- ☐ 5–6 times per week
- ☐ 1 time per day
- ☐ 2 or more times per day

101a. Each time you ate **other fish that was NOT FRIED**, how much did you usually eat?

- ☐ Less than 2 ounces or less than 1 fillet
- ☐ 2 to 5 ounces or 1 fillet
- ☐ More than 5 ounces or more than 1 fillet

Now think about all the meat, poultry, and fish you ate in the past 12 months and how they were prepared.

102. How often was **oil, butter, margarine, or other fat** used to **FRY, SAUTE, BASTE, OR MARINATE** any meat, poultry, or fish you ate? *(Please do not include deep frying.)*

- ☐ NEVER (GO TO QUESTION 103)
- ☐ 1–6 times per year
- ☐ 7–11 times per year
- ☐ 1 time per month
- ☐ 2–3 times per month
- ☐ 1 time per week
- ☐ 2 times per week
- ☐ 3–4 times per week
- ☐ 5–6 times per week
- ☐ 1 time per day
- ☐ 2 or more times per day

Question 103 appears on the next page

This is a sample form. Do not use for scanning.

Over the past 12 months…

102a. Which of the following **fats** were regularly used to prepare your meat, poultry, or fish? ***(Mark all that apply.)***

- ☐ Margarine (including low-fat)
- ☐ Butter (including low-fat)
- ☐ Lard, fatback, or bacon fat
- ☐ Olive oil
- ☐ Corn oil
- ☐ Canola or rapeseed oil
- ☐ Oil spray (such as Pam or others)
- ☐ Other kinds of oils
- ☐ None of the above

103. How often did you eat **tofu, soy burgers**, or **soy meat-substitutes**?

- ☐ NEVER (GO TO QUESTION 104)
- ☐ 1–6 times per year
- ☐ 7–11 times per year
- ☐ 1 time per month
- ☐ 2–3 times per month
- ☐ 1 time per week
- ☐ 2 times per week
- ☐ 3–4 times per week
- ☐ 5–6 times per week
- ☐ 1 time per day
- ☐ 2 or more times per day

103a. Each time you ate **tofu, soy burgers**, or **soy meat-substitutes**, how much did you usually eat?

- ☐ Less than ¼ cup or less than 2 ounces
- ☐ ¼ to ½ cup or 2 to 4 ounces
- ☐ More than ½ cup or more than 4 ounces

104. Over the past 12 months, did you eat **soups**?

- ☐ NO (GO TO QUESTION 105)
- ☐ YES

104a. How often did you eat **soup IN THE WINTER**?

- ☐ NEVER
- ☐ 1–6 times per winter
- ☐ 7–11 times per winter
- ☐ 1 time per month
- ☐ 2–3 times per month
- ☐ 1 time per week
- ☐ 2 times per week
- ☐ 3–4 times per week
- ☐ 5–6 times per week
- ☐ 1 time per day
- ☐ 2 or more times per day

104b. How often did you eat **soup DURING THE REST OF THE YEAR**?

- ☐ NEVER
- ☐ 1–6 times per year
- ☐ 7–11 times per year
- ☐ 1 time per month
- ☐ 2–3 times per month
- ☐ 1 time per week
- ☐ 2 times per week
- ☐ 3–4 times per week
- ☐ 5–6 times per week
- ☐ 1 time per day
- ☐ 2 or more times per day

Question 105 appears in the next column

104c. Each time you ate **soup**, how much did you usually eat?

- ☐ Less than 1 cup
- ☐ 1 to 2 cups
- ☐ More than 2 cups

104d. How often were the soups you ate **bean soups**?

- ☐ Almost never or never
- ☐ About ¼ of the time
- ☐ About ½ of the time
- ☐ About ¾ of the time
- ☐ Almost always or always

104e. How often were the soups you ate **cream soups** (including chowders)?

- ☐ Almost never or never
- ☐ About ¼ of the time
- ☐ About ½ of the time
- ☐ About ¾ of the time
- ☐ Almost always or always

104f. How often were the soups you ate **tomato** or **vegetable soups**?

- ☐ Almost never or never
- ☐ About ¼ of the time
- ☐ About ½ of the time
- ☐ About ¾ of the time
- ☐ Almost always or always

104g. How often were the soups you ate **broth soups** (including chicken) **with** or **without noodles** or **rice**?

- ☐ Almost never or never
- ☐ About ¼ of the time
- ☐ About ½ of the time
- ☐ About ¾ of the time
- ☐ Almost always or always

105. How often did you eat **pizza**?

- ☐ NEVER (GO TO QUESTION 106)
- ☐ 1–6 times per year
- ☐ 7–11 times per year
- ☐ 1 time per month
- ☐ 2–3 times per month
- ☐ 1 time per week
- ☐ 2 times per week
- ☐ 3–4 times per week
- ☐ 5–6 times per week
- ☐ 1 time per day
- ☐ 2 or more times per day

Question 106 appears on the next page

This is a sample form. Do not use for scanning.

Over the past 12 months…

105a. Each time you ate **pizza**, how much did you usually eat?

- ☐ Less than 1 slice or less than 1 mini pizza
- ☐ 1 to 3 slices or 1 mini pizza
- ☐ More than 3 slices or more than 1 mini pizza

105b. How often did you eat pizza with **pepperoni, sausage**, or **other meat**?

- ☐ Almost never or never
- ☐ About ¼ of the time
- ☐ About ½ of the time
- ☐ About ¾ of the time
- ☐ Almost always or always

106. How often did you eat **crackers**?

- ☐ NEVER (GO TO QUESTION 107)

☐ 1–6 times per year	☐ 2 times per week
☐ 7–11 times per year	☐ 3–4 times per week
☐ 1 time per month	☐ 5–6 times per week
☐ 2–3 times per month	☐ 1 time per day
☐ 1 time per week	☐ 2 or more times per day

106a. Each time you ate **crackers**, how many did you usually eat?

- ☐ Fewer than 4 crackers
- ☐ 4 to 10 crackers
- ☐ More than 10 crackers

107. How often did you eat **corn bread** or **corn muffins**?

- ☐ NEVER (GO TO QUESTION 108)

☐ 1–6 times per year	☐ 2 times per week
☐ 7–11 times per year	☐ 3–4 times per week
☐ 1 time per month	☐ 5–6 times per week
☐ 2–3 times per month	☐ 1 time per day
☐ 1 time per week	☐ 2 or more times per day

107a. Each time you ate **corn bread** or **corn muffins**, how much did you usually eat?

- ☐ Less than 1 piece or muffin
- ☐ 1 to 2 pieces or muffins
- ☐ More than 2 pieces or muffins

Question 108 appears in the next column

108. How often did you eat **biscuits**?

- ☐ NEVER (GO TO QUESTION 109)

☐ 1–6 times per year	☐ 2 times per week
☐ 7–11 times per year	☐ 3–4 times per week
☐ 1 time per month	☐ 5–6 times per week
☐ 2–3 times per month	☐ 1 time per day
☐ 1 time per week	☐ 2 or more times per day

108a. Each time you ate **biscuits**, how many did you usually eat?

- ☐ Fewer than 1 biscuit
- ☐ 1 to 2 biscuits
- ☐ More than 2 biscuits

109. How often did you eat **potato chips** (including low-fat, fat-free, or low-salt)?

- ☐ NEVER (GO TO QUESTION 110)

☐ 1–6 times per year	☐ 2 times per week
☐ 7–11 times per year	☐ 3–4 times per week
☐ 1 time per month	☐ 5–6 times per week
☐ 2–3 times per month	☐ 1 time per day
☐ 1 time per week	☐ 2 or more times per day

109a. Each time you ate **potato chips**, how much did you usually eat?

- ☐ Fewer than 10 chips or less than 1 cup
- ☐ 10 to 25 chips or 1 to 2 cups
- ☐ More than 25 chips or more than 2 cups

109b. How often were the potato chips you ate **fat-free**? *(Please do not include reduced-fat chips.)*

- ☐ Almost never or never
- ☐ About ¼ of the time
- ☐ About ½ of the time
- ☐ About ¾ of the time
- ☐ Almost always or always

110. How often did you eat **corn chips or tortilla chips** (including low-fat, fat-free, or low-salt)?

- ☐ NEVER (GO TO QUESTION 111)

☐ 1–6 times per year	☐ 2 times per week
☐ 7–11 times per year	☐ 3–4 times per week
☐ 1 time per month	☐ 5–6 times per week
☐ 2–3 times per month	☐ 1 time per day
☐ 1 time per week	☐ 2 or more times per day

Question 111 appears on the next page

This is a sample form. Do not use for scanning.

Over the past 12 months…

110a. Each time you ate **corn chips**, how much did you usually eat?

- ☐ Fewer than 10 chips or less than 1 cup
- ☐ 10 to 25 chips or 1 to 1½ cups
- ☐ More than 25 chips or more than 1½ cups

110b. How often were the corn chips or tortilla chips you ate **fat-free**? *(Please do not include reduced-fat chips.)*

- ☐ Almost never or never
- ☐ About ¼ of the time
- ☐ About ½ of the time
- ☐ About ¾ of the time
- ☐ Almost always or always

111. How often did you eat **popcorn** (including low-fat)?

- ☐ NEVER (GO TO QUESTION 112)
- ☐ 1–6 times per year
- ☐ 7–11 times per year
- ☐ 1 time per month
- ☐ 2–3 times per month
- ☐ 1 time per week
- ☐ 2 times per week
- ☐ 3–4 times per week
- ☐ 5–6 times per week
- ☐ 1 time per day
- ☐ 2 or more times per day

111a. Each time you ate **popcorn**, how much did you usually eat?

- ☐ Less than 2 cups, popped
- ☐ 2 to 5 cups, popped
- ☐ More than 5 cups, popped

112. How often did you eat **pretzels**?

- ☐ NEVER (GO TO QUESTION 113)
- ☐ 1–6 times per year
- ☐ 7–11 times per year
- ☐ 1 time per month
- ☐ 2–3 times per month
- ☐ 1 time per week
- ☐ 2 times per week
- ☐ 3–4 times per week
- ☐ 5–6 times per week
- ☐ 1 time per day
- ☐ 2 or more times per day

112a. Each time you ate **pretzels**, how many did you usually eat?

- ☐ Fewer than 5 average twists
- ☐ 5 to 20 average twists
- ☐ More than 20 average twists

Question 113 appears in the next column

113. How often did you eat **peanuts, walnuts, seeds**, or **other nuts**?

- ☐ NEVER (GO TO QUESTION 114)
- ☐ 1–6 times per year
- ☐ 7–11 times per year
- ☐ 1 time per month
- ☐ 2–3 times per month
- ☐ 1 time per week
- ☐ 2 times per week
- ☐ 3–4 times per week
- ☐ 5–6 times per week
- ☐ 1 time per day
- ☐ 2 or more times per day

113a. Each time you ate **peanuts, walnuts, seeds,** or **other nuts**, how much did you usually eat?

- ☐ Less than ¼ cup
- ☐ ¼ to ½ cup
- ☐ More than ½ cup

114. How often did you eat **energy, high-protein**, or **breakfast bars** (such as Power Bars, Balance, Clif, or others)?

- ☐ NEVER (GO TO QUESTION 115)
- ☐ 1–6 times per year
- ☐ 7–11 times per year
- ☐ 1 time per month
- ☐ 2–3 times per month
- ☐ 1 time per week
- ☐ 2 times per week
- ☐ 3–4 times per week
- ☐ 5–6 times per week
- ☐ 1 time per day
- ☐ 2 or more times per day

114a. Each time you ate **energy, high-protein**, or **breakfast bars**, how much did you usually eat?

- ☐ Less than 1 bar
- ☐ 1 bar
- ☐ More than 1 bar

115. How often did you eat **yogurt** (NOT including frozen yogurt)?

- ☐ NEVER (GO TO QUESTION 116)
- ☐ 1–6 times per year
- ☐ 7–11 times per year
- ☐ 1 time per month
- ☐ 2–3 times per month
- ☐ 1 time per week
- ☐ 2 times per week
- ☐ 3–4 times per week
- ☐ 5–6 times per week
- ☐ 1 time per day
- ☐ 2 or more times per day

115a. Each time you ate **yogurt**, how much did you usually eat?

- ☐ Less than ½ cup or less than 1 container
- ☐ ½ to 1 cup or 1 container
- ☐ More than 1 cup or more than 1 container

Question 116 appears on the next page

This is a sample form. Do not use for scanning.

Over the past 12 months…

115b. How often was the **yogurt** you ate **low-fat** or **fat-free**?

- ☐ Almost never or never
- ☐ About ¼ of the time
- ☐ About ½ of the time
- ☐ About ¾ of the time
- ☐ Almost always or always

116. How often did you eat **cottage cheese** (including low-fat)?

- ☐ NEVER (GO TO QUESTION 117)

☐ 1–6 times per year	☐ 2 times per week
☐ 7–11 times per year	☐ 3–4 times per week
☐ 1 time per month	☐ 5–6 times per week
☐ 2–3 times per month	☐ 1 time per day
☐ 1 time per week	☐ 2 or more times per day

116a. Each time you ate **cottage cheese**, how much did you usually eat?

- ☐ Less than ¼ cup
- ☐ ¼ to 1 cup
- ☐ More than 1 cup

117. How often did you eat **cheese** (including low-fat; including on cheeseburgers or in sandwiches or subs)?

- ☐ NEVER (GO TO QUESTION 118)

☐ 1–6 times per year	☐ 2 times per week
☐ 7–11 times per year	☐ 3–4 times per week
☐ 1 time per month	☐ 5–6 times per week
☐ 2–3 times per month	☐ 1 time per day
☐ 1 time per week	☐ 2 or more times per day

117a. Each time you ate **cheese**, how much did you usually eat?

- ☐ Less than ½ ounce or less than 1 slice
- ☐ ½ to 1½ ounces or 1 slice
- ☐ More than 1½ ounces or more than 1 slice

117b. How often was the cheese you ate **low-fat or fat-free**?

- ☐ Almost never or never
- ☐ About ¼ of the time
- ☐ About ½ of the time
- ☐ About ¾ of the time
- ☐ Almost always or always

Question 118 appears in the next column

118. How often did you eat **frozen yogurt, sorbet,** or **ices** (including low-fat or fat-free)?

- ☐ NEVER (GO TO QUESTION 119)

☐ 1–6 times per year	☐ 2 times per week
☐ 7–11 times per year	☐ 3–4 times per week
☐ 1 time per month	☐ 5–6 times per week
☐ 2–3 times per month	☐ 1 time per day
☐ 1 time per week	☐ 2 or more times per day

118a. Each time you ate **frozen yogurt, sorbet,** or **ices**, how much did you usually eat?

- ☐ Less than ½ cup or less than 1 scoop
- ☐ ½ to 1 cup or 1 to 2 scoops
- ☐ More than 1 cup or more than 2 scoops

119. How often did you eat **ice cream, ice cream bars,** or **sherbet** (including low-fat or fat-free)?

- ☐ NEVER (GO TO QUESTION 120)

☐ 1–6 times per year	☐ 2 times per week
☐ 7–11 times per year	☐ 3–4 times per week
☐ 1 time per month	☐ 5–6 times per week
☐ 2–3 times per month	☐ 1 time per day
☐ 1 time per week	☐ 2 or more times per day

119a. Each time you ate **ice cream, ice cream bars,** or **sherbet**, how much did you usually eat?

- ☐ Less than ½ cup or less than 1 scoop
- ☐ ½ to 1½ cups or 1 to 2 scoops
- ☐ More than 1½ cups or more than 2 scoops

119b. How often was the ice cream you ate **light, low-fat,** or **fat-free ice cream** or **sherbet**?

- ☐ Almost never or never
- ☐ About ¼ of the time
- ☐ About ½ of the time
- ☐ About ¾ of the time
- ☐ Almost always or always

120. How often did you eat **cake** (including low-fat or fat-free)?

- ☐ NEVER (GO TO QUESTION 121)

☐ 1–6 times per year	☐ 2 times per week
☐ 7–11 times per year	☐ 3–4 times per week
☐ 1 time per month	☐ 5–6 times per week
☐ 2–3 times per month	☐ 1 time per day
☐ 1 time per week	☐ 2 or more times per day

Question 121 appears on the next page

This is a sample form. Do not use for scanning.

Over the past 12 months…

120a. Each time you ate **cake**, how much did you usually eat?

- ☐ Less than 1 medium piece
- ☐ 1 medium piece
- ☐ More than 1 medium piece

121. How often did you eat **cookies** or **brownies** (including low-fat or fat-free)?

- ☐ NEVER (GO TO QUESTION 122)
- ☐ 1–6 times per year
- ☐ 7–11 times per year
- ☐ 1 time per month
- ☐ 2–3 times per month
- ☐ 1 time per week
- ☐ 2 times per week
- ☐ 3–4 times per week
- ☐ 5–6 times per week
- ☐ 1 time per day
- ☐ 2 or more times per day

121a. Each time you ate **cookies** or **brownies**, how much did you usually eat?

- ☐ Less than 2 cookies or 1 small brownie
- ☐ 2 to 4 cookies or 1 medium brownie
- ☐ More than 4 cookies or 1 large brownie

122. How often did you eat **doughnuts**, **sweet rolls**, **Danish**, or **pop-tarts**?

- ☐ NEVER (GO TO QUESTION 123)
- ☐ 1–6 times per year
- ☐ 7–11 times per year
- ☐ 1 time per month
- ☐ 2–3 times per month
- ☐ 1 time per week
- ☐ 2 times per week
- ☐ 3–4 times per week
- ☐ 5–6 times per week
- ☐ 1 time per day
- ☐ 2 or more times per day

122a. Each time you ate **doughnuts**, **sweet rolls**, **Danish**, or **pop-tarts**, how much did you usually eat?

- ☐ Less than 1 piece
- ☐ 1 to 2 pieces
- ☐ More than 2 pieces

123. How often did you eat **sweet muffins** or **dessert breads** (including low-fat or fat-free)?

- ☐ NEVER (GO TO QUESTION 124)
- ☐ 1–6 times per year
- ☐ 7–11 times per year
- ☐ 1 time per month
- ☐ 2–3 times per month
- ☐ 1 time per week
- ☐ 2 times per week
- ☐ 3–4 times per week
- ☐ 5–6 times per week
- ☐ 1 time per day
- ☐ 2 or more times per day

Question 124 appears in the next column

123a. Each time you ate **sweet muffins or dessert breads**, how much did you usually eat?

- ☐ Less than 1 medium piece
- ☐ 1 medium piece
- ☐ More than 1 medium piece

124. How often did you eat **fruit crisp**, **cobbler**, or **strudel**?

- ☐ NEVER (GO TO QUESTION 125)
- ☐ 1–6 times per year
- ☐ 7–11 times per year
- ☐ 1 time per month
- ☐ 2–3 times per month
- ☐ 1 time per week
- ☐ 2 times per week
- ☐ 3–4 times per week
- ☐ 5–6 times per week
- ☐ 1 time per day
- ☐ 2 or more times per day

124a. Each time you ate **fruit crisp**, **cobbler**, or **strudel**, how much did you usually eat?

- ☐ Less than ½ cup
- ☐ ½ to 1 cup
- ☐ More than 1 cup

125. How often did you eat **pie**?

- ☐ NEVER (GO TO QUESTION 126)
- ☐ 1–6 times per year
- ☐ 7–11 times per year
- ☐ 1 time per month
- ☐ 2–3 times per month
- ☐ 1 time per week
- ☐ 2 times per week
- ☐ 3–4 times per week
- ☐ 5–6 times per week
- ☐ 1 time per day
- ☐ 2 or more times per day

125a. Each time you ate **pie**, how much did you usually eat?

- ☐ Less than ⅛ of a pie
- ☐ About ⅛ of a pie
- ☐ More than ⅛ of a pie

The next four questions ask about the kinds of pie you ate. Please read all four questions before answering.

125b. How often were the pies you ate **fruit pie** (such as apple, blueberry, others)?

- ☐ Almost never or never
- ☐ About ¼ of the time
- ☐ About ½ of the time
- ☐ About ¾ of the time
- ☐ Almost always or always

Question 126 appears on the next page

This is a sample form. Do not use for scanning.

Over the past 12 months...

125c. How often were the pies you ate **cream, pudding, custard,** or **meringue pie**?

- ☐ Almost never or never
- ☐ About ¼ of the time
- ☐ About ½ of the time
- ☐ About ¾ of the time
- ☐ Almost always or always

125d. How often were the pies you ate **pumpkin** or **sweet potato pie**?

- ☐ Almost never or never
- ☐ About ¼ of the time
- ☐ About ½ of the time
- ☐ About ¾ of the time
- ☐ Almost always or always

125e. How often were the pies you ate **pecan pie**?

- ☐ Almost never or never
- ☐ About ¼ of the time
- ☐ About ½ of the time
- ☐ About ¾ of the time
- ☐ Almost always or always

126. How often did you eat **chocolate candy**?

☐ NEVER (GO TO QUESTION 127)

- ☐ 1–6 times per year
- ☐ 7–11 times per year
- ☐ 1 time per month
- ☐ 2–3 times per month
- ☐ 1 time per week
- ☐ 2 times per week
- ☐ 3–4 times per week
- ☐ 5–6 times per week
- ☐ 1 time per day
- ☐ 2 or more times per day

126a. Each time you ate **chocolate candy**, how much did you usually eat?

- ☐ Less than 1 average bar or less than 1 ounce
- ☐ 1 average bar or 1 to 2 ounces
- ☐ More than 1 average bar or more than 2 ounces

127. How often did you eat **other candy**?

☐ NEVER (GO TO QUESTION 128)

- ☐ 1–6 times per year
- ☐ 7–11 times per year
- ☐ 1 time per month
- ☐ 2–3 times per month
- ☐ 1 time per week
- ☐ 2 times per week
- ☐ 3–4 times per week
- ☐ 5–6 times per week
- ☐ 1 time per day
- ☐ 2 or more times per day

127a. Each time you ate **other candy**, how much did you usually eat?

- ☐ Fewer than 2 pieces
- ☐ 2 to 9 pieces
- ☐ More than 9 pieces

Question 128 appears in the next column

128. How often did you eat **eggs, egg whites,** or **egg substitutes** (NOT counting eggs in baked goods and desserts)? *(Please include eggs in salads, quiche, and soufflés.)*

☐ NEVER (GO TO QUESTION 129)

- ☐ 1–6 times per year
- ☐ 7–11 times per year
- ☐ 1 time per month
- ☐ 2–3 times per month
- ☐ 1 time per week
- ☐ 2 times per week
- ☐ 3–4 times per week
- ☐ 5–6 times per week
- ☐ 1 time per day
- ☐ 2 or more times per day

128a. Each time you ate **eggs**, how many did you usually eat?

- ☐ 1 egg
- ☐ 2 eggs
- ☐ 3 or more eggs

128b. How often were the eggs you ate **egg substitutes** or **egg whites only**?

- ☐ Almost never or never
- ☐ About ¼ of the time
- ☐ About ½ of the time
- ☐ About ¾ of the time
- ☐ Almost always or always

128c. How often were the eggs you ate **regular whole eggs**?

- ☐ Almost never or never
- ☐ About ¼ of the time
- ☐ About ½ of the time
- ☐ About ¾ of the time
- ☐ Almost always or always

128d. How often were the eggs you ate **cooked in oil, butter,** or **margarine**?

- ☐ Almost never or never
- ☐ About ¼ of the time
- ☐ About ½ of the time
- ☐ About ¾ of the time
- ☐ Almost always or always

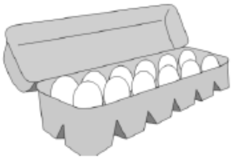

Question 129 appears on the next page

This is a sample form. Do not use for scanning.

Over the past 12 months...

128e. How often were the eggs you ate part of **egg salad**?

- ☐ Almost never or never
- ☐ About ¼ of the time
- ☐ About ½ of the time
- ☐ About ¾ of the time
- ☐ Almost always or always

129. How many cups of **coffee**, caffeinated or decaffeinated, did you drink (including coffee drinks such as Latte, Mocha, Frappuccino, etc.)?

- ☐ NONE (GO TO QUESTION 130)
- ☐ Less than 1 cup per month
- ☐ 1–3 cups per month
- ☐ 1 cup per week
- ☐ 2–4 cups per week
- ☐ 5–6 cups per week
- ☐ 1 cup per day
- ☐ 2–3 cups per day
- ☐ 4–5 cups per day
- ☐ 6 or more cups per day

129a. How often was the coffee you drank **decaffeinated**?

- ☐ Almost never or never
- ☐ About ¼ of the time
- ☐ About ½ of the time
- ☐ About ¾ of the time
- ☐ Almost always or always

130. How many glasses, cans, or bottles of **COLD** or **ICED tea,** caffeinated or decaffeinated, did you drink?

- ☐ NONE (GO TO QUESTION 131)
- ☐ Less than 1 glass, can or bottle per month
- ☐ 1–3 glasses, cans or bottles per month
- ☐ 1 glass, can or bottle per week
- ☐ 2–4 glasses, cans or bottles per week
- ☐ 5–6 glasses, cans or bottles per week
- ☐ 1 glass, can or bottle per day
- ☐ 2–3 glasses, cans or bottles per day
- ☐ 4–5 glasses, cans or bottles per day
- ☐ 6 or more glasses, cans or bottles per day

130a. How often was the cold or iced tea you drank **decaffeinated** or **herbal**?

- ☐ Almost never or never
- ☐ About ¼ of the time
- ☐ About ½ of the time
- ☐ About ¾ of the time
- ☐ Almost always or always

Question 131 appears in the next column

130b. How often was the cold or iced tea you drank **presweetened with either sugar or artificial sweeteners** (such as Splenda, Equal, Sweet'N Low or others)?

- ☐ Almost never or never (GO TO QUESTION 131)
- ☐ About ¼ of the time
- ☐ About ½ of the time
- ☐ About ¾ of the time
- ☐ Almost always or always

130c. What kind of **sweetener** was added to your presweetened cold or iced tea most of the time?

- ☐ Sugar or honey
- ☐ Artificial sweeteners (such as Splenda, Equal, Sweet'N Low or others)

131. How many cups of **HOT tea**, caffeinated or decaffeinated, did you drink?

- ☐ NONE (GO TO QUESTION 132)
- ☐ Less than 1 cup per month
- ☐ 1–3 cups per month
- ☐ 1 cup per week
- ☐ 2–4 cups per week
- ☐ 5–6 cups per week
- ☐ 1 cup per day
- ☐ 2–3 cups per day
- ☐ 4–5 cups per day
- ☐ 6 or more cups per day

131a. How often was the hot tea you drank **decaffeinated** or **herbal**?

- ☐ Almost never or never
- ☐ About ¼ of the time
- ☐ About ½ of the time
- ☐ About ¾ of the time
- ☐ Almost always or always

132. Over the past 12 months, did you add **sugar, honey or other sweeteners** to your tea or coffee (hot or iced)?

- ☐ NO (GO TO QUESTION 133)
- ☐ YES

132a. How often did you add **sugar** or **honey** to your coffee or tea (hot or iced)?

- ☐ Almost never or never (GO TO QUESTION 132c)
- ☐ About ¼ of the time
- ☐ About ½ of the time
- ☐ About ¾ of the time
- ☐ Almost always or always

Question 132c appears on the next page

Question 133 appears on the next page

This is a sample form. Do not use for scanning.

Over the past 12 months...

132b. Each time **sugar** or **honey** was added to your coffee or tea, how much was usually added?

- ☐ Less than 1 teaspoon
- ☐ 1 to 3 teaspoons
- ☐ More than 3 teaspoons

132c. How often did you add **artificial sweetener** (such as Splenda, Equal, Sweet'N Low or others) to your coffee or tea?

- ☐ Almost never or never (GO TO QUESTION 133)
- ☐ About ¼ of the time
- ☐ About ½ of the time
- ☐ About ¾ of the time
- ☐ Almost always or always

132d. What kind of **artificial sweetener** did you usually use?

- ☐ Equal or aspartame
- ☐ Sweet'N Low or saccharin
- ☐ Splenda or sucralose
- ☐ Herbal extracts or other kind

132e. Each time **artificial sweetener** was added to your coffee or tea, how much was usually added?

- ☐ Less than 1 packet or less than 1 teaspoon
- ☐ 1 packet or 1 teaspoon
- ☐ More than 1 packet or more than 1 teaspoon

133. Over the past 12 months, did you add **whiteners** (such as cream, milk, or non-dairy creamer) to your tea or coffee?

- ☐ NO (GO TO QUESTION 134)
- ☐ YES

133a. How often was **non-dairy creamer** added to your coffee or tea?

- ☐ Almost never or never (GO TO QUESTION 133d)
- ☐ About ¼ of the time
- ☐ About ½ of the time
- ☐ About ¾ of the time
- ☐ Almost always or always

Question 133d appears in the next column
Question 134 appears on the next page

133b. Each time **non-dairy creamer** was added to your coffee or tea, how much was usually used?

- ☐ Less than 1 teaspoon
- ☐ 1 to 3 teaspoons
- ☐ More than 3 teaspoons

133c. What kind of **non-dairy creamer** did you usually use?

- ☐ Regular powdered
- ☐ Low-fat or fat-free powdered
- ☐ Regular liquid
- ☐ Low-fat or fat-free liquid

133d. How often was **cream** or **half and half** added to your coffee or tea?

- ☐ Almost never or never (GO TO QUESTION 133f)
- ☐ About ¼ of the time
- ☐ About ½ of the time
- ☐ About ¾ of the time
- ☐ Almost always or always

133e. Each time **cream** or **half and half** was added to your coffee or tea, how much was usually added?

- ☐ Less than 1 tablespoon
- ☐ 1 to 2 tablespoons
- ☐ More than 2 tablespoons

133f. How often was **milk** added to your coffee or tea?

- ☐ Almost never or never (GO TO QUESTION 134)
- ☐ About ¼ of the time
- ☐ About ½ of the time
- ☐ About ¾ of the time
- ☐ Almost always or always

133g. Each time **milk** was added to your coffee or tea, how much was usually added?

- ☐ Less than 1 tablespoon
- ☐ 1 to 3 tablespoons
- ☐ More than 3 tablespoons

133h. What kind of **milk** was usually added to your coffee or tea?

- ☐ Whole milk
- ☐ 2% milk
- ☐ 1% milk
- ☐ Skim, nonfat, or ½% milk
- ☐ Evaporated or condensed (canned) milk
- ☐ Soy milk
- ☐ Rice milk
- ☐ Other

Question 134 appears on the next page

This is a sample form. Do not use for scanning.

Over the past 12 months...

134. How often was **sugar** or **honey** added to foods you ate? *(Please do not include sugar in coffee, tea, other beverages, or baked goods.)*

- ☐ NEVER (GO TO INTRODUCTION TO QUESTION 135)
- ☐ 1–6 times per year
- ☐ 7–11 times per year
- ☐ 1 time per month
- ☐ 2–3 times per month
- ☐ 1 time per week
- ☐ 2 times per week
- ☐ 3–4 times per week
- ☐ 5–6 times per week
- ☐ 1 time per day
- ☐ 2 or more times per day

134a. Each time **sugar** or **honey** was added to foods you ate, how much was usually added?

- ☐ Less than 1 teaspoon
- ☐ 1 to 3 teaspoons
- ☐ More than 3 teaspoons

The following questions are about the kinds of margarine, mayonnaise, sour cream, cream cheese, and salad dressing that you ate. If possible, please check the labels of these foods to help you answer.

135. Over the past 12 months, did you eat **margarine**?

- ☐ NO (GO TO QUESTION 136)
- ☐ YES

135a. How often was the margarine you ate **light**, **low-fat**, or **fat-free** (stick or tub)?

- ☐ Almost never or never
- ☐ About ¼ of the time
- ☐ About ½ of the time
- ☐ About ¾ of the time
- ☐ Almost always or always

136. Over the past 12 months, did you eat **butter**?

- ☐ NO (GO TO QUESTION 137)
- ☐ YES

136a. How often was the butter you ate **light** or **low-fat**?

- ☐ Almost never or never
- ☐ About ¼ of the time
- ☐ About ½ of the time
- ☐ About ¾ of the time
- ☐ Almost always or always

Question 137 appears in the next column

137. Over the past 12 months, did you eat **mayonnaise** or **mayonnaise-type dressing**?

- ☐ NO (GO TO QUESTION 138)
- ☐ YES

137a. How often was the mayonnaise you ate **light, low-fat or fat-free**?

- ☐ Almost never or never
- ☐ About ¼ of the time
- ☐ About ½ of the time
- ☐ About ¾ of the time
- ☐ Almost always or always

138. Over the past 12 months, did you eat **sour cream**?

- ☐ NO (GO TO QUESTION 139)
- ☐ YES

138a. How often was the sour cream you ate **light**, **low-fat**, or **fat-free**?

- ☐ Almost never or never
- ☐ About ¼ of the time
- ☐ About ½ of the time
- ☐ About ¾ of the time
- ☐ Almost always or always

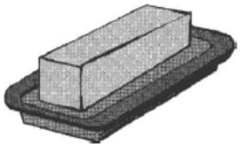

139. Over the past 12 months, did you eat **cream cheese**?

- ☐ NO (GO TO QUESTION 140)
- ☐ YES

139a. How often was the cream cheese you ate **light**, **low-fat**, or **fat-free**?

- ☐ Almost never or never
- ☐ About ¼ of the time
- ☐ About ½ of the time
- ☐ About ¾ of the time
- ☐ Almost always or always

Question 140 appears on the next page

This is a sample form. Do not use for scanning.

Over the past 12 months...

140. Over the past 12 months, did you eat **salad dressing**?

☐ NO (GO TO INTRODUCTION TO QUESTION 141)
☐ YES

140a. How often was the salad dressing you ate **light**, low-**fat** or **fat-free**?

☐ Almost never or never
☐ About ¼ of the time
☐ About ½ of the time
☐ About ¾ of the time
☐ Almost always or always

The following two questions ask you to summarize your usual intake of vegetables and fruits. Please do not include salads, potatoes, or juices.

141. Over the past 12 months, how many servings of **vegetables** (not including salad or potatoes) did you eat per week or per day?

☐ Less than 1 per week
☐ 1–2 per week
☐ 3–4 per week
☐ 5–6 per week
☐ 1 per day
☐ 2 per day
☐ 3 per day
☐ 4 per day
☐ 5 or more per day

142. Over the past 12 months, how many servings of **fruit** (not including juices) did you eat per week or per day?

☐ Less than 1 per week
☐ 1–2 per week
☐ 3–4 per week
☐ 5–6 per week
☐ 1 per day
☐ 2 per day
☐ 3 per day
☐ 4 per day
☐ 5 or more per day

143. Over the past month, which of the following foods did you eat **AT LEAST THREE TIMES?** ***(Mark all that apply.)***

☐ Avocado, guacamole
☐ Cheesecake
☐ Chocolate, fudge, or butterscotch toppings or syrups
☐ Chow mein noodles
☐ Croissants
☐ Dried apricots
☐ Egg rolls
☐ Granola bars
☐ Hot peppers
☐ Jell-O, gelatin
☐ Mangoes
☐ Milkshakes or ice-cream sodas
☐ Olives
☐ Oysters
☐ Pickles or pickled vegetables or fruit
☐ Plantains
☐ Pork neck bones, hock, head, feet
☐ Pudding or custard
☐ Veal, venison, lamb
☐ Whipped cream, regular
☐ Whipped cream, substitute
☐ NONE

144. For **ALL** of the past 12 months, have you followed any type of **vegetarian diet**?

☐ NO (GO TO INTRODUCTION TO QUESTION 145)
☐ YES

144a. Which of the following foods did you **TOTALLY EXCLUDE** from your diet? ***(Mark all that apply.)***

☐ Meat (beef, pork, lamb, etc.)
☐ Poultry (chicken, turkey, duck)
☐ Fish and seafood
☐ Eggs
☐ Dairy products (milk, cheese, etc.)

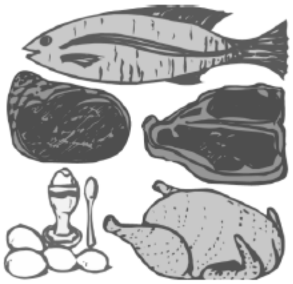

Introduction to Question 145 appears on the next page

This is a sample form. Do not use for scanning.

The next questions are about your use of vitamin pills or other supplements.

145. Over the past 12 months, did you take any **multivitamins**, such as One-a-Day-, Theragran-, Centrum-, or Prenatal-type multivitamins (as pills, liquids, or packets)?

☐ NO (GO TO INTRODUCTION TO QUESTION 147)
☐ YES

146. How often did you take **One-a-day-, Theragran-, Centrum- or Prenatal-type** multivitamins?

☐ Less than 1 day per month
☐ 1–3 days per month
☐ 1–3 days per week
☐ 4–6 days per week
☐ Every day

146a. Did your **multivitamin** usually contain **minerals** (such as iron, zinc, etc.)?

☐ NO
☐ YES
☐ Don't know

146b. For how many years have you taken **multivitamins**?

☐ Less than 1 year
☐ 1–4 years
☐ 5–9 years
☐ 10 or more years

146c. Over the past 12 months, did you take any **vitamins**, **minerals**, or **other herbal supplements** other than your multivitamin?

☐ NO

Thank you *very much* for completing this questionnaire! Because we want to be able to use all the information you have provided, we would greatly appreciate it if you would please take a moment to review each page making sure that you:

- **Did not skip any pages and**
- **Crossed out the incorrect answer and circled the correct answer if you made any changes.**

☐ YES (GO TO INTRODUCTION TO QUESTION 147

Introduction to Question 147 appears in the next column

These last questions are about the vitamins, minerals, or herbal supplements you took that are NOT part of a One-a-day-, Theragran-, or Centrum-type of multivitamin.

Over the past 12 months...

147. How often did you take **Antacids such as Tums or Rolaids**?

☐ NEVER (GO TO QUESTION 148)
☐ Less than 1 day per month
☐ 1–3 days per month
☐ 1–3 days per week
☐ 4–6 days per week
☐ Every day

147a. When you took **Antacids such as Tums or Rolaids**, about how many tablets or lozenges did you take in one day?

☐ Less than 1
☐ 1
☐ 2
☐ 3
☐ 4 or more
☐ Don't know

147b. Was your antacid usually "extra strength"?

☐ NO
☐ YES
☐ Don't know

147c. For how many years have you taken **Antacids such as Tums or Rolaids**?

☐ Less than 1 year
☐ 1–4 years
☐ 5–9 years
☐ 10 or more years

148. How often did you take **Calcium** (with or without Vitamin D) (**NOT** as part of a multivitamin in Question 146 or antacid in Question 147)?

☐ NEVER (GO TO QUESTION 149)
☐ Less than 1 day per month
☐ 1–3 days per month
☐ 1–3 days per week
☐ 4–6 days per week
☐ Every day

Question 149 appears on the next page

Over the <u>past 12 months</u>...

148a. When you took **Calcium**, about how much elemental calcium did you take in one day? *(If possible, please check the label for elemental calcium.)*

- ☐ Less than 500 mg
- ☐ 500–599 mg
- ☐ 600–999 mg
- ☐ 1,000 mg or more
- ☐ Don't know

148b. Did your **Calcium** usually contain **Vitamin D**?

- ☐ NO
- ☐ YES
- ☐ Don't know

148c. Did your **Calcium** usually contain **Magnesium**?

- ☐ NO
- ☐ YES
- ☐ Don't know

148d. Did your **Calcium** usually contain **Zinc**?

- ☐ NO
- ☐ YES
- ☐ Don't know

148e. For how many years have you taken **Calcium**?

- ☐ Less than 1 year
- ☐ 1–4 years
- ☐ 5–9 years
- ☐ 10 or more years

149. How often did you take **Iron** (**NOT** as part of a multivitamin in Question 146)?

- ☐ NEVER (GO TO QUESTION 150)
- ☐ Less than 1 day per month
- ☐ 1–3 days per month
- ☐ 1–3 days per week
- ☐ 4–6 days per week
- ☐ Every day

149a. For how many years have you taken **Iron**?

- ☐ Less than 1 year
- ☐ 1–4 years
- ☐ 5–9 years
- ☐ 10 or more years

Question 150 appears in the next column

150. How often did you take **Vitamin C** (**NOT** as part of a multivitamin in Question 146)?

- ☐ NEVER (GO TO QUESTION 151)
- ☐ Less than 1 day per month
- ☐ 1–3 days per month
- ☐ 1–3 days per week
- ☐ 4–6 days per week
- ☐ Every day

150a. When you took **Vitamin C**, about how much did you take in one day?

- ☐ Less than 500 mg
- ☐ 500–999 mg
- ☐ 1,000–1,499 mg
- ☐ 1,500–1,999 mg
- ☐ 2,000 mg or more
- ☐ Don't know

150b. For how many years have you taken **Vitamin C**?

- ☐ Less than 1 year
- ☐ 1–4 years
- ☐ 5–9 years
- ☐ 10 or more years

151. How often did you take **Vitamin E** (**NOT** as part of a multivitamin in Question 146)?

- ☐ NEVER (GO TO INTRODUCTION TO QUESTION 152)
- ☐ Less than 1 day per month
- ☐ 1–3 days per month
- ☐ 1–3 days per week
- ☐ 4–6 days per week
- ☐ Every day

151a. When you took **Vitamin E**, about how much did you take in one day?

- ☐ Less than 400 IU
- ☐ 400–799 IU
- ☐ 800–999 IU
- ☐ 1,000 IU or more
- ☐ Don't know

151b. For how many years have you taken **Vitamin E**?

- ☐ Less than 1 year
- ☐ 1–4 years
- ☐ 5–9 years
- ☐ 10 or more years

Introduction to Question 152 appears on the next page

Over the past 12 months…

The last two questions ask you about other supplements you took more than once per week.

152. Please mark any of the following **single supplements** you took more than once per week (**NOT** as part of a multivitamin in Question 147):

- ☐ B-6
- ☐ B-complex
- ☐ B-12
- ☐ Beta-carotene
- ☐ Folic acid/folate
- ☐ Magnesium
- ☐ Occu-vite/Eye health
- ☐ Potassium
- ☐ Selenium
- ☐ Vitamin A
- ☐ Vitamin D
- ☐ Zinc

153. Please mark any of the following **herbal, botanical, or other supplements** you took more than once per week.

- ☐ Chondroitin
- ☐ Coenzyme Q-10
- ☐ Echinacea
- ☐ Energy supplements
- ☐ Fish oil/omega 3's
- ☐ Flaxseed/oil
- ☐ Garlic
- ☐ Ginger
- ☐ Ginkgo biloba
- ☐ Ginseng
- ☐ Glucosamine/ chondroitin
- ☐ Peppermint
- ☐ Probiotics
- ☐ Saw palmetto
- ☐ Soy supplement
- ☐ Sports supplements
- ☐ St. John's wort
- ☐ Other

Thank you *very much* for completing this questionnaire! Because we want to be able to use all the information you have provided, we would greatly appreciate it if you would please take a moment to review each page making sure that you:

- **Did not skip any pages and**
- **Crossed out the incorrect answer and circled the correct answer if you made any changes.**

28 Appendix L: Automated Self-Administered 24-hour Recall (ASA24)

Overview of the ASA24 Respondent Web sites and Considerations Related to Data Security and Participant Confidentiality

Extensive evidence has demonstrated that 24-hour dietary recalls provide the highest quality, least biased dietary data. Traditional 24-hour recalls, however, are expensive and impractical for large-scale research because they rely on trained interviewers and multiple administrations to estimate usual intakes. As a result, researchers often make use of food frequency questionnaires, which are less expensive but contain substantial error.

To address this challenge, investigators at NCI created the Automated Self-administered 24-hour Recall (ASA24) system, a freely-available, web-based tool that enables multiple automated self-administered 24-hour recalls. ASA24 was developed under contract with Westat, a social science research firm located in Rockville, MD, and builds on t he Food Intake Recording Software System (FIRSSt) developed by Dr. Tom Baranowski of the Baylor College of Medicine. An Extern al Working Group provided advice about the needs and interests of potential users.

ASA24 consists of a Respondent Web site used to collect recall data and a Researcher Web site used to manage study logistics and obtain data analyses. Two Respondent Web sites are currently available in English and Spanish: ASA24-2014 and ASA24-Kids-2014. This document provides an overview of the methodology and main features of the ASA24 Respondent sites, as well as information on security of the data collected and protections to the confidentiality of the participants of studies that make use of ASA24. Images of the main Respondent site screens are also included.

ASA24 Respondent Web site Methodology

Respondents are guided through the 24-hour recall inter view using a modified version of the USDA's Automated Multiple-Pass Method (AMPM). The steps in the interview process include:

1. Meal-based Quick List
2. Meal Gap Review
3. Detail Pass
4. Forgotten Foods
5. Fina l Review
6. Last Chance
7. Usual Intake Question
8. Supplement Module (if selected by the researcher)

Meal-based Quick List
During the first pass of the interview, respondents are asked to provide a list of the foods and drinks consumed at each meal occasion for one of two possible 24-hour time periods, depending on the preference of the researcher: yesterday, from midnight to midnight; or the past 24-hours based on the time of participant login. Respondents are able to browse food group categories to find their foods and drinks or search for a particular item. Foods and drinks

reported at each meal are recorded in the My Foods and Drinks panel within the instrument. This initial step is called the meal-based Quick List. In addition to selecting an eating occasion (e.g., breakfast, lunch, snack), respondents are also prompted to specify the time of the occasion before reporting the foods and drinks consumed. The researcher can opt to collect additional contextual information including where meals were eaten, television and computer use during meals, and whether the meal was eaten alone or with others.

Meal Gap Review

Once respondents finish creating their My Foods and Drinks list at the end of the Quick List step, they are asked if they consumed anything during any 3-hour gaps between eating occasions, and, for the midnight to midnight version, between midnight and the first eating occasion, and between the last eating occasion and midnight. A response of "Yes" to any gap will return the respondent to the Quick List to add the appropriate food(s) or drink(s).

Detail Pass

Respondents are asked for details about the foods and drinks they recorded during the Quick List, including form (e.g., raw), preparation methods (e.g., grilled or roasted), the amount eaten, and any additions (e.g., sugar, coffee cream, salad dressing). Researchers can also opt to ask the source (grocery store, farmer's market, vending machine, etc.) of each food reported.

Forgotten Foods

Following the Detail Pass, a pop-up window appears with questions probing about the consumption of commonly forgotten foods and drinks (e.g., snack foods, fruits, vegetables, cheese, water, coffee, tea). Respondents must check either "Yes" or "No" for each food or drink probed. For any "Yes" response, the respondent will be returned to the Quick List to add the forgotten item(s).

Final Review

Respondents are prompted to review all of the foods and drinks reported for the intake day, and make edits and add meals, foods and drinks as necessary.

Last Chance

After the Final Review, a pop-up screen appears with an option to add food or drink items if respondents have still forgotten any. Again, respondents will be brought back to the Quick List to add more items; otherwise, they will move forward to the final question in the food and drink module.

Usual Intake Question

The final question asks: Was the amount of food that you ate yesterday more than usual, usual, or less than usual? This question probes whether this was a typical day's intake.

Supplement Module (if selected by the researcher during study set-up)

Respondents are asked to provide information about the type and dose of supplements consumed by comp leting a Quick List, a Detail Pass, and a Final Review . Supplements include vitamins, minerals, and other supplements including prescription supplements . Respondents are able to browse supplement categories (e.g., Multi-Vitamin/ Mineral, Calcium products, Herbal/Botanicals) or search for a particular supplement. The supplements included are based on those reported in the National Health and Nutrition Examination Survey.

ASA24 Respondent Web Site Features

The ASA24 Respondent Web site guides the part icipant t hrou gh the completion of a 24-hour recall for the previous day, from midnight to midnight, using a dynamic user interface. The instrument:

- provides an animated guide and audio and visual cues to instruct participants and enhance use in low-literacy populations and with children (with options to turn off the guide and/or the audio);
- asks respondents to report eating occasion and time of consumption;
- includes optional modules to query where meals were eaten, whether meals were eaten alone or with others, and television and computer use during meals;
- flows as per modified USDA Automated Multiple-Pass Method (AM PM) 24-hour recall; allows respondents to report foods and drinks by browsing by category or searching from a list of food and drink terms derived from the National Health and Nutrition Examination Survey (NHANES);
- asks detailed questions about food preparation, portion size, and additions so that food codes from USDA's Food and Nutrient Database for Dietary Studies (FNDDS) can be assigned; it also provides an option to query respondents about food source;
- uses images to assist respondents in reporting portion size;
- allows the respondent to add or modify food and drink choices at multiple points during the interview;
- includes an optional module to query dietary supplement intake based on supplements reported in NHANES;
- is available in English and Spanish; and
- is compliant with Section 508 of the 1973 Rehabilitation Act.

The Respondent Web sites do not provide any direct feedback to respondents. Instead, researchers can obtain analysis files from the Researcher Website and contact respondents with any findings they choose to share.

Data Security and Protections to Confidentiality of Participants using ASA24

Researchers using ASA24 do not provide the National Cancer Institute, Westat (the research firm that developed and maintains ASA24), or the ASA24 system with any identifying data for participants of their studies. Rather, researchers specify an identifier for each respondent and download system-generated usernames and encrypted passwords that they provide to respondents so that they may access the application.

AS24 also does not collect any identifying data directly from respondents. However, IP address information is accessed for the purpose of routing information between the server and the respondent's computer – often the IP address is that of the user's Internet Service Provider (ISP). IP addresses are not stored or tracked by ASA24. However, logs of connections are kept in the hosting environment for audit trail purposes. This information is not mined in any way but would be available if there were legal obligations to release it.

Response data are secured at the hosting site using industry standard security controls, including firewalls and encruyption. All data entered into ASA24 at the respondent's computer is encrypted by the internet browser (e.g., Internet Explorer, Firefox) before they are transmitted to our servers using Secure Socket Layer (SSL) Technology. SSL allows for the authentication of the sending and receiving computers.

Only a particular study's investigator(s) and the ASA24 operations team can access response data. Access is gained through usernames and strong passwords.

ASA24 Respondent Web site Screen Shots

Figure 1: The participant can choose to complete ASA24 in either English or Spanish.

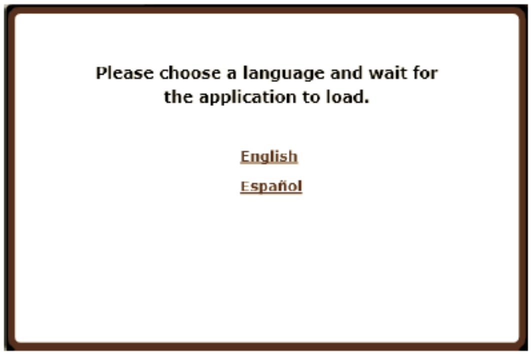

Figure 2: An introductory screen prompts the participant to report everything that she or he had to eat or drink yesterday.

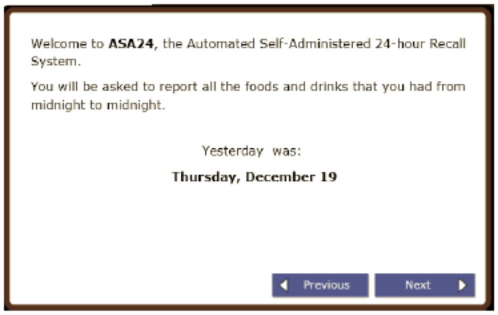

4

Figure 3: The main ASA24 screen is used to select foods and drinks to be added to My Foods and Drinks during the Quick Pass, and to add details and make changes. The animated guide provides an overview of the main ASA24 screen and gives instructions on how to report all foods, drinks, and supplements (if the optional Supplement Module is selected by the researcher) consumed either from midnight to midnight yesterday or for the past 24 hours, starting at the time of login (depending on the researcher preferences).

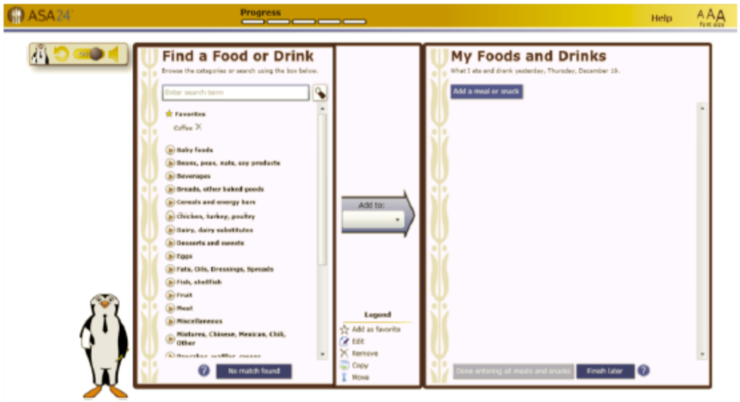

Figure 4: The meal details screen collects information on the eating occasion and time, and displays optional modules that can be selected by the researcher to query where meals were eaten, whether meals were eaten alone or with others, and television and computer use during meals. This is the first step in the meal-based Quick List.

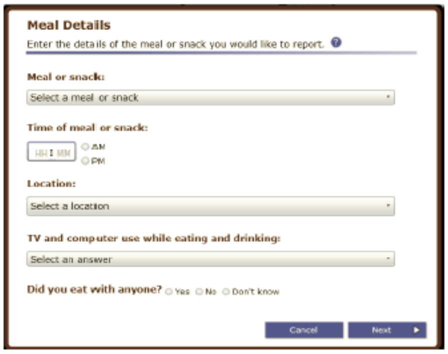

Figure 5: Participants complete the Quick List pass by browsing categories or searching for the foods and drinks they consumed. The food and drink terms are based on the National Health and Nutrition Examination Survey.

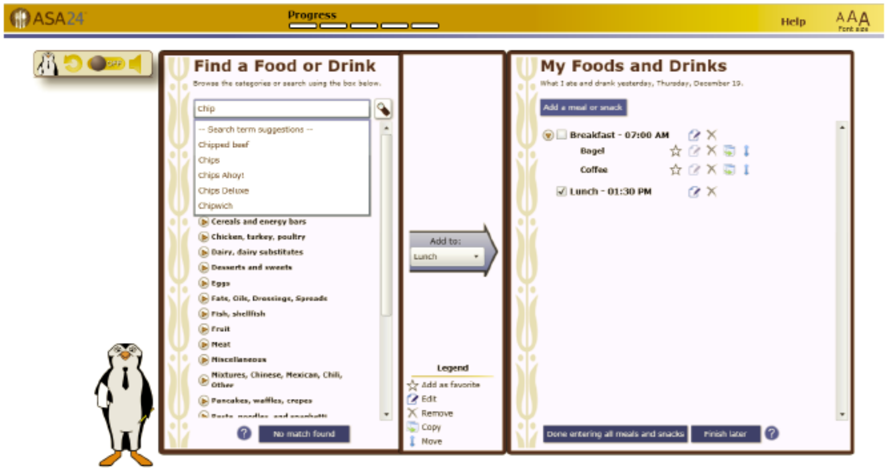

Figure 6: The foods and drinks that the participant reports during the Quick List are displayed in the My Foods and Drinks panel on the right of the main screen.

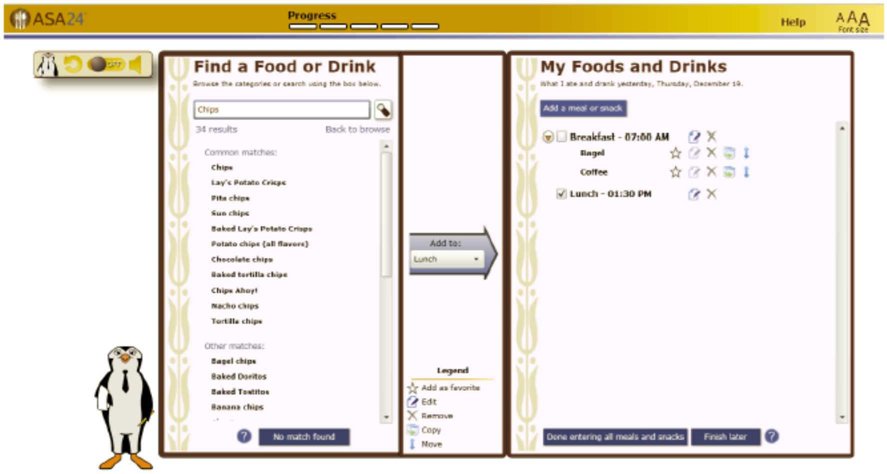

Figure 7: After the participant reports the foods and drinks consumed yesterday and selects Done entering all meals and snacks, the Meal Gap Review is displayed.

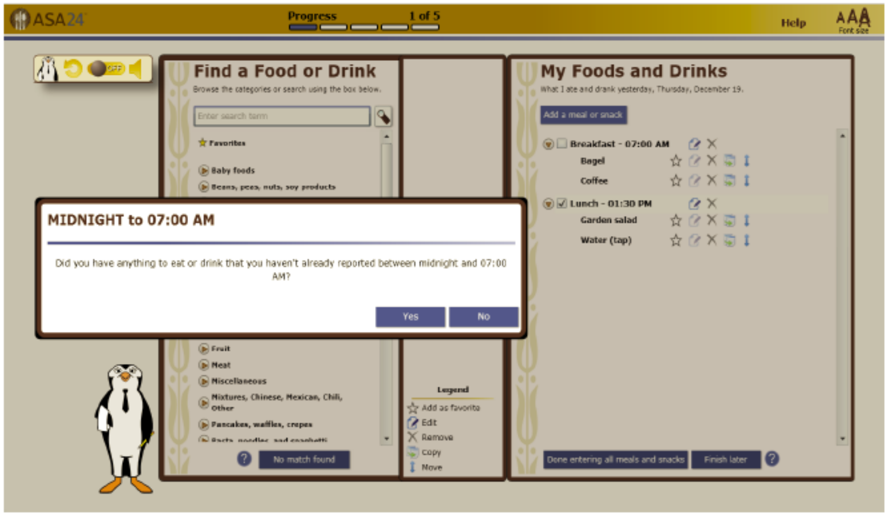

Figures 8a-c: The detail pass prompts the participant to report the details of each food and drink, including the specific type or how it was prepared, anything added to it, and the amount that was consumed.

Figure 8a.

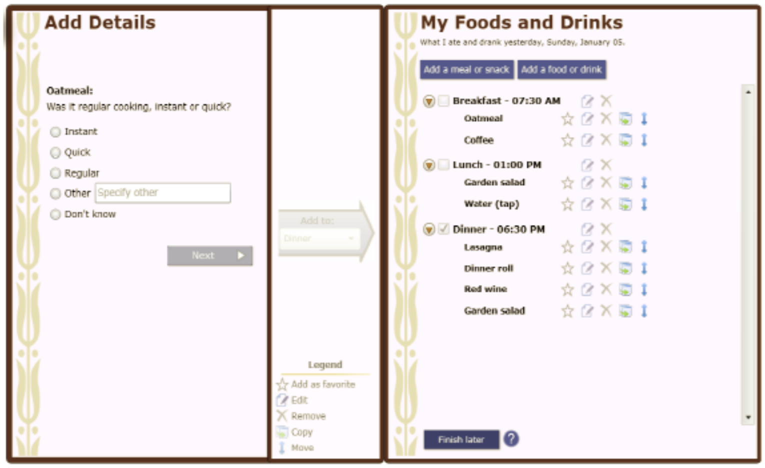

7

Figure 8b.

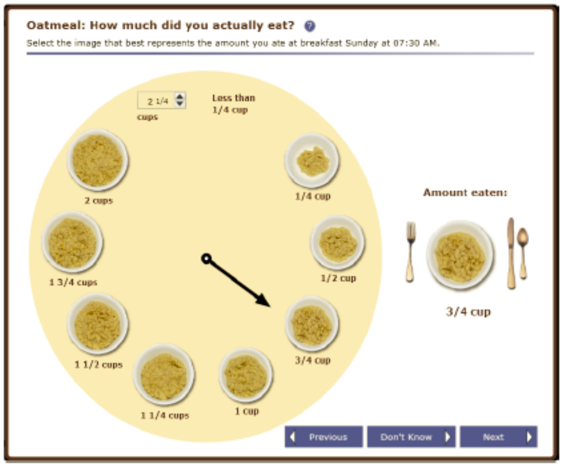

Figure 8c.

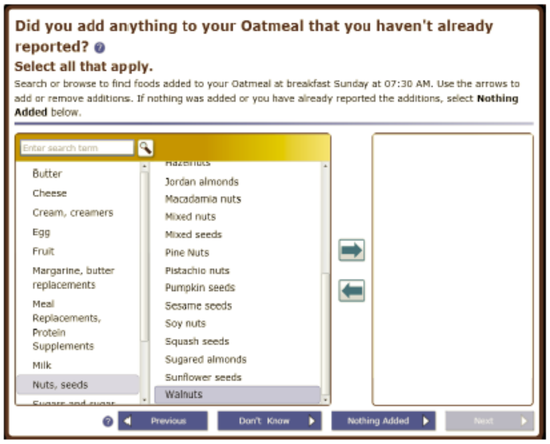

8

Figure 9: Once the Detail Pass is complete, the Final Review begins. The participant can make changes to the details for a given food or drink and add more foods and drinks if necessary.

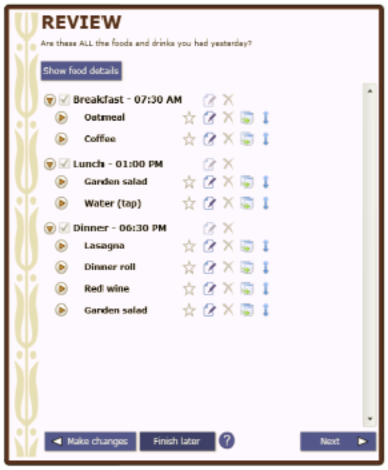

Figure 10: The forgotten foods pass queries the participant about frequently forgotten foods. Responding yes to one or more categories will result in a prompt to add the forgotten foods or drinks.

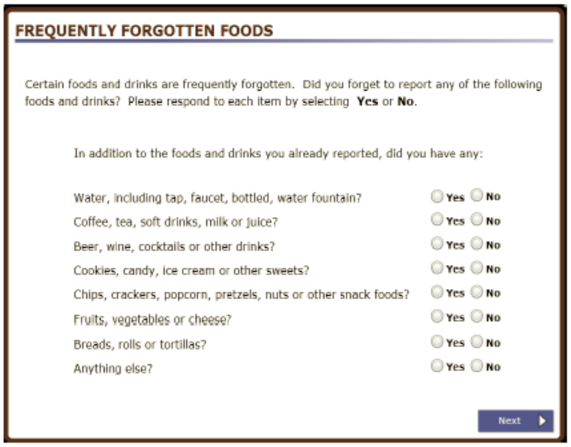

Figure 11: The final question in the food and drink module asks the participant whether the amount of foods and drinks consumed yesterday was usual.

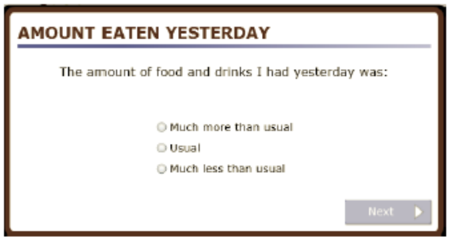

Figure 12: If the Supplement Module is selected by the researcher during study set up, the participant will also be asked about the supplements, if any, that he or she took either from midnight to midnight yesterday or for the past 24 hours, starting at the time of login (depending on researcher preferences).

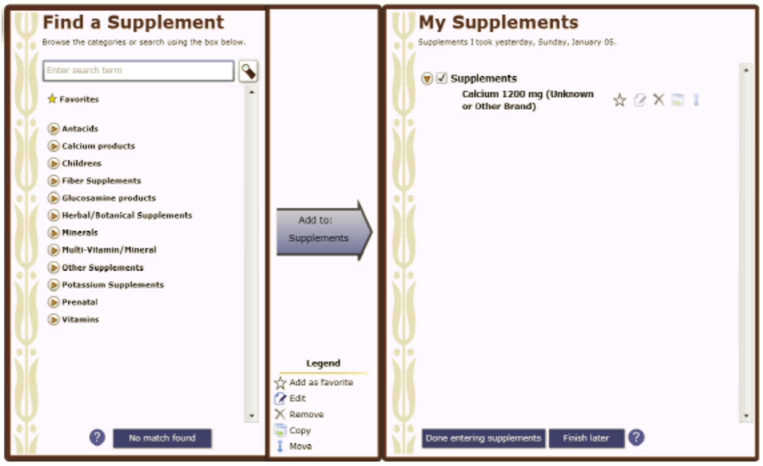

10

Figure 13: The Supplement Module collects details about each supplement reported.

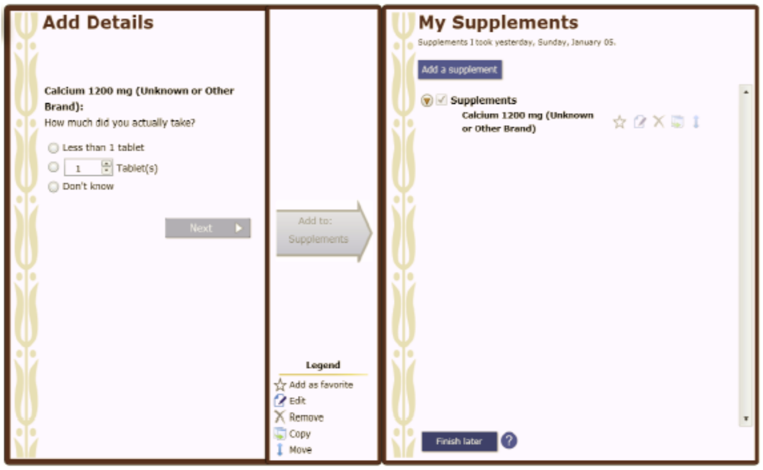

Figure 14: Once the participant completes the food and drink module and the supplement module, if selected by the researcher during study set up, his or her responses are saved in the secure database and he or she can exit ASA24. Only the researcher(s) who are registered to that study and the ASA24 operations team can access the response data.

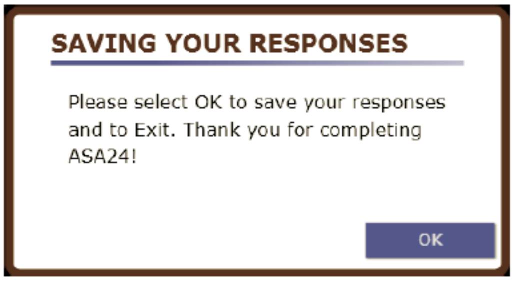

29 APPENDIX M: qADAM Questionnaire

Questions Used as Part of the qADAM Questionnaire

1. How would you rate your libido (sex drive)?
   1(terrible) 2(poor) 3(average) 4(good) 5(excellent)

2. How would you rate your energy level?
   1(terrible) 2(poor) 3(average) 4(good) 5(excellent)

3. How would you rate your strength/endurance?
   1(terrible) 2(poor) 3(average) 4(good) 5(excellent)

4. How would you rate your enjoyment of life?
   1(terrible) 2(poor) 3(average) 4(good) 5(excellent)

5. How would you rate your happiness level?
   1(terrible) 2(poor) 3(average) 4(good) 5(excellent)

6. How strong are your erections?
   (1= extremely weak 5= extremely strong)
   1 2 3 4 5

7. How would you rate your work performance over the past 4 weeks?
   1(terrible) 2(poor) 3(average) 4(good) 5(excellent)

8. How often do you fall asleep after dinner?
   1(never) 2(1-2/week) 3(3-4/week) 4(5-6/week) 5(every night)

9. How would you rate your sports ability over the past 4 weeks?
   1(terrible) 2(poor) 3(average) 4(good) 5(excellent)

10. How much height have you lost?
    1(2" or more) 2(1.5-1.9") 3(1-1.4") 4(0.5-0.9") 5(none-0.4")

The quantitative ADAM questionnaire. Int J Impot Res. 2010 Jan; 22(1): 20–24.

O Mohamed,[1] R E Freundlich,[1] H K Dakik,[1] E D Grober,[2] B Najari,[3] L I Lipshultz,[1] and M Khera[1,*]
Published online 2009 Aug 6. doi: 10.1038/ijir.2009.35
PMCID: PMC2834355

30 Appendix N: Psychosexual Daily Questionnaire

1. Please rate your overall level of sexual desire today by circling the appropriate number below:

0 — 1 — 2 — 3 — 4 — 5 — 6 — 7

none (0) · very low (1) · very high (7)

2. Please rate highest level of enjoyment or pleasure of any sexual activity that you experienced today.

(a) without a partner (e.g., masturbationl sexual fantasies) and/or (b) with a partner (e.g., kissing, intercourse) by circling the appropriate number below.

(a) Without a partner — none 0 — 1 — 2 — 3 — 4 — 5 — 6 — 7 very high enjoyment/pleasure

(b) With a partner — none 0 — 1 — 2 — 3 — 4 — 5 — 6 — 7 very high enjoyment/pleasure

(c) Please indicate if partner is available ☐ yes ☐ no

3. Please rate your mood by writing the number that corresponds to the scale below. For each item 0 indicates that the descriptor is not at all true; 7 indicates that the descriptor is very true for you today.

not at all true 0 — 1 — 2 — 3 — 4 — 5 — 6 — 7 very true

(a) Angry? ___	(d) Full of pep/energetic?___	(g) Friendly?______
(b) Alert? ___	(e) Sad or Blue?__________	(h) Nervous?______
(c) Irritable?___	(f) Tired? _______________	(i) Well/good?______

4. For all of the items below check yes if you have experienced (or are experiencing) today, otherwise check no.

	Yes	No			Yes	No			Yes	No	
(a)	☐	☐	sexual daydreams	(e)	☐	☐	orgasm	(i)	☐	☐	masturbation
(b)	☐	☐	anticipation of sex	(f)	☐	☐	flirting (by others toward you)	(j)	☐	☐	night spontaneous erection
(c)	☐	☐	sexual interactions with partner	(g)	☐	☐	ejaculation	(k)	☐	☐	day spontaneous erection
(d)	☐	☐	flirting (by you)	(h)	☐	☐	intercourse	(l)	☐	☐	erection in response to sexual activity

Answer the following two questions only if you experienced an erection as shown in No. 4. j-l above.

5. If you experienced an erection today, indicate the % full erection that you experienced by circling the appropriate number below (make a reasonable estimate):

%= 0 10 20 30 40 50 60 70 80 90 100

6. If you experienced an erection today, indicate whether it was maintained for a satisfactory duration by circling the appropriate number below:

not satisfactory 0 — 1 — 2 — 3 — 4 — 5 — 6 — 7 very satisfactory

A Simple Self-Report Diary for Assessing Psychosexual Function in Hypogonadal Men
Ka Kui Lee[1,2], Nancy Berman[3], Gerianne M. Alexander[4], Laura Hull[1], Ronald S. Swerdloff[1] and Christina Wang[1,*]
Article first published online: 2 JAN 2013
DOI: 10.1002/j.1939-4640.2003.tb02728.x

31 Appendix O: Sun Exposure and Behaviour Inventory (SEBI v2)

Subject ID:__________ **Survey Administration**: 1st / 2nd **Date**: ________

For **each question** listed, please check off the **ONE** answer that is the **BEST** response to the question.

A **sunburn** is defined as skin redness or pain which lasts at least one day after sun exposure.

1. Which of the following **BEST** describes your skin's reaction to **one hour of summer sun without sunscreen**?

☐ Always burns, never gets darker /tans (Very light skin, Caucasian)
☐ Always burns at first, but sometimes gets darker/tans with continued sun exposure (Light skin, Caucasian)
☐ Sometimes burns first, but always gets darker/tans with continued sun exposure (Medium skin, usually Caucasian)
☐ Rarely burns, gets darker/tans easily with sun exposure (Olive skin, may be of many race/ethnic backgrounds)
☐ Never burns, skin gets darker with sun exposure (Brown to dark-brown skin, may be of many race/ethnic backgrounds)
☐ Never burns, no darkening of skin color with sun exposure (Black skin, African ancestry)

2. How many times in your life have you had a **SUNBURN?**

☐ None
☐ 1-10
☐ 11-20
☐ More than 20

3. How many times in your life have you had a **SUNBURN that BLISTERED?**

☐ None
☐ 1-3
☐ 4-10
☐ More than 10

4. How many times in your life have you used a **tanning bed/booth/sunlamp?**

☐ None
☐ 1-10
☐ 11-50
☐ 51-100
☐ 101-500
☐ More than 500

5. Have you lived in an area with a **significantly sunnier climate** than Boston for six months or more?

☐ Yes
☐ No

IF yes, what city/state or country? ____________________________ For how long?

☐ Less than 5 years
☐ 6-10 years
☐ 11-20 years
☐ More than 20 years

6. Please rate your **lifetime** sun exposure

	Very low	Low	Moderate	High	Very high
**TOTAL** sun exposure					
Exposure from **OUTDOOR RECREATION**					
Exposure from your **JOB**					

7. Which of the following **BEST** describes your **NATURAL** skin and hair color when you were a young adult (18-25)?
☐ Light skin, red or red-blonde hair
☐ Light skin, blonde or light brown hair
☐ Light skin, brown or black hair
☐ Medium-tone skin, brown or black hair
☐ Brown skin, dark brown or black hair
☐ Black skin, dark brown or black hair

8. What is your eye color?
☐ Brown
☐ Blue, green, gray
☐ Hazel (brownish green)

Now, think about your current lifestyle to answer the remaining questions.

9. Think about what you do when you are outside <u>during the summer on a warm sunny day</u>.

	Never	Rarely	Sometimes	Often	Always
How often do you wear **SUNSCREEN**?					
How often do you wear a **SHIRT WITH SLEEVES that cover your shoulders**?					
How often do you wear a **HAT that shades your face, ears, and neck**?					
How often do you stay in the **SHADE** or **UNDER AN UMBRELLA**?					
How often do you wear **SUNGLASSES**?					

10. What is the **Sun Protection Factor (SPF)** of the sunscreen that you use most often?
☐ Less than SPF 15
☐ SPF 15 or higher
☐ Don’t know
☐ I don’t use sunscreen

11. Does your sunscreen have both **UVA** and **UVB** protection?

☐ Yes
☐ No
☐ Don't know
☐ I don't use sunscreen

12. How often do you spend time in the sun or in a tanning bed/booth in order to get a tan or to feel good?

☐ Never
☐ Rarely
☐ Sometimes
☐ Often
☐ Always

13. Have you been tan in the last 12 months?

☐ Yes
☐ No

14. In the **SUMMER** (June, July, August), on average, how many hours per day are you **outside** between 10am and 4pm?

	Less than 1hour	1hour	2hours	3hours	4hours	5hours	6 hours
On **WEEKDAYS** (Monday-Friday)							
On **WEEKENDS** (Saturday and Sunday)							

15. In **NON-SUMMER** months (September to May), on average, how many hours per day are you **outside** between 10am and 4pm?

	Less than 1hour	1hour	2hours	3hours	4hours	5hours	6 hours
On **WEEKDAYS** (Monday-Friday)							
On **WEEKENDS** (Saturday and Sunday)							

32 Appendix P: DSMB Charter

Improving Reproductive Fitness through Pretreatment with Lifestyle Modification in Obese Women with Unexplained Infertility: (FIT-PLESE FEMALE)

DATA AND SAFETY MONITORING BOARD (DSMB) CHARTER

1. Purpose and Responsibilities of the DSMB

The members of the Data and Safety Monitoring Board (DSMB) identified in this Charter for the FIT-PLESE Female study are responsible for safeguarding the interests of study participants, assessing the safety and efficacy of all study procedures, and shall monitor the overall conduct of the FIT-PLESE Female trial. This Committee will serve as an independent advisory group to the Director of NICHD, and is required to provide recommendations about starting, continuing, and stopping the FIT-PLESE Female study.

This Committee is responsible for identifying mechanisms to complete various tasks that will impact the safety and efficacy of all study procedures, and overall conduct. The table below identifies the key areas where oversight is necessary and the ways in which the Committee for the FIT-PLESE Female study will complete those tasks.

Basic Responsibility of DSMB	Method DSMB for FIT-PLESE Female will use to complete task
Familiarize themselves with the study protocol	· Review study protocols and informed consent forms.
Monitor adverse events	· Adverse Event: Review quarterly progress reports prepared by the DCC on behalf of RMN.<br>· Serious Adverse Events: Review report submitted by the DCC on behalf of RMN within one week of the event if life threatening or fatal, or within two weeks otherwise. The DSMB will submit a report of their review to the NICHD Committee Coordinator within 7 business days if the SAE is life threatening or fatal, or within two weeks otherwise.
Monitor data quality	· Conduct interim evaluations of the data.
Oversee participant recruitment and enrollment	· Review interim progress reports prepared by the DCC on behalf of RMN.
Develop an understanding of the Study's risks and benefits	· Review study protocols and related literatures.<br>· Review interim reports of subject accrual and outcome measures provided by the DCC.

	· Assess the need to perform further in-depth evaluation of the benefits and risks of the study after reviewing each report.
Ensure the proper reporting occurs	· Review and approve the meeting and reporting schedule listed in Section 5 of this DSMB charter.

2. Contacts

<u>NICHD</u>
Louis DePaolo, PhD, Program Officer/Committee Coordinator
Esther Eisenberg, MD, MPH, Project Scientist

<u>Data Coordination Center (DCC)</u>
Heping Zhang, PhD, DCC Principal Investigator
Hao Huang, MD, DCC Data Manager

The Data Manager at the DCC will prepare and review the DSMB reports prior to submission to the DSMB, and will not be blind to treatment condition.

<u>Lead Investigator</u>
Richard S. Legro, MD

3. DSMB Members, Organizational Chart, & Communications

<u>Members</u>

The DSMB for the FIT-PLESE Female study is comprised of the members listed in the table below. In addition, their high level roles and responsibilities are identified in the table.

Name of Member	Role on DSMB	High Level Responsibilities
Frank Witter, MD	Chair of DSMB<br>Voting member	· Chair the DSMB discussion and prepare written recommendations to NICHD.<br>· Ensure the safety of study subjects, the integrity of the research data.<br>· Provide NICHD with advice on the ethical and safe progression of studies conducted in the RMN.<br>· Advises on research design issues, data quality and analysis, and research participant protection for each prospective and on-going study.
Rev. Phillip Cato, PhD	Voting member	· Ensure the safety of study subjects, the integrity of the research data.<br>· Provide NICHD with advice on the ethical and safe progression of studies conducted in the RMN.<br>· Advises on research design issues, data quality and analysis, and research participant protection for each prospective and on-going study.
PonJola Coney, MD	Voting member	
Lurdes Y.T. Inoue, PhD	Voting member	
Stacey A. Missmer, ScD	Voting member	
Robert E. Brannigan, MD	Voting member	

Only Voting members for this DSMB may attend closed sessions for this Committee. In addition, only Voting members will have access to unblinded data points for this Committee.

Organizational Chart

The following diagram illustrates the relationship between the DSMB and other entities in the FIT-PLESE Female study.

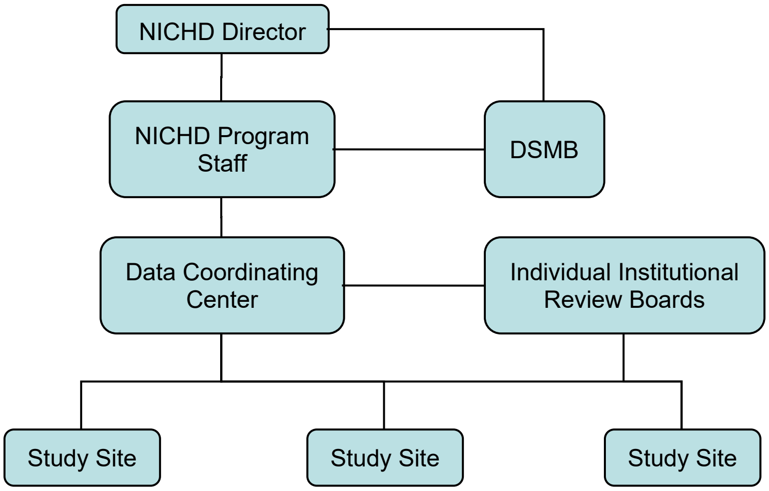

Communication

Communication for members of this DSMB will be primarily through the NICHD Program Office and, where applicable, the Data Coordination Center (DCC). Investigators from the FIT-PLESE Female study will not communicate directly with DSMB members about the study, except when making presentations or responding to questions at DSMB meetings or during scheduled conference calls.

4. Conflict of Interest and Compensation

It is extremely important that all members of the DSMB state any real or apparent conflicts of interests at the onset of the study. Members of the DSMB shall read the NICHD Clinical Research Guidance Document regarding Conflict of Interest and provide their signed summary of any COI for the study, at its onset, to the NICHD Committee Coordinator. A table summarizing any COI within the DSMB is provided in the Appendix.

Prior to each meeting, all members of the RMN DSMB will have an opportunity to state whether they have developed any new conflicts of interest since the meeting. As a new COI is identified it must be documented in the table in the Appendix and a new signed summary of the COI should be provided to the NICHD Committee Coordinator. The Coordinator will forward the COI documentation to the DCC for record-keeping purposes. If a new conflict is reported, the Coordinator and staff will determine if the conflict limits the ability of the DSMB member to participate in the discussion.

All DSMB members will be compensated for their role in supporting the committee. Compensation will include an honorarium for meeting attendance and any travel costs.

5. Meeting Schedule

DSMB meetings will be conducted quarterly. However, the DSMB may hold a meeting at any time in accordance with their mission. The NICHD Committee Coordinator will notify the DCC of any change in schedule.

6. Blinding

All summaries for DSMB reports will be presented in a blinded fashion, unless specified by the DSMB Chair.

7. Report Schedule and Content

The type of reports (full or brief) is indicated below, followed by a description of the contents of each type.

DSMB Report	Report Submission Date	Type of Report
1.	tbd	Brief
2.	tbd	Brief
3.	tbd	Brief
4.	tbd	**Full**
5.	tbd	Brief
6.	tbd	**Full**
7.	tbd	Brief
8.	tbd	**Full**
9.	tbd	Brief
10.	tbd	**Full**
11.	tbd	Brief
12.	tbd	**Full**
13.	tbd	Brief
14.	tbd	**Full**
15.	tbd	Brief
16.	tbd	**Full**

Brief DSMB reports will include the following summaries:
- overall actual versus projected enrollment accrual
- overall randomization update
- overall study drop-out rate

- serious adverse events
- primary outcome measures update

Full DSMB reports will include the following summaries:

- recruitment update (number screened) overall and by site
- enrollment update (enrolled defined as randomized to a treatment) overall and by site
- accrual status including actual enrollment compared to projections overall and by site
- randomization update (i.e., number assigned to each treatment arm)
- study drop-out rate for enrolled patients (number, reason, time point) overall and by site)
- pre-specified subset of baseline demographic data for enrolled patients
- safety data, adverse events, and serious adverse events
- number of case report forms expected
- number/percentage of expected case report forms received – overall and by site
- number of case report forms that are query clean
- primary outcome measures update

References

NIH Policy for Data and Safety Monitoring
http://grants.nih.gov/grants/guide/notice-files/NOT98-084.html
Guidance on Reporting Adverse Events to Institutional Review Boards for NIH-supported Multi-center Clinical Trials
http://grants.nih.gov/grants/guide/notice-files/not99-107.html.

Appendix: Summary of COI within the DSMB

DSMB Member Name	Date Submitted Signed COI	Was a COI Identified?	Will the Member Remain part of the Committee?
Rev. Phillip Cato, PhD			
PonJola Coney, MD			
Lurdes Y.T. Inoue, PhD			
Stacey A. Missmer, ScD			
Robert E. Brannigan, MD			
Frank Witter, MD			

Conflict of Interest Statement

Improving Reproductive Fitness through Pretreatment with Lifestyle Modification in Obese Women with Unexplained Infertility: (FIT-PLESE FEMALE)

I, ______________________, assuming the role of _____________________________ (insert role, for example: DSMB member)

for the ____
(insert project or study name)

agree to the following statements.

□ I agree to:

- protect the interests and safety of study participants;
- uphold the integrity of the research process including data collection and analysis to be as free from bias and preconception as I am able;
- adhere to the highest scientific and ethical standards, to comply with all relevant regulations and to eliminate or disclose, during my involvement with the proposed clinical research project, any real or apparent conflicts of interest.

In addition:

□ I declare that I, my spouse or dependent children, or organization with which I am connected, do/does not have any financial interest in the ___________________ study, where financial interested is defined by the DHHS, as anything of monetary value, including but not limited to, salary or other payments for services (for example, consulting fees or honoraria); equity interests (for example, stocks, stock options or other ownership interests); and intellectual property rights (for example, patents, copyrights and royalties from such rights).
The financial interest term does not include various items which can be found in The Federal regulation, PHS, DHHS Part 50--Policies of General Applicability; Subpart F- *Responsibility of Applicants for Promoting Objectivity in Research for Which PHS Funding Is Sought.*

For Federal employees, financial interests that are allowable and require disclosure are:

Financial Interest Disclosure: Financial interest that require disclosure are stock holdings in pharmaceutical firms, medical device manufacturers, and biotechnology companies

Allowable Financial Interests: In a company that produces a product that is being evaluated by a study, participants may hold up to $15,000 of stock: and up to an aggregate of $25,000 of the stock of that

company and its competitors who produce that (or a similar) product. As an alternative to individual stock holdings, participants may hold up to an aggregate of $50,000 in sector mutual funds-including pharmaceutical/health care sectors.

For holdings in excess of these *de minimus* levels, a conflict of interest analysis needs to be conducted by NIH regarding the holding, the company producing the product being evaluated under the study, and its competitors, and, if a conflict exists, could lead to the need to withdraw from the study.

□ I agree to not withhold any data related to the ______________________ study or to interfere with the analysis or publication of the study's results.

□ I will not engage in activities that could be viewed as real or apparent COI, including but not limited to:

> □ having a part-time, full-time, paid, or unpaid employee status of any organizations that are: (a) involved in the study under review; (b) whose products will be used or tested in the study under review, or whose products or services would be directly and predictably affected in a major way by the outcome of the study;
>
> □ being an officer, member, owner, trustee, director, expert advisor, or consultant of such organizations;
>
> □ being a current collaborator or associate of the principal investigator (applicable to potential members of data safety and monitoring boards);
>
> □ having a scientific interest beyond that required for my role, where scientific interest is defined as having influence over the protocol, the study design, conducting the study analysis or any reporting related to the investigation (applicable to potential members of data safety and monitoring boards).

Reproductive Medicine Network (RMN)
Data and Safety Monitoring Board (DSMB)
Confidentiality Agreement

As a DSMB member I understand that I will be provided with and have access to documents submitted by the NICHD or the Data Coordination Center as they relate to RMN protocols, Registries or other Network-related materials, including proprietary and confidential information.

I shall not disclose any confidential RMN information (oral or written) unless required to do so by law. Confidential documents may be distributed to an administrative assistant, who is not permitted to share Network materials with anyone other than me.

I agree that I will not distribute or publish RMN records. I further agree that I shall not make use of Network materials except for the express purpose of advising the RMN Steering Committee and the NICHD.

I have read this agreement and agree to abide by its terms.

Name (Print or Type): ____

Signature: ______________________________________ **Date:** ______________

33 Appendix Q: Investigator Signature of Agreement

Investigator Signature of Agreement

The Eunice Kennedy Shriver National Institute of Child Health and Human Development
Reproductive Medicine Network

Title: Improving Reproductive Fitness through Pretreatment with Lifestyle Modification in Obese Women with Unexplained Infertility: (FIT-PLESE FEMALE)

Version: 4.0

Principal Investigator:

I, *[Insert PI's name]* , the Principal Investigator for *[Insert Institute Name]*, hereby certify that I have read and agree to conduct this study in accordance with this protocol on behalf of all RMN Investigators and research staff from my site.

I will conduct the clinical study as described and will adhere to the *Code of Federal Regulations,* Title 21 and Title 25, Part 46, Good Clinical Practices (GCP), International Conference on Harmonisation (ICH), and the Declaration of Helsinki. I have read and understood the contents of the Protocol.

The signature of the investigator below indicates acceptance of the protocol and a complete understanding of the investigator commitments as outlined in Form FDA 1572, Statement of Investigator.

____

Principal Investigator's Signature Date

____

Printed Name Date

Reproductive Medicine Network

Funded by *Eunice Kennedy Shriver* NICHD

Improving Reproductive Fitness through Pretreatment with Lifestyle Modification in Obese Women with Unexplained Infertility:

(FIT-PLESE FEMALE)

Protocol Leader: Richard S. Legro, MD
Data and Coordination Leader: Heping Zhang, PhD

Version 3.0

Last Updated: October 7, 2016

Summary of Changes from Version 2.0

1. **Section 2.3 Tertiary Aims**
    - *Changed:* "Are there changes in stress as determined by salivary cortisol according to treatments?"

      To: "Are there changes in stress as determined by salivary biomarker of stress according to treatments?" CHANGED TO INCLUDE MULTIPLE BIO-MARKERS OF STRESS

2. **Section 7.2 Timing of Evaluations**
    - Under Screening Visit(s) -> Complete Questionnaires:
        - *Added:* "Sun Exposure and Behaviour Inventory (SEBI)" ADDED TO CHECK SUNSCREEN USE AND SUN EXPOSURE IN MALES DUE TO POSSIBLE INFLUENCE ON SPERM QUALITY
    - ***End of Empirical Infertility treatment study (Phase 2) Drug Visit:***
        - *Changed:* This visit will mirror the end of Phase I visit with the exception that an additional ultrasound will not be performed specifically for this study visit and we will repeat biometric measurements and QOL surveys in the male. We will repeat most of the parameters obtained at the baseline visit:

          To: This visit will mirror the end of Phase I visit with the exception that we will repeat biometric measurements and QOL surveys in the male. We will repeat most

of the parameters obtained at the baseline visit: CHANGED TO MAKE LESS CONFUSING

- ***End of Empirical Infertility treatment study (Phase 2) Drug Visit:***
  - *Changed:* Males will return for biometric measures and repeat QOL surveys (excluding DHQII)

    To: Males use Aria Scale to record weight and repeat QOL surveys (excluding DHQII) CHANGED TO ELIMINATE THE NEED FOR THE MALES TO COME BACK TO THE CENTER FOR THE END OF PHASE 2 VISIT, THE ARIA SCALE WILL COLLECT THEIR WEIGHT FROM HOME

3. **Section 7.3.4 Biospecimens**
   - *Changed*: The goal is to measure cortisol at the respective peak and nadir of the diurnal cycle.

     To: The goal is to measure biomarkers for stress at the respective peak and nadir of the diurnal cycle. CHANGED TO INCLUDE MULTIPLE BIO-MARKERS OF STRESS

4. **Section 7.3.5 Questionnaires**
   - *Added*: Male sunscreen exposure will be assessed by the Sun Exposure and Behaviour Inventory (SEBI v2). ADDED TO CHECK SUNSCREEN USE AND SUN EXPOSURE IN MALES DUE TO POSSIBLE INFLUENCE ON SPERM QUALITY

5. **Section 10.3.2 Secondary Outcome Measurements**
   - *Changed:* “cortisol” to “biomarkers of stress” CHANGED TO INCLUDE MULTIPLE BIO-MARKERS OF STRESS

6. **Female Informed Consent**
   Under “What am I being asked to do?”

   Table of Visits: corrected HgbA1C to be drawn with female hormones CORRECTION TO TABLE

   Section: Randomization/Beginning of Phase 1 Lifestyle Modification
   - *Changed:* “cortisol testing” to “testing for biomarkers of stress”
   - *Deleted*: “Cortisol is a stress hormone.” CHANGED TO INCLUDE MULTIPLE BIO-MARKERS OF STRESS

   Section: Lifestyle Modification Close-out/End of Phase1
   - *Changed: “cortisol” to “for biomarkers of stress”* CHANGED TO INCLUDE MULTIPLE BIO-MARKERS OF STRESS

Section: Baseline Visit Infertility Treatment Phase 2

- *Deleted:* "(normal organisms found in the vagina)" and "This is done be swabbing the vaginal wall using a cotton swab and then placed in a special media." ELIMINATED CONFUSING DUPLICATED INFORMATION

Under "Who can see or use my information? How will by personal information be protected?"

- *Changed:* "Only investigators from the HMC/PSU and RMN/Yale University have access to these electronic records for study purposes only." to "Only investigators from the HMC/PSU and RMN/Yale University investigators as well as investigators approved by the RMN to use the data will have access to these de-identified electronic records." CLARIFIED THAT OTHER INVESTIGATORS WITH APPROVAL MAY USE RESEARCH DATA COLLECTED.

- *Added:* "Your de-identified information may be used in the future for other research. Your de-identified data will be shared with the National Institute of Child Health and Human Development on the Data and Specimen Hub which is a resource for investigators to share de-identified research data from studies." ADDED TO MORE EXPLICITLY OBTAIN CONSENT FOR NIH DATA SHARING

Under "Optional Study for Cortisol Saliva Collection"

- *Changed:* In heading, changed "Optional Study for Cortisol Saliva Collection" to "Optional Study Saliva for Biomarkers of Stress Collection" CHANGED TO INCLUDE MULTIPLE BIO-MARKERS OF STRESS

- *Changed:* "As an optional part of the study, we would like to obtain a sample of your saliva for cortisol testing. Cortisol is a stress hormone." to "As an optional part of the study, we would like to obtain a sample of your saliva to test for biomarkers of stress such as amylase and cortisol. Cortisol and amylase are stress hormones." CHANGED TO INCLUDE MULTIPLE BIO-MARKERS OF STRESS

7. Male Informed Consent

Under "How long will I be in the Study?"

- *Changed:* "about 3-4 months" to "8 months" CLARIFIED TOTAL LENGTH OF TRIAL INSTEAD OF JUST MALE PARTICIPATION IN PHASE 2
- *Corrected:* number of times male subjects asked to return to research site to "3" times. CHANGED TO REFLECT MALE NOT REQUIRED TO ATTEND END OF STUDY VISIT

Under "What am I being asked to do?"

Section: Medical Assessment & Questionnaires

- *Added*: "During the screening visit,"

- *Added*: "The Sun Exposure and Behaviour Inventory (SEBI) will assess your sunscreen usage." ADDED ADDITIONAL QOL

- *Added*: "When your partner is randomized, you will use your partner's Aria Scale to have your weight recorded. You will be required to weigh yourself once at the time your partner is randomized. This Wi-Fi smart scale will send your data to the secure database for review. If you are unable to use the Aria scale wirelessly, you will be required to contact the study coordinator via email, text or phone call to report your result.

  When your partner has her End of Study visit, you will be required to use the Aria Scale again to have your weight recorded. You will also repeat the quality of life questionnaires as you did during the screening visit, excluding the DHQII. If your partner is pregnant, the FertiQOL questionnaire will be skipped."

  ADDED DETAILS OF USING ARIA SCALE

Section: Saliva Collection for Cortisol Testing

- *Changed*: In heading, from "Saliva Collection for Cortisol Testing" to "Saliva Collection for Testing for Biomarkers of Stress" CHANGED TO INCLUDE MULTIPLE BIO-MARKERS OF STRESS

- *Changed:* "If you have consented to the collection of saliva for cortisol testing, this will be collected at this time. Cortisol is a stress hormone." to "If you have consented to the collection of saliva for testing for biomarkers of stress, such as cortisol or amylase, this will be collected at this time. Cortisol and amylase are stress hormones." CHANGED TO INCLUDE MULTIPLE BIO-MARKERS OF STRESS

Under "Who can see or use my information? How will by personal information be protected?"

- *Added:* "The research records are entered into an electronic password protected database. Only HMC/PSU and RMN/Yale University investigators as well as investigators approved by the RMN to use the data will have access to these de-identified electronic records. This data is managed by the RMN/Yale University Data Coordinating Center and will be kept indefinitely." ADDED TO EXPLICITLY STATE AND OBTAIN CONSENT FOR WHO WILL HAVE ACCESS TO THE RESEARCH DATABASE

Under Optional part(s) of the study:

Under Optional Blood and Urine for RMN Biologic Repository:

  - *Added:* "These samples can be used for other research in the future after the study is over and also be shared with other investigators who collaborate with the RMN."

    CORRECTED TO INCLUDE FUTURE USE AND SHARING OF SAMPLES WHICH WAS ALREADY IN THE OTHER OPTIONAL COLLECTIONS OF BIOLOGICAL SAMPLES

- *Added:* "Your de-identified information may be used in the future for other research. Your de-identified data will be shared with the National Institute of Child Health and Human Development on the Data and Specimen Hub which is a resource for investigators to share de-identified research data from studies." ADDED TO MORE EXPLICITLY OBTAIN CONSENT FOR NIH DATA SHARING

"Optional Study for Cortisol Saliva Collection"

- *Changed:* In heading, removed "Optional Study for Cortisol Saliva Collection" to "Optional Study Saliva for Biomarkers of Stress Collection" CHANGED TO INCLUDE MULTIPLE BIO-MARKERS OF STRESS
- *Changed:* "As an optional part of the study, we would like to obtain a sample of your saliva for cortisol testing. Cortisol is a stress hormone." to "As an optional part of the study, we would like to obtain a sample of your saliva to test for biomarkers of stress such as amylase and cortisol. Cortisol and amylase are stress hormones." CHANGED TO INCLUDE MULTIPLE BIO-MARKERS OF STRESS

8. Appendices

- *Added:* Appendix O: Sun Exposure and Behaviour Inventory (SEBI v2)
- *Re-lettered:* Former Appendix O to become Appendix P, and Former Appendix P to become Appendix Q

Improving Reproductive Fitness through Pretreatment with Lifestyle Modification in Obese Women with Unexplained Infertility:

(FIT-PLESE FEMALE)

Protocol Leader: Richard S. Legro, MD
Data and Coordination Leader: Heping Zhang, PhD

Version 4.0

Last Updated: July 3, 2017

Summary of Changes from Version 3.0

1. Throughout protocol: formatting errors were corrected, version number changed from 3.0 to 4.0; updates made to: email addresses, Table of Contents and date of last protocol revisions (July 3, 2017).

2. **Table 1: Study Visits and Procedures**
   - *__Added__:* Blood work for "genetic tests and hgb electrophoresis"

3. **Section 7.2 Timing of Evaluations**
   - 6. Laboratory tests

     __Added__:

     "Females:
     -Offer optional recommended genetic tests and hemoglobin electrophoresis per ACOG guidelines. The costs for these blood tests are not included in determining the patient care budget for this protocol."

   - *__Baseline Ultrasound Visit for Empirical Infertility Treatment (Phase 2)__*

     6. Dispense Infertility Treatment Study Medication (see Table 9).

     __Added__:

"Please note, if the female has a prior history of over-response to clomiphene citrate at the 100mg dose, the starting dose may be adjusted downward."

- **Protocol medication adjustment:*

  __Changed:__ *"*1. If no ovulation or development of at least one follicle ≥ 18 mm in average diameter, then increase dosage in the subsequent cycle."

  *__To__: "*1. If no ovulation or development of at least one follicle ≥ 18 mm in average diameter on treatment day 18, then bring subject in for a progesterone level and serum hCG. If the progesterone is less than 2.5 ng/ml and the hCG is negative, medication will be increased in a stair-step fashion to the maximum dose of 150mg. Progestin will not be used."

  __Added to #3:__ **"**Dosage may also be reduced in a subsequent cycle if excessive follicular recruitment leads to cycle cancellation before follicles reach maturity."

  __Added:__ "4. If the subject has an adverse reaction to the medication, the medication may be withheld for the remaining cycles."

4. Female Informed Consent

- Under "**What is the purpose of this research study?**"

  __Deleted__: "Approximately 380 women will take part in this research study nationwide as part of the Reproductive Medicine Network (RMN).

- Under **"What am I being asked to do?"**

  __Added__: to Blood work: "genetic tests and hgb electrophoresis"

- Under **"*__Screening Visit__*"**

  __Added__: "(body mass index)" after "BMI"

  *__Deleted:__ "*The Female Sexual Function Index (FSFI) and Female Sexual Distress Scale (FSDS) will assess your sexual function. The PHQ-9 will assess your feelings for depression. The Short Form 12 (SF 12) will assess your daily activities. FertiQoL will assess how your infertility affects your thoughts and feelings. The Stop Bang Questionnaire and Epworth Sleepiness Scale (ESS) will assess your sleep habits. The ASA24 and the Diet History Questionnaire II (DHQ II) will be completed to assess your past and current dietary intake."

  __Added__: "We will also give you questionnaires concerning your sexual function, feelings of depression, daily activities, sleep habits, and past and current dietary history.

  *__Added to__ "*You will be free to skip any questions that you would prefer not to answer"

  -> *"*or that make you uncomfortable."

  __Added__ "checking" *__to__* "hormone levels"

  __Added to__ "anemic" -> "(a condition where your blood lacks healthy red blood cells)"

Deleted:

"- TSH, Prolactin, AMH and/or FSH, E2, and HgbA1C

- Safety labs such as a blood count, lipid panel and liver and kidney tests."

Added:

"- Checking hormone levels

- Checking safety labs"

Changed: "if you have consented to this optional section of the study" *to*: "if you consent to this optional section of the study"

Added to "we must report it to the State Health Department" -> "and someone from the department may contact you"

Added:

"- You will also be offered optional recommended genetic tests and hemoglobin electrophoresis (test performed to test different types of hemoglobin, which transfers oxygen in the blood) per standard guidelines. If any of these tests are abnormal, you will be referred for appropriate treatment and counseling, however, they will not prevent you from being eligible for the study. These tests are NOT included or required for the study and will be at your cost."

*Added to "*5 million total motile sperm" - > "(percent of sperm swimming forward)"

- Under "*-Lifestyle Arm A: Increased Physical Activity"*

  *Added to "*folic acid" -> "(a B vitamin)"

- Under *"-Lifestyle Arm B: Increased Physical Activity with weight loss"*

  *Added to "*restricting" -> "/decreasing" ->

  Added "taking" *to* "weight loss medication"

  *Added to "*folic acid" -> "(a B vitamin)"

  Deleted "and beta-carotene"

- Under ***"Lifestyle Modification Close-out/End of Phase 1-***

  Deleted "including FSFI, FSDS, PHQ-9, SF-12, FertiQOL, Stop Bang, ESS, and ASA24."

- Under ***Baseline Visit Infertility Treatment Phase 2-***

  *Added to "*first day of your next menstrual period" -> "(menstrual cycle day 1)"

- Under ***Monitor Visits (Cycle day 8 to 12 and then beyond as required)-***

*Added: "*the study doctor will decide this. If your lead follicles are too small after 18 days of treatment, your doctor may ask you to come in for some blood tests to see if you can start your next dose of study medication right away."

- Under ***Urine Pregnancy Test-***

  Deleted: "Before being released form the study,"

  Deleted: "You will be contacted at 12 weeks of pregnancy to see how your pregnancy is progressing."

- *Added:* "***Other visits which may occur***

  Unexpected visits, blood tests and procedures may occur during the study. These could include, for example, if your menses are late during Phase 2, an additional visit to the clinic or additional blood tests."

- Under **What are the possible risks or discomforts?**

  Added: "any/" *to* "all of these side effects"

  Deleted: "You will be assigned to a study treatment group by chance, like the flip of a coin."

  Added:

  "Risks of Genetic Testing:
  A Federal law, called the Genetic Information Nondiscrimination Act (GINA), generally makes it illegal for health insurance companies, group health plans, and most employers to discriminate against you based on your genetic information.
  This law generally will protect you in the following ways:
  • Health insurance companies and group health plans may not request your genetic information that we get from this research.
  • Health insurance companies and group health plans may not use your genetic information when making decisions regarding your eligibility or premiums.
  • Employers with 15 or more employees may not use your genetic information that we get from this research when making a decision to hire, promote, or fire you or when setting the terms of your employment.
  Be aware that this Federal law does not protect you or your family against genetic discrimination by companies that sell life insurance, disability insurance, or long-term care insurance. Also, GINA does not prohibit discrimination of individuals with a genetic disorder that has been diagnosed. However, in order to do everything possible to keep this from happening, the results of this test will NOT be given to anyone outside the study staff. This means that it will not be made available to you, your family members, your private physician, your employer, your insurance company or any other party as allowed by law."

- Under **What other choices do I have if I do not participate?**

Added: “Choosing not to participate will not affect your standard of care treatment.”

- Under **Will I be paid for being in this study?**

  Added: “You will keep the FitBit and Aria scale.”

- Under **Will I have to pay for anything?**

  The following is a list of the non-covered services related to this study:

  Added:
  - “Any genetic tests and hemoglobin electrophoresis”
  - “Any additional test which the doctor may order outside of the research procedures while you are in the study

- Under **When is the Study over? Can I leave the Study before it ends?**

  Added: “Your regular medical care at the facility will not be impacted in any way.”

- Under **Who can see or use my information? How will my personal information be protected?**

  Added: “(the Data Coordinating Center)”

- Under **What information about me may be collected, used or shared with others**

  Added: “Any information from study visits, interviews or forms completed by you as part of the study will only be shared with the study team, and not to other entities without your prior authorization and approval.”

- Under **Who may use and share information about me**?

  Added: “Office of Regulatory Affairs”

- Under **What if I decide not to give permission to use and give out my health information?**

  Added: “Please refer to above information regarding other options available to you other than participating in this study.”

- Under **Who can I call with questions, complaints or if I’m concerned about my rights as a research subject?**

  Added “The Office of Regulatory Affairs at the University of Pennsylvania is the central human subjects protection oversight body for this study.”

- Under **Optional part(s) of the study**

  Added “in which you may participate” *to* “the research”

  Added “some or all of” *to* “these optional parts”

- Under *Optional Storage of Blood & Urine Reserve for Future Research*

Deleted: "Your samples will be stored for a minimum of 5 years, and perhaps further into the future."

- Under *Optional Blood and Urine for RMN Biologic Repository*

  Deleted: "and stored for a minimum of 5 years, and perhaps further into the future."

- *Changed order of (to after the Signature of Person Giving Informed Consent):*

  "Signature of Person Obtaining Informed Consent

  Your signature below means that you have explained the optional part(s) to the research to the subject or subject representative and have answered any questions he/she has about the research.

  ___________________________ ____

  Name of Person Obtaining **Signature** **Date**

  Consent (Please Print)"

5. **Male Informed Consent**

- Under ***"What am I being asked to do"***
  - ***Medical Assessment & Questionnaires***

  Deleted: "You will be given 11 additional questionnaires to complete. A Genetic Risk questionnaire is completed to determine if you have an increased risk of having a baby with a genetic disorder. A Patient Health Questionnaire (PHQ-9) and the Short Form 12 (SF-12) will assess your daily activities. The Fertility Quality of Life Questionnaire (FertiQoL) will assess how your infertility affects your thoughts and feelings. The Epworth Sleepiness Scale (ESS) and Stop-Bang Questionnaire will assess your quality of sleep. The International Index of Erectile Function Survey (IIEF), Psychosexual Daily Questionnaire (PDQ), and qADAM questionnaire will assess your sexual function. The Sun Exposure and Behaviour Inventory (SEBI) will assess your sunscreen usage. The Automated Self-Administered 24-hour Recall (ASA24) and Dietary History (DHQ-II) will be completed to assess your past and current dietary intake."

  Added: "We will also give you questionnaires concerning your sexual function, feelings of depression, daily activities, sleep habits, sunscreen usage, and past and current dietary history. "

  Added to "You will be free to skip any questions that you would prefer not to answer"

  -> "or that make you uncomfortable."

  - ***Repository Semen, Blood and Urine Specimen Collection***

Deleted: "These samples will only be collected if you have consented to the optional part of the study."

Added: "Genetic testing may be performed on some of these samples. Additional information about these optional samples is included at the end of this consent form. These samples will only be collected if you have consented to the optional part of the study."

- Under **What are the possible risks or discomforts?**

  Added:

  "Risks of Genetic Testing:
  A Federal law, called the Genetic Information Nondiscrimination Act (GINA), generally makes it illegal for health insurance companies, group health plans, and most employers to discriminate against you based on your genetic information.
  This law generally will protect you in the following ways:
  • Health insurance companies and group health plans may not request your genetic information that we get from this research.
  • Health insurance companies and group health plans may not use your genetic information when making decisions regarding your eligibility or premiums.
  • Employers with 15 or more employees may not use your genetic information that we get from this research when making a decision to hire, promote, or fire you or when setting the terms of your employment.
  Be aware that this Federal law does not protect you or your family against genetic discrimination by companies that sell life insurance, disability insurance, or long-term care insurance. Also, GINA does not prohibit discrimination of individuals with a genetic disorder that has been diagnosed. However, in order to do everything possible to keep this from happening, the results of this test will NOT be given to anyone outside the study staff. This means that it will not be made available to you, your family members, your private physician, your employer, your insurance company or any other party as allowed by law."

- Under **What other choices do I have if I do not participate?**

  Added: "Choosing not to participate will not affect your standard of care treatment."

- Under **When is the Study over? Can I leave the Study before it ends?**

  Added: "Your regular medical care at the facility will not be impacted in any way."

- Under **What information about me may be collected, used or shared with others**

  Added: "Any information from study visits, interviews or forms completed by you as part of the study will only be shared with the study team, and not to other entities without your prior authorization and approval."

- Under **Who may use and share information about me**?

  Added: "Office of Regulatory Affairs"

- Under **What if I decide not to give permission to use and give out my health information?**

  Added: "Please refer to above information regarding other options available to you other than participating in this study."

- Under **Who can I call with questions, complaints or if I'm concerned about my rights as a research subject?**

  Added "The Office of Regulatory Affairs at the University of Pennsylvania is the central human subjects protection oversight body for this study."

- Under **Optional part(s) of the study**
- Under *Optional Blood and Urine for RMN Biologic Repository*

  Deleted: "and stored for a minimum of 5 years, and perhaps further into the future."

- Under *Optional Storage of Semen for Future Use in Research*

  Deleted: "Your sample will be stored for a minimum of 5 years, and perhaps further into the future."

- *Changed order of (to after the Signature of Person Giving Informed Consent):*

  "Signature of Person Obtaining Informed Consent

  Your signature below means that you have explained the optional part(s) to the research to the subject or subject representative and have answered any questions he/she has about the research.

  ___________________________ _______________________________________

  Name of Person Obtaining **Signature** **Date**

  Consent (Please Print)"
