# Supplementary material for: Effects of preconception lifestyle intervention in infertile women with obesity: The FIT-PLESE randomized controlled trial
Source: PLoS Med. 2022 Jan 18;19(1):e1003883. doi: 10.1371/journal.pmed.1003883 (PMC8765626; doi:10.1371/journal.pmed.1003883)
Supplement: S1 Table — (DOCX) [file pmed.1003883.s002.docx]

**S1 Table. Drop out or study exclusion reasons by study timepoint.**

| **Drop out reasons** | **Intervention group** | **After randomization and End of phase I** | **Between end of phase I and phase II**  **cycle 1** | **Between phase II cycle 1 and cycle 2** | **Between phase II cycle 2 and cycle**  **3** | **After phase II cycle 3** | **Total** |
| --- | --- | --- | --- | --- | --- | --- | --- |
| No longer interested in participating | Standard lifestyle | 2 | 5 |  | 1 |  | **8** |
|  | Intensive lifestyle | 4 | 4 | 1 |  | 1 | **10** |
| Unable to continue study due to personal | Standard lifestyle | 2 | 6 | 1 | 2 |  | **11** |
| constraints/access to clinic is difficult/move Intensive lifestyle 2 4 **6**  out of area | | | | | | | |
| Change in relationship status | Standard lifestyle | 1 | 5 |  |  |  | **6** |
|  | Intensive lifestyle | 1 | 1 |  |  |  | **2** |
| Unable to contact patient | Standard lifestyle | 0 | 6 |  |  |  | **6** |
|  | Intensive lifestyle | 1 | 4 | 1 |  |  | **6** |
| Patient non-compliant with protocol | Standard lifestyle | 1 | 1 |  |  | 1 | **3** |
|  | Intensive lifestyle | 1 | 1 |  |  |  | **2** |
| Other | Standard lifestyle |  | 4 |  | 1 | 1 | **6** |
|  | Intensive lifestyle |  | 4 |  | 1 |  | **5** |
| **Total** |  | **15** | **45** | **3** | **5** | **3** | **71** |
