# Supplementary material for: Effects of preconception lifestyle intervention in infertile women with obesity: The FIT-PLESE randomized controlled trial
Source: PLoS Med. 2022 Jan 18;19(1):e1003883. doi: 10.1371/journal.pmed.1003883 (PMC8765626; doi:10.1371/journal.pmed.1003883)
Supplement: S2 Table — (DOCX) [file pmed.1003883.s003.docx]

**S2 Table. Change in hormones from baseline after 16 week preconception intervention**

|  | **Standard Lifestyle** | **Intensive Lifestyle** | **P value** |
| --- | --- | --- | --- |
| Free testosterone (ng/dL) | 160 | 149 |  |
|  | -0.04±0.14 | 0.01±0.11 |  |
|  | -0.03(-0.10 to 0.04) | 0.02(-0.07 to 0.08) | 0.002 |
| AMH (ng/mL) | 160 | 149 |  |
|  | -0.9±2.4 | -0.8±2.1 |  |
|  | -0.4(-2.1 to 0.3) | -0.4(-2.0 to 0.4) | 0.687 |
| FSH (mIU/mL) | 160 | 149 |  |
|  | 0.2±3.7 | 0.1±3.4 |  |
|  | 0.3(-1.9 to 2.2) | -0.2(-1.6 to 1.8) | 0.409 |
| LH (mIU/mL) | 159 | 149 |  |
|  | -0.3±19.3 | -0.6±15.6 |  |
|  | -0.2(-3.7 to 2.3) | -1.0(-3.3 to 1.3) | 0.279 |
| Fasting glucose (mg/dL) | 160 | 149 |  |
|  | -5.0±17.2 | -6.4±15.6 |  |
|  | -2.9(-12.1 to 4.6) | -2.8(-13.4 to 3.3) | 0.532 |
| Total cholesterol (mg/dL) | 152 | 136 |  |
|  | -2.1±22.4 | -6.3±23.8 |  |
|  | -1.0(-15.5 to 13.0) | -4.0(-21.0 to 7.0) | 0.117 |
| HDL cholesterol (mg/dL) | 152 | 136 |  |
|  | -2.4±6.3 | -2.7±7.1 |  |
|  | -2.0(-6.0 to 1.0) | -3.0(-7.0 to 2.0) | 0.540 |

Variables are shown as n (top), mean±SD (middle), median (interquartile range) (bottom).
