# Supplementary material for: Effects of preconception lifestyle intervention in infertile women with obesity: The FIT-PLESE randomized controlled trial
Source: PLoS Med. 2022 Jan 18;19(1):e1003883. doi: 10.1371/journal.pmed.1003883 (PMC8765626; doi:10.1371/journal.pmed.1003883)
Supplement: S3 Table — (DOCX) [file pmed.1003883.s004.docx]

**S3 Table. Pregnancy outcomes-per protocol analysis**

|  | **Standard Lifestyle** | **Intensive Lifestyle** | **Rate Ratio in Intensive Lifestyle Group (95% CI)** | **P value^a^** |
| --- | --- | --- | --- | --- |
| Good live birth | 29/151(19.2%) | 23/157(14.6%) | 0.76(0.46 to 1.26) | 0.286 |
| Live birth | 42/151(27.8%) | 38/157(24.2%) | 0.87(0.60 to 1.27) | 0.470 |
| Singleton live birth | 39/42(92.9%) | 32/38(84.2%) | 0.91(0.77 to 1.07) | 0.296 |
| Twin live birth | 3/42(7.1%) | 6/38(15.8%) | 2.21(0.59 to 8.23) | 0.296 |
| Pregnancy | 45/151(29.8%) | 48/157(30.6%) | 1.03(0.73 to 1.44) | 0.883 |
| Clinical pregnancy | 47/151(31.1%) | 52/157(33.1%) | 1.06(0.77 to 1.47) | 0.708 |
| Conception | 59/151(39.1%) | 63/157(40.1%) | 1.03(0.78 to 1.35) | 0.850 |
| Pregnancy loss among women who conceived | 14/59(23.7%) | 24/63(38.1%) | 1.61(0.92 to 2.80) | 0.087 |

Live birth was defined by the delivery of a live-born infant. Good live birth was defined by the delivery of a live birth of an infant born at ≥ 37 weeks, with a birth weight between 2500 and 4000g and without a major congenital anomaly. Conception was defined as having a rising serum level of human chorionic gonadotropin for two consecutive tests. Clinical pregnancy was defined by the observation of gestational sac on ultrasound. Pregnancy was defined by observation of fetal heart motion on ultrasonography. CI, confidence interval.

^a^ P value was calculated using Chi-square or Fisher’s exact test.
