# Supplementary material for: Effects of preconception lifestyle intervention in infertile women with obesity: The FIT-PLESE randomized controlled trial
Source: PLoS Med. 2022 Jan 18;19(1):e1003883. doi: 10.1371/journal.pmed.1003883 (PMC8765626; doi:10.1371/journal.pmed.1003883)
Supplement: S4 Table — (DOCX) [file pmed.1003883.s005.docx]

**S4 Table. Good live birth, live birth, and conception rates per study phase and treatment cycle**

|  | **Standard Lifestyle** | **Intensive Lifestyle** | **Rate Ratio (95% CI) in Intensive Lifestyle Group** | **P value^a^** |
| --- | --- | --- | --- | --- |
| Good live birth per cycle | | | | |
| Phase I | 12/191(6.3%) | 8/188(4.3%) | 0.68(0.28 to 1.62) | 0.377 |
| Phase II | 17/191 (8.9%) | 15/188 (8.0%) | 0.90 (0.46 to 1.74) | 0.747 |
| Phase II, cycle 1 | 4/174(2.3%) | 9/166(5.4%) | 2.36(0.74 to 7.51) | 0.163 |
| Phase II, cycle 2 | 5/154(3.2%) | 4/147(2.7%) | 0.84(0.23 to 3.06) | 1.000 |
| Phase II, cycle 3 | 8/146(5.5%) | 2/131(1.5%) | 0.28(0.06 to 1.29) | 0.108 |
| Live birth per cycle | | | | |
| Phase I | 13/191(6.8%) | 11/188(5.9%) | 0.86(0.40 to 1.87) | 0.703 |
| Phase II | 29/191 (15.2%) | 27/188 (14.4) | 0.95 (0.58 to 1.53) | 0.822 |
| Phase II, cycle 1 | 12/174(6.9%) | 17/166(10.2%) | 1.48(0.73 to 3.01) | 0.270 |
| Phase II, cycle 2 | 7/154(4.5%) | 7/147(4.8%) | 1.05(0.38 to 2.91) | 0.929 |
| Phase II, cycle 3 | 10/146(6.8%) | 3/131(2.3%) | 0.33(0.09 to 1.19) | 0.091 |
| Conception per cycle | | | | |
| Phase I | 17/191(8.9%) | 22/188(11.7%) | 1.31(0.72 to 2.40) | 0.369 |
| Phase II | 42/191 (22.0%) | 41/188 (21.8%) | 0.99(0.68 to 1.45) | 0.966 |
| Phase II, cycle 1 | 20/174(11.5%) | 19/166(11.4%) | 1.00(0.55 to 1.80) | 0.989 |
| Phase II, cycle 2 | 8/154(5.2%) | 16/147(10.9%) | 2.10(0.92 to 4.75) | 0.069 |
| Phase II, cycle 3 | 14/146(9.6%) | 6/131(4.6%) | 0.48(0.19 to 1.21) | 0.108 |

Variables are shown as no./total n (%). Phase I: 16 week Preconception Intervention; Phase II: Ovarian Stimulation with Clomiphene Citrate/ Intrauterine Insemination. CI, confidence interval.

^a^ P value was calculated using Chi-square or Fisher’s exact test.
