# Supplementary material for: Effects of preconception lifestyle intervention in infertile women with obesity: The FIT-PLESE randomized controlled trial
Source: PLoS Med. 2022 Jan 18;19(1):e1003883. doi: 10.1371/journal.pmed.1003883 (PMC8765626; doi:10.1371/journal.pmed.1003883)
Supplement: S5 Table — (DOCX) [file pmed.1003883.s006.docx]

**S5 Table. Serious Adverse Events (all) and Adverse Events (with more than 2% of patients experiencing them) between the intervention Groups**

|  | **Standard Lifestyle** | **Intensive Lifestyle** | **P value^a^** |
| --- | --- | --- | --- |
| **Before conception** | | | |
| **Serious adverse** | | | |
| Hospitalization | 0/191 | 2/188(1.1%) | 0.245 |
| Pelvic pain | 0/191 | 1/188(0.5%) | 0.496 |
| Appendicitis | 1/191(0.5%) | 0/188 | 1.000 |
| Pneumonia | 1/191(0.5%) | 0/188 | 1.000 |
| Pulmonary Embolism | 1/191(0.5%) | 0/188 | 1.000 |
| Complex cyst resulting in surgical intervention | 1/191(0.5%) | 0/188 | 1.000 |
| **Other adverse events** | | | |
| Abdominal bloating | 5/191(2.6%) | 13/188(6.9%) | 0.049 |
| Abdominal pain | 12/191(6.3%) | 20/188(10.6%) | 0.127 |
| Allergic rhinitis | 7/191(3.7%) | 5/188(2.7%) | 0.576 |
| Anxiety/irritability | 14/191(7.3%) | 11/188(5.9%) | 0.562 |
| Back pain | 20/191(10.5%) | 13/188(6.9%) | 0.220 |
| Bronchospasm | 2/191(1.0%) | 4/188(2.1%) | 0.446 |
| Constipation | 7/191(3.7%) | 23/188(12.2%) | 0.002 |
| Diarrhea | 9/191(4.7%) | 35/188(18.6%) | <0.001 |
| Dizziness | 5/191(2.6%) | 8/188(4.3%) | 0.381 |
| Dysmenorrhea | 25/191(13.1%) | 23/188(12.2%) | 0.802 |
| Dyspepsia | 8/191(4.2%) | 16/188(8.5%) | 0.084 |
| Fatigue | 13/191(6.8%) | 12/188(6.4%) | 0.868 |
| Fever | 0/191 | 5/188(2.7%) | 0.029 |
| Flatulence | 2/191(1.0%) | 33/188(17.6%) | <0.001 |
| Flu-like symptoms | 13/191(6.8%) | 9/188(4.8%) | 0.401 |
| Gastroenteritis | 8/191(4.2%) | 11/188(5.9%) | 0.458 |
| Headache | 47/191(24.6%) | 53/188(28.2%) | 0.429 |
| Hot flashes | 31/191(16.2%) | 24/188(12.8%) | 0.338 |
| hCG trigger injection site reaction | 5/191(2.6%) | 9/188(4.8%) | 0.263 |
| Insomnia | 3/191(1.6%) | 5/188(2.7%) | 0.500 |
| Joint/muscle pain | 19/191(9.9%) | 19/188(10.1%) | 0.959 |
| Mood swings | 9/191(4.7%) | 2/188(1.1%) | 0.062 |
| Nasopharyngitis | 36/191(18.8%) | 35/188(18.6%) | 0.954 |
| Nausea/vomiting | 24/191(12.6%) | 41/188(21.8%) | 0.017 |
| Oily stools/discharge | 0/191 | 43/188(22.9%) | <0.001 |
| Oral pain | 6/191(3.1%) | 5/188(2.7%) | 0.780 |
| Otitis media | 5/191(2.6%) | 3/188(1.6%) | 0.724 |
| Ovulation pain | 2/191(1.0%) | 5/188(2.7%) | 0.281 |
| Pelvic pain | 4/191(2.1%) | 6/188(3.2%) | 0.541 |
| Rash, unspecified | 5/191(2.6%) | 3/188(1.6%) | 0.724 |
| Upper respiratory infection | 30/191(15.7%) | 30/188(16.0%) | 0.947 |
| Vaginal infection | 7/191(3.7%) | 3/188(1.6%) | 0.337 |
| **After conception** | | | |
| **Serious adverse events-mother** | | | |
| Hospitalization during first trimester | 0/59 | 1/63(1.6%) | 1.000 |
| Ectopic pregnancy | 2/59(3.4%) | 1/63(1.6%) | 0.610 |
| Pregnancy of Unknown Location | 3/59(3.4%) | 3/63(4.8%) | 1.000 |
| Marginal Placenta Previa | 0/59 | 1/63(1.6%) | 1.000 |
| Placenta Previa and Pre-term birth | 1/59(1.7%) | 0/63 | 0.484 |
| Hospitalization | 1/59(1.7%) | 2/63(3.2%) | 1.000 |
| **Other adverse events-mother** | | | |
| Pre-term labor | 6/59(10.2%) | 2/63(3.2%) | 0.154 |
| Pre-eclampsia/Eclampsia | 7/59(11.9%) | 4/63(6.3%) | 0.352 |
| Hyperemesis | 3/59(5.1%) | 4/63(6.3%) | 1.000 |
| Gestational Diabetes | 10/59(16.9%) | 6/63(9.5%) | 0.225 |
| Incompetent cervix | 0/59 | 2/63(3.2%) | 0.496 |
| Premature rupture of membranes | 4/59(6.8%) | 2/63(3.2%) | 0.428 |
| Other complication | 3/59(5.1%) | 4/63(6.3%) | 1.000 |
| Placental abnormalities | 4/59(6.8%) | 5/63(7.9%) | 1.000 |
| Post-partum depression | 1/59(1.7%) | 1/63(1.6%) | 1.000 |
| Post-partum Infection | 2/59(3.4%) | 0/63 | 0.232 |
| Post-partum hemorrhage | 1/59(1.7%) | 0/63 | 0.484 |

|  | **Standard Lifestyle** | **Intensive Lifestyle** | **P value** |
| --- | --- | --- | --- |
| Other post-partum complication(s) | 1/59(1.7%) | 3/63(4.8%) | 0.620 |
| **Serious adverse events-fetus/infant** | | | |
| Hospitalization-infant | 2/42(4.8%) | 1/38(2.6%) | 1.000 |
| Myelomeningocele | 0/42 | 1/38(2.6%) | 0.475 |
| Neonatal death | 0/42 | 0/38 |  |
| Stillbirth | 0/42 | 0/38 |  |
| **Other adverse events-fetus/infant** | | | |
| Intrauterine growth restriction | 4/42(9.5%) | 1/38(2.6%) | 0.362 |
| Admission to NICU | 12/42(28.6%) | 7/38(18.4%) | 0.287 |
| Neonatal respiratory distress syndrome | 4/42(9.5%) | 2/38(5.3%) | 0.678 |
| Neonatal jaundice | 5/42(11.9%) | 6/38(15.8%) | 0.614 |
| Neonatal infection | 2/42(4.8%) | 2/38(5.3%) | 1.000 |
| Neonatal hospitalization > 3 days | 7/42(16.7%) | 6/38(15.8%) | 0.915 |
| Other complication of infant after delivery | 2/42(4.8%) | 0/38 | 0.495 |
| Birth defect | 1/42(2.4%) | 0/38 | 1.000 |

^a^ P value was calculated using Chi-square or Fisher’s exact test.
