# Supplementary material for: Effects of preconception lifestyle intervention in infertile women with obesity: The FIT-PLESE randomized controlled trial
Source: PLoS Med. 2022 Jan 18;19(1):e1003883. doi: 10.1371/journal.pmed.1003883 (PMC8765626; doi:10.1371/journal.pmed.1003883)
Supplement: S6 Table — (DOCX) [file pmed.1003883.s007.docx]

**S6 Table. Pregnancy loss of selected lifestyle intervention trials in women with infertility**

|  | **Control group** | **Intervention group** | **Rate Ratio (95% CI) in Intervention group** | **p value^e^** |
| --- | --- | --- | --- | --- |
| **Pregnancy loss among those who conceived** | | | | |
| Dutch Study^a^ | NA^c^ | NA |  |  |
| Swedish Study^b^ | 14/56 (25.0) | 21/66 (31.8) | 1.27(0.72 to 2.26) | 0.407 |
| FIT-PLESE Study | 14/59(23.7) | 24/63(38.1) | 1.61(0.92 to 2.80) | 0.087 |
| **Combined** | **28/115 (24.3)** | **45/129 (34.9)** | **1.43(0.96 to 2.14)** | **0.073** |
|  | | | | |
| **Miscarriage among those who were randomized**^d^ | | | | |
| Dutch Study | 27/284 (9.5) | 41/280 (14.6) | 1.54(0.98 to 2.43) | 0.061 |
| Swedish Study | 5/153 (3.3) | 8/152 (5.3) | 1.61(0.54 to 4.81) | 0.388 |
| FIT-PLESE Study | 3/191(1.6) | 13/188(6.9) | 4.40(1.28 to 15.20) | 0.011 |
| **Combined** | **35/628 (5.6)** | **62/620 (10.0)** | **1.79(1.20 to 2.67)** | **0.004** |

^a^ Dutch study: Mutsaerts et al, NEJM, 2016: https://[www.ncbi.nlm.nih.gov/pubmed/27192672](http://www.ncbi.nlm.nih.gov/pubmed/27192672)

. ^b^ Swedish study: Einarsson et al, Hum Reprod, 2017 https://[www.ncbi.nlm.nih.gov/pubmed/28854592.](http://www.ncbi.nlm.nih.gov/pubmed/28854592)

^c^ Dutch study did not track or report biochemical pregnancy losses

^d^ Miscarriage is the loss of a clinical pregnancy (in which the gestational sac was visualized on ultrasound).

^e^ P value was calculated using Chi-square or Fisher’s exact test.
