# Supplementary material for: Effects of preconception lifestyle intervention in infertile women with obesity: The FIT-PLESE randomized controlled trial
Source: PLoS Med. 2022 Jan 18;19(1):e1003883. doi: 10.1371/journal.pmed.1003883 (PMC8765626; doi:10.1371/journal.pmed.1003883)
Supplement: S7 Table — (DOCX) [file pmed.1003883.s008.docx]

**S7 Table. Pregnancy loss according to the time point of achieved conception (Phase I, cycle 1 in Phase II vs cycle 2, 3 in Phase II)**

|  | **Achieved conception in Phase I or 1^st^ cycle in Phase II** | **Achieved conception in 2^nd^ or 3^rd^ cycle in Phase II** | **P value^b^** |
| --- | --- | --- | --- |
| Pregnancy loss | 22/78 (28.2%) | 16/44 (36.4%) | 0.35 |
| Patients in Standard lifestyle arm only | 10/37 (27.0%) | 4/22 (18.2%)^a^ | 0.537 |
| Patients in Intensive lifestyle arm only | 12/41 (29.3%) | 12/22 (54.6%)^a^ | 0.049 |

Phase I: 16 week Preconception Intervention

Phase II: Ovarian Stimulation with Clomiphene Citrate/Intrauterine Insemination

^a^ p=0.027 for comparison of pregnancy loss between patients in standard lifestyle arm (18.2%, 4/22) and those is intensive lifestyle arm (54.6%, 12/22), for the conceptions that were achieved in 2^nd^ or 3^rd^ cycle in phase II.

^b^ P value was calculated using Chi-square or Fisher’s exact test.
