# Supplementary material for: Effects of preconception lifestyle intervention in infertile women with obesity: The FIT-PLESE randomized controlled trial
Source: PLoS Med. 2022 Jan 18;19(1):e1003883. doi: 10.1371/journal.pmed.1003883 (PMC8765626; doi:10.1371/journal.pmed.1003883)
Supplement: S8 Table — (DOCX) [file pmed.1003883.s009.docx]

**S8 Table. Good live birth, live birth, and conception according to male partner age**

|  | **Standard Lifestyle** | **Intensive Lifestyle** | **Rate Ratio (95% CI) in Intensive Lifestyle Group** | **P value^a^** | **P value for interaction^b^** |
| --- | --- | --- | --- | --- | --- |
| Good live birth | 29/191(15.2%) | 23/188(12.2%) | 0.81(0.48 to 1.34) | 0.404 |  |
| Male partner age <35 | 16/89(18.0%) | 16/114(14.0%) | 0.78(0.41 to 1.47) | 0.444 | 0.968 |
| Male partner age >=35 | 13/102(12.8%) | 7/73(9.6%) | 0.75(0.32 to 1.79) | 0.518 |  |
| Live birth | 42/191(22.0%) | 38/188(20.2%) | 0.92(0.62 to 1.36) | 0.672 |  |
| Male partner age <35 | 23/89(25.8%) | 23/114(20.2%) | 0.78(0.47 to 1.30) | 0.339 | 0.386 |
| Male partner age >=35 | 19/102(18.6%) | 15/73(20.6%) | 1.10(0.60 to 2.02) | 0.752 |  |
| Conception | 59/191(30.9%) | 63/188(33.5%) | 1.08(0.81 to 1.45) | 0.585 |  |
| Male partner age <35 | 33/89(37.1%) | 37/114(32.5%) | 0.88(0.60 to 1.28) | 0.492 | 0.125 |
| Male partner age >=35 | 26/102(25.5%) | 26/73(35.6%) | 1.40(0.89 to 2.20) | 0.148 |  |

Variables are shown as no./total n (%). CI, confidence interval.

^a^ P value was calculated using Chi-square or Fisher’s exact test.

^b^ P value was calculated using logistic regression model.
