# Supplementary figures and images for: Effects of preconception lifestyle intervention in infertile women with obesity: The FIT-PLESE randomized controlled trial
Source: PLoS Med. 2022 Jan 18;19(1):e1003883. doi: 10.1371/journal.pmed.1003883 (PMC8765626; doi:10.1371/journal.pmed.1003883)

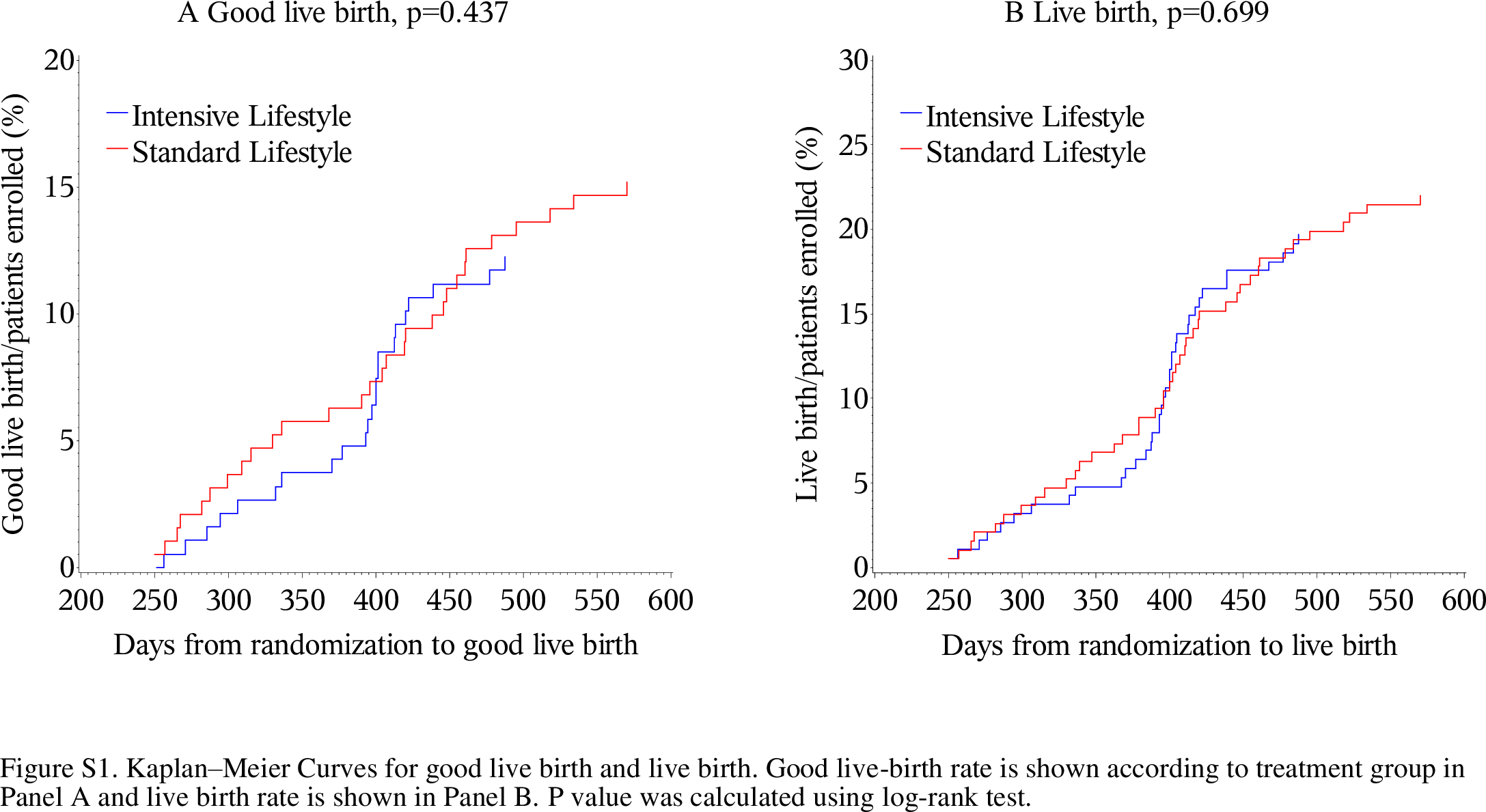

Supplement: S1 Fig — Good live birth rate is shown according to treatment group in panel A, and live birth rate is shown in panel B. (TIF) [file pmed.1003883.s010.tif]

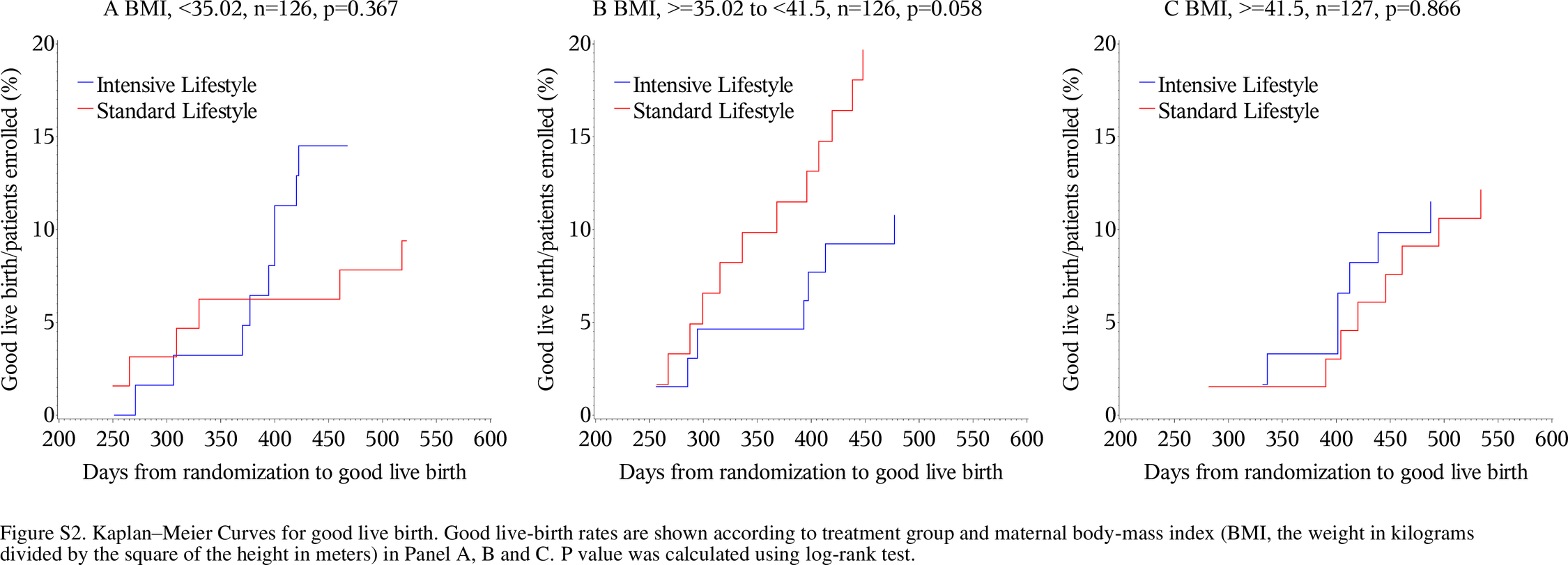

Supplement: S2 Fig — Good live birth rates are shown according to treatment group and maternal BMI (the weight in kilograms divided by the square of the height in meters) in panels A, B, and C. BMI, body mass index. (TIF) [file pmed.1003883.s011.tif]

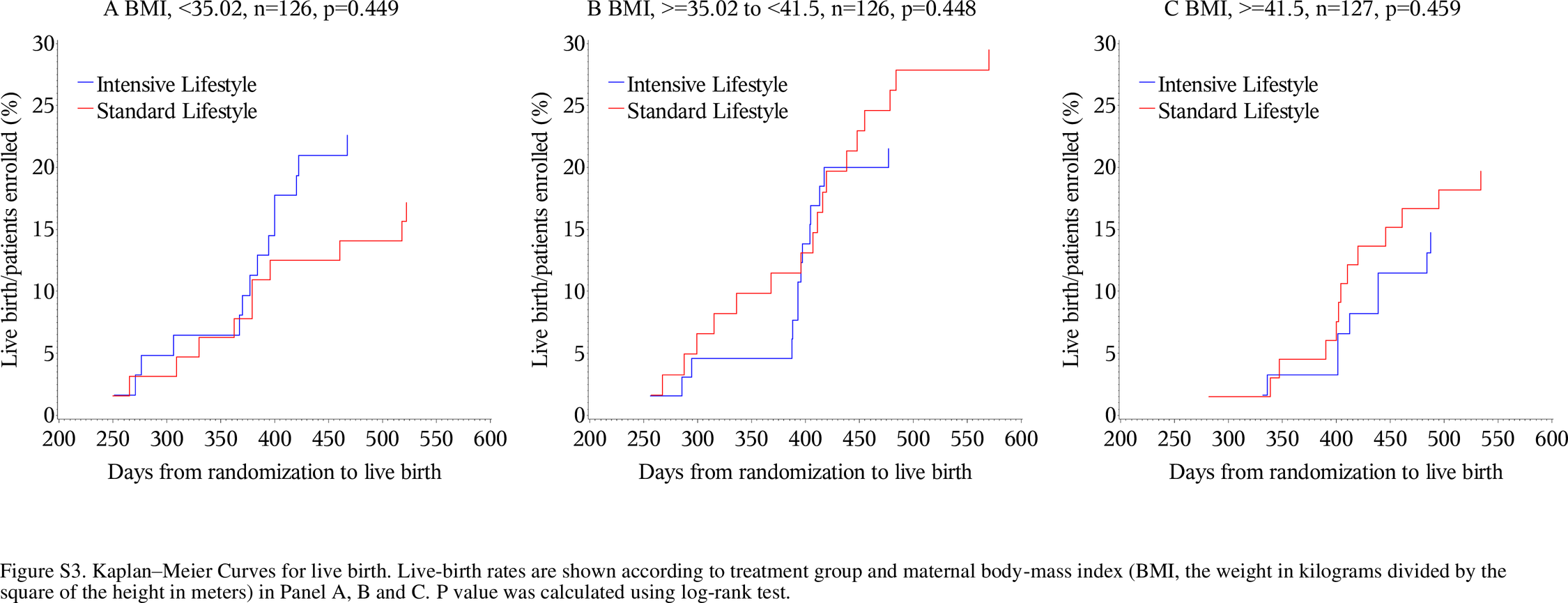

Supplement: S3 Fig — Live birth rates are shown according to treatment group and maternal BMI (the weight in kilograms divided by the square of the height in meters) in panels A, B, and C. BMI, body mass index. (TIF) [file pmed.1003883.s012.tif]

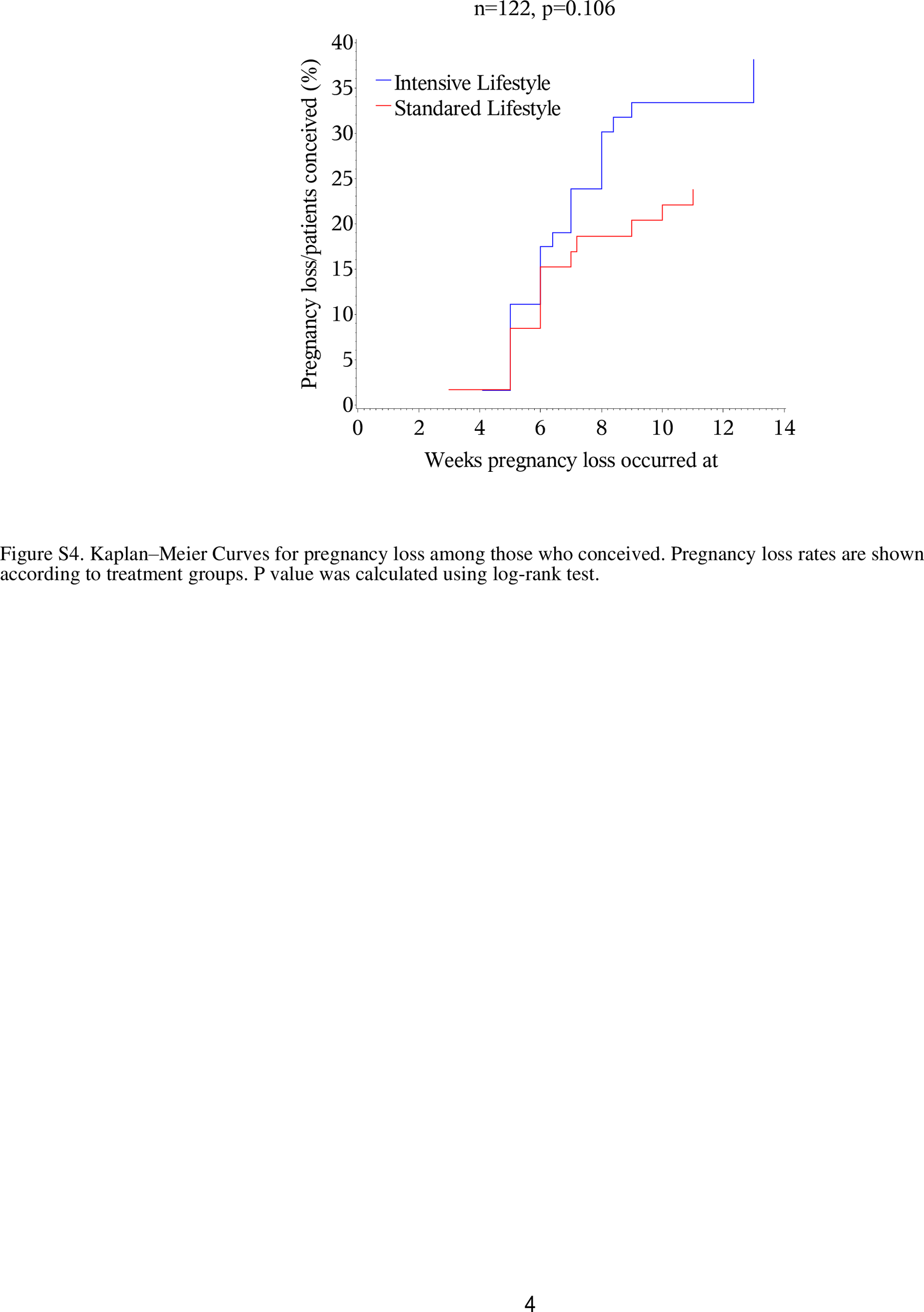

Supplement: S4 Fig — Pregnancy loss rates are shown according to treatment groups. (TIF) [file pmed.1003883.s013.tif]

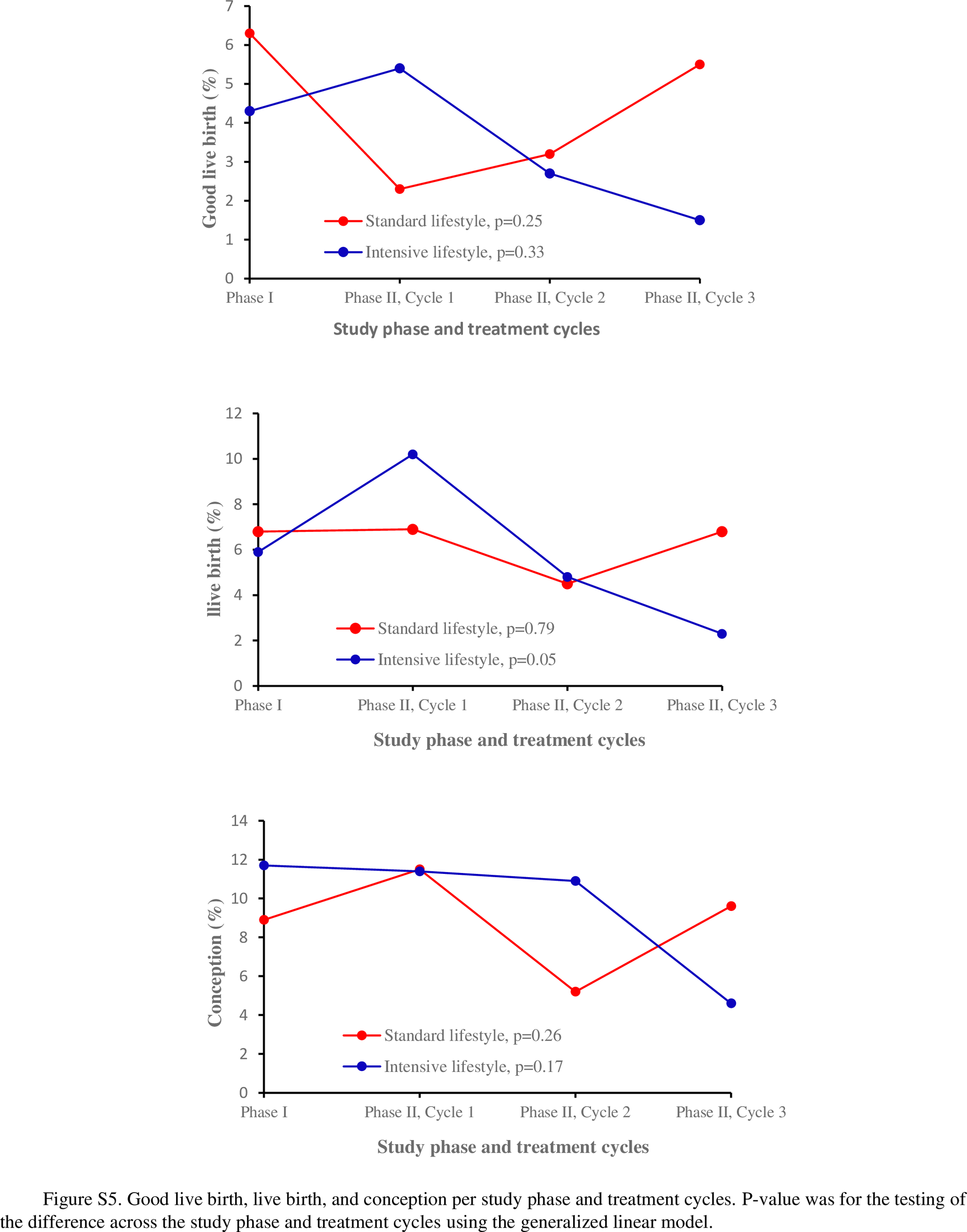

Supplement: S5 Fig — P value was for the testing of the difference across the study phase and treatment cycles using the generalized linear model. (TIF) [file pmed.1003883.s014.tif]
